# Comparative Analysis, Structural Insights, and Substrate/Drug Interaction of CYP128A1 in *Mycobacterium tuberculosis*

**DOI:** 10.3390/ijms21144816

**Published:** 2020-07-08

**Authors:** Nokwanda Samantha Ngcobo, Zinhle Edith Chiliza, Wanping Chen, Jae-Hyuk Yu, David R. Nelson, Jack A. Tuszynski, Jordane Preto, Khajamohiddin Syed

**Affiliations:** 1 Department of Biochemistry and Microbiology, Faculty of Science and Agriculture, University of Zululand, KwaDlangezwa 3886, South Africa; mskwandosamn@gmail.com (N.S.N.); zinhlechiliza01@gmail.com (Z.E.C.); 2 Department of Molecular Microbiology and Genetics, University of Göttingen, 37077 Göttingen, Germany; chenwanping1@foxmail.com; 3 Department of Bacteriology, University of Wisconsin-Madison, 3155 MSB, 1550 Linden Drive, Madison, WI 53706, USA; jyu1@wisc.edu; 4 Department of Systems Biotechnology, Konkuk University, Seoul 05029, Korea; 5 Department of Microbiology, Immunology and Biochemistry, University of Tennessee Health Science Center, Memphis, TN 38163, USA; drnelson1@gmail.com; 6 Department of Physics and Department of Oncology, University of Alberta, Edmonton, AB T6G 2E1, Canada; jack.tuszynski@gmail.com; 7 Department of Mechanical and Aerospace Engineering, Politecnico di Torino, Corso Duca degli Abruzzi, 24, 10129 Torino TO, Italy; 8 Université Claude Bernard Lyon 1, INSERM 1052, CNRS 5286, Centre Léon Bérard, Centre de Recherche en Cancérologie de Lyon, 69622 Lyon, France

**Keywords:** cytochrome P450 monooxygenenases, CYP128A1, *Mycobacterium tuberculosis* H37Rv, tuberculosis, molecular dynamic simulations, azole drugs, menaquinone

## Abstract

Cytochrome P450 monooxygenases (CYPs/P450s) are well known for their role in organisms’ primary and secondary metabolism. Among 20 P450s of the tuberculosis-causing *Mycobacterium tuberculosis* H37Rv, CYP128A1 is particularly important owing to its involvement in synthesizing electron transport molecules such as menaquinone-9 (MK9). This study employs different *in silico* approaches to understand CYP128 P450 family’s distribution and structural aspects. Genome data-mining of 4250 mycobacterial species has revealed the presence of 2674 *CYP128* P450s in 2646 mycobacterial species belonging to six different categories. Contrast features were observed in the *CYP128* gene distribution, subfamily patterns, and characteristics of the secondary metabolite biosynthetic gene cluster (BGCs) between *M. tuberculosis complex* (MTBC) and other mycobacterial category species. In all MTBC species (except one) CYP128 P450s belong to subfamily A, whereas subfamily B is predominant in another four mycobacterial category species. Of CYP128 P450s, 78% was a part of BGCs with *CYP124A1*, or together with *CYP124A1* and *CYP121A1*. The CYP128 family ranked fifth in the conservation ranking. Unique amino acid patterns are present at the EXXR and CXG motifs. Molecular dynamic simulation studies indicate that the CYP128A1 bind to MK9 with the highest affinity compared to the azole drugs analyzed. This study provides comprehensive comparative analysis and structural insights of CYP128A1 in *M. tuberculosis*.

## 1. Introduction

Tuberculosis (TB), caused by *Mycobacterium tuberculosis* H37Rv, remains a serious public health problem despite the existence of international TB control programs [1]. Recent data from the World Health Organization (WHO) shows that about 10 million people fell ill with TB in 2018 [1]. TB’s global threat to human health has been exacerbated in recent years by the emergence of widespread multi- and extensively drug-resistant *M. tuberculosis* strains [1]. The developing countries of South-East Asia and Africa have shown high incidence rates of TB. The prevalence of the disease in these countries is mainly due to lack of basic sanitation (causing an increase in transmission of the disease), human immunodeficiency virus (HIV) infection and lack of drugs to treat the disease [1]. Recent statistics from South Africa revealed that TB is the major killer among infectious diseases, indicating that this disease is still a major challenge in the country [2].

In 1998, determination of the *M. tuberculosis* H37Rv genome sequence encouraged more investigation of new anti-tubercular drugs and the seeking of more knowledge on the complex biology of the *M. tuberculosis* bacterium [3]. This highlighted the importance of lipid metabolism in *M. tuberculosis*; novel biosynthetic pathways were found to be involved in the synthesis of compounds such as phenolphthiocerol, mycolic acids and mycocerosic acid for the complex cell wall structure of the bacterium [4]. Among the enzymes involved in lipid metabolism, cytochrome P450 monooxygenases (CYPs/P450s) in *M. tuberculosis* were found to play a key role in the metabolism of lipids [5,6]. P450s are heme-thiolate proteins found in all species across biological domains [7]. Recent studies revealed the presence of a large number of P450s in mycobacterial species and most of these P450s were found to be involved in lipid metabolism [6]. *M. tuberculosis* H37Rv has 20 P450s in its genome and some of these P450s are indeed involved in lipid metabolism [5]. Furthermore, one of the P450 genes, namely *CYP125A1,* was used as a key factor in determining the cholesterol degrading ability of mycobacterial species [8].

Among *M. tuberculosis* H37Rv P450s, CYP128A1 gained particular interest among researchers owing to its history indicating its essentiality and its physiological importance. Transposon site hybridization mutagenesis studies indicated that *CYP128A1* is essential for in vitro survival of *M. tuberculosis* H37Rv [9]. Interestingly, another study, which used a similar approach, revealed that *CYP128A1* is not essential for survival of *M. tuberculosis* CDC1551 [10]. However, this study had a backdrop of limited gene coverage in its mutant library. In Vitro *M. tuberculosis* H37Rv latency model studies including a carbon starvation model [11] and hypoxia model [12] showed up-regulation of *CYP128A1*, suggesting that this P450 has a potential role in *M. tuberculosis* latency. Until 2016, the nature of *CYP128A1* with respect to its essentiality remained a mystery. Research revealed that *CYP128A1* is non-essential for survival of *M. tuberculosis* H37Rv as the *CYP128A1* gene knock-out strain survives [13]. However, the *CYP128A1* gene knock-out strain has proven to be hyper-virulent [13], indicating this gene actually playing a role in the synthesis of a compound that acts as a negative regulator of virulence.

Heterologous expression of *CYP128A1* posed a great challenge to researchers, as the expression of this particular P450 in *Escherichia coli* has been unsuccessful [14,15]. Genomic analysis revealed that *CYP128A1* is part of an operon that consists of two other genes, *stf3* and *rv2269c* [16]. In Vivo studies using *M. smegmatis* as model strain demonstrated that CYP128A1 is involved in hydroxylation of menaquinone-9 (MK9) and is essential in the synthesis of this compound, whereas Stf3 was found to introduce the sulfate group to menaquinone-9 and *rv2269c* was found to act as a promoter [13]. The sequence of reaction is that CYP128A1 introduces the hydroxyl group into MK9, followed by the addition of the sulfate group by the Stf3 that leads to the synthesis of sulfomenaquinone [13].

Lipoquinones are electron transport molecules that are involved in the respiratory function of bacteria and mainly consist of menaquinone and ubiquinone [17]. Menaquinones (2-methyl-3-polyprenyl-1,4-naphthoquinones) especially MK9 is ubiquitous and unique to mycobacteria [17], indicating that *CYP128A1* should be present in all mycobacterial species. However, to date, the distribution of *CYP128A1* in such a large number of mycobacterial species belonging to six different mycobacterial categories, i.e., *Mycobacterium tuberculosis* complex (MTBC), *M. chelonae-abscessus* complex (MCAC), *M. avium* complex (MAC), mycobacteria causing leprosy (MCL), non-tuberculosis mycobacteria (NTM) and saprophytes (SAP) is still unknown. Anti-fungal azole drugs were shown to be promising new tools to fight TB, particularly as they showed high antimycobacterial activity [18,19,20] and interestingly, to date, characterized *M. tuberculosis* P450s have been found to bind quite a number of azole drugs [21], leading to *M. tuberculosis* P450s becoming the main focus as novel drug targets against this pathogen [5]. The binding of azole drugs to CYP128A1 could have an undesired effect, as this could lead to possible disruption of enzyme function, a vital component in the virulence modulation of *M. tuberculosis*.

To date, genome-wide analysis of *CYP128* P450s has only been carried out in 60 mycobacterial species [22] and because of the failure of *CYP128A1* heterologous expression [14,15], analysis of binding patterns of azole drugs to CYP128A1 has not been performed. To address these research gaps, in this study, genome-wide data mining, annotation, and phylogenetic analysis of CYP128A1 were carried out in 4250 mycobacterial species. Furthermore, *in silico* analysis of binding of different azole drugs with the CYP128A1 model was assessed. The results for CYP128A1 were discussed in the context of gaining more knowledge on the role of this P450 in mycobacterial species.

## 2. Results and Discussion

### 2.1. Presence of CYP128 P450s in Five of the Six Mycobacterial Category Species

**Figure 1:**
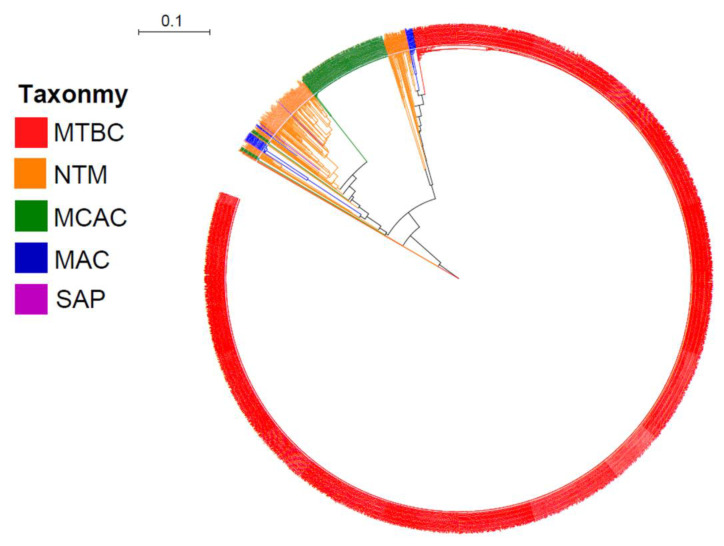
Phylogenetic tree of the cytochrome P450 monooxygenases (CYPs/P450s). Different mycobacterial categories are indicated indifferent colors. Abbreviations: MTBC, *Mycobacterium tuberculosis* complex; NTM, non-tuberculosis mycobacteria; MCAC, *Mycobacterium chelonae-abscessus* complex; MAC, *M. avium* complex and SAP, Saprophytes. A high-resolution phylogenetic tree is provided in Supplementary Dataset 3.

**Figure 2:**
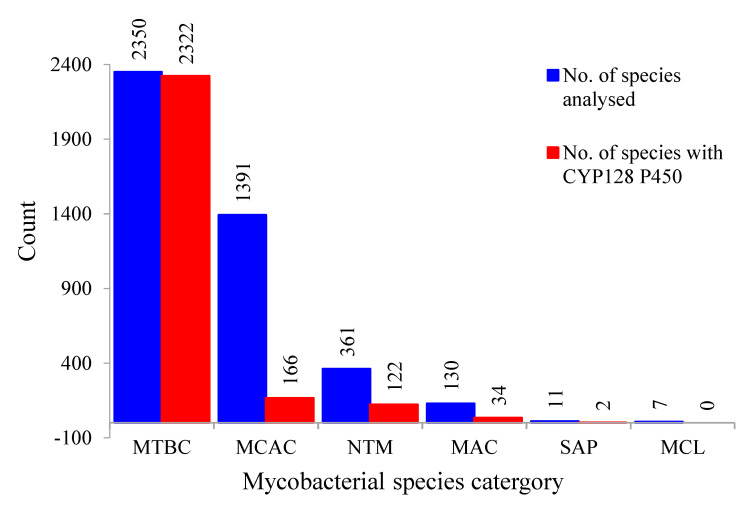
Comparative analysis of the CYP128 P450s in six different mycobacterial categories. Abbreviations: MTBC, *Mycobacterium tuberculosis* complex; NTM, non-tuberculosis mycobacteria; MCAC, Mycobacterium chelonae-abscessus complex; MAC, *M. avium* complex; SAP, saprophytes and MCL, mycobacteria causing leprosy. Information on mycobacterial species and CYP128 P450s is presented in Supplementary Dataset 1.

Comprehensive comparative analysis of *CYP128* P450s in 4250 mycobacterial species belonging to six different categories (MTBC, MCAC, MAC, MCL, NTM and SAP) revealed that this P450 family is present in 2646 mycobacterial species (Figure 1 and Figure 2; Supplementary Dataset 1). Thirty-seven mycobacterial species were found to have short P450 sequences that have none or only one of the highly conserved P450 motifs, i.e., EXXR and CXG (Supplementary Dataset 1). Thus, these short sequences are not included in the study and the species are considered not to have *CYP128* P450. Among mycobacterial species that were used in this study, most of the species (99%) belonging to the MTBC category have this P450 (Figure 2). Among 2350 MTBC species only 38 species do not have *CYP128* (Figure 2). In contrast to MTBC species, most of the species belonging to another five mycobacterial categories do not have this P450 (Figure 2). CYP128 P450s were found in only 34% of NTM species, followed by 26% of MAC species, 12% of MCAC species and 18% of SAP species. The results observed in this study revealed that none of the MCL species has CYP128 P450s, which is consistent with previous observations that MCL species do not have this P450 [22]. Overall, based on the results of this study and comparison with the results reported earlier [22], we conclude that *CYP128* P450s can be found in species belonging to the five mycobacterial categories except in species of the MCL category. Most of the CYP128 P450s (81%) are 489 amino acids in length (Supplementary Dataset 1). An anomaly was observed that two CYP128 P450 sequences, one from *M. tuberculosis* BTB10-120 and the other from *M. tuberculosis* SIT745/EAI1-MYS, has 877 amino acids. However, analysis of genome sequences revealed that *CYP128* and the sulfotransferase (*stf3*) genes were present next to each other on different DNA strands, indicating an error in annotation leading to the fused protein. A single copy of the *CYP128* gene was found in almost all species, except for 25 species where two copies (22 species), three copies (two species) and four copies (one species) of this gene were found (Supplementary Dataset 1). The species with more than one copy of this P450 gene were found across four different categories, where eight species were from MTBC, 12 species from NTM, three species from MAC and two species from MCAC (Supplementary Dataset 1). CYP128 P450 sequences identified in the study are presented in Supplementary Dataset 2.

### 2.2. Different Mycobacterial Category Species Have Different CYP128 Subfamily Preferences

**Figure 3:**
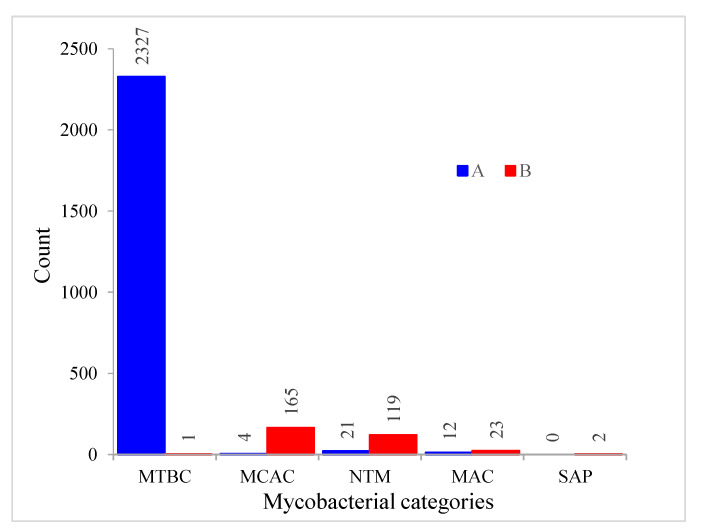
Comparative analysis of CYP128 P450 subfamilies in five different mycobacterial categories. Abbreviations: MTBC, *Mycobacterium tuberculosis* complex; NTM, non-tuberculosis mycobacteria; MCAC, *Mycobacterium chelonae-abscessus* complex; MAC, *M. avium* complex; and SAP, Saprophytes. Information on mycobacterial species and CYP128 P450s is presented in Supplementary Dataset 1.

CYP128 subfamily analysis revealed the presence of a new CYP128 subfamily in mycobacterial species. Thus, at present two CYP128 subfamilies can be found in mycobacterial species, i.e., A and B (Figure 3). Phylogenetic analysis revealed an interesting feature in CYP128 subfamilies with respect to their categories (Figure 1). In a previous study, it was observed that P450s belonging to different mycobacterial categories grouped together according to their category, indicating high conservation of P450 protein sequences after speciation into different categories [22]. In this study, the same phenomenon was observed for CYP128 subfamilies A and B, as these subfamily proteins were grouped together according to their mycobacterial categories, despite the subfamilies being clearly separated on the tree (Figure 1). Also, contrasting subfamily features were observed among different mycobacterial categories (Figure 3). Except for one species, *M. tuberculosis* XTB13-223, all species belonging to the MTBC category have CYP128 subfamily A (Figure 3). However, in contrast to MTBC species, in other mycobacterial categories subfamily B was dominantly present (Figure 3). This indicates that during evolution mycobacterial species had both subfamilies, but owing to their lifestyles or ecological niches only one subfamily was favored. This phenomenon of enriching a particular type of P450 family or subfamilies in microbial species is well known [6,22,23,24,25,26,27,28]. An interesting pattern of subfamilies was found in species having more than one copy of the *CYP128* gene. Eight MTBC species have two copies of the *CYP128* gene, both belonging to the same subfamily A. However, in other category species subfamilies A and B are present; especially in species belonging to the NTM category, subfamily B is populated (Supplementary Dataset 1).

### 2.3. CYP128 Family Ranked Fifth among P450 Families

It has been proposed that the present-day P450s are evolved from the ancient P450 CYP51 [29,30]. Evolution of different P450 families and their rate of evolution indicate that the higher the evolutionary rate, the more catalytically diverse they are [6,22,24]. Parvez and co-workers [22] proposed a ranking system for P450 families where different P450 families are given a rank based on the number of conserved amino acids and it was proposed that the higher the conservation, the less the catalytic diversity. Furthermore, a recent study revealed that better ranking of P450 families can be achieved using a larger sample size [31]. In a previous study, only 49 CYP128 P450s were used to predict the ranking [22]. Identification of quite a large number of CYP128 P450s in this study necessitates re-assessment of ranking, in order to calculate the accurate number of conserved amino acids in P450s, as it was mentioned that P450s with similar amino acid length should be used [22,25,31,32]. Thus, in this study, 2191 CYP128 P450s with amino acid lengths ranging from 479 to 489 amino acids (Supplementary Dataset 1) were used to assess the conservation ranking of the CYP128 P450 family. PROfile Multiple Alignment with Predicted Local Structures and 3D Constraints (PROMALS3D) analysis [33] revealed the presence of 217 amino acids that are invariantly conserved in CYP128 P450s (Table 1). Comparison with other P450 families from different biological kingdoms placed CYP128 family in the fifth position, compared to 23rd position previously (Table 1), indicating that this P450 family is one of the best conserved families. A complete table with updated P450 family ranking is presented in Table S1.

### 2.4. CYP128 Family Has Distinctive Amino Acid Patterns at EXXR and CXG Motif

**Figure 4:**
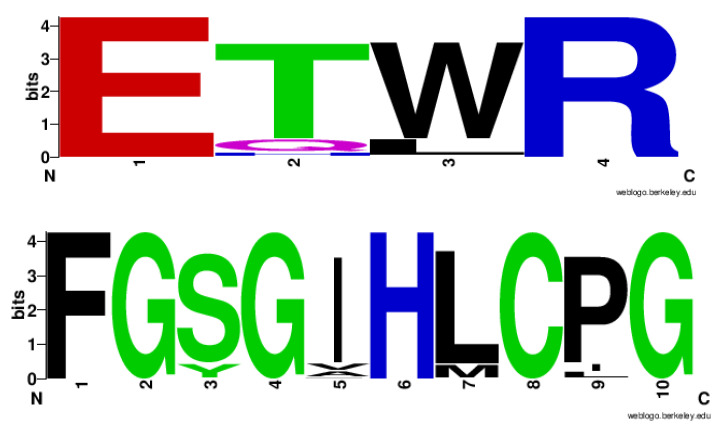
Analysis of amino acid patterns at the EXXR and CXG motif in the CYP128 P450 family. All CYP128 P450 sequences (total of 2674 sequences) were analyzed for the EXXR and CXG signature motifs.

A study of the analysis of P450 motifs EXXR and CXG revealed that all P450 families have a unique amino acid pattern that serves as a signature characteristic of that particular P450 family [34]. Subsequent studies of P450 families strongly supported this hypothesis [25,31,32]. Analysis of the EXXR and CXG P450 motifs in the CYP128 family has not been performed, and the large number of CYP128 P450s identified in this study provides an opportunity to analyze the amino acid patterns at the EXXR and CXG-motifs for this family. Analysis of the amino acids at the EXXR motif indicated the presence of E-T(84%)/Q(11%)/H(1%) -W(84%)/L(15%)-R amino acid patterns at this motif, with most of the CYP128 P450s (84%) having the E-T-W-R amino acid pattern (Figure 4). Analysis of amino acid patterns at the CXG motif revealed the presence of F-G-S(88%)/Y(12%) -G-I(88%)/V(6%)/A(5%)/P(1%) -H-L(89%)/M(11%) -C-P(87%)/I(7%)/L(6%) -G amino acid patterns, with the majority of the CYP128 P450s (86%) containing the F-G-S-G-I-H-L-C-P-G amino acid patterns (Figure 4). Amino acids patterns at the EXXR and CXG motifs of the CYP128 family were found to be unique compared to P450 families from different biological kingdoms [26,31,32,34], further supporting the hypothesis that amino acid patterns at these motifs are a signature of the P450 family [34].

### 2.5. Most CYP128 P450s Exist in Secondary Metabolite Biosynthetic Gene Clusters

P450s are well known to play a key role in the synthesis of different secondary metabolites [35,36] and a recent study undertaking comparative analysis of secondary metabolite biosynthetic gene clusters (BGCs) between 48 *Streptomyces* species and 60 mycobacterial species revealed that *CYP128* P450s are part of a secondary metabolite BGC [6]. The *CYP128* P450 BGCs were found to have one or two other P450s in the cluster and the *CYP128* P450 is always found with CYP124A1 or together with *CYP124A1* and *CYP121A1* P450s [6]. In this study, secondary metabolite BGC analysis revealed that 78% of CYP128 P450s were found to be part of secondary metabolite BGCs; 2090 *CYP128* P450s from 2674 *CYP128* P450s were found to be part of secondary metabolite BGCs. Analysis of cluster types revealed 1994 *CYP128* P450 clusters belonging to the tRNA-dependent cyclodipeptide synthases (CDPS) cluster type, followed by 94 having no cluster types, indicating a novel BGC cluster; one belongs to Type I Polyketide synthase (T1PKS) and the last one belongs to the non-ribosomal peptide synthetase (NRPS) cluster type (Supplementary Dataset 1). In 25 species with two or more *CYP128* P450s, four species (*M. tuberculosis* KI_19771, *M. tuberculosis* 402267, *M. canettii* CIPT 140070010, and *M. canettii* CIPT 140010059) were found to have all their *CYP128* P450s as part of a cluster type, three species (*M. tuberculosis* BTB10-253, *M. tuberculosis* M1415, and *Mycobacterium* sp. GA-1199) had only one and the rest of the species had no *CYP128* P450s as part of any cluster types (Supplementary Dataset 1).

An interesting contrast pattern was observed when comparing *CYP128* P450 BGCs among different mycobacterial category species (Table 2). Most of the CYP128 P450s were found to be part of BGCs in MTBC species, whereas only a handful of CYP128 P450s were part of BGCs in species belonging to the categories MCAC, NTM, and MAC (Table 2). Furthermore, most BGCs of MTBC species have three P450s, *CYP128*, *CYP121,* and *CYP124,* followed by BGCs with *CYP128* P450 and BGCs with *CYP128* and *CYP124* P450s (Table 2). Interestingly, the remaining four mycobacterial category species BGCs have only *CYP128* P450 (Table 2). None of the BGCs from all five different mycobacterial categories has the combination of P450s, *CYP128,* and *CYP121* (Table 2).

### 2.6. CYP128A1 Protein Model Has High Affinity to Menaquinone-9

**Figure 5:**
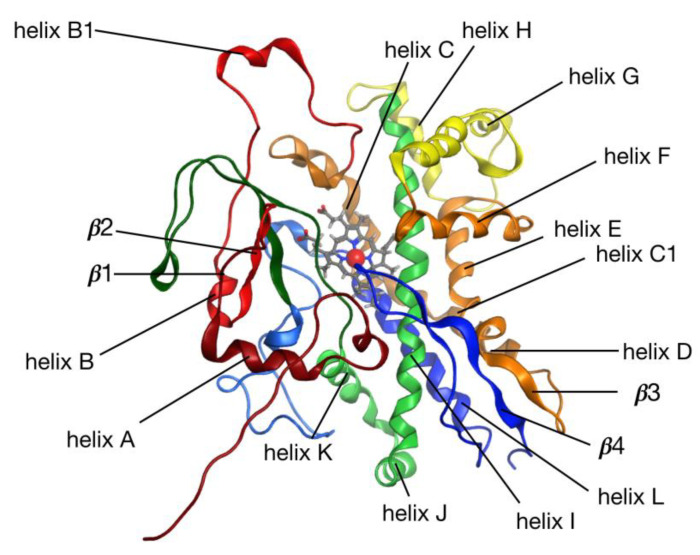
A 3D model of CYP128A1 with structured regions labeled. Heme and the iron atom are shown in grey and red, respectively.

Since the expression of *CYP128A1* in heterologous hosts such as *E. coli* was found to be difficult [14,15] and at present there is no scope to generate the CYP128A1 crystal structure, in this study a 3D model of CYP128A1 was built to explore its binding affinity with MK9 and different azole drugs. The CYP128A1 model was built using the structure of Vitamin D3 hydroxylase (Vdh) from *Pseudonocardia autotrophica* as a template (PDB ID: 3VRM, 33.8% identity) [37]. The model was optimized using molecular-dynamics (MD) simulations (Figure 5) as described in the subsection on Materials and Methods. Notably, other models based on templates sharing similar coverage and identity compared to 3VRM were tested. Other structures include 1Z8P (32.4% identity) [38], 3A4G (33.5% identity) [39] and 5GNM (33.6% identity) [40]. Although some of these structures have a bit better resolution than 3VRM, they either correspond to apo structures (5GNM) or structures bound to small ligands (1Z8P and 3A4G), leaving the catalytic site “packed up” and thus inaccessible through docking simulations. This was confirmed by visual inspection together with our inability to generate binding poses in the catalytic site when docking on models based on 5GNM or 3A4G templates (results not shown). Conversely, 3VRM corresponds to the structure of Vdh mutant bound with Vitamin D3 where the catalytic site is open enough to enable the binding of ligands as big as MK9. As a result, docking simulations on this model were successful in finding binding poses for all the ligands tested.

Homology modeling was carried out using the Molecular Operating Environment (MOE) software as described in the Materials and Methods section. Importantly, sequence alignment did not reveal any major indel regions compared to our target sequence (see Figure S1). We reported a five-residue loop as the most significant insertion with a fairly large distance to the catalytic site (23.5 Å to the ferric ion of the HEME group) and a seven-residue gap as the most important deletion. During our protocol, 10 intermediate models were generated independently. For every model, the RMSD (root-mean-square deviation) to the mean conformation was calculated and a low standard deviation (STD) to the mean structure was obtained (STD =2.654 Å for residues in the five-residue insertion, STD=0.102 Å for residues on both sides of the seven-residue deletion, STD=0.021 Å for the whole structure), suggesting good convergence and a limited number of possible variations in the target model. Our final CYP128A1 model (Figure 5), selected based on MOE’s built-in score, was used as a target in a first round of docking simulations. For each compound, the 10 top docked complexes were then equilibrated through MD (six complexes in the case of MK9, see Materials and Methods section). Next, MK9 and azole drugs were removed and re-docked independently onto each of their corresponding equilibrated receptors. In Table 3, we reported the results of our re-docking procedure with top scores (in kcal/mol) listed in column 2. The order of binding was as follows: MK9 > itraconozole > posaconazole > ketoconazole > econazole > miconzaole > voriconazole > clotrimazole > fluconazole (Table 2).

In P450 enzyme systems, substrates and azole drugs are known to coordinate with the ferric core of the heme group. In the case of MK9, coordination takes place at the end of its hydrophobic tail, consistent with omega hydroxylation of the molecule [13,16]. For azole drugs, coordination occurs via the imidazole ring. Importantly, docking of MK9 and azole drugs was performed in a non-covalent way in the present paper. Therefore, no coordination was predicted for the tested ligands, which would require more thorough investigation, especially considering changes in electronic density. However, since non-covalent binding is a critical step in the establishment of covalent interactions, we believe the present results still provide a good indication of how azoles drugs can interact with CYP128A1 and how they rank in terms of affinity.

**Figure 6:**
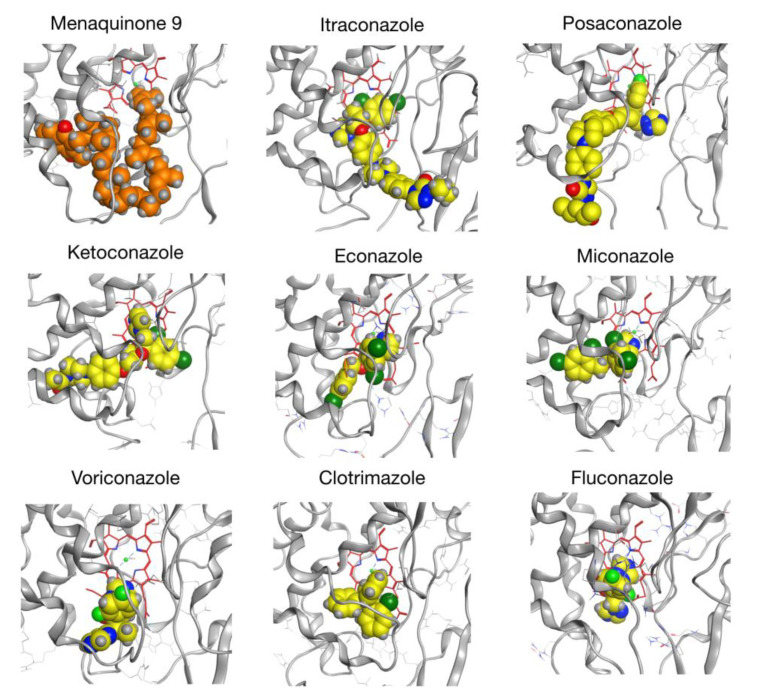
First well-oriented binding poses obtained for a substrate (menaquinone 9) and the azole compounds. The substrate/azole compounds are shown in orange/yellow, respectively, while the heme is shown in red and the ferric core is depicted in light green.

Column 3 in Table 3 shows the score of the first well-oriented pose, i.e., where the end of the hydrocarbon tail (MK9) or the imidazole ring (azole drugs) faces the ferric core of the heme. Interestingly, considering well-oriented poses does not significantly affect the ranking of compounds. Column 4 shows the rank of the first well-oriented pose over all the poses generated for each ligand. While top-ranked poses are already well oriented in some cases (posaconazole, miconazole, voriconazole, fluconazole), well-oriented poses of other compounds such as itraconazole and ketoconazole exhibit lower ranking (10 and 9, respectively). Nonetheless, we observed that at least one pose among the top 10 is well oriented for each compound, leading to a score value similar to the top-ranked pose in each case. We depicted the first well-oriented poses of each compound in Figure 6.

In summary, *in silico* results indicated that the CYP128A1 indeed binds to the substrate (MK9) with highest affinity compared to the azole drugs analyzed in the study (Table 3). Among azole drugs itraconazole, posaconazole and ketoconazole have the highest binding affinity with CYP128A1. One possible reason for the high binding affinity of these compounds compared to other azoles is that these molecules are more extended depicting the longer side chain of MK9. A point to be noted that same phenomenon of better interactions of azoles due to their more extended structures was also observed for CYP51 of *Sporothrix schenckii* [41]. CYP128A1 binding to different azole drugs is also consistent with other *M. tuberculosis* H37Rv P450s that are experimentally shown to bind azole drugs albeit with different affinities [21].

## 3. Materials and Methods

### 3.1. Species and Databases

A total of 4250 mycobacterial species genomes (permanent draft) available at the Joint Genome Institute (JGI)/Integrated Microbial Genomes and Microbes (IMG/M) database (from February 2019 to March 2019) were used in this study [42]. Species were grouped in six different mycobacterial categories, as described elsewhere [22,31]. The six categories included MTBC (2350 species), MCAC (1391 species), MAC (130 species), MCL (7 species), NTM (361 species), and SAP (11 species). Mycobacterial species along with their genome IDs and their mycobacterial categories were presented in Supplementary Dataset 1.

### 3.2. Genome Data Mining and Annotation of CYP128 P450s

CYP128 P450s mining in different bacterial species was carried out following the method described elsewhere [22,31]. Briefly, BLAST analysis was performed with the *M. tuberculosis* H37Rv CYP128A1 (*Rv2268c*) P450 sequence with default settings against individual mycobacterial species genomes at the IMG/M database. Considering the International Cytochrome P450 Nomenclature criteria, i.e., P450s showing > 40% identity belong to the same family [43,44,45], all the hit proteins with more than 40% identity were selected and subjected to P450 characteristic motifs analysis as described elsewhere [34,46,47]. Proteins that showed all P450 characteristic motifs were selected for further analysis. Proteins that were short in amino acid length and had none or only one of the highly conserved P450 motifs, such as EXXR and CXG, were considered P450 fragments and not included in the study. The selected proteins were then subjected to BLAST analysis at the P450 webpage (http://www.p450.unizulu.ac.za/) to identify the named homology protein, in this case CYP128 P450. Hit proteins that showed homology to CYP128 were then selected and different subfamilies were assigned following the International Cytochrome P450 Nomenclature criteria, i.e., P450s showing >55% identity belong to the same subfamily [43,44,45].

### 3.3. Phylogenetic Analysis of CYP128 P450s

Phylogenetic analysis of CYP128 P450s was carried out following the method described elsewhere [31]. First, the protein sequences were aligned by MAFFT v6.864 embedded on the Trex web server [48]. Thereafter, the alignments were automatically subjected to tree inferring and optimization by the Trex web server [49]. Finally, the best-inferred tree was envisioned and colored using iTOL (http://itol.embl.de/about.cgi) [50].

### 3.4. Amino Acid Conservation Analysis

Amino acid conservation analysis of CYP128 P450s was carried out following the methods described elsewhere [22,31,32]. Briefly, 2191 CYP128 P450s with amino acid length ranging from 475–496 amino acids (Supplementary Dataset 1) were selected and subjected to PROMALS3D analysis [33]. In order to calculate the accurate number of conserved amino acids in P450s, it was mentioned that P450s with similar amino acid length should be used [22,25,31,32]. PROMALS3D analysis provided the amino acid conservation index at different protein sequence positions [33], using the numbers from 5 to 9, where 9 is the invariantly conserved amino acid. The conserved number of amino acids for each conservation index was counted and compared with other P450 families from different biological kingdoms [22,25,31,32] to determine the CYP128 P450 family conservation rank.

### 3.5. Generation of EXXR and CXG Sequence Logos

CYP128 P450 family EXXR and CXG sequence logos were constructed using the method described elsewhere [22,32,34]. Briefly, all CYP128 P450 sequences were aligned using ClustalW multiple alignments embedded in MEGA7 [51]. Then the EXXR and CXG region amino acids (4 and 10 amino acids, respectively), were copied and pasted in the WebLogo program (http://weblogo.berkeley.edu/logo.cgi) [52,53]. As a selection parameter, the image format was selected as PNG (bitmap) at 300 dpi resolution. The percentage predominance of amino acids at specific positions was calculated, taking into account the total number of amino acids as 100%. The generated EXXR and CXG logos were used for analysis and comparison to the different P450 family EXXR and CXG logos that have been published and are accessible to the public [22,31,32,34].

### 3.6. Identification of CYP128 P450 Secondary Metabolite BGCs

Secondary metabolite BGCs analysis of CYP128 P450s was carried out following the method described elsewhere [31]. Mycobacterial species BGCs listed at the IMG/M website were manually searched for the presence of CYP128 P450s using their gene ID [42]. The BGCs that contained CYP128 P450 were selected and the entire gene cluster sequence was downloaded. The listed BGCs at IMG/M are unspecific and to identify the particular BGC type, the downloaded gene cluster sequence was subjected to secondary metabolite BGC analysis using anti-SMASH [54]. The type of BGC, percentage similarity to a known cluster and the known cluster name were recorded from the anti-SMASH analysis. Standard BGC abbreviation terminology developed by anti-SMASH was used in the study.

### 3.7. CYP128 Homology Modeling

The Molecular Operating Environment (Chemical Computing Group) was used to build a 3D model of CYP128A1. The crystallographic structure of the P450 Vitamin D3 hydoxylase bound with vitamin D3 (VD3) (PDB ID: 3VRM) [37] was used as a template, showing 33.8% identity and 48% similarity with the targeted sequence after alignment. Homology modeling of CYP128A1 was performed by setting the number of generated models to 10 and by selecting the final model based on MOE’s Generalized Born/Volume Integral (GB/VI) scoring function. During the modeling, the heme group of the template—including the ferric ion—was kept as part of the environment and included in the refinement step. The final model was eventually protonated at neutral pH and minimized using a MOE’s built-in protocol.

### 3.8. Molecular Docking of the CYP128 Model

Non-covalent docking of menaquinone-9 (MK9), a known substrate of CYP128A1, and different azole compounds (Figure S2) was done with MOE’s dock utility. Prior to this, all compounds were “washed” using MOE, i.e., we generated the most dominant protonation state of each compound at neutral pH, computed its atomic partial charges, and minimized the generated 3D structure. Docking was performed into the catalytic site of our CYP128A1 model, setting the placement method to “Triangle Matcher” and the scoring and rescoring methods to “London dG” and “GBVI/WSA dG”, respectively. After docking, the ligand structures were further refined using the fixed receptor option. This refinement step entails energy minimization using the conventional Amber10:Extended Huckel Theory molecular mechanics force field to take electronic effects into account. For each compound, the top 10 complexes as identified from GBVI/WSA-dG scores were considered for further MD equilibration (see Section 3.10). In the case of MK9, since the molecule was predicted to undergo omega hydroxylation via the heme group, only poses with proper orientation (hydrophobic tail facing the heme group) were kept, resulting in six (out of 10) poses being selected.

### 3.9. Density Functional Theory

MD parameters—e.g., partial charges and force constants—for non-standard residues like the heme group are not provided by standard MD force fields. Hence, to run further equilibration of our docked complexes, it was necessary to generate those parameters via quantum calculations. Such calculations were performed using Gaussian 09 (g09) together with the Metal Center Parameter Builder (MCPB.py) available in the Amber16 package [55,56]. MCPB.py was applied to create the correct g09 input files by including the heme ferric cation and its nearby residues from our 3D structures. g09 was successively utilized for geometry optimization, force constant calculation and Merz-Kollman RESP charge calculation of the selected atoms. Every calculation was performed at the B3LYP/6-31G* level of theory. Finally, the MCPB.py program was re-applied to fit RESP charges and generate parameters compatible with Amber’s ff14SB force field. Note that a partial charge of 0.250*e* was calculated for the ferric cation in the heme group. Keeping in mind that the partial charge of a metal ion is less than 2*e*, no matter how big its formal charge (oxidation state) is, our result looks reasonable.

### 3.10. Molecular Dynamics

While MCPB.py and g09 were used to get the correct force field parameters for the ferric core of the heme and nearby atoms, Amber’s antechamber utility [55,56] was applied to generate MD parameters for MK9 and azole compounds. Amber’s tleap program was applied to solvate all our docked complexes in TIP3P water and to generate the correct topology file to conduct MD simulations using the ff14SB force field. For each complex, a rectangular box with at least 10 Å distance between the box edges and the protein was considered, resulting in about 16,380 water molecules in each case. Na^+^ and Cl^−^ ions were added to approximate 0.15 M concentration as well as to neutralize the system. Minimization, NVT, NPT, and MD production runs were all performed with Amber’s pmemd utility. Minimization of each structure was carried out in two phases, both using the steepest descent and conjugate gradient methods successively. Briefly, minimization was done in 10,000 minimizations steps on hydrogens and solvent atoms only, i.e., by restraining the protein–ligand complexes. Next, a 20,000-step minimization was run without restraints. The structures were then equilibrated in the NVT ensemble during 20 ps and in the NPT ensemble during 40 ps, setting the temperature to 298 K and the pressure to 1 bar. Finally, MD production was run to relax each complex for at least 20 ns. The stability of each complex was assessed by checking if the RMSD of both the protein and the ligand reached a plateau by the end of the simulation (see Figure S3). In general, we found 20 ns sufficient to equilibrate the complexes, although in some cases, an extra 5–10 ns may have been required to equilibrate the structure fully.

### 3.11. Redocking of Compounds

MK9 and the azoles compounds were re-docked on their corresponding set of equilibrated structures using the MOE’s dock (six structures for MK9, 10 structures for each of the azole drugs). The same options as in the first docking step were used (see Section 3.8).

## 4. Conclusions

The availability of quite a large number of bacterial genome sequences and different bioinformatics tools gives us an opportunity to understand the role of different genes/proteins in bacterial communities at large rather than confining the results to a single bacterium. In this study, we utilized such information and tools to understand the CYP128 P450 family profiles in different mycobacterial species and understand its structure. The study revealed interesting aspects; for example, P450 was found to be highly conserved in the MTBC species causing lung disease in humans and other animals, indicating the important function of this enzyme during the latent phase of these organisms, as this P450 was found to be expressed during such stage [11,12]. Furthermore, only 12–34% of non-MTBC species that have this P450 strongly support its important role during the latent phase of MTBC species. Contrasting CYP128 subfamily profiles between MTBC and four other different mycobacterial categories revealed by this study suggests that CYP128 P450s may play different roles in different category species, as previously observed for the CYP53 family in fungi [25]. Molecular dynamic simulation of CYP128A1 interactions with different ligands revealed that this P450 has the highest binding affinity to its substrate compared to azole drugs. Binding of azole drugs to CYP128A1 protein models further shows the complex biology of mycobacterial species, as azole drugs have been found to be effective against *Mycobacterium tuberculosis* [18,19,20], indicating the expression of *CYP128A1* in the latent phase [11,12], and possible binding of azole drugs during experimentation did not resulted in a hypervirulent bacterium. This may be due to the inhibition of essential P450s such as CYP121A1 and CYP125A1, leading to the death of *M. tuberculosis* H37Rv. One of the interesting observations of this study is that more than one copy of this gene was present in some species and one of these genes was found not to be part of the gene cluster. This poses a fascinating evolutionary question on the need for having more than one copy of this gene. Research on the CYP128 P450s that are not part of gene clusters would be interesting in discerning other roles of CYP128 P450s in mycobacterial species. It is also important that *CYP128* gene clusters were found to have *CYP124A1* or both *CYP124A1* and *CYP121A1*, indicating the collective efforts of these P450s in generating complex lipids in mycobacterial species, especially in MTBC species, as none of the other four mycobacterial category species has this type of combination in its *CYP128* gene clusters. Future studies should include cloning of the entire *CYP128A1* gene cluster (containing *CYP121A1* and *CYP124A1*) and analyzing the effect of the cluster molecule in *M. tuberculosis* pathogenesis.

## Figures and Tables

**Table 1:** Comparative amino acid conservation analysis of CYP128 P450 family with top 10 ranked families. The conservation index score (5–9) is obtained as described elsewhere [33] using PROMALS3D, where the number 9 indicates invariantly conserved amino acids in P450 members. The CYP128 family is indicated in bold.

P450 Family	Number of Member P450s	Kingdom	PROMALS3D Conservation Index					Rank (Highest to Lowest Conservation)
			5	6	7	8	9	
CYP141	29	Bacteria	0	0	0	0	389	1
CYP51	50	Bacteria	11	102	0	0	264	2
CYP137	38	Bacteria	145	0	0	0	251	3
CYP121	34	Bacteria	0	0	0	0	233	4
**CYP128**	**2191**	**Bacteria**	**118**	**25**	**0**	**0**	**217**	**5 (previously 23rd)**
CYP132	39	Bacteria	175	0	0	0	217	5
CYP5619	23	Stramenopila	118	38	170	0	199	6
CYP124	71	Bacteria	52	35	59	0	170	7
CYP139	894	Bacteria	0	127	0	0	165	8
CYP188	67	Bacteria	62	0	100	0	141	9
CYP123	74	Bacteria	62	0	82	0	137	10

The bold shows the position of CYP128 as it been revised from 23rd place to 5th because of this study results.

**Table 2:** *CYP128* P450 gene cluster analysis in five mycobacterial category species.

Data type	Mycobacterial Category				
	MTBC	MCAC	NTM	MAC	SAP
Total number of *CYP128* P450s	2328	169	140	35	2
Number of *CYP128* P450s not part of BGCs	282	147	120	34	1
Number of *CYP128* P450s part of BGCs	2046	22	20	1	1
Number of BGCs with *CYP128*, *CYP121* and *CYP124* P450s	1908	0	0	0	0
Number of BGCs with *CYP128* P450	136	22	21	1	1
Number of BGCs with *CYP128* and *CYP121* P450s	0	0	0	0	0
Number of BGCs with *CYP128* and *CYP124* P450s	2	0	0	0	0

Abbreviations: MTBC, *Mycobacterium tuberculosis* complex; NTM, non-tuberculosis mycobacteria; MCAC, *Mycobacterium chelonae-abscessus* complex; MAC, *M. avium* complex; and SAP, saprophytes; BGCs, biosynthetic gene clusters.

**Table 3:** Final results obtained after re-docking MK9 and azole drugs on equilibrated CYP128A1 models. The compounds are ranked according to their top docking score, from highest to lowest affinity (column 2). Column 3 shows the score of the first well-orientated pose with respect to the catalytic site. In the case of MK9, proper orientation means that the hydrophobic tail of MK9 is facing the ferric ion of heme, which is consistent with omega hydroxylation of the molecule reported elsewhere [13,16]. For azole drugs, the imidazole ring is facing Fe of heme and the tail should be out of the cavity. In column 3, numbers in parenthesis refer to the rank of the compound based on that score (over all compounds). In column 4, we reported the rank of the first well-oriented pose among all the poses generated for each ligand.

Compound	Top Score (kcal/mol)	Score of First Well-Oriented Pose (kcal/mol)	Rank of First Well-Oriented Pose
Menaquinone-9 (MK9)	−13.48	−12.83 (1)	7
Itraconazole	−12.57	−10.72 (3)	10
Posaconazole	−11.81	−11.81 (2)	1
Ketoconazole	−10.47	−9.84 (4)	9
Econazole	−8.24	−7.56 (7)	6
Miconazole	−8.10	−8.10 (5)	1
Voriconazole	−7.63	−7.57 (6)	2
Clotrimazole	−7.46	−7.21 (9)	5
Fluconazole	−7.38	−7.38 (8)	1

## Supplementary Materials

The following are available online at https://www.mdpi.com/1422-0067/21/14/4816/s1, Figure S1: 3VRM/CYP128A1 alignement., Figure S2: 2D structures of substrate (menaquinone 9) and azole compounds used in the study, Figure S3: RMSD *vs* time during MD simulations of MK9-CYP128A1 complexes (colors are explained in the legend of each figure), Table S1: Comparative amino acid conservation acid analysis of CYP128 P450 family with top 10 ranked families, Supplementary Dataset 1: Comprehensive comparative analyses of CYP128 P450s and those associated with secondary metabolite biosynthetic gene clusters in mycobacterial species, Supplementary Dataset 2: CYP128 P450 sequences identified and annotated in mycobacterial species, Supplementary Dataset 3: A high-resolution phylogenetic tree of CYP128 P450s.
