# Supplementary material for: Comparative Analysis, Structural Insights, and Substrate/Drug Interaction of CYP128A1 in Mycobacterium tuberculosis
Source: Int J Mol Sci. 2020 Jul 8;21(14):4816. doi: 10.3390/ijms21144816 (PMC7404182; doi:10.3390/ijms21144816)
Supplement: Supplementary file 1 [file ijms-21-04816-s001.zip › Supplementary Information/Supplemenatary Dataset 2.docx]

Article

Comparative analysis, structural insights, and substrate/drug interaction of CYP128A1 in *Mycobacterium tuberculosis*

Nokwanda Samantha Ngcobo 1, Zinhle Edith Chiliza 1, Wanping Chen 2, Jae-Hyuk Yu 3,4, David R Nelson 5, Jack A Tuszynski 6,7, Jordane Preto 8*, Khajamohiddin Syed 1,*

1 Department of Biochemistry and Microbiology, Faculty of Science and Agriculture, University of Zululand, KwaDlangezwa 3886, South Africa; [mskwandosamn@gmail.com](mailto:mskwandosamn@gmail.com) (N.S.N.); zinhlechiliza01@gmail.com (Z.C.E); khajamohiddinsyed@gmail.com (K.S.)

2 Department of Molecular Microbiology and Genetics, University of Göttingen, Göttingen 37077, Germany; chenwanping1@foxmail.com (W.C.)

3 Department of Bacteriology, University of Wisconsin-Madison, 3155 MSB, 1550 Linden Drive, Madison, WI 53706, USA; [jyu1@wisc.edu](mailto:jyu1@wisc.edu) (J-H.Y.)

4 Department of Systems Biotechnology, Konkuk University, Seoul 05029, Korea (J-H. Y.)

5 Department of Microbiology, Immunology and Biochemistry, University of Tennessee Health Science Center, Memphis, TN, 38163; [drnelson1@gmail.com](mailto:drnelson1@gmail.com) (D.R.N.)

6 Department of Physics and Department of Oncology, University of Alberta, Edmonton, AB T6G 2E1, Canada; [jack.tuszynski@gmail.com](mailto:jack.tuszynski@gmail.com) (J.A.T.)

7 Department of Mechanical and Aerospace Engineering, Politecnico di Torino, Corso Duca degli Abruzzi, 24, 10129 Torino TO, Italy

8 Université Claude Bernard Lyon 1, INSERM 1052, CNRS 5286, Centre Léon Bérard, Centre de Recherche en Cancérologie de Lyon, France; [jordane.preto@gmail.com](mailto:jordane.preto@gmail.com) (J.P.)

***** Correspondence: jordane.preto@gmail.com (J.P.) and khajamohiddinsyed@gmail.com (K.S.)

Received: date; Accepted: date; Published: date

**Supplementary Dataset 2. CYP128 P450 sequences identified and annotated in mycobacterial species.** Each CYP128 P450 is presented with its gene ID from Integrated Microbial Genomes and Microbiomes in parenthesis followed by species name.

>CYP128A1 (2555325781) Mycobacterium tuberculosis PanR0604

MTATQSPPEPAPDRVRLAGCPLAGTPDVGLTAQDATTALGVPTRRRASSG

GIPVATSMWRDAQTVRTYGPAVAKALALRVAGKARSRLTGRHCRKFMQLT

DFDPFDPAIAADPYPHYRELLAGERVQYNPKRDVYILSRYADVREAARNH

DTLSSARGVTFSRGWLPFLPTSDPPAHTRMRKQLAPGMARGALETWRPMV

DQLARELVGGLLTQTPADVVSTVAAPMPMRAITSVLGVDGPDEAAFCRLS

NQAVRITDVALSASGLISLVQGFAGFRRLRALFTHRRDNGLLRECTVLGK

LATHAEQGRLSDDELFFFAVLLLVAGYESTAHMISTLFLTLADYPDQLTL

LAQQPDLIPSAIEEHLRFISPIQNICRTTRVDYSVGQAVIPAGSLVLLAW

GAANRDPRQYEDPDVFRADRNPVGHLAFGSGIHLCPGTQLARMEGQAILR

EIVANIDRIEVVEPPTWTTNANLRGLTRLRVAVTPRVAP

>CYP128A1 (2566259653) Mycobacterium tuberculosis BT2

MTATQSPPEPAPDRVRLAGCPLAGTPDVGLTAQDATTALGVPTRRRASSG

GIPVATSMWRDAQTVRTYGPAVAKALALRVAGKARSRLTGRHCRKFMQLT

DFDPFDPAIAADPYPHYRELLAGERVQYNPKRDVYILSRYADVREAARNH

DTLSSARGVTFSRGWLPFLPTSDPPAHTRMRKQLAPGMARGALETWRPMV

DQLARELVGGLLTQTPADVVSTVAAPMPMRAITSVLGVDGPDEAAFCRLS

NQAVRITDVALSASGLISLVQGFAGFRRLRALFTHRRDNGLLRECTVLGK

LATHAEQGRLSDDELFFFAVLLLVAGYESTAHMISTLFLTLADYPDQLTL

LAQQPDLIPSAIEEHLRFISPIQNICRTTRVDYSVGQAVIPAGSLVLLAW

GAANRDPRQYEDPDVFRADRNPVGHLAFGSGIHLCPGTQLARMEGQAILR

EIVANIDRIEVVEPPTWTTNANLRGLTRLRVAVTPRVAP

>CYP128A1 (2577804119) Mycobacterium tuberculosis M1283

MTATQSPPEPAPDRVRLAGCPLAGTPDVGLTAQDATTALGVPTRRRASSG

GIPVATSMWRDAQTVRTYGPAVAKALALRVAGKARSRLTGRHCRKFMQLT

DFDPFDPAIAADPYPHYRELLAGERVQYNPKRDVYILSRYADVREAARNH

DTLSSARGVTFSRGWLPFLPTSDPPAHTRMRKQLAPGMARGALETWRPMV

DQLARELVGGLLTQTPADVVSTVAAPMPMRAITSVLGVDGPDEAAFCRLS

NQAVRITDVALSASGLISLVQGFAGFRRLRALFTHRRDNGLLRECTVLGK

LATHAEQGRLSDDELFFFAVLLLVAGYESTAHMISTLFLTLADYPDQLTL

LAQQPDLIPSAIEEHLRFISPIQNICRTTRVDYSVGQAVIPAGSLVLLAW

GAANRDPRQYEDPDVFRADRNPVGHLAFGSGIHLCPGTQLARMEGQAILR

EIVANIDRIEVVEPPTWTTNANLRGLTRLRVAVTPRVAP

>CYP128A1 (2592402469) Mycobacterium tuberculosis TKK_02_0022

MTATQSPPEPAPDRVRLAGCPLAGTPDVGLTAQDATTALGVPTRRRASSG

GIPVATSMWRDAQTVRTYGPAVAKALALRVAGKARSRLTGRHCRKFMQLT

DFDPFDPAIAADPYPHYRELLAGERVQYNPKRDVYILSRYADVREAARNH

DTLSSARGVTFSRGWLPFLPTSDPPAHTRMRKQLAPGMARGALETWRPMV

DQLARELVGGLLTQTPADVVSTVAAPMPMRAITSVLGVDGPDEAAFCRLS

NQAVRITDVALSASGLISLVQGFAGFRRLRALFTHRRDNGLLRECTVLGK

LATHAEQGRLSDDELFFFAVLLLVAGYESTAHMISTLFLTLADYPDQLTL

LAQQPDLIPSAIEEHLRFISPIQNICRTTRVDYSVGQAVIPAGSLVLLAW

GAANRDPRQYEDPDVFRADRNPVGHLAFGSGIHLCPGTQLARMEGQAILR

EIVANIDRIEVVEPPTWTTNANLRGLTRLRVAVTPRVAP

>CYP128A1 (2604697374) Mycobacterium tuberculosis TB_RSA56

MTATQSPPEPAPDRVRLAGCPLAGTPDVGLTAQDATTALGVPTRRRASSG

GIPVATSMWRDAQTVRTYGPAVAKALALRVAGKARSRLTGRHCRKFMQLT

DFDPFDPAIAADPYPHYRELLAGERVQYNPKRDVYILSRYADVREAARNH

DTLSSARGVTFSRGWLPFLPTSDPPAHTRMRKQLAPGMARGALETWRPMV

DQLARELVGGLLTQTPADVVSTVAAPMPMRAITSVLGVDGPDEAAFCRLS

NQAVRITDVALSASGLISLVQGFAGFRRLRALFTHRRDNGLLRECTVLGK

LATHAEQGRLSDDELFFFAVLLLVAGYESTAHMISTLFLTLADYPDQLTL

LAQQPDLIPSAIEEHLRFISPIQNICRTTRVDYSVGQAVIPAGSLVLLAW

GAANRDPRQYEDPDVFRADRNPVGHLAFGSGIHLCPGTQLARMEGQAILR

EIVANIDRIEVVEPPTWTTNANLRGLTRLRVAVTPRVAP

>CYP128A1 (2622428465) Mycobacterium tuberculosis M1292

MTATQSPPEPAPDRVRLAGCPLAGTPDVGLTAQDATTALGVPTRRRASSG

GIPVATSMWRDAQTVRTYGPAVAKALALRVAGKARSRLTGRHCRKFMQLT

DFDPFDPAIAADPYPHYRELLAGERVQYNPKRDVYILSRYADVREAARNH

DTLSSARGVTFSRGWLPFLPTSDPPAHTRMRKQLAPGMARGALETWRPMV

DQLARELVGGLLTQTPADVVSTVAAPMPMRAITSVLGVDGPDEAAFCRLS

NQAVRITDVALSASGLISLVQGFAGFRRLRALFTHRRDNGLLRECTVLGK

LATHAEQGRLSDDELFFFAVLLLVAGYESTAHMISTLFLTLADYPDQLTL

LAQQPDLIPSAIEEHLRFISPIQNICRTTRVDYSVGQAVIPAGSLVLLAW

GAANRDPRQYEDPDVFRADRNPVGHLAFGSGIHLCPGTQLARMEGQAILR

EIVANIDRIEVVEPPTWTTNANLRGLTRLRVAVTPRVAP

>CYP128A1 (2659515410) Mycobacterium tuberculosis 400711

MTATQSPPEPAPDRVRLAGCPLAGTPDVGLTAQDATTALGVPTRRRASSG

GIPVATSMWRDAQTVRTYGPAVAKALALRVAGKARSRLTGRHCRKFMQLT

DFDPFDPAIAADPYPHYRELLAGERVQYNPKRDVYILSRYADVREAARNH

DTLSSARGVTFSRGWLPFLPTSDPPAHTRMRKQLAPGMARGALETWRPMV

DQLARELVGGLLTQTPADVVSTVAAPMPMRAITSVLGVDGPDEAAFCRLS

NQAVRITDVALSASGLISLVQGFAGFRRLRALFTHRRDNGLLRECTVLGK

LATHAEQGRLSDDELFFFAVLLLVAGYESTAHMISTLFLTLADYPDQLTL

LAQQPDLIPSAIEEHLRFISPIQNICRTTRVDYSVGQAVIPAGSLVLLAW

GAANRDPRQYEDPDVFRADRNPVGHLAFGSGIHLCPGTQLARMEGQAILR

EIVANIDRIEVVEPPTWTTNANLRGLTRLRVAVTPRVAP

>CYP128A1 (2703663993) Mycobacterium tuberculosis K09901972

MTATQSPPEPAPDRVRLAGCPLAGTPDVGLTAQDATTALGVPTRRRASSG

GIPVATSMWRDAQTVRTYGPAVAKALALRVAGKARSRLTGRHCRKFMQLT

DFDPFDPAIAADPYPHYRELLAGERVQYNPKRDVYILSRYADVREAARNH

DTLSSARGVTFSRGWLPFLPTSDPPAHTRMRKQLAPGMARGALETWRPMV

DQLARELVGGLLTQTPADVVSTVAAPMPMRAITSVLGVDGPDEAAFCRLS

NQAVRITDVALSASGLISLVQGFAGFRRLRALFTHRRDNGLLRECTVLGK

LATHAEQGRLSDDELFFFAVLLLVAGYESTAHMISTLFLTLADYPDQLTL

LAQQPDLIPSAIEEHLRFISPIQNICRTTRVDYSVGQAVIPAGSLVLLAW

GAANRDPRQYEDPDVFRADRNPVGHLAFGSGIHLCPGTQLARMEGQAILR

EIVANIDRIEVVEPPTWTTNANLRGLTRLRVAVTPRVAP

>CYP128A1 (2621405764) Mycobacterium tuberculosis XTB13-112

MTATQSPPEPAPDRVRLAGCPLAGTPDVGLTAQDATTALGVPTRRRAASG

GIPVATSMWRDAQTVRTYGPAVAKALALRVAGKARSRLTGRHCRKFMQLT

DFDPFDPAIAADPYPHYRELLAGERVQYNPKRDVYILSRYADVREAARNH

DTLSSARGVTFSRGWLPFLPTSDPPAHTRMRKQLAPGMARGALETWRPMV

DQLARELVGGLLTQTPADVVSTVAAPMPMRAITSVLGVDGPDEAAFCRLS

NQAVRITDVALSASGLISLVQGFAGFRRLRALFTHRRDNGLLRECTVLGK

LATHAEQGRLSDDELFFFAVLLLVAGYESTAHMISTLFLTLADYPDQLTL

LAQQPDLIPSAIEEHLRFISPIQNICRTTRVDYSVGQAVIPAGSLVLLAW

GAANRDPRQYEDPDVFRADRNPVGHLAFGSGIHLCPGTQLARMEGQAILR

EIVANIDRIEVVEPPTWTTNANLRGLTRLRVAVTPRVAP

>CYP128A1 (2581356877) Mycobacterium bovis Wt 21419

MTATQSPPEPAPDRVRLAGCPLAGTPDVGLTAQDATTALGVPTRRRASSG

GIPVATSMWRDAQTVRTYGPAVAKALALRVAGKARSRLTGRHCRKFMQLT

DFDPFDPAIAADPYPHYRELLAGERVQDNPKRDVYILSRYADVREAARNH

DTLSSARGVTFSRGWLPFLPTSDPPAHTRMRKQLAPGMARGALETWRPMV

DQLARELVGGLLTQTPADVVSTVAAPMPMRAITSVLGVDGPDEAAFCRLS

NQAVRITDVALSASGLISLVQGFAGFRRLRALFTHRRDNGLLRECTVLGK

LATHAEQGRLSDDELFFFAVLLLVAGYESTAHMISTLFLTLADYPDQLTL

LAQQPDLIPSAIEEHLRFISPIQNICRTTRVDYSVGQAVIPAGSLVLLAW

GAANRDPRQYEDPDVFRADRNPVGHLAFGSGIHLCPGTQLARMEGQAILR

EIVANIDRIEVVEPPTWTTNANLRGLTRLRVAVTPRVAP

>CYP128B4 (2662865069) Mycobacterium abscessus PAP008

MSRWRSDSAVRQIGAPVVAAVGMNIAAAVRVRRRGYAGWTGAVNTDYDPL

DPATAAQPFDAYRALHAGGRVHYNPKRATFILSRHEDIRAALRDTDAVTS

SQGVTRMKISAPILVLTDGDDHTRLRKQVQPGFTRGAMSDWQGMADQLAK

ELVADVVANPGCDVMERLAVPLPIRMIAHIIGIPPEDVQNFRSWSEDGVG

VINAGVSPAGLRQGLKGVRAIAALRRYFKDQLASGKLKGSDTVLGRLVDN

NEDGKLSDDELFFIAMLLLFAGNETTTNLIGGMFDTLAHAPDQFAMIRDD

PDLIPSAVEEQLRYSAPIQNLYRYTRTDYRVGEVTIPSGSRLLLAFGAAN

RDPEVFEDPDTYRADRNPRNHIAFGYGVHMCIGATLSRMEGQAVLRELTS

QASAIAAAGSATWSTNSSLRGTTYLPIRLTPAR

>CYP128B4 (2797827088) Mycobacteroides abscessus abscessus 402

MSRWRSDSAVRQIGAPVVAAVGMNIAAAVRVRRRGYAGWTGAVNTDYDPL

DPATAAQPFDAYRALHAGGRVHYNPKRATFILSRHEDIRAALRDTDAVTS

SQGVTRMKISAPILVLTDGDDHTRLRKQVQPGFTRGAMSDWQGMADQLAK

ELVADVVANPGCDVMERLAVPLPIRMIAHIIGIPPEDVQNFRSWSEDGVG

VINAGVSPAGLRQGLKGVRAIAALRRYFKDQLASGKLKGSDTVLGRLVDN

NEDGKLSDDELFFIAMLLLFAGNETTTNLIGGMFDTLAHAPDQFAMIRDD

PDLIPSAVEEQLRYSAPIQNLYRYTRTDYRVGEVTIPSGSRLLLAFGAAN

RDPEVFEDPDTYRADRNPRNHIAFGYGVHMCIGATLSRMEGQAVLRELTS

QASAIAAAGSATWSTNSSLRGTTYLPIRLTPAR

>CYP128A1 (2630330262) Mycobacterium chelonae CM 6388

MTLKHDARLVYTTTKPVVRSLITNAATEMRAKVSGRRFPGVQETVFDPMD

PETAANPYPGYRMLLAGGRVHYNRKRNAFILCRYEDVRAAARNDALLSNR

DGVVRARFEVPVLLNMDRPRHTELRRKALPGFTRGALEGWAPTVDRMAAE

LVTGLLSDPGADVVEHLAVPLPMRMIAHILGIPPEDEAFFRHWSNESVRV

ANVEFSPKGLRQVPGTLNGVRHLHDYFMTQLGKGNLLGSDTVLGKLVADA

GEGQISHDELFYFALLLLLAGNETTTNLLSTMFLTMSENPDQFEVIRSDP

RLLAGAVEEQLRYSSPIQNFYRTAAQDYPVEDAVIPAGARVALLWGAANR

DPREFDDPDRFLATRPVAQHVAFGSGVHLCLGAGLARMEGQAVLRELINR

VRRVEIEGTPRWTTNSSLRGLEELRVRLVAR

>CYP128A1 (2584983681) Mycobacterium tuberculosis M1415

MTATQSPPEPAPDRVRLAGCPLAGTPDVGLTAQDATTALGVPTRRRASSG

GIPVATSMWRDAQTVRTYGPAVAKALALRVAGKARSRLTGRHCRKFMQLT

DFDPFDPAIAADPYPHYRELLAGERVQYNPKRDVYILSRYADVREAARNH

DTLSSARGVTFSRGWLPFLPTSDPPAHTRMRKQLAPGMARGALETWRPMV

DQLARELVGGLLTQTPADVVSTVAAPMPMRAITSVLGVDGPDEAAFCRLS

NQAVRITDVALSASGLISLVQGFAGFRRLRALFTHRRDNGLLRECTVLGK

LATHAEQGRLSDDELFFFAVLLLVAGYESTAHMISTLFLTLADYPDQLTL

LAQQPDLIPSAIEEHLRFISPIQNICRTTRVDYSVGQAVIPAGSLVLLAW

GAANRDPRQYEDPDVFRADRNPVGHLAFGSGIHLCPGTQLARMEGQAILR

EIVANIDRIEVVEPPTWTTNANLRGLTRLRVAVTPRVAP

>CYP128A1 (2589032170) Mycobacterium tuberculosis TBR5

MTATQSPPEPAPDRVRLAGCPLAGTPDVGLTAQDATTALGVPTRRRASSG

GIPVATSMWRDAQTVRTYGPAVAKALALRVAGKARSRLTGRHCRKFMQLT

DFDPFDPAIAADPYPHYRELLAGERVQYNPKRDVYILSRYADVREAARNH

DTLSSARGVTFSRGWLPFLPTSDPPAHTRMRKQLAPGMARGALETWRPMV

DQLARELVGGLLTQTPADVVSTVAAPMPMRAITSVLGVDGPDEAAFCRLS

NQAVRITDVALSASGLISLVQGFAGFRRLRALFTHRRDNGLLRECTVLGK

LATHAEQGRLSDDELFFFAVLLLVAGYESTAHMISTLFLTLADYPDQLTL

LAQQPDLIPSAIEEHLRFISPIQNICRTTRVDYSVGQAVIPAGSLVLLAW

GAANRDPRQYEDPDVFRADRNPVGHLAFGSGIHLCPGTQLARMEGQAILR

EIVANIDRIEVVEPPTWTTNANLRGLTRLRVAVTPRVAP

>CYP128A1 (2592422877) Mycobacterium tuberculosis TKK_02_0016

MTATQSPPEPAPDRVRLAGCPLAGTPDVGLTAQDATTALGVPTRRRASSG

GIPVATSMWRDAQTVRTYGPAVAKALALRVAGKARSRLTGRHCRKFMQLT

DFDPFDPAIAADPYPHYRELLAGERVQYNPKRDVYILSRYADVREAARNH

DTLSSARGVTFSRGWLPFLPTSDPPAHTRMRKQLAPGMARGALETWRPMV

DQLARELVGGLLTQTPADVVSTVAAPMPMRAITSVLGVDGPDEAAFCRLS

NQAVRITDVALSASGLISLVQGFAGFRRLRALFTHRRDNGLLRECTVLGK

LATHAEQGRLSDDELFFFAVLLLVAGYESTAHMISTLFLTLADYPDQLTL

LAQQPDLIPSAIEEHLRFISPIQNICRTTRVDYSVGQAVIPAGSLVLLAW

GAANRDPRQYEDPDVFRADRNPVGHLAFGSGIHLCPGTQLARMEGQAILR

EIVANIDRIEVVEPPTWTTNANLRGLTRLRVAVTPRVAP

>CYP128A1 (2622586052) Mycobacterium tuberculosis M1461

MTATQSPPEPAPDRVRLAGCPLAGTPDVGLTAQDATTALGVPTRRRASSG

GIPVATSMWRDAQTVRTYGPAVAKALALRVAGKARSRLTGRHCRKFMQLT

DFDPFDPAIAADPYPHYRELLAGERVQYNPKRDVYILSRYADVREAARNH

DTLSSARGVTFSRGWLPFLPTSDPPAHTRMRKQLAPGMARGALETWRPMV

DQLARELVGGLLTQTPADVVSTVAAPMPMRAITSVLGVDGPDEAAFCRLS

NQAVRITDVALSASGLISLVQGFAGFRRLRALFTHRRDNGLLRECTVLGK

LATHAEQGRLSDDELFFFAVLLLVAGYESTAHMISTLFLTLADYPDQLTL

LAQQPDLIPSAIEEHLRFISPIQNICRTTRVDYSVGQAVIPAGSLVLLAW

GAANRDPRQYEDPDVFRADRNPVGHLAFGSGIHLCPGTQLARMEGQAILR

EIVANIDRIEVVEPPTWTTNANLRGLTRLRVAVTPRVAP

>CYP128A1 (2633813046) Mycobacterium tuberculosis KT-0191

MTATQSPPEPAPDRVRLAGCPLAGTPDVGLTAQDATTALGVPTRRRASSG

GIPVATSMWRDAQTVRTYGPAVAKALALRVAGKARSRLTGRHCRKFMQLT

DFDPFDPAIAADPYPHYRELLAGERVQYNPKRDVYILSRYADVREAARNH

DTLSSARGVTFSRGWLPFLPTSDPPAHTRMRKQLAPGMARGALETWRPMV

DQLARELVGGLLTQTPADVVSTVAAPMPMRAITSVLGVDGPDEAAFCRLS

NQAVRITDVALSASGLISLVQGFAGFRRLRALFTHRRDNGLLRECTVLGK

LATHAEQGRLSDDELFFFAVLLLVAGYESTAHMISTLFLTLADYPDQLTL

LAQQPDLIPSAIEEHLRFISPIQNICRTTRVDYSVGQAVIPAGSLVLLAW

GAANRDPRQYEDPDVFRADRNPVGHLAFGSGIHLCPGTQLARMEGQAILR

EIVANIDRIEVVEPPTWTTNANLRGLTRLRVAVTPRVAP

>CYP128A1 (2659963381) Mycobacterium tuberculosis 300239

MTATQSPPEPAPDRVRLAGCPLAGTPDVGLTAQDATTALGVPTRRRASSG

GIPVATSMWRDAQTVRTYGPAVAKALALRVAGKARSRLTGRHCRKFMQLT

DFDPFDPAIAADPYPHYRELLAGERVQYNPKRDVYILSRYADVREAARNH

DTLSSARGVTFSRGWLPFLPTSDPPAHTRMRKQLAPGMARGALETWRPMV

DQLARELVGGLLTQTPADVVSTVAAPMPMRAITSVLGVDGPDEAAFCRLS

NQAVRITDVALSASGLISLVQGFAGFRRLRALFTHRRDNGLLRECTVLGK

LATHAEQGRLSDDELFFFAVLLLVAGYESTAHMISTLFLTLADYPDQLTL

LAQQPDLIPSAIEEHLRFISPIQNICRTTRVDYSVGQAVIPAGSLVLLAW

GAANRDPRQYEDPDVFRADRNPVGHLAFGSGIHLCPGTQLARMEGQAILR

EIVANIDRIEVVEPPTWTTNANLRGLTRLRVAVTPRVAP

>CYP128A1 (2675743370) Mycobacterium tuberculosis R09401256

MTATQSPPEPAPDRVRLAGCPLAGTPDVGLTAQDATTALGVPTRRRASSG

GIPVATSMWRDAQTVRTYGPAVAKALALRVAGKARSRLTGRHCRKFMQLT

DFDPFDPAIAADPYPHYRELLAGERVQYNPKRDVYILSRYADVREAARNH

DTLSSARGVTFSRGWLPFLPTSDPPAHTRMRKQLAPGMARGALETWRPMV

DQLARELVGGLLTQTPADVVSTVAAPMPMRAITSVLGVDGPDEAAFCRLS

NQAVRITDVALSASGLISLVQGFAGFRRLRALFTHRRDNGLLRECTVLGK

LATHAEQGRLSDDELFFFAVLLLVAGYESTAHMISTLFLTLADYPDQLTL

LAQQPDLIPSAIEEHLRFISPIQNICRTTRVDYSVGQAVIPAGSLVLLAW

GAANRDPRQYEDPDVFRADRNPVGHLAFGSGIHLCPGTQLARMEGQAILR

EIVANIDRIEVVEPPTWTTNANLRGLTRLRVAVTPRVAP

>CYP128A1 (2697436822) Mycobacterium tuberculosis SH5-875

MTATQSPPEPAPDRVRLAGCPLAGTPDVGLTAQDATTALGVPTRRRASSG

GIPVATSMWRDAQTVRTYGPAVAKALALRVAGKARSRLTGRHCRKFMQLT

DFDPFDPAIAADPYPHYRELLAGERVQYNPKRDVYILSRYADVREAARNH

DTLSSARGVTFSRGWLPFLPTSDPPAHTRMRKQLAPGMARGALETWRPMV

DQLARELVGGLLTQTPADVVSTVAAPMPMRAITSVLGVDGPDEAAFCRLS

NQAVRITDVALSASGLISLVQGFAGFRRLRALFTHRRDNGLLRECTVLGK

LATHAEQGRLSDDELFFFAVLLLVAGYESTAHMISTLFLTLADYPDQLTL

LAQQPDLIPSAIEEHLRFISPIQNICRTTRVDYSVGQAVIPAGSLVLLAW

GAANRDPRQYEDPDVFRADRNPVGHLAFGSGIHLCPGTQLARMEGQAILR

EIVANIDRIEVVEPPTWTTNANLRGLTRLRVAVTPRVAP

>CYP128A1 (648336636) Mycobacterium tuberculosis KZN R506 NZ_ACVU02000049

MTATQSPPEPAPDRVRLAGCPLAGTPDVGLTAQDATTALGVPTRRRASSG

GIPVATSMWRDAQTVRTYGPAVAKALALRVAGKARSRLTGRHCRKFMQLT

DFDPFDPAIAADPYPHYRELLAGERVQYNPKRDVYILSRYADVREAARNH

DTLSSARGVTFSRGWLPFLPTSDPPAHTRMRKQLAPGMARGALETWRPMV

DQLARELVGGLLTQTPADVVSTVAAPMPMRAITSVLGVDGPDEAAFCRLS

NQAVRITDVALSASGLISLVQGFAGFRRLRALFTHRRDNGLLRECTVLGK

LATHAEQGRLSDDELFFFAVLLLVAGYESTAHMISTLFLTLADYPDQLTL

LAQQPDLIPSAIEEHLRFISPIQNICRTTRVDYSVGQAVIPAGSLVLLAW

GAANRDPRQYEDPDVFRADRNPVGHLAFGSGIHLCPGTQLARMEGQAILR

EIVANIDRIEVVEPPTWTTNANLRGLTRLRVAVTPRVAP

>CYP128A1 (643735104) Mycobacterium bovis BCG str. Tokyo 172

MTATQSPPEPAPDRVRLAGCPLAGTPDVGLTAQDATTALGVPTRRRASSG

GIPVATSMWRDAQTVRTYGPAVAKALALRVAGKARSRLTGRHCRKFMQLT

DFDPFDPAIAADPYPHYRELLAGERVQYNPKRDVYILSRYADVREAARNH

DTLSSARGVTFSRGWLPFLPTSDPPAHTRMRKQLAPGMARGALETWRPMV

DQFARELVGGLLTQTPADVVSTVAAPMPMRAITSVLGVDGPDEAAFCRLS

NQAVRITDVALSASGLISLVQGFAGFRRLRALFTHRRDNGLLRECTVLGK

LATHAEQGRLSDDELFFFAVLLLVAGYESTAHMISTLFLTLADYPDQLTL

LAQQPDLIPSAIEEHLRFISPIQNICRTTRVDYSVGQAVIPAGSLVLLAW

GAANRDPRQYEDPDVFRADRNPVGHLAFGSGIHLCPGTQLARMEGQAILR

EIVANIDRIEVVEPPTWTTNANLRGLTRLRVAVTPRVAP

>CYP128A1 (2703633199) Mycobacterium tuberculosis I09702330

MTATQSPPEPAPDRVRLAGCPLAGTPDVGLTAQDATTALGVPTRRRASSG

GIPVATSMWRDAQTVRTYGPAVAKALALRVAGKARSRLTGRHCRKFMQLT

DFDPFDPAIAADPYPHYRELLAGERVQYNPKRDVYILSRYADVREAARNH

DTLSSARGVTFSRGWLPFLPTSDPPAHTRMRKQLAPGMARGALETWRPMV

DQLARELVGGLLTQTPADVVSTVAAPMPMRAITSVLGVDGPDEAAFCRLS

NQAVRITDVALSASGLISLVQGFAGFRRLRALFTHRRDNGLLRECTVLGK

LATHAEQGRLSDDELFFFAVLLLVAGYESTAHMISTLFLTLADYPDQLTL

LAQQPDLIPSAIEEHLRFISPIQNICRTTRVDYSVGQAVIPAGSLVLLAW

GAANRDPRQYEDPDVFRADRNPVGHLAFGSGIHLCPGPSWRAWRVRRSCA

RSSPISTE

>CYP128B3 (2518812981) Mycobacterium sp. MOTT36Y

MSAPLAVLTDGEEHTRLRKQIQPGFSKGAMSAWQEIVEKLAVELVSDLLN

NPGCDVVQHLAIPMPIRLIAQILGVPEDDVDDFRRWSENAVRVMDLTPTR

AGLIGATRSVSAMVALQRYFVKQFAHGGLKGSGTVLGRLLEHNTDGSLTD

RQLLLIATHLLIAGNETTTNLLGGMFDTLARHPNQYDLIRANPDLIPTAV

EEQLRITTPIQNLYRYTRADYQIGDVTIPAGSRVLLSFGAANRDPAVFDE

PDEYRADRNPRAHVAFGYGPHMCLGAPLARMEAQAVLRQLVTRVSRLSPN

GPTTWSAHSSLRGPTYLPIHLTPA

>CYP128A1 (2575957277) Mycobacterium tuberculosis TB_RSA06

MTATQSPPEPAPDRVRLAGCPLAGTPDVGLTAQDATTALGVPTRRRASSG

GIPVATSMWRDAQTVRTYGPAVAKALALRVAGKARSRLTGRHCRKFMQLT

DFDPFDPAIAADPYPHYRELLAGERVQYNPKRDVYILSRYADVREAARNH

DTLSSARGVTFSRGWLPFLPTSDPPAHTRMRKQLAPGMARGALETWRPMV

DQLARELVGGLLTQTPADVVSTVAAPMPMRAITSVLGVDGPDEAAFCRLS

NQAVRITDVALSASGLISLVQGFAGFRRLRALFTHRRDNGLLRECTVLGK

LATHAEQGRLSDDELFFFAVLLLVAGYESTAHMISTLFLTLADYPDQLTL

LAQQPDLIPSAIEEHLRFISPIQNICRTTRVDYSVGQAVIPAGSLVLLAW

GAANRDPRQYEDPDVFRADRNPVGHLAFGSGIHLCPGTQLARMEGQAILR

EIVANIDRIEVVEPPTWTTNANLRGLTRLRVAVTPRVAP

>CYP128A1 (2576509773) Mycobacterium tuberculosis TRUG0107

MTATQSPPEPAPDRVRLAGCPLAGTPDVGLTAQDATTALGVPTRRRASSG

GIPVATSMWRDAQTVRTYGPAVAKALALRVAGKARSRLTGRHCRKFMQLT

DFDPFDPAIAADPYPHYRELLAGERVQYNPKRDVYILSRYADVREAARNH

DTLSSARGVTFSRGWLPFLPTSDPPAHTRMRKQLAPGMARGALETWRPMV

DQLARELVGGLLTQTPADVVSTVAAPMPMRAITSVLGVDGPDEAAFCRLS

NQAVRITDVALSASGLISLVQGFAGFRRLRALFTHRRDNGLLRECTVLGK

LATHAEQGRLSDDELFFFAVLLLVAGYESTAHMISTLFLTLADYPDQLTL

LAQQPDLIPSAIEEHLRFISPIQNICRTTRVDYSVGQAVIPAGSLVLLAW

GAANRDPRQYEDPDVFRADRNPVGHLAFGSGIHLCPGTQLARMEGQAILR

EIVANIDRIEVVEPPTWTTNANLRGLTRLRVAVTPRVAP

>CYP128A1 (2589110070) Mycobacterium tuberculosis TBR59

MTATQSPPEPAPDRVRLAGCPLAGTPDVGLTAQDATTALGVPTRRRASSG

GIPVATSMWRDAQTVRTYGPAVAKALALRVAGKARSRLTGRHCRKFMQLT

DFDPFDPAIAADPYPHYRELLAGERVQYNPKRDVYILSRYADVREAARNH

DTLSSARGVTFSRGWLPFLPTSDPPAHTRMRKQLAPGMARGALETWRPMV

DQLARELVGGLLTQTPADVVSTVAAPMPMRAITSVLGVDGPDEAAFCRLS

NQAVRITDVALSASGLISLVQGFAGFRRLRALFTHRRDNGLLRECTVLGK

LATHAEQGRLSDDELFFFAVLLLVAGYESTAHMISTLFLTLADYPDQLTL

LAQQPDLIPSAIEEHLRFISPIQNICRTTRVDYSVGQAVIPAGSLVLLAW

GAANRDPRQYEDPDVFRADRNPVGHLAFGSGIHLCPGTQLARMEGQAILR

EIVANIDRIEVVEPPTWTTNANLRGLTRLRVAVTPRVAP

>CYP128A1 (2590020806) Mycobacterium tuberculosis MAL010078

MTATQSPPEPAPDRVRLAGCPLAGTPDVGLTAQDATTALGVPTRRRASSG

GIPVATSMWRDAQTVRTYGPAVAKALALRVAGKARSRLTGRHCRKFMQLT

DFDPFDPAIAADPYPHYRELLAGERVQYNPKRDVYILSRYADVREAARNH

DTLSSARGVTFSRGWLPFLPTSDPPAHTRMRKQLAPGMARGALETWRPMV

DQLARELVGGLLTQTPADVVSTVAAPMPMRAITSVLGVDGPDEAAFCRLS

NQAVRITDVALSASGLISLVQGFAGFRRLRALFTHRRDNGLLRECTVLGK

LATHAEQGRLSDDELFFFAVLLLVAGYESTAHMISTLFLTLADYPDQLTL

LAQQPDLIPSAIEEHLRFISPIQNICRTTRVDYSVGQAVIPAGSLVLLAW

GAANRDPRQYEDPDVFRADRNPVGHLAFGSGIHLCPGTQLARMEGQAILR

EIVANIDRIEVVEPPTWTTNANLRGLTRLRVAVTPRVAP

>CYP128A1 (2590130752) Mycobacterium tuberculosis MAL020182

MTATQSPPEPAPDRVRLAGCPLAGTPDVGLTAQDATTALGVPTRRRASSG

GIPVATSMWRDAQTVRTYGPAVAKALALRVAGKARSRLTGRHCRKFMQLT

DFDPFDPAIAADPYPHYRELLAGERVQYNPKRDVYILSRYADVREAARNH

DTLSSARGVTFSRGWLPFLPTSDPPAHTRMRKQLAPGMARGALETWRPMV

DQLARELVGGLLTQTPADVVSTVAAPMPMRAITSVLGVDGPDEAAFCRLS

NQAVRITDVALSASGLISLVQGFAGFRRLRALFTHRRDNGLLRECTVLGK

LATHAEQGRLSDDELFFFAVLLLVAGYESTAHMISTLFLTLADYPDQLTL

LAQQPDLIPSAIEEHLRFISPIQNICRTTRVDYSVGQAVIPAGSLVLLAW

GAANRDPRQYEDPDVFRADRNPVGHLAFGSGIHLCPGTQLARMEGQAILR

EIVANIDRIEVVEPPTWTTNANLRGLTRLRVAVTPRVAP

>CYP128A1 (2603406094) Mycobacterium tuberculosis TB_RSA153

MTATQSPPEPAPDRVRLAGCPLAGTPDVGLTAQDATTALGVPTRRRASSG

GIPVATSMWRDAQTVRTYGPAVAKALALRVAGKARSRLTGRHCRKFMQLT

DFDPFDPAIAADPYPHYRELLAGERVQYNPKRDVYILSRYADVREAARNH

DTLSSARGVTFSRGWLPFLPTSDPPAHTRMRKQLAPGMARGALETWRPMV

DQLARELVGGLLTQTPADVVSTVAAPMPMRAITSVLGVDGPDEAAFCRLS

NQAVRITDVALSASGLISLVQGFAGFRRLRALFTHRRDNGLLRECTVLGK

LATHAEQGRLSDDELFFFAVLLLVAGYESTAHMISTLFLTLADYPDQLTL

LAQQPDLIPSAIEEHLRFISPIQNICRTTRVDYSVGQAVIPAGSLVLLAW

GAANRDPRQYEDPDVFRADRNPVGHLAFGSGIHLCPGTQLARMEGQAILR

EIVANIDRIEVVEPPTWTTNANLRGLTRLRVAVTPRVAP

>CYP128A1 (2621667262) Mycobacterium tuberculosis XTB13-265

MTATQSPPEPAPDRVRLAGCPLAGTPDVGLTAQDATTALGVPTRRRASSG

GIPVATSMWRDAQTVRTYGPAVAKALALRVAGKARSRLTGRHCRKFMQLT

DFDPFDPAIAADPYPHYRELLAGERVQYNPKRDVYILSRYADVREAARNH

DTLSSARGVTFSRGWLPFLPTSDPPAHTRMRKQLAPGMARGALETWRPMV

DQLARELVGGLLTQTPADVVSTVAAPMPMRAITSVLGVDGPDEAAFCRLS

NQAVRITDVALSASGLISLVQGFAGFRRLRALFTHRRDNGLLRECTVLGK

LATHAEQGRLSDDELFFFAVLLLVAGYESTAHMISTLFLTLADYPDQLTL

LAQQPDLIPSAIEEHLRFISPIQNICRTTRVDYSVGQAVIPAGSLVLLAW

GAANRDPRQYEDPDVFRADRNPVGHLAFGSGIHLCPGTQLARMEGQAILR

EIVANIDRIEVVEPPTWTTNANLRGLTRLRVAVTPRVAP

>CYP128A1 (2621823827) Mycobacterium tuberculosis TKK_05MA_0016

MTATQSPPEPAPDRVRLAGCPLAGTPDVGLTAQDATTALGVPTRRRASSG

GIPVATSMWRDAQTVRTYGPAVAKALALRVAGKARSRLTGRHCRKFMQLT

DFDPFDPAIAADPYPHYRELLAGERVQYNPKRDVYILSRYADVREAARNH

DTLSSARGVTFSRGWLPFLPTSDPPAHTRMRKQLAPGMARGALETWRPMV

DQLARELVGGLLTQTPADVVSTVAAPMPMRAITSVLGVDGPDEAAFCRLS

NQAVRITDVALSASGLISLVQGFAGFRRLRALFTHRRDNGLLRECTVLGK

LATHAEQGRLSDDELFFFAVLLLVAGYESTAHMISTLFLTLADYPDQLTL

LAQQPDLIPSAIEEHLRFISPIQNICRTTRVDYSVGQAVIPAGSLVLLAW

GAANRDPRQYEDPDVFRADRNPVGHLAFGSGIHLCPGTQLARMEGQAILR

EIVANIDRIEVVEPPTWTTNANLRGLTRLRVAVTPRVAP

>CYP128A1 (2622072878) Mycobacterium tuberculosis MD18112

MTATQSPPEPAPDRVRLAGCPLAGTPDVGLTAQDATTALGVPTRRRASSG

GIPVATSMWRDAQTVRTYGPAVAKALALRVAGKARSRLTGRHCRKFMQLT

DFDPFDPAIAADPYPHYRELLAGERVQYNPKRDVYILSRYADVREAARNH

DTLSSARGVTFSRGWLPFLPTSDPPAHTRMRKQLAPGMARGALETWRPMV

DQLARELVGGLLTQTPADVVSTVAAPMPMRAITSVLGVDGPDEAAFCRLS

NQAVRITDVALSASGLISLVQGFAGFRRLRALFTHRRDNGLLRECTVLGK

LATHAEQGRLSDDELFFFAVLLLVAGYESTAHMISTLFLTLADYPDQLTL

LAQQPDLIPSAIEEHLRFISPIQNICRTTRVDYSVGQAVIPAGSLVLLAW

GAANRDPRQYEDPDVFRADRNPVGHLAFGSGIHLCPGTQLARMEGQAILR

EIVANIDRIEVVEPPTWTTNANLRGLTRLRVAVTPRVAP

>CYP128A1 (2622470838) Mycobacterium tuberculosis M1319

MTATQSPPEPAPDRVRLAGCPLAGTPDVGLTAQDATTALGVPTRRRASSG

GIPVATSMWRDAQTVRTYGPAVAKALALRVAGKARSRLTGRHCRKFMQLT

DFDPFDPAIAADPYPHYRELLAGERVQYNPKRDVYILSRYADVREAARNH

DTLSSSRGVTFSRGWLPFLPTSDPPAHTRMRKQLAPGMARGALETWRPMV

DQLARELVGGLLTQTPADVVSTVAAPMPMRAITSVLGVDGPDEAAFCRLS

NQAVRITDVALSASGLISLVQGFAGFRRLRALFTHRRDNGLLRECTVLGK

LATHAEQGRLSDDELFFFAVLLLVAGYESTAHMISTLFLTLADYPDQLTL

LAQQPDLIPSAIEEHLRFISPIQNICRTTRVDYSVGQAVIPAGSLVLLAW

GAANRDPRQYEDPDVFRADRNPVGHLAFGSGIHLCPGTQLARMEGQAILR

EIVANIDRIEVVEPPTWTTNANLRGLTRLRVAVTPRVAP

>CYP128A1 (2631906155) Mycobacterium tuberculosis MD18115

MTATQSPPEPAPDRVRLAGCPLAGTPDVGLTAQDATTALGVPTRRRASSG

GIPVATSMWRDAQTVRTYGPAVAKALALRVAGKARSRLTGRHCRKFMQLT

DFDPFDPAIAADPYPHYRELLAGERVQYNPKRDVYILSRYADVREAARNH

DTLSSARGVTFSRGWLPFLPTSDPPAHTRMRKQLAPGMARGALETWRPMV

DQLARELVGGLLTQTPADVVSTVAAPMPMRAITSVLGVDGPDEAAFCRLS

NQAVRITDVALSASGLISLVQGFAGFRRLRALFTHRRDNGLLRECTVLGK

LATHAEQGRLSDDELFFFAVLLLVAGYESTAHMISTLFLTLADYPDQLTL

LAQQPDLIPSAIEEHLRFISPIQNICRTTRVDYSVGQAVIPAGSLVLLAW

GAANRDPRQYEDPDVFRADRNPVGHLAFGSGIHLCPGTQLARMEGQAILR

EIVANIDRIEVVEPPTWTTNANLRGLTRLRVAVTPRVAP

>CYP128A1 (2661053197) Mycobacterium tuberculosis 1700302

MTATQSPPEPAPDRVRLAGCPLAGTPDVGLTAQDATTALGVPTRRRASSG

GIPVATSMWRDAQTVRTYGPAVAKALALRVAGKARSRLTGRHCRKFMQLT

DFDPFDPAIAADPYPHYRELLAGERVQYNPKRDVYILSRYADVREAARNH

DTLSSARGVTFSRGWLPFLPTSDPPAHTRMRKQLAPGMARGALETWRPMV

DQLARELVGGLLTQTPADVVSTVAAPMPMRAITSVLGVDGPDEAAFCRLS

NQAVRITDVALSASGLISLVQGFAGFRRLRALFTHRRDNGLLRECTVLGK

LATHAEQGRLSDDELFFFAVLLLVAGYESTAHMISTLFLTLADYPDQLTL

LAQQPDLIPSAIEEHLRFISPIQNICRTTRVDYSVGQAVIPAGSLVLLAW

GAANRDPRQYEDPDVFRADRNPVGHLAFGSGIHLCPGTQLARMEGQAILR

EIVANIDRIEVVEPPTWTTNANLRGLTRLRVAVTPRVAP

>CYP128A1 (2621526599) Mycobacterium tuberculosis XTB13-182

MTATQSPPEPAPDRVRLAGCPLAGTPDVGLTAQDATTALGVPTRRRAASG

GIPVATSMWRDAQTVRTYGPAVAKALALRVAGKARSRLTGRHCRKFMQLT

DFDPFDPAIAADPYPHYRELLAGERVQYNPKRDVYILSRYADVREAARNH

DTLSSARGVTFSRGWLPFLPTSDPPAHTRMRKQLAPGMARGALETWRPMV

DQLARELVGGLLTQTPADVVSTVAAPMPMRAITSVLGVDGPDEAAFCRLS

NQAVRITDVALSASGLISLVQGFAGFRRLRALFTHRRDNGLLRECTVLGK

LATHAEQGRLSDDELFFFAVLLLVAGYESTAHMISTLFLTLADYPDQLTL

LAQQPDLIPSAIEEHLRFISPIQNICRTTRVDYSVGQAVIPAGSLVLLAW

GAANRDPRQYEDPDVFRADRNPVGHLAFGSGIHLCPGTQLARMEGQAILR

EIVANIDRIEVVEPPTWTTNANLRGLTRLRVAVTPRVAP

>CYP128A1 (2810696391) Mycobacterium colombiense 1164983.0

MTATLSGAEEAEGWVPKGGECPFGRDASYSGPAARDLSTPIGDVATRRVP

TLAATMKRDAQLARACAPMLTKVLAITAARKVRSGLAGRHTPEHVQITEF

DPMNPAIARDPYPHYRELLAGERVHYNPKRDVYILSRYSDVREAARNHEM

LSSAEGVTFSRGAPPFLPTSDPPAHTRMRKQLAPGMARGALESWRPMVDQ

LARELVCDLKTRTTADVVSLVAAPMPIRTITNVLGIAGPDEAEFIRLSNQ

AARITDVNLSVSGLGSLVHGFTGFRRLRALFTHMRDNGLLGECTVLGQLA

GHADRGRLSDDELFLFAVLLVVAGHETTANMISTLFLTLAEYPEQLRLLA

QQPELIPSAIEEQLRFLPPVQNMCRTTRVDYPVGHAVIPAGSRVLLLWGA

ANRDPRQYDDPDVFRAERNPAGHLAFGSGIHSCPGTHLARMEGQAVLREI

VANLDRIEVVEPPTWTTNANLRGLVRLRVAVTPRATA

>CYP128B4 (2802877334) Mycobacteroides abscessus abscessus 1152

MSRWRSDSAVRQIGAPVVAAVGMNIAAAVRVRRRGYAGWTGAVNTDYDPL

DPATAAQPFDAYRALHAGGRVHYNPKRATFILSRHEDIRAALRDTDAVTS

SQGVTRMKISAPILVLTDGDDHTRLRKQVQPGFTRGAMSDWQGMADQLAK

ELVADVVANPGCDVMERLAVPLPIRMIAHIIGIPPEDVQNFRSWSEDGVG

VINAGVSPAGLRQGLKGVRAIAALRRYFKDQLASGKLKGSDTVLGRLVDN

NEDGKLSDDELFFIAMLLLFAGNETTTNLIGGMFDTLAHAPDQFAMIRDD

PDLIPSAVEEQLRYSAPIQNLYRYTRTDYRVGEVTIPSGSRLLLAFGAAN

RDPEVFEDPDTYRADRNPRNHIAFGYGVHMCIGATLSRMEGQAVLRELTS

QASAIAAAGSATWSTNSSLRGTTYLPIRLTPAR

>CYP128B2 (2810696296) Mycobacterium colombiense 1164983.0

MAVKNSPRGMGTLLKPLGSAIEMNLAGAIRTRRRGYRGWTGAVNTDYDPL

DPATAAQPHEAYRALHRSGRVHYNPKRATWILSRLNDVRAALRDTDQVTS

TRGVTRVRMAADLLVLTDGDEHARLRKQVQPAFTRGALENWQEIADKLAA

ELVSEVLAESGCDVVQRLAIPMPMRMIAAILGVPDTDIADFRRWSENSVR

IINFSPTPKGVVDIANSMRAVIALRRYFLRHLATGDLKGSGTVLGRLLQH

SSEGNLSDDQLFYVALLLLIAGNETTTNLLGGMFDTLAHNPDQYDLIRAN

PDLIPIAVEEQLRFSSPIQNLYRYTRADYHVGEVTIPAGARVLLSFGAAN

RDPLVFDEPDEYRADRNPRMHVGFGYGAHMCVGAPLARMEAQAVLRELVD

RGVTRITAVGPTTWSTNSSLRGPTHLPVCCTAAESDLSTPAR

>CYP128B2 (2810688718) Mycobacterium vulneris ICS2043

MFKPLTSAIQMNLSAAVRTRRLGYRGWAGAVNTDYDPLDPATAAQPFEAY

AQLHSSDRVHYSPRRATWILHRLEDVRAALRDTEQVTSSHGVTRIRMAAD

LVVVTDGEEHNRLRKQVQPAFTKRALDSWQRTVNDLAAELVDTLIAEPGS

DVVARLAIPMPLRLIAAILGVPERDIDDFRRWSENSVKLIDFTPTPAGVL

NTAKSLRAAMALRRYFLDHLATGELKGSDTVLGRLLEHNTDGTLTDDQLF

YIAILLLIAGNETTTNLLGGLFDTFARYPDQYDMIRAEPDLIPMAVEEQL

RYGSPIQNLYRYTRAPYRVGKVTIPSGSRLLLSFGAANRDPSAFDDPDTY

RADRNPRSHVAFGYGAHMCLGAPLARMEAVAVLRELVNRVSRISATAPTI

WSTNSSLRGPVHLPVRLHC

>CYP128A1 (2576602345) Mycobacterium tuberculosis MAL020179

MTATQSPPEPAPDRVRLAGCPLAGTPDVGLTAQDATTALGVPTRRRASSG

GIPVATSMWRDAQTVRTYGPAVAKALALRVAGKARSRLTGRHCRKFMQLT

DFDPFDPAIAADPYPHYRELLAGERVQYNPKRDVYILSRYADVREAARNH

DTLSSARGVTFSRGWLPFLPTSDPPAHTRMRKQLAPGMARGALETWRPMV

DQLARELVGGLLTQTPADVVSTVAAPMPMRAITSVLGVDGPDEAAFCRLS

NQAVRITDVALSASGLISLVQGFAGFRRLRALFTHRRDNGLLRECTVLGK

LATHAEQGRLSDDELFFFAVLLLVAGYESTAHMISTLFLTLADYPDQLTL

LAQQPDLIPSAIEEHLRFISPIQNICRTTRVDYSVGQAVIPAGSLVLLAW

GAANRDPRQYEDPDVFRADRNPVGHLAFGSGIHLCPGTQLARMEGQAILR

EIVANIDRIEVVEPPTWTTNANLRGLTRLRVAVTPRVAP

>CYP128A1 (2589732687) Mycobacterium tuberculosis TKK-01-0092

MTATQSPPEPAPDRVRLAGCPLAGTPDVGLTAQDATTALGVPTRRRASSG

GIPVATSMWRDAQTVRTYGPAVAKALALRVAGKARSRLTGRHCRKFMQLT

DFDPFDPAIAADPYPHYRELLAGERVQYNPKRDVYILSRYADVREAARNH

DTLSSARGVTFSRGWLPFLPTSDPPAHTRMRKQLAPGMARGALETWRPMV

DQLARELVGGLLTQTPADVVSTVAAPMPMRAITSVLGVDGPDEAAFCRLS

NQAVRITDVALSASGLISLVQGFAGFRRLRALFTHRRDNGLLRECTVLGK

LATHAEQGRLSDDELFFFAVLLLVAGYESTAHMISTLFLTLADYPDQLTL

LAQQPDLIPSAIEEHLRFISPIQNICRTTRVDYSVGQAVIPAGSLVLLAW

GAANRDPRQYEDPDVFRADRNPVGHLAFGSGIHLCPGTQLARMEGQAILR

EIVANIDRIEVVEPPTWTTNANLRGLTRLRVAVTPRVAP

>CYP128A1 (2590263914) Mycobacterium tuberculosis KT-0017

MTATQSPPEPAPDRVRLAGCPLAGTPDVGLTAQDATTALGVPTRRRASSG

GIPVATSMWRDAQTVRTYGPAVAKALALRVAGKARSRLTGRHCRKFMQLT

DFDPFDPAIAADPYPHYRELLAGERVQYNPKRDVYILSRYADVREAARNH

DTLSSARGVTFSRGWLPFLPTSDPPAHTRMRKQLAPGMARGALETWRPMV

DQLARELVGGLLTQTPADVVSTVAAPMPMRAITSVLGVDGPDEAAFCRLS

NQAVRITDVALSASGLISLVQGFAGFRRLRALFTHRRDNGLLRECTVLGK

LATHAEQGRLSDDELFFFAVLLLVAGYESTAHMISTLFLTLADYPDQLTL

LAQQPDLIPSAIEEHLRFISPIQNICRTTRVDYSVGQAVIPAGSLVLLAW

GAANRDPRQYEDPDVFRADRNPVGHLAFGSGIHLCPGTQLARMEGQAILR

EIVANIDRIEVVEPPTWTTNANLRGLTRLRVAVTPRVAP

>CYP128A1 (2590361968) Mycobacterium tuberculosis OFXR-25

MTATQSPPEPAPDRVRLAGCPLAGTPDVGLTAQDATTALGVPTRRRASSG

GIPVATSMWRDAQTVRTYGPAVAKALALRVAGKARSRLTGRHCRKFMQLT

DFDPFDPAIAADPYPHYRELLAGERVQYNPKRDVYILSRYADVREAARNH

DTLSSARGVTFSRGWLPFLPTSDPPAHTRMRKQLAPGMARGALETWRPMV

DQLARELVGGLLTQTPADVVSTVAAPMPMRAITSVLGVDGPDEAAFCRLS

NQAVRITDVALSASGLISLVQGFAGFRRLRALFTHRRDNGLLRECTVLGK

LATHAEQGRLSDDELFFFAVLLLVAGYESTAHMISTLFLTLADYPDQLTL

LAQQPDLIPSAIEEHLRFISPIQNICRTTRVDYSVGQAVIPAGSLVLLAW

GAANRDPRQYEDPDVFRADRNPVGHLAFGSGIHLCPGTQLARMEGQAILR

EIVANIDRIEVVEPPTWTTNANLRGLTRLRVAVTPRVAP

>CYP128A1 (2603443522) Mycobacterium tuberculosis TB_RSA89

MTATQSPPEPAPDRVRLAGCPLAGTPDVGLTAQDATTALGVPTRRRASSG

GIPVATSMWRDAQTVRTYGPAVAKALALRVAGKARSRLTGRHCRKFMQLT

DFDPFDPAIAADPYPHYRELLAGERVQYNPKRDVYILSRYADVREAARNH

DTLSSARGVTFSRGWLPFLPTSDPPAHTRMRKQLAPGMARGALETWRPMV

DQLARELVGGLLTQTPADVVSTVAAPMPMRAITSVLGVDGPDEAAFCRLS

NQAVRITDVALSASGLISLVQGFAGFRRLRALFTHRRDNGLLRECTVLGK

LATHAEQGRLSDDELFFFAVLLLVAGYESTAHMISTLFLTLADYPDQLTL

LAQQPDLIPSAIEEHLRFISPIQNICRTTRVDYSVGQAVIPAGSLVLLAW

GAANRDPRQYEDPDVFRADRNPVGHLAFGSGIHLCPGTQLARMEGQAILR

EIVANIDRIEVVEPPTWTTNANLRGLTRLRVAVTPRVAP

>CYP128A1 (2605655206) Mycobacterium tuberculosis BTB11-192

MTATQSPPEPAPDRVRLAGCPLAGTPDVGLTAQDATTALGVPTRRRASSG

GIPVATSMWRDAQTVRTYGPAVAKALALRVAGKARSRLTGRHCRKFMQLT

DFDPFDPAIAADPYPHYRELLAGERVQYNPKRDVYILSRYADVREAARNH

DTLSSARGVTFSRGWLPFLPTSDPPAHTRMRKQLAPGMARGALETWRPMV

DQLARELVGGLLTQTPADVVSTVAAPMPMRAITSVLGVDGPDEAAFCRLS

NQAVRITDVALSASGLISLVQGFAGFRRLRALFTHRRDNGLLRECTVLGK

LATHAEQGRLSDDELFFFAVLLLVAGYESTAHMISTLFLTLADYPDQLTL

LAQQPDLIPSAIEEHLRFISPIQNICRTTRVDYSVGQAVIPAGSLVLLAW

GAANRDPRQYEDPDVFRADRNPVGHLAFGSGIHLCPGTQLARMEGQAILR

EIVANIDRIEVVEPPTWTTNANLRGLTRLRVAVTPRVAP

>CYP128A1 (2642109449) Mycobacterium tuberculosis M2376

MTATQSPPEPAPDRVRLAGCPLAGTPDVGLTAQDATTALGVPTRRRASSG

GIPVATSMWRDAQTVRTYGPAVAKALALRVAGKARSRLTGRHCRKFMQLT

DFDPFDPAIAADPYPHYRELLAGERVQYNPKRDVYILSRYADVREAARNH

DTLSSARGVTFSRGWLPFLPTSDPPAHTRMRKQLAPGMARGALETWRPMV

DQLARELVGGLLTQTPADVVSTVAAPMPMRAITSVLGVDGPDEAAFCRLS

NQAVRITDVALSASGLISLVQGFAGFRRLRALFTHRRDNGLLRECTVLGK

LATHAEQGRLSDDELFFFAVLLLVAGYESTAHMISTLFLTLADYPDQLTL

LAQQPDLIPSAIEEHLRFISPIQNICRTTRVDYSVGQAVIPAGSLVLLAW

GAANRDPRQYEDPDVFRADRNPVGHLAFGSGIHLCPGTQLARMEGQAILR

EIVANIDRIEVVEPPTWTTNANLRGLTRLRVAVTPRVAP

>CYP128A1 (2669899411) Mycobacterium tuberculosis 301747

MTATQSPPEPAPDRVRLAGCPLAGTPDVGLTAQDATTALGVPTRRRASSG

GIPVATSMWRDAQTVRTYGPAVAKALALRVAGKARSRLTGRHCRKFMQLT

DFDPFDPAIAADPYPHYRELLAGERVQYNPKRDVYILSRYADVREAARNH

DTLSSARGVTFSRGWLPFLPTSDPPAHTRMRKQLAPGMARGALETWRPMV

DQLARELVGGLLTQTPADVVSTVAAPMPMRAITSVLGVDGPDEAAFCRLS

NQAVRITDVALSASGLISLVQGFAGFRRLRALFTHRRDNGLLRECTVLGK

LATHAEQGRLSDDELFFFAVLLLVAGYESTAHMISTLFLTLADYPDQLTL

LAQQPDLIPSAIEEHLRFISPIQNICRTTRVDYSVGQAVIPAGSLVLLAW

GAANRDPRQYEDPDVFRADRNPVGHLAFGSGIHLCPGTQLARMEGQAILR

EIVANIDRIEVVEPPTWTTNANLRGLTRLRVAVTPRVAP

>CYP128A1 (2696316614) Mycobacterium tuberculosis N00800620

MTATQSPPEPAPDRVRLAGCPLAGTPDVGLTAQDATTALGVPTRRRASSG

GIPVATSMWRDAQTVRTYGPAVAKALALRVAGKARSRLTGRHCRKFMQLT

DFDPFDPAIAADPYPHYRELLAGERVQYNPKRDVYILSRYADVREAARNH

DTLSSARGVTFSRGWLPFLPTSDPPAHTRMRKQLAPGMARGALETWRPMV

DQLARELVGGLLTQTPADVVSTVAAPMPMRAITSVLGVDGPDEAAFCRLS

NQAVRITDVALSASGLISLVQGFAGFRRLRALFTHRRDNGLLRECTVLGK

LATHAEQGRLSDDELFFFAVLLLVAGYESTAHMISTLFLTLADYPDQLTL

LAQQPDLIPSAIEEHLRFISPIQNICRTTRVDYSVGQAVIPAGSLVLLAW

GAANRDPRQYEDPDVFRADRNPVGHLAFGSGIHLCPGTQLARMEGQAILR

EIVANIDRIEVVEPPTWTTNANLRGLTRLRVAVTPRVAP

>CYP128A1 (2589682139) Mycobacterium tuberculosis TKK-01-0067

MTATQSPPEPAPDRVRLAGCPLAGTPDVGLTAQDATTALGVPTRRRASSG

GIPVATSMWRDAQTVRTYGPAVAKALALRVAGKARSRLTGRHCRKFMQLT

DFDPFDPAIAADPYPHYRELLAGERVQYNPKRDVYILSRYADVREAARNH

DTLSSARGVTFSRGWLPFLPTSDPPAHTRMRKQLAPGMARGALETWRPMV

DQLARELVGGLLTQTPADVVSTVAAPMPMRAITSVLGVDGPDEAAFCRLS

NQAVRITDVALSASGLISLVQGFAGFRRLRALFTHRRDNGLLRECTVLGK

LATHAEQGRLSDDELFFFAVLLLVAGYESPAHMISTLFLTLADYPDQLTL

LAQQPDLIPSAIEEHLRFISPIQNICRTTRVDYSVGQAVIPAGSLVLLAW

GAANRDPRQYEDPDVFRADRNPVGHLAFGSGIHLCPGTQLARMEGQAILR

EIVANIDRIEVVEPPTWTTNANLRGLTRLRVAVTPRVAP

>CYP128A1 (2677959470) Mycobacterium tuberculosis B00801080

MTATQSPPEPAPDRVRLAGCPLAGTPDVGLTAQDATTALGVPTRRRASSG

GIPVATSMWRDAQTVRTYGPAVAKALALRVAGKARSRLTGRHCRKFMQLT

DFDPFDPAIAADPYPHYRELLAGERVQYNPKRDVYILSRYADVREAARNH

QLARELVGGLLTQTPADVVSTVAAPMPMRAITSVLGVDGPDEAAFCRLS

NQAVRITDVALSASGLISLVQGFAGFRRLRALFTHRRDNGLLRECTVLGK

LATHAEQGRLSDDELFFFAVLLLVAGYESTAHMISTLFLTLADYPDQLTL

LAQQPDLIPSAIEEHLRFISPIQNICRTTRVDYSVGQAVIPAGSLVLLAW

GDTLSSARGVTFSRGWLPFLPTSDPPAHTRMRKQLAPGMARGALETWRPMV

DAANRDPRQYEDPDVFRADRNPVGHLAFGSGIHLCPGPSWRAWRVRRSCA

RSSPISTE

>CYP128B4 (2666184698) Mycobacterium abscessus PAP003

MSRWRSDSAVRQIGAPVVAAVGMNIAAAVRVRRRGYAGWTGAVNTDYDPL

DPATAAQPFDAYRALHAGGRVHYNPKRATFILSRHEDIRAALRDTDAVTS

SQGVTRMKISAPILVLTDGDDHTRLRKQVQPGFTRGAMSDWQGMADQLAK

ELVADVVANPGCDVMERLAVPLPIRMIAHIIGIPPEDVQNFRSWSEDGVG

VINAGVSPAGLRQGLKGVRAIAALRRYFKDQLASGKLKGSDTVLGRLVDN

NEDGKLSDDELFFIAMLLLFAGNETTTNLIGGMFDTLAHAPDQFAMIRDD

PDLIPSAVEEQLRYSAPIQNLYRYTRTDYRVGEVTIPSGSRLLLAFGAAN

RDPEVFEDPDTYRADRNPRNHIAFGYGVHMCIGATLSRMEGQAVLRELTS

QASAIAAAGSATWSTNSSLRGTTYLPIRLTPAR

>CYP128B4 (2697124848) Mycobacterium abscessus PAP046

MSRWRSDSAVRQIGAPVVAAVGMNIAAAVRVRRRGYAGWTGAVNTDYDPL

DPATAAQPFDAYRALHAGGRVHYNPKRATFILSRHEDIRAALRDTDAVTS

SQGVTRMKISAPILVLTDGDDHTRLRKQVQPGFTRGAMSDWQGMADQLAK

ELVADVVANPGCDVMERLAVPLPIRMIAHIIGIPPEDVQNFRSWSEDGVG

VINAGVSPAGLRQGLKGVRAIAALRRYFKDQLASGKLKGSDTVLGRLVDN

NEDGKLSDDELFFIAMLLLFAGNETTTNLIGGMFDTLAHAPDQFAMIRDD

PDLIPSAVEEQLRYSAPIQNLYRYTRTDYRVGEVTIPSGSRLLLAFGAAN

RDPEVFEDPDTYRADRNPRNHIAFGYGVHMCIGATLSRMEGQAVLRELTS

QASAIAAAGSATWSTNSSLRGTTYLPIRLTPAR

>CYP128A1 (2740567983) Mycobacterium kansasii 1010001458

VRSPGATRRLSRVASPCPLTTQFSGQLRLLARRPELIPSAIEEQLRFVSP

IQNICRTTRVDYPVGGAVIPAGSLVLLAWGAANRDPRQHDEPDVFRADRN

PTGLLEFGSGIHLCPGTQLARMEGHAVLREIVTNIDRIDVVEPPAWTTNA

NLRGPTRLRVAVTARASR

>CYP128A1 (2555583678) Mycobacterium tuberculosis PanR0410

MTATQSPPEPAPDRVRLAGCPLAGTPDVGLTAQDATTALGVPTRRRASSG

GIPVATSMWRDAQTVRTYGPAVAKALALRVAGKARSRLTGRHCRKFMQLT

DFDPFDPAIAADPYPHYRELLAGERVQYNPKRDVYILSRYADVREAARNH

DTLSSARGVTFSRGWLPFLPTSDPPAHTRMRKQLAPGMARGALETWRPMV

DQLARELVGGLLTQTPADVVSTVAAPMPMRAITSVLGVDGPDEAAFCRLS

NQAVRITDVALSASGLISLVQGFAGFRRLRALFTHRRDNGLLRECTVLGK

LATHAEQGRLSDDELFFFAVLLLVAGYESTAHMISTLFLTLADYPDQLTL

LAQQPDLIPSAIEEHLRFISPIQNICRTTRVDYSVGQAVIPAGSLVLLAW

GAANRDPRQYEDPDVFRADRNPVGHLAFGSGIHLCPGTQLARMEGQAILR

EIVANIDRIEVVEPPTWTTNANLRGLTRLRVAVTPRVAP

>CYP128A1 (2577099011) Mycobacterium tuberculosis BTB12-449

MTATQSPPEPAPDRVRLAGCPLAGTPDVGLTAQDATTALGVPTRRRASSG

GIPVATSMWRDAQTVRTYGPAVAKALALRVAGKARSRLTGRHCRKFMQLT

DFDPFDPAIAADPYPHYRELLAGERVQYNPKRDVYILSRYADVREAARNH

DTLSSARGVTFSRGWLPFLPTSDPPAHTRMRKQLAPGMARGALETWRPMV

DQLARELVGGLLTQTPADVVSTVAAPMPMRAITSVLGVDGPDEAAFCRLS

NQAVRITDVALSASGLISLVQGFAGFRRLRALFTHRRDNGLLRECTVLGK

LATHAEQGRLSDDELFFFAVLLLVAGYESTAHMISTLFLTLADYPDQLTL

LAQQPDLIPSAIEEHLRFISPIQNICRTTRVDYSVGQAVIPAGSLVLLAW

GAANRDPRQYEDPDVFRADRNPVGHLAFGSGIHLCPGTQLARMEGQAILR

EIVANIDRIEVVEPPTWTTNANLRGLTRLRVAVTPRVAP

>CYP128A1 (2589163093) Mycobacterium tuberculosis OFXR-4

MTATQSPPEPAPDRVRLAGCPLAGTPDVGLTAQDATTALGVPTRRRASSG

GIPVATSMWRDAQTVRTYGPAVAKALALRVAGKARSRLTGRHCRKFMQLT

DFDPFDPAIAADPYPHYRELLAGERVQYNPKRDVYILSRYADVREAARNH

DTLSSARGVTFSRGWLPFLPTSDPPAHTRMRKQLAPGMARGALETWRPMV

DQLARELVGGLLTQTPADVVSTVAAPMPMRAITSVLGVDGPDEAAFCRLS

NQAVRITDVALSASGLISLVQGFAGFRRLRALFTHRRDNGLLRECTVLGK

LATHAEQGRLSDDELFFFAVLLLVAGYESTAHMISTLFLTLADYPDQLTL

LAQQPDLIPSAIEEHLRFISPIQNICRTTRVDYSVGQAVIPAGSLVLLAW

GAANRDPRQYEDPDVFRADRNPVGHLAFGSGIHLCPGTQLARMEGQAILR

EIVANIDRIEVVEPPTWTTNANLRGLTRLRVAVTPRVAP

>CYP128A1 (2603214436) Mycobacterium tuberculosis TRUG0093

MTATQSPPEPAPDRVRLAGCPLAGTPDVGLTAQDATTALGVPTRRRASSG

GIPVATSMWRDAQTVRTYGPAVAKALALRVAGKARSRLTGRHCRKFMQLT

DFDPFDPAIAADPYPHYRELLAGERVQYNPKRDVYILSRYADVREAARNH

DTLSSARGVTFSRGWLPFLPTSDPPAHTRMRKQLAPGMARGALETWRPMV

DQLARELVGGLLTQTPADVVSTVAAPMPMRAITSVLGVDGPDEAAFCRLS

NQAVRITDVALSASGLISLVQGFAGFRRLRALFTHRRDNGLLRECTVLGK

LATHAEQGRLSDDELFFFAVLLLVAGYESTAHMISTLFLTLADYPDQLTL

LAQQPDLIPSAIEEHLRFISPIQNICRTTRVDYSVGQAVIPAGSLVLLAW

GAANRDPRQYEDPDVFRADRNPVGHLAFGSGIHLCPGTQLARMEGQAILR

EIVANIDRIEVVEPPTWTTNANLRGLTRLRVAVTPRVAP

>CYP128A1 (2603311467) Mycobacterium tuberculosis BTB05-481

MTATQSPPEPAPDRVRLAGCPLAGTPDVGLTAQDATTALGVPTRRRASSG

GIPVATSMWRDAQTVRTYGPAVAKALALRVAGKARSRLTGRHCRKFMQLT

DFDPFDPAIAADPYPHYRELLAGERVQYNPKRDVYILSRYADVREAARNH

DTLSSARGVTFSRGWLPFLPTSDPPAHTRMRKQLAPGMARGALETWRPMV

DQLARELVGGLLTQTPADVVSTVAAPMPMRAITSVLGVDGPDEAAFCRLS

NQAVRITDVALSASGLISLVQGFAGFRRLRALFTHRRDNGLLRECTVLGK

LATHAEQGRLSDDELFFFAVLLLVAGYESTAHMISTLFLTLADYPDQLTL

LAQQPDLIPSAIEEHLRFISPIQNICRTTRVDYSVGQAVIPAGSLVLLAW

GAANRDPRQYEDPDVFRADRNPVGHLAFGSGIHLCPGTQLARMEGQAILR

EIVANIDRIEVVEPPTWTTNANLRGLTRLRVAVTPRVAP

>CYP128A1 (2605643143) Mycobacterium tuberculosis BTB09-086

MTATQSPPEPAPDRVRLAGCPLAGTPDVGLTAQDATTALGVPTRRRASSG

GIPVATSMWRDAQTVRTYGPAVAKALALRVAGKARSRLTGRHCRKFMQLT

DFDPFDPAIAADPYPHYRELLAGERVQYNPKRDVYILSRYADVREAARNH

DTLSSARGVTFSRGWLPFLPTSDPPAHTRMRKQLAPGMARGALETWRPMV

DQLARELVGGLLTQTPADVVSTVAAPMPMRAITSVLGVDGPDEAAFCRLS

NQAVRITDVALSASGLISLVQGFAGFRRLRALFTHRRDNGLLRECTVLGK

LATHAEQGRLSDDELFFFAVLLLVAGYESTAHMISTLFLTLADYPDQLTL

LAQQPDLIPSAIEEHLRFISPIQNICRTTRVDYSVGQAVIPAGSLVLLAW

GAANRDPRQYEDPDVFRADRNPVGHLAFGSGIHLCPGTQLARMEGQAILR

EIVANIDRIEVVEPPTWTTNANLRGLTRLRVAVTPRVAP

>CYP128A1 (2670579514) Mycobacterium tuberculosis A70757

MTATQSPPEPAPDRVRLAGCPLAGTPDVGLTAQDATTALGVPTRRRASSG

GIPVATSMWRDAQTVRTYGPAVAKALALRVAGKARSRLTGRHCRKFMQLT

DFDPFDPAIAADPYPHYRELLAGERVQYNPKRDVYILSRYADVREAARNH

DTLSSARGVTFSRGWLPFLPTSDPPAHTRMRKQLAPGMARGALETWRPMV

DQLARELVGGLLTQTPADVVSTVAAPMPMRAITSVLGVDGPDEAAFCRLS

NQAVRITDVALSASGLISLVQGFAGFRRLRALFTHRRDNGLLRECTVLGK

LATHAEQGRLSDDELFFFAVLLLVAGYESTAHMISTLFLTLADYPDQLTL

LAQQPDLIPSAIEEHLRFISPIQNICRTTRVDYSVGQAVIPAGSLVLLAW

GAANRDPRQYEDPDVFRADRNPVGHLAFGSGIHLCPGTQLARMEGQAILR

EIVANIDRIEVVEPPTWTTNANLRGLTRLRVAVTPRVAP

>CYP128A1 (2673318087) Mycobacterium tuberculosis P09501258

MTATQSPPEPAPDRVRLAGCPLAGTPDVGLTAQDATTALGVPTRRRASSG

GIPVATSMWRDAQTVRTYGPAVAKALALRVAGKARSRLTGRHCRKFMQLT

DFDPFDPAIAADPYPHYRELLAGERVQYNPKRDVYILSRYADVREAARNH

DTLSSARGVTFSRGWLPFLPTSDPPAHTRMRKQLAPGMARGALETWRPMV

DQLARELVGGLLTQTPADVVSTVAAPMPMRAITSVLGVDGPDEAAFCRLS

NQAVRITDVALSASGLISLVQGFAGFRRLRALFTHRRDNGLLRECTVLGK

LATHAEQGRLSDDELFFFAVLLLVAGYESTAHMISTLFLTLADYPDQLTL

LAQQPDLIPSAIEEHLRFISPIQNICRTTRVDYSVGQAVIPAGSLVLLAW

GAANRDPRQYEDPDVFRADRNPVGHLAFGSGIHLCPGTQLARMEGQAILR

EIVANIDRIEVVEPPTWTTNANLRGLTRLRVAVTPRVAP

>CYP128A1 (2693204366) Mycobacterium tuberculosis J09800554

MTATQSPPEPAPDRVRLAGCPLAGTPDVGLTAQDATTALGVPTRRRASSG

GIPVATSMWRDAQTVRTYGPAVAKALALRVAGKARSRLTGRHCRKFMQLT

DFDPFDPAIAADPYPHYRELLAGERVQYNPKRDVYILSRYADVREAARNH

DTLSSARGVTFSRGWLPFLPTSDPPAHTRMRKQLAPGMARGALETWRPMV

DQLARELVGGLLTQTPADVVSTVAAPMPMRAITSVLGVDGPDEAAFCRLS

NQAVRITDVALSASGLISLVQGFAGFRRLRALFTHRRDNGLLRECTVLGK

LATHAEQGRLSDDELFFFAVLLLVAGYESTAHMISTLFLTLADYPDQLTL

LAQQPDLIPSAIEEHLRFISPIQNICRTTRVDYSVGQAVIPAGSLVLLAW

GAANRDPRQYEDPDVFRADRNPVGHLAFGSGIHLCPGTQLARMEGQAILR

EIVANIDRIEVVEPPTWTTNANLRGLTRLRVAVTPRVAP

>CYP128A1 (2699765601) Mycobacterium caprae MB2

MTATQSPPEPAPDRVRLAGCPLAGTPDVGLTAQDATTALGVPTRRRASSG

GIPVATSMWRDAQTVRTYGPAVAKALALRVAGKARSRLTGRHCRKFMQLT

DFDPFDPAIAADPYPHYRELLAGERVQYNPKRDVYILSRYADVREAARNH

DTLSSARGVTFSRGWLPFLPTSDPPAHTRMRKQLAPGMARGALETWRPMV

DQLARELVGGLLTQTPADVVSTVAAPMPMRAITSVLGVDGPDEAAFCRLS

NQAVRITDVALSASGLISLVQGFAGFRRLRALFTHRRDNGLLRECTVLGK

LATHAEQGRLSDDELFFFAVLLLVAGYESTAHMISTLFLTLADYPDQLTL

LAQQPDLIPSAIEEHLRFISPIQNICRTTRVDYSVGQAVIPAGSLVLLAW

GAANRDPRQYEDPDVFRADRNPVGHLAFGSGIHLCPGTQLARMEGQAILR

EIVANIDRIEVVEPPTWTTNANLRGLTRLRVAVTPPASHHEGSPFVVSTF

PVARVGRTAVGRLQLLGLDAAFDP

>CYP128B4 (2797931145) Mycobacteroides abscessus abscessus 481

MSRWRSDSAVRQIGAPVVAAVGMNIAAAVRVRRRGYAGWTGAVNTDYDPL

DPATAAQPFDAYRALHAGGRVHYNPKRATFILSRHEDIRAALRDTDAVTS

SQGVTRMKISAPILVLTDGDDHTRLRKQVQPGFTRGAMSDWQGMADQLAK

ELVADVVANPGCDVMERLAVPLPIRMIAHIIGIPPEDVQNFRSWSEDGVG

VINAGVSPAGLRQGLKGVRAIAALRRYFKDQLASGKLKGSDTVLGRLVDN

NEDGKLSDDELFFIAMLLLFAGNETTTNLIGGMFDTLAHAPDQFAMIRDD

PDLIPSAVEEQLRYSAPIQNLYRYTRTDYRVGEVTIPSGSRLLLAFGAAN

RDPEVFEDPDTYRADRNPRNHIAFGYGVHMCIGATLSRMEGQAVLRELTS

QASAIAAAGSATWSTNSSLRGTTYLPIRLTPAR

>CYP128B1 (2621120977) Mycobacterium asiaticum 3056

MAGKQAFQGTAEGARLLGHAARMNLAAAVRTRRRGYAGWTGAINTDYDPQ

DPLTAAQPFDAYRALHGGGRVHYNPRRATFIISRLDDVRAALRDTDQVTS

SQGVTRLRMSAPLAVLTDGEEHARLRRQVQPGFSKGAMNAWQGMIEQLAE

ELVGDVLANPGCDVVRRLAIPMPIRLIAQILGIPNDDVGDFRRWSERGVG

VMDVTPTLPGLIGAARSVTAMAALQRYFVKQFTAGELKGSNTVLGRLLAH

NTDGSLTDRQLLLIAIHLLIAGNETTTNLLGGMFDTLAHHPDQYEMIRAQ

PDLVPLAVEEQLRTTTPIQNLYRYTRADYQVGDVTIPNGSRVLLSFGAAN

RDPTAFEEPDQYRADRNPRTHVAFGYGAHMCLGAPLARMEAQAVLRQLIT

RVSRITPAGPTTWSTHSSLRGPTRLPVRLTAA

>CYP128A1 (2589057084) Mycobacterium tuberculosis TBR29

MTATQSPPEPAPDRVRLAGCPLAGTPDVGLTAQDATTALGVPTRRRASSG

GIPVATSMWRDAQTVRTYGPAVAKALALRVAGKARSRLTGRHCRKFMQLT

DFDPFDPAIAADPYPHYRELLAGERVQYNPKRDVYILSRYADVREAARNH

DTLSSARGVTFSRGWLPFLPTSDPPAHTRMRKQLAPGMARGALETWRPMV

DQLARELVGGLLTQTPADVVSTVAAPMPMRAITSVLGVDGPDEAAFCRLS

NQAVRITDVALSASGLISLVQGFAGFRRLRALFTHRRDNGLLRECTVLGK

LATHAEQGRLSDDELFFFAVLLLVAGYESTAHMISTLFLTLADYPDQLTL

LAQQPDLIPSAIEEHLRFISPIQNICRTTRVDYSVGQAVIPAGSLVLLAW

GAANRDPRQYEDPDVFRADRNPVGHLAFGSGIHLCPGTQLARMEGQAILR

EIVANIDRIEVVEPPTWTTNANLRGLTRLRVAVTPRVAP

>CYP128A1 (2604656585) Mycobacterium tuberculosis H37Rv

MTATQSPPEPAPDRVRLAGCPLAGTPDVGLTAQDATTALGVPTRRRASSG

GIPVATSMWRDAQTVRTYGPAVAKALALRVAGKARSRLTGRHCRKFMQLT

DFDPFDPAIAADPYPHYRELLAGERVQYNPKRDVYILSRYADVREAARNH

DTLSSARGVTFSRGWLPFLPTSDPPAHTRMRKQLAPGMARGALETWRPMV

DQLARELVGGLLTQTPADVVSTVAAPMPMRAITSVLGVDGPDEAAFCRLS

NQAVRITDVALSASGLISLVQGFAGFRRLRALFTHRRDNGLLRECTVLGK

LATHAEQGRLSDDELFFFAVLLLVAGYESTAHMISTLFLTLADYPDQLTL

LAQQPDLIPSAIEEHLRFISPIQNICRTTRVDYSVGQAVIPAGSLVLLAW

GAANRDPRQYEDPDVFRADRNPVGHLAFGSGIHLCPGTQLARMEGQAILR

EIVANIDRIEVVEPPTWTTNANLRGLTRLRVAVTPRVAP

>CYP128A1 (2605349603) Mycobacterium tuberculosis TB_RSA103

MTATQSPPEPAPDRVRLAGCPLAGTPDVGLTAQDATTALGVPTRRRASSG

GIPVATSMWRDAQTVRTYGPAVAKALALRVAGKARSRLTGRHCRKFMQLT

DFDPFDPAIAADPYPHYRELLAGERVQYNPKRDVYILSRYADVREAARNH

DTLSSARGVTFSRGWLPFLPTSDPPAHTRMRKQLAPGMARGALETWRPMV

DQLARELVGGLLTQTPADVVSTVAAPMPMRAITSVLGVDGPDEAAFCRLS

NQAVRITDVALSASGLISLVQGFAGFRRLRALFTHRRDNGLLRECTVLGK

LATHAEQGRLSDDELFFFAVLLLVAGYESTAHMISTLFLTLADYPDQLTL

LAQQPDLIPSAIEEHLRFISPIQNICRTTRVDYSVGQAVIPAGSLVLLAW

GAANRDPRQYEDPDVFRADRNPVGHLAFGSGIHLCPGTQLARMEGQAILR

EIVANIDRIEVVEPPTWTTNANLRGLTRLRVAVTPRVAP

>CYP128A1 (2605764794) Mycobacterium tuberculosis TB_RSA63

MTATQSPPEPAPDRVRLAGCPLAGTPDVGLTAQDATTALGVPTRRRASSG

GIPVATSMWRDAQTVRTYGPAVAKALALRVAGKARSRLTGRHCRKFMQLT

DFDPFDPAIAADPYPHYRELLAGERVQYNPKRDVYILSRYADVREAARNH

DTLSSARGVTFSRGWLPFLPTSDPPAHTRMRKQLAPGMARGALETWRPMV

DQLARELVGGLLTQTPADVVSTVAAPMPMRAITSVLGVDGPDEAAFCRLS

NQAVRITDVALSASGLISLVQGFAGFRRLRALFTHRRDNGLLRECTVLGK

LATHAEQGRLSDDELFFFAVLLLVAGYESTAHMISTLFLTLADYPDQLTL

LAQQPDLIPSAIEEHLRFISPIQNICRTTRVDYSVGQAVIPAGSLVLLAW

GAANRDPRQYEDPDVFRADRNPVGHLAFGSGIHLCPGTQLARMEGQAILR

EIVANIDRIEVVEPPTWTTNANLRGLTRLRVAVTPRVAP

>CYP128A1 (2622239417) Mycobacterium tuberculosis MD15765

MTATQSPPEPAPDRVRLAGCPLAGTPDVGLTAQDATTALGVPTRRRASSG

GIPVATSMWRDAQTVRTYGPAVAKALALRVAGKARSRLTGRHCRKFMQLT

DFDPFDPAIAADPYPHYRELLAGERVQYNPKRDVYILSRYADVREAARNH

DTLSSARGVTFSRGWLPFLPTSDPPAHTRMRKQLAPGMARGALETWRPMV

DQLARELVGGLLTQTPADVVSTVAAPMPMRAITSVLGVDGPDEAAFCRLS

NQAVRITDVALSASGLISLVQGFAGFRRLRALFTHRRDNGLLRECTVLGK

LATHAEQGRLSDDELFFFAVLLLVAGYESTAHMISTLFLTLADYPDQLTL

LAQQPDLIPSAIEEHLRFISPIQNICRTTRVDYSVGQAVIPAGSLVLLAW

GAANRDPRQYEDPDVFRADRNPVGHLAFGSGIHLCPGTQLARMEGQAILR

EIVANIDRIEVVEPPTWTTNANLRGLTRLRVAVTPRVAP

>CYP128A1 (2622283975) Mycobacterium tuberculosis H2264

MTATQSPPEPAPDRVRLAGCPLAGTPDVGLTAQDATTALGVPTRRRASSG

GIPVATSMWRDAQTVRTYGPAVAKALALRVAGKARSRLTGRHCRKFMQLT

DFDPFDPAIAADPYPHYRELLAGERVQYNPKRDVYILSRYADVREAARNH

DTLSSARGVTFSRGWLPFLPTSDPPAHTRMRKQLAPGMARGALETWRPMV

DQLARELVGGLLTQTPADVVSTVAAPMPMRAITSVLGVDGPDEAAFCRLS

NQAVRITDVALSASGLISLVQGFAGFRRLRALFTHRRDNGLLRECTVLGK

LATHAEQGRLSDDELFFFAVLLLVAGYESTAHMISTLFLTLADYPDQLTL

LAQQPDLIPSAIEEHLRFISPIQNICRTTRVDYSVGQAVIPAGSLVLLAW

GAANRDPRQYEDPDVFRADRNPVGHLAFGSGIHLCPGTQLARMEGQAILR

EIVANIDRIEVVEPPTWTTNANLRGLTRLRVAVTPRVAP

>CYP128A1 (2632682653) Mycobacterium tuberculosis KT-0202

MTATQSPPEPAPDRVRLAGCPLAGTPDVGLTAQDATTALGVPTRRRASSG

GIPVATSMWRDAQTVRTYGPAVAKALALRVAGKARSRLTGRHCRKFMQLT

DFDPFDPAIAADPYPHYRELLAGERVQYNPKRDVYILSRYADVREAARNH

DTLSSARGVTFSRGWLPFLPTSDPPAHTRMRKQLAPGMARGALETWRPMV

DQLARELVGGLLTQTPADVVSTVAAPMPMRAITSVLGVDGPDEAAFCRLS

NQAVRITDVALSASGLISLVQGFAGFRRLRALFTHRRDNGLLRECTVLGK

LATHAEQGRLSDDELFFFAVLLLVAGYESTAHMISTLFLTLADYPDQLTL

LAQQPDLIPSAIEEHLRFISPIQNICRTTRVDYSVGQAVIPAGSLVLLAW

GAANRDPRQYEDPDVFRADRNPVGHLAFGSGIHLCPGTQLARMEGQAILR

EIVANIDRIEVVEPPTWTTNANLRGLTRLRVAVTPRVAP

>CYP128A1 (2683869418) Mycobacterium tuberculosis N00401131

MTATQSPPEPAPDRVRLAGCPLAGTPDVGLTAQDATTALGVPTRRRASSG

GIPVATSMWRDAQTVRTYGPAVAKALALRVAGKARSRLTGRHCRKFMQLT

DFDPFDPAIAADPYPHYRELLAGERVQYNPKRDVYILSRYADVREAARNH

DTLSSARGVTFSRGWLPFLPTSDPPAHTRMRKQLAPGMARGALETWRPMV

DQLARELVGGLLTQTPADVVSTVAAPMPMRAITSVLGVDGPDEAAFCRLS

NQAVRITDVALSASGLISLVQGFAGFRRLRALFTHRRDNGLLRECTVLGK

LATHAEQGRLSDDELFFFAVLLLVAGYESTAHMISTLFLTLADYPDQLTL

LAQQPDLIPSAIEEHLRFISPIQNICRTTRVDYSVGQAVIPAGSLVLLAW

GAANRDPRQYEDPDVFRADRNPVGHLAFGSGIHLCPGTQLARMEGQAILR

EIVANIDRIEVVEPPTWTTNANLRGLTRLRVAVTPRVAP

>CYP128A1 (2724808702) Mycobacterium tuberculosis C_913

MTATQSPPEPAPDRVRLAGCPLAGTPDVGLTAQDATTALGVPTRRRASSG

GIPVATSMWRDAQTVRTYGPAVAKALALRVAGKARSRLTGRHCRKFMQLT

DFDPFDPAIAADPYPHYRELLAGERVQYNPKRDVYILSRYADVREAARNH

DTLSSARGVTFSRGWLPFLPTSDPPAHTRMRKQLAPGMARGALETWRPMV

DQLARELVGGLLTQTPADVVSTVAAPMPMRAITSVLGVDGPDEAAFCRLS

NQAVRITDVALSASGLISLVQGFAGFRRLRALFTHRRDNGLLRECTVLGK

LATHAEQGRLSDDELFFFAVLLLVAGYESTAHMISTLFLTLADYPDQLTL

LAQQPDLIPSAIEEHLRFISPIQNICRTTRVDYSVGQAVIPAGSLVLLAW

GAANRDPRQYEDPDVFRADRNPVGHLAFGSGIHLCPGTQLARMEGQAILR

EIVANIDRIEVVEPPTWTTNANLRGLTRLRVAVTPRVAP

>CYP128B4 (2693494799) Mycobacterium abscessus PAP047

MSRWRSDSAVRQIGAPVVAAVGMNIAAAVRVRRRGYAGWTGAVNTDYDPL

DPATAAQPFDAYRALHAGGRVHYNPKRATFILSRHEDIRAALRDTDAVTS

SQGVTRMKISAPILVLTDGDDHTRLRKQVQPGFTRGAMSDWQGMADQLAK

ELVADVVANPGCDVMERLAVPLPIRMIAHIIGIPPEDVQNFRSWSEDGVG

VINAGVSPAGLRQGLKGVRAIAALRRYFKDQLASGKLKGSDTVLGRLVDN

NEDGKLSDDELFFIAMLLLFAGNETTTNLIGGMFDTLAHAPDQFAMIRDD

PDLIPSAVEEQLRYSAPIQNLYRYTRTDYRVGEVTIPSGSRLLLAFGAAN

RDPEVFEDPDTYRADRNPRNHIAFGYGVHMCIGATLSRMEGQAVLRELTS

QASAIAAAGSATWSTNSSLRGTTYLPIRLTPAR

>CYP128B4 (2803433257) Mycobacteroides abscessus abscessus 270

MSRWRSDSAVRQIGAPVVAAVGMNIAAAVRVRRRGYAGWTGAVNTDYDPL

DPATAAQPFDAYRALHAGGRVHYNPKRATFILSRHEDIRAALRDTDAVTS

SQGVTRMKISAPILVLTDGDDHTRLRKQVQPGFTRGAMSDWQGMADQLAK

ELVADVVANPGCDVMERLAVPLPIRMIAHIIGIPPEDVQNFRSWSEDGVG

VINAGVSPAGLRQGLKGVRAIAALRRYFKDQLASGKLKGSDTVLGRLVDN

NEDGKLSDDELFFIAMLLLFAGNETTTNLIGGMFDTLAHAPDQFAMIRDD

PDLIPSAVEEQLRYSAPIQNLYRYTRTDYRVGEVTIPSGSRLLLAFGAAN

RDPEVFEDPDTYRADRNPRNHIAFGYGVHMCIGATLSRMEGQAVLRELTS

QASAIAAAGSATWSTNSSLRGTTYLPIRLTPAR

>CYP128B1 (2657977459) Mycobacterium sp. GA-1285

MAGMQITPGVIGPFANAVRMNVVAAVQNRARGYRGWTGAVNTDFDPMDPA

TASAPFEAYRRLHRSGRVHYNPRRSNFILSRLDDVRTALRDTEYVTSAEG

VTRLKFSAPLAVLTDGDEHARLRKQVQPGFSKGAMDSWKGMTEKLATELV

TEVIDDPGCDVVQRLAIPMPIRMIAQILGIPECDADDFRRWSEAGVGIMN

FSPTPRGVIDAVKSMAAMGALRRYFLNQFAHGGLKGSGTVLGRLLEHNSD

GSLTDEQLFLIAIHLLIAGNETTTNLLGGMFDTLAHNPDQYDMIRADPDL

IPMAVEEQLRVTTPIQNLYRYTCADYEIAGVTIPRGSRVLLLFGAANRDP

IAFEDPDQYRADRNPQTHIAFGYGAHMCLGAPLARMEAQAVLRELVSRVS

RIDAVGDTTWSRHSSLRGPTRLAVRLTPVR

>CYP128B1 (2807150093) Mycobacterium mantenii E2660

MSISQTVQGAAEAIGPFSNAVRMNLAAAIRTRRRGYDAWTGAVNTDYDPQ

NPVTAAQPFDAYRALHRTGRVHYNPRRATWIVSRLDDVRAALRDTDQVTS

TQGVTRLRMSAPLAVLTDGEEHARLRKQIQPGFSKGAMSAWQEIVDKLAV

ELVSDLLNNPGCDVVRHLAIPMPIRLIAQILGVPEDDVNDFRGWSENAVK

VMELTPTRAALTEAARSISAMVALQRYFVKQFARGGLKGSGTVLGRLLEH

NTDGSLTDRQLWLIAIHLLIAGNETTTNLLGGMFDTLAHHPDQYDLIRAN

PNLIPIAVEEQLRITTPIQNLYRYTRADYQIGDITIPAGSRVLLSFGAAN

RDPAVFDEPDEYRADRNPRTHVAFGYGAHMCIGAPLARMEAHAVLRQLVT

HVSRLSPNGSTTWSTHSSLRGPTHLPIHLTPA

>CYP128A1 (2555338399) Mycobacterium tuberculosis PanR0602

MTATQSPPEPAPDRVRLAGCPLAGTPDVGLTAQDATTALGVPTRRRASSG

GIPVATSMWRDAQTVRTYGPAVAKALALRVAGKARSRLTGRHCRKFMQLT

DFDPFDPAIAADPYPHYRELLAGERVQYNPKRDVYILSRYADVREAARNH

DTLSSARGVTFSRGWLPFLPTSDPPAHTRMRKQLAPGMARGALETWRPMV

DQLARELVGGLLTQTPADVVSTVAAPMPMRAITSVLGVDGPDEAAFCRLS

NQAVRITDVALSASGLISLVQGFAGFRRLRALFTHRRDNGLLRECTVLGK

LATHAEQGRLSDDELFFFAVLLLVAGYESTAHMISTLFLTLADYPDQLTL

LAQQPDLIPSAIEEHLRFISPIQNICRTTRVDYSVGQAVIPAGSLVLLAW

GAANRDPRQYEDPDVFRADRNPVGHLAFGSGIHLCPGTQLARMEGQAILR

EIVANIDRIEVVEPPTWTTNANLRGLTRLRVAVTPRVAP

>CYP128A1 (2555516661) Mycobacterium tuberculosis PanR0405

MTATQSPPEPAPDRVRLAGCPLAGTPDVGLTAQDATTALGVPTRRRASSG

GIPVATSMWRDAQTVRTYGPAVAKALALRVAGKARSRLTGRHCRKFMQLT

DFDPFDPAIAADPYPHYRELLAGERVQYNPKRDVYILSRYADVREAARNH

DTLSSARGVTFSRGWLPFLPTSDPPAHTRMRKQLAPGMARGALETWRPMV

DQLARELVGGLLTQTPADVVSTVAAPMPMRAITSVLGVDGPDEAAFCRLS

NQAVRITDVALSASGLISLVQGFAGFRRLRALFTHRRDNGLLRECTVLGK

LATHAEQGRLSDDELFFFAVLLLVAGYESTAHMISTLFLTLADYPDQLTL

LAQQPDLIPSAIEEHLRFISPIQNICRTTRVDYSVGQAVIPAGSLVLLAW

GAANRDPRQYEDPDVFRADRNPVGHLAFGSGIHLCPGTQLARMEGQAILR

EIVANIDRIEVVEPPTWTTNANLRGLTRLRVAVTPRVAP

>CYP128A1 (2576106269) Mycobacterium tuberculosis TRUG0037

MTATQSPPEPAPDRVRLAGCPLAGTPDVGLTAQDATTALGVPTRRRASSG

GIPVATSMWRDAQTVRTYGPAVAKALALRVAGKARSRLTGRHCRKFMQLT

DFDPFDPAIAADPYPHYRELLAGERVQYNPKRDVYILSRYADVREAARNH

DTLSSARGVTFSRGWLPFLPTSDPPAHTRMRKQLAPGMARGALETWRPMV

DQLARELVGGLLTQTPADVVSTVAAPMPMRAITSVLGVDGPDEAAFCRLS

NQAVRITDVALSASGLISLVQGFAGFRRLRALFTHRRDNGLLRECTVLGK

LATHAEQGRLSDDELFFFAVLLLVAGYESTAHMISTLFLTLADYPDQLTL

LAQQPDLIPSAIEEHLRFISPIQNICRTTRVDYSVGQAVIPAGSLVLLAW

GAANRDPRQYEDPDVFRADRNPVGHLAFGSGIHLCPGTQLARMEGQAILR

EIVANIDRIEVVEPPTWTTNANLRGLTRLRVAVTPRVAP

>CYP128A1 (2576247773) Mycobacterium tuberculosis TKK_04_0103

MTATQSPPEPAPDRVRLAGCPLAGTPDVGLTAQDATTALGVPTRRRASSG

GIPVATSMWRDAQTVRTYGPAVAKALALRVAGKARSRLTGRHCRKFMQLT

DFDPFDPAIAADPYPHYRELLAGERVQYNPKRDVYILSRYADVREAARNH

DTLSSARGVTFSRGWLPFLPTSDPPAHTRMRKQLAPGMARGALETWRPMV

DQLARELVGGLLTQTPADVVSTVAAPMPMRAITSVLGVDGPDEAAFCRLS

NQAVRITDVALSASGLISLVQGFAGFRRLRALFTHRRDNGLLRECTVLGK

LATHAEQGRLSDDELFFFAVLLLVAGYESTAHMISTLFLTLADYPDQLTL

LAQQPDLIPSAIEEHLRFISPIQNICRTTRVDYSVGQAVIPAGSLVLLAW

GAANRDPRQYEDPDVFRADRNPVGHLAFGSGIHLCPGTQLARMEGQAILR

EIVANIDRIEVVEPPTWTTNANLRGLTRLRVAVTPRVAP

>CYP128A1 (2577199325) Mycobacterium tuberculosis MD15974

MTATQSPPEPAPDRVRLAGCPLAGTPDVGLTAQDATTALGVPTRRRASSG

GIPVATSMWRDAQTVRTYGPAVAKALALRVAGKARSRLTGRHCRKFMQLT

DFDPFDPAIAADPYPHYRELLAGERVQYNPKRDVYILSRYADVREAARNH

DTLSSARGVTFSRGWLPFLPTSDPPAHTRMRKQLAPGMARGALETWRPMV

DQLARELVGGLLTQTPADVVSTVAAPMPMRAITSVLGVDGPDEAAFCRLS

NQAVRITDVALSASGLISLVQGFAGFRRLRALFTHRRDNGLLRECTVLGK

LATHAEQGRLSDDELFFFAVLLLVAGYESTAHMISTLFLTLADYPDQLTL

LAQQPDLIPSAIEEHLRFISPIQNICRTTRVDYSVGQAVIPAGSLVLLAW

GAANRDPRQYEDPDVFRADRNPVGHLAFGSGIHLCPGTQLARMEGQAILR

EIVANIDRIEVVEPPTWTTNANLRGLTRLRVAVTPRVAP

>CYP128A1 (2577516677) Mycobacterium tuberculosis TKK_04_0132

MTATQSPPEPAPDRVRLAGCPLAGTPDVGLTAQDATTALGVPTRRRASSG

GIPVATSMWRDAQTVRTYGPAVAKALALRVAGKARSRLTGRHCRKFMQLT

DFDPFDPAIAADPYPHYRELLAGERVQYNPKRDVYILSRYADVREAARNH

DTLSSARGVTFSRGWLPFLPTSDPPAHTRMRKQLAPGMARGALETWRPMV

DQLARELVGGLLTQTPADVVSTVAAPMPMRAITSVLGVDGPDEAAFCRLS

NQAVRITDVALSASGLISLVQGFAGFRRLRALFTHRRDNGLLRECTVLGK

LATHAEQGRLSDDELFFFAVLLLVAGYESTAHMISTLFLTLADYPDQLTL

LAQQPDLIPSAIEEHLRFISPIQNICRTTRVDYSVGQAVIPAGSLVLLAW

GAANRDPRQYEDPDVFRADRNPVGHLAFGSGIHLCPGTQLARMEGQAILR

EIVANIDRIEVVEPPTWTTNANLRGLTRLRVAVTPRVAP

>CYP128A1 (2622457011) Mycobacterium tuberculosis

MTATQSPPEPAPDRVRLAGCPLAGTPDVGLTAQDATTALGVPTRRRASSG

GIPVATSMWRDAQTVRTYGPAVAKALALRVAGKARSRLTGRHCRKFMQLT

DFDPFDPAIAADPYPHYRELLAGERVQYNPKRDVYILSRYADVREAARNH

DTLSSARGVTFSRGWLPFLPTSDPPAHTRMRKQLAPGMARGALETWRPMV

DQLARELVGGLLTQTPADVVSTVAAPMPMRAITSVLGVDGPDEAAFCRLS

NQAVRITDVALSASGLISLVQGFAGFRRLRALFTHRRDNGLLRECTVLGK

LATHAEQGRLSDDELFFFAVLLLVAGYESTAHMISTLFLTLADYPDQLTL

LAQQPDLIPSAIEEHLRFISPIQNICRTTRVDYSVGQAVIPAGSLVLLAW

GAANRDPRQYEDPDVFRADRNPVGHLAFGSGIHLCPGTQLARMEGQAILR

EIVANIDRIEVVEPPTWTTNANLRGLTRLRVAVTPRVAP

>CYP128A1 (2641038739) Mycobacterium tuberculosis TKK_03_0023

MTATQSPPEPAPDRVRLAGCPLAGTPDVGLTAQDATTALGVPTRRRASSG

GIPVATSMWRDAQTVRTYGPAVAKALALRVAGKARSRLTGRHCRKFMQLT

DFDPFDPAIAADPYPHYRELLAGERVQYNPKRDVYILSRYADVREAARNH

DTLSSARGVTFSRGWLPFLPTSDPPAHTRMRKQLAPGMARGALETWRPMV

DQLARELVGGLLTQTPADVVSTVAAPMPMRAITSVLGVDGPDEAAFCRLS

NQAVRITDVALSASGLISLVQGFAGFRRLRALFTHRRDNGLLRECTVLGK

LATHAEQGRLSDDELFFFAVLLLVAGYESTAHMISTLFLTLADYPDQLTL

LAQQPDLIPSAIEEHLRFISPIQNICRTTRVDYSVGQAVIPAGSLVLLAW

GAANRDPRQYEDPDVFRADRNPVGHLAFGSGIHLCPGTQLARMEGQAILR

EIVANIDRIEVVEPPTWTTNANLRGLTRLRVAVTPRVAP

>CYP128A1 (2657825766) Mycobacterium tuberculosis 401325

MTATQSPPEPAPDRVRLAGCPLAGTPDVGLTAQDATTALGVPTRRRASSG

GIPVATSMWRDAQTVRTYGPAVAKALALRVAGKARSRLTGRHCRKFMQLT

DFDPFDPAIAADPYPHYRELLAGERVQYNPKRDVYILSRYADVREAARNH

DTLSSARGVTFSRGWLPFLPTSDPPAHTRMRKQLAPGMARGALETWRPMV

DQLARELVGGLLTQTPADVVSTVAAPMPMRAITSVLGVDGPDEAAFCRLS

NQAVRITDVALSASGLISLVQGFAGFRRLRALFTHRRDNGLLRECTVLGK

LATHAEQGRLSDDELFFFAVLLLVAGYESTAHMISTLFLTLADYPDQLTL

LAQQPDLIPSAIEEHLRFISPIQNICRTTRVDYSVGQAVIPAGSLVLLAW

GAANRDPRQYEDPDVFRADRNPVGHLAFGSGIHLCPGTQLARMEGQAILR

EIVANIDRIEVVEPPTWTTNANLRGLTRLRVAVTPRVAP

>CYP128A1 (2621858803) Mycobacterium tuberculosis TKK_05MA_0046

MTATQSPPEPAPDRVRLAGCPLAGTPDVGLTAQDATTALGVPTRRRASSG

GIPVATSMWRDAQTVRTYGPAVAKALALRVAGKARSRLTGRHCRKFMQLT

DFDPFDPAIAADPYLHYRELLAGERVQYNPKRDVYILSRYADVREAARNH

DTLSSARGVTFSRGWLPFLPTSDPPAHTRMRKQLAPGMARGALETWRPMV

DQLARELVGGLLTQTPADVVSTVAAPMPMRAITSVLGVDGPDEAAFCRLS

NQAVRITDVALSASGLISLVQGFAGFRRLRALFTHRRDNGLLRECTVLGK

LATHAEQGRLSDDELFFFAVLLLVAGYESPAHMISTLFLTLADYPDQLTL

LAQQPDLIPSAIEEHLRFISPIQNICRTTRVDYSVGQAVIPAGSLVLLAW

GAANRDPRQYEDPDVFRADRNPVGHLAFGSGIHLCPGTQLARMEGQAILR

EIVANIDRIEVVEPPTWTTNANLRGLTRLRVAVTPRVAP

>CYP128A1 (2589604919) Mycobacterium tuberculosis TKK-01-0050

MTATQSPPEPAPDRVRLAGCPLAGTPDVGLTAQDATTALGVPTRRRASSG

GIPVATSMWRDAQTVRTYGPAVAKALALRVAGKARSRLTGRHCRKFMQLT

DFDPFDPAIAADPYPHYRELLAGERVQYNPKRDVYILSRYADVREAARNH

DTLSSARGVTFSRGWLPFLPTSDPPAHTRMRKQLAPGMARGALETWRPMV

DQLARELVGGLLTQTPADVVSTVAAPMPMRAITSVLGVDGPDEAAFCRLS

NQAVRITDVALSASGLISLVQGFAGFRRLRALFTHRRDNGLLRECTVLGK

LATHAEQGRLSDDELFFFAVLLLVAGYESTAHMISTLFLTLADYPDQLTL

LAQQPDLIPSAIEEHLRFISPIQNICRTTRVDYSVGQAVIPAGSLVLLAW

GAANRDPRQYEDPDVFRADRNPVGHLAFGSGIHLCPGPSWRAWRVRRSCA

RSSPISTE

>CYP128A1 (2606435017) Mycobacterium tuberculosis XTB13-119

MTATQSPPEPAPDRVRLAGCPLAGTPDVGLTAQDATTALGVPTRRRASSG

GIPVATSMWRDAQTVRTYGPAVAKALALRVAGKARSRLTGRHCRKFMQLT

DFDPFDPAIAADPYPHYRELLAGERVQYNPKRDVYILSRYADVREAARNH

DTLSSARGVTFSRGWLPFLPTSDPPAHTRMRKQLAPGMARGALETWRPMV

DQLARELVGGLLTQTPADVVSTVAAPMPMRAITSVLGVDGPDEAAFCRLS

NQAVRITDVALSASGLISLVQGFAGFRRLRALFTHRRDNGLLRECTVLGK

LATHAEQGRLSDDELFFFAVLLLVAGYESTAHMISTLFLTLADYPDQLTL

LAQQPDLIPSAIEEHLRFISPIQNICRTTRVDYSVGQAVIPAGSLVLLAW

GAANRDPRQYEDPDVFRADRNPVGHLAFGSGIHLCPGPSWRAWRVRRSCA

RSSPISTE

>CYP128A1 (2575059778) Mycobacterium tuberculosis XTB13-209

MTATQSPPEPAPDRVRLAGCPLAGTPDVGLTAQDATTALGVPTRRRASSG

GIPVATSMWRDAQTVRTYGPAVAKALALRVAGKARSRLTGRHCRKFMQLT

DFDPFDPAIAADPYPHYRELLAGERVQYNPKRDVYILSRYADVREAARNH

DTLSSARGVTFSRGWLPFLPTSDPPAHTRMRKQLAPGMARGALETWRPMV

DQLARELVGGLLTQTPADVVSTVAAPMPMRAITSVLGVDGPDEAAFCRLS

NQAVRITDVALSASGLISLVQGFAGFRRLRALFTHRRDNGLLRECTVLGK

LATHAEQGRLSDDELFFFAVLLLVAGYESTAHMISTLFLTLADYPDQLTL

LAQQPDLIPSAIEEHLRFISPIQNICRTTRVDYSVGQAVIPAGSLVLLAW

GAANRDPRQYEDPDVFRADRNPVGHLAFGSGIHLCPGTQLARMEGQAILR

EIVANIDRIEVVEPPTWTTNANLRGLTRLRVAVTPRVAP

>CYP128A1 (2576197130) Mycobacterium tuberculosis BTB13-063

MTATQSPPEPAPDRVRLAGCPLAGTPDVGLTAQDATTALGVPTRRRASSG

GIPVATSMWRDAQTVRTYGPAVAKALALRVAGKARSRLTGRHCRKFMQLT

DFDPFDPAIAADPYPHYRELLAGERVQYNPKRDVYILSRYADVREAARNH

DTLSSARGVTFSRGWLPFLPTSDPPAHTRMRKQLAPGMARGALETWRPMV

DQLARELVGGLLTQTPADVVSTVAAPMPMRAITSVLGVDGPDEAAFCRLS

NQAVRITDVALSASGLISLVQGFAGFRRLRALFTHRRDNGLLRECTVLGK

LATHAEQGRLSDDELFFFAVLLLVAGYESTAHMISTLFLTLADYPDQLTL

LAQQPDLIPSAIEEHLRFISPIQNICRTTRVDYSVGQAVIPAGSLVLLAW

GAANRDPRQYEDPDVFRADRNPVGHLAFGSGIHLCPGTQLARMEGQAILR

EIVANIDRIEVVEPPTWTTNANLRGLTRLRVAVTPRVAP

>CYP128A1 (2576981642) Mycobacterium tuberculosis TB_RSA149

MTATQSPPEPAPDRVRLAGCPLAGTPDVGLTAQDATTALGVPTRRRASSG

GIPVATSMWRDAQTVRTYGPAVAKALALRVAGKARSRLTGRHCRKFMQLT

DFDPFDPAIAADPYPHYRELLAGERVQYNPKRDVYILSRYADVREAARNH

DTLSSARGVTFSRGWLPFLPTSDPPAHTRMRKQLAPGMARGALETWRPMV

DQLARELVGGLLTQTPADVVSTVAAPMPMRAITSVLGVDGPDEAAFCRLS

NQAVRITDVALSASGLISLVQGFAGFRRLRALFTHRRDNGLLRECTVLGK

LATHAEQGRLSDDELFFFAVLLLVAGYESTAHMISTLFLTLADYPDQLTL

LAQQPDLIPSAIEEHLRFISPIQNICRTTRVDYSVGQAVIPAGSLVLLAW

GAANRDPRQYEDPDVFRADRNPVGHLAFGSGIHLCPGTQLARMEGQAILR

EIVANIDRIEVVEPPTWTTNANLRGLTRLRVAVTPRVAP

>CYP128A1 (2584711873) Mycobacterium tuberculosis TB_RSA01

MTATQSPPEPAPDRVRLAGCPLAGTPDVGLTAQDATTALGVPTRRRASSG

GIPVATSMWRDAQTVRTYGPAVAKALALRVAGKARSRLTGRHCRKFMQLT

DFDPFDPAIAADPYPHYRELLAGERVQYNPKRDVYILSRYADVREAARNH

DTLSSARGVTFSRGWLPFLPTSDPPAHTRMRKQLAPGMARGALETWRPMV

DQLARELVGGLLTQTPADVVSTVAAPMPMRAITSVLGVDGPDEAAFCRLS

NQAVRITDVALSASGLISLVQGFAGFRRLRALFTHRRDNGLLRECTVLGK

LATHAEQGRLSDDELFFFAVLLLVAGYESTAHMISTLFLTLADYPDQLTL

LAQQPDLIPSAIEEHLRFISPIQNICRTTRVDYSVGQAVIPAGSLVLLAW

GAANRDPRQYEDPDVFRADRNPVGHLAFGSGIHLCPGTQLARMEGQAILR

EIVANIDRIEVVEPPTWTTNANLRGLTRLRVAVTPRVAP

>CYP128A1 (2647879050) Mycobacterium bovis MbURU-001

MTATQSPPEPAPDRVRLAGCPLAGTPDVGLTAQDATTALGVPTRRRASSG

GIPVATSMWRDAQTVRTYGPAVAKALALRVAGKARSRLTGRHCRKFMQLT

DFDPFDPAIAADPYPHYRELLAGERVQYNPKRDVYILSRYADVREAARNH

DTLSSARGVTFSRGWLPFLPTSDPPAHTRMRKQLAPGMARGALETWRPMV

DQLARELVGGLLTQTPADVVSTVAAPMPMRAITSVLGVDGPDEAAFCRLS

NQAVRITDVALSASGLISLVQGFAGFRRLRALFTHRRDNGLLRECTVLGK

LATHAEQGRLSDDELFFFAVLLLVAGYESTAHMISTLFLTLADYPDQLTL

LAQQPDLIPSAIEEHLRFISPIQNICRTTRVDYSVGQAVIPAGSLVLLAW

GAANRDPRQYEDPDVFRADRNPVGHLAFGSGIHLCPGTQLARMEGQAILR

EIVANIDRIEVVEPPTWTTNANLRGLTRLRVAVTPRVAP

>CYP128A1 (2666672977) Mycobacterium tuberculosis 1300039

MTATQSPPEPAPDRVRLAGCPLAGTPDVGLTAQDATTALGVPTRRRASSG

GIPVATSMWRDAQTVRTYGPAVAKALALRVAGKARSRLTGRHCRKFMQLT

DFDPFDPAIAADPYPHYRELLAGERVQYNPKRDVYILSRYADVREAARNH

DTLSSARGVTFSRGWLPFLPTSDPPAHTRMRKQLAPGMARGALETWRPMV

DQLARELVGGLLTQTPADVVSTVAAPMPMRAITSVLGVDGPDEAAFCRLS

NQAVRITDVALSASGLISLVQGFAGFRRLRALFTHRRDNGLLRECTVLGK

LATHAEQGRLSDDELFFFAVLLLVAGYESTAHMISTLFLTLADYPDQLTL

LAQQPDLIPSAIEEHLRFISPIQNICRTTRVDYSVGQAVIPAGSLVLLAW

GAANRDPRQYEDPDVFRADRNPVGHLAFGSGIHLCPGTQLARMEGQAILR

EIVANIDRIEVVEPPTWTTNANLRGLTRLRVAVTPRVAP

>CYP128A1 (2584816049) Mycobacterium tuberculosis XTB13-113

MTATQSPPEPAPDRVRLAGCPLAGTPDVGLTAQDATTALGVPTRRRAASG

GIPVATSMWRDAQTVRTYGPAVAKALALRVAGKARSRLTGRHCRKFMQLT

DFDPFDPAIAADPYPHYRELLAGERVQYNPKRDVYILSRYADVREAARNH

DTLSSARGVTFSRGWLPFLPTSDPPAHTRMRKQLAPGMARGALETWRPMV

DQLARELVGGLLTQTPADVVSTVAAPMPMRAITSVLGVDGPDEAAFCRLS

NQAVRITDVALSASGLISLVQGFAGFRRLRALFTHRRDNGLLRECTVLGK

LATHAEQGRLSDDELFFFAVLLLVAGYESTAHMISTLFLTLADYPDQLTL

LAQQPDLIPSAIEEHLRFISPIQNICRTTRVDYSVGQAVIPAGSLVLLAW

GAANRDPRQYEDPDVFRADRNPVGHLAFGSGIHLCPGTQLARMEGQAILR

EIVANIDRIEVVEPPTWTTNANLRGLTRLRVAVTPRVAP

>CYP128B2 (2725096863) Mycobacterium avium hominissuis MAH-P-9060-06

LTLSQDVRFLGTMAQPLSHAARVNTARAFRSRWRQYRFWTGAQITDYDPI

DPANIAQPDAAYRALHAGGRVHYNPKLGLWILTRLPDVRAGARAAETLSS

ADGVTRLRMAGPLLVTMDGKPHNEMRRHVLPAFTKAALESWQAMIDELAA

KLVGEVLDNPGCDVVQRLAIPMPMLLIAHMLGIPDGDIDDFRRWSSDAVK

MADVDISRRGLTRLKSSIGGIRDIYRYFRQQFAVGGLKGSDTLLGKLLSV

NASGSIDDNELFFFAMLLLIAGNETTTNLLGGMFDIFARHPEKFDMVRAD

HSLIPKVVEEQLRFSSPVQMLYRTARTNYEVGPVTIPAGARVLLSLGAAN

RDPQVFDEPDEFRVDRNPTEHLAFGFGAHLCLGAQLTRMEAQAVLRELVT

RARRIEAIGETQWSTGYLLRGPERMNVRLTPSVVAG

>CYP128A1 (2546206759) Mycobacterium tuberculosis EAI5/NITR206

MTATQSPPEPAPDRVRLAGCPLAGTPDVGLTAQDATTALGVPTRRRASSG

GIPVATSMWRDAQTVRTYGPAVAKALALRVAGKARSRLTGRHCRKFMQLT

DFDPFDPAIAADPYPHYRELLAGERVQYNPKRDVYILSRYADVREAARNH

DTLSSARGVTFSRGWLPFLPTSDPPAHTRMRKQLAPGMARGALETWRPMV

DQLARELVGGLLTQTPADVVSTVAAPMPMRAITSVLGVDGPDEAAFCRLS

NQAVRITDVALSASGLISLVQGFAGFRRLRALFTHRRDNGLLRECTVLGK

LATHAEQGRLSDDELFFFAVLLLVAGYESTAHMISTLFLTLADYPDQLTL

LAQQPDLIPSAIEEHLRFISPIQNICRTTRVDYSVGQAVIPAGSLVLLAW

GAANRDPRQYEDPDVFRADRNPVGHLAFGSGIHLCPGTQLARMEGQAILR

EIVANIDRIEVVEPPTWTTNANLRGLTRLRVAVTPRVAP

>CYP128A1 (2576948334) Mycobacterium tuberculosis BTB03-144

MTATQSPPEPAPDRVRLAGCPLAGTPDVGLTAQDATTALGVPTRRRASSG

GIPVATSMWRDAQTVRTYGPAVAKALALRVAGKARSRLTGRHCRKFMQLT

DFDPFDPAIAADPYPHYRELLAGERVQYNPKRDVYILSRYADVREAARNH

DTLSSARGVTFSRGWLPFLPTSDPPAHTRMRKQLAPGMARGALETWRPMV

DQLARELVGGLLTQTPADVVSTVAAPMPMRAITSVLGVDGPDEAAFCRLS

NQAVRITDVALSASGLISLVQGFAGFRRLRALFTHRRDNGLLRECTVLGK

LATHAEQGRLSDDELFFFAVLLLVAGYESTAHMISTLFLTLADYPDQLTL

LAQQPDLIPSAIEEHLRFISPIQNICRTTRVDYSVGQAVIPAGSLVLLAW

GAANRDPRQYEDPDVFRADRNPVGHLAFGSGIHLCPGTQLARMEGQAILR

EIVANIDRIEVVEPPTWTTNANLRGLTRLRVAVTPRVAP

>CYP128A1 (2577893844) Mycobacterium tuberculosis GM 1503

MTATQSPPEPAPDRVRLAGCPLAGTPDVGLTAQDATTALGVPTRRRASSG

GIPVATSMWRDAQTVRTYGPAVAKALALRVAGKARSRLTGRHCRKFMQLT

DFDPFDPAIAADPYPHYRELLAGERVQYNPKRDVYILSRYADVREAARNH

DTLSSARGVTFSRGWLPFLPTSDPPAHTRMRKQLAPGMARGALETWRPMV

DQLARELVGGLLTQTPADVVSTVAAPMPMRAITSVLGVDGPDEAAFCRLS

NQAVRITDVALSASGLISLVQGFAGFRRLRALFTHRRDNGLLRECTVLGK

LATHAEQGRLSDDELFFFAVLLLVAGYESTAHMISTLFLTLADYPDQLTL

LAQQPDLIPSAIEEHLRFISPIQNICRTTRVDYSVGQAVIPAGSLVLLAW

GAANRDPRQYEDPDVFRADRNPVGHLAFGSGIHLCPGTQLARMEGQAILR

EIVANIDRIEVVEPPTWTTNANLRGLTRLRVAVTPRVAP

>CYP128A1 (2603980648) Mycobacterium tuberculosis TB_RSA180

MTATQSPPEPAPDRVRLAGCPLAGTPDVGLTAQDATTALGVPTRRRASSG

GIPVATSMWRDAQTVRTYGPAVAKALALRVAGKARSRLTGRHCRKFMQLT

DFDPFDPAIAADPYPHYRELLAGERVQYNPKRDVYILSRYADVREAARNH

DTLSSARGVTFSRGWLPFLPTSDPPAHTRMRKQLAPGMARGALETWRPMV

DQLARELVGGLLTQTPADVVSTVAAPMPMRAITSVLGVDGPDEAAFCRLS

NQAVRITDVALSASGLISLVQGFAGFRRLRALFTHRRDNGLLRECTVLGK

LATHAEQGRLSDDELFFFAVLLLVAGYESTAHMISTLFLTLADYPDQLTL

LAQQPDLIPSAIEEHLRFISPIQNICRTTRVDYSVGQAVIPAGSLVLLAW

GAANRDPRQYEDPDVFRADRNPVGHLAFGSGIHLCPGTQLARMEGQAILR

EIVANIDRIEVVEPPTWTTNANLRGLTRLRVAVTPRVAP

>CYP128A1 (2604582211) Mycobacterium tuberculosis BTB10-253

MTATQSPPEPAPDRVRLAGCPLAGTPDVGLTAQDATTALGVPTRRRASSG

GIPVATSMWRDAQTVRTYGPAVAKALALRVAGKARSRLTGRHCRKFMQLT

DFDPFDPAIAADPYPHYRELLAGERVQYNPKRDVYILSRYADVREAARNH

DTLSSARGVTFSRGWLPFLPTSDPPAHTRMRKQLAPGMARGALETWRPMV

DQLARELVGGLLTQTPADVVSTVAAPMPMRAITSVLGVDGPDEAAFCRLS

NQAVRITDVALSASGLISLVQGFAGFRRLRALFTHRRDNGLLRECTVLGK

LATHAEQGRLSDDELFFFAVLLLVAGYESTAHMISTLFLTLADYPDQLTL

LAQQPDLIPSAIEEHLRFISPIQNICRTTRVDYSVGQAVIPAGSLVLLAW

GAANRDPRQYEDPDVFRADRNPVGHLAFGSGIHLCPGTQLARMEGQAILR

EIVANIDRIEVVEPPTWTTNANLRGLTRLRVAVTPRVAP

>CYP128A1 (2621580415) Mycobacterium tuberculosis XTB13-212

MTATQSPPEPAPDRVRLAGCPLAGTPDVGLTAQDATTALGVPTRRRASSG

GIPVATSMWRDAQTVRTYGPAVAKALALRVAGKARSRLTGRHCRKFMQLT

DFDPFDPAIAADPYPHYRELLAGERVQYNPKRDVYILSRYADVREAARNH

DTLSSARGVTFSRGWLPFLPTSDPPAHTRMRKQLAPGMARGALETWRPMV

DQLARELVGGLLTQTPADVVSTVAAPMPMRAITSVLGVDGPDEAAFCRLS

NQAVRITDVALSASGLISLVQGFAGFRRLRALFTHRRDNGLLRECTVLGK

LATHAEQGRLSDDELFFFAVLLLVAGYESTAHMISTLFLTLADYPDQLTL

LAQQPDLIPSAIEEHLRFISPIQNICRTTRVDYSVGQAVIPAGSLVLLAW

GAANRDPRQYEDPDVFRADRNPVGHLAFGSGIHLCPGTQLARMEGQAILR

EIVANIDRIEVVEPPTWTTNANLRGLTRLRVAVTPRVAP

>CYP128A1 (2622393005) Mycobacterium tuberculosis M1236

MTATQSPPEPAPDRVRLAGCPLAGTPDVGLTAQDATTALGVPTRRRASSG

GIPVATSMWRDAQTVRTYGPAVAKALALRVAGKARSRLTGRHCRKFMQLT

DFDPFDPAIAADPYPHYRELLAGERVQYNPKRDVYILSRYADVREAARNH

DTLSSARGVTFSRGWLPFLPTSDPPAHTRMRKQLAPGMARGALETWRPMV

DQLARELVGGLLTQTPADVVSTVAAPMPMRAITSVLGVDGPDEAAFCRLS

NQAVRITDVALSASGLISLVQGFAGFRRLRALFTHRRDNGLLRECTVLGK

LATHAEQGRLSDDELFFFAVLLLVAGYESTAHMISTLFLTLADYPDQLTL

LAQQPDLIPSAIEEHLRFISPIQNICRTTRVDYSVGQAVIPAGSLVLLAW

GAANRDPRQYEDPDVFRADRNPVGHLAFGSGIHLCPGTQLARMEGQAILR

EIVANIDRIEVVEPPTWTTNANLRGLTRLRVAVTPRVAP

>CYP128A1 (2670438982) Mycobacterium tuberculosis A70088

MTATQSPPEPAPDRVRLAGCPLAGTPDVGLTAQDATTALGVPTRRRASSG

GIPVATSMWRDAQTVRTYGPAVAKALALRVAGKARSRLTGRHCRKFMQLT

DFDPFDPAIAADPYPHYRELLAGERVQYNPKRDVYILSRYADVREAARNH

DTLSSARGVTFSRGWLPFLPTSDPPAHTRMRKQLAPGMARGALETWRPMV

DQLARELVGGLLTQTPADVVSTVAAPMPMRAITSVLGVDGPDEAAFCRLS

NQAVRITDVALSASGLISLVQGFAGFRRLRALFTHRRDNGLLRECTVLGK

LATHAEQGRLSDDELFFFAVLLLVAGYESTAHMISTLFLTLADYPDQLTL

LAQQPDLIPSAIEEHLRFISPIQNICRTTRVDYSVGQAVIPAGSLVLLAW

GAANRDPRQYEDPDVFRADRNPVGHLAFGSGIHLCPGTQLARMEGQAILR

EIVANIDRIEVVEPPTWTTNANLRGLTRLRVAVTPRVAP

>CYP128A1 (2703074307) Mycobacterium tuberculosis MTBR3/09

MTATQSPPEPAPDRVRLAGCPLAGTPDVGLTAQDATTALGVPTRRRASSG

GIPVATSMWRDAQTVRTYGPAVAKALALRVAGKARSRLTGRHCRKFMQLT

DFDPFDPAIAADPYPHYRELLAGERVQYNPKRDVYILSRYADVREAARNH

DTLSSARGVTFSRGWLPFLPTSDPPAHTRMRKQLAPGMARGALETWRPMV

DQLARELVGGLLTQTPADVVSTVAAPMPMRAITSVLGVDGPDEAAFCRLS

NQAVRITDVALSASGLISLVQGFAGFRRLRALFTHRRDNGLLRECTVLGK

LATHAEQGRLSDDELFFFAVLLLVAGYESTAHMISTLFLTLADYPDQLTL

LAQQPDLIPSAIEEHLRFISPIQNICRTTRVDYSVGQAVIPAGSLVLLAW

GAANRDPRQYEDPDVFRADRNPVGHLAFGSGIHLCPGTQLARMEGQAILR

EIVANIDRIEVVEPPTWTTNANLRGLTRLRVAVTPRVAP

>CYP128A1 (2641478967) Mycobacterium tuberculosis TKK_03_0100

MTATQSPPEPAPDRVRLAGCPLAGTPDVGLTAQDATTALGVPTRRRASSG

GIPVATSMWRDAQTVRTYGPAVAKALALRVAGKARSRLTGRHCRKFMQLT

DFDPFDPAIAADPYPHYRELLAGERVQYNPKRDVYILSRYADVREAARNH

DTLSSARGVTFSRGWLPFLPTSDPPAHTRMRKQLAPGMARGALETWRPMV

DQLARELVGGLLTQTPADVVSTVAAPMPMRAITSVLGVDGPDEAAFCRLS

NQAVRITDVALSASGLISLVQGFAGFRRLRALFTHRRDNGLLRECTVLGK

LATHAEQGRLSDDELFFFAVLLLVAGYESPAHMISTLFLTLADYPDQLTL

LAQQPDLIPSAIEEHLRFISPIQNICRTTRVDYSVGQAVIPAGSLVLLAW

GAANRDPRQYEDPDVFRADRNPVGHLAFGSGIHLCPGTQLARMEGQAILR

EIVANIDRIEVVEPPTWTTNANLRGLTRLRVAVTPRVAP

>CYP128A1 (2702063418) Mycobacterium tuberculosis A70387

MTATQSPPEPAPDRVRLAGCPLAGTPDVGLTAQDATTALGVPTRRRASSG

GIPVATSMWRDAQTVRTYGPAVAKALALRVAGKARSRLTGRHCRKFMQLT

DFDPFDPAIAADPYPHYRELLAGERVQDNPKRDVYILSRYADVREAARNH

DTLSSARGVTFSRGWLPFLPTSDPPAHTRMRKQLAPGMARGALETWRPMV

DQLARELVGGLLTQTPADVVSTVAAPMPMRAITSVLGVDGPDEAAFCRLS

NQAVRITDVALSASGLISLVQGFAGFRRLRALFTHRRDNGLLRECTVLGK

LATHAEQGRLSDDELFFFAVLLLVAGYESTAHMISTLFLTLADYPDQLTL

LAQQPDLIPSAIEEHLRFISPIQNICRTTRVDYSVGQAVIPAGSLVLLAW

GAANRDPRQYEDPDVFRADRNPVGHLAFGSGIHLCPGTQLARMEGQAILR

EIVANIDRIEVVEPPTWTTNANLRGLTRLRVAVTPRVAP

>CYP128A1 (2603138015) Mycobacterium tuberculosis OSDD105

MTATQSPPEPAPDRVRLAGCPLAGTPDVGLTAQDATTALGVPTRRRASSG

GIPVATSMWRDAQTVRTYGPAVAKALALRVAGKARSRLTGRHCRKFMQLT

DFDPFDPAIAADPYPHYRELLAGERVQYNPKRDVYILSRYADVREAARNH

DTLSSARGVTFSRGWLPFLPTSDPPAHTRMRKQLAPGMARGALETWRPMV

DQLARELVGGLLTQTPADVVSTVAAPMPMRAITSVLGVDGPDEAAFCRLS

NQAVRITDVALSASGLISLVQGFAGFRRLRALFTHRRDNGLLRECTVLGK

LATHAEQGRLSDDELFFFAVLLLVAGYESTAHMISTLFLTLADYPDQLTL

LAQQPDLIPSAIEEHLRFISPIQNICRTTRVDYSVGQAVIPAGSLVLLAW

GAANRDPRQYEDPDVFRADRNPVGHLAFGSGIHLCPGPSWRAWRVRRSCA

RSSPISTE

>CYP128B4 (2802871432) Mycobacteroides abscessus abscessus 1148

MSRWRSDSAVRQIGAPVVAAVGMNIAAAVRVRRRGYAGWTGAVNTDYDPL

DPATAAQPFDAYRALHAGGRVHYNPKRATFILSRHEDIRAALRDTDAVTS

SQGVTRMKISAPILVLTDGDDHTRLRKQVQPGFTRGAMSDWQGMADQLAK

ELVADVVANPGCDVMERLAVPLPIRMIAHIIGIPPEDVQNFRSWSEDGVG

VINAGVSPAGLRQGLKGVRAIAALRRYFKDQLASGKLKGSDTVLGRLVDN

NEDGKLSDDELFFIAMLLLFAGNETTTNLIGGMFDTLAHAPDQFAMIRDD

PDLIPSAVEEQLRYSAPIQNLYRYTRTDYRVGEVTIPSGSRLLLAFGAAN

RDPEVFEDPDTYRADRNPRNHIAFGYGVHMCIGATLSRMEGQAVLRELTS

QASAIAAAGSATWSTNSSLRGTTYLPIRLTPAR

>CYP128A1 (2511736677) Mycobacterium tuberculosis CTRI-2

MTATQSPPEPAPDRVRLAGCPLAGTPDVGLTAQDATTALGVPTRRRASSG

GIPVATSMWRDAQTVRTYGPAVAKALALRVAGKARSRLTGRHCRKFMQLT

DFDPFDPAIAADPYPHYRELLAGERVQYNPKRDVYILSRYADVREAARNH

DTLSSARGVTFSRGWLPFLPTSDPPAHTRMRKQLAPGMARGALETWRPMV

DQLARELVGGLLTQTPADVVSTVAAPMPMRAITSVLGVDGPDEAAFCRLS

NQAVRITDVALSASGLISLVQGFAGFRRLRALFTHRRDNGLLRECTVLGK

LATHAEQGRLSDDELFFFAVLLLVAGYESTAHMISTLFLTLADYPDQLTL

LAQQPDLIPSAIEEHLRFISPIQNICRTTRVDYSVGQAVIPAGSLVLLAW

GAANRDPRQYEDPDVFRADRNPVGHLAFGSGIHLCPGTQLARMEGQAILR

EIVANIDRIEVVEPPTWTTNANLRGLTRLRVAVTPRVAP

>CYP128A1 (2584801635) Mycobacterium tuberculosis TKK_02_0002

MTATQSPPEPAPDRVRLAGCPLAGTPDVGLTAQDATTALGVPTRRRASSG

GIPVATSMWRDAQTVRTYGPAVAKALALRVAGKARSRLTGRHCRKFMQLT

DFDPFDPAIAADPYPHYRELLAGERVQYNPKRDVYILSRYADVREAARNH

DTLSSARGVTFSRGWLPFLPTSDPPAHTRMRKQLAPGMARGALETWRPMV

DQLARELVGGLLTQTPADVVSTVAAPMPMRAITSVLGVDGPDEAAFCRLS

NQAVRITDVALSASGLISLVQGFAGFRRLRALFTHRRDNGLLRECTVLGK

LATHAEQGRLSDDELFFFAVLLLVAGYESTAHMISTLFLTLADYPDQLTL

LAQQPDLIPSAIEEHLRFISPIQNICRTTRVDYSVGQAVIPAGSLVLLAW

GAANRDPRQYEDPDVFRADRNPVGHLAFGSGIHLCPGTQLARMEGQAILR

EIVANIDRIEVVEPPTWTTNANLRGLTRLRVAVTPRVAP

>CYP128A1 (2590191527) Mycobacterium tuberculosis MAL020208

MTATQSPPEPAPDRVRLAGCPLAGTPDVGLTAQDATTALGVPTRRRASSG

GIPVATSMWRDAQTVRTYGPAVAKALALRVAGKARSRLTGRHCRKFMQLT

DFDPFDPAIAADPYPHYRELLAGERVQYNPKRDVYILSRYADVREAARNH

DTLSSARGVTFSRGWLPFLPTSDPPAHTRMRKQLAPGMARGALETWRPMV

DQLARELVGGLLTQTPADVVSTVAAPMPMRAITSVLGVDGPDEAAFCRLS

NQAVRITDVALSASGLISLVQGFAGFRRLRALFTHRRDNGLLRECTVLGK

LATHAEQGRLSDDELFFFAVLLLVAGYESTAHMISTLFLTLADYPDQLTL

LAQQPDLIPSAIEEHLRFISPIQNICRTTRVDYSVGQAVIPAGSLVLLAW

GAANRDPRQYEDPDVFRADRNPVGHLAFGSGIHLCPGTQLARMEGQAILR

EIVANIDRIEVVEPPTWTTNANLRGLTRLRVAVTPRVAP

>CYP128A1 (2592267916) Mycobacterium tuberculosis TKK_04_0005

MTATQSPPEPAPDRVRLAGCPLAGTPDVGLTAQDATTALGVPTRRRASSG

GIPVATSMWRDAQTVRTYGPAVAKALALRVAGKARSRLTGRHCRKFMQLT

DFDPFDPAIAADPYPHYRELLAGERVQYNPKRDVYILSRYADVREAARNH

DTLSSSRGVTFSRGWLPFLPTSDPPAHTRMRKQLAPGMARGALETWRPMV

DQLARELVGGLLTQTPADVVSTVAAPMPMRAITSVLGVDGPDEAAFCRLS

NQAVRITDVALSASGLISLVQGFAGFRRLRALFTHRRDNGLLRECTVLGK

LATHAEQGRLSDDELFFFAVLLLVAGYESTAHMISTLFLTLADYPDQLTL

LAQQPDLIPSAIEEHLRFISPIQNICRTTRVDYSVGQAVIPAGSLVLLAW

GAANRDPRQYEDPDVFRADRNPVGHLAFGSGIHLCPGTQLARMEGQAILR

EIVANIDRIEVVEPPTWTTNANLRGLTRLRVAVTPRVAP

>CYP128A1 (2592578379) Mycobacterium tuberculosis TKK_04_0029

MTATQSPPEPAPDRVRLAGCPLAGTPDVGLTAQDATTALGVPTRRRASSG

GIPVATSMWRDAQTVRTYGPAVAKALALRVAGKARSRLTGRHCRKFMQLT

DFDPFDPAIAADPYPHYRELLAGERVQYNPKRDVYILSRYADVREAARNH

DTLSSARGVTFSRGWLPFLPTSDPPAHTRMRKQLAPGMARGALETWRPMV

DQLARELVGGLLTQTPADVVSTVAAPMPMRAITSVLGVDGPDEAAFCRLS

NQAVRITDVALSASGLISLVQGFAGFRRLRALFTHRRDNGLLRECTVLGK

LATHAEQGRLSDDELFFFAVLLLVAGYESTAHMISTLFLTLADYPDQLTL

LAQQPDLIPSAIEEHLRFISPIQNICRTTRVDYSVGQAVIPAGSLVLLAW

GAANRDPRQYEDPDVFRADRNPVGHLAFGSGIHLCPGTQLARMEGQAILR

EIVANIDRIEVVEPPTWTTNANLRGLTRLRVAVTPRVAP

>CYP128A1 (2696526784) Mycobacterium tuberculosis R09401839

MTATQSPPEPAPDRVRLAGCPLAGTPDVGLTAQDATTALGVPTRRRASSG

GIPVATSMWRDAQTVRTYGPAVAKALALRVAGKARSRLTGRHCRKFMQLT

DFDPFDPAIAADPYPHYRELLAGERVQYNPKRDVYILSRYADVREAARNH

DTLSSARGVTFSRGWLPFLPTSDPPAHTRMRKQLAPGMARGALETWRPMV

DQLARELVGGLLTQTPADVVSTVAAPMPMRAITSVLGVDGPDEAAFCRLS

NQAVRITDVALSASGLISLVQGFAGFRRLRALFTHRRDNGLLRECTVLGK

LATHAEQGRLSDDELFFFAVLLLVAGYESTAHMISTLFLTLADYPDQLTL

LAQQPDLIPSAIEEHLRFISPIQNICRTTRVDYSVGQAVIPAGSLVLLAW

GAANRDPRQYEDPDVFRADRNPVGHLAFGSGIHLCPGTQLARMEGQAILR

EIVANIDRIEVVEPPTWTTNANLRGLTRLRVAVTPRVAP

>CYP128A1 (2674706798) Mycobacterium tuberculosis W09900434

MTATQSPPEPAPDRVRLAGCPLAGTPDVGLTAQDATTALGVPTRRRASSG

GIPVATSMWRDAQTVRTYGPAVAKALALRVAGKARSRLTGRHCRKFMQLT

DFDPFDPAIAADPYPHYRELLAGERVQYNPKRDVYILSRYADVREAARNH

DTLSSARGVTFSRGWLPFLPTSDPPAHTRMRKQLAPGMARGALETWRPMV

DQLARELVGGLLTQTPADVVSTVAAPMPMRAITSVLGVDGPDEAAFCRLS

NQAVRITDVALSASGLISLVQGFAGFRRLRALFTHRRDNGLLRECTVLGK

LATHAEQGRLSDDELFFFAVLLLVAGYESTAHMISTLFLTLADYPDQLTL

LAQQPDLIPSAIEEHLRFISPIQNICRTTRVDYSVGQAVIPAGSLVLLAW

GAANRDPRQYEDPDVFRADRNPVGHLAFGSGIHLCPGPSWRAWRVRRSCA

RSSPISTE

>CYP128B4 (2802815619) Mycobacteroides abscessus abscessus 1085

MSRWRSDSAVRQIGAPVVAAVGMNIAAAVRVRRRGYAGWTGAVNTDYDPL

DPATAAQPFDAYRALHAGGRVHYNPKRATFILSRHEDIRAALRDTDAVTS

SQGVTRMKISAPILVLTDGDDHTRLRKQVQPGFTRGAMSDWQGMADQLAK

ELVADVVANPGCDVMERLAVPLPIRMIAHIIGIPPEDVQNFRSWSEDGVG

VINAGVSPAGLRQGLKGVRAIAALRRYFKDQLASGKLKGSDTVLGRLVDN

NEDGKLSDDELFFIAMLLLFAGNETTTNLIGGMFDTLAHAPDQFAMIRDD

PDLIPSAVEEQLRYSAPIQNLYRYTRTDYRVGEVTIPSGSRLLLAFGAAN

RDPEVFEDPDTYRADRNPRNHIAFGYGVHMCIGATLSRMEGQAVLRELTS

QASAIAAAGSATWSTNSSLRGTTYLPIRLTPAR

>CYP128B1 (2809688439) Mycobacterium sp. E735

MAAKQTIQGAAEGARLLGHAVRMNVAAAVRTRRRGRAGWTGAVNTDYDPQ

DPATAADPYDAYRALHRSGRVHYNPRRATFIVSRLDDVRAALRDTDRVTS

TQGVTRLRMSAPLAVLTDGEEHTRLRKQVQPGFSKGAMRSWQEMTEKLAI

DLVTDVMDDPGCDVVQRLAIPMPIRLIAQILGVPDSDAGDFRRWSERAVG

VMELRPTFAGIVDAAKSATGMVALWRYFTAQFAAGGLKGSTTVLGRLIEH

NTDGSLTDRQLLLIAIHLLIAGNETTTNLLGGMFDTLARRPDQYDLIRAN

PDLIPLAVEEQLRFTTPIQNLYRYTRADYRIGDVTIPTGSRVLLSFGAAN

RDPSAFDDPDEYRADRDPRTHVAFGYGAHMCLGAPLARMEAQAVLRQLVS

RVARITPAGPTQWSRHSSLRGPTRLPIRLTPA

>CYP128B4 (2797193916) Mycobacteroides abscessus abscessus 498

MSRWRSDSAVRQIGAPVVAAVGMNIAAAVRVRRRGYAGWTGAVNTDYDPL

DPATAAQPFDAYRALHAGGRVHYNPKRATFILSRHEDIRAALRDTDAVTS

SQGVTRMKISAPILVLTDGDDHTRLRKQVQPGFTRGAMSDWQGMADQLAK

ELVADVVANPGCDVMERLAVPLPIRMIAHIIGIPPEDVQNFRSWSEDGVG

VINAGVSPAGLRQGLKGVRAIAALRRYFKDQLASGKLKGSDTVLGRLVDN

NEDGKLSDDELFFIAMLLLFAGNETTTNLIGGMFDTLAHAPDQFAMIRDD

PDLIPSAVEEQLRYSAPIQNLYRYTRTDYRVGEVTIPSGSRLLLAFGAAN

RDPEVFEDPDTYRADRNPRNHIAFGYGVHMCIGATLSRMEGQAVLRELTS

QASAIAAAGSATWSTNSSLRGTTYLPIRLTPAR

>CYP128A1 (2555430755) Mycobacterium tuberculosis PanR0909

MTATQSPPEPAPDRVRLAGCPLAGTPDVGLTAQDATTALGVPTRRRASSG

GIPVATSMWRDAQTVRTYGPAVAKALALRVAGKARSRLTGRHCRKFMQLT

DFDPFDPAIAADPYPHYRELLAGERVQYNPKRDVYILSRYADVREAARNH

DTLSSARGVTFSRGWLPFLPTSDPPAHTRMRKQLAPGMARGALETWRPMV

DQLARELVGGLLTQTPADVVSTVAAPMPMRAITSVLGVDGPDEAAFCRLS

NQAVRITDVALSASGLISLVQGFAGFRRLRALFTHRRDNGLLRECTVLGK

LATHAEQGRLSDDELFFFAVLLLVAGYESTAHMISTLFLTLADYPDQLTL

LAQQPDLIPSAIEEHLRFISPIQNICRTTRVDYSVGQAVIPAGSLVLLAW

GAANRDPRQYEDPDVFRADRNPVGHLAFGSGIHLCPGTQLARMEGQAILR

EIVANIDRIEVVEPPTWTTNANLRGLTRLRVAVTPRVAP

>CYP128A1 (2575139699) Mycobacterium tuberculosis MD17902

MTATQSPPEPAPDRVRLAGCPLAGTPDVGLTAQDATTALGVPTRRRASSG

GIPVATSMWRDAQTVRTYGPAVAKALALRVAGKARSRLTGRHCRKFMQLT

DFDPFDPAIAADPYPHYRELLAGERVQYNPKRDVYILSRYADVREAARNH

DTLSSARGVTFSRGWLPFLPTSDPPAHTRMRKQLAPGMARGALETWRPMV

DQLARELVGGLLTQTPADVVSTVAAPMPMRAITSVLGVDGPDEAAFCRLS

NQAVRITDVALSASGLISLVQGFAGFRRLRALFTHRRDNGLLRECTVLGK

LATHAEQGRLSDDELFFFAVLLLVAGYESTAHMISTLFLTLADYPDQLTL

LAQQPDLIPSAIEEHLRFISPIQNICRTTRVDYSVGQAVIPAGSLVLLAW

GAANRDPRQYEDPDVFRADRNPVGHLAFGSGIHLCPGTQLARMEGQAILR

EIVANIDRIEVVEPPTWTTNANLRGLTRLRVAVTPRVAP

>CYP128A1 (2577879831) Mycobacterium tuberculosis MD18478

MTATQSPPEPAPDRVRLAGCPLAGTPDVGLTAQDATTALGVPTRRRASSG

GIPVATSMWRDAQTVRTYGPAVAKALALRVAGKARSRLTGRHCRKFMQLT

DFDPFDPAIAADPYPHYRELLAGERVQYNPKRDVYILSRYADVREAARNH

DTLSSARGVTFSRGWLPFLPTSDPPAHTRMRKQLAPGMARGALETWRPMV

DQLARELVGGLLTQTPADVVSTVAAPMPMRAITSVLGVDGPDEAAFCRLS

NQAVRITDVALSASGLISLVQGFAGFRRLRALFTHRRDNGLLRECTVLGK

LATHAEQGRLSDDELFFFAVLLLVAGYESTAHMISTLFLTLADYPDQLTL

LAQQPDLIPSAIEEHLRFISPIQNICRTTRVDYSVGQAVIPAGSLVLLAW

GAANRDPRQYEDPDVFRADRNPVGHLAFGSGIHLCPGTQLARMEGQAILR

EIVANIDRIEVVEPPTWTTNANLRGLTRLRVAVTPRVAP

>CYP128A1 (2592374081) Mycobacterium tuberculosis TKK_02_0046

MTATQSPPEPAPDRVRLAGCPLAGTPDVGLTAQDATTALGVPTRRRASSG

GIPVATSMWRDAQTVRTYGPAVAKALALRVAGKARSRLTGRHCRKFMQLT

DFDPFDPAIAADPYPHYRELLAGERVQYNPKRDVYILSRYADVREAARNH

DTLSSARGVTFSRGWLPFLPTSDPPAHTRMRKQLAPGMARGALETWRPMV

DQLARELVGGLLTQTPADVVSTVAAPMPMRAITSVLGVDGPDEAAFCRLS

NQAVRITDVALSASGLISLVQGFAGFRRLRALFTHRRDNGLLRECTVLGK

LATHAEQGRLSDDELFFFAVLLLVAGYESTAHMISTLFLTLADYPDQLTL

LAQQPDLIPSAIEEHLRFISPIQNICRTTRVDYSVGQAVIPAGSLVLLAW

GAANRDPRQYEDPDVFRADRNPVGHLAFGSGIHLCPGTQLARMEGQAILR

EIVANIDRIEVVEPPTWTTNANLRGLTRLRVAVTPRVAP

>CYP128A1 (2603189718) Mycobacterium tuberculosis BTB13-276

MTATQSPPEPAPDRVRLAGCPLAGTPDVGLTAQDATTALGVPTRRRASSG

GIPVATSMWRDAQTVRTYGPAVAKALALRVAGKARSRLTGRHCRKFMQLT

DFDPFDPAIAADPYPHYRELLAGERVQYNPKRDVYILSRYADVREAARNH

DTLSSARGVTFSRGWLPFLPTSDPPAHTRMRKQLAPGMARGALETWRPMV

DQLARELVGGLLTQTPADVVSTVAAPMPMRAITSVLGVDGPDEAAFCRLS

NQAVRITDVALSASGLISLVQGFAGFRRLRALFTHRRDNGLLRECTVLGK

LATHAEQGRLSDDELFFFAVLLLVAGYESTAHMISTLFLTLADYPDQLTL

LAQQPDLIPSAIEEHLRFISPIQNICRTTRVDYSVGQAVIPAGSLVLLAW

GAANRDPRQYEDPDVFRADRNPVGHLAFGSGIHLCPGTQLARMEGQAILR

EIVANIDRIEVVEPPTWTTNANLRGLTRLRVAVTPRVAP

>CYP128A1 (2604242509) Mycobacterium tuberculosis BTB11-299

MTATQSPPEPAPDRVRLAGCPLAGTPDVGLTAQDATTALGVPTRRRASSG

GIPVATSMWRDAQTVRTYGPAVAKALALRVAGKARSRLTGRHCRKFMQLT

DFDPFDPAIAADPYPHYRELLAGERVQYNPKRDVYILSRYADVREAARNH

DTLSSARGVTFSRGWLPFLPTSDPPAHTRMRKQLAPGMARGALETWRPMV

DQLARELVGGLLTQTPADVVSTVAAPMPMRAITSVLGVDGPDEAAFCRLS

NQAVRITDVALSASGLISLVQGFAGFRRLRALFTHRRDNGLLRECTVLGK

LATHAEQGRLSDDELFFFAVLLLVAGYESTAHMISTLFLTLADYPDQLTL

LAQQPDLIPSAIEEHLRFISPIQNICRTTRVDYSVGQAVIPAGSLVLLAW

GAANRDPRQYEDPDVFRADRNPVGHLAFGSGIHLCPGTQLARMEGQAILR

EIVANIDRIEVVEPPTWTTNANLRGLTRLRVAVTPRVAP

>CYP128A1 (2621731842) Mycobacterium tuberculosis 2228BH

MTATQSPPEPAPDRVRLAGCPLAGTPDVGLTAQDATTALGVPTRRRASSG

GIPVATSMWRDAQTVRTYGPAVAKALALRVAGKARSRLTGRHCRKFMQLT

DFDPFDPAIAADPYPHYRELLAGERVQYNPKRDVYILSRYADVREAARNH

DTLSSARGVTFSRGWLPFLPTSDPPAHTRMRKQLAPGMARGALETWRPMV

DQLARELVGGLLTQTPADVVSTVAAPMPMRAITSVLGVDGPDEAAFCRLS

NQAVRITDVALSASGLISLVQGFAGFRRLRALFTHRRDNGLLRECTVLGK

LATHAEQGRLSDDELFFFAVLLLVAGYESTAHMISTLFLTLADYPDQLTL

LAQQPDLIPSAIEEHLRFISPIQNICRTTRVDYSVGQAVIPAGSLVLLAW

GAANRDPRQYEDPDVFRADRNPVGHLAFGSGIHLCPGTQLARMEGQAILR

EIVANIDRIEVVEPPTWTTNANLRGLTRLRVAVTPRVAP

>CYP128A1 (2631398008) Mycobacterium tuberculosis KT-0194

MTATQSPPEPAPDRVRLAGCPLAGTPDVGLTAQDATTALGVPTRRRASSG

GIPVATSMWRDAQTVRTYGPAVAKALALRVAGKARSRLTGRHCRKFMQLT

DFDPFDPAIAADPYPHYRELLAGERVQYNPKRDVYILSRYADVREAARNH

DTLSSARGVTFSRGWLPFLPTSDPPAHTRMRKQLAPGMARGALETWRPMV

DQLARELVGGLLTQTPADVVSTVAAPMPMRAITSVLGVDGPDEAAFCRLS

NQAVRITDVALSASGLISLVQGFAGFRRLRALFTHRRDNGLLRECTVLGK

LATHAEQGRLSDDELFFFAVLLLVAGYESTAHMISTLFLTLADYPDQLTL

LAQQPDLIPSAIEEHLRFISPIQNICRTTRVDYSVGQAVIPAGSLVLLAW

GAANRDPRQYEDPDVFRADRNPVGHLAFGSGIHLCPGTQLARMEGQAILR

EIVANIDRIEVVEPPTWTTNANLRGLTRLRVAVTPRVAP

>CYP128A1 (2631963711) Mycobacterium tuberculosis TKK_04_0071

MTATQSPPEPAPDRVRLAGCPLAGTPDVGLTAQDATTALGVPTRRRASSG

GIPVATSMWRDAQTVRTYGPAVAKALALRVAGKARSRLTGRHCRKFMQLT

DFDPFDPAIAADPYPHYRELLAGERVQYNPKRDVYILSRYADVREAARNH

DTLSSARGVTFSRGWLPFLPTSDPPAHTRMRKQLAPGMARGALETWRPMV

DQLARELVGGLLTQTPADVVSTVAAPMPMRAITSVLGVDGPDEAAFCRLS

NQAVRITDVALSASGLISLVQGFAGFRRLRALFTHRRDNGLLRECTVLGK

LATHAEQGRLSDDELFFFAVLLLVAGYESTAHMISTLFLTLADYPDQLTL

LAQQPDLIPSAIEEHLRFISPIQNICRTTRVDYSVGQAVIPAGSLVLLAW

GAANRDPRQYEDPDVFRADRNPVGHLAFGSGIHLCPGTQLARMEGQAILR

EIVANIDRIEVVEPPTWTTNANLRGLTRLRVAVTPRVAP

>CYP128A1 (2647009125) Mycobacterium tuberculosis 323

MTATQSPPEPAPDRVRLAGCPLAGTPDVGLTAQDATTALGVPTRRRASSG

GIPVATSMWRDAQTVRTYGPAVAKALALRVAGKARSRLTGRHCRKFMQLT

DFDPFDPAIAADPYPHYRELLAGERVQYNPKRDVYILSRYADVREAARNH

DTLSSARGVTFSRGWLPFLPTSDPPAHTRMRKQLAPGMARGALETWRPMV

DQLARELVGGLLTQTPADVVSTVAAPMPMRAITSVLGVDGPDEAAFCRLS

NQAVRITDVALSASGLISLVQGFAGFRRLRALFTHRRDNGLLRECTVLGK

LATHAEQGRLSDDELFFFAVLLLVAGYESTAHMISTLFLTLADYPDQLTL

LAQQPDLIPSAIEEHLRFISPIQNICRTTRVDYSVGQAVIPAGSLVLLAW

GAANRDPRQYEDPDVFRADRNPVGHLAFGSGIHLCPGTQLARMEGQAILR

EIVANIDRIEVVEPPTWTTNANLRGLTRLRVAVTPRVAP

>CYP128A1 (2674504661) Mycobacterium tuberculosis P00401210

MTATQSPPEPAPDRVRLAGCPLAGTPDVGLTAQDATTALGVPTRRRASSG

GIPVATSMWRDAQTVRTYGPAVAKALALRVAGKARSRLTGRHCRKFMQLT

DFDPFDPAIAADPYPHYRELLAGERVQYNPKRDVYILSRYADVREAARNH

DTLSSARGVTFSRGWLPFLPTSDPPAHTRMRKQLAPGMARGALETWRPMV

DQLARELVGGLLTQTPADVVSTVAAPMPMRAITSVLGVDGPDEAAFCRLS

NQAVRITDVALSASGLISLVQGFAGFRRLRALFTHRRDNGLLRECTVLGK

LATHAEQGRLSDDELFFFAVLLLVAGYESTAHMISTLFLTLADYPDQLTL

LAQQPDLIPSAIEEHLRFISPIQNICRTTRVDYSVGQAVIPAGSLVLLAW

GAANRDPRQYEDPDVFRADRNPVGHLAFGSGIHLCPGTQLARMEGQAILR

EIVANIDRIEVVEPPTWTTNANLRGLTRLRVAVTPRVAP

>CYP128A1 (2574803870) Mycobacterium tuberculosis TKK_03_0026

MTATQSPPEPAPDRVRLAGCPLAGTPDVGLTAQDATTALGVPTRRRASSG

GIPVATSMWRDAQTVRTYGPAVAKALALRVAGKARSRLTGRHCRKFMQLT

DFDPFDPAIAADPYPHYRELLAGERVQYNPKRDVYILSRYADVREAARNH

DTLSSARGVTFSRGWLPFLPTSDPPAHTRMRKQLAPGMARGALETWRPMV

DQLARELVGGLLTQTPADVVSTVAAPMPMRAITSVLGVDGPDEAAFCRLS

NQAVRITDVALSASGLISLVQGFAGFRRLRALFTHRRDNGLLRECTVLGK

LATHAEQGRLSDDELFFFAVLLLVAGYESTAHMISTLFLTLADYPDQLTL

LAQQPDLIPSAIEEHLRFISPIQNICRTTRVDYSVGQAVIPAGSLVLLAW

GAANRDPRQYEDPDVFRADRNPVGHLAFGSGIHLCPGPSWRAWRVRRSCA

RSSPISTE

>CYP128A1 (2703456271) Mycobacterium tuberculosis B09701572

MTATQSPPEPAPDRVRLAGCPLAGTPDVGLTAQDATTALGVPTRRRASSG

GIPVATSMWRDAQTVRTYGPAVAKALALRVAGKARSRLTGRHCRKFMQLT

DFDPFDPAIAADPYPHYRELLAGERVQYNPKRDVYILSRYADVREAARNH

DTLSSARGVTFSRGWLPFLPTSDPPAHTRMRKQLAPGMARGALETWRPMV

DQLARELVGGLLTQTPADVVSTVAAPMPMRAITSVLGVDGPDEAAFCRLS

NQAVRITDVALSASGLISLVQGFAGFRRLRALFTHRRDNGLLRECTVLGK

LATHAEQGRLSDDELFFFAVLLLVAGYESTAHMISTLFLTLADYPDQLTL

LAQQPDLIPSAIEEHLRFISPIQNICRTTRVDYSVGQAVIPAGSLVLLAW

GAANRDPRQYEDPDVFRADRNPVGHLAFGSGIHLCPGPSWRAWRVRRSCA

RSSPISTE

>CYP128A1 (2810792334) Mycobacterium colombiense 852002-51834_SCH5396731

VPQGGGCPFGRGGSYSVPATRDLPLPIGEVAARRVPTLAATMRRDAQLAR

ACAPMLAKVLALTAARKVRTRLAGRHAPEHVQITEFDPMSPAIARDPYPH

YRELLAGERVQYNAKRDVYILSRYSDVREAARNHEMLSSAQGVTFSRGAA

PFLPTSDPPAHTRMRKQLAPGMARGALESWRPMVDELARELVGGLKPQRT

ADVVSLVAAPMPMRTITNVLGIAGPDEAAFIRLSNQAARITDVNLSVSGL

GSLVHGFTGFRRLRALFTHMRDNGQLGECTVLGRLAGHADRGRLSDDELF

LFAVLLVVAGHETTANMIGTLFLTLAEYPEQLRLLAQRPELIPSAIEEQL

RFLPPVQNMCRTTRVDYRVGRAVIPAGSRVLLLWGAANRDPRQYDDPDVF

RAERNPAGHLAFGSGIHSCPGTHLARMEGQAVLREIVTNLDRIEVVEPPT

WTTNANLRGLVRLRVAVTPRATA

>CYP128A1 (2745880597) Mycobacterium kansasii 1010001469

MARGAVESWRPMVDRLAHELVVELLTRTPADVVATVAAPLPMRTITTVLG

VPEPDETMFCRLSNQAARITDVTLSASGLVSLAQGFNGFRRLRALFTQRR

ANGLLGAHTVFGRLAAHAEHGRLSDDELFFFAVLLLVAGYESTAHMISTL

FLTLAEFPDQLRLLARRPNLIPSAIEEQLRFVSPIQNICRTTRVDYQVGG

AIIPAGSLVLLAWGAANRDPRQYDDPDVFRADRNPTGHLAFGSGIHLCPG

TQLARMEGHAVLREIVTNIDRIDVVDAPVWTTSANLRGPTLLRVAVTPRA

SR

>CYP128B4 (2801868666) Mycobacteroides abscessus abscessus 38

MSRWRSDSAVRQIGAPVVAAVGMNIAAAVRVRRRGYAGWTGAVNTDYDPL

DPATAAQPFDAYRALHAGGRVHYNPKRATFILSRHEDIRAALRDTDAVTS

SQGVTRMKISAPILVLTDGDDHTRLRKQVQPGFTRGAMSDWQGMADQLAK

ELVADVVANPGCDVMERLAVPLPIRMIAHIIGIPPEDVQNFRSWSEDGVG

VINAGVSPAGLRQGLKGVRAIAALRRYFKDQLASGKLKGSDTVLGRLVDN

NEDGKLSDDELFFIAMLLLFAGNETTTNLIGGMFDTLAHAPDQFAMIRDD

PDLIPSAVEEQLRYSAPIQNLYRYTRTDYRVGEVTIPSGSRLLLAFGAAN

RDPEVFEDPDTYRADRNPRNHIAFGYGVHMCIGATLSRMEGQAVLRELTS

QASAIAAAGSATWSTNSSLRGTTYLPIRLTPAR

>CYP128A1 (2547881406) Mycobacterium tuberculosis CTRI-4

MTATQSPPEPAPDRVRLAGCPLAGTPDVGLTAQDATTALGVPTRRRASSG

GIPVATSMWRDAQTVRTYGPAVAKALALRVAGKARSRLTGRHCRKFMQLT

DFDPFDPAIAADPYPHYRELLAGERVQYNPKRDVYILSRYADVREAARNH

DTLSSARGVTFSRGWLPFLPTSDPPAHTRMRKQLAPGMARGALETWRPMV

DQLARELVGGLLTQTPADVVSTVAAPMPMRAITSVLGVDGPDEAAFCRLS

NQAVRITDVALSASGLISLVQGFAGFRRLRALFTHRRDNGLLRECTVLGK

LATHAEQGRLSDDELFFFAVLLLVAGYESTAHMISTLFLTLADYPDQLTL

LAQQPDLIPSAIEEHLRFISPIQNICRTTRVDYSVGQAVIPAGSLVLLAW

GAANRDPRQYEDPDVFRADRNPVGHLAFGSGIHLCPGTQLARMEGQAILR

EIVANIDRIEVVEPPTWTTNANLRGLTRLRVAVTPRVAP

>CYP128A1 (2577953214) Mycobacterium tuberculosis 1615

MTATQSPPEPAPDRVRLAGCPLAGTPDVGLTAQDATTALGVPTRRRASSG

GIPVATSMWRDAQTVRTYGPAVAKALALRVAGKARSRLTGRHCRKFMQLT

DFDPFDPAIAADPYPHYRELLAGERVQYNPKRDVYILSRYADVREAARNH

DTLSSARGVTFSRGWLPFLPTSDPPAHTRMRKQLAPGMARGALETWRPMV

DQLARELVGGLLTQTPADVVSTVAAPMPMRAITSVLGVDGPDEAAFCRLS

NQAVRITDVALSASGLISLVQGFAGFRRLRALFTHRRDNGLLRECTVLGK

LATHAEQGRLSDDELFFFAVLLLVAGYESTAHMISTLFLTLADYPDQLTL

LAQQPDLIPSAIEEHLRFISPIQNICRTTRVDYSVGQAVIPAGSLVLLAW

GAANRDPRQYEDPDVFRADRNPVGHLAFGSGIHLCPGTQLARMEGQAILR

EIVANIDRIEVVEPPTWTTNANLRGLTRLRVAVTPRVAP

>CYP128A1 (2592325042) Mycobacterium tuberculosis TKK_03_0020

MTATQSPPEPAPDRVRLAGCPLAGTPDVGLTAQDATTALGVPTRRRASSG

GIPVATSMWRDAQTVRTYGPAVAKALALRVAGKARSRLTGRHCRKFMQLT

DFDPFDPAIAADPYPHYRELLAGERVQYNPKRDVYILSRYADVREAARNH

DTLSSARGVTFSRGWLPFLPTSDPPAHTRMRKQLAPGMARGALETWRPMV

DQLARELVGGLLTQTPADVVSTVAAPMPMRAITSVLGVDGPDEAAFCRLS

NQAVRITDVALSASGLISLVQGFAGFRRLRALFTHRRDNGLLRECTVLGK

LATHAEQGRLSDDELFFFAVLLLVAGYESTAHMISTLFLTLADYPDQLTL

LAQQPDLIPSAIEEHLRFISPIQNICRTTRVDYSVGQAVIPAGSLVLLAW

GAANRDPRQYEDPDVFRADRNPVGHLAFGSGIHLCPGTQLARMEGQAILR

EIVANIDRIEVVEPPTWTTNANLRGLTRLRVAVTPRVAP

>CYP128A1 (2603282772) Mycobacterium tuberculosis TB_RSA52

MTATQSPPEPAPDRVRLAGCPLAGTPDVGLTAQDATTALGVPTRRRASSG

GIPVATSMWRDAQTVRTYGPAVAKALALRVAGKARSRLTGRHCRKFMQLT

DFDPFDPAIAADPYPHYRELLAGERVQYNPKRDVYILSRYADVREAARNH

DTLSSARGVTFSRGWLPFLPTSDPPAHTRMRKQLAPGMARGALETWRPMV

DQLARELVGGLLTQTPADVVSTVAAPMPMRAITSVLGVDGPDEAAFCRLS

NQAVRITDVALSASGLISLVQGFAGFRRLRALFTHRRDNGLLRECTVLGK

LATHAEQGRLSDDELFFFAVLLLVAGYESTAHMISTLFLTLADYPDQLTL

LAQQPDLIPSAIEEHLRFISPIQNICRTTRVDYSVGQAVIPAGSLVLLAW

GAANRDPRQYEDPDVFRADRNPVGHLAFGSGIHLCPGTQLARMEGQAILR

EIVANIDRIEVVEPPTWTTNANLRGLTRLRVAVTPRVAP

>CYP128A1 (2603362696) Mycobacterium tuberculosis BTB13-001

MTATQSPPEPAPDRVRLAGCPLAGTPDVGLTAQDATTALGVPTRRRASSG

GIPVATSMWRDAQTVRTYGPAVAKALALRVAGKARSRLTGRHCRKFMQLT

DFDPFDPAIAADPYPHYRELLAGERVQYNPKRDVYILSRYADVREAARNH

DTLSSARGVTFSRGWLPFLPTSDPPAHTRMRKQLAPGMARGALETWRPMV

DQLARELVGGLLTQTPADVVSTVAAPMPMRAITSVLGVDGPDEAAFCRLS

NQAVRITDVALSASGLISLVQGFAGFRRLRALFTHRRDNGLLRECTVLGK

LATHAEQGRLSDDELFFFAVLLLVAGYESTAHMISTLFLTLADYPDQLTL

LAQQPDLIPSAIEEHLRFISPIQNICRTTRVDYSVGQAVIPAGSLVLLAW

GAANRDPRQYEDPDVFRADRNPVGHLAFGSGIHLCPGTQLARMEGQAILR

EIVANIDRIEVVEPPTWTTNANLRGLTRLRVAVTPRVAP

>CYP128A1 (2603927166) Mycobacterium tuberculosis TRUG0105

MTATQSPPEPAPDRVRLAGCPLAGTPDVGLTAQDATTALGVPTRRRASSG

GIPVATSMWRDAQTVRTYGPAVAKALALRVAGKARSRLTGRHCRKFMQLT

DFDPFDPAIAADPYPHYRELLAGERVQYNPKRDVYILSRYADVREAARNH

DTLSSARGVTFSRGWLPFLPTSDPPAHTRMRKQLAPGMARGALETWRPMV

DQLARELVGGLLTQTPADVVSTVAAPMPMRAITSVLGVDGPDEAAFCRLS

NQAVRITDVALSASGLISLVQGFAGFRRLRALFTHRRDNGLLRECTVLGK

LATHAEQGRLSDDELFFFAVLLLVAGYESTAHMISTLFLTLADYPDQLTL

LAQQPDLIPSAIEEHLRFISPIQNICRTTRVDYSVGQAVIPAGSLVLLAW

GAANRDPRQYEDPDVFRADRNPVGHLAFGSGIHLCPGTQLARMEGQAILR

EIVANIDRIEVVEPPTWTTNANLRGLTRLRVAVTPRVAP

>CYP128A1 (2622031001) Mycobacterium tuberculosis C2

MTATQSPPEPAPDRVRLAGCPLAGTPDVGLTAQDATTALGVPTRRRASSG

GIPVATSMWRDAQTVRTYGPAVAKALALRVAGKARSRLTGRHCRKFMQLT

DFDPFDPAIAADPYPHYRELLAGERVQYNPKRDVYILSRYADVREAARNH

DTLSSARGVTFSRGWLPFLPTSDPPAHTRMRKQLAPGMARGALETWRPMV

DQLARELVGGLLTQTPADVVSTVAAPMPMRAITSVLGVDGPDEAAFCRLS

NQAVRITDVALSASGLISLVQGFAGFRRLRALFTHRRDNGLLRECTVLGK

LATHAEQGRLSDDELFFFAVLLLVAGYESTAHMISTLFLTLADYPDQLTL

LAQQPDLIPSAIEEHLRFISPIQNICRTTRVDYSVGQAVIPAGSLVLLAW

GAANRDPRQYEDPDVFRADRNPVGHLAFGSGIHLCPGTQLARMEGQAILR

EIVANIDRIEVVEPPTWTTNANLRGLTRLRVAVTPRVAP

>CYP128A1 (2630106126) Mycobacterium tuberculosis TKK_03_0047

MTATQSPPEPAPDRVRLAGCPLAGTPDVGLTAQDATTALGVPTRRRASSG

GIPVATSMWRDAQTVRTYGPAVAKALALRVAGKARSRLTGRHCRKFMQLT

DFDPFDPAIAADPYPHYRELLAGERVQYNPKRDVYILSRYADVREAARNH

DTLSSARGVTFSRGWLPFLPTSDPPAHTRMRKQLAPGMARGALETWRPMV

DQLARELVGGLLTQTPADVVSTVAAPMPMRAITSVLGVDGPDEAAFCRLS

NQAVRITDVALSASGLISLVQGFAGFRRLRALFTHRRDNGLLRECTVLGK

LATHAEQGRLSDDELFFFAVLLLVAGYESTAHMISTLFLTLADYPDQLTL

LAQQPDLIPSAIEEHLRFISPIQNICRTTRVDYSVGQAVIPAGSLVLLAW

GAANRDPRQYEDPDVFRADRNPVGHLAFGSGIHLCPGTQLARMEGQAILR

EIVANIDRIEVVEPPTWTTNANLRGLTRLRVAVTPRVAP

>CYP128A1 (2634008947) Mycobacterium tuberculosis MD15751

MTATQSPPEPAPDRVRLAGCPLAGTPDVGLTAQDATTALGVPTRRRASSG

GIPVATSMWRDAQTVRTYGPAVAKALALRVAGKARSRLTGRHCRKFMQLT

DFDPFDPAIAADPYPHYRELLAGERVQYNPKRDVYILSRYADVREAARNH

DTLSSARGVTFSRGWLPFLPTSDPPAHTRMRKQLAPGMARGALETWRPMV

DQLARELVGGLLTQTPADVVSTVAAPMPMRAITSVLGVDGPDEAAFCRLS

NQAVRITDVALSASGLISLVQGFAGFRRLRALFTHRRDNGLLRECTVLGK

LATHAEQGRLSDDELFFFAVLLLVAGYESTAHMISTLFLTLADYPDQLTL

LAQQPDLIPSAIEEHLRFISPIQNICRTTRVDYSVGQAVIPAGSLVLLAW

GAANRDPRQYEDPDVFRADRNPVGHLAFGSGIHLCPGTQLARMEGQAILR

EIVANIDRIEVVEPPTWTTNANLRGLTRLRVAVTPRVAP

>CYP128A1 (2647498976) Mycobacterium tuberculosis Mtb984

MTATQSPPEPAPDRVRLAGCPLAGTPDVGLTAQDATTALGVPTRRRASSG

GIPVATSMWRDAQTVRTYGPAVAKALALRVAGKARSRLTGRHCRKFMQLT

DFDPFDPAIAADPYPHYRELLAGERVQYNPKRDVYILSRYADVREAARNH

DTLSSARGVTFSRGWLPFLPTSDPPAHTRMRKQLAPGMARGALETWRPMV

DQLARELVGGLLTQTPADVVSTVAAPMPMRAITSVLGVDGPDEAAFCRLS

NQAVRITDVALSASGLISLVQGFAGFRRLRALFTHRRDNGLLRECTVLGK

LATHAEQGRLSDDELFFFAVLLLVAGYESTAHMISTLFLTLADYPDQLTL

LAQQPDLIPSAIEEHLRFISPIQNICRTTRVDYSVGQAVIPAGSLVLLAW

GAANRDPRQYEDPDVFRADRNPVGHLAFGSGIHLCPGTQLARMEGQAILR

EIVANIDRIEVVEPPTWTTNANLRGLTRLRVAVTPRVAP

>CYP128A1 (2692822653) Mycobacterium tuberculosis P09501329

MTATQSPPEPAPDRVRLAGCPLAGTPDVGLTAQDATTALGVPTRRRASSG

GIPVATSMWRDAQTVRTYGPAVAKALALRVAGKARSRLTGRHCRKFMQLT

DFDPFDPAIAADPYPHYRELLAGERVQYNPKRDVYILSRYADVREAARNH

DTLSSARGVTFSRGWLPFLPTSDPPAHTRMRKQLAPGMARGALETWRPMV

DQLARELVGGLLTQTPADVVSTVAAPMPMRAITSVLGVDGPDEAAFCRLS

NQAVRITDVALSASGLISLVQGFAGFRRLRALFTHRRDNGLLRECTVLGK

LATHAEQGRLSDDELFFFAVLLLVAGYESTAHMISTLFLTLADYPDQLTL

LAQQPDLIPSAIEEHLRFISPIQNICRTTRVDYSVGQAVIPAGSLVLLAW

GAANRDPRQYEDPDVFRADRNPVGHLAFGSGIHLCPGTQLARMEGQAILR

EIVANIDRIEVVEPPTWTTNANLRGLTRLRVAVTPRVAP

>CYP128A1 (2694331724) Mycobacterium tuberculosis O09501127

MTATQSPPEPAPDRVRLAGCPLAGTPDVGLTAQDATTALGVPTRRRASSG

GIPVATSMWRDAQTVRTYGPAVAKALALRVAGKARSRLTGRHCRKFMQLT

DFDPFDPAIAADPYPHYRELLAGERVQYNPKRDVYILSRYADVREAARNH

DTLSSARGVTFSRGWLPFLPTSDPPAHTRMRKQLAPGMARGALETWRPMV

DQLARELVGGLLTQTPADVVSTVAAPMPMRAITSVLGVDGPDEAAFCRLS

NQAVRITDVALSASGLISLVQGFAGFRRLRALFTHRRDNGLLRECTVLGK

LATHAEQGRLSDDELFFFAVLLLVAGYESTAHMISTLFLTLADYPDQLTL

LAQQPDLIPSAIEEHLRFISPIQNICRTTRVDYSVGQAVIPAGSLVLLAW

GAANRDPRQYEDPDVFRADRNPVGHLAFGSGIHLCPGTQLARMEGQAILR

EIVANIDRIEVVEPPTWTTNANLRGLTRLRVAVTPRVAP

>CYP128A1 (2702164276) Mycobacterium tuberculosis 1700284

MTATQSPPEPAPDRVRLAGCPLAGTPDVGLTAQDATTALGVPTRRRASSG

GIPVATSMWRDAQTVRTYGPAVAKALALRVAGKARSRLTGRHCRKFMQLT

DFDPFDPAIAADPYPHYRELLAGERVQYNPKRDVYILSRYADVREAARNH

DTLSSARGVTFSRGWLPFLPTSDPPAHTRMRKQLAPGMARGALETWRPMV

DQLARELVGGLLTQTPADVVSTVAAPMPMRAITSVLGVDGPDEAAFCRLS

NQAVRITDVALSASGLISLVQGFAGFRRLRALFTHRRDNGLLRECTVLGK

LATHAEQGRLSDDELFFFAVLLLVAGYESTAHMISTLFLTLADYPDQLTL

LAQQPDLIPSAIEEHLRFISPIQNICRTTRVDYSVGQAVIPAGSLVLLAW

GAANRDPRQYEDPDVFRADRNPVGHLAFGSGIHLCPGTQLARMEGQAILR

EIVANIDRIEVVEPPTWTTNANLRGLTRLRVAVTPRVAP

>CYP128A1 (2606357986) Mycobacterium tuberculosis XTB13-218

MTATQSPPEPAPDRVRLAGCPLAGTPDVGLTAQDATTALGVPTRRRAASG

GIPVATSMWRDAQTVRTYGPAVAKALALRVAGKARSRLTGRHCRKFMQLT

DFDPFDPAIAADPYPHYRELLAGERVQYNPKRDVYILSRYADVREAARNH

DTLSSARGVTFSRGWLPFLPTSDPPAHTRMRKQLAPGMARGALETWRPMV

DQLARELVGGLLTQTPADVVSTVAAPMPMRAITSVLGVDGPDEAAFCRLS

NQAVRITDVALSASGLISLVQGFAGFRRLRALFTHRRDNGLLRECTVLGK

LATHAEQGRLSDDELFFFAVLLLVAGYESTAHMISTLFLTLADYPDQLTL

LAQQPDLIPSAIEEHLRFISPIQNICRTTRVDYSVGQAVIPAGSLVLLAW

GAANRDPRQYEDPDVFRADRNPVGHLAFGSGIHLCPGTQLARMEGQAILR

EIVANIDRIEVVEPPTWTTNANLRGLTRLRVAVTPRVAP

>CYP128A1 (2621442787) Mycobacterium tuberculosis XTB13-133

MTATQSPPEPAPDRVRLAGCPLAGTPDVGLTAQDATTALGVPTRRRAASG

GIPVATSMWRDAQTVRTYGPAVAKALALRVAGKARSRLTGRHCRKFMQLT

DFDPFDPAIAADPYPHYRELLAGERVQYNPKRDVYILSRYADVREAARNH

DTLSSARGVTFSRGWLPFLPTSDPPAHTRMRKQLAPGMARGALETWRPMV

DQLARELVGGLLTQTPADVVSTVAAPMPMRAITSVLGVDGPDEAAFCRLS

NQAVRITDVALSASGLISLVQGFAGFRRLRALFTHRRDNGLLRECTVLGK

LATHAEQGRLSDDELFFFAVLLLVAGYESTAHMISTLFLTLADYPDQLTL

LAQQPDLIPSAIEEHLRFISPIQNICRTTRVDYSVGQAVIPAGSLVLLAW

GAANRDPRQYEDPDVFRADRNPVGHLAFGSGIHLCPGTQLARMEGQAILR

EIVANIDRIEVVEPPTWTTNANLRGLTRLRVAVTPRVAP

>CYP128A1 (2577144100) Mycobacterium tuberculosis 2094HD

MTATQSPPEPAPDRVRLAGCPLAGTPDVGLTAQDATTALGVPTRRRASSG

GIPVATSMWRDAQTVRTYGPAVAKALALRVAGKARSRLTGRHCRKFMQLT

DFDPFDPAIAADPYPHYRELLAGERVQYNPKRDVYILSRYADVREAARNH

DTLSSARGVTFSRGWLPFLPTSDPPAHTRMRKQLAPGMARGALETWRPMV

DQLARELVGGLLTQTPADVVSTVAAPMPMRAITSVLGVDGPDEAAFCRLS

NQAVRITDVALSASGLISLVQGFAGFRRLRALFTHRRDNGLLRECTVLGK

LATHAEQGRLSDDELFFFAVLLLVAGYESTAHMISTLFLTLADYPDQLTL

LAQQPDLIPSAIEEHLRFISPIQNICRTTRVDYSVGQAVIPAGSLVLLAW

GAANRDPRQYEDPDVFRADRNPVGHLAFGSGIHLCPGPSWRAWRVRRSCA

RSSPISTE

>CYP128B4 (2801979228) Mycobacteroides abscessus abscessus 40

MSRWRSDSAVRQIGAPVVAAVGMNIAAAVRVRRRGYAGWTGAVNTDYDPL

DPATAAQPFDAYRALHAGGRVHYNPKRATFILSRHEDIRAALRDTDAVTS

SQGVTRMKISAPILVLTDGDDHTRLRKQVQPGFTRGAMSDWQGMADQLAK

ELVADVVANPGCDVMERLAVPLPIRMIAHIIGIPPEDVQNFRSWSEDGVG

VINAGVSPAGLRQGLKGVRAIAALRRYFKDQLASGKLKGSDTVLGRLVDN

NEDGKLSDDELFFIAMLLLFAGNETTTNLIGGMFDTLAHAPDQFAMIRDD

PDLIPSAVEEQLRYSAPIQNLYRYTRTDYRVGEVTIPSGSRLLLAFGAAN

RDPEVFEDPDTYRADRNPRNHIAFGYGVHMCIGATLSRMEGQAVLRELTS

QASAIAAAGSATWSTNSSLRGTTYLPIRLTPAR

>CYP128A1 (2575382312) Mycobacterium tuberculosis TBR28

MTATQSPPEPAPDRVRLAGCPLAGTPDVGLTAQDATTALGVPTRRRASSG

GIPVATSMWRDAQTVRTYGPAVAKALALRVAGKARSRLTGRHCRKFMQLT

DFDPFDPAIAADPYPHYRELLAGERVQYNPKRDVYILSRYADVREAARNH

DTLSSARGVTFSRGWLPFLPTSDPPAHTRMRKQLAPGMARGALETWRPMV

DQLARELVGGLLTQTPADVVSTVAAPMPMRAITSVLGVDGPDEAAFCRLS

NQAVRITDVALSASGLISLVQGFAGFRRLRALFTHRRDNGLLRECTVLGK

LATHAEQGRLSDDELFFFAVLLLVAGYESTAHMISTLFLTLADYPDQLTL

LAQQPDLIPSAIEEHLRFISPIQNICRTTRVDYSVGQAVIPAGSLVLLAW

GAANRDPRQYEDPDVFRADRNPVGHLAFGSGIHLCPGTQLARMEGQAILR

EIVANIDRIEVVEPPTWTTNANLRGLTRLRVAVTPRVAP

>CYP128A1 (2603922770) Mycobacterium tuberculosis TRUG0081

MTATQSPPEPAPDRVRLAGCPLAGTPDVGLTAQDATTALGVPTRRRASSG

GIPVATSMWRDAQTVRTYGPAVAKALALRVAGKARSRLTGRHCRKFMQLT

DFDPFDPAIAADPYPHYRELLAGERVQYNPKRDVYILSRYADVREAARNH

DTLSSARGVTFSRGWLPFLPTSDPPAHTRMRKQLAPGMARGALETWRPMV

DQLARELVGGLLTQTPADVVSTVAAPMPMRAITSVLGVDGPDEAAFCRLS

NQAVRITDVALSASGLISLVQGFAGFRRLRALFTHRRDNGLLRECTVLGK

LATHAEQGRLSDDELFFFAVLLLVAGYESTAHMISTLFLTLADYPDQLTL

LAQQPDLIPSAIEEHLRFISPIQNICRTTRVDYSVGQAVIPAGSLVLLAW

GAANRDPRQYEDPDVFRADRNPVGHLAFGSGIHLCPGTQLARMEGQAILR

EIVANIDRIEVVEPPTWTTNANLRGLTRLRVAVTPRVAP

>CYP128A1 (2603949987) Mycobacterium tuberculosis TB_RSA113

MTATQSPPEPAPDRVRLAGCPLAGTPDVGLTAQDATTALGVPTRRRASSG

GIPVATSMWRDAQTVRTYGPAVAKALALRVAGKARSRLTGRHCRKFMQLT

DFDPFDPAIAADPYPHYRELLAGERVQYNPKRDVYILSRYADVREAARNH

DTLSSARGVTFSRGWLPFLPTSDPPAHTRMRKQLAPGMARGALETWRPMV

DQLARELVGGLLTQTPADVVSTVAAPMPMRAITSVLGVDGPDEAAFCRLS

NQAVRITDVALSASGLISLVQGFAGFRRLRALFTHRRDNGLLRECTVLGK

LATHAEQGRLSDDELFFFAVLLLVAGYESTAHMISTLFLTLADYPDQLTL

LAQQPDLIPSAIEEHLRFISPIQNICRTTRVDYSVGQAVIPAGSLVLLAW

GAANRDPRQYEDPDVFRADRNPVGHLAFGSGIHLCPGTQLARMEGQAILR

EIVANIDRIEVVEPPTWTTNANLRGLTRLRVAVTPRVAP

>CYP128A1 (2604291550) Mycobacterium tuberculosis TB_RSA110

MTATQSPPEPAPDRVRLAGCPLAGTPDVGLTAQDATTALGVPTRRRASSG

GIPVATSMWRDAQTVRTYGPAVAKALALRVAGKARSRLTGRHCRKFMQLT

DFDPFDPAIAADPYPHYRELLAGERVQYNPKRDVYILSRYADVREAARNH

DTLSSARGVTFSRGWLPFLPTSDPPAHTRMRKQLAPGMARGALETWRPMV

DQLARELVGGLLTQTPADVVSTVAAPMPMRAITSVLGVDGPDEAAFCRLS

NQAVRITDVALSASGLISLVQGFAGFRRLRALFTHRRDNGLLRECTVLGK

LATHAEQGRLSDDELFFFAVLLLVAGYESTAHMISTLFLTLADYPDQLTL

LAQQPDLIPSAIEEHLRFISPIQNICRTTRVDYSVGQAVIPAGSLVLLAW

GAANRDPRQYEDPDVFRADRNPVGHLAFGSGIHLCPGTQLARMEGQAILR

EIVANIDRIEVVEPPTWTTNANLRGLTRLRVAVTPRVAP

>CYP128A1 (2605683265) Mycobacterium tuberculosis TRUG0038

MTATQSPPEPAPDRVRLAGCPLAGTPDVGLTAQDATTALGVPTRRRASSG

GIPVATSMWRDAQTVRTYGPAVAKALALRVAGKARSRLTGRHCRKFMQLT

DFDPFDPAIAADPYPHYRELLAGERVQYNPKRDVYILSRYADVREAARNH

DTLSSARGVTFSRGWLPFLPTSDPPAHTRMRKQLAPGMARGALETWRPMV

DQLARELVGGLLTQTPADVVSTVAAPMPMRAITSVLGVDGPDEAAFCRLS

NQAVRITDVALSASGLISLVQGFAGFRRLRALFTHRRDNGLLRECTVLGK

LATHAEQGRLSDDELFFFAVLLLVAGYESTAHMISTLFLTLADYPDQLTL

LAQQPDLIPSAIEEHLRFISPIQNICRTTRVDYSVGQAVIPAGSLVLLAW

GAANRDPRQYEDPDVFRADRNPVGHLAFGSGIHLCPGTQLARMEGQAILR

EIVANIDRIEVVEPPTWTTNANLRGLTRLRVAVTPRVAP

>CYP128A1 (2622557274) Mycobacterium tuberculosis M1439

MTATQSPPEPAPDRVRLAGCPLAGTPDVGLTAQDATTALGVPTRRRASSG

GIPVATSMWRDAQTVRTYGPAVAKALALRVAGKARSRLTGRHCRKFMQLT

DFDPFDPAIAADPYPHYRELLAGERVQYNPKRDVYILSRYADVREAARNH

DTLSSARGVTFSRGWLPFLPTSDPPAHTRMRKQLAPGMARGALETWRPMV

DQLARELVGGLLTQTPADVVSTVAAPMPMRAITSVLGVDGPDEAAFCRLS

NQAVRITDVALSASGLISLVQGFAGFRRLRALFTHRRDNGLLRECTVLGK

LATHAEQGRLSDDELFFFAVLLLVAGYESTAHMISTLFLTLADYPDQLTL

LAQQPDLIPSAIEEHLRFISPIQNICRTTRVDYSVGQAVIPAGSLVLLAW

GAANRDPRQYEDPDVFRADRNPVGHLAFGSGIHLCPGTQLARMEGQAILR

EIVANIDRIEVVEPPTWTTNANLRGLTRLRVAVTPRVAP

>CYP128A1 (2629406400) Mycobacterium tuberculosis M2350

MTATQSPPEPAPDRVRLAGCPLAGTPDVGLTAQDATTALGVPTRRRASSG

GIPVATSMWRDAQTVRTYGPAVAKALALRVAGKARSRLTGRHCRKFMQLT

DFDPFDPAIAADPYPHYRELLAGERVQYNPKRDVYILSRYADVREAARNH

DTLSSARGVTFSRGWLPFLPTSDPPAHTRMRKQLAPGMARGALETWRPMV

DQLARELVGGLLTQTPADVVSTVAAPMPMRAITSVLGVDGPDEAAFCRLS

NQAVRITDVALSASGLISLVQGFAGFRRLRALFTHRRDNGLLRECTVLGK

LATHAEQGRLSDDELFFFAVLLLVAGYESTAHMISTLFLTLADYPDQLTL

LAQQPDLIPSAIEEHLRFISPIQNICRTTRVDYSVGQAVIPAGSLVLLAW

GAANRDPRQYEDPDVFRADRNPVGHLAFGSGIHLCPGTQLARMEGQAILR

EIVANIDRIEVVEPPTWTTNANLRGLTRLRVAVTPRVAP

>CYP128A1 (2631968714) Mycobacterium tuberculosis MD17901

MTATQSPPEPAPDRVRLAGCPLAGTPDVGLTAQDATTALGVPTRRRASSG

GIPVATSMWRDAQTVRTYGPAVAKALALRVAGKARSRLTGRHCRKFMQLT

DFDPFDPAIAADPYPHYRELLAGERVQYNPKRDVYILSRYADVREAARNH

DTLSSARGVTFSRGWLPFLPTSDPPAHTRMRKQLAPGMARGALETWRPMV

DQLARELVGGLLTQTPADVVSTVAAPMPMRAITSVLGVDGPDEAAFCRLS

NQAVRITDVALSASGLISLVQGFAGFRRLRALFTHRRDNGLLRECTVLGK

LATHAEQGRLSDDELFFFAVLLLVAGYESTAHMISTLFLTLADYPDQLTL

LAQQPDLIPSAIEEHLRFISPIQNICRTTRVDYSVGQAVIPAGSLVLLAW

GAANRDPRQYEDPDVFRADRNPVGHLAFGSGIHLCPGTQLARMEGQAILR

EIVANIDRIEVVEPPTWTTNANLRGLTRLRVAVTPRVAP

>CYP128A1 (2641738643) Mycobacterium tuberculosis M2278

MTATQSPPEPAPDRVRLAGCPLAGTPDVGLTAQDATTALGVPTRRRASSG

GIPVATSMWRDAQTVRTYGPAVAKALALRVAGKARSRLTGRHCRKFMQLT

DFDPFDPAIAADPYPHYRELLAGERVQYNPKRDVYILSRYADVREAARNH

DTLSSARGVTFSRGWLPFLPTSDPPAHTRMRKQLAPGMARGALETWRPMV

DQLARELVGGLLTQTPADVVSTVAAPMPMRAITSVLGVDGPDEAAFCRLS

NQAVRITDVALSASGLISLVQGFAGFRRLRALFTHRRDNGLLRECTVLGK

LATHAEQGRLSDDELFFFAVLLLVAGYESTAHMISTLFLTLADYPDQLTL

LAQQPDLIPSAIEEHLRFISPIQNICRTTRVDYSVGQAVIPAGSLVLLAW

GAANRDPRQYEDPDVFRADRNPVGHLAFGSGIHLCPGTQLARMEGQAILR

EIVANIDRIEVVEPPTWTTNANLRGLTRLRVAVTPRVAP

>CYP128A1 (2646189190) Mycobacterium tuberculosis TKK_04_0118

MTATQSPPEPAPDRVRLAGCPLAGTPDVGLTAQDATTALGVPTRRRASSG

GIPVATSMWRDAQTVRTYGPAVAKALALRVAGKARSRLTGRHCRKFMQLT

DFDPFDPAIAADPYPHYRELLAGERVQYNPKRDVYILSRYADVREAARNH

DTLSSARGVTFSRGWLPFLPTSDPPAHTRMRKQLAPGMARGALETWRPMV

DQLARELVGGLLTQTPADVVSTVAAPMPMRAITSVLGVDGPDEAAFCRLS

NQAVRITDVALSASGLISLVQGFAGFRRLRALFTHRRDNGLLRECTVLGK

LATHAEQGRLSDDELFFFAVLLLVAGYESTAHMISTLFLTLADYPDQLTL

LAQQPDLIPSAIEEHLRFISPIQNICRTTRVDYSVGQAVIPAGSLVLLAW

GAANRDPRQYEDPDVFRADRNPVGHLAFGSGIHLCPGTQLARMEGQAILR

EIVANIDRIEVVEPPTWTTNANLRGLTRLRVAVTPRVAP

>CYP128A1 (2695921935) Mycobacterium tuberculosis A70144_2

MTATQSPPEPAPDRVRLAGCPLAGTPDVGLTAQDATTALGVPTRRRASSG

GIPVATSMWRDAQTVRTYGPAVAKALALRVAGKARSRLTGRHCRKFMQLT

DFDPFDPAIAADPYPHYRELLAGERVQYNPKRDVYILSRYADVREAARNH

DTLSSARGVTFSRGWLPFLPTSDPPAHTRMRKQLAPGMARGALETWRPMV

DQLARELVGGLLTQTPADVVSTVAAPMPMRAITSVLGVDGPDEAAFCRLS

NQAVRITDVALSASGLISLVQGFAGFRRLRALFTHRRDNGLLRECTVLGK

LATHAEQGRLSDDELFFFAVLLLVAGYESTAHMISTLFLTLADYPDQLTL

LAQQPDLIPSAIEEHLRFISPIQNICRTTRVDYSVGQAVIPAGSLVLLAW

GAANRDPRQYEDPDVFRADRNPVGHLAFGSGIHLCPGTQLARMEGQAILR

EIVANIDRIEVVEPPTWTTNANLRGLTRLRVAVTPRVAP

>CYP128A1 (2691881126) Mycobacterium tuberculosis 200541

MTATQSPPEPAPDRVRLAGCPLAGTPDVGLTAQDATTALGVPTRRRASSG

GIPVATSMWRDAQTVRTYGPAVAKALALRVAGKARSRLTGRHCRKFMQLT

DFDPFDPAIAADPYPHYRELLAGERVQYNPKRDVYILSRYADVREAARNH

DTLSSARGVTFSRGWLPFLPTSDPPAHTRMRKQLAPGMARGALETWRPMV

DQLARELVGGLLTQTPADVVSTVAAPMPMRAITSVLGVDGPDEAAFCRLS

NQAVRITDVALSASGLISLVQGFAGFRRLRALFTHRRDNGLLRECTVLGK

LATHAEQGRLSDDELFFFAVLLLVAGYESPAHMISTLFLTLADYPDQLTL

LAQQPDLIPSAIEEHLRFISPIQNICRTTRVDYSVGQAVIPAGSLVLLAW

GAANRDPRQYEDPDVFRADRNPVGHLAFGSGIHLCPGTQLARMEGQAILR

EIVANIDRIEVVEPPTWTTNANLRGLTRLRVAVTPRVAP

>CYP128A1 (2577270067) Mycobacterium tuberculosis M1004

MTATQSPPEPAPDRVRLAGCPLAGTPDVGLTAQDATTALGVPTRRRASSG

GIPVATSMWRDAQTVRTYGPAVAKALALRVAGKARSRLTGRHCRKFMQLT

DFDPFDPAIAADPYPHYRELLAGERVQYNPKRDVYILSRYADVREAARNH

DTLSSARGVTFSRGWLPFLPTSDPPAHTRMRKQLAPGMARGALETWRPMV

DQLARELVGGLLTQTPADVVSTVAAPMPMRAITSVLGVDGPDEAAFCRLS

NQAVRITDVALSASGLISLVGFAGFRRLRALFTHRRDNGLLRECTVLGKL

ATHAEQGRLSDDELFFFAVLLLVAGYESTAHMISTLFLTLADYPDQLTLL

AQQPDLIPSAIEEHLRFISPIQNICRTTRVDYSVGQAVIPAGSLVLLAWG

AANRDPRQYEDPDVFRADRNPVGHLAFGSGIHLCPGTQLARMEGQAILRE

IVANIDRIEVVEPPTWTTNANLRGLTRLRVAVTPRVAP

>CYP128A1 (2540555207) Mycobacterium canettii CIPT 140070010

MTAAQSPPEPVPDRVQLAGCPLAGTPDVGLTTQDATAALGGPMRRRGSSG

GIPVATSMWRDARTVRTYGPAVAKALALRVAGKARSRLAGRHCRKSMQLT

DFDPFDPAIAADPYPHYRELLTGERVQYNRKRDVYILSRYADVREAARNH

DTLSSARGVTFSRGWLPFLPTSDPPAHTRMRKQLAPGMARGALETWRPMV

DQLARELVGGLLTQTPADVVSTVAAPMPMRAITSVLGVDGPDEAAFCRLS

NQAVRITDVALSASGLISLVQGFAGFRRLRALFTHRRDNGLLRECTVLGK

LATHAEQGRLSDDELFFFAVLLLVAGYESTAHMISTLFLTLADYPDQLTL

LAQQPDLIPSAIEEHLRFVSPIQNICRTTRVDYSVGQAVIPAGSLVLLAW

GAANRDPRQYDDPDVFRADRNPVGHLAFGSGIHLCPGTQLVRMEGQAILR

EIVANFDRIEVVEPPTWTTNANLRGLTRLRVAVTPRVAP

>CYP128A1 (2678228049) Mycobacterium tuberculosis G00000978

VVTFSRGWLPFLPTSDPPAHTRMRKQLAPGMARGALETWRPMVDQLAREL

VGGLLTQTPADVVSTVAAPMPMRAITSVLGVDGPDEAAFCRLSNQAVRIT

DVALSASGLISLVQGFAGFRRLRALFTHRRDNGLLRECTVLGKLATHAEQ

GRLSDDELFFFAVLLLVAGYESTAHMISTLFLTLADYPDQLTLLAQQPDL

IPSAIEEHLRFISPIQNICRTTRVDYSVGQAVIPAGSLVLLAWGAANRDP

RQYEDPDVFRADRNPVGHLAFGSGIHLCPGTQLARMEGQAILREIVANID

RIEVVEPPTWTTNANLRGLTRLRVAVTPRVAP

>CYP128A1 (2540553410) Mycobacterium canettii CIPT 140070010

MTATRSSADHPSAEVAQGGCPFDRGVRPALALKGAAKPGESVRWRASSRV

SLPVSARLRRDVQLARLYAPMLAKTLALTLARKARSKLVVEHDPKHVAIT

DFDPFDPTVARDPYPHYRALLAGPRVHYNPKRDVYILSRYADVRAAARNH

DVLSSAGGVTYSRLQLPFLPTSDPPEHTRMRKQLRPAFTRSALESWRPTI

DQLAQELIARLMTQAGADVVSTVAAPIPMRTITHILGVSGPDQAAFRDWS

SQAARMTNINLSASGLFSLARTFNGFRHLHAFFTQRLRHDGPLRVETVLE

RLAAQADDGALSDEELFFFAVLLLVAGYESTANLLSTLFLTLASWPDQLR

LLAQRPELIPSAIEEQLRIASPIQNICRTTRVDYPVGRAVIPKGSLVLLA

WGAANRDPRQFDDPDVFRADRNPTAHVAFGSGIHSCPGAQLARMEGQAVL

REIVENIERIEVVEPPRWSTNANLRGLTRLRVSVTRRTPASGPSQI

>CYP128B1 (2730964375) Mycobacterium phlei DSM 43239

MSQTVRLVGDAVRMNVAAAIRTRRRGYTGWTGAINTDYDPQDPATAAQPF

DAYRALHRGGRVHYNPRRATFIVAGLDDVRAALRDTDQVTSSQGVTRLRF

SAPLAVLTDGDEHARLRRQVQPGFSRGAMESWQEIVEKLAVELVIELLNN

PGCDVVTRLAIPMPIRLIAAILGVPDTDLDDFRRWSERGVGLMELKPTIT

GVADAARSAVAMAALHRYFRRQFAAGRLKGSDTVLGRLLAHNTDGSLTDQ

QLLLIAIHLLIAGNETTTNLLGGMFDTFARHPDQYDLIRANPDLIPLAVE

EHLRFTTPIQNLYRYTRADYRIGDVVIPTGSRVLLSFGAANRDPTVFDEP

DEFRADRDPRKHVAFGYGAHMCLGAPLARMEAQAVLRQLVTRVARISPAG

PTKWSTHSSLRGPTRLPIHLTPA

>CYP128A1 (2575665019) Mycobacterium tuberculosis M1233

MTATQSPPEPAPDRVRLAGCPLAGTPDVGLTAQDATTALGVPTRRRASSG

GIPVATSMWRDAQTVRTYGPAVAKALALRVAGKARSRLTGRHCRKFMQLT

DFDPFDPAIAADPYPHYRELLAGERVQYNPKRDVYILSRYADVREAARNH

DTLSSARGVTFSRGWLPFLPTSDPPAHTRMRKQLAPGMARGALETWRPMV

DQLARELVGGLLTQTPADVVSTVAAPMPMRAITSVLGVDGPDEAAFCRLS

NQAVRITDVALSASGLISLVQGFAGFRRLRALFTHRRDNGLLRECTVLGK

LATHAEQGRLSDDELFFFAVLLLVAGYESTAHMISTLFLTLADYPDQLTL

LAQQPDLIPSAIEEHLRFISPIQNICRTTRVDYSVGQAVIPAGSLVLLAW

GAANRDPRQYEDPDVFRADRNPVGHLAFGSGIHLCPGTQLARMEGQAILR

EIVANIDRIEVVEPPTWTTNANLRGLTRLRVAVTPRVAP

>CYP128A1 (2575787523) Mycobacterium tuberculosis TKK-01-0019

MTATQSPPEPAPDRVRLAGCPLAGTPDVGLTAQDATTALGVPTRRRASSG

GIPVATSMWRDAQTVRTYGPAVAKALALRVAGKARSRLTGRHCRKFMQLT

DFDPFDPAIAADPYPHYRELLAGERVQYNPKRDVYILSRYADVREAARNH

DTLSSARGVTFSRGWLPFLPTSDPPAHTRMRKQLAPGMARGALETWRPMV

DQLARELVGGLLTQTPADVVSTVAAPMPMRAITSVLGVDGPDEAAFCRLS

NQAVRITDVALSASGLISLVQGFAGFRRLRALFTHRRDNGLLRECTVLGK

LATHAEQGRLSDDELFFFAVLLLVAGYESTAHMISTLFLTLADYPDQLTL

LAQQPDLIPSAIEEHLRFISPIQNICRTTRVDYSVGQAVIPAGSLVLLAW

GAANRDPRQYEDPDVFRADRNPVGHLAFGSGIHLCPGTQLARMEGQAILR

EIVANIDRIEVVEPPTWTTNANLRGLTRLRVAVTPRVAP

>CYP128A1 (2576431734) Mycobacterium tuberculosis BTB13-222

MTATQSPPEPAPDRVRLAGCPLAGTPDVGLTAQDATTALGVPTRRRASSG

GIPVATSMWRDAQTVRTYGPAVAKALALRVAGKARSRLTGRHCRKFMQLT

DFDPFDPAIAADPYPHYRELLAGERVQYNPKRDVYILSRYADVREAARNH

DTLSSARGVTFSRGWLPFLPTSDPPAHTRMRKQLAPGMARGALETWRPMV

DQLARELVGGLLTQTPADVVSTVAAPMPMRAITSVLGVDGPDEAAFCRLS

NQAVRITDVALSASGLISLVQGFAGFRRLRALFTHRRDNGLLRECTVLGK

LATHAEQGRLSDDELFFFAVLLLVAGYESTAHMISTLFLTLADYPDQLTL

LAQQPDLIPSAIEEHLRFISPIQNICRTTRVDYSVGQAVIPAGSLVLLAW

GAANRDPRQYEDPDVFRADRNPVGHLAFGSGIHLCPGTQLARMEGQAILR

EIVANIDRIEVVEPPTWTTNANLRGLTRLRVAVTPRVAP

>CYP128A1 (2590532306) Mycobacterium tuberculosis KT-0084

MTATQSPPEPAPDRVRLAGCPLAGTPDVGLTAQDATTALGVPTRRRASSG

GIPVATSMWRDAQTVRTYGPAVAKALALRVAGKARSRLTGRHCRKFMQLT

DFDPFDPAIAADPYPHYRELLAGERVQYNPKRDVYILSRYADVREAARNH

DTLSSARGVTFSRGWLPFLPTSDPPAHTRMRKQLAPGMARGALETWRPMV

DQLARELVGGLLTQTPADVVSTVAAPMPMRAITSVLGVDGPDEAAFCRLS

NQAVRITDVALSASGLISLVQGFAGFRRLRALFTHRRDNGLLRECTVLGK

LATHAEQGRLSDDELFFFAVLLLVAGYESTAHMISTLFLTLADYPDQLTL

LAQQPDLIPSAIEEHLRFISPIQNICRTTRVDYSVGQAVIPAGSLVLLAW

GAANRDPRQYEDPDVFRADRNPVGHLAFGSGIHLCPGTQLARMEGQAILR

EIVANIDRIEVVEPPTWTTNANLRGLTRLRVAVTPRVAP

>CYP128A1 (2603383279) Mycobacterium tuberculosis F11

MTATQSPPEPAPDRVRLAGCPLAGTPDVGLTAQDATTALGVPTRRRASSG

GIPVATSMWRDAQTVRTYGPAVAKALALRVAGKARSRLTGRHCRKFMQLT

DFDPFDPAIAADPYPHYRELLAGERVQYNPKRDVYILSRYADVREAARNH

DTLSSARGVTFSRGWLPFLPTSDPPAHTRMRKQLAPGMARGALETWRPMV

DQLARELVGGLLTQTPADVVSTVAAPMPMRAITSVLGVDGPDEAAFCRLS

NQAVRITDVALSASGLISLVQGFAGFRRLRALFTHRRDNGLLRECTVLGK

LATHAEQGRLSDDELFFFAVLLLVAGYESTAHMISTLFLTLADYPDQLTL

LAQQPDLIPSAIEEHLRFISPIQNICRTTRVDYSVGQAVIPAGSLVLLAW

GAANRDPRQYEDPDVFRADRNPVGHLAFGSGIHLCPGTQLARMEGQAILR

EIVANIDRIEVVEPPTWTTNANLRGLTRLRVAVTPRVAP

>CYP128A1 (2622151354) Mycobacterium tuberculosis MD16571

MTATQSPPEPAPDRVRLAGCPLAGTPDVGLTAQDATTALGVPTRRRASSG

GIPVATSMWRDAQTVRTYGPAVAKALALRVAGKARSRLTGRHCRKFMQLT

DFDPFDPAIAADPYPHYRELLAGERVQYNPKRDVYILSRYADVREAARNH

DTLSSARGVTFSRGWLPFLPTSDPPAHTRMRKQLAPGMARGALETWRPMV

DQLARELVGGLLTQTPADVVSTVAAPMPMRAITSVLGVDGPDEAAFCRLS

NQAVRITDVALSASGLISLVQGFAGFRRLRALFTHRRDNGLLRECTVLGK

LATHAEQGRLSDDELFFFAVLLLVAGYESTAHMISTLFLTLADYPDQLTL

LAQQPDLIPSAIEEHLRFISPIQNICRTTRVDYSVGQAVIPAGSLVLLAW

GAANRDPRQYEDPDVFRADRNPVGHLAFGSGIHLCPGTQLARMEGQAILR

EIVANIDRIEVVEPPTWTTNANLRGLTRLRVAVTPRVAP

>CYP128A1 (2672977603) Mycobacterium tuberculosis D00000538

MTATQSPPEPAPDRVRLAGCPLAGTPDVGLTAQDATTALGVPTRRRASSG

GIPVATSMWRDAQTVRTYGPAVAKALALRVAGKARSRLTGRHCRKFMQLT

DFDPFDPAIAADPYPHYRELLAGERVQYNPKRDVYILSRYADVREAARNH

DTLSSARGVTFSRGWLPFLPTSDPPAHTRMRKQLAPGMARGALETWRPMV

DQLARELVGGLLTQTPADVVSTVAAPMPMRAITSVLGVDGPDEAAFCRLS

NQAVRITDVALSASGLISLVQGFAGFRRLRALFTHRRDNGLLRECTVLGK

LATHAEQGRLSDDELFFFAVLLLVAGYESTAHMISTLFLTLADYPDQLTL

LAQQPDLIPSAIEEHLRFISPIQNICRTTRVDYSVGQAVIPAGSLVLLAW

GAANRDPRQYEDPDVFRADRNPVGHLAFGSGIHLCPGTQLARMEGQAILR

EIVANIDRIEVVEPPTWTTNANLRGLTRLRVAVTPRVAP

>CYP128A1 (2673506829) Mycobacterium tuberculosis 300234

MTATQSPPEPAPDRVRLAGCPLAGTPDVGLTAQDATTALGVPTRRRASSG

GIPVATSMWRDAQTVRTYGPAVAKALALRVAGKARSRLTGRHCRKFMQLT

DFDPFDPAIAADPYPHYRELLAGERVQYNPKRDVYILSRYADVREAARNH

DTLSSARGVTFSRGWLPFLPTSDPPAHTRMRKQLAPGMARGALETWRPMV

DQLARELVGGLLTQTPADVVSTVAAPMPMRAITSVLGVDGPDEAAFCRLS

NQAVRITDVALSASGLISLVQGFAGFRRLRALFTHRRDNGLLRECTVLGK

LATHAEQGRLSDDELFFFAVLLLVAGYESTAHMISTLFLTLADYPDQLTL

LAQQPDLIPSAIEEHLRFISPIQNICRTTRVDYSVGQAVIPAGSLVLLAW

GAANRDPRQYEDPDVFRADRNPVGHLAFGSGIHLCPGTQLARMEGQAILR

EIVANIDRIEVVEPPTWTTNANLRGLTRLRVAVTPRVAP

>CYP128A1 (2693263279) Mycobacterium tuberculosis 201009

MTATQSPPEPAPDRVRLAGCPLAGTPDVGLTAQDATTALGVPTRRRASSG

GIPVATSMWRDAQTVRTYGPAVAKALALRVAGKARSRLTGRHCRKFMQLT

DFDPFDPAIAADPYPHYRELLAGERVQYNPKRDVYILSRYADVREAARNH

DTLSSARGVTFSRGWLPFLPTSDPPAHTRMRKQLAPGMARGALETWRPMV

DQLARELVGGLLTQTPADVVSTVAAPMPMRAITSVLGVDGPDEAAFCRLS

NQAVRITDVALSASGLISLVQGFAGFRRLRALFTHRRDNGLLRECTVLGK

LATHAEQGRLSDDELFFFAVLLLVAGYESTAHMISTLFLTLADYPDQLTL

LAQQPDLIPSAIEEHLRFISPIQNICRTTRVDYSVGQAVIPAGSLVLLAW

GAANRDPRQYEDPDVFRADRNPVGHLAFGSGIHLCPGTQLARMEGQAILR

EIVANIDRIEVVEPPTWTTNANLRGLTRLRVAVTPRVAP

>CYP128A1 (2697889783) Mycobacterium tuberculosis 302235

MTATQSPPEPAPDRVRLAGCPLAGTPDVGLTAQDATTALGVPTRRRASSG

GIPVATSMWRDAQTVRTYGPAVAKALALRVAGKARSRLTGRHCRKFMQLT

DFDPFDPAIAADPYPHYRELLAGERVQYNPKRDVYILSRYADVREAARNH

DTLSSARGVTFSRGWLPFLPTSDPPAHTRMRKQLAPGMARGALETWRPMV

DQLARELVGGLLTQTPADVVSTVAAPMPMRAITSVLGVDGPDEAAFCRLS

NQAVRITDVALSASGLISLVQGFAGFRRLRALFTHRRDNGLLRECTVLGK

LATHAEQGRLSDDELFFFAVLLLVAGYESTAHMISTLFLTLADYPDQLTL

LAQQPDLIPSAIEEHLRFISPIQNICRTTRVDYSVGQAVIPAGSLVLLAW

GAANRDPRQYEDPDVFRADRNPVGHLAFGSGIHLCPGTQLARMEGQAILR

EIVANIDRIEVVEPPTWTTNANLRGLTRLRVAVTPRVAP

>CYP128A1 (648490711) Mycobacterium tuberculosis SUMu012

MTATQSPPEPAPDRVRLAGCPLAGTPDVGLTAQDATTALGVPTRRRASSG

GIPVATSMWRDAQTVRTYGPAVAKALALRVAGKARSRLTGRHCRKFMQLT

DFDPFDPAIAADPYPHYRELLAGERVQYNPKRDVYILSRYADVREAARNH

DTLSSARGVTFSRGWLPFLPTSDPPAHTRMRKQLAPGMARGALETWRPMV

DQLARELVGGLLTQTPADVVSTVAAPMPMRAITSVLGVDGPDEAAFCRLS

NQAVRITDVALSASGLISLVQGFAGFRRLRALFTHRRDNGLLRECTVLGK

LATHAEQGRLSDDELFFFAVLLLVAGYESTAHMISTLFLTLADYPDQLTL

LAQQPDLIPSAIEEHLRFISPIQNICRTTRVDYSVGQAVIPAGSLVLLAW

GAANRDPRQYEDPDVFRADRNPVGHLAFGSGIHLCPGTQLARMEGQAILR

EIVANIDRIEVVEPPTWTTNANLRGLTRLRVAVTPRVAP

>CYP128A1 (2584869755) Mycobacterium tuberculosis MD16555

MTATQSPPEPAPDRVRLAGCPLAGTPDVGLTAQDATTALGVPTRRRAASG

GIPVATSMWRDAQTVRTYGPAVAKALALRVAGKARSRLTGRHCRKFMQLT

DFDPFDPAIAADPYPHYRELLAGERVQYNPKRDVYILSRYADVREAARNH

DTLSSARGVTFSRGWLPFLPTSDPPAHTRMRKQLAPGMARGALETWRPMV

DQLARELVGGLLTQTPADVVSTVAAPMPMRAITSVLGVDGPDEAAFCRLS

NQAVRITDVALSASGLISLVQGFAGFRRLRALFTHRRDNGLLRECTVLGK

LATHAEQGRLSDDELFFFAVLLLVAGYESTAHMISTLFLTLADYPDQLTL

LAQQPDLIPSAIEEHLRFISPIQNICRTTRVDYSVGQAVIPAGSLVLLAW

GAANRDPRQYEDPDVFRADRNPVGHLAFGSGIHLCPGTQLARMEGQAILR

EIVANIDRIEVVEPPTWTTNANLRGLTRLRVAVTPRVAP

>CYP128A1 (2695633051) Mycobacterium bovis BCG Tice

MTATQSPPEPAPDRVRLAGCPLAGTPDVGLTAQDATTALGVPTRRRASSG

GIPVATSMWRDAQTVRTYGPAVAKALALRVAGKARSRLTGRHCRKFMQLT

DFDPFDPAIAADPYPHYRELLAGERVQYNPKRDVYILSRYADVREAARNH

DTLSSARGVTFSRGWLPFLPTSDPPAHTRMRKQLAPGMARGALETWRPMV

DQFARELVGGLLTQTPADVVSTVAAPMPMRAITSVLGVDGPDEAAFCRLS

NQAVRITDVALSASGLISLVQGFAGFRRLRALFTHRRDNGLLRECTVLGK

LATHAEQGRLSDDELFFFAVLLLVAGYESTAHMISTLFLTLADYPDQLTL

LAQQPDLIPSAIEEHLRFISPIQNICRTTRVDYSVGQAVIPAGSLVLLAW

GAANRDPRQYEDPDVFRADRNPVGHLAFGSGIHLCPGTQLARMEGQAILR

EIVANIDRIEVVEPPTWTTNANLRGLTRLRVAVTPRVAP

>CYP128A1 (2574929175) Mycobacterium tuberculosis TKK_04_0075

MTATQSPPEPAPDRVRLAGCPLAGTPDVGLTAQDATTALGVPTRRRASSG

GIPVATSMWRDAQTVRTYGPAVAKALALRVAGKARSRLTGRHCRKFMQLT

DFDPFDPAIAADPYPHYRELLAGERVQYNPKRDVYILSRYADVREAARNH

DTLSSARGVTFSRGWLPFLPTSDPPAHTRMRKQLAPGMARGALETWRPMV

DQLARELVGGLLTQTPADVVSTVAAPMPMRAITSVLGVDGPDEAAFCRLS

NQAVRITDVALSASGLISLVQGFAGFRRLRALFTHRRDNGLLRECTVLGK

LATHAEQGRLSDDELFFFAVLLLVAGYESTAHMISTLFLTLADYPDQLTL

LAQQPDLIPSAIEEHLRFISPIQNICRTTRVDYSVGQAVIPAGSLVLLAW

GAANRDPRQYEDPDVFRADRNPVGHLAFGSGIHLCPGPSWRAWRVRRSCA

RSSPISTE

>CYP128B4 (2802867501) Mycobacteroides abscessus abscessus 1158

MSRWRSDSAVRQIGAPVVAAVGMNIAAAVRVRRRGYAGWTGAVNTDYDPL

DPATAAQPFDAYRALHAGGRVHYNPKRATFILSRHEDIRAALRDTDAVTS

SQGVTRMKISAPILVLTDGDDHTRLRKQVQPGFTRGAMSDWQGMADQLAK

ELVADVVANPGCDVMERLAVPLPIRMIAHIIGIPPEDVQNFRSWSEDGVG

VINAGVSPAGLRQGLKGVRAIAALRRYFKDQLASGKLKGSDTVLGRLVDN

NEDGKLSDDELFFIAMLLLFAGNETTTNLIGGMFDTLAHAPDQFAMIRDD

PDLIPSAVEEQLRYSAPIQNLYRYTRTDYRVGEVTIPSGSRLLLAFGAAN

RDPEVFEDPDTYRADRNPRNHIAFGYGVHMCIGATLSRMEGQAVLRELTS

QASAIAAAGSATWSTNSSLRGTTYLPIRLTPAR

>CYP128B1 (2714014010) Mycobacterium sp. UM_11

VPISEAARQATDALRPVVSTVRMNVSAAVRTRHRGHDGWIGAVNTDYDPQ

DQATAANPFDAYRALHRTGRVHYNPRRATFIVSRLGDVRAALRDTDRVTS

TQGVTRLRMSAPLAVLTDGEEHTRLRKQVQPGFSKGAMKSWQEMTETLAI

DLVTDVMSDPGCDVVQRLAIPMPIRLIAQILGVPDSDIRDFRRWSQRAVG

VMELTPTLSGVVDATKSMSAMAALLRYFAKQFAAGGLKGSDTVLGRLIEH

NTDGSLTDRQLLLIAIHLLIAGNETTTNLLGGMFDTLAHHPDQYELIRSQ

PDLIPLAVEEQLRFTSPIQNLYRYTRADYRIGDVTIPNGSRILLSFGAAN

RDPTAFDEPDEYRADRDPRTHVAFGYGAHMCLGAPLARMEAQAVLRHLVT

RVARITPAGPTQWSRHSSLRGPTRLPIRLDPA

>CYP128A1 (2584927091) Mycobacterium tuberculosis M2346

MTATQSPPEPAPDRVRLAGCPLAGTPDVGLTAQDATTALGVPTRRRASSG

GIPVATSMWRDAQTVRTYGPAVAKALALRVAGKARSRLTGRHCRKFMQLT

DFDPFDPAIAADPYPHYRELLAGERVQYNPKRDVYILSRYADVREAARNH

DTLSSARGVTFSRGWLPFLPTSDPPAHTRMRKQLAPGMARGALETWRPMV

DQLARELVGGLLTQTPADVVSTVAAPMPMRAITSVLGVDGPDEAAFCRLS

NQAVRITDVALSASGLISLVQGFAGFRRLRALFTHRRDNGLLRECTVLGK

LATHAEQGRLSDDELFFFAVLLLVAGYESTAHMISTLFLTLADYPDQLTL

LAQQPDLIPSAIEEHLRFISPIQNICRTTRVDYSVGQAVIPAGSLVLLAW

GAANRDPRQYEDPDVFRADRNPVGHLAFGSGIHLCPGTQLARMEGQAILR

EIVANIDRIEVVEPPTWTTNANLRGLTRLRVAVTPRVAP

>CYP128A1 (2603160900) Mycobacterium tuberculosis BTB07-299

MTATQSPPEPAPDRVRLAGCPLAGTPDVGLTAQDATTALGVPTRRRASSG

GIPVATSMWRDAQTVRTYGPAVAKALALRVAGKARSRLTGRHCRKFMQLT

DFDPFDPAIAADPYPHYRELLAGERVQYNPKRDVYILSRYADVREAARNH

DTLSSARGVTFSRGWLPFLPTSDPPAHTRMRKQLAPGMARGALETWRPMV

DQLARELVGGLLTQTPADVVSTVAAPMPMRAITSVLGVDGPDEAAFCRLS

NQAVRITDVALSASGLISLVQGFAGFRRLRALFTHRRDNGLLRECTVLGK

LATHAEQGRLSDDELFFFAVLLLVAGYESTAHMISTLFLTLADYPDQLTL

LAQQPDLIPSAIEEHLRFISPIQNICRTTRVDYSVGQAVIPAGSLVLLAW

GAANRDPRQYEDPDVFRADRNPVGHLAFGSGIHLCPGTQLARMEGQAILR

EIVANIDRIEVVEPPTWTTNANLRGLTRLRVAVTPRVAP

>CYP128A1 (2604956808) Mycobacterium tuberculosis BTB09-230

MTATQSPPEPAPDRVRLAGCPLAGTPDVGLTAQDATTALGVPTRRRASSG

GIPVATSMWRDAQTVRTYGPAVAKALALRVAGKARSRLTGRHCRKFMQLT

DFDPFDPAIAADPYPHYRELLAGERVQYNPKRDVYILSRYADVREAARNH

DTLSSARGVTFSRGWLPFLPTSDPPAHTRMRKQLAPGMARGALETWRPMV

DQLARELVGGLLTQTPADVVSTVAAPMPMRAITSVLGVDGPDEAAFCRLS

NQAVRITDVALSASGLISLVQGFAGFRRLRALFTHRRDNGLLRECTVLGK

LATHAEQGRLSDDELFFFAVLLLVAGYESTAHMISTLFLTLADYPDQLTL

LAQQPDLIPSAIEEHLRFISPIQNICRTTRVDYSVGQAVIPAGSLVLLAW

GAANRDPRQYEDPDVFRADRNPVGHLAFGSGIHLCPGTQLARMEGQAILR

EIVANIDRIEVVEPPTWTTNANLRGLTRLRVAVTPRVAP

>CYP128A1 (2605300512) Mycobacterium tuberculosis BTB10-257

MTATQSPPEPAPDRVRLAGCPLAGTPDVGLTAQDATTALGVPTRRRASSG

GIPVATSMWRDAQTVRTYGPAVAKALALRVAGKARSRLTGRHCRKFMQLT

DFDPFDPAIAADPYPHYRELLAGERVQYNPKRDVYILSRYADVREAARNH

DTLSSARGVTFSRGWLPFLPTSDPPAHTRMRKQLAPGMARGALETWRPMV

DQLARELVGGLLTQTPADVVSTVAAPMPMRAITSVLGVDGPDEAAFCRLS

NQAVRITDVALSASGLISLVQGFAGFRRLRALFTHRRDNGLLRECTVLGK

LATHAEQGRLSDDELFFFAVLLLVAGYESTAHMISTLFLTLADYPDQLTL

LAQQPDLIPSAIEEHLRFISPIQNICRTTRVDYSVGQAVIPAGSLVLLAW

GAANRDPRQYEDPDVFRADRNPVGHLAFGSGIHLCPGTQLARMEGQAILR

EIVANIDRIEVVEPPTWTTNANLRGLTRLRVAVTPRVAP

>CYP128A1 (2606459699) Mycobacterium tuberculosis XTB13-208

MTATQSPPEPAPDRVRLAGCPLAGTPDVGLTAQDATTALGVPTRRRASSG

GIPVATSMWRDAQTVRTYGPAVAKALALRVAGKARSRLTGRHCRKFMQLT

DFDPFDPAIAADPYPHYRELLAGERVQYNPKRDVYILSRYADVREAARNH

DTLSSARGVTFSRGWLPFLPTSDPPAHTRMRKQLAPGMARGALETWRPMV

DQLARELVGGLLTQTPADVVSTVAAPMPMRAITSVLGVDGPDEAAFCRLS

NQAVRITDVALSASGLISLVQGFAGFRRLRALFTHRRDNGLLRECTVLGK

LATHAEQGRLSDDELFFFAVLLLVAGYESTAHMISTLFLTLADYPDQLTL

LAQQPDLIPSAIEEHLRFISPIQNICRTTRVDYSVGQAVIPAGSLVLLAW

GAANRDPRQYEDPDVFRADRNPVGHLAFGSGIHLCPGTQLARMEGQAILR

EIVANIDRIEVVEPPTWTTNANLRGLTRLRVAVTPRVAP

>CYP128A1 (2621383086) Mycobacterium tuberculosis XTB13-102

MTATQSPPEPAPDRVRLAGCPLAGTPDVGLTAQDATTALGVPTRRRASSG

GIPVATSMWRDAQTVRTYGPAVAKALALRVAGKARSRLTGRHCRKFMQLT

DFDPFDPAIAADPYPHYRELLAGERVQYNPKRDVYILSRYADVREAARNH

DTLSSARGVTFSRGWLPFLPTSDPPAHTRMRKQLAPGMARGALETWRPMV

DQLARELVGGLLTQTPADVVSTVAAPMPMRAITSVLGVDGPDEAAFCRLS

NQAVRITDVALSASGLISLVQGFAGFRRLRALFTHRRDNGLLRECTVLGK

LATHAEQGRLSDDELFFFAVLLLVAGYESTAHMISTLFLTLADYPDQLTL

LAQQPDLIPSAIEEHLRFISPIQNICRTTRVDYSVGQAVIPAGSLVLLAW

GAANRDPRQYEDPDVFRADRNPVGHLAFGSGIHLCPGTQLARMEGQAILR

EIVANIDRIEVVEPPTWTTNANLRGLTRLRVAVTPRVAP

>CYP128A1 (2621551932) Mycobacterium tuberculosis XTB13-196

MTATQSPPEPAPDRVRLAGCPLAGTPDVGLTAQDATTALGVPTRRRASSG

GIPVATSMWRDAQTVRTYGPAVAKALALRVAGKARSRLTGRHCRKFMQLT

DFDPFDPAIAADPYPHYRELLAGERVQYNPKRDVYILSRYADVREAARNH

DTLSSARGVTFSRGWLPFLPTSDPPAHTRMRKQLAPGMARGALETWRPMV

DQLARELVGGLLTQTPADVVSTVAAPMPMRAITSVLGVDGPDEAAFCRLS

NQAVRITDVALSASGLISLVQGFAGFRRLRALFTHRRDNGLLRECTVLGK

LATHAEQGRLSDDELFFFAVLLLVAGYESTAHMISTLFLTLADYPDQLTL

LAQQPDLIPSAIEEHLRFISPIQNICRTTRVDYSVGQAVIPAGSLVLLAW

GAANRDPRQYEDPDVFRADRNPVGHLAFGSGIHLCPGTQLARMEGQAILR

EIVANIDRIEVVEPPTWTTNANLRGLTRLRVAVTPRVAP

>CYP128A1 (2621992785) Mycobacterium tuberculosis MAL020151

MTATQSPPEPAPDRVRLAGCPLAGTPDVGLTAQDATTALGVPTRRRASSG

GIPVATSMWRDAQTVRTYGPAVAKALALRVAGKARSRLTGRHCRKFMQLT

DFDPFDPAIAADPYPHYRELLAGERVQYNPKRDVYILSRYADVREAARNH

DTLSSARGVTFSRGWLPFLPTSDPPAHTRMRKQLAPGMARGALETWRPMV

DQLARELVGGLLTQTPADVVSTVAAPMPMRAITSVLGVDGPDEAAFCRLS

NQAVRITDVALSASGLISLVQGFAGFRRLRALFTHRRDNGLLRECTVLGK

LATHAEQGRLSDDELFFFAVLLLVAGYESTAHMISTLFLTLADYPDQLTL

LAQQPDLIPSAIEEHLRFISPIQNICRTTRVDYSVGQAVIPAGSLVLLAW

GAANRDPRQYEDPDVFRADRNPVGHLAFGSGIHLCPGTQLARMEGQAILR

EIVANIDRIEVVEPPTWTTNANLRGLTRLRVAVTPRVAP

>CYP128A1 (2622234575) Mycobacterium tuberculosis MD15758

MTATQSPPEPAPDRVRLAGCPLAGTPDVGLTAQDATTALGVPTRRRASSG

GIPVATSMWRDAQTVRTYGPAVAKALALRVAGKARSRLTGRHCRKFMQLT

DFDPFDPAIAADPYPHYRELLAGERVQYNPKRDVYILSRYADVREAARNH

DTLSSARGVTFSRGWLPFLPTSDPPAHTRMRKQLAPGMARGALETWRPMV

DQLARELVGGLLTQTPADVVSTVAAPMPMRAITSVLGVDGPDEAAFCRLS

NQAVRITDVALSASGLISLVQGFAGFRRLRALFTHRRDNGLLRECTVLGK

LATHAEQGRLSDDELFFFAVLLLVAGYESTAHMISTLFLTLADYPDQLTL

LAQQPDLIPSAIEEHLRFISPIQNICRTTRVDYSVGQAVIPAGSLVLLAW

GAANRDPRQYEDPDVFRADRNPVGHLAFGSGIHLCPGTQLARMEGQAILR

EIVANIDRIEVVEPPTWTTNANLRGLTRLRVAVTPRVAP

>CYP128A1 (2630307494) Mycobacterium tuberculosis TKK_04_0122

MTATQSPPEPAPDRVRLAGCPLAGTPDVGLTAQDATTALGVPTRRRASSG

GIPVATSMWRDAQTVRTYGPAVAKALALRVAGKARSRLTGRHCRKFMQLT

DFDPFDPAIAADPYPHYRELLAGERVQYNPKRDVYILSRYADVREAARNH

DTLSSARGVTFSRGWLPFLPTSDPPAHTRMRKQLAPGMARGALETWRPMV

DQLARELVGGLLTQTPADVVSTVAAPMPMRAITSVLGVDGPDEAAFCRLS

NQAVRITDVALSASGLISLVQGFAGFRRLRALFTHRRDNGLLRECTVLGK

LATHAEQGRLSDDELFFFAVLLLVAGYESTAHMISTLFLTLADYPDQLTL

LAQQPDLIPSAIEEHLRFISPIQNICRTTRVDYSVGQAVIPAGSLVLLAW

GAANRDPRQYEDPDVFRADRNPVGHLAFGSGIHLCPGTQLARMEGQAILR

EIVANIDRIEVVEPPTWTTNANLRGLTRLRVAVTPRVAP

>CYP128A1 (2638416520) Mycobacterium tuberculosis VRFCWCF XDRTB 232

MTATQSPPEPAPDRVRLAGCPLAGTPDVGLTAQDATTALGVPTRRRASSG

GIPVATSMWRDAQTVRTYGPAVAKALALRVAGKARSRLTGRHCRKFMQLT

DFDPFDPAIAADPYPHYRELLAGERVQYNPKRDVYILSRYADVREAARNH

DTLSSARGVTFSRGWLPFLPTSDPPAHTRMRKQLAPGMARGALETWRPMV

DQLARELVGGLLTQTPADVVSTVAAPMPMRAITSVLGVDGPDEAAFCRLS

NQAVRITDVALSASGLISLVQGFAGFRRLRALFTHRRDNGLLRECTVLGK

LATHAEQGRLSDDELFFFAVLLLVAGYESTAHMISTLFLTLADYPDQLTL

LAQQPDLIPSAIEEHLRFISPIQNICRTTRVDYSVGQAVIPAGSLVLLAW

GAANRDPRQYEDPDVFRADRNPVGHLAFGSGIHLCPGTQLARMEGQAILR

EIVANIDRIEVVEPPTWTTNANLRGLTRLRVAVTPRVAP

>CYP128A1 (2566985862) Mycobacterium canettii CIPT 140070005

MTATQSPPEPAPDRVQLAGCPSAGTPDVGLTAQDATTALGVPTRRRASSG

GIPVATSMWRDAQTVRTYGPAVAKALALRVAGKARSRLAGRHCRKFMQLT

DFDPFDPAIAADPYPHYRELLAGERVQYNPKRDVYILSRYADVREAARNH

DTLSSARGVTFSRGWLPFLPTSDPPAHTRMRKQLAPGMARGALETWRPMV

DQLARELVGELLTQTPADVVSAVAAPMPMRAITSVLGVDGPDEAAFCRLS

NQAVRITDVALSASGLISLVQGFAGFRRLRALFTHRRDNGLLRECTVLGK

LATHAEQGRLSDDELFFFAVLLLVAGYESTAHMISTLFLTLADYPDQLTL

LAQQPDLIPSAIEEHLRFISPIQNICRTTRVDYSVGQAVIPAGSLVLLAW

GAANRDPRQYEDPDVFRADRNPVGHLAFGSGIHLCPGTQLARMEGQAILR

EIVANIDRIEVVEPPTWTANANLRGLTRLRVAVTPRVAP

>CYP128B4 (2801301371) Mycobacteroides abscessus abscessus 350

MSRWRSDSAVRQIGAPVVAAVGMNIAAAVRVRRRGYAGWTGAVNTDYDPL

DPATAAQPFDAYRALHAGGRVHYNPKRATFILSRHEDIRAALRDTDAVTS

SQGVTRMKISAPILVLTDGDDHTRLRKQVQPGFTRGAMSDWQGMADQLAK

ELVADVVANPGCDVMERLAVPLPIRMIAHIIGIPPEDVQNFRSWSEDGVG

VINAGVSPAGLRQGLKGVRAIAALRRYFKDQLASGKLKGSDTVLGRLVDN

NEDGKLSDDELFFIAMLLLFAGNETTTNLIGGMFDTLAHAPDQFAMIRDD

PDLIPSAVEEQLRYSAPIQNLYRYTRTDYRVGEVTIPSGSRLLLAFGAAN

RDPEVFEDPDTYRADRNPRNHIAFGYGVHMCIGATLSRMEGQAVLRELTS

QASAIAAAGSATWSTNSSLRGTTYLPIRLTPAR

>CYP128B4 (2802309376) Mycobacteroides abscessus abscessus 205

MSRWRSDSAVRQIGAPVVAAVGMNIAAAVRVRRRGYAGWTGAVNTDYDPL

DPATAAQPFDAYRALHAGGRVHYNPKRATFILSRHEDIRAALRDTDAVTS

SQGVTRMKISAPILVLTDGDDHTRLRKQVQPGFTRGAMSDWQGMADQLAK

ELVADVVANPGCDVMERLAVPLPIRMIAHIIGIPPEDVQNFRSWSEDGVG

VINAGVSPAGLRQGLKGVRAIAALRRYFKDQLASGKLKGSDTVLGRLVDN

NEDGKLSDDELFFIAMLLLFAGNETTTNLIGGMFDTLAHAPDQFAMIRDD

PDLIPSAVEEQLRYSAPIQNLYRYTRTDYRVGEVTIPSGSRLLLAFGAAN

RDPEVFEDPDTYRADRNPRNHIAFGYGVHMCIGATLSRMEGQAVLRELTS

QASAIAAAGSATWSTNSSLRGTTYLPIRLTPAR

>CYP128A1 (2687869967) Mycobacterium sp. QIA-37

MTLKHDARLVYTTTKPVVRSLITNAATEMRAKVSGRRFPGVQETAFDPMD

PETAANPYPGYRTLLAGARVHYNRKRNAFILCRYEDVRAAARNDALLSNR

DGVVRARFEVPVLLNMDRPRHTELRRKALPGFTRGALEGWAPTVDRMAAE

LVTGLLDDPGSDVVEHLAVPLPMRMIAHILGIPPEDEAFFRHWSNESVRV

ANVEFSPKGLRQVPGTLNGVRHLHDYFMTQLGKGNLLGADTVLGKLVVDA

GEGQISHDELFYFALLLLLAGNETTTNLLSTMFLTMSENPDQFELIRSDP

RLLAGAVEEQLRYSSPIQNFYRTAAQDYPVGDAVIPAGARVALLWGAANR

DPREFDDPDRFLATRPVTQHVAFGSGVHLCLGAGLARMEGQAVLRELVNR

VQRVEIEGTPRWTTNSSLRGLEELRVRLVAR

>CYP128B4 (2810644007) Mycobacterium asiaticum 1081914.2

LTVLASVSRNARAAGSVVKPFVESARLNLSTAVNQRRLGASVYAGAELTD

YDPLDPRVAAQPFAAYRKLHAGGRVHYNRRRRTWILHRHSDVRAALTDTD

KINSTQGVTRMKFSMPLIVMTDGEQHAQLRRKVQPAFTKRAMDTWNPMID

ELARTLVAEVLANPGCDVVEKLAIPMPIRMIAHILGVPDGDTAKFRRASD

NAIHIIDFAPNAAAMARCLRGVGGVMRLRRYFMDQFAAGRLKGSNTALGR

LVDGNADGAMTDDDLFFIAMLLLIAGNETTTNVLGGMFDTFARNPEQYDR

IRQDPTLIPMAVEEQLRYSTPIQNLYRYTVADYRVGDVTIPAGARVMLSF

AAANRDPLVFESPDEYRVDRNPRTHVAFGYGAHMCLGAPLARLEAQAVLR

ELVANVSRISPAGEVHWSTNSSLRGPTRLPVRLTRA

>CYP128B1 (2810639960) Mycobacterium asiaticum 1081914.2

MAITETARSVVDIFQPFVSAVRLNAAAAIRTRRRGYAGWTGAVNTDYDPM

DPVTAAQPFDAYRALHRSGRVHYNAKRATFILNRLADVKAALRDTEQVTS

TQGVTRLKFSAPLAVLTDGEEHARLRKQVQPGFSKGAMDLWRGMTEKLAI

ELVSDVLAHPGCDVVQQLAIPMPIRLIAHILGVPECDVGEFRRWSEDAVR

VMDFSLTARGLTGTARSMAAMTALRRYFLQQFSAGGLKDSSTVLGKLLEH

NTDGSLTDKQLFLIAIHLLIAGNETTTNLLGGMFDTLARNPQQYDMIRAD

PDLIPMAVEEQLRITTPIQNLYRYTRADYRVGDVTIPNGSRILLSFGAAN

RDPLAFDNPDEYRADRNPRTHVAFGYGAHMCLGAPLARMEAQAVLRELVT

HVSRISAVGKTIWSSHSSLRGPTHLPINLIPA

>CYP128A1 (2641312290) Mycobacterium bovis MB3

VLVSSEVDGERLSDDELVMETLLILIGGDETTRHTLSGGTEQLLRNRDQW

DLLQRDPSLLPGAIEEMLRWTAPVKNMCRVLTADTEFHGTALCAGEKMML

LFESANFDEAVFCEPEKFDVQRNPNSHLAFGFGTHFCLGNQLARLELSLM

TERVLRRLPDLRLVADDSVLPLRPANFVSGLESMPVVFTPSPPLG

>CYP128A1 (2575619861) Mycobacterium tuberculosis OFXR-

MTATQSPPEPAPDRVRLAGCPLAGTPDVGLTAQDATTALGVPTRRRASSG

GIPVATSMWRDAQTVRTYGPAVAKALALRVAGKARSRLTGRHCRKFMQLT

DFDPFDPAIAADPYPHYRELLAGERVQYNPKRDVYILSRYADVREAARNH

DTLSSARGVTFSRGWLPFLPTSDPPAHTRMRKQLAPGMARGALETWRPMV

DQLARELVGGLLTQTPADVVSTVAAPMPMRAITSVLGVDGPDEAAFCRLS

NQAVRITDVALSASGLISLVQGFAGFRRLRALFTHRRDNGLLRECTVLGK

LATHAEQGRLSDDELFFFAVLLLVAGYESTAHMISTLFLTLADYPDQLTL

LAQQPDLIPSAIEEHLRFISPIQNICRTTRVDYSVGQAVIPAGSLVLLAW

GAANRDPRQYEDPDVFRADRNPVGHLAFGSGIHLCPGTQLARMEGQAILR

EIVANIDRIEVVEPPTWTTNANLRGLTRLRVAVTPRVAP

>CYP128A1 (2576882890) Mycobacterium tuberculosis TB_RSA163

MTATQSPPEPAPDRVRLAGCPLAGTPDVGLTAQDATTALGVPTRRRASSG

GIPVATSMWRDAQTVRTYGPAVAKALALRVAGKARSRLTGRHCRKFMQLT

DFDPFDPAIAADPYPHYRELLAGERVQYNPKRDVYILSRYADVREAARNH

DTLSSARGVTFSRGWLPFLPTSDPPAHTRMRKQLAPGMARGALETWRPMV

DQLARELVGGLLTQTPADVVSTVAAPMPMRAITSVLGVDGPDEAAFCRLS

NQAVRITDVALSASGLISLVQGFAGFRRLRALFTHRRDNGLLRECTVLGK

LATHAEQGRLSDDELFFFAVLLLVAGYESTAHMISTLFLTLADYPDQLTL

LAQQPDLIPSAIEEHLRFISPIQNICRTTRVDYSVGQAVIPAGSLVLLAW

GAANRDPRQYEDPDVFRADRNPVGHLAFGSGIHLCPGTQLARMEGQAILR

EIVANIDRIEVVEPPTWTTNANLRGLTRLRVAVTPRVAP

>CYP128A1 (2577922933) Mycobacterium tuberculosis NRITLD14

MTATQSPPEPAPDRVRLAGCPLAGTPDVGLTAQDATTALGVPTRRRASSG

GIPVATSMWRDAQTVRTYGPAVAKALALRVAGKARSRLTGRHCRKFMQLT

DFDPFDPAIAADPYPHYRELLAGERVQYNPKRDVYILSRYADVREAARNH

DTLSSARGVTFSRGWLPFLPTSDPPAHTRMRKQLAPGMARGALETWRPMV

DQLARELVGGLLTQTPADVVSTVAAPMPMRAITSVLGVDGPDEAAFCRLS

NQAVRITDVALSASGLISLVQGFAGFRRLRALFTHRRDNGLLRECTVLGK

LATHAEQGRLSDDELFFFAVLLLVAGYESTAHMISTLFLTLADYPDQLTL

LAQQPDLIPSAIEEHLRFISPIQNICRTTRVDYSVGQAVIPAGSLVLLAW

GAANRDPRQYEDPDVFRADRNPVGHLAFGSGIHLCPGTQLARMEGQAILR

EIVANIDRIEVVEPPTWTTNANLRGLTRLRVAVTPRVAP

>CYP128A1 (2621440358) Mycobacterium tuberculosis XTB13-128

MTATQSPPEPAPDRVRLAGCPLAGTPDVGLTAQDATTALGVPTRRRASSG

GIPVATSMWRDAQTVRTYGPAVAKALALRVAGKARSRLTGRHCRKFMQLT

DFDPFDPAIAADPYPHYRELLAGERVQYNPKRDVYILSRYADVREAARNH

DTLSSARGVTFSRGWLPFLPTSDPPAHTRMRKQLAPGMARGALETWRPMV

DQLARELVGGLLTQTPADVVSTVAAPMPMRAITSVLGVDGPDEAAFCRLS

NQAVRITDVALSASGLISLVQGFAGFRRLRALFTHRRDNGLLRECTVLGK

LATHAEQGRLSDDELFFFAVLLLVAGYESTAHMISTLFLTLADYPDQLTL

LAQQPDLIPSAIEEHLRFISPIQNICRTTRVDYSVGQAVIPAGSLVLLAW

GAANRDPRQYEDPDVFRADRNPVGHLAFGSGIHLCPGTQLARMEGQAILR

EIVANIDRIEVVEPPTWTTNANLRGLTRLRVAVTPRVAP

>CYP128A1 (2621932872) Mycobacterium tuberculosis TKK_05SA_0044

MTATQSPPEPAPDRVRLAGCPLAGTPDVGLTAQDATTALGVPTRRRASSG

GIPVATSMWRDAQTVRTYGPAVAKALALRVAGKARSRLTGRHCRKFMQLT

DFDPFDPAIAADPYPHYRELLAGERVQYNPKRDVYILSRYADVREAARNH

DTLSSARGVTFSRGWLPFLPTSDPPAHTRMRKQLAPGMARGALETWRPMV

DQLARELVGGLLTQTPADVVSTVAAPMPMRAITSVLGVDGPDEAAFCRLS

NQAVRITDVALSASGLISLVQGFAGFRRLRALFTHRRDNGLLRECTVLGK

LATHAEQGRLSDDELFFFAVLLLVAGYESTAHMISTLFLTLADYPDQLTL

LAQQPDLIPSAIEEHLRFISPIQNICRTTRVDYSVGQAVIPAGSLVLLAW

GAANRDPRQYEDPDVFRADRNPVGHLAFGSGIHLCPGTQLARMEGQAILR

EIVANIDRIEVVEPPTWTTNANLRGLTRLRVAVTPRVAP

>CYP128A1 (2622680465) Mycobacterium tuberculosis M1929

MTATQSPPEPAPDRVRLAGCPLAGTPDVGLTAQDATTALGVPTRRRASSG

GIPVATSMWRDAQTVRTYGPAVAKALALRVAGKARSRLTGRHCRKFMQLT

DFDPFDPAIAADPYPHYRELLAGERVQYNPKRDVYILSRYADVREAARNH

DTLSSARGVTFSRGWLPFLPTSDPPAHTRMRKQLAPGMARGALETWRPMV

DQLARELVGGLLTQTPADVVSTVAAPMPMRAITSVLGVDGPDEAAFCRLS

NQAVRITDVALSASGLISLVQGFAGFRRLRALFTHRRDNGLLRECTVLGK

LATHAEQGRLSDDELFFFAVLLLVAGYESTAHMISTLFLTLADYPDQLTL

LAQQPDLIPSAIEEHLRFISPIQNICRTTRVDYSVGQAVIPAGSLVLLAW

GAANRDPRQYEDPDVFRADRNPVGHLAFGSGIHLCPGTQLARMEGQAILR

EIVANIDRIEVVEPPTWTTNANLRGLTRLRVAVTPRVAP

>CYP128A1 (2630525347) Mycobacterium tuberculosis 2542MS

MTATQSPPEPAPDRVRLAGCPLAGTPDVGLTAQDATTALGVPTRRRASSG

GIPVATSMWRDAQTVRTYGPAVAKALALRVAGKARSRLTGRHCRKFMQLT

DFDPFDPAIAADPYPHYRELLAGERVQYNPKRDVYILSRYADVREAARNH

DTLSSARGVTFSRGWLPFLPTSDPPAHTRMRKQLAPGMARGALETWRPMV

DQLARELVGGLLTQTPADVVSTVAAPMPMRAITSVLGVDGPDEAAFCRLS

NQAVRITDVALSASGLISLVQGFAGFRRLRALFTHRRDNGLLRECTVLGK

LATHAEQGRLSDDELFFFAVLLLVAGYESTAHMISTLFLTLADYPDQLTL

LAQQPDLIPSAIEEHLRFISPIQNICRTTRVDYSVGQAVIPAGSLVLLAW

GAANRDPRQYEDPDVFRADRNPVGHLAFGSGIHLCPGTQLARMEGQAILR

EIVANIDRIEVVEPPTWTTNANLRGLTRLRVAVTPRVAP

>CYP128A1 (2641311494) Mycobacterium bovis MB3

MTATQSPPEPAPDRVRLAGCPLAGTPDVGLTAQDATTALGVPTRRRASSG

GIPVATSMWRDAQTVRTYGPAVAKALALRVAGKARSRLTGRHCRKFMQLT

DFDPFDPAIAADPYPHYRELLAGERVQYNPKRDVYILSRYADVREAARNH

DTLSSARGVTFSRGWLPFLPTSDPPAHTRMRKQLAPGMARGALETWRPMV

DQLARELVGGLLTQTPADVVSTVAAPMPMRAITSVLGVDGPDEAAFCRLS

NQAVRITDVALSASGLISLVQGFAGFRRLRALFTHRRDNGLLRECTVLGK

LATHAEQGRLSDDELFFFAVLLLVAGYESTAHMISTLFLTLADYPDQLTL

LAQQPDLIPSAIEEHLRFISPIQNICRTTRVDYSVGQAVIPAGSLVLLAW

GAANRDPRQYEDPDVFRADRNPVGHLAFGSGIHLCPGTQLARMEGQAILR

EIVANIDRIEVVEPPTWTTNANLRGLTRLRVAVTPRVAP

>CYP128A1 (2673862062) Mycobacterium tuberculosis A70441

MTATQSPPEPAPDRVRLAGCPLAGTPDVGLTAQDATTALGVPTRRRASSG

GIPVATSMWRDAQTVRTYGPAVAKALALRVAGKARSRLTGRHCRKFMQLT

DFDPFDPAIAADPYPHYRELLAGERVQYNPKRDVYILSRYADVREAARNH

DTLSSARGVTFSRGWLPFLPTSDPPAHTRMRKQLAPGMARGALETWRPMV

DQLARELVGGLLTQTPADVVSTVAAPMPMRAITSVLGVDGPDEAAFCRLS

NQAVRITDVALSASGLISLVQGFAGFRRLRALFTHRRDNGLLRECTVLGK

LATHAEQGRLSDDELFFFAVLLLVAGYESTAHMISTLFLTLADYPDQLTL

LAQQPDLIPSAIEEHLRFISPIQNICRTTRVDYSVGQAVIPAGSLVLLAW

GAANRDPRQYEDPDVFRADRNPVGHLAFGSGIHLCPGTQLARMEGQAILR

EIVANIDRIEVVEPPTWTTNANLRGLTRLRVAVTPRVAP

>CYP128A1 (2692178503) Mycobacterium tuberculosis O00100977

MTATQSPPEPAPDRVRLAGCPLAGTPDVGLTAQDATTALGVPTRRRASSG

GIPVATSMWRDAQTVRTYGPAVAKALALRVAGKARSRLTGRHCRKFMQLT

DFDPFDPAIAADPYPHYRELLAGERVQYNPKRDVYILSRYADVREAARNH

DTLSSARGVTFSRGWLPFLPTSDPPAHTRMRKQLAPGMARGALETWRPMV

DQLARELVGGLLTQTPADVVSTVAAPMPMRAITSVLGVDGPDEAAFCRLS

NQAVRITDVALSASGLISLVQGFAGFRRLRALFTHRRDNGLLRECTVLGK

LATHAEQGRLSDDELFFFAVLLLVAGYESTAHMISTLFLTLADYPDQLTL

LAQQPDLIPSAIEEHLRFISPIQNICRTTRVDYSVGQAVIPAGSLVLLAW

GAANRDPRQYEDPDVFRADRNPVGHLAFGSGIHLCPGTQLARMEGQAILR

EIVANIDRIEVVEPPTWTTNANLRGLTRLRVAVTPRVAP

>CYP128A1 (2716354004) Mycobacterium tuberculosis H_13559

MTATQSPPEPAPDRVRLAGCPLAGTPDVGLTAQDATTALGVPTRRRASSG

GIPVATSMWRDAQTVRTYGPAVAKALALRVAGKARSRLTGRHCRKFMQLT

DFDPFDPAIAADPYPHYRELLAGERVQYNPKRDVYILSRYADVREAARNH

DTLSSARGVTFSRGWLPFLPTSDPPAHTRMRKQLAPGMARGALETWRPMV

DQLARELVGGLLTQTPADVVSTVAAPMPMRAITSVLGVDGPDEAAFCRLS

NQAVRITDVALSASGLISLVQGFAGFRRLRALFTHRRDNGLLRECTVLGK

LATHAEQGRLSDDELFFFAVLLLVAGYESTAHMISTLFLTLADYPDQLTL

LAQQPDLIPSAIEEHLRFISPIQNICRTTRVDYSVGQAVIPAGSLVLLAW

GAANRDPRQYEDPDVFRADRNPVGHLAFGSGIHLCPGTQLARMEGQAILR

EIVANIDRIEVVEPPTWTTNANLRGLTRLRVAVTPRVAP

>CYP128B4 (2797630912) Mycobacteroides abscessus abscessus 9

MSRWRSDSAVRQIGAPVVAAVGMNIAAAVRVRRRGYAGWTGAVNTDYDPL

DPATAAQPFDAYRALHAGGRVHYNPKRATFILSRHEDIRAALRDTDAVTS

SQGVTRMKISAPILVLTDGDDHTRLRKQVQPGFTRGAMSDWQGMADQLAK

ELVADVVANPGCDVMERLAVPLPIRMIAHIIGIPPEDVQNFRSWSEDGVG

VINAGVSPAGLRQGLKGVRAIAALRRYFKDQLASGKLKGSDTVLGRLVDN

NEDGKLSDDELFFIAMLLLFAGNETTTNLIGGMFDTLAHAPDQFAMIRDD

PDLIPSAVEEQLRYSAPIQNLYRYTRTDYRVGEVTIPSGSRLLLAFGAAN

RDPEVFEDPDTYRADRNPRNHIAFGYGVHMCIGATLSRMEGQAVLRELTS

QASAIAAAGSATWSTNSSLRGTTYLPIRLTPAR

>CYP128B4 (2803358721) Mycobacteroides abscessus abscessus 527

MSRWRSDSAVRQIGAPVVAAVGMNIAAAVRVRRRGYAGWTGAVNTDYDPL

DPATAAQPFDAYRALHAGGRVHYNPKRATFILSRHEDIRAALRDTDAVTS

SQGVTRMKISAPILVLTDGDDHTRLRKQVQPGFTRGAMSDWQGMADQLAK

ELVADVVANPGCDVMERLAVPLPIRMIAHIIGIPPEDVQNFRSWSEDGVG

VINAGVSPAGLRQGLKGVRAIAALRRYFKDQLASGKLKGSDTVLGRLVDN

NEDGKLSDDELFFIAMLLLFAGNETTTNLIGGMFDTLAHAPDQFAMIRDD

PDLIPSAVEEQLRYSAPIQNLYRYTRTDYRVGEVTIPSGSRLLLAFGAAN

RDPEVFEDPDTYRADRNPRNHIAFGYGVHMCIGATLSRMEGQAVLRELTS

QASAIAAAGSATWSTNSSLRGTTYLPIRLTPAR

>CYP128A1 (2575016813) Mycobacterium tuberculosis BTB12-001

MTATQSPPEPAPDRVRLAGCPLAGTPDVGLTAQDATTALGVPTRRRASSG

GIPVATSMWRDAQTVRTYGPAVAKALALRVAGKARSRLTGRHCRKFMQLT

DFDPFDPAIAADPYPHYRELLAGERVQYNPKRDVYILSRYADVREAARNH

DTLSSARGVTFSRGWLPFLPTSDPPAHTRMRKQLAPGMARGALETWRPMV

DQLARELVGGLLTQTPADVVSTVAAPMPMRAITSVLGVDGPDEAAFCRLS

NQAVRITDVALSASGLISLVQGFAGFRRLRALFTHRRDNGLLRECTVLGK

LATHAEQGRLSDDELFFFAVLLLVAGYESTAHMISTLFLTLADYPDQLTL

LAQQPDLIPSAIEEHLRFISPIQNICRTTRVDYSVGQAVIPAGSLVLLAW

GAANRDPRQYEDPDVFRADRNPVGHLAFGSGIHLCPGTQLARMEGQAILR

EIVANIDRIEVVEPPTWTTNANLRGLTRLRVAVTPRVAP

>CYP128A1 (2575427474) Mycobacterium tuberculosis TB_RSA118

MTATQSPPEPAPDRVRLAGCPLAGTPDVGLTAQDATTALGVPTRRRASSG

GIPVATSMWRDAQTVRTYGPAVAKALALRVAGKARSRLTGRHCRKFMQLT

DFDPFDPAIAADPYPHYRELLAGERVQYNPKRDVYILSRYADVREAARNH

DTLSSARGVTFSRGWLPFLPTSDPPAHTRMRKQLAPGMARGALETWRPMV

DQLARELVGGLLTQTPADVVSTVAAPMPMRAITSVLGVDGPDEAAFCRLS

NQAVRITDVALSASGLISLVQGFAGFRRLRALFTHRRDNGLLRECTVLGK

LATHAEQGRLSDDELFFFAVLLLVAGYESTAHMISTLFLTLADYPDQLTL

LAQQPDLIPSAIEEHLRFISPIQNICRTTRVDYSVGQAVIPAGSLVLLAW

GAANRDPRQYEDPDVFRADRNPVGHLAFGSGIHLCPGTQLARMEGQAILR

EIVANIDRIEVVEPPTWTTNANLRGLTRLRVAVTPRVAP

>CYP128A1 (2577470915) Mycobacterium tuberculosis NRITLD56

MTATQSPPEPAPDRVRLAGCPLAGTPDVGLTAQDATTALGVPTRRRASSG

GIPVATSMWRDAQTVRTYGPAVAKALALRVAGKARSRLTGRHCRKFMQLT

DFDPFDPAIAADPYPHYRELLAGERVQYNPKRDVYILSRYADVREAARNH

DTLSSARGVTFSRGWLPFLPTSDPPAHTRMRKQLAPGMARGALETWRPMV

DQLARELVGGLLTQTPADVVSTVAAPMPMRAITSVLGVDGPDEAAFCRLS

NQAVRITDVALSASGLISLVQGFAGFRRLRALFTHRRDNGLLRECTVLGK

LATHAEQGRLSDDELFFFAVLLLVAGYESTAHMISTLFLTLADYPDQLTL

LAQQPDLIPSAIEEHLRFISPIQNICRTTRVDYSVGQAVIPAGSLVLLAW

GAANRDPRQYEDPDVFRADRNPVGHLAFGSGIHLCPGTQLARMEGQAILR

EIVANIDRIEVVEPPTWTTNANLRGLTRLRVAVTPRVAP

>CYP128A1 (2584837588) Mycobacterium tuberculosis TKK_04_0038

MTATQSPPEPAPDRVRLAGCPLAGTPDVGLTAQDATTALGVPTRRRASSG

GIPVATSMWRDAQTVRTYGPAVAKALALRVAGKARSRLTGRHCRKFMQLT

DFDPFDPAIAADPYPHYRELLAGERVQYNPKRDVYILSRYADVREAARNH

DTLSSARGVTFSRGWLPFLPTSDPPAHTRMRKQLAPGMARGALETWRPMV

DQLARELVGGLLTQTPADVVSTVAAPMPMRAITSVLGVDGPDEAAFCRLS

NQAVRITDVALSASGLISLVQGFAGFRRLRALFTHRRDNGLLRECTVLGK

LATHAEQGRLSDDELFFFAVLLLVAGYESTAHMISTLFLTLADYPDQLTL

LAQQPDLIPSAIEEHLRFISPIQNICRTTRVDYSVGQAVIPAGSLVLLAW

GAANRDPRQYEDPDVFRADRNPVGHLAFGSGIHLCPGTQLARMEGQAILR

EIVANIDRIEVVEPPTWTTNANLRGLTRLRVAVTPRVAP

>CYP128A1 (2589592713) Mycobacterium tuberculosis TKK-01-0034

MTATQSPPEPAPDRVRLAGCPLAGTPDVGLTAQDATTALGVPTRRRASSG

GIPVATSMWRDAQTVRTYGPAVAKALALRVAGKARSRLTGRHCRKFMQLT

DFDPFDPAIAADPYPHYRELLAGERVQYNPKRDVYILSRYADVREAARNH

DTLSSARGVTFSRGWLPFLPTSDPPAHTRMRKQLAPGMARGALETWRPMV

DQLARELVGGLLTQTPADVVSTVAAPMPMRAITSVLGVDGPDEAAFCRLS

NQAVRITDVALSASGLISLVQGFAGFRRLRALFTHRRDNGLLRECTVLGK

LATHAEQGRLSDDELFFFAVLLLVAGYESTAHMISTLFLTLADYPDQLTL

LAQQPDLIPSAIEEHLRFISPIQNICRTTRVDYSVGQAVIPAGSLVLLAW

GAANRDPRQYEDPDVFRADRNPVGHLAFGSGIHLCPGTQLARMEGQAILR

EIVANIDRIEVVEPPTWTTNANLRGLTRLRVAVTPRVAP

>CYP128A1 (2592353648) Mycobacterium tuberculosis TKK_02_0067

MTATQSPPEPAPDRVRLAGCPLAGTPDVGLTAQDATTALGVPTRRRASSG

GIPVATSMWRDAQTVRTYGPAVAKALALRVAGKARSRLTGRHCRKFMQLT

DFDPFDPAIAADPYPHYRELLAGERVQYNPKRDVYILSRYADVREAARNH

DTLSSARGVTFSRGWLPFLPTSDPPAHTRMRKQLAPGMARGALETWRPMV

DQLARELVGGLLTQTPADVVSTVAAPMPMRAITSVLGVDGPDEAAFCRLS

NQAVRITDVALSASGLISLVQGFAGFRRLRALFTHRRDNGLLRECTVLGK

LATHAEQGRLSDDELFFFAVLLLVAGYESTAHMISTLFLTLADYPDQLTL

LAQQPDLIPSAIEEHLRFISPIQNICRTTRVDYSVGQAVIPAGSLVLLAW

GAANRDPRQYEDPDVFRADRNPVGHLAFGSGIHLCPGTQLARMEGQAILR

EIVANIDRIEVVEPPTWTTNANLRGLTRLRVAVTPRVAP

>CYP128A1 (2603255014) Mycobacterium tuberculosis TB_RSA152

MTATQSPPEPAPDRVRLAGCPLAGTPDVGLTAQDATTALGVPTRRRASSG

GIPVATSMWRDAQTVRTYGPAVAKALALRVAGKARSRLTGRHCRKFMQLT

DFDPFDPAIAADPYPHYRELLAGERVQYNPKRDVYILSRYADVREAARNH

DTLSSARGVTFSRGWLPFLPTSDPPAHTRMRKQLAPGMARGALETWRPMV

DQLARELVGGLLTQTPADVVSTVAAPMPMRAITSVLGVDGPDEAAFCRLS

NQAVRITDVALSASGLISLVQGFAGFRRLRALFTHRRDNGLLRECTVLGK

LATHAEQGRLSDDELFFFAVLLLVAGYESTAHMISTLFLTLADYPDQLTL

LAQQPDLIPSAIEEHLRFISPIQNICRTTRVDYSVGQAVIPAGSLVLLAW

GAANRDPRQYEDPDVFRADRNPVGHLAFGSGIHLCPGTQLARMEGQAILR

EIVANIDRIEVVEPPTWTTNANLRGLTRLRVAVTPRVAP

>CYP128A1 (2605037845) Mycobacterium tuberculosis TB_RSA72

MTATQSPPEPAPDRVRLAGCPLAGTPDVGLTAQDATTALGVPTRRRASSG

GIPVATSMWRDAQTVRTYGPAVAKALALRVAGKARSRLTGRHCRKFMQLT

DFDPFDPAIAADPYPHYRELLAGERVQYNPKRDVYILSRYADVREAARNH

DTLSSARGVTFSRGWLPFLPTSDPPAHTRMRKQLAPGMARGALETWRPMV

DQLARELVGGLLTQTPADVVSTVAAPMPMRAITSVLGVDGPDEAAFCRLS

NQAVRITDVALSASGLISLVQGFAGFRRLRALFTHRRDNGLLRECTVLGK

LATHAEQGRLSDDELFFFAVLLLVAGYESTAHMISTLFLTLADYPDQLTL

LAQQPDLIPSAIEEHLRFISPIQNICRTTRVDYSVGQAVIPAGSLVLLAW

GAANRDPRQYEDPDVFRADRNPVGHLAFGSGIHLCPGTQLARMEGQAILR

EIVANIDRIEVVEPPTWTTNANLRGLTRLRVAVTPRVAP

>CYP128A1 (2621760964) Mycobacterium tuberculosis 1430BH

MTATQSPPEPAPDRVRLAGCPLAGTPDVGLTAQDATTALGVPTRRRASSG

GIPVATSMWRDAQTVRTYGPAVAKALALRVAGKARSRLTGRHCRKFMQLT

DFDPFDPAIAADPYPHYRELLAGERVQYNPKRDVYILSRYADVREAARNH

DTLSSARGVTFSRGWLPFLPTSDPPAHTRMRKQLAPGMARGALETWRPMV

DQLARELVGGLLTQTPADVVSTVAAPMPMRAITSVLGVDGPDEAAFCRLS

NQAVRITDVALSASGLISLVQGFAGFRRLRALFTHRRDNGLLRECTVLGK

LATHAEQGRLSDDELFFFAVLLLVAGYESTAHMISTLFLTLADYPDQLTL

LAQQPDLIPSAIEEHLRFISPIQNICRTTRVDYSVGQAVIPAGSLVLLAW

GAANRDPRQYEDPDVFRADRNPVGHLAFGSGIHLCPGTQLARMEGQAILR

EIVANIDRIEVVEPPTWTTNANLRGLTRLRVAVTPRVAP

>CYP128A1 (2621982195) Mycobacterium tuberculosis MAL010077

MTATQSPPEPAPDRVRLAGCPLAGTPDVGLTAQDATTALGVPTRRRASSG

GIPVATSMWRDAQTVRTYGPAVAKALALRVAGKARSRLTGRHCRKFMQLT

DFDPFDPAIAADPYPHYRELLAGERVQYNPKRDVYILSRYADVREAARNH

DTLSSARGVTFSRGWLPFLPTSDPPAHTRMRKQLAPGMARGALETWRPMV

DQLARELVGGLLTQTPADVVSTVAAPMPMRAITSVLGVDGPDEAAFCRLS

NQAVRITDVALSASGLISLVQGFAGFRRLRALFTHRRDNGLLRECTVLGK

LATHAEQGRLSDDELFFFAVLLLVAGYESTAHMISTLFLTLADYPDQLTL

LAQQPDLIPSAIEEHLRFISPIQNICRTTRVDYSVGQAVIPAGSLVLLAW

GAANRDPRQYEDPDVFRADRNPVGHLAFGSGIHLCPGTQLARMEGQAILR

EIVANIDRIEVVEPPTWTTNANLRGLTRLRVAVTPRVAP

>CYP128A1 (2622197683) Mycobacterium tuberculosis MD14621

MTATQSPPEPAPDRVRLAGCPLAGTPDVGLTAQDATTALGVPTRRRASSG

GIPVATSMWRDAQTVRTYGPAVAKALALRVAGKARSRLTGRHCRKFMQLT

DFDPFDPAIAADPYPHYRELLAGERVQYNPKRDVYILSRYADVREAARNH

DTLSSARGVTFSRGWLPFLPTSDPPAHTRMRKQLAPGMARGALETWRPMV

DQLARELVGGLLTQTPADVVSTVAAPMPMRAITSVLGVDGPDEAAFCRLS

NQAVRITDVALSASGLISLVQGFAGFRRLRALFTHRRDNGLLRECTVLGK

LATHAEQGRLSDDELFFFAVLLLVAGYESTAHMISTLFLTLADYPDQLTL

LAQQPDLIPSAIEEHLRFISPIQNICRTTRVDYSVGQAVIPAGSLVLLAW

GAANRDPRQYEDPDVFRADRNPVGHLAFGSGIHLCPGTQLARMEGQAILR

EIVANIDRIEVVEPPTWTTNANLRGLTRLRVAVTPRVAP

>CYP128A1 (2622614329) Mycobacterium tuberculosis M1615

MTATQSPPEPAPDRVRLAGCPLAGTPDVGLTAQDATTALGVPTRRRASSG

GIPVATSMWRDAQTVRTYGPAVAKALALRVAGKARSRLTGRHCRKFMQLT

DFDPFDPAIAADPYPHYRELLAGERVQYNPKRDVYILSRYADVREAARNH

DTLSSARGVTFSRGWLPFLPTSDPPAHTRMRKQLAPGMARGALETWRPMV

DQLARELVGGLLTQTPADVVSTVAAPMPMRAITSVLGVDGPDEAAFCRLS

NQAVRITDVALSASGLISLVQGFAGFRRLRALFTHRRDNGLLRECTVLGK

LATHAEQGRLSDDELFFFAVLLLVAGYESTAHMISTLFLTLADYPDQLTL

LAQQPDLIPSAIEEHLRFISPIQNICRTTRVDYSVGQAVIPAGSLVLLAW

GAANRDPRQYEDPDVFRADRNPVGHLAFGSGIHLCPGTQLARMEGQAILR

EIVANIDRIEVVEPPTWTTNANLRGLTRLRVAVTPRVAP

>CYP128A1 (2647411139) Mycobacterium tuberculosis H2375

MTATQSPPEPAPDRVRLAGCPLAGTPDVGLTAQDATTALGVPTRRRASSG

GIPVATSMWRDAQTVRTYGPAVAKALALRVAGKARSRLTGRHCRKFMQLT

DFDPFDPAIAADPYPHYRELLAGERVQYNPKRDVYILSRYADVREAARNH

DTLSSARGVTFSRGWLPFLPTSDPPAHTRMRKQLAPGMARGALETWRPMV

DQLARELVGGLLTQTPADVVSTVAAPMPMRAITSVLGVDGPDEAAFCRLS

NQAVRITDVALSASGLISLVQGFAGFRRLRALFTHRRDNGLLRECTVLGK

LATHAEQGRLSDDELFFFAVLLLVAGYESTAHMISTLFLTLADYPDQLTL

LAQQPDLIPSAIEEHLRFISPIQNICRTTRVDYSVGQAVIPAGSLVLLAW

GAANRDPRQYEDPDVFRADRNPVGHLAFGSGIHLCPGTQLARMEGQAILR

EIVANIDRIEVVEPPTWTTNANLRGLTRLRVAVTPRVAP

>CYP128A1 (2687242989) Mycobacterium africanum UT307

MTATQSPPEPAPDRVRLAGCPLAGTPDVGLTAQDATTALGVPTRRRASSG

GIPVATSMWRDAQTVRTYGPAVAKALALRVAGKARSRLTGRHCRKFMQLT

DFDPFDPAIAADPYPHYRELLAGERVQYNPKRDVYILSRYADVREAARNH

DTLSSARGVTFSRGWLPFLPTSDPPAHTRMRKQLAPGMARGALETWRPMV

DQLARELVGGLLTQTPADVVSTVAAPMPMRAITSVLGVDGPDEAAFCRLS

NQAVRITDVALSASGLISLVQGFAGFRRLRALFTHRRDNGLLRECTVLGK

LATHAEQGRLSDDELFFFAVLLLVAGYESTAHMISTLFLTLADYPDQLTL

LAQQPDLIPSAIEEHLRFISPIQNICRTTRVDYSVGQAVIPAGSLVLLAW

GAANRDPRQYEDPDVFRADRNPVGHLAFGSGIHLCPGTQLARMEGQAILR

EIVANIDRIEVVEPPTWTTNANLRGLTRLRVAVTPRVAP

>CYP128A1 (637027493) Mycobacterium tuberculosis H37Rv

MTATQSPPEPAPDRVRLAGCPLAGTPDVGLTAQDATTALGVPTRRRASSG

GIPVATSMWRDAQTVRTYGPAVAKALALRVAGKARSRLTGRHCRKFMQLT

DFDPFDPAIAADPYPHYRELLAGERVQYNPKRDVYILSRYADVREAARNH

DTLSSARGVTFSRGWLPFLPTSDPPAHTRMRKQLAPGMARGALETWRPMV

DQLARELVGGLLTQTPADVVSTVAAPMPMRAITSVLGVDGPDEAAFCRLS

NQAVRITDVALSASGLISLVQGFAGFRRLRALFTHRRDNGLLRECTVLGK

LATHAEQGRLSDDELFFFAVLLLVAGYESTAHMISTLFLTLADYPDQLTL

LAQQPDLIPSAIEEHLRFISPIQNICRTTRVDYSVGQAVIPAGSLVLLAW

GAANRDPRQYEDPDVFRADRNPVGHLAFGSGIHLCPGTQLARMEGQAILR

EIVANIDRIEVVEPPTWTTNANLRGLTRLRVAVTPRVAP

>CYP128A1 (2696812326) Mycobacterium tuberculosis A70448

MTATQSPPEPAPDRVRLAGCPLAGTPDVGLTAQDATTALGVPTRRRASSG

GIPVATSMWRDAQTVRTYGPAVAKALALRVAGKARSRLTGRHCRKFMQLT

DFDPFDPAIAADPYPHYRELLAGERVQDNPKRDVYILSRYADVREAARNH

DTLSSARGVTFSRGWLPFLPTSDPPAHTRMRKQLAPGMARGALETWRPMV

DQLARELVGGLLTQTPADVVSTVAAPMPMRAITSVLGVDGPDEAAFCRLS

NQAVRITDVALSASGLISLVQGFAGFRRLRALFTHRRDNGLLRECTVLGK

LATHAEQGRLSDDELFFFAVLLLVAGYESTAHMISTLFLTLADYPDQLTL

LAQQPDLIPSAIEEHLRFISPIQNICRTTRVDYSVGQAVIPAGSLVLLAW

GAANRDPRQYEDPDVFRADRNPVGHLAFGSGIHLCPGTQLARMEGQAILR

EIVANIDRIEVVEPPTWTTNANLRGLTRLRVAVTPRVAP

>CYP128A1 (2724795061) Mycobacterium tuberculosis BE_116771

ALGVPTRRRASSGGIPVATSMWRDAQTVRTYGPAVAKALALRVAGKARSR

LTGRHCRKFMQLTDFDPFDPAIAADPYPHYRELLAGERVQYNPKRDVYIL

SRYADVREAARNHDTLSSARGVTFSRGWLPFLPTSDPPAHTRMRKQLAPG

MARGALETWRPMVDQLARELVGGLLTQTPADVVSTVAAPMPMRAITSVLG

VDGPDEAAFCRLSNQAVRITDVALSASGLISLVQGFAGFRRLRALFTHRR

DNGLLRECTVLGKLATHAEQGRLSDDELFFFAVLLLVAGYESTAHMISTL

FLTLADYPDQLTLLAQQPDLIPSAIEEHLRFISPIQNICRTTRVDYSVGQ

AVIPAGSLVLLAWGAANRDPRQYEDPDVFRADRNPVGHLAFGSGIHLCPG

TQLARMEGQAILREIVANIDRIEVVEPPTWTTNANLRGLTRLRVAVTPRV

AP

>CYP128B4 (2801628407) Mycobacteroides abscessus abscessus 394

MSRWRSDSAVRQIGAPVVAAVGMNIAAAVRVRRRGYAGWTGAVNTDYDPL

DPATAAQPFDAYRALHAGGRVHYNPKRATFILSRHEDIRAALRDTDAVTS

SQGVTRMKISAPILVLTDGDDHTRLRKQVQPGFTRGAMSDWQGMADQLAK

ELVADVVANPGCDVMERLAVPLPIRMIAHIIGIPPEDVQNFRSWSEDGVG

VINAGVSPAGLRQGLKGVRAIAALRRYFKDQLASGKLKGSDTVLGRLVDN

NEDGKLSDDELFFIAMLLLFAGNETTTNLIGGMFDTLAHAPDQFAMIRDD

PDLIPSAVEEQLRYSAPIQNLYRYTRTDYRVGEVTIPSGSRLLLAFGAAN

RDPEVFEDPDTYRADRNPRNHIAFGYGVHMCIGATLSRMEGQAVLRELTS

QASAIAAAGSATWSTNSSLRGTTYLPIRLTPAR

>CYP128A1 (2688704869) Mycobacterium immunogenum CCUG 47286

MTLKHDARLVRTTTKPVVRSLISNASDELRAKVRRRTFAGVQETAFDPMD

PQTAANPHAGYRELLAGGRVHYNRKRNIFILCRYEDVRAAARNDALLSNR

DGVVRARFEVPVLLNMDRPRHTELRRKALPGFTRGALDGWAPTVDRLAAA

LVTGLLDTPEADVVEHLAVPLPMQMIAHILGIPPEDEAFFRHWSNESVRV

ANVEFSAKGLMQVPGTLNGVRHLHDYFMTQLGRGNLLGSDTMLGRLVADA

GEGQISHDELFYFALLLLLAGNETTTNLLSTMFLTLSENPEQFELIRADP

ALIAGAVEEQLRYSSPIQNFYRTATKDYVVGGAVIPAGSRVALLWGAANR

DPREFDDPDRFLAARPVSQHVAFGSGVHLCLGAGLARMEGQAVLRELVNR

VQRIEIADTPRWTTNSSLRGLERLRVRLVAR

>CYP128B2 (2507119127) Mycobacterium smegmatis JS623

MKLGRAADGAASMFSPLIAAGRLNVSAAIRTHRRGYSGWTGAVNTGYDPL

DPAVAAQPHAAYDALHRSGRVHYNPRRATWILHRLDDVRAALRDTDRVTS

SHGVTRVRMVADLVVVTDGEQHSRLRKQVQPAFTKGALDSWRTIIDKLAA

ELVDDMIAAGNCDAVERLTMPMPLRVIAAILGVPESDIADFRRWSDDSTQ

LINFTPTFSGVVSTARSMRAAVALHRYFLKHLADGELKGSDTVLGRLLEH

NTDGALTDNQLFYIAILLLIAGNETTTNLLGGMLHTYAEHPDQYDMIRAN

PDLIPQAIEEHVRYTSPIQNLYRYTRADYQVGEVTIPSGSRVLMSFGAAN

RDPLAFDDPNTFRADRNPRSHVGFGYGPHLCLGAPLARMEAQAVLRELVD

RVSRIETTGPTTWSTNSSLRGPTQLPVILHEGSTP

>CYP128A1 (2555153346) Mycobacterium tuberculosis PanR0208

MTATQSPPEPAPDRVRLAGCPLAGTPDVGLTAQDATTALGVPTRRRASSG

GIPVATSMWRDAQTVRTYGPAVAKALALRVAGKARSRLTGRHCRKFMQLT

DFDPFDPAIAADPYPHYRELLAGERVQYNPKRDVYILSRYADVREAARNH

DTLSSARGVTFSRGWLPFLPTSDPPAHTRMRKQLAPGMARGALETWRPMV

DQLARELVGGLLTQTPADVVSTVAAPMPMRAITSVLGVDGPDEAAFCRLS

NQAVRITDVALSASGLISLVQGFAGFRRLRALFTHRRDNGLLRECTVLGK

LATHAEQGRLSDDELFFFAVLLLVAGYESTAHMISTLFLTLADYPDQLTL

LAQQPDLIPSAIEEHLRFISPIQNICRTTRVDYSVGQAVIPAGSLVLLAW

GAANRDPRQYEDPDVFRADRNPVGHLAFGSGIHLCPGTQLARMEGQAILR

EIVANIDRIEVVEPPTWTTNANLRGLTRLRVAVTPRVAP

>CYP128A1 (2577975523) Mycobacterium tuberculosis XTB13-214

MTATQSPPEPAPDRVRLAGCPLAGTPDVGLTAQDATTALGVPTRRRASSG

GIPVATSMWRDAQTVRTYGPAVAKALALRVAGKARSRLTGRHCRKFMQLT

DFDPFDPAIAADPYPHYRELLAGERVQYNPKRDVYILSRYADVREAARNH

DTLSSARGVTFSRGWLPFLPTSDPPAHTRMRKQLAPGMARGALETWRPMV

DQLARELVGGLLTQTPADVVSTVAAPMPMRAITSVLGVDGPDEAAFCRLS

NQAVRITDVALSASGLISLVQGFAGFRRLRALFTHRRDNGLLRECTVLGK

LATHAEQGRLSDDELFFFAVLLLVAGYESTAHMISTLFLTLADYPDQLTL

LAQQPDLIPSAIEEHLRFISPIQNICRTTRVDYSVGQAVIPAGSLVLLAW

GAANRDPRQYEDPDVFRADRNPVGHLAFGSGIHLCPGTQLARMEGQAILR

EIVANIDRIEVVEPPTWTTNANLRGLTRLRVAVTPRVAP

>CYP128A1 (2578010844) Mycobacterium tuberculosis TB_RSA32

MTATQSPPEPAPDRVRLAGCPLAGTPDVGLTAQDATTALGVPTRRRASSG

GIPVATSMWRDAQTVRTYGPAVAKALALRVAGKARSRLTGRHCRKFMQLT

DFDPFDPAIAADPYPHYRELLAGERVQYNPKRDVYILSRYADVREAARNH

DTLSSARGVTFSRGWLPFLPTSDPPAHTRMRKQLAPGMARGALETWRPMV

DQLARELVGGLLTQTPADVVSTVAAPMPMRAITSVLGVDGPDEAAFCRLS

NQAVRITDVALSASGLISLVQGFAGFRRLRALFTHRRDNGLLRECTVLGK

LATHAEQGRLSDDELFFFAVLLLVAGYESTAHMISTLFLTLADYPDQLTL

LAQQPDLIPSAIEEHLRFISPIQNICRTTRVDYSVGQAVIPAGSLVLLAW

GAANRDPRQYEDPDVFRADRNPVGHLAFGSGIHLCPGTQLARMEGQAILR

EIVANIDRIEVVEPPTWTTNANLRGLTRLRVAVTPRVAP

>CYP128A1 (2578181993) Mycobacterium tuberculosis TKK-01-0025

MTATQSPPEPAPDRVRLAGCPLAGTPDVGLTAQDATTALGVPTRRRASSG

GIPVATSMWRDAQTVRTYGPAVAKALALRVAGKARSRLTGRHCRKFMQLT

DFDPFDPAIAADPYPHYRELLAGERVQYNPKRDVYILSRYADVREAARNH

DTLSSARGVTFSRGWLPFLPTSDPPAHTRMRKQLAPGMARGALETWRPMV

DQLARELVGGLLTQTPADVVSTVAAPMPMRAITSVLGVDGPDEAAFCRLS

NQAVRITDVALSASGLISLVQGFAGFRRLRALFTHRRDNGLLRECTVLGK

LATHAEQGRLSDDELFFFAVLLLVAGYESTAHMISTLFLTLADYPDQLTL

LAQQPDLIPSAIEEHLRFISPIQNICRTTRVDYSVGQAVIPAGSLVLLAW

GAANRDPRQYEDPDVFRADRNPVGHLAFGSGIHLCPGTQLARMEGQAILR

EIVANIDRIEVVEPPTWTTNANLRGLTRLRVAVTPRVAP

>CYP128A1 (2580769505) Mycobacterium bovis Mr 4387

MTATQSPPEPAPDRVRLAGCPLAGTPDVGLTAQDATTALGVPTRRRASSG

GIPVATSMWRDAQTVRTYGPAVAKALALRVAGKARSRLTGRHCRKFMQLT

DFDPFDPAIAADPYPHYRELLAGERVQYNPKRDVYILSRYADVREAARNH

DTLSSARGVTFSRGWLPFLPTSDPPAHTRMRKQLAPGMARGALETWRPMV

DQLARELVGGLLTQTPADVVSTVAAPMPMRAITSVLGVDGPDEAAFCRLS

NQAVRITDVALSASGLISLVQGFAGFRRLRALFTHRRDNGLLRECTVLGK

LATHAEQGRLSDDELFFFAVLLLVAGYESTAHMISTLFLTLADYPDQLTL

LAQQPDLIPSAIEEHLRFISPIQNICRTTRVDYSVGQAVIPAGSLVLLAW

GAANRDPRQYEDPDVFRADRNPVGHLAFGSGIHLCPGTQLARMEGQAILR

EIVANIDRIEVVEPPTWTTNANLRGLTRLRVAVTPRVAP

>CYP128A1 (2603957949) Mycobacterium tuberculosis TB_RSA135

MTATQSPPEPAPDRVRLAGCPLAGTPDVGLTAQDATTALGVPTRRRASSG

GIPVATSMWRDAQTVRTYGPAVAKALALRVAGKARSRLTGRHCRKFMQLT

DFDPFDPAIAADPYPHYRELLAGERVQYNPKRDVYILSRYADVREAARNH

DTLSSARGVTFSRGWLPFLPTSDPPAHTRMRKQLAPGMARGALETWRPMV

DQLARELVGGLLTQTPADVVSTVAAPMPMRAITSVLGVDGPDEAAFCRLS

NQAVRITDVALSASGLISLVQGFAGFRRLRALFTHRRDNGLLRECTVLGK

LATHAEQGRLSDDELFFFAVLLLVAGYESTAHMISTLFLTLADYPDQLTL

LAQQPDLIPSAIEEHLRFISPIQNICRTTRVDYSVGQAVIPAGSLVLLAW

GAANRDPRQYEDPDVFRADRNPVGHLAFGSGIHLCPGTQLARMEGQAILR

EIVANIDRIEVVEPPTWTTNANLRGLTRLRVAVTPRVAP

>CYP128A1 (2603976538) Mycobacterium tuberculosis TB_RSA179

MTATQSPPEPAPDRVRLAGCPLAGTPDVGLTAQDATTALGVPTRRRASSG

GIPVATSMWRDAQTVRTYGPAVAKALALRVAGKARSRLTGRHCRKFMQLT

DFDPFDPAIAADPYPHYRELLAGERVQYNPKRDVYILSRYADVREAARNH

DTLSSARGVTFSRGWLPFLPTSDPPAHTRMRKQLAPGMARGALETWRPMV

DQLARELVGGLLTQTPADVVSTVAAPMPMRAITSVLGVDGPDEAAFCRLS

NQAVRITDVALSASGLISLVQGFAGFRRLRALFTHRRDNGLLRECTVLGK

LATHAEQGRLSDDELFFFAVLLLVAGYESTAHMISTLFLTLADYPDQLTL

LAQQPDLIPSAIEEHLRFISPIQNICRTTRVDYSVGQAVIPAGSLVLLAW

GAANRDPRQYEDPDVFRADRNPVGHLAFGSGIHLCPGTQLARMEGQAILR

EIVANIDRIEVVEPPTWTTNANLRGLTRLRVAVTPRVAP

>CYP128A1 (2605381544) Mycobacterium tuberculosis TB_RSA164

MTATQSPPEPAPDRVRLAGCPLAGTPDVGLTAQDATTALGVPTRRRASSG

GIPVATSMWRDAQTVRTYGPAVAKALALRVAGKARSRLTGRHCRKFMQLT

DFDPFDPAIAADPYPHYRELLAGERVQYNPKRDVYILSRYADVREAARNH

DTLSSARGVTFSRGWLPFLPTSDPPAHTRMRKQLAPGMARGALETWRPMV

DQLARELVGGLLTQTPADVVSTVAAPMPMRAITSVLGVDGPDEAAFCRLS

NQAVRITDVALSASGLISLVQGFAGFRRLRALFTHRRDNGLLRECTVLGK

LATHAEQGRLSDDELFFFAVLLLVAGYESTAHMISTLFLTLADYPDQLTL

LAQQPDLIPSAIEEHLRFISPIQNICRTTRVDYSVGQAVIPAGSLVLLAW

GAANRDPRQYEDPDVFRADRNPVGHLAFGSGIHLCPGTQLARMEGQAILR

EIVANIDRIEVVEPPTWTTNANLRGLTRLRVAVTPRVAP

>CYP128A1 (2622400805) Mycobacterium tuberculosis M1270

MTATQSPPEPAPDRVRLAGCPLAGTPDVGLTAQDATTALGVPTRRRASSG

GIPVATSMWRDAQTVRTYGPAVAKALALRVAGKARSRLTGRHCRKFMQLT

DFDPFDPAIAADPYPHYRELLAGERVQYNPKRDVYILSRYADVREAARNH

DTLSSARGVTFSRGWLPFLPTSDPPAHTRMRKQLAPGMARGALETWRPMV

DQLARELVGGLLTQTPADVVSTVAAPMPMRAITSVLGVDGPDEAAFCRLS

NQAVRITDVALSASGLISLVQGFAGFRRLRALFTHRRDNGLLRECTVLGK

LATHAEQGRLSDDELFFFAVLLLVAGYESTAHMISTLFLTLADYPDQLTL

LAQQPDLIPSAIEEHLRFISPIQNICRTTRVDYSVGQAVIPAGSLVLLAW

GAANRDPRQYEDPDVFRADRNPVGHLAFGSGIHLCPGTQLARMEGQAILR

EIVANIDRIEVVEPPTWTTNANLRGLTRLRVAVTPRVAP

>CYP128A1 (2622617987) Mycobacterium tuberculosis M1638

MTATQSPPEPAPDRVRLAGCPLAGTPDVGLTAQDATTALGVPTRRRASSG

GIPVATSMWRDAQTVRTYGPAVAKALALRVAGKARSRLTGRHCRKFMQLT

DFDPFDPAIAADPYPHYRELLAGERVQYNPKRDVYILSRYADVREAARNH

DTLSSARGVTFSRGWLPFLPTSDPPAHTRMRKQLAPGMARGALETWRPMV

DQLARELVGGLLTQTPADVVSTVAAPMPMRAITSVLGVDGPDEAAFCRLS

NQAVRITDVALSASGLISLVQGFAGFRRLRALFTHRRDNGLLRECTVLGK

LATHAEQGRLSDDELFFFAVLLLVAGYESTAHMISTLFLTLADYPDQLTL

LAQQPDLIPSAIEEHLRFISPIQNICRTTRVDYSVGQAVIPAGSLVLLAW

GAANRDPRQYEDPDVFRADRNPVGHLAFGSGIHLCPGTQLARMEGQAILR

EIVANIDRIEVVEPPTWTTNANLRGLTRLRVAVTPRVAP

>CYP128A1 (2665483571) Mycobacterium tuberculosis 400968

MTATQSPPEPAPDRVRLAGCPLAGTPDVGLTAQDATTALGVPTRRRASSG

GIPVATSMWRDAQTVRTYGPAVAKALALRVAGKARSRLTGRHCRKFMQLT

DFDPFDPAIAADPYPHYRELLAGERVQYNPKRDVYILSRYADVREAARNH

DTLSSARGVTFSRGWLPFLPTSDPPAHTRMRKQLAPGMARGALETWRPMV

DQLARELVGGLLTQTPADVVSTVAAPMPMRAITSVLGVDGPDEAAFCRLS

NQAVRITDVALSASGLISLVQGFAGFRRLRALFTHRRDNGLLRECTVLGK

LATHAEQGRLSDDELFFFAVLLLVAGYESTAHMISTLFLTLADYPDQLTL

LAQQPDLIPSAIEEHLRFISPIQNICRTTRVDYSVGQAVIPAGSLVLLAW

GAANRDPRQYEDPDVFRADRNPVGHLAFGSGIHLCPGTQLARMEGQAILR

EIVANIDRIEVVEPPTWTTNANLRGLTRLRVAVTPRVAP

>CYP128A1 (2695499063) Mycobacterium bovis BCG Danish

MTATQSPPEPAPDRVRLAGCPLAGTPDVGLTAQDATTALGVPTRRRASSG

GIPVATSMWRDAQTVRTYGPAVAKALALRVAGKARSRLTGRHCRKFMQLT

DFDPFDPAIAADPYPHYRELLAGERVQYNPKRDVYILSRYADVREAARNH

DTLSSARGVTFSRGWLPFLPTSDPPAHTRMRKQLAPGMARGALETWRPMV

DQFARELVGGLLTQTPADVVSTVAAPMPMRAITSVLGVDGPDEAAFCRLS

NQAVRITDVALSASGLISLVQGFAGFRRLRALFTHRRDNGLLRECTVLGK

LATHAEQGRLSDDELFFFAVLLLVAGYESTAHMISTLFLTLADYPDQLTL

LAQQPDLIPSAIEEHLRFISPIQNICRTTRVDYSVGQAVIPAGSLVLLAW

GAANRDPRQYEDPDVFRADRNPVGHLAFGSGIHLCPGTQLARMEGQAILR

EIVANIDRIEVVEPPTWTTNANLRGLTRLRVAVTPRVAP

>CYP128A1 (2592320914) Mycobacterium tuberculosis TKK_03_0022

MTATQSPPEPAPDRVRLAGCPLAGTPDVGLTAQDATTALGVPTRRRASSG

GIPVATSMWRDAQTVRTYGPAVAKALALRVAGKARSRLTGRHCRKFMQLT

DFDPFDPAIAADPYPHYRELLAGERVQYNPKRDVYILSRYADVREAARNH

DTLSSARGVTFSRGWLPFLPTSDPPAHTRMRKQLAPGMARGALETWRPMV

DQLARELVGGLLTQTPADVVSTVAAPMPMRAITSVLGVDGPDEAAFCRLS

NQAVRITDVALSASGLISLVQGFAGFRRLRALFTHRRDNGLLRECTVLGK

LATHAEQGRLSDDELFFFAVLLLVAGYESPAHMISTLFLTLADYPDQLTL

LAQQPDLIPSAIEEHLRFISPIQNICRTTRVDYSVGQAVIPAGSLVLLAW

GAANRDPRQYEDPDVFRADRNPVGHLAFGSGIHLCPGTQLARMEGQAILR

EIVANIDRIEVVEPPTWTTNANLRGLTRLRVAVTPRVAP

>CYP128A1 (2600457115) Mycobacterium bovis BCG Moreau

MTATQSPPEPAPDRVRLAGCPLAGTPDVGLTAQDATTALGVPTRRRASSG

GIPVATSMWRDAQTVRTYGPAVAKALALRVAGKARSRLTGRHCRKFMQLT

DFDPFDPAIAADPYPHYRELLAGERVQYNPKRDVYILSRYADVREAARNH

DTLSSARGVTFSRGWLPFLPTSDPPAHTRMRKQLAPGMARGALETWRPMV

DQFARELVGGLLTQTPADVVSTVAAPMPMRAITSVLGVDGPDEAAFCRLS

NQAVRITDVALSASGLISLVQGFAGFRRLRALFTHRRDNGLLRECTVLGK

LATHAEQGRLSDDELFFFAVLLLVAGYESTAHMISTLFLTLADYPDQLTL

LAQQPDLIRRRSRSTSALYRQSKTSAAQRASTIRSVKRSSRQAHWCCWHG

VQPTVTRASTKTRMSFAPTATRSGISRSAPASTCVRGPSWRAWRVRRSCA

RSSPISTE

>CYP128A1 (2567130772) Mycobacterium kansasii 662

MQFTDFDPFDPVTAADPYPHYRELLAGERVQYNPKRDVYIVSRYTDVREA

ARNHHALSSAGGVTFSRGCLPFLPTSDPPTHTRLRKQLAPSMARGAVESW

RPMIDRLAHELVAELLTRTPADVVATVAAPLPMRTITAVLGVPGPDEGMF

CRLSNQAARITDVNLSASGLVSLAQGFTGFRRLRALFTQRRATGLLGEHT

VFGRLAAHAEHGRLSDDELFFFAVLLLVAGYESTAHMISTLFLTLTEFPD

QLSLLARRPDLIPSAIEEQLRFVSPIQNICRTTRVDYPVGGAVIPAGSLV

LLAWGAANRDPRQYDDPDVFRADRNPTGHLAFGSGIHLCPGTQLARMEGH

AVLREIVTNIDRIDVVEPPAWTTNANLRGPTRLRVAVTPRASR

>CYP128A1 (2575295955) Mycobacterium tuberculosis TB_RSA148

MTATQSPPEPAPDRVRLAGCPLAGTPDVGLTAQDATTALGVPTRRRASSG

GIPVATSMWRDAQTVRTYGPAVAKALALRVAGKARSRLTGRHCRKFMQLT

DFDPFDPAIAADPYPHYRELLAGERVQYNPKRDVYILSRYADVREAARNH

DTLSSARGVTFSRGWLPFLPTSDPPAHTRMRKQLAPGMARGALETWRPMV

DQLARELVGGLLTQTPADVVSTVAAPMPMRAITSVLGVDGPDEAAFCRLS

NQAVRITDVALSASGLISLVQGFAGFRRLRALFTHRRDNGLLRECTVLGK

LATHAEQGRLSDDELFFFAVLLLVAGYESTAHMISTLFLTLADYPDQLTL

LAQQPDLIPSAIEEHLRFISPIQNICRTTRVDYSVGQAVIPAGSLVLLAW

GAANRDPRQYEDPDVFRADRNPVGHLAFGSGIHLCPGTQLARMEGQAILR

EIVANIDRIEVVEPPTWTTNANLRGLTRLRVAVTPRVAP

>CYP128A1 (2584649875) Mycobacterium tuberculosis M2203

MTATQSPPEPAPDRVRLAGCPLAGTPDVGLTAQDATTALGVPTRRRASSG

GIPVATSMWRDAQTVRTYGPAVAKALALRVAGKARSRLTGRHCRKFMQLT

DFDPFDPAIAADPYPHYRELLAGERVQYNPKRDVYILSRYADVREAARNH

DTLSSARGVTFSRGWLPFLPTSDPPAHTRMRKQLAPGMARGALETWRPMV

DQLARELVGGLLTQTPADVVSTVAAPMPMRAITSVLGVDGPDEAAFCRLS

NQAVRITDVALSASGLISLVQGFAGFRRLRALFTHRRDNGLLRECTVLGK

LATHAEQGRLSDDELFFFAVLLLVAGYESTAHMISTLFLTLADYPDQLTL

LAQQPDLIPSAIEEHLRFISPIQNICRTTRVDYSVGQAVIPAGSLVLLAW

GAANRDPRQYEDPDVFRADRNPVGHLAFGSGIHLCPGTQLARMEGQAILR

EIVANIDRIEVVEPPTWTTNANLRGLTRLRVAVTPRVAP

>CYP128A1 (2584967618) Mycobacterium tuberculosis TKK_04_0002

MTATQSPPEPAPDRVRLAGCPLAGTPDVGLTAQDATTALGVPTRRRASSG

GIPVATSMWRDAQTVRTYGPAVAKALALRVAGKARSRLTGRHCRKFMQLT

DFDPFDPAIAADPYPHYRELLAGERVQYNPKRDVYILSRYADVREAARNH

DTLSSARGVTFSRGWLPFLPTSDPPAHTRMRKQLAPGMARGALETWRPMV

DQLARELVGGLLTQTPADVVSTVAAPMPMRAITSVLGVDGPDEAAFCRLS

NQAVRITDVALSASGLISLVQGFAGFRRLRALFTHRRDNGLLRECTVLGK

LATHAEQGRLSDDELFFFAVLLLVAGYESTAHMISTLFLTLADYPDQLTL

LAQQPDLIPSAIEEHLRFISPIQNICRTTRVDYSVGQAVIPAGSLVLLAW

GAANRDPRQYEDPDVFRADRNPVGHLAFGSGIHLCPGTQLARMEGQAILR

EIVANIDRIEVVEPPTWTTNANLRGLTRLRVAVTPRVAP

>CYP128A1 (2589052957) Mycobacterium tuberculosis TBR23

MTATQSPPEPAPDRVRLAGCPLAGTPDVGLTAQDATTALGVPTRRRASSG

GIPVATSMWRDAQTVRTYGPAVAKALALRVAGKARSRLTGRHCRKFMQLT

DFDPFDPAIAADPYPHYRELLAGERVQYNPKRDVYILSRYADVREAARNH

DTLSSARGVTFSRGWLPFLPTSDPPAHTRMRKQLAPGMARGALETWRPMV

DQLARELVGGLLTQTPADVVSTVAAPMPMRAITSVLGVDGPDEAAFCRLS

NQAVRITDVALSASGLISLVQGFAGFRRLRALFTHRRDNGLLRECTVLGK

LATHAEQGRLSDDELFFFAVLLLVAGYESTAHMISTLFLTLADYPDQLTL

LAQQPDLIPSAIEEHLRFISPIQNICRTTRVDYSVGQAVIPAGSLVLLAW

GAANRDPRQYEDPDVFRADRNPVGHLAFGSGIHLCPGTQLARMEGQAILR

EIVANIDRIEVVEPPTWTTNANLRGLTRLRVAVTPRVAP

>CYP128A1 (2590081889) Mycobacterium tuberculosis MAL020160

MTATQSPPEPAPDRVRLAGCPLAGTPDVGLTAQDATTALGVPTRRRASSG

GIPVATSMWRDAQTVRTYGPAVAKALALRVAGKARSRLTGRHCRKFMQLT

DFDPFDPAIAADPYPHYRELLAGERVQYNPKRDVYILSRYADVREAARNH

DTLSSARGVTFSRGWLPFLPTSDPPAHTRMRKQLAPGMARGALETWRPMV

DQLARELVGGLLTQTPADVVSTVAAPMPMRAITSVLGVDGPDEAAFCRLS

NQAVRITDVALSASGLISLVQGFAGFRRLRALFTHRRDNGLLRECTVLGK

LATHAEQGRLSDDELFFFAVLLLVAGYESTAHMISTLFLTLADYPDQLTL

LAQQPDLIPSAIEEHLRFISPIQNICRTTRVDYSVGQAVIPAGSLVLLAW

GAANRDPRQYEDPDVFRADRNPVGHLAFGSGIHLCPGTQLARMEGQAILR

EIVANIDRIEVVEPPTWTTNANLRGLTRLRVAVTPRVAP

>CYP128A1 (2592284241) Mycobacterium tuberculosis TKK_03_0045

MTATQSPPEPAPDRVRLAGCPLAGTPDVGLTAQDATTALGVPTRRRASSG

GIPVATSMWRDAQTVRTYGPAVAKALALRVAGKARSRLTGRHCRKFMQLT

DFDPFDPAIAADPYPHYRELLAGERVQYNPKRDVYILSRYADVREAARNH

DTLSSARGVTFSRGWLPFLPTSDPPAHTRMRKQLAPGMARGALETWRPMV

DQLARELVGGLLTQTPADVVSTVAAPMPMRAITSVLGVDGPDEAAFCRLS

NQAVRITDVALSASGLISLVQGFAGFRRLRALFTHRRDNGLLRECTVLGK

LATHAEQGRLSDDELFFFAVLLLVAGYESTAHMISTLFLTLADYPDQLTL

LAQQPDLIPSAIEEHLRFISPIQNICRTTRVDYSVGQAVIPAGSLVLLAW

GAANRDPRQYEDPDVFRADRNPVGHLAFGSGIHLCPGTQLARMEGQAILR

EIVANIDRIEVVEPPTWTTNANLRGLTRLRVAVTPRVAP

>CYP128A1 (2603325561) Mycobacterium tuberculosis BTB08-072

MTATQSPPEPAPDRVRLAGCPLAGTPDVGLTAQDATTALGVPTRRRASSG

GIPVATSMWRDAQTVRTYGPAVAKALALRVAGKARSRLTGRHCRKFMQLT

DFDPFDPAIAADPYPHYRELLAGERVQYNPKRDVYILSRYADVREAARNH

DTLSSARGVTFSRGWLPFLPTSDPPAHTRMRKQLAPGMARGALETWRPMV

DQLARELVGGLLTQTPADVVSTVAAPMPMRAITSVLGVDGPDEAAFCRLS

NQAVRITDVALSASGLISLVQGFAGFRRLRALFTHRRDNGLLRECTVLGK

LATHAEQGRLSDDELFFFAVLLLVAGYESTAHMISTLFLTLADYPDQLTL

LAQQPDLIPSAIEEHLRFISPIQNICRTTRVDYSVGQAVIPAGSLVLLAW

GAANRDPRQYEDPDVFRADRNPVGHLAFGSGIHLCPGTQLARMEGQAILR

EIVANIDRIEVVEPPTWTTNANLRGLTRLRVAVTPRVAP

>CYP128A1 (2604335285) Mycobacterium tuberculosis TB_RSA58

MTATQSPPEPAPDRVRLAGCPLAGTPDVGLTAQDATTALGVPTRRRASSG

GIPVATSMWRDAQTVRTYGPAVAKALALRVAGKARSRLTGRHCRKFMQLT

DFDPFDPAIAADPYPHYRELLAGERVQYNPKRDVYILSRYADVREAARNH

DTLSSARGVTFSRGWLPFLPTSDPPAHTRMRKQLAPGMARGALETWRPMV

DQLARELVGGLLTQTPADVVSTVAAPMPMRAITSVLGVDGPDEAAFCRLS

NQAVRITDVALSASGLISLVQGFAGFRRLRALFTHRRDNGLLRECTVLGK

LATHAEQGRLSDDELFFFAVLLLVAGYESTAHMISTLFLTLADYPDQLTL

LAQQPDLIPSAIEEHLRFISPIQNICRTTRVDYSVGQAVIPAGSLVLLAW

GAANRDPRQYEDPDVFRADRNPVGHLAFGSGIHLCPGTQLARMEGQAILR

EIVANIDRIEVVEPPTWTTNANLRGLTRLRVAVTPRVAP

>CYP128A1 (2584640497) Mycobacterium tuberculosis XTB13-088

MTATQSPPEPAPDRVRLAGCPLAGTPDVGLTAQDATTALGVPTRRRAASG

GIPVATSMWRDAQTVRTYGPAVAKALALRVAGKARSRLTGRHCRKFMQLT

DFDPFDPAIAADPYPHYRELLAGERVQYNPKRDVYILSRYADVREAARNH

DTLSSARGVTFSRGWLPFLPTSDPPAHTRMRKQLAPGMARGALETWRPMV

DQLARELVGGLLTQTPADVVSTVAAPMPMRAITSVLGVDGPDEAAFCRLS

NQAVRITDVALSASGLISLVQGFAGFRRLRALFTHRRDNGLLRECTVLGK

LATHAEQGRLSDDELFFFAVLLLVAGYESTAHMISTLFLTLADYPDQLTL

LAQQPDLIPSAIEEHLRFISPIQNICRTTRVDYSVGQAVIPAGSLVLLAW

GAANRDPRQYEDPDVFRADRNPVGHLAFGSGIHLCPGTQLARMEGQAILR

EIVANIDRIEVVEPPTWTTNANLRGLTRLRVAVTPRVAP

>CYP128A1 (2576714007) Mycobacterium tuberculosis KT-0056

MTATQSPPEPAPDRVRLAGCPLAGTPDVGLTAQDATTALGVPTRRRASSG

GIPVATSMWRDAQTVRTYGPAVAKALALRVAGKARSRLTGRHCRKFMQLT

DFDPFDPAIAADPYPHYRELLAGERVQYNPKRDVYILSRYADVREAARNH

DTLSSARGVTFSRGWLPFLPTSDPPAHTRMRKQLAPGMARGALETWRPMV

DQLARELVGGLLTQTPADVVSTVAAPMPMRAITSVLGVDGPDEAAFCRLS

NQAVRITDVALSASGLISLVQGFAGFRRLRALFTHRRDNGLLRECTVLGK

LAAHAEQGRLSDDELFFFAVLLLVAGYESTAHMISTLFLTLADYPDQLTL

LAQQPDLIPSAIEEHLRFISPIQNICRTTRVDYSVGQAVIPAGSLVLLAW

GAANRDPRQYEDPDVFRADRNPVGHLAFGSGIHLCPGTQLARMEGQAILR

EIVANIDRIEVVEPPTWTTNANLRGLTRLRVAVTPRVAP

>CYP128A1 (2605588815) Mycobacterium tuberculosis BTB04-158

MTATQSPPEPAPDRVRLAGCPLAGTPDVGLTAQDATTALGVPTRRRASSG

GIPVATSMWRDAQTVRTYGPAVAKALALRVAGKARSRLTGRHCRKFMQLT

DFDPFDPAIAADPYPHYRELLAGERVQYNPKRDVYILSRYADVREAARNH

DTLSSARGVTFSRGWLPFLPTSDPPAHTRMRKQLAPGMARGALETWRPMV

DQLARELVGGLLTQTPADVVSTVAAPMPMRAITSVLGADGPDEAAFCRLS

NQAVRITDVALSASGLISLVQGFAGFRRLRALFTHRRDNGLLRECTVLGK

LATHAEQGRLSDDELFFFAVLLLVAGYESTAHMISTLFLTLADYPDQLTL

LAQQPDLIPSAIEEHLRFISPIQNICRTTRVDYSVGQAVIPAGSLVLLAW

GAANRDPRQYEDPDVFRADRNPVGHLAFGSGIHLCPGTQLARMEGQAILR

EIVANIDRIEVVEPPTWTTNANLRGLTRLRVAVTPRVAP

>CYP128A1 (2695543263) Mycobacterium bovis BCG Connaught

MTATQSPPEPAPDRVRLAGCPLAGTPDVGLTAQDATTALGVPTRRRASSG

GIPVATSMWRDAQTVRTYGPAVAKALALRVAGKARSRLTGRHCRKFMQLT

DFDPFDPAIAADPYPHYRELLAGERVQYNPKRDVYILSRYADVREAARNH

DTLSSARGVTFSRGWLPFLPTSDPPAHTRMRKQLAPGMARGALETWRPMV

DQFARELVGGLLTQTPADVVSTVAAPMPMRAITSVLGVDGPDEAAFCRLS

NQAVRITDVALSASGLISLVQGFAGFRRLRALFTHRRDNGLLRECTVLGK

LATHAEQGRLSDDELFFFAVLLLVAGYESTAHMISTLFLTLADYPDQLTL

LAQQPDLIPSAIEEHLRFISPIQNICRTTRVDYSVGQAVIPAGSLVLLAW

GAANRDPRQYEDPDVFRADRNPVGHLAFGSGIHLCPGTQLARMEGQAILR

EIVANIDRIEVVEPPTWTTNANLRGLTRLRVAVTPRVAP

>CYP128A1 (2671006424) Mycobacterium tuberculosis 1500108

MTATQSPPEPAPDRVRLAGCPLAGTPDVGLTAQDATTALGVPTRRRASSG

GIPVATSMWRDAQTVRTYGPAVAKALALRVAGKARSRLTGRHCRKFMQLT

DFDPFDPAIAADPYPHYRELLAGERVQYNPKRDVYILSRYADVREAARNH

DTLSSARGVTFSRGWLPFLPTSDPPAHTRMRKQLAPGMARGALETWRPMV

DQLARELVGGLLTQTPADVVSTVAAPMPMRAITSVLGVDGPDEAAFCRLS

NQAVRITDVALSASGLISLVQGFAGFRRLRALFTHRRDNGLLRECTVLGK

LATHAEQGRLSDDELFFFAVLLLVAGYESTAHMISTLFLTLADYPDQLTL

LAQQPDLIPSAIEEHRRFISPIQNICRTTRVDYSVGQAVIPAGSLVLLAW

GAANRDPRQYEDPDVFRADRNPVGHLAFGSGIHLCPGTQLARMEGQAILR

EIVANIDRIEVVEPPTWTTNANLRGLTRLRVAVTPRVAP

>CYP128A1 (2675246507) Mycobacterium tuberculosis N00500166

MASLTSLAVRVRRLRALFTHRRDNGLLRECTVLGKLATHAEQGRLSDDEL

FFFAVLLLVAGYESTAHMISTLFLTLADYPDQLTLLAQQPDLIPSAIEEH

LRFISPIQNICRTTRVDYSVGQAVIPAGSLVLLAWGAANRDPRQYEDPDV

FRADRNPVGHLAFGSGIHLCPGTQLARMEGQAILREIVANIDRIEVVEPP

TWTTNANLRGLTRLRVAVTPRVAP

>CYP128B4 (2669868338) Mycobacterium abscessus PAP019

MSRWRSDSAVRQIGAPVVAAVGMNIAAAVRVRRRGYAGWTGAVNTDYDPL

DPATAAQPFDAYRALHAGGRVHYNPKRATFILSRHEDIRAALRDTDAVTS

SQGVTRMKISAPILVLTDGDDHTRLRKQVQPGFTRGAMSDWQGMADQLAK

ELVADVVANPGCDVMERLAVPLPIRMIAHIIGIPPEDVQNFRSWSEDGVG

VINAGVSPAGLRQGLKGVRAIAALRRYFKDQLASGKLKGSDTVLGRLVDN

NEDGKLSDDELFFIAMLLLFAGNETTTNLIGGMFDTLAHAPDQFAMIRDD

PDLIPSAVEEQLRYSAPIQNLYRYTRTDYRVGEVTIPSGSRLLLAFGAAN

RDPEVFEDPDTYRADRNPRNHIAFGYGVHMCIGATLSRMEGQAVLRELTS

QASAIAAAGSATWSTNSSLRGTTYLPIRLTPAR

>CYP128A1 (2574726751) Mycobacterium tuberculosis TKK_02_0015

MTATQSPPEPAPDRVRLAGCPLAGTPDVGLTAQDATTALGVPTRRRASSG

GIPVATSMWRDAQTVRTYGPAVAKALALRVAGKARSRLTGRHCRKFMQLT

DFDPFDPAIAADPYPHYRELLAGERVQYNPKRDVYILSRYADVREAARNH

DTLSSARGVTFSRGWLPFLPTSDPPAHTRMRKQLAPGMARGALETWRPMV

DQLARELVGGLLTQTPADVVSTVAAPMPMRAITSVLGVDGPDEAAFCRLS

NQAVRITDVALSASGLISLVQGFAGFRRLRALFTHRRDNGLLRECTVLGK

LATHAEQGRLSDDELFFFAVLLLVAGYESTAHMISTLFLTLADYPDQLTL

LAQQPDLIPSAIEEHLRFISPIQNICRTTRVDYSVGQAVIPAGSLVLLAW

GAANRDPRQYEDPDVFRADRNPVGHLAFGSGIHLCPGTQLARMEGQAILR

EIVANIDRIEVVEPPTWTTNANLRGLTRLRVAVTPRVAP

>CYP128A1 (2575363124) Mycobacterium tuberculosis M1449

MTATQSPPEPAPDRVRLAGCPLAGTPDVGLTAQDATTALGVPTRRRASSG

GIPVATSMWRDAQTVRTYGPAVAKALALRVAGKARSRLTGRHCRKFMQLT

DFDPFDPAIAADPYPHYRELLAGERVQYNPKRDVYILSRYADVREAARNH

DTLSSARGVTFSRGWLPFLPTSDPPAHTRMRKQLAPGMARGALETWRPMV

DQLARELVGGLLTQTPADVVSTVAAPMPMRAITSVLGVDGPDEAAFCRLS

NQAVRITDVALSASGLISLVQGFAGFRRLRALFTHRRDNGLLRECTVLGK

LATHAEQGRLSDDELFFFAVLLLVAGYESTAHMISTLFLTLADYPDQLTL

LAQQPDLIPSAIEEHLRFISPIQNICRTTRVDYSVGQAVIPAGSLVLLAW

GAANRDPRQYEDPDVFRADRNPVGHLAFGSGIHLCPGTQLARMEGQAILR

EIVANIDRIEVVEPPTWTTNANLRGLTRLRVAVTPRVAP

>CYP128A1 (2577750549) Mycobacterium tuberculosis TKK-01-0068

MTATQSPPEPAPDRVRLAGCPLAGTPDVGLTAQDATTALGVPTRRRASSG

GIPVATSMWRDAQTVRTYGPAVAKALALRVAGKARSRLTGRHCRKFMQLT

DFDPFDPAIAADPYPHYRELLAGERVQYNPKRDVYILSRYADVREAARNH

DTLSSARGVTFSRGWLPFLPTSDPPAHTRMRKQLAPGMARGALETWRPMV

DQLARELVGGLLTQTPADVVSTVAAPMPMRAITSVLGVDGPDEAAFCRLS

NQAVRITDVALSASGLISLVQGFAGFRRLRALFTHRRDNGLLRECTVLGK

LATHAEQGRLSDDELFFFAVLLLVAGYESTAHMISTLFLTLADYPDQLTL

LAQQPDLIPSAIEEHLRFISPIQNICRTTRVDYSVGQAVIPAGSLVLLAW

GAANRDPRQYEDPDVFRADRNPVGHLAFGSGIHLCPGTQLARMEGQAILR

EIVANIDRIEVVEPPTWTTNANLRGLTRLRVAVTPRVAP

>CYP128A1 (2584105772) Mycobacterium bovis Kc 32216

MTATQSPPEPAPDRVRLAGCPLAGTPDVGLTAQDATTALGVPTRRRASSG

GIPVATSMWRDAQTVRTYGPAVAKALALRVAGKARSRLTGRHCRKFMQLT

DFDPFDPAIAADPYPHYRELLAGERVQYNPKRDVYILSRYADVREAARNH

DTLSSARGVTFSRGWLPFLPTSDPPAHTRMRKQLAPGMARGALETWRPMV

DQLARELVGGLLTQTPADVVSTVAAPMPMRAITSVLGVDGPDEAAFCRLS

NQAVRITDVALSASGLISLVQGFAGFRRLRALFTHRRDNGLLRECTVLGK

LATHAEQGRLSDDELFFFAVLLLVAGYESTAHMISTLFLTLADYPDQLTL

LAQQPDLIPSAIEEHLRFISPIQNICRTTRVDYSVGQAVIPAGSLVLLAW

GAANRDPRQYEDPDVFRADRNPVGHLAFGSGIHLCPGTQLARMEGQAILR

EIVANIDRIEVVEPPTWTTNANLRGLTRLRVAVTPRVAP

>CYP128A1 (2584898236) Mycobacterium tuberculosis TKK-01-0065

MTATQSPPEPAPDRVRLAGCPLAGTPDVGLTAQDATTALGVPTRRRASSG

GIPVATSMWRDAQTVRTYGPAVAKALALRVAGKARSRLTGRHCRKFMQLT

DFDPFDPAIAADPYPHYRELLAGERVQYNPKRDVYILSRYADVREAARNH

DTLSSARGVTFSRGWLPFLPTSDPPAHTRMRKQLAPGMARGALETWRPMV

DQLARELVGGLLTQTPADVVSTVAAPMPMRAITSVLGVDGPDEAAFCRLS

NQAVRITDVALSASGLISLVQGFAGFRRLRALFTHRRDNGLLRECTVLGK

LATHAEQGRLSDDELFFFAVLLLVAGYESTAHMISTLFLTLADYPDQLTL

LAQQPDLIPSAIEEHLRFISPIQNICRTTRVDYSVGQAVIPAGSLVLLAW

GAANRDPRQYEDPDVFRADRNPVGHLAFGSGIHLCPGTQLARMEGQAILR

EIVANIDRIEVVEPPTWTTNANLRGLTRLRVAVTPRVAP

>CYP128A1 (2590214024) Mycobacterium tuberculosis KT-0058

MTATQSPPEPAPDRVRLAGCPLAGTPDVGLTAQDATTALGVPTRRRASSG

GIPVATSMWRDAQTVRTYGPAVAKALALRVAGKARSRLTGRHCRKFMQLT

DFDPFDPAIAADPYPHYRELLAGERVQYNPKRDVYILSRYADVREAARNH

DTLSSARGVTFSRGWLPFLPTSDPPAHTRMRKQLAPGMARGALETWRPMV

DQLARELVGGLLTQTPADVVSTVAAPMPMRAITSVLGVDGPDEAAFCRLS

NQAVRITDVALSASGLISLVQGFAGFRRLRALFTHRRDNGLLRECTVLGK

LATHAEQGRLSDDELFFFAVLLLVAGYESTAHMISTLFLTLADYPDQLTL

LAQQPDLIPSAIEEHLRFISPIQNICRTTRVDYSVGQAVIPAGSLVLLAW

GAANRDPRQYEDPDVFRADRNPVGHLAFGSGIHLCPGTQLARMEGQAILR

EIVANIDRIEVVEPPTWTTNANLRGLTRLRVAVTPRVAP

>CYP128A1 (2592347597) Mycobacterium tuberculosis TKK_02_0068

MTATQSPPEPAPDRVRLAGCPLAGTPDVGLTAQDATTALGVPTRRRASSG

GIPVATSMWRDAQTVRTYGPAVAKALALRVAGKARSRLTGRHCRKFMQLT

DFDPFDPAIAADPYPHYRELLAGERVQYNPKRDVYILSRYADVREAARNH

DTLSSARGVTFSRGWLPFLPTSDPPAHTRMRKQLAPGMARGALETWRPMV

DQLARELVGGLLTQTPADVVSTVAAPMPMRAITSVLGVDGPDEAAFCRLS

NQAVRITDVALSASGLISLVQGFAGFRRLRALFTHRRDNGLLRECTVLGK

LATHAEQGRLSDDELFFFAVLLLVAGYESTAHMISTLFLTLADYPDQLTL

LAQQPDLIPSAIEEHLRFISPIQNICRTTRVDYSVGQAVIPAGSLVLLAW

GAANRDPRQYEDPDVFRADRNPVGHLAFGSGIHLCPGTQLARMEGQAILR

EIVANIDRIEVVEPPTWTTNANLRGLTRLRVAVTPRVAP

>CYP128A1 (2604020088) Mycobacterium tuberculosis TB_RSA85

MTATQSPPEPAPDRVRLAGCPLAGTPDVGLTAQDATTALGVPTRRRASSG

GIPVATSMWRDAQTVRTYGPAVAKALALRVAGKARSRLTGRHCRKFMQLT

DFDPFDPAIAADPYPHYRELLAGERVQYNPKRDVYILSRYADVREAARNH

DTLSSARGVTFSRGWLPFLPTSDPPAHTRMRKQLAPGMARGALETWRPMV

DQLARELVGGLLTQTPADVVSTVAAPMPMRAITSVLGVDGPDEAAFCRLS

NQAVRITDVALSASGLISLVQGFAGFRRLRALFTHRRDNGLLRECTVLGK

LATHAEQGRLSDDELFFFAVLLLVAGYESTAHMISTLFLTLADYPDQLTL

LAQQPDLIPSAIEEHLRFISPIQNICRTTRVDYSVGQAVIPAGSLVLLAW

GAANRDPRQYEDPDVFRADRNPVGHLAFGSGIHLCPGTQLARMEGQAILR

EIVANIDRIEVVEPPTWTTNANLRGLTRLRVAVTPRVAP

>CYP128A1 (2606482665) Mycobacterium tuberculosis 2543MS

MTATQSPPEPAPDRVRLAGCPLAGTPDVGLTAQDATTALGVPTRRRASSG

GIPVATSMWRDAQTVRTYGPAVAKALALRVAGKARSRLTGRHCRKFMQLT

DFDPFDPAIAADPYPHYRELLAGERVQYNPKRDVYILSRYADVREAARNH

DTLSSARGVTFSRGWLPFLPTSDPPAHTRMRKQLAPGMARGALETWRPMV

DQLARELVGGLLTQTPADVVSTVAAPMPMRAITSVLGVDGPDEAAFCRLS

NQAVRITDVALSASGLISLVQGFAGFRRLRALFTHRRDNGLLRECTVLGK

LATHAEQGRLSDDELFFFAVLLLVAGYESTAHMISTLFLTLADYPDQLTL

LAQQPDLIPSAIEEHLRFISPIQNICRTTRVDYSVGQAVIPAGSLVLLAW

GAANRDPRQYEDPDVFRADRNPVGHLAFGSGIHLCPGTQLARMEGQAILR

EIVANIDRIEVVEPPTWTTNANLRGLTRLRVAVTPRVAP

>CYP128A1 (2621650758) Mycobacterium tuberculosis XTB13-246

MTATQSPPEPAPDRVRLAGCPLAGTPDVGLTAQDATTALGVPTRRRASSG

GIPVATSMWRDAQTVRTYGPAVAKALALRVAGKARSRLTGRHCRKFMQLT

DFDPFDPAIAADPYPHYRELLAGERVQYNPKRDVYILSRYADVREAARNH

DTLSSARGVTFSRGWLPFLPTSDPPAHTRMRKQLAPGMARGALETWRPMV

DQLARELVGGLLTQTPADVVSTVAAPMPMRAITSVLGVDGPDEAAFCRLS

NQAVRITDVALSASGLISLVQGFAGFRRLRALFTHRRDNGLLRECTVLGK

LATHAEQGRLSDDELFFFAVLLLVAGYESTAHMISTLFLTLADYPDQLTL

LAQQPDLIPSAIEEHLRFISPIQNICRTTRVDYSVGQAVIPAGSLVLLAW

GAANRDPRQYEDPDVFRADRNPVGHLAFGSGIHLCPGTQLARMEGQAILR

EIVANIDRIEVVEPPTWTTNANLRGLTRLRVAVTPRVAP

>CYP128A1 (2622634870) Mycobacterium tuberculosis M1728

MTATQSPPEPAPDRVRLAGCPLAGTPDVGLTAQDATTALGVPTRRRASSG

GIPVATSMWRDAQTVRTYGPAVAKALALRVAGKARSRLTGRHCRKFMQLT

DFDPFDPAIAADPYPHYRELLAGERVQYNPKRDVYILSRYADVREAARNH

DTLSSARGVTFSRGWLPFLPTSDPPAHTRMRKQLAPGMARGALETWRPMV

DQLARELVGGLLTQTPADVVSTVAAPMPMRAITSVLGVDGPDEAAFCRLS

NQAVRITDVALSASGLISLVQGFAGFRRLRALFTHRRDNGLLRECTVLGK

LATHAEQGRLSDDELFFFAVLLLVAGYESTAHMISTLFLTLADYPDQLTL

LAQQPDLIPSAIEEHLRFISPIQNICRTTRVDYSVGQAVIPAGSLVLLAW

GAANRDPRQYEDPDVFRADRNPVGHLAFGSGIHLCPGTQLARMEGQAILR

EIVANIDRIEVVEPPTWTTNANLRGLTRLRVAVTPRVAP

>CYP128A1 (2651638210) Mycobacterium tuberculosis M2138

MTATQSPPEPAPDRVRLAGCPLAGTPDVGLTAQDATTALGVPTRRRASSG

GIPVATSMWRDAQTVRTYGPAVAKALALRVAGKARSRLTGRHCRKFMQLT

DFDPFDPAIAADPYPHYRELLAGERVQYNPKRDVYILSRYADVREAARNH

DTLSSARGVTFSRGWLPFLPTSDPPAHTRMRKQLAPGMARGALETWRPMV

DQLARELVGGLLTQTPADVVSTVAAPMPMRAITSVLGVDGPDEAAFCRLS

NQAVRITDVALSASGLISLVQGFAGFRRLRALFTHRRDNGLLRECTVLGK

LATHAEQGRLSDDELFFFAVLLLVAGYESTAHMISTLFLTLADYPDQLTL

LAQQPDLIPSAIEEHLRFISPIQNICRTTRVDYSVGQAVIPAGSLVLLAW

GAANRDPRQYEDPDVFRADRNPVGHLAFGSGIHLCPGTQLARMEGQAILR

EIVANIDRIEVVEPPTWTTNANLRGLTRLRVAVTPRVAP

>CYP128A1 (2693128271) Mycobacterium tuberculosis A70785

MTATQSPPEPAPDRVRLAGCPLAGTPDVGLTAQDATTALGVPTRRRASSG

GIPVATSMWRDAQTVRTYGPAVAKALALRVAGKARSRLTGRHCRKFMQLT

DFDPFDPAIAADPYPHYRELLAGERVQYNPKRDVYILSRYADVREAARNH

DTLSSARGVTFSRGWLPFLPTSDPPAHTRMRKQLAPGMARGALETWRPMV

DQLARELVGGLLTQTPADVVSTVAAPMPMRAITSVLGVDGPDEAAFCRLS

NQAVRITDVALSASGLISLVQGFAGFRRLRALFTHRRDNGLLRECTVLGK

LATHAEQGRLSDDELFFFAVLLLVAGYESTAHMISTLFLTLADYPDQLTL

LAQQPDLIPSAIEEHLRFISPIQNICRTTRVDYSVGQAVIPAGSLVLLAW

GAANRDPRQYEDPDVFRADRNPVGHLAFGSGIHLCPGTQLARMEGQAILR

EIVANIDRIEVVEPPTWTTNANLRGLTRLRVAVTPRVAP

>CYP128A1 (2703554014) Mycobacterium tuberculosis R09601315

MTATQSPPEPAPDRVRLAGCPLAGTPDVGLTAQDATTALGVPTRRRASSG

GIPVATSMWRDAQTVRTYGPAVAKALALRVAGKARSRLTGRHCRKFMQLT

DFDPFDPAIAADPYPHYRELLAGERVQYNPKRDVYILSRYADVREAARNH

DTLSSARGVTFSRGWLPFLPTSDPPAHTRMRKQLAPGMARGALETWRPMV

DQLARELVGGLLTQTPADVVSTVAAPMPMRAITSVLGVDGPDEAAFCRLS

NQAVRITDVALSASGLISLVQGFAGFRRLRALFTHRRDNGLLRECTVLGK

LATHAEQGRLSDDELFFFAVLLLVAGYESTAHMISTLFLTLADYPDQLTL

LAQQPDLIPSAIEEHLRFISPIQNICRTTRVDYSVGQAVIPAGSLVLLAW

GAANRDPRQYEDPDVFRADRNPVGHLAFGSGIHLCPGTQLARMEGQAILR

EIVANIDRIEVVEPPTWTTNANLRGLTRLRVAVTPRVAP

>CYP128A1 (2707489331) Mycobacterium bovis MB4

MTATQSPPEPAPDRVRLAGCPLAGTPDVGLTAQDATTALGVPTRRRASSG

GIPVATSMWRDAQTVRTYGPAVAKALALRVAGKARSRLTGRHCRKFMQLT

DFDPFDPAIAADPYPHYRELLAGERVQYNPKRDVYILSRYADVREAARNH

DTLSSARGVTFSRGWLPFLPTSDPPAHTRMRKQLAPGMARGALETWRPMV

DQLARELVGGLLTQTPADVVSTVAAPMPMRAITSVLGVDGPDEAAFCRLS

NQAVRITDVALSASGLISLVQGFAGFRRLRALFTHRRDNGLLRECTVLGK

LATHAEQGRLSDDELFFFAVLLLVAGYESTAHMISTLFLTLADYPDQLTL

LAQQPDLIPSAIEEHLRFISPIQNICRTTRVDYSVGQAVIPAGSLVLLAW

GAANRDPRQYEDPDVFRADRNPVGHLAFGSGIHLCPGTQLARMEGQAILR

EIVANIDRIEVVEPPTWTTNANLRGLTRLRVAVTPRVAP

>CYP128A1 (2722808891) Mycobacterium tuberculosis 1458

MTATQSPPEPAPDRVRLAGCPLAGTPDVGLTAQDATTALGVPTRRRASSG

GIPVATSMWRDAQTVRTYGPAVAKALALRVAGKARSRLTGRHCRKFMQLT

DFDPFDPAIAADPYPHYRELLAGERVQYNPKRDVYILSRYADVREAARNH

DTLSSARGVTFSRGWLPFLPTSDPPAHTRMRKQLAPGMARGALETWRPMV

DQLARELVGGLLTQTPADVVSTVAAPMPMRAITSVLGVDGPDEAAFCRLS

NQAVRITDVALSASGLISLVQGFAGFRRLRALFTHRRDNGLLRECTVLGK

LATHAEQGRLSDDELFFFAVLLLVAGYESTAHMISTLFLTLADYPDQLTL

LAQQPDLIPSAIEEHLRFISPIQNICRTTRVDYSVGQAVIPAGSLVLLAW

GAANRDPRQYEDPDVFRADRNPVGHLAFGSGIHLCPGTQLARMEGQAILR

EIVANIDRIEVVEPPTWTTNANLRGLTRLRVAVTPRVAP

>CYP128A1 (2547310369) Mycobacterium bovis BCG-Denmark TMC 1010, ATCC 35733

MTATQSPPEPAPDRVRLAGCPLAGTPDVGLTAQDATTALGVPTRRRASSG

GIPVATSMWRDAQTVRTYGPAVAKALALRVAGKARSRLTGRHCRKFMQLT

DFDPFDPAIAADPYPHYRELLAGERVQYNPKRDVYILSRYADVREAARNH

DTLSSARGVTFSRGWLPFLPTSDPPAHTRMRKQLAPGMARGALETWRPMV

DQFARELVGGLLTQTPADVVSTVAAPMPMRAITSVLGVDGPDEAAFCRLS

NQAVRITDVALSASGLISLVQGFAGFRRLRALFTHRRDNGLLRECTVLGK

LATHAEQGRLSDDELFFFAVLLLVAGYESTAHMISTLFLTLADYPDQLTL

LAQQPDLIPSAIEEHLRFISPIQNICRTTRVDYSVGQAVIPAGSLVLLAW

GAANRDPRQYEDPDVFRADRNPVGHLAFGSGIHLCPGTQLARMEGQAILR

EIVANIDRIEVVEPPTWTTNANLRGLTRLRVAVTPRVAP

>CYP128A1 (2600667903) Mycobacterium bovis BCG Phipps

MTATQSPPEPAPDRVRLAGCPLAGTPDVGLTAQDATTALGVPTRRRASSG

GIPVATSMWRDAQTVRTYGPAVAKALALRVAGKARSRLTGRHCRKFMQLT

DFDPFDPAIAADPYPHYRELLAGERVQYNPKRDVYILSRYADVREAARNH

DTLSSARGVTFSRGWLPFLPTSDPPAHTRMRKQLAPGMARGALETWRPMV

DQFARELVGGLLTQTPADVVSTVAAPMPMRAITSVLGVDGPDEAAFCRLS

NQAVRITDVALSASGLISLVQGFAGFRRLRALFTHRRDNGLLRECTVLGK

LATHAEQGRLSDDELFFFAVLLLVAGYESTAHMISTLFLTLADYPDQLTL

LAQQPDLIPSAIEEHLRFISPIQNICRTTRVDYSVGQAVIPAGSLVLLAW

GAANRDPRQYEDPDVFRADRNPVGHLAFGSGIHLCPGTQLARMEGQAILR

EIVANIDRIEVVEPPTWTTNANLRGLTRLRVAVTPRVAP

>CYP128A1 (2604312418) Mycobacterium tuberculosis TB_RSA191

MTATQSPPEPAPDRVRLAGCPLAGTPDVGLTAQDATTALGVPTRRRASSG

GIPVATSMWRDAQTVRTYGPAVAKALALRVAGKARSRLTGRHCRKFMQLT

DFDPFDPAIAADPYPHYRELLAGERVQYNPKRDVYILSRYADVREAARNH

DTLSSARGVTFSRGWLPFLPTSDPPAHTRMRKQLAPGMARGALETWRPMV

DQLARELVGGLLTQTPADVVSTVAAPMPMRAITSVLGVDGPDEAAFCRLS

NQAVRITDVALSASGLISLVQGFAGFRRLRALFTHRRDNGLLRECTVLGK

LATHAEQGRLSDDELFFFAVLLLVAGYESTAHMISTLFLTLADYPDQLTL

LAQQPDLIPSAIEEHLRFISPIQNICRTTRVDYSVGQAVIPAGSLVLLAW

GAANRDPRQYEDPDVFRADRNPVGHLAFGSGIHLCPGPSWRAWRVRRSCA

RSSPISTE

>CYP128B4 (2803913587) Mycobacteroides abscessus abscessus 805

MSRWRSDSAVRQIGAPVVAAVGMNIAAAVRVRRRGYAGWTGAVNTDYDPL

DPATAAQPFDAYRALHAGGRVHYNPKRATFILSRHEDIRAALRDTDAVTS

SQGVTRMKISAPILVLTDGDDHTRLRKQVQPGFTRGAMSDWQGMADQLAK

ELVADVVANPGCDVMERLAVPLPIRMIAHIIGIPPEDVQNFRSWSEDGVG

VINAGVSPAGLRQGLKGVRAIAALRRYFKDQLASGKLKGSDTVLGRLVDN

NEDGKLSDDELFFIAMLLLFAGNETTTNLIGGMFDTLAHAPDQFAMIRDD

PDLIPSAVEEQLRYSAPIQNLYRYTRTDYRVGEVTIPSGSRLLLAFGAAN

RDPEVFEDPDTYRADRNPRNHIAFGYGVHMCIGATLSRMEGQAVLRELTS

QASAIAAAGSATWSTNSSLRGTTYLPIRLTPAR

>CYP128A1 (2575108167) Mycobacterium tuberculosis 16955

MTATQSPPEPAPDRVRLAGCPLAGTPDVGLTAQDATTALGVPTRRRASSG

GIPVATSMWRDAQTVRTYGPAVAKALALRVAGKARSRLTGRHCRKFMQLT

DFDPFDPAIAADPYPHYRELLAGERVQYNPKRDVYILSRYADVREAARNH

DTLSSARGVTFSRGWLPFLPTSDPPAHTRMRKQLAPGMARGALETWRPMV

DQLARELVGGLLTQTPADVVSTVAAPMPMRAITSVLGVDGPDEAAFCRLS

NQAVRITDVALSASGLISLVQGFAGFRRLRALFTHRRDNGLLRECTVLGK

LATHAEQGRLSDDELFFFAVLLLVAGYESTAHMISTLFLTLADYPDQLTL

LAQQPDLIPSAIEEHLRFISPIQNICRTTRVDYSVGQAVIPAGSLVLLAW

GAANRDPRQYEDPDVFRADRNPVGHLAFGSGIHLCPGTQLARMEGQAILR

EIVANIDRIEVVEPPTWTTNANLRGLTRLRVAVTPRVAP

>CYP128A1 (2575157692) Mycobacterium tuberculosis BTB08-362

MTATQSPPEPAPDRVRLAGCPLAGTPDVGLTAQDATTALGVPTRRRASSG

GIPVATSMWRDAQTVRTYGPAVAKALALRVAGKARSRLTGRHCRKFMQLT

DFDPFDPAIAADPYPHYRELLAGERVQYNPKRDVYILSRYADVREAARNH

DTLSSARGVTFSRGWLPFLPTSDPPAHTRMRKQLAPGMARGALETWRPMV

DQLARELVGGLLTQTPADVVSTVAAPMPMRAITSVLGVDGPDEAAFCRLS

NQAVRITDVALSASGLISLVQGFAGFRRLRALFTHRRDNGLLRECTVLGK

LATHAEQGRLSDDELFFFAVLLLVAGYESTAHMISTLFLTLADYPDQLTL

LAQQPDLIPSAIEEHLRFISPIQNICRTTRVDYSVGQAVIPAGSLVLLAW

GAANRDPRQYEDPDVFRADRNPVGHLAFGSGIHLCPGTQLARMEGQAILR

EIVANIDRIEVVEPPTWTTNANLRGLTRLRVAVTPRVAP

>CYP128A1 (2590142969) Mycobacterium tuberculosis MAL020192

MTATQSPPEPAPDRVRLAGCPLAGTPDVGLTAQDATTALGVPTRRRASSG

GIPVATSMWRDAQTVRTYGPAVAKALALRVAGKARSRLTGRHCRKFMQLT

DFDPFDPAIAADPYPHYRELLAGERVQYNPKRDVYILSRYADVREAARNH

DTLSSARGVTFSRGWLPFLPTSDPPAHTRMRKQLAPGMARGALETWRPMV

DQLARELVGGLLTQTPADVVSTVAAPMPMRAITSVLGVDGPDEAAFCRLS

NQAVRITDVALSASGLISLVQGFAGFRRLRALFTHRRDNGLLRECTVLGK

LATHAEQGRLSDDELFFFAVLLLVAGYESTAHMISTLFLTLADYPDQLTL

LAQQPDLIPSAIEEHLRFISPIQNICRTTRVDYSVGQAVIPAGSLVLLAW

GAANRDPRQYEDPDVFRADRNPVGHLAFGSGIHLCPGTQLARMEGQAILR

EIVANIDRIEVVEPPTWTTNANLRGLTRLRVAVTPRVAP

>CYP128A1 (2603486172) Mycobacterium tuberculosis BTB07-323

MTATQSPPEPAPDRVRLAGCPLAGTPDVGLTAQDATTALGVPTRRRASSG

GIPVATSMWRDAQTVRTYGPAVAKALALRVAGKARSRLTGRHCRKFMQLT

DFDPFDPAIAADPYPHYRELLAGERVQYNPKRDVYILSRYADVREAARNH

DTLSSARGVTFSRGWLPFLPTSDPPAHTRMRKQLAPGMARGALETWRPMV

DQLARELVGGLLTQTPADVVSTVAAPMPMRAITSVLGVDGPDEAAFCRLS

NQAVRITDVALSASGLISLVQGFAGFRRLRALFTHRRDNGLLRECTVLGK

LATHAEQGRLSDDELFFFAVLLLVAGYESTAHMISTLFLTLADYPDQLTL

LAQQPDLIPSAIEEHLRFISPIQNICRTTRVDYSVGQAVIPAGSLVLLAW

GAANRDPRQYEDPDVFRADRNPVGHLAFGSGIHLCPGTQLARMEGQAILR

EIVANIDRIEVVEPPTWTTNANLRGLTRLRVAVTPRVAP

>CYP128A1 (2621852718) Mycobacterium tuberculosis TKK_05MA_0044

MTATQSPPEPAPDRVRLAGCPLAGTPDVGLTAQDATTALGVPTRRRASSG

GIPVATSMWRDAQTVRTYGPAVAKALALRVAGKARSRLTGRHCRKFMQLT

DFDPFDPAIAADPYPHYRELLAGERVQYNPKRDVYILSRYADVREAARNH

DTLSSARGVTFSRGWLPFLPTSDPPAHTRMRKQLAPGMARGALETWRPMV

DQLARELVGGLLTQTPADVVSTVAAPMPMRAITSVLGVDGPDEAAFCRLS

NQAVRITDVALSASGLISLVQGFAGFRRLRALFTHRRDNGLLRECTVLGK

LATHAEQGRLSDDELFFFAVLLLVAGYESTAHMISTLFLTLADYPDQLTL

LAQQPDLIPSAIEEHLRFISPIQNICRTTRVDYSVGQAVIPAGSLVLLAW

GAANRDPRQYEDPDVFRADRNPVGHLAFGSGIHLCPGTQLARMEGQAILR

EIVANIDRIEVVEPPTWTTNANLRGLTRLRVAVTPRVAP

>CYP128A1 (2622425513) Mycobacterium tuberculosis M1288

MTATQSPPEPAPDRVRLAGCPLAGTPDVGLTAQDATTALGVPTRRRASSG

GIPVATSMWRDAQTVRTYGPAVAKALALRVAGKARSRLTGRHCRKFMQLT

DFDPFDPAIAADPYPHYRELLAGERVQYNPKRDVYILSRYADVREAARNH

DTLSSARGVTFSRGWLPFLPTSDPPAHTRMRKQLAPGMARGALETWRPMV

DQLARELVGGLLTQTPADVVSTVAAPMPMRAITSVLGVDGPDEAAFCRLS

NQAVRITDVALSASGLISLVQGFAGFRRLRALFTHRRDNGLLRECTVLGK

LATHAEQGRLSDDELFFFAVLLLVAGYESTAHMISTLFLTLADYPDQLTL

LAQQPDLIPSAIEEHLRFISPIQNICRTTRVDYSVGQAVIPAGSLVLLAW

GAANRDPRQYEDPDVFRADRNPVGHLAFGSGIHLCPGTQLARMEGQAILR

EIVANIDRIEVVEPPTWTTNANLRGLTRLRVAVTPRVAP

>CYP128A1 (2622630906) Mycobacterium tuberculosis M1726

MTATQSPPEPAPDRVRLAGCPLAGTPDVGLTAQDATTALGVPTRRRASSG

GIPVATSMWRDAQTVRTYGPAVAKALALRVAGKARSRLTGRHCRKFMQLT

DFDPFDPAIAADPYPHYRELLAGERVQYNPKRDVYILSRYADVREAARNH

DTLSSSRGVTFSRGWLPFLPTSDPPAHTRMRKQLAPGMARGALETWRPMV

DQLARELVGGLLTQTPADVVSTVAAPMPMRAITSVLGVDGPDEAAFCRLS

NQAVRITDVALSASGLISLVQGFAGFRRLRALFTHRRDNGLLRECTVLGK

LATHAEQGRLSDDELFFFAVLLLVAGYESTAHMISTLFLTLADYPDQLTL

LAQQPDLIPSAIEEHLRFISPIQNICRTTRVDYSVGQAVIPAGSLVLLAW

GAANRDPRQYEDPDVFRADRNPVGHLAFGSGIHLCPGTQLARMEGQAILR

EIVANIDRIEVVEPPTWTTNANLRGLTRLRVAVTPRVAP

>CYP128A1 (2622684438) Mycobacterium tuberculosis M1948

MTATQSPPEPAPDRVRLAGCPLAGTPDVGLTAQDATTALGVPTRRRASSG

GIPVATSMWRDAQTVRTYGPAVAKALALRVAGKARSRLTGRHCRKFMQLT

DFDPFDPAIAADPYPHYRELLAGERVQYNPKRDVYILSRYADVREAARNH

DTLSSARGVTFSRGWLPFLPTSDPPAHTRMRKQLAPGMARGALETWRPMV

DQLARELVGGLLTQTPADVVSTVAAPMPMRAITSVLGVDGPDEAAFCRLS

NQAVRITDVALSASGLISLVQGFAGFRRLRALFTHRRDNGLLRECTVLGK

LATHAEQGRLSDDELFFFAVLLLVAGYESTAHMISTLFLTLADYPDQLTL

LAQQPDLIPSAIEEHLRFISPIQNICRTTRVDYSVGQAVIPAGSLVLLAW

GAANRDPRQYEDPDVFRADRNPVGHLAFGSGIHLCPGTQLARMEGQAILR

EIVANIDRIEVVEPPTWTTNANLRGLTRLRVAVTPRVAP

>CYP128A1 (2632883337) Mycobacterium tuberculosis TKK_04_0105

MTATQSPPEPAPDRVRLAGCPLAGTPDVGLTAQDATTALGVPTRRRASSG

GIPVATSMWRDAQTVRTYGPAVAKALALRVAGKARSRLTGRHCRKFMQLT

DFDPFDPAIAADPYPHYRELLAGERVQYNPKRDVYILSRYADVREAARNH

DTLSSARGVTFSRGWLPFLPTSDPPAHTRMRKQLAPGMARGALETWRPMV

DQLARELVGGLLTQTPADVVSTVAAPMPMRAITSVLGVDGPDEAAFCRLS

NQAVRITDVALSASGLISLVQGFAGFRRLRALFTHRRDNGLLRECTVLGK

LATHAEQGRLSDDELFFFAVLLLVAGYESTAHMISTLFLTLADYPDQLTL

LAQQPDLIPSAIEEHLRFISPIQNICRTTRVDYSVGQAVIPAGSLVLLAW

GAANRDPRQYEDPDVFRADRNPVGHLAFGSGIHLCPGTQLARMEGQAILR

EIVANIDRIEVVEPPTWTTNANLRGLTRLRVAVTPRVAP

>CYP128A1 (2652352732) Mycobacterium bovis B-3222

MTATQSPPEPAPDRVRLAGCPLAGTPDVGLTAQDATTALGVPTRRRASSG

GIPVATSMWRDAQTVRTYGPAVAKALALRVAGKARSRLTGRHCRKFMQLT

DFDPFDPAIAADPYPHYRELLAGERVQYNPKRDVYILSRYADVREAARNH

DTLSSARGVTFSRGWLPFLPTSDPPAHTRMRKQLAPGMARGALETWRPMV

DQLARELVGGLLTQTPADVVSTVAAPMPMRAITSVLGVDGPDEAAFCRLS

NQAVRITDVALSASGLISLVQGFAGFRRLRALFTHRRDNGLLRECTVLGK

LATHAEQGRLSDDELFFFAVLLLVAGYESTAHMISTLFLTLADYPDQLTL

LAQQPDLIPSAIEEHLRFISPIQNICRTTRVDYSVGQAVIPAGSLVLLAW

GAANRDPRQYEDPDVFRADRNPVGHLAFGSGIHLCPGTQLARMEGQAILR

EIVANIDRIEVVEPPTWTTNANLRGLTRLRVAVTPRVAP

>CYP128A1 (2661732655) Mycobacterium tuberculosis A70661

MTATQSPPEPAPDRVRLAGCPLAGTPDVGLTAQDATTALGVPTRRRASSG

GIPVATSMWRDAQTVRTYGPAVAKALALRVAGKARSRLTGRHCRKFMQLT

DFDPFDPAIAADPYPHYRELLAGERVQYNPKRDVYILSRYADVREAARNH

DTLSSARGVTFSRGWLPFLPTSDPPAHTRMRKQLAPGMARGALETWRPMV

DQLARELVGGLLTQTPADVVSTVAAPMPMRAITSVLGVDGPDEAAFCRLS

NQAVRITDVALSASGLISLVQGFAGFRRLRALFTHRRDNGLLRECTVLGK

LATHAEQGRLSDDELFFFAVLLLVAGYESTAHMISTLFLTLADYPDQLTL

LAQQPDLIPSAIEEHLRFISPIQNICRTTRVDYSVGQAVIPAGSLVLLAW

GAANRDPRQYEDPDVFRADRNPVGHLAFGSGIHLCPGTQLARMEGQAILR

EIVANIDRIEVVEPPTWTTNANLRGLTRLRVAVTPRVAP

>CYP128A1 (2675871408) Mycobacterium tuberculosis J09902073

MTATQSPPEPAPDRVRLAGCPLAGTPDVGLTAQDATTALGVPTRRRASSG

GIPVATSMWRDAQTVRTYGPAVAKALALRVAGKARSRLTGRHCRKFMQLT

DFDPFDPAIAADPYPHYRELLAGERVQYNPKRDVYILSRYADVREAARNH

DTLSSARGVTFSRGWLPFLPTSDPPAHTRMRKQLAPGMARGALETWRPMV

DQLARELVGGLLTQTPADVVSTVAAPMPMRAITSVLGVDGPDEAAFCRLS

NQAVRITDVALSASGLISLVQGFAGFRRLRALFTHRRDNGLLRECTVLGK

LATHAEQGRLSDDELFFFAVLLLVAGYESTAHMISTLFLTLADYPDQLTL

LAQQPDLIPSAIEEHLRFISPIQNICRTTRVDYSVGQAVIPAGSLVLLAW

GAANRDPRQYEDPDVFRADRNPVGHLAFGSGIHLCPGTQLARMEGQAILR

EIVANIDRIEVVEPPTWTTNANLRGLTRLRVAVTPRVAP

>CYP128A1 (2742636545) Mycobacterium tuberculosis Aethiop_vetus_230

MTATQSPPEPAPDRVRLAGCPLAGTPDVGLTAQDATTALGVPTRRRASSG

GIPVATSMWRDAQTVRTYGPAVAKALALRVAGKARSRLTGRHCRKFMQLT

DFDPFDPAIAADPYPHYRELLAGERVQYNPKRDVYILSRYADVREAARNH

DTLSSARGVTFSRGWLPFLPTSDPPAHTRMRKQLAPGMARGALETWRPMV

DQLARELVGGLLTQTPADVVSTVAAPMPMRAITSVLGVDGPDEAAFCRLS

NQAVRITDVALSASGLISLVQGFAGFRRLRALFTHRRDNGLLRECTVLGK

LATHAEQGRLSDDELFFFAVLLLVAGYESTAHMISTLFLTLADYPDQLTL

LAQQPDLIPSAIEEHLRFISPIQNICRTTRVDYSVGQAVIPAGSLVLLAW

GAANRDPRQYEDPDVFRADRNPVGHLAFGSGIHLCPGTQLARMEGQAILR

EIVANIDRIEVVEPPTWTTNANLRGLTRLRVAVTPRVAP

>CYP128A1 (2772237870) Mycobacterium holsaticum M7

MPIRSQMRFLKMYGPSLAVGFAHDVSHLVMRKVRRAPVPPGVEVTDFDPL

DPQTAADPYPHYRQLLQSGPVHYNPKRDIFILSRYEDVRAGARTHEVFSS

ADGISYNRMRAPSLLTVDPPRHTQMRKQAQPAFTRGALESWQSTVVQLAR

DHTARLFDNPPVDIVQLLAEPLPTAVIAHILGIADDDLVAFKEWSNETVR

LANANMSLSGMKQGFAGMRAIGHFHHYFTDKLRRGRFLEADTVLGRLVAN

AHDGKLSDDELFFFAFLLLLAGNETTTNMLGTLFLTLSDNPDQLRLLQKR

PDLIPSAIEEQLRYYAPIQGLYRTARSDYVVGSATIPAGARVLLLWGAAN

RDPRQFEDPDAFRVERNPTGHVSFGSGVHLCLGAQLARLEGQSVLREIVA

HVDRIEIVGDPSWRANPTLRGLTKLDVRATRRNGAPDAALADVH

>CYP128B4 (2804354140) Mycobacteroides abscessus abscessus 975

MSRWRSDSAVRQIGAPVVAAVGMNIAAAVRVRRRGYAGWTGAVNTDYDPL

DPATAAQPFDAYRALHAGGRVHYNPKRATFILSRHEDIRAALRDTDAVTS

SQGVTRMKISAPILVLTDGDDHTRLRKQVQPGFTRGAMSDWQGMADQLAK

ELVADVVANPGCDVMERLAVPLPIRMIAHIIGIPPEDVQNFRSWSEDGVG

VINAGVSPAGLRQGLKGVRAIAALRRYFKDQLASGKLKGSDTVLGRLVDN

NEDGKLSDDELFFIAMLLLFAGNETTTNLIGGMFDTLAHAPDQFAMIRDD

PDLIPSAVEEQLRYSAPIQNLYRYTRTDYRVGEVTIPSGSRLLLAFGAAN

RDPEVFEDPDTYRADRNPRNHIAFGYGVHMCIGATLSRMEGQAVLRELTS

QASAIAAAGSATWSTNSSLRGTTYLPIRLTPAR

>CYP128A1 (2576732426) Mycobacterium tuberculosis M2131

MTATQSPPEPAPDRVRLAGCPLAGTPDVGLTAQDATTALGVPTRRRASSG

GIPVATSMWRDAQTVRTYGPAVAKALALRVAGKARSRLTGRHCRKFMQLT

DFDPFDPAIAADPYPHYRELLAGERVQYNPKRDVYILSRYADVREAARNH

DTLSSARGVTFSRGWLPFLPTSDPPAHTRMRKQLAPGMARGALETWRPMV

DQLARELVGGLLTQTPADVVSTVAAPMPMRAITSVLGVDGPDEAAFCRLS

NQAVRITDVALSASGLISLVQGFAGFRRLRALFTHRRDNGLLRECTVLGK

LATHAEQGRLSDDELFFFAVLLLVAGYESTAHMISTLFLTLADYPDQLTL

LAQQPDLIPSAIEEHLRFISPIQNICRTTRVDYSVGQAVIPAGSLVLLAW

GAANRDPRQYEDPDVFRADRNPVGHLAFGSGIHLCPGTQLARMEGQAILR

EIVANIDRIEVVEPPTWTTNANLRGLTRLRVAVTPRVAP

>CYP128A1 (2589141480) Mycobacterium tuberculosis OFXR-2

MTATQSPPEPAPDRVRLAGCPLAGTPDVGLTAQDATTALGVPTRRRASSG

GIPVATSMWRDAQTVRTYGPAVAKALALRVAGKARSRLTGRHCRKFMQLT

DFDPFDPAIAADPYPHYRELLAGERVQYNPKRDVYILSRYADVREAARNH

DTLSSARGVTFSRGWLPFLPTSDPPAHTRMRKQLAPGMARGALETWRPMV

DQLARELVGGLLTQTPADVVSTVAAPMPMRAITSVLGVDGPDEAAFCRLS

NQAVRITDVALSASGLISLVQGFAGFRRLRALFTHRRDNGLLRECTVLGK

LATHAEQGRLSDDELFFFAVLLLVAGYESTAHMISTLFLTLADYPDQLTL

LAQQPDLIPSAIEEHLRFISPIQNICRTTRVDYSVGQAVIPAGSLVLLAW

GAANRDPRQYEDPDVFRADRNPVGHLAFGSGIHLCPGTQLARMEGQAILR

EIVANIDRIEVVEPPTWTTNANLRGLTRLRVAVTPRVAP

>CYP128A1 (2590267595) Mycobacterium tuberculosis KT-0016

MTATQSPPEPAPDRVRLAGCPLAGTPDVGLTAQDATTALGVPTRRRASSG

GIPVATSMWRDAQTVRTYGPAVAKALALRVAGKARSRLTGRHCRKFMQLT

DFDPFDPAIAADPYPHYRELLAGERVQYNPKRDVYILSRYADVREAARNH

DTLSSARGVTFSRGWLPFLPTSDPPAHTRMRKQLAPGMARGALETWRPMV

DQLARELVGGLLTQTPADVVSTVAAPMPMRAITSVLGVDGPDEAAFCRLS

NQAVRITDVALSASGLISLVQGFAGFRRLRALFTHRRDNGLLRECTVLGK

LATHAEQGRLSDDELFFFAVLLLVAGYESTAHMISTLFLTLADYPDQLTL

LAQQPDLIPSAIEEHLRFISPIQNICRTTRVDYSVGQAVIPAGSLVLLAW

GAANRDPRQYEDPDVFRADRNPVGHLAFGSGIHLCPGTQLARMEGQAILR

EIVANIDRIEVVEPPTWTTNANLRGLTRLRVAVTPRVAP

>CYP128A1 (2603225730) Mycobacterium tuberculosis TRUG0114

MTATQSPPEPAPDRVRLAGCPLAGTPDVGLTAQDATTALGVPTRRRASSG

GIPVATSMWRDAQTVRTYGPAVAKALALRVAGKARSRLTGRHCRKFMQLT

DFDPFDPAIAADPYPHYRELLAGERVQYNPKRDVYILSRYADVREAARNH

DTLSSARGVTFSRGWLPFLPTSDPPAHTRMRKQLAPGMARGALETWRPMV

DQLARELVGGLLTQTPADVVSTVAAPMPMRAITSVLGVDGPDEAAFCRLS

NQAVRITDVALSASGLISLVQGFAGFRRLRALFTHRRDNGLLRECTVLGK

LATHAEQGRLSDDELFFFAVLLLVAGYESTAHMISTLFLTLADYPDQLTL

LAQQPDLIPSAIEEHLRFISPIQNICRTTRVDYSVGQAVIPAGSLVLLAW

GAANRDPRQYEDPDVFRADRNPVGHLAFGSGIHLCPGTQLARMEGQAILR

EIVANIDRIEVVEPPTWTTNANLRGLTRLRVAVTPRVAP

>CYP128A1 (2604428532) Mycobacterium tuberculosis TB_RSA143

MTATQSPPEPAPDRVRLAGCPLAGTPDVGLTAQDATTALGVPTRRRASSG

GIPVATSMWRDAQTVRTYGPAVAKALALRVAGKARSRLTGRHCRKFMQLT

DFDPFDPAIAADPYPHYRELLAGERVQYNPKRDVYILSRYADVREAARNH

DTLSSARGVTFSRGWLPFLPTSDPPAHTRMRKQLAPGMARGALETWRPMV

DQLARELVGGLLTQTPADVVSTVAAPMPMRAITSVLGVDGPDEAAFCRLS

NQAVRITDVALSASGLISLVQGFAGFRRLRALFTHRRDNGLLRECTVLGK

LATHAEQGRLSDDELFFFAVLLLVAGYESTAHMISTLFLTLADYPDQLTL

LAQQPDLIPSAIEEHLRFISPIQNICRTTRVDYSVGQAVIPAGSLVLLAW

GAANRDPRQYEDPDVFRADRNPVGHLAFGSGIHLCPGTQLARMEGQAILR

EIVANIDRIEVVEPPTWTTNANLRGLTRLRVAVTPRVAP

>CYP128A1 (2621707604) Mycobacterium tuberculosis 9956

MTATQSPPEPAPDRVRLAGCPLAGTPDVGLTAQDATTALGVPTRRRASSG

GIPVATSMWRDAQTVRTYGPAVAKALALRVAGKARSRLTGRHCRKFMQLT

DFDPFDPAIAADPYPHYRELLAGERVQYNPKRDVYILSRYADVREAARNH

DTLSSARGVTFSRGWLPFLPTSDPPAHTRMRKQLAPGMARGALETWRPMV

DQLARELVGGLLTQTPADVVSTVAAPMPMRAITSVLGVDGPDEAAFCRLS

NQAVRITDVALSASGLISLVQGFAGFRRLRALFTHRRDNGLLRECTVLGK

LATHAEQGRLSDDELFFFAVLLLVAGYESTAHMISTLFLTLADYPDQLTL

LAQQPDLIPSAIEEHLRFISPIQNICRTTRVDYSVGQAVIPAGSLVLLAW

GAANRDPRQYEDPDVFRADRNPVGHLAFGSGIHLCPGTQLARMEGQAILR

EIVANIDRIEVVEPPTWTTNANLRGLTRLRVAVTPRVAP

>CYP128A1 (2621790186) Mycobacterium tuberculosis TKK_04_0153

MTATQSPPEPAPDRVRLAGCPLAGTPDVGLTAQDATTALGVPTRRRASSG

GIPVATSMWRDAQTVRTYGPAVAKALALRVAGKARSRLTGRHCRKFMQLT

DFDPFDPAIAADPYPHYRELLAGERVQYNPKRDVYILSRYADVREAARNH

DTLSSARGVTFSRGWLPFLPTSDPPAHTRMRKQLAPGMARGALETWRPMV

DQLARELVGGLLTQTPADVVSTVAAPMPMRAITSVLGVDGPDEAAFCRLS

NQAVRITDVALSASGLISLVQGFAGFRRLRALFTHRRDNGLLRECTVLGK

LATHAEQGRLSDDELFFFAVLLLVAGYESTAHMISTLFLTLADYPDQLTL

LAQQPDLIPSAIEEHLRFISPIQNICRTTRVDYSVGQAVIPAGSLVLLAW

GAANRDPRQYEDPDVFRADRNPVGHLAFGSGIHLCPGTQLARMEGQAILR

EIVANIDRIEVVEPPTWTTNANLRGLTRLRVAVTPRVAP

>CYP128A1 (2621891042) Mycobacterium tuberculosis TKK_05SA_0011

MTATQSPPEPAPDRVRLAGCPLAGTPDVGLTAQDATTALGVPTRRRASSG

GIPVATSMWRDAQTVRTYGPAVAKALALRVAGKARSRLTGRHCRKFMQLT

DFDPFDPAIAADPYPHYRELLAGERVQYNPKRDVYILSRYADVREAARNH

DTLSSARGVTFSRGWLPFLPTSDPPAHTRMRKQLAPGMARGALETWRPMV

DQLARELVGGLLTQTPADVVSTVAAPMPMRAITSVLGVDGPDEAAFCRLS

NQAVRITDVALSASGLISLVQGFAGFRRLRALFTHRRDNGLLRECTVLGK

LATHAEQGRLSDDELFFFAVLLLVAGYESTAHMISTLFLTLADYPDQLTL

LAQQPDLIPSAIEEHLRFISPIQNICRTTRVDYSVGQAVIPAGSLVLLAW

GAANRDPRQYEDPDVFRADRNPVGHLAFGSGIHLCPGTQLARMEGQAILR

EIVANIDRIEVVEPPTWTTNANLRGLTRLRVAVTPRVAP

>CYP128A1 (2622101098) Mycobacterium tuberculosis MD16732

MTATQSPPEPAPDRVRLAGCPLAGTPDVGLTAQDATTALGVPTRRRASSG

GIPVATSMWRDAQTVRTYGPAVAKALALRVAGKARSRLTGRHCRKFMQLT

DFDPFDPAIAADPYPHYRELLAGERVQYNPKRDVYILSRYADVREAARNH

DTLSSARGVTFSRGWLPFLPTSDPPAHTRMRKQLAPGMARGALETWRPMV

DQLARELVGGLLTQTPADVVSTVAAPMPMRAITSVLGVDGPDEAAFCRLS

NQAVRITDVALSASGLISLVQGFAGFRRLRALFTHRRDNGLLRECTVLGK

LATHAEQGRLSDDELFFFAVLLLVAGYESTAHMISTLFLTLADYPDQLTL

LAQQPDLIPSAIEEHLRFISPIQNICRTTRVDYSVGQAVIPAGSLVLLAW

GAANRDPRQYEDPDVFRADRNPVGHLAFGSGIHLCPGTQLARMEGQAILR

EIVANIDRIEVVEPPTWTTNANLRGLTRLRVAVTPRVAP

>CYP128A1 (2660523600) Mycobacterium tuberculosis 1700745

MTATQSPPEPAPDRVRLAGCPLAGTPDVGLTAQDATTALGVPTRRRASSG

GIPVATSMWRDAQTVRTYGPAVAKALALRVAGKARSRLTGRHCRKFMQLT

DFDPFDPAIAADPYPHYRELLAGERVQYNPKRDVYILSRYADVREAARNH

DTLSSARGVTFSRGWLPFLPTSDPPAHTRMRKQLAPGMARGALETWRPMV

DQLARELVGGLLTQTPADVVSTVAAPMPMRAITSVLGVDGPDEAAFCRLS

NQAVRITDVALSASGLISLVQGFAGFRRLRALFTHRRDNGLLRECTVLGK

LATHAEQGRLSDDELFFFAVLLLVAGYESTAHMISTLFLTLADYPDQLTL

LAQQPDLIPSAIEEHLRFISPIQNICRTTRVDYSVGQAVIPAGSLVLLAW

GAANRDPRQYEDPDVFRADRNPVGHLAFGSGIHLCPGTQLARMEGQAILR

EIVANIDRIEVVEPPTWTTNANLRGLTRLRVAVTPRVAP

>CYP128A1 (2662945341) Mycobacterium tuberculosis 402138

MTATQSPPEPAPDRVRLAGCPLAGTPDVGLTAQDATTALGVPTRRRASSG

GIPVATSMWRDAQTVRTYGPAVAKALALRVAGKARSRLTGRHCRKFMQLT

DFDPFDPAIAADPYPHYRELLAGERVQYNPKRDVYILSRYADVREAARNH

DTLSSARGVTFSRGWLPFLPTSDPPAHTRMRKQLAPGMARGALETWRPMV

DQLARELVGGLLTQTPADVVSTVAAPMPMRAITSVLGVDGPDEAAFCRLS

NQAVRITDVALSASGLISLVQGFAGFRRLRALFTHRRDNGLLRECTVLGK

LATHAEQGRLSDDELFFFAVLLLVAGYESTAHMISTLFLTLADYPDQLTL

LAQQPDLIPSAIEEHLRFISPIQNICRTTRVDYSVGQAVIPAGSLVLLAW

GAANRDPRQYEDPDVFRADRNPVGHLAFGSGIHLCPGTQLARMEGQAILR

EIVANIDRIEVVEPPTWTTNANLRGLTRLRVAVTPRVAP

>CYP128A1 (2669035220) Mycobacterium tuberculosis 301310

MTATQSPPEPAPDRVRLAGCPLAGTPDVGLTAQDATTALGVPTRRRASSG

GIPVATSMWRDAQTVRTYGPAVAKALALRVAGKARSRLTGRHCRKFMQLT

DFDPFDPAIAADPYPHYRELLAGERVQYNPKRDVYILSRYADVREAARNH

DTLSSARGVTFSRGWLPFLPTSDPPAHTRMRKQLAPGMARGALETWRPMV

DQLARELVGGLLTQTPADVVSTVAAPMPMRAITSVLGVDGPDEAAFCRLS

NQAVRITDVALSASGLISLVQGFAGFRRLRALFTHRRDNGLLRECTVLGK

LATHAEQGRLSDDELFFFAVLLLVAGYESTAHMISTLFLTLADYPDQLTL

LAQQPDLIPSAIEEHLRFISPIQNICRTTRVDYSVGQAVIPAGSLVLLAW

GAANRDPRQYEDPDVFRADRNPVGHLAFGSGIHLCPGTQLARMEGQAILR

EIVANIDRIEVVEPPTWTTNANLRGLTRLRVAVTPRVAP

>CYP128A1 (2675372419) Mycobacterium tuberculosis J09400698

MTATQSPPEPAPDRVRLAGCPLAGTPDVGLTAQDATTALGVPTRRRASSG

GIPVATSMWRDAQTVRTYGPAVAKALALRVAGKARSRLTGRHCRKFMQLT

DFDPFDPAIAADPYPHYRELLAGERVQYNPKRDVYILSRYADVREAARNH

DTLSSARGVTFSRGWLPFLPTSDPPAHTRMRKQLAPGMARGALETWRPMV

DQLARELVGGLLTQTPADVVSTVAAPMPMRAITSVLGVDGPDEAAFCRLS

NQAVRITDVALSASGLISLVQGFAGFRRLRALFTHRRDNGLLRECTVLGK

LATHAEQGRLSDDELFFFAVLLLVAGYESTAHMISTLFLTLADYPDQLTL

LAQQPDLIPSAIEEHLRFISPIQNICRTTRVDYSVGQAVIPAGSLVLLAW

GAANRDPRQYEDPDVFRADRNPVGHLAFGSGIHLCPGTQLARMEGQAILR

EIVANIDRIEVVEPPTWTTNANLRGLTRLRVAVTPRVAP

>CYP128A1 (2688158546) Mycobacterium tuberculosis 37004

MTATQSPPEPAPDRVRLAGCPLAGTPDVGLTAQDATTALGVPTRRRASSG

GIPVATSMWRDAQTVRTYGPAVAKALALRVAGKARSRLTGRHCRKFMQLT

DFDPFDPAIAADPYPHYRELLAGERVQYNPKRDVYILSRYADVREAARNH

DTLSSARGVTFSRGWLPFLPTSDPPAHTRMRKQLAPGMARGALETWRPMV

DQLARELVGGLLTQTPADVVSTVAAPMPMRAITSVLGVDGPDEAAFCRLS

NQAVRITDVALSASGLISLVQGFAGFRRLRALFTHRRDNGLLRECTVLGK

LATHAEQGRLSDDELFFFAVLLLVAGYESTAHMISTLFLTLADYPDQLTL

LAQQPDLIPSAIEEHLRFISPIQNICRTTRVDYSVGQAVIPAGSLVLLAW

GAANRDPRQYEDPDVFRADRNPVGHLAFGSGIHLCPGTQLARMEGQAILR

EIVANIDRIEVVEPPTWTTNANLRGLTRLRVAVTPRVAP

>CYP128A1 (2674730594) Mycobacterium tuberculosis S00600143

MTATQSPPEPAPDRVRLAGCPLAGTPDVGLTAQDATTALGVPTRRRASSG

GIPVATSMWRDAQTVRTYGPAVAKALALRVAGKARSRLTGRHCRKFMQLT

DFDPFDPAIAADPYPHYRELLAGERVQYNPKRDVYILSRYADVREAARNH

DTLSSARGVTFSRGWLPFLPTSDPPAHTRMRKQLAPGMARGALETWRPMV

DQLARELVGGLLTQTPADVVSTVAAPMPMRAITSVLGVDGPDEAAFCRLS

NQAVRITDVALSASGLISLVQGFAGFRRLRALFTHRRDNGLLRECTVLGK

LATHAEQGRLSDDELFFFAVLLLVAGYESTAHMISTLFLTLADYPDQLTL

LAQQPDLIPSAIEEHLRFISPIQNICRTTRVDYSVGQAVIPAGSLVLLAW

GAANRDPRQYEDPDVFRADRNPVGHLAFGSGIHLCPGPSWRAWRVRRSCA

RSSPISTE

>CYP128B1 (2684705710) Mycobacterium lentiflavum CSUR P1491

VNTDYDPQNPVTAAQPFDAYRGLHRSGRVHYNPRRATWVLSRLNDVRAAL

RDTDRVTSTQGVTRLRISAPLAVLTDGDEHTRLRKQVQPGFSKGAMDSWQ

AMTERLAAELVSDLLANPGGDVVHGLAVPMPIRLIAQILGVPERDVTDFR

RWSEKGLGIMDLTPTPAGLVDAAKSVSAIAALRRYFIRQFAAGNLKGSGT

VLGRLLEHNTDGSLTDNQLVLIAIHLLIAGNETTTNLLGGMFDTLARNPE

QYELIRANPDLIPAAVEEQLRITTPIQNLYRYTRADYRLGDVTIPTGSRI

LLSFGAANRDPAVFENPDEYRADRNPRMHVAFGYGAHMCLGAPLARMEAQ

AVLRELVTHVARISPEAPTTWSTHSSLRGPTHLPVRLTPA

>CYP128A1 (2575601026) Mycobacterium tuberculosis TKK_05SA_0012

MTATQSPPEPAPDRVRLAGCPLAGTPDVGLTAQDATTALGVPTRRRASSG

GIPVATSMWRDAQTVRTYGPAVAKALALRVAGKARSRLTGRHCRKFMQLT

DFDPFDPAIAADPYPHYRELLAGERVQYNPKRDVYILSRYADVREAARNH

DTLSSARGVTFSRGWLPFLPTSDPPAHTRMRKQLAPGMARGALETWRPMV

DQLARELVGGLLTQTPADVVSTVAAPMPMRAITSVLGVDGPDEAAFCRLS

NQAVRITDVALSASGLISLVQGFAGFRRLRALFTHRRDNGLLRECTVLGK

LATHAEQGRLSDDELFFFAVLLLVAGYESTAHMISTLFLTLADYPDQLTL

LAQQPDLIPSAIEEHLRFISPIQNICRTTRVDYSVGQAVIPAGSLVLLAW

GAANRDPRQYEDPDVFRADRNPVGHLAFGSGIHLCPGTQLARMEGQAILR

EIVANIDRIEVVEPPTWTTNANLRGLTRLRVAVTPRVAP

>CYP128A1 (2581564120) Mycobacterium africanum MAL020148

MTATQSPPEPAPDRVRLAGCPLAGTPDVGLTAQDATTALGVPTRRRASSG

GIPVATSMWRDAQTVRTYGPAVAKALALRVAGKARSRLTGRHCRKFMQLT

DFDPFDPAIAADPYPHYRELLAGERVQYNPKRDVYILSRYADVREAARNH

DTLSSARGVTFSRGWLPFLPTSDPPAHTRMRKQLAPGMARGALETWRPMV

DQLARELVGGLLTQTPADVVSTVAAPMPMRAITSVLGVDGPDEAAFCRLS

NQAVRITDVALSASGLISLVQGFAGFRRLRALFTHRRDNGLLRECTVLGK

LATHAEQGRLSDDELFFFAVLLLVAGYESTAHMISTLFLTLADYPDQLTL

LAQQPDLIPSAIEEHLRFISPIQNICRTTRVDYSVGQAVIPAGSLVLLAW

GAANRDPRQYEDPDVFRADRNPVGHLAFGSGIHLCPGTQLARMEGQAILR

EIVANIDRIEVVEPPTWTTNANLRGLTRLRVAVTPRVAP

>CYP128A1 (2589081467) Mycobacterium tuberculosis TBR44

MTATQSPPEPAPDRVRLAGCPLAGTPDVGLTAQDATTALGVPTRRRASSG

GIPVATSMWRDAQTVRTYGPAVAKALALRVAGKARSRLTGRHCRKFMQLT

DFDPFDPAIAADPYPHYRELLAGERVQYNPKRDVYILSRYADVREAARNH

DTLSSARGVTFSRGWLPFLPTSDPPAHTRMRKQLAPGMARGALETWRPMV

DQLARELVGGLLTQTPADVVSTVAAPMPMRAITSVLGVDGPDEAAFCRLS

NQAVRITDVALSASGLISLVQGFAGFRRLRALFTHRRDNGLLRECTVLGK

LATHAEQGRLSDDELFFFAVLLLVAGYESTAHMISTLFLTLADYPDQLTL

LAQQPDLIPSAIEEHLRFISPIQNICRTTRVDYSVGQAVIPAGSLVLLAW

GAANRDPRQYEDPDVFRADRNPVGHLAFGSGIHLCPGTQLARMEGQAILR

EIVANIDRIEVVEPPTWTTNANLRGLTRLRVAVTPRVAP

>CYP128A1 (2590041188) Mycobacterium tuberculosis MAL010105

MTATQSPPEPAPDRVRLAGCPLAGTPDVGLTAQDATTALGVPTRRRASSG

GIPVATSMWRDAQTVRTYGPAVAKALALRVAGKARSRLTGRHCRKFMQLT

DFDPFDPAIAADPYPHYRELLAGERVQYNPKRDVYILSRYADVREAARNH

DTLSSARGVTFSRGWLPFLPTSDPPAHTRMRKQLAPGMARGALETWRPMV

DQLARELVGGLLTQTPADVVSTVAAPMPMRAITSVLGVDGPDEAAFCRLS

NQAVRITDVALSASGLISLVQGFAGFRRLRALFTHRRDNGLLRECTVLGK

LATHAEQGRLSDDELFFFAVLLLVAGYESTAHMISTLFLTLADYPDQLTL

LAQQPDLIPSAIEEHLRFISPIQNICRTTRVDYSVGQAVIPAGSLVLLAW

GAANRDPRQYEDPDVFRADRNPVGHLAFGSGIHLCPGTQLARMEGQAILR

EIVANIDRIEVVEPPTWTTNANLRGLTRLRVAVTPRVAP

>CYP128A1 (2603180777) Mycobacterium tuberculosis BTB13-127

MTATQSPPEPAPDRVRLAGCPLAGTPDVGLTAQDATTALGVPTRRRASSG

GIPVATSMWRDAQTVRTYGPAVAKALALRVAGKARSRLTGRHCRKFMQLT

DFDPFDPAIAADPYPHYRELLAGERVQYNPKRDVYILSRYADVREAARNH

DTLSSARGVTFSRGWLPFLPTSDPPAHTRMRKQLAPGMARGALETWRPMV

DQLARELVGGLLTQTPADVVSTVAAPMPMRAITSVLGVDGPDEAAFCRLS

NQAVRITDVALSASGLISLVQGFAGFRRLRALFTHRRDNGLLRECTVLGK

LATHAEQGRLSDDELFFFAVLLLVAGYESTAHMISTLFLTLADYPDQLTL

LAQQPDLIPSAIEEHLRFISPIQNICRTTRVDYSVGQAVIPAGSLVLLAW

GAANRDPRQYEDPDVFRADRNPVGHLAFGSGIHLCPGTQLARMEGQAILR

EIVANIDRIEVVEPPTWTTNANLRGLTRLRVAVTPRVAP

>CYP128A1 (2603881612) Mycobacterium tuberculosis BTB10-142

MTATQSPPEPAPDRVRLAGCPLAGTPDVGLTAQDATTALGVPTRRRASSG

GIPVATSMWRDAQTVRTYGPAVAKALALRVAGKARSRLTGRHCRKFMQLT

DFDPFDPAIAADPYPHYRELLAGERVQYNPKRDVYILSRYADVREAARNH

DTLSSARGVTFSRGWLPFLPTSDPPAHTRMRKQLAPGMARGALETWRPMV

DQLARELVGGLLTQTPADVVSTVAAPMPMRAITSVLGVDGPDEAAFCRLS

NQAVRITDVALSASGLISLVQGFAGFRRLRALFTHRRDNGLLRECTVLGK

LATHAEQGRLSDDELFFFAVLLLVAGYESTAHMISTLFLTLADYPDQLTL

LAQQPDLIPSAIEEHLRFISPIQNICRTTRVDYSVGQAVIPAGSLVLLAW

GAANRDPRQYEDPDVFRADRNPVGHLAFGSGIHLCPGTQLARMEGQAILR

EIVANIDRIEVVEPPTWTTNANLRGLTRLRVAVTPRVAP

>CYP128A1 (2604965100) Mycobacterium tuberculosis BTB10-305

MTATQSPPEPAPDRVRLAGCPLAGTPDVGLTAQDATTALGVPTRRRASSG

GIPVATSMWRDAQTVRTYGPAVAKALALRVAGKARSRLTGRHCRKFMQLT

DFDPFDPAIAADPYPHYRELLAGERVQYNPKRDVYILSRYADVREAARNH

DTLSSARGVTFSRGWLPFLPTSDPPAHTRMRKQLAPGMARGALETWRPMV

DQLARELVGGLLTQTPADVVSTVAAPMPMRAITSVLGVDGPDEAAFCRLS

NQAVRITDVALSASGLISLVQGFAGFRRLRALFTHRRDNGLLRECTVLGK

LATHAEQGRLSDDELFFFAVLLLVAGYESTAHMISTLFLTLADYPDQLTL

LAQQPDLIPSAIEEHLRFISPIQNICRTTRVDYSVGQAVIPAGSLVLLAW

GAANRDPRQYEDPDVFRADRNPVGHLAFGSGIHLCPGTQLARMEGQAILR

EIVANIDRIEVVEPPTWTTNANLRGLTRLRVAVTPRVAP

>CYP128A1 (2605770094) Mycobacterium tuberculosis TB_RSA75

MTATQSPPEPAPDRVRLAGCPLAGTPDVGLTAQDATTALGVPTRRRASSG

GIPVATSMWRDAQTVRTYGPAVAKALALRVAGKARSRLTGRHCRKFMQLT

DFDPFDPAIAADPYPHYRELLAGERVQYNPKRDVYILSRYADVREAARNH

DTLSSARGVTFSRGWLPFLPTSDPPAHTRMRKQLAPGMARGALETWRPMV

DQLARELVGGLLTQTPADVVSTVAAPMPMRAITSVLGVDGPDEAAFCRLS

NQAVRITDVALSASGLISLVQGFAGFRRLRALFTHRRDNGLLRECTVLGK

LATHAEQGRLSDDELFFFAVLLLVAGYESTAHMISTLFLTLADYPDQLTL

LAQQPDLIPSAIEEHLRFISPIQNICRTTRVDYSVGQAVIPAGSLVLLAW

GAANRDPRQYEDPDVFRADRNPVGHLAFGSGIHLCPGTQLARMEGQAILR

EIVANIDRIEVVEPPTWTTNANLRGLTRLRVAVTPRVAP

>CYP128A1 (2621681844) Mycobacterium tuberculosis 6605

MTATQSPPEPAPDRVRLAGCPLAGTPDVGLTAQDATTALGVPTRRRASSG

GIPVATSMWRDAQTVRTYGPAVAKALALRVAGKARSRLTGRHCRKFMQLT

DFDPFDPAIAADPYPHYRELLAGERVQYNPKRDVYILSRYADVREAARNH

DTLSSARGVTFSRGWLPFLPTSDPPAHTRMRKQLAPGMARGALETWRPMV

DQLARELVGGLLTQTPADVVSTVAAPMPMRAITSVLGVDGPDEAAFCRLS

NQAVRITDVALSASGLISLVQGFAGFRRLRALFTHRRDNGLLRECTVLGK

LATHAEQGRLSDDELFFFAVLLLVAGYESTAHMISTLFLTLADYPDQLTL

LAQQPDLIPSAIEEHLRFISPIQNICRTTRVDYSVGQAVIPAGSLVLLAW

GAANRDPRQYEDPDVFRADRNPVGHLAFGSGIHLCPGTQLARMEGQAILR

EIVANIDRIEVVEPPTWTTNANLRGLTRLRVAVTPRVAP

>CYP128A1 (2659774209) Mycobacterium tuberculosis 402248

MTATQSPPEPAPDRVRLAGCPLAGTPDVGLTAQDATTALGVPTRRRASSG

GIPVATSMWRDAQTVRTYGPAVAKALALRVAGKARSRLTGRHCRKFMQLT

DFDPFDPAIAADPYPHYRELLAGERVQYNPKRDVYILSRYADVREAARNH

DTLSSARGVTFSRGWLPFLPTSDPPAHTRMRKQLAPGMARGALETWRPMV

DQLARELVGGLLTQTPADVVSTVAAPMPMRAITSVLGVDGPDEAAFCRLS

NQAVRITDVALSASGLISLVQGFAGFRRLRALFTHRRDNGLLRECTVLGK

LATHAEQGRLSDDELFFFAVLLLVAGYESTAHMISTLFLTLADYPDQLTL

LAQQPDLIPSAIEEHLRFISPIQNICRTTRVDYSVGQAVIPAGSLVLLAW

GAANRDPRQYEDPDVFRADRNPVGHLAFGSGIHLCPGTQLARMEGQAILR

EIVANIDRIEVVEPPTWTTNANLRGLTRLRVAVTPRVAP

>CYP128A1 (2670953599) Mycobacterium tuberculosis 302434

MTATQSPPEPAPDRVRLAGCPLAGTPDVGLTAQDATTALGVPTRRRASSG

GIPVATSMWRDAQTVRTYGPAVAKALALRVAGKARSRLTGRHCRKFMQLT

DFDPFDPAIAADPYPHYRELLAGERVQYNPKRDVYILSRYADVREAARNH

DTLSSARGVTFSRGWLPFLPTSDPPAHTRMRKQLAPGMARGALETWRPMV

DQLARELVGGLLTQTPADVVSTVAAPMPMRAITSVLGVDGPDEAAFCRLS

NQAVRITDVALSASGLISLVQGFAGFRRLRALFTHRRDNGLLRECTVLGK

LATHAEQGRLSDDELFFFAVLLLVAGYESTAHMISTLFLTLADYPDQLTL

LAQQPDLIPSAIEEHLRFISPIQNICRTTRVDYSVGQAVIPAGSLVLLAW

GAANRDPRQYEDPDVFRADRNPVGHLAFGSGIHLCPGTQLARMEGQAILR

EIVANIDRIEVVEPPTWTTNANLRGLTRLRVAVTPRVAP

>CYP128A1 (2688582655) Mycobacterium tuberculosis BT1

MTATQSPPEPAPDRVRLAGCPLAGTPDVGLTAQDATTALGVPTRRRASSG

GIPVATSMWRDAQTVRTYGPAVAKALALRVAGKARSRLTGRHCRKFMQLT

DFDPFDPAIAADPYPHYRELLAGERVQYNPKRDVYILSRYADVREAARNH

DTLSSARGVTFSRGWLPFLPTSDPPAHTRMRKQLAPGMARGALETWRPMV

DQLARELVGGLLTQTPADVVSTVAAPMPMRAITSVLGVDGPDEAAFCRLS

NQAVRITDVALSASGLISLVQGFAGFRRLRALFTHRRDNGLLRECTVLGK

LATHAEQGRLSDDELFFFAVLLLVAGYESTAHMISTLFLTLADYPDQLTL

LAQQPDLIPSAIEEHLRFISPIQNICRTTRVDYSVGQAVIPAGSLVLLAW

GAANRDPRQYEDPDVFRADRNPVGHLAFGSGIHLCPGTQLARMEGQAILR

EIVANIDRIEVVEPPTWTTNANLRGLTRLRVAVTPRVAP

>CYP128A1 (2548033846) Mycobacterium tuberculosis BTB05-552

MTATQSPPEPAPDRVRLAGCPLAGTPDVGLTAQDATTALGVPTRRRASSG

GIPVATSMWRDAQTVRTYGPAVAKALALRVAGKARSRLTGRHCRKFMQLT

DFDPFDPAIAADPYPHYRELLAGERVQDNPKRDVYILSRYADVREAARNH

DTLSSARGVTFSRGWLPFLPTSDPPAHTRMRKQLAPGMARGALETWRPMV

DQLARELVGGLLTQTPADVVSTVAAPMPMRAITSVLGVDGPDEAAFCRLS

NQAVRITDVALSASGLISLVQGFAGFRRLRALFTHRRDNGLLRECTVLGK

LATHAEQGRLSDDELFFFAVLLLVAGYESTAHMISTLFLTLADYPDQLTL

LAQQPDLIPSAIEEHLRFISPIQNICRTTRVDYSVGQAVIPAGSLVLLAW

GAANRDPRQYEDPDVFRADRNPVGHLAFGSGIHLCPGTQLARMEGQAILR

EIVANIDRIEVVEPPTWTTNANLRGLTRLRVAVTPRVAP

>CYP128A1 (2548038083) Mycobacterium tuberculosis BTB05-559

MTATQSPPEPAPDRVRLAGCPLAGTPDVGLTAQDATTALGVPTRRRASSG

GIPVATSMWRDAQTVRTYGPAVAKALALRVAGKARSRLTGRHCRKFMQLT

DFDPFDPAIAADPYPHYRELLAGERVQDNPKRDVYILSRYADVREAARNH

DTLSSARGVTFSRGWLPFLPTSDPPAHTRMRKQLAPGMARGALETWRPMV

DQLARELVGGLLTQTPADVVSTVAAPMPMRAITSVLGVDGPDEAAFCRLS

NQAVRITDVALSASGLISLVQGFAGFRRLRALFTHRRDNGLLRECTVLGK

LATHAEQGRLSDDELFFFAVLLLVAGYESTAHMISTLFLTLADYPDQLTL

LAQQPDLIPSAIEEHLRFISPIQNICRTTRVDYSVGQAVIPAGSLVLLAW

GAANRDPRQYEDPDVFRADRNPVGHLAFGSGIHLCPGTQLARMEGQAILR

EIVANIDRIEVVEPPTWTTNANLRGLTRLRVAVTPRVAP

>CYP128A1 (2567231942) Mycobacterium kansasii 824

MQFTDFDPFDPVTAADPYPHYRELLAGERVQYNPKRDVYIVSRYTDVREA

ARNHHALSSAGGVTFSRGCLPFLPTSDPPTHTRLRKQLAPSMARGAVESW

RPMIDRLAHELVAELLTRTPADVVATVAAPLPMRTITAVLGVPGPDEGMF

CRLSNQAARITDVNLSASGLVSLAQGFTGFRRLRALFTQRRATGLLGEHT

VFGRLAAHAEHGRLSDDELFFFAVLLLVAGYESTAHMISTLFLTLTEFPD

QLSLLARRPDLIPSAIEEQLRFVSPIQNICRTTRVDYPVGGAVIPAGSLV

LLAWGAANRDPRQYDDPDVFRADRNPTGHLAFGSGIHLCPGTQLARMEGH

AVLREIVTNIDRIDVVEPPAWTTNANLRGPTRLRVAVTPRASR

>CYP128A1 (2605659639) Mycobacterium tuberculosis BTB11-207

MTATQSPPEPAPDRVRLAGCPLAGTPDVGLTAQDATTALGVPTRRRASSG

GIPVATSMWRDAQTVRTYGPAVAKALALRVAGKARSRLTGRHCRKFMQLT

DFDPFDPAIAADPYPHYRELLAGERVQYNPKRDVYILSRYADVREAARNH

DTLSSARGVTFSRGWLPFLPTSDPPAHTRMRKQLAPGMARGALETWRPMV

DQLARELVGGLLTQTPADVVSTVAAPMPMRAITSVLGVDGPDEAAFCRLS

NQAVRITDVALSASGLISLVQGFAGFRRLRALFTHRRDNGLLRECTVLGK

LATHAEQGRLSDDELFFFAVLLLVAGYESTAHMISTLFLTLADYPDQLTL

LAQQPDLIPSAIEEHLRFISPIQNICRTTRVDYSVGQAVIPAGSLVLLAW

GAANRDPRQYEDPDVFRADRNPVGHLAFGSGIHLCPGTQLARMEGQAILR

EIVANIDRIEVVEPPTWTTNANLRGLTRLRVAVTPRVAP

>CYP128A1 (2605741622) Mycobacterium tuberculosis TB_RSA35

MTATQSPPEPAPDRVRLAGCPLAGTPDVGLTAQDATTALGVPTRRRASSG

GIPVATSMWRDAQTVRTYGPAVAKALALRVAGKARSRLTGRHCRKFMQLT

DFDPFDPAIAADPYPHYRELLAGERVQYNPKRDVYILSRYADVREAARNH

DTLSSARGVTFSRGWLPFLPTSDPPAHTRMRKQLAPGMARGALETWRPMV

DQLARELVGGLLTQTPADVVSTVAAPMPMRAITSVLGVDGPDEAAFCRLS

NQAVRITDVALSASGLISLVQGFAGFRRLRALFTHRRDNGLLRECTVLGK

LATHAEQGRLSDDELFFFAVLLLVAGYESTAHMISTLFLTLADYPDQLTL

LAQQPDLIPSAIEEHLRFISPIQNICRTTRVDYSVGQAVIPAGSLVLLAW

GAANRDPRQYEDPDVFRADRNPVGHLAFGSGIHLCPGTQLARMEGQAILR

EIVANIDRIEVVEPPTWTTNANLRGLTRLRVAVTPRVAP

>CYP128A1 (2630355243) Mycobacterium tuberculosis TKK_04_0123

MTATQSPPEPAPDRVRLAGCPLAGTPDVGLTAQDATTALGVPTRRRASSG

GIPVATSMWRDAQTVRTYGPAVAKALALRVAGKARSRLTGRHCRKFMQLT

DFDPFDPAIAADPYPHYRELLAGERVQYNPKRDVYILSRYADVREAARNH

DTLSSARGVTFSRGWLPFLPTSDPPAHTRMRKQLAPGMARGALETWRPMV

DQLARELVGGLLTQTPADVVSTVAAPMPMRAITSVLGVDGPDEAAFCRLS

NQAVRITDVALSASGLISLVQGFAGFRRLRALFTHRRDNGLLRECTVLGK

LATHAEQGRLSDDELFFFAVLLLVAGYESTAHMISTLFLTLADYPDQLTL

LAQQPDLIPSAIEEHLRFISPIQNICRTTRVDYSVGQAVIPAGSLVLLAW

GAANRDPRQYEDPDVFRADRNPVGHLAFGSGIHLCPGTQLARMEGQAILR

EIVANIDRIEVVEPPTWTTNANLRGLTRLRVAVTPRVAP

>CYP128A1 (2665903179) Mycobacterium tuberculosis 402495

MTATQSPPEPAPDRVRLAGCPLAGTPDVGLTAQDATTALGVPTRRRASSG

GIPVATSMWRDAQTVRTYGPAVAKALALRVAGKARSRLTGRHCRKFMQLT

DFDPFDPAIAADPYPHYRELLAGERVQYNPKRDVYILSRYADVREAARNH

DTLSSARGVTFSRGWLPFLPTSDPPAHTRMRKQLAPGMARGALETWRPMV

DQLARELVGGLLTQTPADVVSTVAAPMPMRAITSVLGVDGPDEAAFCRLS

NQAVRITDVALSASGLISLVQGFAGFRRLRALFTHRRDNGLLRECTVLGK

LATHAEQGRLSDDELFFFAVLLLVAGYESTAHMISTLFLTLADYPDQLTL

LAQQPDLIPSAIEEHLRFISPIQNICRTTRVDYSVGQAVIPAGSLVLLAW

GAANRDPRQYEDPDVFRADRNPVGHLAFGSGIHLCPGTQLARMEGQAILR

EIVANIDRIEVVEPPTWTTNANLRGLTRLRVAVTPRVAP

>CYP128A1 (2668688295) Mycobacterium tuberculosis 200253

MTATQSPPEPAPDRVRLAGCPLAGTPDVGLTAQDATTALGVPTRRRASSG

GIPVATSMWRDAQTVRTYGPAVAKALALRVAGKARSRLTGRHCRKFMQLT

DFDPFDPAIAADPYPHYRELLAGERVQYNPKRDVYILSRYADVREAARNH

DTLSSARGVTFSRGWLPFLPTSDPPAHTRMRKQLAPGMARGALETWRPMV

DQLARELVGGLLTQTPADVVSTVAAPMPMRAITSVLGVDGPDEAAFCRLS

NQAVRITDVALSASGLISLVQGFAGFRRLRALFTHRRDNGLLRECTVLGK

LATHAEQGRLSDDELFFFAVLLLVAGYESTAHMISTLFLTLADYPDQLTL

LAQQPDLIPSAIEEHLRFISPIQNICRTTRVDYSVGQAVIPAGSLVLLAW

GAANRDPRQYEDPDVFRADRNPVGHLAFGSGIHLCPGTQLARMEGQAILR

EIVANIDRIEVVEPPTWTTNANLRGLTRLRVAVTPRVAP

>CYP128A1 (2673021302) Mycobacterium tuberculosis 301816

MTATQSPPEPAPDRVRLAGCPLAGTPDVGLTAQDATTALGVPTRRRASSG

GIPVATSMWRDAQTVRTYGPAVAKALALRVAGKARSRLTGRHCRKFMQLT

DFDPFDPAIAADPYPHYRELLAGERVQYNPKRDVYILSRYADVREAARNH

DTLSSARGVTFSRGWLPFLPTSDPPAHTRMRKQLAPGMARGALETWRPMV

DQLARELVGGLLTQTPADVVSTVAAPMPMRAITSVLGVDGPDEAAFCRLS

NQAVRITDVALSASGLISLVQGFAGFRRLRALFTHRRDNGLLRECTVLGK

LATHAEQGRLSDDELFFFAVLLLVAGYESTAHMISTLFLTLADYPDQLTL

LAQQPDLIPSAIEEHLRFISPIQNICRTTRVDYSVGQAVIPAGSLVLLAW

GAANRDPRQYEDPDVFRADRNPVGHLAFGSGIHLCPGTQLARMEGQAILR

EIVANIDRIEVVEPPTWTTNANLRGLTRLRVAVTPRVAP

>CYP128A1 (2683851312) Mycobacterium tuberculosis A00401371

MTATQSPPEPAPDRVRLAGCPLAGTPDVGLTAQDATTALGVPTRRRASSG

GIPVATSMWRDAQTVRTYGPAVAKALALRVAGKARSRLTGRHCRKFMQLT

DFDPFDPAIAADPYPHYRELLAGERVQYNPKRDVYILSRYADVREAARNH

DTLSSARGVTFSRGWLPFLPTSDPPAHTRMRKQLAPGMARGALETWRPMV

DQLARELVGGLLTQTPADVVSTVAAPMPMRAITSVLGVDGPDEAAFCRLS

NQAVRITDVALSASGLISLVQGFAGFRRLRALFTHRRDNGLLRECTVLGK

LATHAEQGRLSDDELFFFAVLLLVAGYESTAHMISTLFLTLADYPDQLTL

LAQQPDLIPSAIEEHLRFISPIQNICRTTRVDYSVGQAVIPAGSLVLLAW

GAANRDPRQYEDPDVFRADRNPVGHLAFGSGIHLCPGTQLARMEGQAILR

EIVANIDRIEVVEPPTWTTNANLRGLTRLRVAVTPRVAP

>CYP128A1 (2606428939) Mycobacterium tuberculosis XTB13-097

MTATQSPPEPAPDRVRLAGCPLAGTPDVGLTAQDATTALGVPTRRRAASG

GIPVATSMWRDAQTVRTYGPAVAKALALRVAGKARSRLTGRHCRKFMQLT

DFDPFDPAIAADPYPHYRELLAGERVQYNPKRDVYILSRYADVREAARNH

DTLSSARGVTFSRGWLPFLPTSDPPAHTRMRKQLAPGMARGALETWRPMV

DQLARELVGGLLTQTPADVVSTVAAPMPMRAITSVLGVDGPDEAAFCRLS

NQAVRITDVALSASGLISLVQGFAGFRRLRALFTHRRDNGLLRECTVLGK

LATHAEQGRLSDDELFFFAVLLLVAGYESTAHMISTLFLTLADYPDQLTL

LAQQPDLIPSAIEEHLRFISPIQNICRTTRVDYSVGQAVIPAGSLVLLAW

GAANRDPRQYEDPDVFRADRNPVGHLAFGSGIHLCPGTQLARMEGQAILR

EIVANIDRIEVVEPPTWTTNANLRGLTRLRVAVTPRVAP

>CYP128A1 (2589706733) Mycobacterium tuberculosis TKK-01-0082

MTATQSPPEPAPDRVRLAGCPLAGTPDVGLTAQDATTALGVPTRRRASSG

GIPVATSMWRDAQTVRTYGPAVAKALALRVAGKARSRLTGRHCRKFMQLT

DFDPFDPAIAADPYPHYRELLAGERVQYNPKRDVYILSRYADVREAARNH

DTLSSARGVTFSRGWLPFLPTSDPPAHTRMRKQLAPGMARGALETWRPMV

DQLARELVGGLLTQTPADVVSTVAAPMPMRAITSVLGVDGPDEAAFCRLS

NQAVRITDVALSASGLISLVQGFAGFRRLRALFTHRRDNGLLRECTVLGK

LATHAEQGRLSDDELFFFAVLLLVAGYESPAHMISTLFLTLADYPDQLTL

LAQQPDLIPSAIEEHLRFISPIQNICRTTRVDYSVGQAVIPAGSLVLLAW

GAANRDPRQYEDPDVFRADRNPVGHLAFGSGIHLCPGTQLARMEGQAILR

EIVANIDRIEVVEPPTWTTNANLRGLTRLRVAVTPRVAP

>CYP128A1 (2810621570) Mycobacterium colombiense 1211504.4

MTATLSGAEEAEGWVPKGGECPFGRDASYSGPAARDLSTPIGDVATRRVP

TLAATMKRDAQLARACAPMLTKVLAITAARKVRSGLAGRHTPEHVQITEF

DPMNPAIARDPYPHYRELLAGERVHYNPKRDVYILSRYSDVREAARNHEM

LSSAEGVTFSRGAPPFLPTSDPPAHTRMRKQLAPGMARGALESWRPMVDQ

LARELVCDLKTRTTADVVSLVAAPMPIRTITNVLGIAGPDEAEFIRLSNQ

AARITDVNLSVSGLGSLVHGFTGFRRLRALFTHMRDNGLLGECTVLGQLA

GHADRGRLSDDELFLFAVLLVVAGHETTANMISTLFLTLAEYPEQLRLLA

QQPELIPSAIEEQLRFLPPVQNMCRTTRVDYPVGHAVIPAGSRVLLLWGA

ANRDPRQYDDPDVFRAERNPAGHLAFGSGIHSCPGTHLARMEGQAVLREI

VANLDRIEVVEPPTWTTNANLRGLVRLRVAVTPRATA

>CYP128B4 (2802883506) Mycobacteroides abscessus abscessus 1149

MSRWRSDSAVRQIGAPVVAAVGMNIAAAVRVRRRGYAGWTGAVNTDYDPL

DPATAAQPFDAYRALHAGGRVHYNPKRATFILSRHEDIRAALRDTDAVTS

SQGVTRMKISAPILVLTDGDDHTRLRKQVQPGFTRGAMSDWQGMADQLAK

ELVADVVANPGCDVMERLAVPLPIRMIAHIIGIPPEDVQNFRSWSEDGVG

VINAGVSPAGLRQGLKGVRAIAALRRYFKDQLASGKLKGSDTVLGRLVDN

NEDGKLSDDELFFIAMLLLFAGNETTTNLIGGMFDTLAHAPDQFAMIRDD

PDLIPSAVEEQLRYSAPIQNLYRYTRTDYRVGEVTIPSGSRLLLAFGAAN

RDPEVFEDPDTYRADRNPRNHIAFGYGVHMCIGATLSRMEGQAVLRELTS

QASAIAAAGSATWSTNSSLRGTTYLPIRLTPAR

>CYP128B2 (2810622236) Mycobacterium colombiense 1211504.4

MAVKNSPRGMGTLLKPLGSAIEMNLAGAIRTRRRGYRGWTGAVNTDYDPL

DPATAAQPHEAYRALHRSGRVHYNPKRATWILSRLNDVRAALRDTDQVTS

TRGVTRVRMAADLLVLTDGDEHARLRKQVQPAFTRGALENWQEIADKLAA

ELVSEVLAESGCDVVQRLAIPMPMRMIAAILGVPDTDIADFRRWSENSVR

IINFSPTPKGVVDIANSMRAVIALRRYFLRHLATGDLKGSGTVLGRLLQH

SSEGNLSDDQLFYVALLLLIAGNETTTNLLGGMFDTLAHNPDQYDLIRAN

PDLIPIAVEEQLRFSSPIQNLYRYTRADYHVGEVTIPAGARVLLSFGAAN

RDPLVFDEPDEYRADRNPRMHVGFGYGAHMCVGAPLARMEAQAVLRELVD

RGVTRITAVGPTTWSTNSSLRGPTHLPVCCTAAESDLSTPAR

>CYP128B4 (2809724758) Mycobacterium sp. 1245111.1

VKISKPLTDRGALFTPLVSAVGLNLAANARVWRRGHRAWKGAVNTDYDPL

DRATAAQPFEAFRALHSAGRVHYNPKRATWILCRHEDVRAALRDTDAVTS

TQGVTRMRISAPVLVLSDGAEHTRLRKQVQPGFSKGAMASWQEMTDKLAA

ELVSDVLANPGCDVVERLTIPMPMRMIAHILGIPDADVGQFRAWSEAGMH

IVDFEATRHGVVRSAKAVASLAALRRYFLRQFASGGLKSSDTVLGRLLAH

TDDGSLTDDQLFYIAMLLLIAGNETTTNLLGGMFDTLAHNPVQFELIRAN

PQLIPMAVEEQLRISTPIQNLYRYTRADYKVGEVTIPVGSRIMLNFAAAN

RDPLVFDEPDEYRADRNPRNHIAFGYGAHMCVGAPLARMEAQAVLRELVS

QASAIAPAGDAIWSTNTSLRGPTRLPITLTRA

>CYP128B1 (2772232661) Mycobacterium shimoidei HMC_M2

MARSTPGVIGPFANAVRMNVVAAVKTRARGYRGWTGAVNTDFDPMDPVTA

SDPFEAYRKLHRSGRVHYNPRRSNFILSRLEDVRAALRDTEYVTSAEGVT

RLKFSAPLAVLTDGDEHARLRKQVQPGFSKGAMDSWKGMTKKLATELVTE

VIDNPGCDVVQRLAIPMPIRMIAQILGVPECDADDFRRWSEAGVGIMDFS

PTPRGVIDAAKSMAAMAALRRYFLDQFAHGGLKGSGTVLGRLLQHNSDGS

LTDEQLFLIAIHLLIAGNETTTNLLGGMFDTVARNPDQYDMIRADPGLIP

MAVEEQLRVTTPIQNLYRYTCADYEIAGVTIPRGSRVLLLFGAANRDPLA

FEDPDVYRADRNPRMHIAFGHGAHMCLGAPLARMEAQAVLCELVSRVSRI

DVLGDTTWSRHSSLRGPTRLAVRLTAA

>CYP128B2 (2809723781) Mycobacterium sp. 1245111.1

MTIAQTARGAVSLLTPLFAAGRLNISAAIQVRRRGYSAWTGAVNTDYDPL

DPATAAQPHAAYDALHRGGRVHYNPRRATWILHRLDDVRAALRDTDQVTS

SQGVTRIRMVADMVVFTDDDVHSRLRKQVQPAFTKGALDNWRDIIDRLAT

EVVDNMVNNPGDDAVKRLTVPMPVRVIAAILGIPDSDYHDFHQWSDQCTQ

FLNITPTVNGVVHGARAMRAAVTLRRYFMAHLAAGDIKGSNTVLGRLLEH

STDGELTDDQLFYIAITLLIAGNETTTNLLGGMLDTYAQFPDQYDMIRAN

PELIPHAIEEHLRYTSPVQHMYRYTRAPHQIGEVIIPTGSRLLVSVGAAN

RDPLAFDEPNTFRADRNPRTHVAFGYGPHLCLGAPLARMEAHAVLRELIN

RVSRISATGPATWSTNSALRGPTRLPLVLHKVSG

>CYP128A1 (2574844629) Mycobacterium tuberculosis TKK_04_0157

MTATQSPPEPAPDRVRLAGCPLAGTPDVGLTAQDATTALGVPTRRRASSG

GIPVATSMWRDAQTVRTYGPAVAKALALRVAGKARSRLTGRHCRKFMQLT

DFDPFDPAIAADPYPHYRELLAGERVQYNPKRDVYILSRYADVREAARNH

DTLSSARGVTFSRGWLPFLPTSDPPAHTRMRKQLAPGMARGALETWRPMV

DQLARELVGGLLTQTPADVVSTVAAPMPMRAITSVLGVDGPDEAAFCRLS

NQAVRITDVALSASGLISLVQGFAGFRRLRALFTHRRDNGLLRECTVLGK

LATHAEQGRLSDDELFFFAVLLLVAGYESTAHMISTLFLTLADYPDQLTL

LAQQPDLIPSAIEEHLRFISPIQNICRTTRVDYSVGQAVIPAGSLVLLAW

GAANRDPRQYEDPDVFRADRNPVGHLAFGSGIHLCPGTQLARMEGQAILR

EIVANIDRIEVVEPPTWTTNANLRGLTRLRVAVTPRVAP

>CYP128A1 (2584618346) Mycobacterium tuberculosis TRUG0116

MTATQSPPEPAPDRVRLAGCPLAGTPDVGLTAQDATTALGVPTRRRASSG

GIPVATSMWRDAQTVRTYGPAVAKALALRVAGKARSRLTGRHCRKFMQLT

DFDPFDPAIAADPYPHYRELLAGERVQYNPKRDVYILSRYADVREAARNH

DTLSSARGVTFSRGWLPFLPTSDPPAHTRMRKQLAPGMARGALETWRPMV

DQLARELVGGLLTQTPADVVSTVAAPMPMRAITSVLGVDGPDEAAFCRLS

NQAVRITDVALSASGLISLVQGFAGFRRLRALFTHRRDNGLLRECTVLGK

LATHAEQGRLSDDELFFFAVLLLVAGYESTAHMISTLFLTLADYPDQLTL

LAQQPDLIPSAIEEHLRFISPIQNICRTTRVDYSVGQAVIPAGSLVLLAW

GAANRDPRQYEDPDVFRADRNPVGHLAFGSGIHLCPGTQLARMEGQAILR

EIVANIDRIEVVEPPTWTTNANLRGLTRLRVAVTPRVAP

>CYP128A1 (2584637383) Mycobacterium tuberculosis 51628

MTATQSPPEPAPDRVRLAGCPLAGTPDVGLTAQDATTALGVPTRRRASSG

GIPVATSMWRDAQTVRTYGPAVAKALALRVAGKARSRLTGRHCRKFMQLT

DFDPFDPAIAADPYPHYRELLAGERVQYNPKRDVYILSRYADVREAARNH

DTLSSARGVTFSRGWLPFLPTSDPPAHTRMRKQLAPGMARGALETWRPMV

DQLARELVGGLLTQTPADVVSTVAAPMPMRAITSVLGVDGPDEAAFCRLS

NQAVRITDVALSASGLISLVQGFAGFRRLRALFTHRRDNGLLRECTVLGK

LATHAEQGRLSDDELFFFAVLLLVAGYESTAHMISTLFLTLADYPDQLTL

LAQQPDLIPSAIEEHLRFISPIQNICRTTRVDYSVGQAVIPAGSLVLLAW

GAANRDPRQYEDPDVFRADRNPVGHLAFGSGIHLCPGTQLARMEGQAILR

EIVANIDRIEVVEPPTWTTNANLRGLTRLRVAVTPRVAP

>CYP128A1 (2592328653) Mycobacterium tuberculosis TKK_03_0018

MTATQSPPEPAPDRVRLAGCPLAGTPDVGLTAQDATTALGVPTRRRASSG

GIPVATSMWRDAQTVRTYGPAVAKALALRVAGKARSRLTGRHCRKFMQLT

DFDPFDPAIAADPYPHYRELLAGERVQYNPKRDVYILSRYADVREAARNH

DTLSSARGVTFSRGWLPFLPTSDPPAHTRMRKQLAPGMARGALETWRPMV

DQLARELVGGLLTQTPADVVSTVAAPMPMRAITSVLGVDGPDEAAFCRLS

NQAVRITDVALSASGLISLVQGFAGFRRLRALFTHRRDNGLLRECTVLGK

LATHAEQGRLSDDELFFFAVLLLVAGYESTAHMISTLFLTLADYPDQLTL

LAQQPDLIPSAIEEHLRFISPIQNICRTTRVDYSVGQAVIPAGSLVLLAW

GAANRDPRQYEDPDVFRADRNPVGHLAFGSGIHLCPGTQLARMEGQAILR

EIVANIDRIEVVEPPTWTTNANLRGLTRLRVAVTPRVAP

>CYP128A1 (2603317136) Mycobacterium tuberculosis BTB08-014

MTATQSPPEPAPDRVRLAGCPLAGTPDVGLTAQDATTALGVPTRRRASSG

GIPVATSMWRDAQTVRTYGPAVAKALALRVAGKARSRLTGRHCRKFMQLT

DFDPFDPAIAADPYPHYRELLAGERVQYNPKRDVYILSRYADVREAARNH

DTLSSARGVTFSRGWLPFLPTSDPPAHTRMRKQLAPGMARGALETWRPMV

DQLARELVGGLLTQTPADVVSTVAAPMPMRAITSVLGVDGPDEAAFCRLS

NQAVRITDVALSASGLISLVQGFAGFRRLRALFTHRRDNGLLRECTVLGK

LATHAEQGRLSDDELFFFAVLLLVAGYESTAHMISTLFLTLADYPDQLTL

LAQQPDLIPSAIEEHLRFISPIQNICRTTRVDYSVGQAVIPAGSLVLLAW

GAANRDPRQYEDPDVFRADRNPVGHLAFGSGIHLCPGTQLARMEGQAILR

EIVANIDRIEVVEPPTWTTNANLRGLTRLRVAVTPRVAP

>CYP128A1 (2605343638) Mycobacterium tuberculosis TB_RSA101

MTATQSPPEPAPDRVRLAGCPLAGTPDVGLTAQDATTALGVPTRRRASSG

GIPVATSMWRDAQTVRTYGPAVAKALALRVAGKARSRLTGRHCRKFMQLT

DFDPFDPAIAADPYPHYRELLAGERVQYNPKRDVYILSRYADVREAARNH

DTLSSARGVTFSRGWLPFLPTSDPPAHTRMRKQLAPGMARGALETWRPMV

DQLARELVGGLLTQTPADVVSTVAAPMPMRAITSVLGVDGPDEAAFCRLS

NQAVRITDVALSASGLISLVQGFAGFRRLRALFTHRRDNGLLRECTVLGK

LATHAEQGRLSDDELFFFAVLLLVAGYESTAHMISTLFLTLADYPDQLTL

LAQQPDLIPSAIEEHLRFISPIQNICRTTRVDYSVGQAVIPAGSLVLLAW

GAANRDPRQYEDPDVFRADRNPVGHLAFGSGIHLCPGTQLARMEGQAILR

EIVANIDRIEVVEPPTWTTNANLRGLTRLRVAVTPRVAP

>CYP128A1 (2605419437) Mycobacterium tuberculosis 38765

MTATQSPPEPAPDRVRLAGCPLAGTPDVGLTAQDATTALGVPTRRRASSG

GIPVATSMWRDAQTVRTYGPAVAKALALRVAGKARSRLTGRHCRKFMQLT

DFDPFDPAIAADPYPHYRELLAGERVQYNPKRDVYILSRYADVREAARNH

DTLSSARGVTFSRGWLPFLPTSDPPAHTRMRKQLAPGMARGALETWRPMV

DQLARELVGGLLTQTPADVVSTVAAPMPMRAITSVLGVDGPDEAAFCRLS

NQAVRITDVALSASGLISLVQGFAGFRRLRALFTHRRDNGLLRECTVLGK

LATHAEQGRLSDDELFFFAVLLLVAGYESTAHMISTLFLTLADYPDQLTL

LAQQPDLIPSAIEEHLRFISPIQNICRTTRVDYSVGQAVIPAGSLVLLAW

GAANRDPRQYEDPDVFRADRNPVGHLAFGSGIHLCPGTQLARMEGQAILR

EIVANIDRIEVVEPPTWTTNANLRGLTRLRVAVTPRVAP

>CYP128A1 (2605761031) Mycobacterium tuberculosis TB_RSA61

MTATQSPPEPAPDRVRLAGCPLAGTPDVGLTAQDATTALGVPTRRRASSG

GIPVATSMWRDAQTVRTYGPAVAKALALRVAGKARSRLTGRHCRKFMQLT

DFDPFDPAIAADPYPHYRELLAGERVQYNPKRDVYILSRYADVREAARNH

DTLSSARGVTFSRGWLPFLPTSDPPAHTRMRKQLAPGMARGALETWRPMV

DQLARELVGGLLTQTPADVVSTVAAPMPMRAITSVLGVDGPDEAAFCRLS

NQAVRITDVALSASGLISLVQGFAGFRRLRALFTHRRDNGLLRECTVLGK

LATHAEQGRLSDDELFFFAVLLLVAGYESTAHMISTLFLTLADYPDQLTL

LAQQPDLIPSAIEEHLRFISPIQNICRTTRVDYSVGQAVIPAGSLVLLAW

GAANRDPRQYEDPDVFRADRNPVGHLAFGSGIHLCPGTQLARMEGQAILR

EIVANIDRIEVVEPPTWTTNANLRGLTRLRVAVTPRVAP

>CYP128A1 (2622524083) Mycobacterium tuberculosis M1410

MTATQSPPEPAPDRVRLAGCPLAGTPDVGLTAQDATTALGVPTRRRASSG

GIPVATSMWRDAQTVRTYGPAVAKALALRVAGKARSRLTGRHCRKFMQLT

DFDPFDPAIAADPYPHYRELLAGERVQYNPKRDVYILSRYADVREAARNH

DTLSSARGVTFSRGWLPFLPTSDPPAHTRMRKQLAPGMARGALETWRPMV

DQLARELVGGLLTQTPADVVSTVAAPMPMRAITSVLGVDGPDEAAFCRLS

NQAVRITDVALSASGLISLVQGFAGFRRLRALFTHRRDNGLLRECTVLGK

LATHAEQGRLSDDELFFFAVLLLVAGYESTAHMISTLFLTLADYPDQLTL

LAQQPDLIPSAIEEHLRFISPIQNICRTTRVDYSVGQAVIPAGSLVLLAW

GAANRDPRQYEDPDVFRADRNPVGHLAFGSGIHLCPGTQLARMEGQAILR

EIVANIDRIEVVEPPTWTTNANLRGLTRLRVAVTPRVAP

>CYP128A1 (2629964916) Mycobacterium tuberculosis NRITLD55

MTATQSPPEPAPDRVRLAGCPLAGTPDVGLTAQDATTALGVPTRRRASSG

GIPVATSMWRDAQTVRTYGPAVAKALALRVAGKARSRLTGRHCRKFMQLT

DFDPFDPAIAADPYPHYRELLAGERVQYNPKRDVYILSRYADVREAARNH

DTLSSARGVTFSRGWLPFLPTSDPPAHTRMRKQLAPGMARGALETWRPMV

DQLARELVGGLLTQTPADVVSTVAAPMPMRAITSVLGVDGPDEAAFCRLS

NQAVRITDVALSASGLISLVQGFAGFRRLRALFTHRRDNGLLRECTVLGK

LATHAEQGRLSDDELFFFAVLLLVAGYESTAHMISTLFLTLADYPDQLTL

LAQQPDLIPSAIEEHLRFISPIQNICRTTRVDYSVGQAVIPAGSLVLLAW

GAANRDPRQYEDPDVFRADRNPVGHLAFGSGIHLCPGTQLARMEGQAILR

EIVANIDRIEVVEPPTWTTNANLRGLTRLRVAVTPRVAP

>CYP128A1 (2630668017) Mycobacterium tuberculosis MD17614

MTATQSPPEPAPDRVRLAGCPLAGTPDVGLTAQDATTALGVPTRRRASSG

GIPVATSMWRDAQTVRTYGPAVAKALALRVAGKARSRLTGRHCRKFMQLT

DFDPFDPAIAADPYPHYRELLAGERVQYNPKRDVYILSRYADVREAARNH

DTLSSARGVTFSRGWLPFLPTSDPPAHTRMRKQLAPGMARGALETWRPMV

DQLARELVGGLLTQTPADVVSTVAAPMPMRAITSVLGVDGPDEAAFCRLS

NQAVRITDVALSASGLISLVQGFAGFRRLRALFTHRRDNGLLRECTVLGK

LATHAEQGRLSDDELFFFAVLLLVAGYESTAHMISTLFLTLADYPDQLTL

LAQQPDLIPSAIEEHLRFISPIQNICRTTRVDYSVGQAVIPAGSLVLLAW

GAANRDPRQYEDPDVFRADRNPVGHLAFGSGIHLCPGTQLARMEGQAILR

EIVANIDRIEVVEPPTWTTNANLRGLTRLRVAVTPRVAP

>CYP128A1 (2638123618) Mycobacterium bovis AF 2122/97

MTATQSPPEPAPDRVRLAGCPLAGTPDVGLTAQDATTALGVPTRRRASSG

GIPVATSMWRDAQTVRTYGPAVAKALALRVAGKARSRLTGRHCRKFMQLT

DFDPFDPAIAADPYPHYRELLAGERVQYNPKRDVYILSRYADVREAARNH

DTLSSARGVTFSRGWLPFLPTSDPPAHTRMRKQLAPGMARGALETWRPMV

DQLARELVGGLLTQTPADVVSTVAAPMPMRAITSVLGVDGPDEAAFCRLS

NQAVRITDVALSASGLISLVQGFAGFRRLRALFTHRRDNGLLRECTVLGK

LATHAEQGRLSDDELFFFAVLLLVAGYESTAHMISTLFLTLADYPDQLTL

LAQQPDLIPSAIEEHLRFISPIQNICRTTRVDYSVGQAVIPAGSLVLLAW

GAANRDPRQYEDPDVFRADRNPVGHLAFGSGIHLCPGTQLARMEGQAILR

EIVANIDRIEVVEPPTWTTNANLRGLTRLRVAVTPRVAP

>CYP128A1 (2706560847) Mycobacterium tuberculosis A09901284

MTATQSPPEPAPDRVRLAGCPLAGTPDVGLTAQDATTALGVPTRRRASSG

GIPVATSMWRDAQTVRTYGPAVAKALALRVAGKARSRLTGRHCRKFMQLT

DFDPFDPAIAADPYPHYRELLAGERVQYNPKRDVYILSRYADVREAARNH

DTLSSARGVTFSRGWLPFLPTSDPPAHTRMRKQLAPGMARGALETWRPMV

DQLARELVGGLLTQTPADVVSTVAAPMPMRAITSVLGVDGPDEAAFCRLS

NQAVRITDVALSASGLISLVQGFAGFRRLRALFTHRRDNGLLRECTVLGK

LATHAEQGRLSDDELFFFAVLLLVAGYESTAHMISTLFLTLADYPDQLTL

LAQQPDLIPSAIEEHLRFISPIQNICRTTRVDYSVGQAVIPAGSLVLLAW

GAANRDPRQYEDPDVFRADRNPVGHLAFGSGIHLCPGTQLARMEGQAILR

EIVANIDRIEVVEPPTWTTNANLRGLTRLRVAVTPRVAP

>CYP128A1 (2554285483) Mycobacterium tuberculosis SP21

MTATQSPPEPAPDRVRLAGCPLAGTPDVGLTAQDATTALGVPTRRRAASG

GIPVATSMWRDAQTVRTYGPAVAKALALRVAGKARSRLTGRHCRKFMQLT

DFDPFDPAIAADPYPHYRELLAGERVQYNPKRDVYILSRYADVREAARNH

DTLSSARGVTFSRGWLPFLPTSDPPAHTRMRKQLAPGMARGALETWRPMV

DQLARELVGGLLTQTPADVVSTVAAPMPMRAITSVLGVDGPDEAAFCRLS

NQAVRITDVALSASGLISLVQGFAGFRRLRALFTHRRDNGLLRECTVLGK

LATHAEQGRLSDDELFFFAVLLLVAGYESTAHMISTLFLTLADYPDQLTL

LAQQPDLIPSAIEEHLRFISPIQNICRTTRVDYSVGQAVIPAGSLVLLAW

GAANRDPRQYEDPDVFRADRNPVGHLAFGSGIHLCPGTQLARMEGQAILR

EIVANIDRIEVVEPPTWTTNANLRGLTRLRVAVTPRVAP

>CYP128A1 (2622368310) Mycobacterium tuberculosis M1030

MTATQSPPEPAPDRVRLAGCPLAGTPDVGLTAQDATTALGVPTRRRASSG

GIPVATSMWRDAQTVRTYGPAVAKALALRVAGKARSRLTGRHCRKFMQLT

DFDPFDPAIAADPYPHYRELLAGERVQYNPKRDVYILSRYADVREAARNH

DTLSSARGVTFSRGWLPFLPTSDPPAHTRMRKQLAPGMARGALETWRPMV

DQLARELVGGLLTQTPADVVSTVAAPMPMRAITSVLGVDGPDEAAFCRLS

NQAVRITDVALSASGLISLVQGFAGFRRLRALFTHRRDNGLLRECTVLGK

LATHAEQGRLSDDELFFFAVLLLVAGYESTAHMISTLFLTLADYPDQLTL

LAQQPDLIPSAIEEHLRFISPIQNICRTTRVDYSVGQAVIPAGSLVLLAW

GAANRDPRQYEDPDVFRADRNPVGHLAFGSGIHLCPGTQLARMEGQAILR

EIVANIDRIEVVEPPTWTTNANLHGLTRLRVAVTPRVAP

>CYP128A1 (2555380339) Mycobacterium tuberculosis PanR0801

MTATQSPPEPAPDRVRLAGCPLAGTPDVGLTAQDATTALGVPTRRRASSG

GIPVATSMWRDAQTVRTYGPAVAKALALRVAGKARSRLTGRHCRKFMQLT

DFDPFDPAIAADPYPHYRELLAGERVQYNPKRDVYILSRYADVREAARNH

DTLSSARGVTFSRGWLPFLPTSDPPAHTRMRKQLAPGMARGALETWRPMV

DQLARELVGGLLTQTPADVVSTVAAPMPMRAITSVLGVDGPDEAAFCRLS

NQAVRITDVALSASGLISLVQGFAGFRRLRALFTHRRDNGLLRECTVLGK

LATHAEQGRLSDDELFFFAVLLLVAGYESTAHMISTLFLTLADYPDQLTL

LAQQPDLIPSAIEEHLRFISPIQNICRTTRVDYSVGQAVIPAGSLVLLAW

GAANRDPRQYEDPDVFRADRNPVGHLAFGSGIHLCPGPSWRAWRVRRSCA

RSSPISTE

>CYP128B4 (2797926211) Mycobacteroides abscessus abscessus 480

MSRWRSDSAVRQIGAPVVAAVGMNIAAAVRVRRRGYAGWTGAVNTDYDPL

DPATAAQPFDAYRALHAGGRVHYNPKRATFILSRHEDIRAALRDTDAVTS

SQGVTRMKISAPILVLTDGDDHTRLRKQVQPGFTRGAMSDWQGMADQLAK

ELVADVVANPGCDVMERLAVPLPIRMIAHIIGIPPEDVQNFRSWSEDGVG

VINAGVSPAGLRQGLKGVRAIAALRRYFKDQLASGKLKGSDTVLGRLVDN

NEDGKLSDDELFFIAMLLLFAGNETTTNLIGGMFDTLAHAPDQFAMIRDD

PDLIPSAVEEQLRYSAPIQNLYRYTRTDYRVGEVTIPSGSRLLLAFGAAN

RDPEVFEDPDTYRADRNPRNHIAFGYGVHMCIGATLSRMEGQAVLRELTS

QASAIAAAGSATWSTNSSLRGTTYLPIRLTPAR

>CYP128B2 (2810495025) Mycobacterium asiaticum 1165133.8

MRASAALRRYFLKHLADGHLKGSDTVLGRLLAHSSDGALTDNQLFYIAIL

LLIAGNETTTNLLGGMLHTYAHCPDQYDMIRANPDLIPQAIEEHVRYTSP

IQNLYRYTRADYQVGDVTIPSGSRVLISFGAANRDPLAFDDPNTFRANRN

PRSHVGFGYGPHLCLGAPLARMEAQAVLRELVDRVSQISITGPTTWSTNS

SLRGPTHLPVVLHKRSTPI

>CYP128A1 (2555587853) Mycobacterium tuberculosis PanR0409

MTATQSPPEPAPDRVRLAGCPLAGTPDVGLTAQDATTALGVPTRRRASSG

GIPVATSMWRDAQTVRTYGPAVAKALALRVAGKARSRLTGRHCRKFMQLT

DFDPFDPAIAADPYPHYRELLAGERVQYNPKRDVYILSRYADVREAARNH

DTLSSARGVTFSRGWLPFLPTSDPPAHTRMRKQLAPGMARGALETWRPMV

DQLARELVGGLLTQTPADVVSTVAAPMPMRAITSVLGVDGPDEAAFCRLS

NQAVRITDVALSASGLISLVQGFAGFRRLRALFTHRRDNGLLRECTVLGK

LATHAEQGRLSDDELFFFAVLLLVAGYESTAHMISTLFLTLADYPDQLTL

LAQQPDLIPSAIEEHLRFISPIQNICRTTRVDYSVGQAVIPAGSLVLLAW

GAANRDPRQYEDPDVFRADRNPVGHLAFGSGIHLCPGTQLARMEGQAILR

EIVANIDRIEVVEPPTWTTNANLRGLTRLRVAVTPRVAP

>CYP128A1 (2589609011) Mycobacterium tuberculosis TKK-01-0049

MTATQSPPEPAPDRVRLAGCPLAGTPDVGLTAQDATTALGVPTRRRASSG

GIPVATSMWRDAQTVRTYGPAVAKALALRVAGKARSRLTGRHCRKFMQLT

DFDPFDPAIAADPYPHYRELLAGERVQYNPKRDVYILSRYADVREAARNH

DTLSSARGVTFSRGWLPFLPTSDPPAHTRMRKQLAPGMARGALETWRPMV

DQLARELVGGLLTQTPADVVSTVAAPMPMRAITSVLGVDGPDEAAFCRLS

NQAVRITDVALSASGLISLVQGFAGFRRLRALFTHRRDNGLLRECTVLGK

LATHAEQGRLSDDELFFFAVLLLVAGYESTAHMISTLFLTLADYPDQLTL

LAQQPDLIPSAIEEHLRFISPIQNICRTTRVDYSVGQAVIPAGSLVLLAW

GAANRDPRQYEDPDVFRADRNPVGHLAFGSGIHLCPGTQLARMEGQAILR

EIVANIDRIEVVEPPTWTTNANLRGLTRLRVAVTPRVAP

>CYP128A1 (2590155154) Mycobacterium tuberculosis MAL020187

MTATQSPPEPAPDRVRLAGCPLAGTPDVGLTAQDATTALGVPTRRRASSG

GIPVATSMWRDAQTVRTYGPAVAKALALRVAGKARSRLTGRHCRKFMQLT

DFDPFDPAIAADPYPHYRELLAGERVQYNPKRDVYILSRYADVREAARNH

DTLSSARGVTFSRGWLPFLPTSDPPAHTRMRKQLAPGMARGALETWRPMV

DQLARELVGGLLTQTPADVVSTVAAPMPMRAITSVLGVDGPDEAAFCRLS

NQAVRITDVALSASGLISLVQGFAGFRRLRALFTHRRDNGLLRECTVLGK

LATHAEQGRLSDDELFFFAVLLLVAGYESTAHMISTLFLTLADYPDQLTL

LAQQPDLIPSAIEEHLRFISPIQNICRTTRVDYSVGQAVIPAGSLVLLAW

GAANRDPRQYEDPDVFRADRNPVGHLAFGSGIHLCPGTQLARMEGQAILR

EIVANIDRIEVVEPPTWTTNANLRGLTRLRVAVTPRVAP

>CYP128A1 (2603278661) Mycobacterium tuberculosis TB_RSA24

MTATQSPPEPAPDRVRLAGCPLAGTPDVGLTAQDATTALGVPTRRRASSG

GIPVATSMWRDAQTVRTYGPAVAKALALRVAGKARSRLTGRHCRKFMQLT

DFDPFDPAIAADPYPHYRELLAGERVQYNPKRDVYILSRYADVREAARNH

DTLSSARGVTFSRGWLPFLPTSDPPAHTRMRKQLAPGMARGALETWRPMV

DQLARELVGGLLTQTPADVVSTVAAPMPMRAITSVLGVDGPDEAAFCRLS

NQAVRITDVALSASGLISLVQGFAGFRRLRALFTHRRDNGLLRECTVLGK

LATHAEQGRLSDDELFFFAVLLLVAGYESTAHMISTLFLTLADYPDQLTL

LAQQPDLIPSAIEEHLRFISPIQNICRTTRVDYSVGQAVIPAGSLVLLAW

GAANRDPRQYEDPDVFRADRNPVGHLAFGSGIHLCPGTQLARMEGQAILR

EIVANIDRIEVVEPPTWTTNANLRGLTRLRVAVTPRVAP

>CYP128A1 (2604689455) Mycobacterium tuberculosis TB_RSA38

MTATQSPPEPAPDRVRLAGCPLAGTPDVGLTAQDATTALGVPTRRRASSG

GIPVATSMWRDAQTVRTYGPAVAKALALRVAGKARSRLTGRHCRKFMQLT

DFDPFDPAIAADPYPHYRELLAGERVQYNPKRDVYILSRYADVREAARNH

DTLSSARGVTFSRGWLPFLPTSDPPAHTRMRKQLAPGMARGALETWRPMV

DQLARELVGGLLTQTPADVVSTVAAPMPMRAITSVLGVDGPDEAAFCRLS

NQAVRITDVALSASGLISLVQGFAGFRRLRALFTHRRDNGLLRECTVLGK

LATHAEQGRLSDDELFFFAVLLLVAGYESTAHMISTLFLTLADYPDQLTL

LAQQPDLIPSAIEEHLRFISPIQNICRTTRVDYSVGQAVIPAGSLVLLAW

GAANRDPRQYEDPDVFRADRNPVGHLAFGSGIHLCPGTQLARMEGQAILR

EIVANIDRIEVVEPPTWTTNANLRGLTRLRVAVTPRVAP

>CYP128A1 (2605729358) Mycobacterium tuberculosis TB_RSA170

MTATQSPPEPAPDRVRLAGCPLAGTPDVGLTAQDATTALGVPTRRRASSG

GIPVATSMWRDAQTVRTYGPAVAKALALRVAGKARSRLTGRHCRKFMQLT

DFDPFDPAIAADPYPHYRELLAGERVQYNPKRDVYILSRYADVREAARNH

DTLSSARGVTFSRGWLPFLPTSDPPAHTRMRKQLAPGMARGALETWRPMV

DQLARELVGGLLTQTPADVVSTVAAPMPMRAITSVLGVDGPDEAAFCRLS

NQAVRITDVALSASGLISLVQGFAGFRRLRALFTHRRDNGLLRECTVLGK

LATHAEQGRLSDDELFFFAVLLLVAGYESTAHMISTLFLTLADYPDQLTL

LAQQPDLIPSAIEEHLRFISPIQNICRTTRVDYSVGQAVIPAGSLVLLAW

GAANRDPRQYEDPDVFRADRNPVGHLAFGSGIHLCPGTQLARMEGQAILR

EIVANIDRIEVVEPPTWTTNANLRGLTRLRVAVTPRVAP

>CYP128A1 (2622326771) Mycobacterium tuberculosis H3367

MTATQSPPEPAPDRVRLAGCPLAGTPDVGLTAQDATTALGVPTRRRASSG

GIPVATSMWRDAQTVRTYGPAVAKALALRVAGKARSRLTGRHCRKFMQLT

DFDPFDPAIAADPYPHYRELLAGERVQYNPKRDVYILSRYADVREAARNH

DTLSSARGVTFSRGWLPFLPTSDPPAHTRMRKQLAPGMARGALETWRPMV

DQLARELVGGLLTQTPADVVSTVAAPMPMRAITSVLGVDGPDEAAFCRLS

NQAVRITDVALSASGLISLVQGFAGFRRLRALFTHRRDNGLLRECTVLGK

LATHAEQGRLSDDELFFFAVLLLVAGYESTAHMISTLFLTLADYPDQLTL

LAQQPDLIPSAIEEHLRFISPIQNICRTTRVDYSVGQAVIPAGSLVLLAW

GAANRDPRQYEDPDVFRADRNPVGHLAFGSGIHLCPGTQLARMEGQAILR

EIVANIDRIEVVEPPTWTTNANLRGLTRLRVAVTPRVAP

>CYP128A1 (2632967904) Mycobacterium tuberculosis TKK_03_0072

MTATQSPPEPAPDRVRLAGCPLAGTPDVGLTAQDATTALGVPTRRRASSG

GIPVATSMWRDAQTVRTYGPAVAKALALRVAGKARSRLTGRHCRKFMQLT

DFDPFDPAIAADPYPHYRELLAGERVQYNPKRDVYILSRYADVREAARNH

DTLSSARGVTFSRGWLPFLPTSDPPAHTRMRKQLAPGMARGALETWRPMV

DQLARELVGGLLTQTPADVVSTVAAPMPMRAITSVLGVDGPDEAAFCRLS

NQAVRITDVALSASGLISLVQGFAGFRRLRALFTHRRDNGLLRECTVLGK

LATHAEQGRLSDDELFFFAVLLLVAGYESTAHMISTLFLTLADYPDQLTL

LAQQPDLIPSAIEEHLRFISPIQNICRTTRVDYSVGQAVIPAGSLVLLAW

GAANRDPRQYEDPDVFRADRNPVGHLAFGSGIHLCPGTQLARMEGQAILR

EIVANIDRIEVVEPPTWTTNANLRGLTRLRVAVTPRVAP

>CYP128A1 (2665026037) Mycobacterium tuberculosis 402568

MTATQSPPEPAPDRVRLAGCPLAGTPDVGLTAQDATTALGVPTRRRASSG

GIPVATSMWRDAQTVRTYGPAVAKALALRVAGKARSRLTGRHCRKFMQLT

DFDPFDPAIAADPYPHYRELLAGERVQYNPKRDVYILSRYADVREAARNH

DTLSSARGVTFSRGWLPFLPTSDPPAHTRMRKQLAPGMARGALETWRPMV

DQLARELVGGLLTQTPADVVSTVAAPMPMRAITSVLGVDGPDEAAFCRLS

NQAVRITDVALSASGLISLVQGFAGFRRLRALFTHRRDNGLLRECTVLGK

LATHAEQGRLSDDELFFFAVLLLVAGYESTAHMISTLFLTLADYPDQLTL

LAQQPDLIPSAIEEHLRFISPIQNICRTTRVDYSVGQAVIPAGSLVLLAW

GAANRDPRQYEDPDVFRADRNPVGHLAFGSGIHLCPGTQLARMEGQAILR

EIVANIDRIEVVEPPTWTTNANLRGLTRLRVAVTPRVAP

>CYP128A1 (2693750156) Mycobacterium tuberculosis A70067_1

MTATQSPPEPAPDRVRLAGCPLAGTPDVGLTAQDATTALGVPTRRRASSG

GIPVATSMWRDAQTVRTYGPAVAKALALRVAGKARSRLTGRHCRKFMQLT

DFDPFDPAIAADPYPHYRELLAGERVQYNPKRDVYILSRYADVREAARNH

DTLSSARGVTFSRGWLPFLPTSDPPAHTRMRKQLAPGMARGALETWRPMV

DQLARELVGGLLTQTPADVVSTVAAPMPMRAITSVLGVDGPDEAAFCRLS

NQAVRITDVALSASGLISLVQGFAGFRRLRALFTHRRDNGLLRECTVLGK

LATHAEQGRLSDDELFFFAVLLLVAGYESTAHMISTLFLTLADYPDQLTL

LAQQPDLIPSAIEEHLRFISPIQNICRTTRVDYSVGQAVIPAGSLVLLAW

GAANRDPRQYEDPDVFRADRNPVGHLAFGSGIHLCPGTQLARMEGQAILR

EIVANIDRIEVVEPPTWTTNANLRGLTRLRVAVTPRVAP

>CYP128A1 (2715370170) Mycobacterium tuberculosis A4

MTATQSPPEPAPDRVRLAGCPLAGTPDVGLTAQDATTALGVPTRRRASSG

GIPVATSMWRDAQTVRTYGPAVAKALALRVAGKARSRLTGRHCRKFMQLT

DFDPFDPAIAADPYPHYRELLAGERVQYNPKRDVYILSRYADVREAARNH

DTLSSARGVTFSRGWLPFLPTSDPPAHTRMRKQLAPGMARGALETWRPMV

DQLARELVGGLLTQTPADVVSTVAAPMPMRAITSVLGVDGPDEAAFCRLS

NQAVRITDVALSASGLISLVQGFAGFRRLRALFTHRRDNGLLRECTVLGK

LATHAEQGRLSDDELFFFAVLLLVAGYESTAHMISTLFLTLADYPDQLTL

LAQQPDLIPSAIEEHLRFISPIQNICRTTRVDYSVGQAVIPAGSLVLLAW

GAANRDPRQYEDPDVFRADRNPVGHLAFGSGIHLCPGTQLARMEGQAILR

EIVANIDRIEVVEPPTWTTNANLRGLTRLRVAVTPRVAP

>CYP128A1 (2541570444) Mycobacterium canettii CIPT 140070008

MTATQSPPEPAPDRVQLAGCPLAGTPDVGLTAQDATTALGVPTRRRASSG

GIPVATSMWRDAQTVRTYGPAVAKALALRVAGKARSRLAGRHCRKFMQLT

DFDPFDPAIAADPYPHYRELLAGERVQYNPKRDVYILSRYADVREAARNH

DTLSSARGVTFSRGWLPFLPTSDPPAHTRMRKQLAPGMARGALETWRPMV

DQLARELVGGLLTQTPADVVSTVAAPMPMRAITSVLGVDGPDEAAFCRLS

NQAVRITDVALSASGLISLVQGFAGFRRLRALFTHRRDNGLLRECTVLGK

LATHAEQGRLSDDELFFFAVLLLVAGYESTAHMISTLFLTLADYPDQLTL

LAQQPDLIPSAIEEHLRFISPIQNICRTTRVDYSVGQAVIPAGSLVLLAW

GAANRDPRQYEDPDVFRADRNPVGHLAFGSGIHLCPGTQLARMEGQAILR

EIVANIDRIEVVEPPTWTTNANLRGLTRLRVAVTPRVAP

>CYP128A1 (2603973530) Mycobacterium tuberculosis TB_RSA176

MTATQSPPEPAPDRVRLAGCPLAGTPDVGLTAQDATTALGVPTRRRASSG

GIPVATSMWRDAQTVRTYGPAVAKALALRVAGKARSRLTGRHCRKFMQLT

DFDPFDPAIAADPYPHYRELLAGERVQYNPKRDVYILSRYADVREAARNH

DTLSSARGVTFSRGWLPFLPTSDPPAHTRMRKQLAPGMARGALETWRPMV

DQLARELVGGLLTQTPADVVSTVAAPMPMRAITSVLGVDGPDEAAFCRLS

NQAVRITDVALSASGLISLVQGFAGFRRLRALFTHRRDNGLLRECTVLGK

LATHAEQGRLSDDELFFFAVLLLVAGYESPAHMISTLFLTLADYPDQLTL

LAQQPDLIPSAIEEHLRFISPIQNICRTTRVDYSVGQAVIPAGSLVLLAW

GAANRDPRQYEDPDVFRADRNPVGHLAFGSGIHLCPGTQLARMEGQAILR

EIVANIDRIEVVEPPTWTTNANLRGLTRLRVAVTPRVAP

>CYP128A1 (2683873926) Mycobacterium tuberculosis Q09901557

MTATQSPPEPAPDRVRLAGCPLAGTPDVGLTAQDATTALGVPTRRRASSG

GIPVATSMWRDAQTVRTYGPAVAKALALRVAGKARSRLTGRHCRKFMQLT

DFDPFDPAIAADPYPHYRELLAGERVQYNPKRDVYILSRYADVREAARNH

DTLSSARGVTFSRGWLPFLPTSDPPAHTRMRKQLAPGMARGALETWRPMV

DQLARELVGGLLTQTPADVVSTVAAPMPMRAITSVLGVDGPDEAAFCRLS

NQAVRITDVALSASGLISLVQGFAGFRRLRALFTHRRDNGLLRECTVLGK

LATHAEQGRLSDDELFFFAVLLLVAGYESTAHMISTLFLTLADYPDQLTL

LAQQPDLIPSAIEEHLRFISPIQNICRTTRVDYSVGQAVIPAGSLVLMAW

GAANRDPRQYEDPDVFRADRNPVGHLAFGSGIHLCPGPSWRAWRVRRSCA

RSSPISTE

>CYP128B4 (2803113232) Mycobacteroides abscessus abscessus 1154

MSRWRSDSAVRQIGAPVVAAVGMNIAAAVRVRRRGYAGWTGAVNTDYDPL

DPATAAQPFDAYRALHAGGRVHYNPKRATFILSRHEDIRAALRDTDAVTS

SQGVTRMKISAPILVLTDGDDHTRLRKQVQPGFTRGAMSDWQGMADQLAK

ELVADVVANPGCDVMERLAVPLPIRMIAHIIGIPPEDVQNFRSWSEDGVG

VINAGVSPAGLRQGLKGVRAIAALRRYFKDQLASGKLKGSDTVLGRLVDN

NEDGKLSDDELFFIAMLLLFAGNETTTNLIGGMFDTLAHAPDQFAMIRDD

PDLIPSAVEEQLRYSAPIQNLYRYTRTDYRVGEVTIPSGSRLLLAFGAAN

RDPEVFEDPDTYRADRNPRNHIAFGYGVHMCIGATLSRMEGQAVLRELTS

QASAIAAAGSATWSTNSSLRGTTYLPIRLTPAR

>CYP128B4 (2809942142) Mycobacterium sp. E2327

MALTDGVRFTGSAARTLAGAALANAVGARRRLPLDADITAYDPLDAATAA

QPHEAYRRLHAGARVQYNPKRKVFVLSRLDDVRAAARDDAKLSSADGPTL

TRIRTPILVSLDGDAHARQRRQVLPAFTKAALDSWRPIIDRLAAETVHDV

LADPGCDVVQRLAIPMPVRLIARLLGVPDTDVDDFRRWSEASVQITDIDL

TARGARKLAGSLRGSWAMHRYFTRQFAVGGLKGSDTILGRLLAENESGSL

PDRELFYFAMLLLLAGNETTTNLLGGMFDTLAAHPEQYDLLRADPDAVPM

AVEELLRYLSPAQNVYRTALSDYRVGDTTIPAGARIMLSIGAANRDPRAF

DEPDAFRVDRNPTQHITFGFGAHLCIGAQLTRMEAQAVLRELTAHVSRIS

VVGAPQWSTNSLLRGPTRLAVHLTPA

>CYP128B2 (2724977653) Mycobacterium avium hominissuis MAH-E-96-2

LTLSQDVRFLGTMAQPLSHAARVNTARAFRSRWRQYRFWTGAQITDYDPI

DPANIAQPDAAYRALHAGGRVHYNPKLGLWILTRLPDVRAGARAAETLSS

ADGVTRLRMAGPLLVTMDGKPHNEMRRHVLPAFTKAALESWQAMIDDLAA

KLVGEVLDNPGCDVVQRLAIPMPMLLIAHMLGIPNGDIDDFRRWSTAAVK

MADVDISRRGLTRLKSSIGGIRDIYRYFRQQFAVGGLKGSDTLLGKLLSV

NASGSIDDNELFFFAMLLLIAGNETTTNLLGGMFDIFARHPEKFDMVRAD

HSLIPKVVEEQLRFSSPVQMLYRTARTNYEVGPVTIPAGARVLLSLGAAN

RDPQVFDEPDEFRVDRNPTEHLAFGFGAHLCLGAQLTRMEAQAVLRELVT

RARRIEAIGETQWSTGYLLRGPERMNVRLTPSVVAG

>CYP128B1 (2809679049) Mycobacterium alsense E2978

MSISQTAQGAAEAIRPFSNAVRMNLTAAVRTRRRGYGAWTGAVNTDYDPQ

NPTTAAQPFDAYRALHRTGRVHYNPRRATWIISRLDDVRAALRDTDQVTS

TQGVTRLRMSAPLAVLTDGEEHTRLRKQVQPGFSKGAMSAWQEIVEKLAV

ELVADLLNNPGCDVVQHLAIPMPIRLIAQILGVPEGDVNDFRRWSENAVK

VMELTPTRAGFVGAVRSISAMVALQRYFVKQFAVGGLKGSGTVLGRLLDQ

NTDGSLTDRQLWLIAIHLLIAGNETTTNLLGGMFDTLANHPDQYDLICAN

PDIIPMAVEEQLRITTPIQNLYRYTRADYQIGHVTIPAGSRVLLSFGAAN

RDPAVFDQPDEYRADRNPRTHVAFGYGAHMCLGAPLARMEAQAVLRELVT

RVSRLTPKGSTTWSTHSSLRGPTHLPIHLTPA

>CYP128A1 (2576052442) Mycobacterium tuberculosis TB_RSA67

MTATQSPPEPAPDRVRLAGCPLAGTPDVGLTAQDATTALGVPTRRRASSG

GIPVATSMWRDAQTVRTYGPAVAKALALRVAGKARSRLTGRHCRKFMQLT

DFDPFDPAIAADPYPHYRELLAGERVQYNPKRDVYILSRYADVREAARNH

DTLSSARGVTFSRGWLPFLPTSDPPAHTRMRKQLAPGMARGALETWRPMV

DQLARELVGGLLTQTPADVVSTVAAPMPMRAITSVLGVDGPDEAAFCRLS

NQAVRITDVALSASGLISLVQGFAGFRRLRALFTHRRDNGLLRECTVLGK

LATHAEQGRLSDDELFFFAVLLLVAGYESTAHMISTLFLTLADYPDQLTL

LAQQPDLIPSAIEEHLRFISPIQNICRTTRVDYSVGQAVIPAGSLVLLAW

GAANRDPRQYEDPDVFRADRNPVGHLAFGSGIHLCPGTQLARMEGQAILR

EIVANIDRIEVVEPPTWTTNANLRGLTRLRVAVTPRVAP

>CYP128A1 (2589165931) Mycobacterium tuberculosis OFXR-16

MTATQSPPEPAPDRVRLAGCPLAGTPDVGLTAQDATTALGVPTRRRASSG

GIPVATSMWRDAQTVRTYGPAVAKALALRVAGKARSRLTGRHCRKFMQLT

DFDPFDPAIAADPYPHYRELLAGERVQYNPKRDVYILSRYADVREAARNH

DTLSSARGVTFSRGWLPFLPTSDPPAHTRMRKQLAPGMARGALETWRPMV

DQLARELVGGLLTQTPADVVSTVAAPMPMRAITSVLGVDGPDEAAFCRLS

NQAVRITDVALSASGLISLVQGFAGFRRLRALFTHRRDNGLLRECTVLGK

LATHAEQGRLSDDELFFFAVLLLVAGYESTAHMISTLFLTLADYPDQLTL

LAQQPDLIPSAIEEHLRFISPIQNICRTTRVDYSVGQAVIPAGSLVLLAW

GAANRDPRQYEDPDVFRADRNPVGHLAFGSGIHLCPGTQLARMEGQAILR

EIVANIDRIEVVEPPTWTTNANLRGLTRLRVAVTPRVAP

>CYP128A1 (2590260791) Mycobacterium tuberculosis KT-0022

MTATQSPPEPAPDRVRLAGCPLAGTPDVGLTAQDATTALGVPTRRRASSG

GIPVATSMWRDAQTVRTYGPAVAKALALRVAGKARSRLTGRHCRKFMQLT

DFDPFDPAIAADPYPHYRELLAGERVQYNPKRDVYILSRYADVREAARNH

DTLSSARGVTFSRGWLPFLPTSDPPAHTRMRKQLAPGMARGALETWRPMV

DQLARELVGGLLTQTPADVVSTVAAPMPMRAITSVLGVDGPDEAAFCRLS

NQAVRITDVALSASGLISLVQGFAGFRRLRALFTHRRDNGLLRECTVLGK

LATHAEQGRLSDDELFFFAVLLLVAGYESTAHMISTLFLTLADYPDQLTL

LAQQPDLIPSAIEEHLRFISPIQNICRTTRVDYSVGQAVIPAGSLVLLAW

GAANRDPRQYEDPDVFRADRNPVGHLAFGSGIHLCPGTQLARMEGQAILR

EIVANIDRIEVVEPPTWTTNANLRGLTRLRVAVTPRVAP

>CYP128A1 (2592426951) Mycobacterium tuberculosis TKK_02_0013

MTATQSPPEPAPDRVRLAGCPLAGTPDVGLTAQDATTALGVPTRRRASSG

GIPVATSMWRDAQTVRTYGPAVAKALALRVAGKARSRLTGRHCRKFMQLT

DFDPFDPAIAADPYPHYRELLAGERVQYNPKRDVYILSRYADVREAARNH

DTLSSARGVTFSRGWLPFLPTSDPPAHTRMRKQLAPGMARGALETWRPMV

DQLARELVGGLLTQTPADVVSTVAAPMPMRAITSVLGVDGPDEAAFCRLS

NQAVRITDVALSASGLISLVQGFAGFRRLRALFTHRRDNGLLRECTVLGK

LATHAEQGRLSDDELFFFAVLLLVAGYESTAHMISTLFLTLADYPDQLTL

LAQQPDLIPSAIEEHLRFISPIQNICRTTRVDYSVGQAVIPAGSLVLLAW

GAANRDPRQYEDPDVFRADRNPVGHLAFGSGIHLCPGTQLARMEGQAILR

EIVANIDRIEVVEPPTWTTNANLRGLTRLRVAVTPRVAP

>CYP128A1 (2604997371) Mycobacterium tuberculosis XDR KZN 4207

MTATQSPPEPAPDRVRLAGCPLAGTPDVGLTAQDATTALGVPTRRRASSG

GIPVATSMWRDAQTVRTYGPAVAKALALRVAGKARSRLTGRHCRKFMQLT

DFDPFDPAIAADPYPHYRELLAGERVQYNPKRDVYILSRYADVREAARNH

DTLSSARGVTFSRGWLPFLPTSDPPAHTRMRKQLAPGMARGALETWRPMV

DQLARELVGGLLTQTPADVVSTVAAPMPMRAITSVLGVDGPDEAAFCRLS

NQAVRITDVALSASGLISLVQGFAGFRRLRALFTHRRDNGLLRECTVLGK

LATHAEQGRLSDDELFFFAVLLLVAGYESTAHMISTLFLTLADYPDQLTL

LAQQPDLIPSAIEEHLRFISPIQNICRTTRVDYSVGQAVIPAGSLVLLAW

GAANRDPRQYEDPDVFRADRNPVGHLAFGSGIHLCPGTQLARMEGQAILR

EIVANIDRIEVVEPPTWTTNANLRGLTRLRVAVTPRVAP

>CYP128A1 (2622112027) Mycobacterium tuberculosis MD17503

MTATQSPPEPAPDRVRLAGCPLAGTPDVGLTAQDATTALGVPTRRRASSG

GIPVATSMWRDAQTVRTYGPAVAKALALRVAGKARSRLTGRHCRKFMQLT

DFDPFDPAIAADPYPHYRELLAGERVQYNPKRDVYILSRYADVREAARNH

DTLSSARGVTFSRGWLPFLPTSDPPAHTRMRKQLAPGMARGALETWRPMV

DQLARELVGGLLTQTPADVVSTVAAPMPMRAITSVLGVDGPDEAAFCRLS

NQAVRITDVALSASGLISLVQGFAGFRRLRALFTHRRDNGLLRECTVLGK

LATHAEQGRLSDDELFFFAVLLLVAGYESTAHMISTLFLTLADYPDQLTL

LAQQPDLIPSAIEEHLRFISPIQNICRTTRVDYSVGQAVIPAGSLVLLAW

GAANRDPRQYEDPDVFRADRNPVGHLAFGSGIHLCPGTQLARMEGQAILR

EIVANIDRIEVVEPPTWTTNANLRGLTRLRVAVTPRVAP

>CYP128A1 (2622242361) Mycobacterium tuberculosis MD15762

MTATQSPPEPAPDRVRLAGCPLAGTPDVGLTAQDATTALGVPTRRRASSG

GIPVATSMWRDAQTVRTYGPAVAKALALRVAGKARSRLTGRHCRKFMQLT

DFDPFDPAIAADPYPHYRELLAGERVQYNPKRDVYILSRYADVREAARNH

DTLSSARGVTFSRGWLPFLPTSDPPAHTRMRKQLAPGMARGALETWRPMV

DQLARELVGGLLTQTPADVVSTVAAPMPMRAITSVLGVDGPDEAAFCRLS

NQAVRITDVALSASGLISLVQGFAGFRRLRALFTHRRDNGLLRECTVLGK

LATHAEQGRLSDDELFFFAVLLLVAGYESTAHMISTLFLTLADYPDQLTL

LAQQPDLIPSAIEEHLRFISPIQNICRTTRVDYSVGQAVIPAGSLVLLAW

GAANRDPRQYEDPDVFRADRNPVGHLAFGSGIHLCPGTQLARMEGQAILR

EIVANIDRIEVVEPPTWTTNANLRGLTRLRVAVTPRVAP

>CYP128A1 (2629710364) Mycobacterium tuberculosis TKK_04_0070

MTATQSPPEPAPDRVRLAGCPLAGTPDVGLTAQDATTALGVPTRRRASSG

GIPVATSMWRDAQTVRTYGPAVAKALALRVAGKARSRLTGRHCRKFMQLT

DFDPFDPAIAADPYPHYRELLAGERVQYNPKRDVYILSRYADVREAARNH

DTLSSARGVTFSRGWLPFLPTSDPPAHTRMRKQLAPGMARGALETWRPMV

DQLARELVGGLLTQTPADVVSTVAAPMPMRAITSVLGVDGPDEAAFCRLS

NQAVRITDVALSASGLISLVQGFAGFRRLRALFTHRRDNGLLRECTVLGK

LATHAEQGRLSDDELFFFAVLLLVAGYESTAHMISTLFLTLADYPDQLTL

LAQQPDLIPSAIEEHLRFISPIQNICRTTRVDYSVGQAVIPAGSLVLLAW

GAANRDPRQYEDPDVFRADRNPVGHLAFGSGIHLCPGTQLARMEGQAILR

EIVANIDRIEVVEPPTWTTNANLRGLTRLRVAVTPRVAP

>CYP128A1 (2633034961) Mycobacterium tuberculosis KT-0193

MTATQSPPEPAPDRVRLAGCPLAGTPDVGLTAQDATTALGVPTRRRASSG

GIPVATSMWRDAQTVRTYGPAVAKALALRVAGKARSRLTGRHCRKFMQLT

DFDPFDPAIAADPYPHYRELLAGERVQYNPKRDVYILSRYADVREAARNH

DTLSSARGVTFSRGWLPFLPTSDPPAHTRMRKQLAPGMARGALETWRPMV

DQLARELVGGLLTQTPADVVSTVAAPMPMRAITSVLGVDGPDEAAFCRLS

NQAVRITDVALSASGLISLVQGFAGFRRLRALFTHRRDNGLLRECTVLGK

LATHAEQGRLSDDELFFFAVLLLVAGYESTAHMISTLFLTLADYPDQLTL

LAQQPDLIPSAIEEHLRFISPIQNICRTTRVDYSVGQAVIPAGSLVLLAW

GAANRDPRQYEDPDVFRADRNPVGHLAFGSGIHLCPGTQLARMEGQAILR

EIVANIDRIEVVEPPTWTTNANLRGLTRLRVAVTPRVAP

>CYP128A1 (2678507765) Mycobacterium tuberculosis C53_20899

MTATQSPPEPAPDRVRLAGCPLAGTPDVGLTAQDATTALGVPTRRRASSG

GIPVATSMWRDAQTVRTYGPAVAKALALRVAGKARSRLTGRHCRKFMQLT

DFDPFDPAIAADPYPHYRELLAGERVQYNPKRDVYILSRYADVREAARNH

DTLSSARGVTFSRGWLPFLPTSDPPAHTRMRKQLAPGMARGALETWRPMV

DQLARELVGGLLTQTPADVVSTVAAPMPMRAITSVLGVDGPDEAAFCRLS

NQAVRITDVALSASGLISLVQGFAGFRRLRALFTHRRDNGLLRECTVLGK

LATHAEQGRLSDDELFFFAVLLLVAGYESTAHMISTLFLTLADYPDQLTL

LAQQPDLIPSAIEEHLRFISPIQNICRTTRVDYSVGQAVIPAGSLVLLAW

GAANRDPRQYEDPDVFRADRNPVGHLAFGSGIHLCPGTQLARMEGQAILR

EIVANIDRIEVVEPPTWTTNANLRGLTRLRVAVTPRVAP

>CYP128A1 (651039611) Mycobacterium canettii CIPT 140010059

MTATQSPPEPAPDRVQLAGCPLAGTPDVGLTAQDATTALGVPTRRRASSG

GIPVATSMWRDAQTVRTYGPAVAKALALRVAGKARSRLAGRHCRKFMQLT

DFDPFDPAIAADPYPHYRELLAGERVQYNPKRDVYILSRYADVREAARNH

DTLSSARGVTFSRGWLPFLPTSDPPAHTRMRKQLAPGMARGALETWRPMV

DQLARELVGGLLTQTPADVVSTVAAPMPMRAITSVLGVDGPDEAAFCRLS

NQAVRITDVALSASGLISLVQGFAGFRRLRALFTHRRDNGLLRECTVLGK

LATHAEQGRLSDDELFFFAVLLLVAGYESTAHMISTLFLTLADYPDQLTL

LAQQPDLIPSAIEEHLRFISPIQNICRTTRVDYSVGQAVIPAGSLVLLAW

GAANRDPRQYEDPDVFRADRNPVGHLAFGSGIHLCPGTQLARMEGQAILR

EIVANIDRIEVVEPPTWTTNANLRGLTRLRVAVTPRVAP

>CYP128A1 (2672114961) Mycobacterium tuberculosis A70762

MTATQSPPEPAPDRVRLAGCPLAGTPDVGLTAQDATTALGVPTRRRASSG

GIPVATSMWRDAQTVRTYGPAVAKALALRVAGKARSRLTGRHCRKFMQLT

DFDPFDPAIAADPYPHYRELLAGERVQDNPKRDVYILSRYADVREAARNH

DTLSSARGVTFSRGWLPFLPTSDPPAHTRMRKQLAPGMARGALETWRPMV

DQLARELVGGLLTQTPADVVSTVAAPMPMRAITSVLGVDGPDEAAFCRLS

NQAVRITDVALSASGLISLVQGFAGFRRLRALFTHRRDNGLLRECTVLGK

LATHAEQGRLSDDELFFFAVLLLVAGYESTAHMISTLFLTLADYPDQLTL

LAQQPDLIPSAIEEHLRFISPIQNICRTTRVDYSVGQAVIPAGSLVLLAW

GAANRDPRQYEDPDVFRADRNPVGHLAFGSGIHLCPGTQLARMEGQAILR

EIVANIDRIEVVEPPTWTTNANLRGLTRLRVAVTPRVAP

>CYP128A1 (641783875) Mycobacterium tuberculosis str. Haarlem

MQLTDFDPFDPAIAADPYPHYRELLAGERVQYNPKRDVYILSRYADVREA

ARNHDTLSSARGVTFSRGWLPFLPTSDPPAHTRMRKQLAPGMARGALETW

RPMVDQLARELVGGLLTQTPADVVSTVAAPMPMRAITSVLGVDGPDEAAF

CRLSNQAVRITDVALSASGLISLVQGFAGFRRLRALFTHRRDNGLLRECT

VLGKLATHAEQGRLSDDELFFFAVLLLVAGYESTAHMISTLFLTLADYPD

QLTLLAQQPDLIPSAIEEHLRFISPIQNICRTTRVDYSVGQAGIPAGSLV

LLAWGAANRDPRQYEDPDVFRADRNPVGHLAFGSGIHLCPGPSWRAWRVR

RSCARSSPISTE

>CYP128A1 (651037922) Mycobacterium canettii CIPT 140010059

MTATRSSAEHPSAEVAQGGCPFDRGVRPALALKGAAKPGESVRWRASSRV

SLPVSATLRRDVQLARLYAPMLAKTLALTLARKARSKLVVEHDPKHVAIT

DFDPFDPTVAHDPYPHYRALLAGPRVHYNPKRDVYILSRYADVRAAARNH

DVLSSAGGVTYSRLQLPFLPTSDPPEHTRMRKQLRPAFTRSALESWRPTI

DQLAQELIARLMTQAGADVVSTVAAPIAMRTITHILGVSGPDQAAFRDWS

SQAARMTNINLSASGLFSLARTFNGFRHLHAFFTQRLRHDGPLRVETVLE

RLAAQADDGALSDEELFFFAVLLLVAGYESTANLLSTLFLTPASWPDQLR

LLAQRPELIPSAIEEQLRFASPIQNICRTTRVDYPVGRAVIPKGSLVLLA

WGAANRDPRQFDDPDVFRADRNPTAHVAFGSGIHSCPGAQLARMEGQAVL

REIVENIERIEVVEPPRWSTNANLRGLTRLRVSVTRRTPASGPSQI

>CYP128B4 (2796830832) Mycobacteroides abscessus abscessus 41

MSRWRSDSAVRQIGAPVVAAVGMNIAAAVRVRRRGYAGWTGAVNTDYDPL

DPATAAQPFDAYRALHAGGRVHYNPKRATFILSRHEDIRAALRDTDAVTS

SQGVTRMKISAPILVLTDGDDHTRLRKQVQPGFTRGAMSDWQGMADQLAK

ELVADVVANPGCDVMERLAVPLPIRMIAHIIGIPPEDVQNFRSWSEDGVG

VINAGVSPAGLRQGLKGVRAIAALRRYFKDQLASGKLKGSDTVLGRLVDN

NEDGKLSDDELFFIAMLLLFAGNETTTNLIGGMFDTLAHAPDQFAMIRDD

PDLIPSAVEEQLRYSAPIQNLYRYTRTDYRVGEVTIPSGSRLLLAFGAAN

RDPEVFEDPDTYRADRNPRNHIAFGYGVHMCIGATLSRMEGQAVLRELTS

QASAIAAAGSATWSTNSSLRGTTYLPIRLTPAR

>CYP128B4 (2798746170) Mycobacteroides abscessus abscessus 367

MSRWRSDSAVRQIGAPVVAAVGMNIAAAVRVRRRGYAGWTGAVNTDYDPL

DPATAAQPFDAYRALHAGGRVHYNPKRATFILSRHEDIRAALRDTDAVTS

SQGVTRMKISAPILVLTDGDDHTRLRKQVQPGFTRGAMSDWQGMADQLAK

ELVADVVANPGCDVMERLAVPLPIRMIAHIIGIPPEDVQNFRSWSEDGVG

VINAGVSPAGLRQGLKGVRAIAALRRYFKDQLASGKLKGSDTVLGRLVDN

NEDGKLSDDELFFIAMLLLFAGNETTTNLIGGMFDTLAHAPDQFAMIRDD

PDLIPSAVEEQLRYSAPIQNLYRYTRTDYRVGEVTIPSGSRLLLAFGAAN

RDPEVFEDPDTYRADRNPRNHIAFGYGVHMCIGATLSRMEGQAVLRELTS

QASAIAAAGSATWSTNSSLRGTTYLPIRLTPAR

>CYP128B4 (2797965341) Mycobacteroides abscessus abscessus 1160

MSRWRSDSAVRQIGAPVVAAVGMNIAAAVRVRRRGYAGWTGAVNTDYDPL

DPATAAQPFDAYRALHAGGRVHYNPKRATFILSRHEDIRAALRDTDAVTS

SQGVTRMKISAPILVLTDGDDHTRLRKQVQPGFTRGAMSDWQGMADQLAK

ELVADVVANPGCDVMERLAVPLPIRMIAHIIGIPPEDVQNFRSWSEDGVG

VINAGVSPAGLRQGLKGVRAIAALRRYFKDQLASGKLKGSDTVLGRLVDN

NEDGKLSDDELFFIAMLLLFAGNETTTNLIGGMFDTLAHAPDQFAMIRDD

PDLIPSAVEEQLRYSAPIQNLYRYTRTDYRVGEVTIPSGSRLLLAFGAAN

RDPEVFEDPDTHRADRNPRNHIAFGYGVHMCIGATLSRMEGQAVLRELTS

QASAIAAAGSATWSTNSSLRGTTYLPIRLTPAR

>CYP128B2 (2739332033) Mycobacterium sp. YR782

MTLTQDAHFAGMMVRRLSNAARMNFTAGLQARRLGRPVGGDVGLTNYDPL

DPSTSAHPHEWYRRLHDGPRVHYNSRRALWILTRADDVRAAARADTQLSS

ADGVTRSRFAAPILITTDGEQHAQMRRHVLPAFTKSALANWRIEIDRLAV

ELVQDVFDNPGCDIVQRLAVPMPTRLIARLLGVPDSDVDDFRRWSEGAIQ

ATELALTPRGMSKWPASIGGTHSIYRYFRRQFKAGGLKGSDTILGRLLTD

NEDGTVSDDELFFFAFLLLLAGNETTTNLLGGMFDAFARHPDQYDIVRQD

PSLIPMAVEELLRYISPVQNLYRTARTDYQVGDTTIPRGARVQLSFGAGN

RDPRVFEDPDAFLVRRNPTQHIAFGFGAHFCLGAQLTRFEAQAVLREVVA

RTTCISITSDTTWSTNSSLRGPASLPVRMIPS

>CYP128A1 (2546454208) Mycobacterium tuberculosis GuangZ0019

MQLTDFDPFDPAIAADPYPHYRELLAGERVQYNPKRDVYILSRYADVREA

ARNHDTLSSARGVTFSRGWLPFLPTSDPPAHTRMRKQLAPGMARGALETW

RPMVDQLARELVGGLLTQTPADVVSTVAAPMPMRAITSVLGVDGPDEAAF

CRLSNQAVRITDVALSASGLISLVQGFAGFRRLRALFTHRRDNGLLRECT

VLGKLATHAEQGRLSDDELFFFAVLLLVAGYESTAHMISTLFLTLADYPD

QLTLLAQQPDLIPSAIEEHLRFISPIQNICRTTRVDYSVGQAVIPAGSLV

LLAWGAANRDPRQYEDPDVFRADRNPVGHLAFGSGIHLCPGTQLARMEGQ

AILREIVANIDRIEVVEPPTWTTNANLRGLTRLRVAVTPRVAP

>CYP128A1 (2604586353) Mycobacterium tuberculosis BTB10-277

MTATQSPPEPAPDRVRLAGCPLAGTPDVGLTAQDATTALGVPTRRRASSG

GIPVATSMWRDAQTVRTYGPAVAKALALRVAGKARSRLTGRHCRKFMQLT

DFDPFDPAIAADPYPHYRELLAGERVQYNPKRDVYILSRYADVREAARNH

DTLSSARGVTFSRGWLPFLPTSDPPAHTRMRKQLAPGMARGALETWRPMV

DQLARELVGGLLTQTPADVVSTVAAPMPMRAITSVLGVDGPDEAAFCRLS

NQAVRITDVALSASGLISLVQGFAGFRRLRALFTHRRDNGLLRECTVLGK

LATHAEQGRLSDDELFFFAVLLLVAGYESTAHMISTLFLTLADYPDQLTL

LAQQPDLIPSAIEEHLRFISPIQNICRTTRVDYSVGQAVIPAGSLVLLAW

GAANRDPRQYEDPDVFRADRNPVGHLAFGSGIHLCPGTQLARMEGQAILR

EIVANIDRIEVVEPPTWTTNANLRGLTRLRVAVTPRVAP

>CYP128A1 (2621576636) Mycobacterium tuberculosis XTB13-211

MTATQSPPEPAPDRVRLAGCPLAGTPDVGLTAQDATTALGVPTRRRASSG

GIPVATSMWRDAQTVRTYGPAVAKALALRVAGKARSRLTGRHCRKFMQLT

DFDPFDPAIAADPYPHYRELLAGERVQYNPKRDVYILSRYADVREAARNH

DTLSSARGVTFSRGWLPFLPTSDPPAHTRMRKQLAPGMARGALETWRPMV

DQLARELVGGLLTQTPADVVSTVAAPMPMRAITSVLGVDGPDEAAFCRLS

NQAVRITDVALSASGLISLVQGFAGFRRLRALFTHRRDNGLLRECTVLGK

LATHAEQGRLSDDELFFFAVLLLVAGYESTAHMISTLFLTLADYPDQLTL

LAQQPDLIPSAIEEHLRFISPIQNICRTTRVDYSVGQAVIPAGSLVLLAW

GAANRDPRQYEDPDVFRADRNPVGHLAFGSGIHLCPGTQLARMEGQAILR

EIVANIDRIEVVEPPTWTTNANLRGLTRLRVAVTPRVAP

>CYP128A1 (2655076967) Mycobacterium tuberculosis NRITLD28

MTATQSPPEPAPDRVRLAGCPLAGTPDVGLTAQDATTALGVPTRRRASSG

GIPVATSMWRDAQTVRTYGPAVAKALALRVAGKARSRLTGRHCRKFMQLT

DFDPFDPAIAADPYPHYRELLAGERVQYNPKRDVYILSRYADVREAARNH

DTLSSARGVTFSRGWLPFLPTSDPPAHTRMRKQLAPGMARGALETWRPMV

DQLARELVGGLLTQTPADVVSTVAAPMPMRAITSVLGVDGPDEAAFCRLS

NQAVRITDVALSASGLISLVQGFAGFRRLRALFTHRRDNGLLRECTVLGK

LATHAEQGRLSDDELFFFAVLLLVAGYESTAHMISTLFLTLADYPDQLTL

LAQQPDLIPSAIEEHLRFISPIQNICRTTRVDYSVGQAVIPAGSLVLLAW

GAANRDPRQYEDPDVFRADRNPVGHLAFGSGIHLCPGTQLARMEGQAILR

EIVANIDRIEVVEPPTWTTNANLRGLTRLRVAVTPRVAP

>CYP128A1 (2659218031) Mycobacterium tuberculosis A70376

MTATQSPPEPAPDRVRLAGCPLAGTPDVGLTAQDATTALGVPTRRRASSG

GIPVATSMWRDAQTVRTYGPAVAKALALRVAGKARSRLTGRHCRKFMQLT

DFDPFDPAIAADPYPHYRELLAGERVQYNPKRDVYILSRYADVREAARNH

DTLSSARGVTFSRGWLPFLPTSDPPAHTRMRKQLAPGMARGALETWRPMV

DQLARELVGGLLTQTPADVVSTVAAPMPMRAITSVLGVDGPDEAAFCRLS

NQAVRITDVALSASGLISLVQGFAGFRRLRALFTHRRDNGLLRECTVLGK

LATHAEQGRLSDDELFFFAVLLLVAGYESTAHMISTLFLTLADYPDQLTL

LAQQPDLIPSAIEEHLRFISPIQNICRTTRVDYSVGQAVIPAGSLVLLAW

GAANRDPRQYEDPDVFRADRNPVGHLAFGSGIHLCPGTQLARMEGQAILR

EIVANIDRIEVVEPPTWTTNANLRGLTRLRVAVTPRVAP

>CYP128A1 (2665808659) Mycobacterium tuberculosis 201314

MTATQSPPEPAPDRVRLAGCPLAGTPDVGLTAQDATTALGVPTRRRASSG

GIPVATSMWRDAQTVRTYGPAVAKALALRVAGKARSRLTGRHCRKFMQLT

DFDPFDPAIAADPYPHYRELLAGERVQYNPKRDVYILSRYADVREAARNH

DTLSSARGVTFSRGWLPFLPTSDPPAHTRMRKQLAPGMARGALETWRPMV

DQLARELVGGLLTQTPADVVSTVAAPMPMRAITSVLGVDGPDEAAFCRLS

NQAVRITDVALSASGLISLVQGFAGFRRLRALFTHRRDNGLLRECTVLGK

LATHAEQGRLSDDELFFFAVLLLVAGYESTAHMISTLFLTLADYPDQLTL

LAQQPDLIPSAIEEHLRFISPIQNICRTTRVDYSVGQAVIPAGSLVLLAW

GAANRDPRQYEDPDVFRADRNPVGHLAFGSGIHLCPGTQLARMEGQAILR

EIVANIDRIEVVEPPTWTTNANLRGLTRLRVAVTPRVAP

>CYP128A1 (2703496927) Mycobacterium tuberculosis J09701231

MTATQSPPEPAPDRVRLAGCPLAGTPDVGLTAQDATTALGVPTRRRASSG

GIPVATSMWRDAQTVRTYGPAVAKALALRVAGKARSRLTGRHCRKFMQLT

DFDPFDPAIAADPYPHYRELLAGERVQYNPKRDVYILSRYADVREAARNH

DTLSSARGVTFSRGWLPFLPTSDPPAHTRMRKQLAPGMARGALETWRPMV

DQLARELVGGLLTQTPADVVSTVAAPMPMRAITSVLGVDGPDEAAFCRLS

NQAVRITDVALSASGLISLVQGFAGFRRLRALFTHRRDNGLLRECTVLGK

LATHAEQGRLSDDELFFFAVLLLVAGYESTAHMISTLFLTLADYPDQLTL

LAQQPDLIPSAIEEHLRFISPIQNICRTTRVDYSVGQAVIPAGSLVLLAW

GAANRDPRQYEDPDVFRADRNPVGHLAFGSGIHLCPGTQLARMEGQAILR

EIVANIDRIEVVEPPTWTTNANLRGLTRLRVAVTPRVAP

>CYP128A1 (2706588020) Mycobacterium tuberculosis V09601310

MTATQSPPEPAPDRVRLAGCPLAGTPDVGLTAQDATTALGVPTRRRASSG

GIPVATSMWRDAQTVRTYGPAVAKALALRVAGKARSRLTGRHCRKFMQLT

DFDPFDPAIAADPYPHYRELLAGERVQYNPKRDVYILSRYADVREAARNH

DTLSSARGVTFSRGWLPFLPTSDPPAHTRMRKQLAPGMARGALETWRPMV

DQLARELVGGLLTQTPADVVSTVAAPMPMRAITSVLGVDGPDEAAFCRLS

NQAVRITDVALSASGLISLVQGFAGFRRLRALFTHRRDNGLLRECTVLGK

LATHAEQGRLSDDELFFFAVLLLVAGYESTAHMISTLFLTLADYPDQLTL

LAQQPDLIPSAIEEHLRFISPIQNICRTTRVDYSVGQAVIPAGSLVLLAW

GAANRDPRQYEDPDVFRADRNPVGHLAFGSGIHLCPGPSWRAWRVRRSCA

RSSPISTE

>CYP128A1 (2578093241) Mycobacterium tuberculosis NRITLD15

MTATQSPPEPAPDRVRLAGCPLAGTPDVGLTAQDATTALGVPTRRRAASG

GIPVATSMWRDAQTVRTYGPAVAKALALRVAGKARSRLTGRHCRKFMQLT

DFDPFDPAIAADPYPHYRELLAGERVQYNPKRDVYILSRYADVREAARNH

DTLSSARGVTFSRGWLPFLPTSDPPAHTRMRKQLAPGMARGALETWRPMV

DQLARELVGGLLTQTPADVVSTVAAPMPMRAITSVLGVDGPDEAAFCRLS

NQAVRITDVALSASGLISLVQGFAGFRRLRALFTHRRDNGLLRECTVLGK

LATHAEQGRLSDDELFFFAVLLLVAGYESTAHMISTLFLTLADYPDQLTL

LAQQPDLIPSAIEEHLRFISPIQNICRTTRVDYSVGQAVIPAGSLVLLAW

GAANRDPRQYEDPDVFRADRNPVGHLAFGSGIHLCPGTQLARMEGQAILR

EIVANIDRIEVVEPPTWTTNANLRGLTRLRVAVTPRVAP

>CYP128A1 (2621467506) Mycobacterium tuberculosis XTB13-145

MTATQSPPEPAPDRVRLAGCPLAGTPDVGLTAQDATTALGVPTRRRAASG

GIPVATSMWRDAQTVRTYGPAVAKALALRVAGKARSRLTGRHCRKFMQLT

DFDPFDPAIAADPYPHYRELLAGERVQYNPKRDVYILSRYADVREAARNH

DTLSSARGVTFSRGWLPFLPTSDPPAHTRMRKQLAPGMARGALETWRPMV

DQLARELVGGLLTQTPADVVSTVAAPMPMRAITSVLGVDGPDEAAFCRLS

NQAVRITDVALSASGLISLVQGFAGFRRLRALFTHRRDNGLLRECTVLGK

LATHAEQGRLSDDELFFFAVLLLVAGYESTAHMISTLFLTLADYPDQLTL

LAQQPDLIPSAIEEHLRFISPIQNICRTTRVDYSVGQAVIPAGSLVLLAW

GAANRDPRQYEDPDVFRADRNPVGHLAFGSGIHLCPGTQLARMEGQAILR

EIVANIDRIEVVEPPTWTTNANLRGLTRLRVAVTPRVAP

>CYP128A1 (2622466742) Mycobacterium tuberculosis M1317

MTATQSPPEPAPDRVRLAGCPLAGTPDVGLTAQDATTALGVPTRRRASSG

GIPVATSMWRDAQTVRTYGPAVAKALALRVAGKARSRLTGRHCRKFMQLT

DFDPFDPAIAADPYPHYRELLAGERVQYNPKRDVYILSRYADVREAARNH

DTLSSARGVTFSRGWLPFLPTSDPPAHTRMRKQLAPGMARGALETWRPMV

DQLARELVGGLLTQTPADVVSTVAAPMPMRAITSVLGVDGPDEAAFCRLS

NQAVRITDVALSASGLISLVGFAGFRRLRALFTHRRDNGLLRECTVLGKL

ATHAEQGRLSDDELFFFAVLLLVAGYESTAHMISTLFLTLADYPDQLTLL

AQQPDLIPSAIEEHLRFISPIQNICRTTRVDYSVGQAVIPAGSLVLLAWG

AANRDPRQYEDPDVFRADRNPVGHLAFGSGIHLCPGTQLARMEGQAILRE

IVANIDRIEVVEPPTWTTNANLRGLTRLRVAVTPRVAP

>CYP128B4 (2798675136) Mycobacteroides abscessus abscessus 88

MSRWRSDSAVRQIGAPVVAAVGMNIAAAVRVRRRGYAGWTGAVNTDYDPL

DPATAAQPFDAYRALHAGGRVHYNPKRATFILSRHEDIRAALRDTDAVTS

SQGVTRMKISAPILVLTDGDDHTRLRKQVQPGFTRGAMSDWQGMADQLAK

ELVADVVANPGCDVMERLAVPLPIRMIAHIIGIPPEDVQNFRSWSEDGVG

VINAGVSPAGLRQGLKGVRAIAALRRYFKDQLASGKLKGSDTVLGRLVDN

NEDGKLSDDELFFIAMLLLFAGNETTTNLIGGMFDTLAHAPDQFAMIRDD

PDLIPSAVEEQLRYSAPIQNLYRYTRTDYRVGEVTIPSGSRLLLAFGAAN

RDPEVFEDPDTYRADRNPRNHIAFGYGVHMCIGATLSRMEGQAVLRELTS

QASAIAAAGSATWSTNSSLRGTTYLPIRLTPAR

>CYP128B4 (2798771791) Mycobacteroides abscessus abscessus 248

MSRWRSDSAVRQIGAPVVAAVGMNIAAAVRVRRRGYAGWTGAVNTDYDPL

DPATAAQPFDAYRALHAGGRVHYNPKRATFILSRHEDIRAALRDTDAVTS

SQGVTRMKISAPILVLTDGDDHTRLRKQVQPGFTRGAMSDWQGMADQLAK

ELVADVVANPGCDVMERLAVPLPIRMIAHIIGIPPEDVQNFRSWSEDGVG

VINAGVSPAGLRQGLKGVRAIAALRRYFKDQLASGKLKGSDTVLGRLVDN

NEDGKLSDDELFFIAMLLLFAGNETTTNLIGGMFDTLAHAPDQFAMIRDD

PDLIPSAVEEQLRYSAPIQNLYRYTRTDYRVGEVTIPSGSRLLLAFGAAN

RDPEVFEDPDTYRADRNPRNHIAFGYGVHMCIGATLSRMEGQAVLRELTS

QASAIAAAGSATWSTNSSLRGTTYLPIRLTPAR

>CYP128B1 (2707835115) Mycobacterium sp. GPK 1020

MDVRGAVQGVVETVKPFAKAAHLNLSAAVRTRRRGYRGWTGAVNTDYDPL

DRATAAQPFDAYRALHRSGRVHYNPKRATWILSRLEDVRAALRDTDTVTS

AEGVTRLKFTAPLAVLTDGAEHARLRRQVQPGFSKGAMDSWQGMVDKLAT

ELVSEVLDEPGCDVVQQLAIPMPIRMIAQILGIPECDVGDFRRWSEDGVR

IMDFSPTPQGIVGAAKSIASMAALRRYFLQQFATGGLKGSGTVLGRLLAH

NTDGSLTDEQLFLIAIHLLIAGNETTTNLLGGMFDTFARNPDQYDMVRSN

PDLIPMAVEEMLRITTPIQNLYRYTRADYVVGGVTIPTGSRLLLSFGAAN

RDPLAFEEPDEYHADRNPKMHVAFGYGAHMCLGAPLARMEAHAVLRELVS

RVSRISAVGETTWSTSSSLRGPTHLPIQLTPA

>CYP128A1 (2574798349) Mycobacterium tuberculosis TKK_05MA_0020

MTATQSPPEPAPDRVRLAGCPLAGTPDVGLTAQDATTALGVPTRRRASSG

GIPVATSMWRDAQTVRTYGPAVAKALALRVAGKARSRLTGRHCRKFMQLT

DFDPFDPAIAADPYPHYRELLAGERVQYNPKRDVYILSRYADVREAARNH

DTLSSARGVTFSRGWLPFLPTSDPPAHTRMRKQLAPGMARGALETWRPMV

DQLARELVGGLLTQTPADVVSTVAAPMPMRAITSVLGVDGPDEAAFCRLS

NQAVRITDVALSASGLISLVQGFAGFRRLRALFTHRRDNGLLRECTVLGK

LATHAEQGRLSDDELFFFAVLLLVAGYESTAHMISTLFLTLADYPDQLTL

LAQQPDLIPSAIEEHLRFISPIQNICRTTRVDYSVGQAVIPAGSLVLLAW

GAANRDPRQYEDPDVFRADRNPVGHLAFGSGIHLCPGTQLARMEGQAILR

EIVANIDRIEVVEPPTWTTNANLRGLTRLRVAVTPRVAP

>CYP128A1 (2577883715) Mycobacterium tuberculosis M1017

MTATQSPPEPAPDRVRLAGCPLAGTPDVGLTAQDATTALGVPTRRRASSG

GIPVATSMWRDAQTVRTYGPAVAKALALRVAGKARSRLTGRHCRKFMQLT

DFDPFDPAIAADPYPHYRELLAGERVQYNPKRDVYILSRYADVREAARNH

DTLSSARGVTFSRGWLPFLPTSDPPAHTRMRKQLAPGMARGALETWRPMV

DQLARELVGGLLTQTPADVVSTVAAPMPMRAITSVLGVDGPDEAAFCRLS

NQAVRITDVALSASGLISLVQGFAGFRRLRALFTHRRDNGLLRECTVLGK

LATHAEQGRLSDDELFFFAVLLLVAGYESTAHMISTLFLTLADYPDQLTL

LAQQPDLIPSAIEEHLRFISPIQNICRTTRVDYSVGQAVIPAGSLVLLAW

GAANRDPRQYEDPDVFRADRNPVGHLAFGSGIHLCPGTQLARMEGQAILR

EIVANIDRIEVVEPPTWTTNANLRGLTRLRVAVTPRVAP

>CYP128A1 (2579814399) Mycobacterium tuberculosis TBR10

MTATQSPPEPAPDRVRLAGCPLAGTPDVGLTAQDATTALGVPTRRRASSG

GIPVATSMWRDAQTVRTYGPAVAKALALRVAGKARSRLTGRHCRKFMQLT

DFDPFDPAIAADPYPHYRELLAGERVQYNPKRDVYILSRYADVREAARNH

DTLSSARGVTFSRGWLPFLPTSDPPAHTRMRKQLAPGMARGALETWRPMV

DQLARELVGGLLTQTPADVVSTVAAPMPMRAITSVLGVDGPDEAAFCRLS

NQAVRITDVALSASGLISLVQGFAGFRRLRALFTHRRDNGLLRECTVLGK

LATHAEQGRLSDDELFFFAVLLLVAGYESTAHMISTLFLTLADYPDQLTL

LAQQPDLIPSAIEEHLRFISPIQNICRTTRVDYSVGQAVIPAGSLVLLAW

GAANRDPRQYEDPDVFRADRNPVGHLAFGSGIHLCPGTQLARMEGQAILR

EIVANIDRIEVVEPPTWTTNANLRGLTRLRVAVTPRVAP

>CYP128A1 (2584894414) Mycobacterium tuberculosis TB_RSA107

MTATQSPPEPAPDRVRLAGCPLAGTPDVGLTAQDATTALGVPTRRRASSG

GIPVATSMWRDAQTVRTYGPAVAKALALRVAGKARSRLTGRHCRKFMQLT

DFDPFDPAIAADPYPHYRELLAGERVQYNPKRDVYILSRYADVREAARNH

DTLSSARGVTFSRGWLPFLPTSDPPAHTRMRKQLAPGMARGALETWRPMV

DQLARELVGGLLTQTPADVVSTVAAPMPMRAITSVLGVDGPDEAAFCRLS

NQAVRITDVALSASGLISLVQGFAGFRRLRALFTHRRDNGLLRECTVLGK

LATHAEQGRLSDDELFFFAVLLLVAGYESTAHMISTLFLTLADYPDQLTL

LAQQPDLIPSAIEEHLRFISPIQNICRTTRVDYSVGQAVIPAGSLVLLAW

GAANRDPRQYEDPDVFRADRNPVGHLAFGSGIHLCPGTQLARMEGQAILR

EIVANIDRIEVVEPPTWTTNANLRGLTRLRVAVTPRVAP

>CYP128A1 (2589153891) Mycobacterium tuberculosis OFXR-11

MTATQSPPEPAPDRVRLAGCPLAGTPDVGLTAQDATTALGVPTRRRASSG

GIPVATSMWRDAQTVRTYGPAVAKALALRVAGKARSRLTGRHCRKFMQLT

DFDPFDPAIAADPYPHYRELLAGERVQYNPKRDVYILSRYADVREAARNH

DTLSSARGVTFSRGWLPFLPTSDPPAHTRMRKQLAPGMARGALETWRPMV

DQLARELVGGLLTQTPADVVSTVAAPMPMRAITSVLGVDGPDEAAFCRLS

NQAVRITDVALSASGLISLVQGFAGFRRLRALFTHRRDNGLLRECTVLGK

LATHAEQGRLSDDELFFFAVLLLVAGYESTAHMISTLFLTLADYPDQLTL

LAQQPDLIPSAIEEHLRFISPIQNICRTTRVDYSVGQAVIPAGSLVLLAW

GAANRDPRQYEDPDVFRADRNPVGHLAFGSGIHLCPGTQLARMEGQAILR

EIVANIDRIEVVEPPTWTTNANLRGLTRLRVAVTPRVAP

>CYP128A1 (2592378082) Mycobacterium tuberculosis TKK_02_0039

MTATQSPPEPAPDRVRLAGCPLAGTPDVGLTAQDATTALGVPTRRRASSG

GIPVATSMWRDAQTVRTYGPAVAKALALRVAGKARSRLTGRHCRKFMQLT

DFDPFDPAIAADPYPHYRELLAGERVQYNPKRDVYILSRYADVREAARNH

DTLSSARGVTFSRGWLPFLPTSDPPAHTRMRKQLAPGMARGALETWRPMV

DQLARELVGGLLTQTPADVVSTVAAPMPMRAITSVLGVDGPDEAAFCRLS

NQAVRITDVALSASGLISLVQGFAGFRRLRALFTHRRDNGLLRECTVLGK

LATHAEQGRLSDDELFFFAVLLLVAGYESTAHMISTLFLTLADYPDQLTL

LAQQPDLIPSAIEEHLRFISPIQNICRTTRVDYSVGQAVIPAGSLVLLAW

GAANRDPRQYEDPDVFRADRNPVGHLAFGSGIHLCPGTQLARMEGQAILR

EIVANIDRIEVVEPPTWTTNANLRGLTRLRVAVTPRVAP

>CYP128A1 (2603192948) Mycobacterium tuberculosis KT-0033

MTATQSPPEPAPDRVRLAGCPLAGTPDVGLTAQDATTALGVPTRRRASSG

GIPVATSMWRDAQTVRTYGPAVAKALALRVAGKARSRLTGRHCRKFMQLT

DFDPFDPAIAADPYPHYRELLAGERVQYNPKRDVYILSRYADVREAARNH

DTLSSARGVTFSRGWLPFLPTSDPPAHTRMRKQLAPGMARGALETWRPMV

DQLARELVGGLLTQTPADVVSTVAAPMPMRAITSVLGVDGPDEAAFCRLS

NQAVRITDVALSASGLISLVQGFAGFRRLRALFTHRRDNGLLRECTVLGK

LATHAEQGRLSDDELFFFAVLLLVAGYESTAHMISTLFLTLADYPDQLTL

LAQQPDLIPSAIEEHLRFISPIQNICRTTRVDYSVGQAVIPAGSLVLLAW

GAANRDPRQYEDPDVFRADRNPVGHLAFGSGIHLCPGTQLARMEGQAILR

EIVANIDRIEVVEPPTWTTNANLRGLTRLRVAVTPRVAP

>CYP128A1 (2604239934) Mycobacterium tuberculosis BTB11-289

MTATQSPPEPAPDRVRLAGCPLAGTPDVGLTAQDATTALGVPTRRRASSG

GIPVATSMWRDAQTVRTYGPAVAKALALRVAGKARSRLTGRHCRKFMQLT

DFDPFDPAIAADPYPHYRELLAGERVQYNPKRDVYILSRYADVREAARNH

DTLSSARGVTFSRGWLPFLPTSDPPAHTRMRKQLAPGMARGALETWRPMV

DQLARELVGGLLTQTPADVVSTVAAPMPMRAITSVLGVDGPDEAAFCRLS

NQAVRITDVALSASGLISLVQGFAGFRRLRALFTHRRDNGLLRECTVLGK

LATHAEQGRLSDDELFFFAVLLLVAGYESTAHMISTLFLTLADYPDQLTL

LAQQPDLIPSAIEEHLRFISPIQNICRTTRVDYSVGQAVIPAGSLVLLAW

GAANRDPRQYEDPDVFRADRNPVGHLAFGSGIHLCPGTQLARMEGQAILR

EIVANIDRIEVVEPPTWTTNANLRGLTRLRVAVTPRVAP

>CYP128A1 (2668926321) Mycobacterium tuberculosis A70596

MTATQSPPEPAPDRVRLAGCPLAGTPDVGLTAQDATTALGVPTRRRASSG

GIPVATSMWRDAQTVRTYGPAVAKALALRVAGKARSRLTGRHCRKFMQLT

DFDPFDPAIAADPYPHYRELLAGERVQYNPKRDVYILSRYADVREAARNH

DTLSSARGVTFSRGWLPFLPTSDPPAHTRMRKQLAPGMARGALETWRPMV

DQLARELVGGLLTQTPADVVSTVAAPMPMRAITSVLGVDGPDEAAFCRLS

NQAVRITDVALSASGLISLVQGFAGFRRLRALFTHRRDNGLLRECTVLGK

LATHAEQGRLSDDELFFFAVLLLVAGYESTAHMISTLFLTLADYPDQLTL

LAQQPDLIPSAIEEHLRFISPIQNICRTTRVDYSVGQAVIPAGSLVLLAW

GAANRDPRQYEDPDVFRADRNPVGHLAFGSGIHLCPGTQLARMEGQAILR

EIVANIDRIEVVEPPTWTTNANLRGLTRLRVAVTPRVAP

>CYP128A1 (2674819932) Mycobacterium tuberculosis T09900518

MTATQSPPEPAPDRVRLAGCPLAGTPDVGLTAQDATTALGVPTRRRASSG

GIPVATSMWRDAQTVRTYGPAVAKALALRVAGKARSRLTGRHCRKFMQLT

DFDPFDPAIAADPYPHYRELLAGERVQYNPKRDVYILSRYADVREAARNH

DTLSSARGVTFSRGWLPFLPTSDPPAHTRMRKQLAPGMARGALETWRPMV

DQLARELVGGLLTQTPADVVSTVAAPMPMRAITSVLGVDGPDEAAFCRLS

NQAVRITDVALSASGLISLVQGFAGFRRLRALFTHRRDNGLLRECTVLGK

LATHAEQGRLSDDELFFFAVLLLVAGYESTAHMISTLFLTLADYPDQLTL

LAQQPDLIPSAIEEHLRFISPIQNICRTTRVDYSVGQAVIPAGSLVLLAW

GAANRDPRQYEDPDVFRADRNPVGHLAFGSGIHLCPGTQLARMEGQAILR

EIVANIDRIEVVEPPTWTTNANLRGLTRLRVAVTPRVAP

>CYP128A1 (651088657) Mycobacterium tuberculosis CCDC5180

MQLTDFDPFDPAIAADPYPHYRELLAGERVQYNPKRDVYILSRYADVREA

ARNHDTLSSARGVTFSRGWLPFLPTSDPPAHTRMRKQLAPGMARGALETW

RPMVDQLARELVGGLLTQTPADVVSTVAAPMPMRAITSVLGVDGPDEAAF

CRLSNQAVRITDVALSASGLISLVQGFAGFRRLRALFTHRRDNGLLRECT

VLGKLATHAEQGRLSDDELFFFAVLLLVAGYESTAHMISTLFLTLADYPD

QLTLLAQQPDLIPSAIEEHLRFISPIQNICRTTRVDYSVGQAVIPAGSLV

LLAWGAANRDPRQYEDPDVFRADRNPVGHLAFGSGIHLCPGTQLARMEGQ

AILREIVANIDRIEVVEPPTWTTNANLRGLTRLRVAVTPRVAP

>CYP128A1 (2547305801) Mycobacterium bovis BCG China

MTATQSPPEPAPDRVRLAGCPLAGTPDVGLTAQDATTALGVPTRRRASSG

GIPVATSMWRDAQTVRTYGPAVAKALALRVAGKARSRLTGRHCRKFMQLT

DFDPFDPAIAADPYPHYRELLAGERVQYNPKRDVYILSRYADVREAARNH

DTLSSARGVTFSRGWLPFLPTSDPPAHTRMRKQLAPGMARGALETWRPMV

DQFARELVGGLLTQTPADVVSTVAAPMPMRAITSVLGVDGPDEAAFCRLS

NQAVRITDVALSASGLISLVQGFAGFRRLRALFTHRRDNGLLRECTVLGK

LATHAEQGRLSDDELFFFAVLLLVAGYESTAHMISTLFLTLADYPDQLTL

LAQQPDLIPSAIEEHLRFISPIQNICRTTRVDYSVGQAVIPAGSLVLLAW

GAANRDPRQYEDPDVFRADRNPVGHLAFGSGIHLCPGTQLARMEGQAILR

EIVANIDRIEVVEPPTWTTNANLRGLTRLRVAVTPRVAP

>CYP128A1 (2622605622) Mycobacterium tuberculosis M1560

MTATQSPPEPAPDRVRLAGCPLAGTPDVGLTAQDATTALGVPTRRRASSG

GIPVATSMWRDAQTVRTYGPAVAKALALRVAGKARSRLTGRHCRKFMQLT

DFDPFDPAIAADPYPHYRELLAGERVQYNPKRDVYILSRYADVREAARNH

DTLSSSRGVTFSRGWLPFLPTSDPPAHTRMRKQLAPGMARGALETWRPMV

DQLARELVGGLLTQTPADVVSTVAAPMPMRAITSVLGVDGPDEAAFCRLS

NQAVRITDVALSASGLISLVQGFAGFRRLRALFTHRRDNGLLRECTVLGK

LATHAEQGRLSDDELFFFAVLLLVAGYESTAHMISTLFLTLADYPDQLTL

LAQQPDLIPSAIEEHLRFISPIQNICRTTRVDYSVGQAVIPAGSLVLLAW

GAANRDPRQYEDPDVFRADRNPVGHLAFGSGIHLCPGTQLARMEGQAILR

EIVANIDRIEVVEPPTWTTNANLRGLTRLRVAVTPRVAP

>CYP128B4 (2803677220) Mycobacteroides abscessus abscessus 1132

MSRWRSDSAVRQIGAPVVAAVGMNIAAAVRVRRRGYAGWTGAVNTDYDPL

DPATAAQPFDAYRALHAGGRVHYNPKRATFILSRHEDIRAALRDTDAVTS

SQGVTRMKISAPILVLTDGDDHTRLRKQVQPGFTRGAMSDWQGMADQLAK

ELVADVVANPGCDVMERLAVPLPIRMIAHIIGIPPEDVQNFRSWSEDGVG

VINAGVSPAGLRQGLKGVRAIAALRRYFKDQLASGKLKGSDTVLGRLVDN

NEDGKLSDDELFFIAMLLLFAGNETTTNLIGGMFDTLAHAPDQFAMIRDD

PDLIPSAVEEQLRYSAPIQNLYRYTRTDYRVGEVTIPSGSRLLLAFGAAN

RDPEVFEDPDTYRADRNPRNHIAFGYGVHMCIGATLSRMEGQAVLRELTS

QASAIAAAGSATWSTNSSLRGTTYLPIRLTPAR

>CYP128B2 (2678751950) Mycobacterium avium hominissuis MAH-P-10203-06

LTLSQDVRFLGTMAQPLSHAARVNTARAFRSRWRQYRFWTGAQITDYDPI

DPANIAQPDAAYRALHAGGRVHYNPKLGLWILTRLPDVRAGARAAETLSS

ADGVTRLRMAGPLLVTMDGKPHNEMRRHVLPAFTKAALESWQAMIDDLAA

KLVGEVLDNPGCDVVQRLAIPMPMLLIAHMLGIPNGDIDDFRRWSTAAVK

MADVDISRRGLTRLKSSIGGIRDIYRYFRQQFAVGGLKGSDTLLGKLLSV

NASGSIDDNELFFFAMLLLIAGNETTTNLLGGMFDIFARHPEKFDMVRAD

HSLIPKVVEEQLRFSSPVQMLYRTARTNYEVGPVTIPAGARVLLSLGAAN

RDPQVFDEPDEFRVDRNPTEHLAFGFGAHLCLGAQLTRMEAQAVLRELVT

RARRIEAIGETQWSTGYLLRGPERMNVRLTPSVVAG

>CYP128B2 (2724972025) Mycobacterium avium hominissuis MAH-E-63-1

LTLSQDVRFLGTMAQPLSHAARVNTARAFRSRWRQYRFWTGAQITDYDPI

DPANIAQPDAAYRALHAGGRVHYNPKLGLWILTRLPDVRAGARAAETLSS

ADGVTRLRMAGPLLVTMDGKPHNEMRRHVLPAFTKAALESWQAMIDELAA

KLVGEVLDNPGCDVVQRLAIPMPMLLIAHMLGIPDGDIDDFRRWSSDAVK

MADVDISRRGLTRLKSSIGGIRDIYRYFRQQFAVGGLKGSDTLLGKLLSV

NASGSIDDNELFFFAMLLLIAGNETTTNLLGGMFDIFARHPEKFDMVRAD

HSLIPKVVEEQLRFSSPVQMLYRTARTNYEVGPVTIPAGARVLLSLGAAN

RDPQVFDEPDEFRVDRNPTEHLAFGFGAHLCLGAQLTRMEAQAVLRELVT

RARRIEAIGETQWSTGYLLRGPERMNVRLTPSVVAG

>CYP128B1 (2809694708) Mycobacterium sp. E188

MAAKQTIQGAAEGARLLGHAVRMNVAAAVRTRRRGRAGWTGAVNTDYDPQ

DPATAADPYDAYRALHRSGRVHYNPRRATFIVSRLDDVRAALRDTDRVTS

TQGVTRLRMSAPLAVLTDGEEHTRLRKQVQPGFSKGAMRSWQEMTEKLAI

DLVTDVMDDPGCDVVQRLAIPMPIRLIAQILGVPDSDAGDFRRWSERAVG

VMELRPTFAGIVDAAKSATGMVALWRYFTAQFAAGGLKGSTTVLGRLIEH

NTDGSLTDRQLLLIAIHLLIAGNETTTNLLGGMFDTLARRPDQYDLIRAN

PDLIPLAVEEQLRFTTPIQNLYRYTRADYRIGDVTIPTGSRVLLSFGAAN

RDPSAFDDPDEYRADRDPRTHVAFGYGAHMCLGAPLARMEAQAVLRQLVS

RVARITPAGPTQWSRHSSLRGPTRLPIRLTPA

>CYP128A1 (2560452212) Mycobacterium tuberculosis WX1

MTATQSPPEPAPDRVRLAGCPLAGTPDVGLTAQDATTALGVPTRRRASSG

GIPVATSMWRDAQTVRTYGPAVAKALALRVAGKARSRLTGRHCRKFMQLT

DFDPFDPAIAADPYPHYRELLAGERVQYNPKRDVYILSRYADVREAARNH

DTLSSARGVTFSRGWLPFLPTSDPPAHTRMRKQLAPGMARGALETWRPMV

DQLARELVGGLLTQTPADVVSTVAAPMPMRAITSVLGVDGPDEAAFCRLS

NQAVRITDVALSASGLISLVQGFAGFRRLRALFTHRRDNGLLRECTVLGK

LATHAEQGRLSDDELFFFAVLLLVAGYESTAHMISTLFLTLADYPDQLTL

LAQQPDLIPSAIEEHLRFISPIQNICRTTRVDYSVGQAVIPAGSLVLLAW

GAANRDPRQYEDPDVFRADRNPVGHLAFGSGIHLCPGTQLARMEGQAILR

EIVANIDRIEVVEPPTWTTNANLRGLTRLRVAVTPRVAP

>CYP128A1 (2570709327) Mycobacterium tuberculosis OM-V02_005

MTATQSPPEPAPDRVRLAGCPLAGTPDVGLTAQDATTALGVPTRRRASSG

GIPVATSMWRDAQTVRTYGPAVAKALALRVAGKARSRLTGRHCRKFMQLT

DFDPFDPAIAADPYPHYRELLAGERVQYNPKRDVYILSRYADVREAARNH

DTLSSARGVTFSRGWLPFLPTSDPPAHTRMRKQLAPGMARGALETWRPMV

DQLARELVGGLLTQTPADVVSTVAAPMPMRAITSVLGVDGPDEAAFCRLS

NQAVRITDVALSASGLISLVQGFAGFRRLRALFTHRRDNGLLRECTVLGK

LATHAEQGRLSDDELFFFAVLLLVAGYESTAHMISTLFLTLADYPDQLTL

LAQQPDLIPSAIEEHLRFISPIQNICRTTRVDYSVGQAVIPAGSLVLLAW

GAANRDPRQYEDPDVFRADRNPVGHLAFGSGIHLCPGTQLARMEGQAILR

EIVANIDRIEVVEPPTWTTNANLRGLTRLRVAVTPRVAP

>CYP128A1 (2577010225) Mycobacterium tuberculosis M1418

MTATQSPPEPAPDRVRLAGCPLAGTPDVGLTAQDATTALGVPTRRRASSG

GIPVATSMWRDAQTVRTYGPAVAKALALRVAGKARSRLTGRHCRKFMQLT

DFDPFDPAIAADPYPHYRELLAGERVQYNPKRDVYILSRYADVREAARNH

DTLSSARGVTFSRGWLPFLPTSDPPAHTRMRKQLAPGMARGALETWRPMV

DQLARELVGGLLTQTPADVVSTVAAPMPMRAITSVLGVDGPDEAAFCRLS

NQAVRITDVALSASGLISLVQGFAGFRRLRALFTHRRDNGLLRECTVLGK

LATHAEQGRLSDDELFFFAVLLLVAGYESTAHMISTLFLTLADYPDQLTL

LAQQPDLIPSAIEEHLRFISPIQNICRTTRVDYSVGQAVIPAGSLVLLAW

GAANRDPRQYEDPDVFRADRNPVGHLAFGSGIHLCPGTQLARMEGQAILR

EIVANIDRIEVVEPPTWTTNANLRGLTRLRVAVTPRVAP

>CYP128A1 (2577873217) Mycobacterium tuberculosis TB_RSA07

MTATQSPPEPAPDRVRLAGCPLAGTPDVGLTAQDATTALGVPTRRRASSG

GIPVATSMWRDAQTVRTYGPAVAKALALRVAGKARSRLTGRHCRKFMQLT

DFDPFDPAIAADPYPHYRELLAGERVQYNPKRDVYILSRYADVREAARNH

DTLSSARGVTFSRGWLPFLPTSDPPAHTRMRKQLAPGMARGALETWRPMV

DQLARELVGGLLTQTPADVVSTVAAPMPMRAITSVLGVDGPDEAAFCRLS

NQAVRITDVALSASGLISLVQGFAGFRRLRALFTHRRDNGLLRECTVLGK

LATHAEQGRLSDDELFFFAVLLLVAGYESTAHMISTLFLTLADYPDQLTL

LAQQPDLIPSAIEEHLRFISPIQNICRTTRVDYSVGQAVIPAGSLVLLAW

GAANRDPRQYEDPDVFRADRNPVGHLAFGSGIHLCPGTQLARMEGQAILR

EIVANIDRIEVVEPPTWTTNANLRGLTRLRVAVTPRVAP

>CYP128A1 (2581808303) Mycobacterium africanum MAL010123

MQLTDFDPFDPAIAADPYPHYRELLAGERVQYNPKRDVYILSRYADVREA

ARNHDTLSSARGVTFSRGWLPFLPTSDPPAHTRMRKQLAPGMARGALETW

RPMVDQLARELVGGLLTQTPADVVSTVAAPMPMRAITSVLGVDGPDEAAF

CRLSNQAVRITDVALSASGLISLVQGFAGFRRLRALFTHRRDNGLLRECT

VLGKLATHAEQGRLSDDELFFFAVLLLVAGYESTAHMISTLFLTLADYPD

QLTLLAQQPDLIPSAIEEHLRFISPIQNICRTTRVDYSVGQAVIPAGSLV

LLAWGAANRDPRQYEDPDVFRADRNPVGHLAFGSGIHLCPGTQLARMEGQ

AILREIVANIDRIEVVEPPTWTTNANLRGLTRLRVAVTPRVAP

>CYP128A1 (2589030367) Mycobacterium tuberculosis TBR8

MTATQSPPEPAPDRVRLAGCPLAGTPDVGLTAQDATTALGVPTRRRASSG

GIPVATSMWRDAQTVRTYGPAVAKALALRVAGKARSRLTGRHCRKFMQLT

DFDPFDPAIAADPYPHYRELLAGERVQYNPKRDVYILSRYADVREAARNH

DTLSSARGVTFSRGWLPFLPTSDPPAHTRMRKQLAPGMARGALETWRPMV

DQLARELVGGLLTQTPADVVSTVAAPMPMRAITSVLGVDGPDEAAFCRLS

NQAVRITDVALSASGLISLVQGFAGFRRLRALFTHRRDNGLLRECTVLGK

LATHAEQGRLSDDELFFFAVLLLVAGYESTAHMISTLFLTLADYPDQLTL

LAQQPDLIPSAIEEHLRFISPIQNICRTTRVDYSVGQAVIPAGSLVLLAW

GAANRDPRQYEDPDVFRADRNPVGHLAFGSGIHLCPGTQLARMEGQAILR

EIVANIDRIEVVEPPTWTTNANLRGLTRLRVAVTPRVAP

>CYP128A1 (2592243426) Mycobacterium tuberculosis TKK_04_0015

MTATQSPPEPAPDRVRLAGCPLAGTPDVGLTAQDATTALGVPTRRRASSG

GIPVATSMWRDAQTVRTYGPAVAKALALRVAGKARSRLTGRHCRKFMQLT

DFDPFDPAIAADPYPHYRELLAGERVQYNPKRDVYILSRYADVREAARNH

DTLSSARGVTFSRGWLPFLPTSDPPAHTRMRKQLAPGMARGALETWRPMV

DQLARELVGGLLTQTPADVVSTVAAPMPMRAITSVLGVDGPDEAAFCRLS

NQAVRITDVALSASGLISLVQGFAGFRRLRALFTHRRDNGLLRECTVLGK

LATHAEQGRLSDDELFFFAVLLLVAGYESTAHMISTLFLTLADYPDQLTL

LAQQPDLIPSAIEEHLRFISPIQNICRTTRVDYSVGQAVIPAGSLVLLAW

GAANRDPRQYEDPDVFRADRNPVGHLAFGSGIHLCPGTQLARMEGQAILR

EIVANIDRIEVVEPPTWTTNANLRGLTRLRVAVTPRVAP

>CYP128A1 (2603931199) Mycobacterium tuberculosis XDR KZN 1435

MTATQSPPEPAPDRVRLAGCPLAGTPDVGLTAQDATTALGVPTRRRASSG

GIPVATSMWRDAQTVRTYGPAVAKALALRVAGKARSRLTGRHCRKFMQLT

DFDPFDPAIAADPYPHYRELLAGERVQYNPKRDVYILSRYADVREAARNH

DTLSSARGVTFSRGWLPFLPTSDPPAHTRMRKQLAPGMARGALETWRPMV

DQLARELVGGLLTQTPADVVSTVAAPMPMRAITSVLGVDGPDEAAFCRLS

NQAVRITDVALSASGLISLVQGFAGFRRLRALFTHRRDNGLLRECTVLGK

LATHAEQGRLSDDELFFFAVLLLVAGYESTAHMISTLFLTLADYPDQLTL

LAQQPDLIPSAIEEHLRFISPIQNICRTTRVDYSVGQAVIPAGSLVLLAW

GAANRDPRQYEDPDVFRADRNPVGHLAFGSGIHLCPGTQLARMEGQAILR

EIVANIDRIEVVEPPTWTTNANLRGLTRLRVAVTPRVAP

>CYP128A1 (2621368850) Mycobacterium tuberculosis XTB13-089

MTATQSPPEPAPDRVRLAGCPLAGTPDVGLTAQDATTALGVPTRRRASSG

GIPVATSMWRDAQTVRTYGPAVAKALALRVAGKARSRLTGRHCRKFMQLT

DFDPFDPAIAADPYPHYRELLAGERVQYNPKRDVYILSRYADVREAARNH

DTLSSARGVTFSRGWLPFLPTSDPPAHTRMRKQLAPGMARGALETWRPMV

DQLARELVGGLLTQTPADVVSTVAAPMPMRAITSVLGVDGPDEAAFCRLS

NQAVRITDVALSASGLISLVQGFAGFRRLRALFTHRRDNGLLRECTVLGK

LATHAEQGRLSDDELFFFAVLLLVAGYESTAHMISTLFLTLADYPDQLTL

LAQQPDLIPSAIEEHLRFISPIQNICRTTRVDYSVGQAVIPAGSLVLLAW

GAANRDPRQYEDPDVFRADRNPVGHLAFGSGIHLCPGTQLARMEGQAILR

EIVANIDRIEVVEPPTWTTNANLRGLTRLRVAVTPRVAP

>CYP128A1 (2621730394) Mycobacterium tuberculosis 2230BH

MTATQSPPEPAPDRVRLAGCPLAGTPDVGLTAQDATTALGVPTRRRASSG

GIPVATSMWRDAQTVRTYGPAVAKALALRVAGKARSRLTGRHCRKFMQLT

DFDPFDPAIAADPYPHYRELLAGERVQYNPKRDVYILSRYADVREAARNH

DTLSSARGVTFSRGWLPFLPTSDPPAHTRMRKQLAPGMARGALETWRPMV

DQLARELVGGLLTQTPADVVSTVAAPMPMRAITSVLGVDGPDEAAFCRLS

NQAVRITDVALSASGLISLVQGFAGFRRLRALFTHRRDNGLLRECTVLGK

LATHAEQGRLSDDELFFFAVLLLVAGYESTAHMISTLFLTLADYPDQLTL

LAQQPDLIPSAIEEHLRFISPIQNICRTTRVDYSVGQAVIPAGSLVLLAW

GAANRDPRQYEDPDVFRADRNPVGHLAFGSGIHLCPGTQLARMEGQAILR

EIVANIDRIEVVEPPTWTTNANLRGLTRLRVAVTPRVAP

>CYP128A1 (2667026762) Mycobacterium tuberculosis MTBR1/09

MTATQSPPEPAPDRVRLAGCPLAGTPDVGLTAQDATTALGVPTRRRASSG

GIPVATSMWRDAQTVRTYGPAVAKALALRVAGKARSRLTGRHCRKFMQLT

DFDPFDPAIAADPYPHYRELLAGERVQYNPKRDVYILSRYADVREAARNH

DTLSSARGVTFSRGWLPFLPTSDPPAHTRMRKQLAPGMARGALETWRPMV

DQLARELVGGLLTQTPADVVSTVAAPMPMRAITSVLGVDGPDEAAFCRLS

NQAVRITDVALSASGLISLVQGFAGFRRLRALFTHRRDNGLLRECTVLGK

LATHAEQGRLSDDELFFFAVLLLVAGYESTAHMISTLFLTLADYPDQLTL

LAQQPDLIPSAIEEHLRFISPIQNICRTTRVDYSVGQAVIPAGSLVLLAW

GAANRDPRQYEDPDVFRADRNPVGHLAFGSGIHLCPGTQLARMEGQAILR

EIVANIDRIEVVEPPTWTTNANLRGLTRLRVAVTPRVAP

>CYP128A1 (2675218102) Mycobacterium tuberculosis C_10367

MTATQSPPEPAPDRVRLAGCPLAGTPDVGLTAQDATTALGVPTRRRASSG

GIPVATSMWRDAQTVRTYGPAVAKALALRVAGKARSRLTGRHCRKFMQLT

DFDPFDPAIAADPYPHYRELLAGERVQYNPKRDVYILSRYADVREAARNH

DTLSSARGVTFSRGWLPFLPTSDPPAHTRMRKQLAPGMARGALETWRPMV

DQLARELVGGLLTQTPADVVSTVAAPMPMRAITSVLGVDGPDEAAFCRLS

NQAVRITDVALSASGLISLVQGFAGFRRLRALFTHRRDNGLLRECTVLGK

LATHAEQGRLSDDELFFFAVLLLVAGYESTAHMISTLFLTLADYPDQLTL

LAQQPDLIPSAIEEHLRFISPIQNICRTTRVDYSVGQAVIPAGSLVLLAW

GAANRDPRQYEDPDVFRADRNPVGHLAFGSGIHLCPGTQLARMEGQAILR

EIVANIDRIEVVEPPTWTTNANLRGLTRLRVAVTPRVAP

>CYP128A1 (2699227728) Mycobacterium tuberculosis P09501164

MTATQSPPEPAPDRVRLAGCPLAGTPDVGLTAQDATTALGVPTRRRASSG

GIPVATSMWRDAQTVRTYGPAVAKALALRVAGKARSRLTGRHCRKFMQLT

DFDPFDPAIAADPYPHYRELLAGERVQYNPKRDVYILSRYADVREAARNH

DTLSSARGVTFSRGWLPFLPTSDPPAHTRMRKQLAPGMARGALETWRPMV

DQLARELVGGLLTQTPADVVSTVAAPMPMRAITSVLGVDGPDEAAFCRLS

NQAVRITDVALSASGLISLVQGFAGFRRLRALFTHRRDNGLLRECTVLGK

LATHAEQGRLSDDELFFFAVLLLVAGYESTAHMISTLFLTLADYPDQLTL

LAQQPDLIPSAIEEHLRFISPIQNICRTTRVDYSVGQAVIPAGSLVLLAW

GAANRDPRQYEDPDVFRADRNPVGHLAFGSGIHLCPGTQLARMEGQAILR

EIVANIDRIEVVEPPTWTTNANLRGLTRLRVAVTPRVAP

>CYP128A1 (2703458116) Mycobacterium tuberculosis D00700688

TAQDATTALGVPTRRRASSGGIPVATSMWRDAQTVRTYGPAVAKALALRV

AGKARSRLTGRHCRKFMQLTDFDPFDPAIAADPYPHYRELLAGERVQYNP

KRDVYILSRYADVREAARNHDTLSSARGVTFSRGWLPFLPTSDPPAHTRM

RKQLAPGMARGALETWRPMVDQLARELVGGLLTQTPADVVSTVAAPMPMR

AITSVLGVDGPDEAAFCRLSNQAVRITDVALSASGLISLVQGFAGFRRLR

ALFTHRRDNGLLRECTVLGKLATHAEQGRLSDDELFFFAVLLLVAGYEST

AHMISTLFLTLADYPDQLTLLAQQPDLIPSAIEEHLRFISPIQNICRTTR

VDYSVGQAVIPAGSLVLLAWGAANRDPRQYEDPDVFRADRNPVGHLAFGS

GIHLCPGTQLARMEGQAILREIVANIDRIEVVEPPTWTTNANLRGLTRLR

VAVTPRVAP

>CYP128A1 (2592231191) Mycobacterium tuberculosis TKK_04_0020

MTATQSPPEPAPDRVRLAGCPLAGTPDVGLTAQDATTALGVPTRRRASSG

GIPVATSMWRDAQTVRTYGPAVAKALALRVAGKARSRLTGRHCRKFMQLT

DFDPFDPAIAADPYPHYRELLAGERVQYNPKRDVYILSRYADVREAARNH

DTLSSSRGVTFSRGWLPFLPTSDPPAHTRMRKQLAPGMARGALETWRPMV

DQLARELVGGLLTQTPADVVSTVAAPMPMRAITSVLGVDGPDEAAFCRLS

NQAVRITDVALSASGLISLVQGFAGFRRLRALFTHRRDNGLLRECTVLGK

LATHAEQGRLSDDELFFFAVLLLVAGYESTAHMISTLFLTLADYPDQLTL

LAQQPDLIPSAIEEHLRFISPIQNICRTTRVDYSVGQAVIPAGSLVLLAW

GAANRDPRQYEDPDVFRADRNPVGHLAFGSGIHLCPGTQLARMEGQAILR

EIVANIDRIEVVEPPTWTTNANLRGLTRLRVAVTPRVAP

>CYP128B4 (2796965797) Mycobacteroides abscessus abscessus 982

MSRWRSDSAVRQIGAPVVAAVGMNIAAAVRVRRRGYAGWTGAVNTDYDPL

DPATAAQPFDAYRALHAGGRVHYNPKRATFILSRHEDIRAALRDTDAVTS

SQGVTRMKISAPILVLTDGDDHTRLRKQVQPGFTRGAMSDWQGMADQLAK

ELVADVVANPGCDVMERLAVPLPIRMIAHIIGIPPEDVQNFRSWSEDGVG

VINAGVSPAGLRQGLKGVRAIAALRRYFKDQLASGKLKGSDTVLGRLVDN

NEDGKLSDDELFFIAMLLLFAGNETTTNLIGGMFDTLAHAPDQFAMIRDD

PDLIPSAVEEQLRYSAPIQNLYRYTRTDYRVGEVTIPSGSRLLLAFGAAN

RDPEVFEDPDTYRADRNPRNHIAFGYGVHMCIGATLSRMEGQAVLRELTS

QASAIAAAGSATWSTNSSLRGTTYLPIRLTPAR

>CYP128A1 (2555342589) Mycobacterium tuberculosis PanR0605

MTATQSPPEPAPDRVRLAGCPLAGTPDVGLTAQDATTALGVPTRRRASSG

GIPVATSMWRDAQTVRTYGPAVAKALALRVAGKARSRLTGRHCRKFMQLT

DFDPFDPAIAADPYPHYRELLAGERVQYNPKRDVYILSRYADVREAARNH

DTLSSARGVTFSRGWLPFLPTSDPPAHTRMRKQLAPGMARGALETWRPMV

DQLARELVGGLLTQTPADVVSTVAAPMPMRAITSVLGVDGPDEAAFCRLS

NQAVRITDVALSASGLISLVQGFAGFRRLRALFTHRRDNGLLRECTVLGK

LATHAEQGRLSDDELFFFAVLLLVAGYESTAHMISTLFLTLADYPDQLTL

LAQQPDLIPSAIEEHLRFISPIQNICRTTRVDYSVGQAVIPAGSLVLLAW

GAANRDPRQYEDPDVFRADRNPVGHLAFGSGIHLCPGTQLARMEGQAILR

EIVANIDRIEVVEPPTWTTNANLRGLTRLRVAVTPRVAP

>CYP128A1 (2576400336) Mycobacterium tuberculosis TB_RSA195

MTATQSPPEPAPDRVRLAGCPLAGTPDVGLTAQDATTALGVPTRRRASSG

GIPVATSMWRDAQTVRTYGPAVAKALALRVAGKARSRLTGRHCRKFMQLT

DFDPFDPAIAADPYPHYRELLAGERVQYNPKRDVYILSRYADVREAARNH

DTLSSARGVTFSRGWLPFLPTSDPPAHTRMRKQLAPGMARGALETWRPMV

DQLARELVGGLLTQTPADVVSTVAAPMPMRAITSVLGVDGPDEAAFCRLS

NQAVRITDVALSASGLISLVQGFAGFRRLRALFTHRRDNGLLRECTVLGK

LATHAEQGRLSDDELFFFAVLLLVAGYESTAHMISTLFLTLADYPDQLTL

LAQQPDLIPSAIEEHLRFISPIQNICRTTRVDYSVGQAVIPAGSLVLLAW

GAANRDPRQYEDPDVFRADRNPVGHLAFGSGIHLCPGTQLARMEGQAILR

EIVANIDRIEVVEPPTWTTNANLRGLTRLRVAVTPRVAP

>CYP128A1 (2576928888) Mycobacterium tuberculosis M1913

MTATQSPPEPAPDRVRLAGCPLAGTPDVGLTAQDATTALGVPTRRRASSG

GIPVATSMWRDAQTVRTYGPAVAKALALRVAGKARSRLTGRHCRKFMQLT

DFDPFDPAIAADPYPHYRELLAGERVQYNPKRDVYILSRYADVREAARNH

DTLSSARGVTFSRGWLPFLPTSDPPAHTRMRKQLAPGMARGALETWRPMV

DQLARELVGGLLTQTPADVVSTVAAPMPMRAITSVLGVDGPDEAAFCRLS

NQAVRITDVALSASGLISLVQGFAGFRRLRALFTHRRDNGLLRECTVLGK

LATHAEQGRLSDDELFFFAVLLLVAGYESTAHMISTLFLTLADYPDQLTL

LAQQPDLIPSAIEEHLRFISPIQNICRTTRVDYSVGQAVIPAGSLVLLAW

GAANRDPRQYEDPDVFRADRNPVGHLAFGSGIHLCPGTQLARMEGQAILR

EIVANIDRIEVVEPPTWTTNANLRGLTRLRVAVTPRVAP

>CYP128A1 (2580939780) Mycobacterium africanum MAL010079

MTATQSPPEPAPDRVRLAGCPLAGTPDVGLTAQDATTALGVPTRRRASSG

GIPVATSMWRDAQTVRTYGPAVAKALALRVAGKARSRLTGRHCRKFMQLT

DFDPFDPAIAADPYPHYRELLAGERVQYNPKRDVYILSRYADVREAARNH

DTLSSARGVTFSRGWLPFLPTSDPPAHTRMRKQLAPGMARGALETWRPMV

DQLARELVGGLLTQTPADVVSTVAAPMPMRAITSVLGVDGPDEAAFCRLS

NQAVRITDVALSASGLISLVQGFAGFRRLRALFTHRRDNGLLRECTVLGK

LATHAEQGRLSDDELFFFAVLLLVAGYESTAHMISTLFLTLADYPDQLTL

LAQQPDLIPSAIEEHLRFISPIQNICRTTRVDYSVGQAVIPAGSLVLLAW

GAANRDPRQYEDPDVFRADRNPVGHLAFGSGIHLCPGTQLARMEGQAILR

EIVANIDRIEVVEPPTWTTNANLRGLTRLRVAVTPRVAP

>CYP128A1 (2581511485) Mycobacterium africanum MAL010084

MTATQSPPEPAPDRVRLAGCPLAGTPDVGLTAQDATTALGVPTRRRASSG

GIPVATSMWRDAQTVRTYGPAVAKALALRVAGKARSRLTGRHCRKFMQLT

DFDPFDPAIAADPYPHYRELLAGERVQYNPKRDVYILSRYADVREAARNH

DTLSSARGVTFSRGWLPFLPTSDPPAHTRMRKQLAPGMARGALETWRPMV

DQLARELVGGLLTQTPADVVSTVAAPMPMRAITSVLGVDGPDEAAFCRLS

NQAVRITDVALSASGLISLVQGFAGFRRLRALFTHRRDNGLLRECTVLGK

LATHAEQGRLSDDELFFFAVLLLVAGYESTAHMISTLFLTLADYPDQLTL

LAQQPDLIPSAIEEHLRFISPIQNICRTTRVDYSVGQAVIPAGSLVLLAW

GAANRDPRQYEDPDVFRADRNPVGHLAFGSGIHLCPGTQLARMEGQAILR

EIVANIDRIEVVEPPTWTTNANLRGLTRLRVAVTPRVAP

>CYP128A1 (2589547871) Mycobacterium tuberculosis TKK-01-0027

MTATQSPPEPAPDRVRLAGCPLAGTPDVGLTAQDATTALGVPTRRRASSG

GIPVATSMWRDAQTVRTYGPAVAKALALRVAGKARSRLTGRHCRKFMQLT

DFDPFDPAIAADPYPHYRELLAGERVQYNPKRDVYILSRYADVREAARNH

DTLSSARGVTFSRGWLPFLPTSDPPAHTRMRKQLAPGMARGALETWRPMV

DQLARELVGGLLTQTPADVVSTVAAPMPMRAITSVLGVDGPDEAAFCRLS

NQAVRITDVALSASGLISLVQGFAGFRRLRALFTHRRDNGLLRECTVLGK

LATHAEQGRLSDDELFFFAVLLLVAGYESTAHMISTLFLTLADYPDQLTL

LAQQPDLIPSAIEEHLRFISPIQNICRTTRVDYSVGQAVIPAGSLVLLAW

GAANRDPRQYEDPDVFRADRNPVGHLAFGSGIHLCPGTQLARMEGQAILR

EIVANIDRIEVVEPPTWTTNANLRGLTRLRVAVTPRVAP

>CYP128A1 (2590195897) Mycobacterium tuberculosis MAL020209

MTATQSPPEPAPDRVRLAGCPLAGTPDVGLTAQDATTALGVPTRRRASSG

GIPVATSMWRDAQTVRTYGPAVAKALALRVAGKARSRLTGRHCRKFMQLT

DFDPFDPAIAADPYPHYRELLAGERVQYNPKRDVYILSRYADVREAARNH

DTLSSARGVTFSRGWLPFLPTSDPPAHTRMRKQLAPGMARGALETWRPMV

DQLARELVGGLLTQTPADVVSTVAAPMPMRAITSVLGVDGPDEAAFCRLS

NQAVRITDVALSASGLISLVQGFAGFRRLRALFTHRRDNGLLRECTVLGK

LATHAEQGRLSDDELFFFAVLLLVAGYESTAHMISTLFLTLADYPDQLTL

LAQQPDLIPSAIEEHLRFISPIQNICRTTRVDYSVGQAVIPAGSLVLLAW

GAANRDPRQYEDPDVFRADRNPVGHLAFGSGIHLCPGTQLARMEGQAILR

EIVANIDRIEVVEPPTWTTNANLRGLTRLRVAVTPRVAP

>CYP128A1 (2603955909) Mycobacterium tuberculosis TB_RSA128

MTATQSPPEPAPDRVRLAGCPLAGTPDVGLTAQDATTALGVPTRRRASSG

GIPVATSMWRDAQTVRTYGPAVAKALALRVAGKARSRLTGRHCRKFMQLT

DFDPFDPAIAADPYPHYRELLAGERVQYNPKRDVYILSRYADVREAARNH

DTLSSARGVTFSRGWLPFLPTSDPPAHTRMRKQLAPGMARGALETWRPMV

DQLARELVGGLLTQTPADVVSTVAAPMPMRAITSVLGVDGPDEAAFCRLS

NQAVRITDVALSASGLISLVQGFAGFRRLRALFTHRRDNGLLRECTVLGK

LATHAEQGRLSDDELFFFAVLLLVAGYESTAHMISTLFLTLADYPDQLTL

LAQQPDLIPSAIEEHLRFISPIQNICRTTRVDYSVGQAVIPAGSLVLLAW

GAANRDPRQYEDPDVFRADRNPVGHLAFGSGIHLCPGTQLARMEGQAILR

EIVANIDRIEVVEPPTWTTNANLRGLTRLRVAVTPRVAP

>CYP128A1 (2621403388) Mycobacterium tuberculosis XTB13-108

MTATQSPPEPAPDRVRLAGCPLAGTPDVGLTAQDATTALGVPTRRRASSG

GIPVATSMWRDAQTVRTYGPAVAKALALRVAGKARSRLTGRHCRKFMQLT

DFDPFDPAIAADPYPHYRELLAGERVQYNPKRDVYILSRYADVREAARNH

DTLSSARGVTFSRGWLPFLPTSDPPAHTRMRKQLAPGMARGALETWRPMV

DQLARELVGGLLTQTPADVVSTVAAPMPMRAITSVLGVDGPDEAAFCRLS

NQAVRITDVALSASGLISLVQGFAGFRRLRALFTHRRDNGLLRECTVLGK

LATHAEQGRLSDDELFFFAVLLLVAGYESTAHMISTLFLTLADYPDQLTL

LAQQPDLIPSAIEEHLRFISPIQNICRTTRVDYSVGQAVIPAGSLVLLAW

GAANRDPRQYEDPDVFRADRNPVGHLAFGSGIHLCPGTQLARMEGQAILR

EIVANIDRIEVVEPPTWTTNANLRGLTRLRVAVTPRVAP

>CYP128A1 (2621795762) Mycobacterium tuberculosis TKK_04_0137

MTATQSPPEPAPDRVRLAGCPLAGTPDVGLTAQDATTALGVPTRRRASSG

GIPVATSMWRDAQTVRTYGPAVAKALALRVAGKARSRLTGRHCRKFMQLT

DFDPFDPAIAADPYPHYRELLAGERVQYNPKRDVYILSRYADVREAARNH

DTLSSARGVTFSRGWLPFLPTSDPPAHTRMRKQLAPGMARGALETWRPMV

DQLARELVGGLLTQTPADVVSTVAAPMPMRAITSVLGVDGPDEAAFCRLS

NQAVRITDVALSASGLISLVQGFAGFRRLRALFTHRRDNGLLRECTVLGK

LATHAEQGRLSDDELFFFAVLLLVAGYESTAHMISTLFLTLADYPDQLTL

LAQQPDLIPSAIEEHLRFISPIQNICRTTRVDYSVGQAVIPAGSLVLLAW

GAANRDPRQYEDPDVFRADRNPVGHLAFGSGIHLCPGTQLARMEGQAILR

EIVANIDRIEVVEPPTWTTNANLRGLTRLRVAVTPRVAP

>CYP128A1 (2640537524) Mycobacterium tuberculosis KT-0201

MTATQSPPEPAPDRVRLAGCPLAGTPDVGLTAQDATTALGVPTRRRASSG

GIPVATSMWRDAQTVRTYGPAVAKALALRVAGKARSRLTGRHCRKFMQLT

DFDPFDPAIAADPYPHYRELLAGERVQYNPKRDVYILSRYADVREAARNH

DTLSSARGVTFSRGWLPFLPTSDPPAHTRMRKQLAPGMARGALETWRPMV

DQLARELVGGLLTQTPADVVSTVAAPMPMRAITSVLGVDGPDEAAFCRLS

NQAVRITDVALSASGLISLVQGFAGFRRLRALFTHRRDNGLLRECTVLGK

LATHAEQGRLSDDELFFFAVLLLVAGYESTAHMISTLFLTLADYPDQLTL

LAQQPDLIPSAIEEHLRFISPIQNICRTTRVDYSVGQAVIPAGSLVLLAW

GAANRDPRQYEDPDVFRADRNPVGHLAFGSGIHLCPGTQLARMEGQAILR

EIVANIDRIEVVEPPTWTTNANLRGLTRLRVAVTPRVAP

>CYP128A1 (2670882083) Mycobacterium tuberculosis 400260

MTATQSPPEPAPDRVRLAGCPLAGTPDVGLTAQDATTALGVPTRRRASSG

GIPVATSMWRDAQTVRTYGPAVAKALALRVAGKARSRLTGRHCRKFMQLT

DFDPFDPAIAADPYPHYRELLAGERVQYNPKRDVYILSRYADVREAARNH

DTLSSARGVTFSRGWLPFLPTSDPPAHTRMRKQLAPGMARGALETWRPMV

DQLARELVGGLLTQTPADVVSTVAAPMPMRAITSVLGVDGPDEAAFCRLS

NQAVRITDVALSASGLISLVQGFAGFRRLRALFTHRRDNGLLRECTVLGK

LATHAEQGRLSDDELFFFAVLLLVAGYESTAHMISTLFLTLADYPDQLTL

LAQQPDLIPSAIEEHLRFISPIQNICRTTRVDYSVGQAVIPAGSLVLLAW

GAANRDPRQYEDPDVFRADRNPVGHLAFGSGIHLCPGTQLARMEGQAILR

EIVANIDRIEVVEPPTWTTNANLRGLTRLRVAVTPRVAP

>CYP128A1 (2701656984) Mycobacterium tuberculosis 402251

MTATQSPPEPAPDRVRLAGCPLAGTPDVGLTAQDATTALGVPTRRRASSG

GIPVATSMWRDAQTVRTYGPAVAKALALRVAGKARSRLTGRHCRKFMQLT

DFDPFDPAIAADPYPHYRELLAGERVQYNPKRDVYILSRYADVREAARNH

DTLSSARGVTFSRGWLPFLPTSDPPAHTRMRKQLAPGMARGALETWRPMV

DQLARELVGGLLTQTPADVVSTVAAPMPMRAITSVLGVDGPDEAAFCRLS

NQAVRITDVALSASGLISLVQGFAGFRRLRALFTHRRDNGLLRECTVLGK

LATHAEQGRLSDDELFFFAVLLLVAGYESTAHMISTLFLTLADYPDQLTL

LAQQPDLIPSAIEEHLRFISPIQNICRTTRVDYSVGQAVIPAGSLVLLAW

GAANRDPRQYEDPDVFRADRNPVGHLAFGSGIHLCPGTQLARMEGQAILR

EIVANIDRIEVVEPPTWTTNANLRGLTRLRVAVTPRVAP

>CYP128B4 (2809994855) Mycobacterium sp. E787

MTLTDGVRFTGSAARTLAGAVLANAAGARRRAPIDAAITAYDPLYAATAA

QPHSAYRRLHAGPRVQYNPKRRVFVLSRLDDVRAAARDDANLSSAEGPML

TRIRTPILVSLDGEKHSRQRRQVLPAFTRAALDSWRPIIDRLAAETVRDV

LANPGCDVVQRLAIPMPVRLIAQLLGVPDTDVDDFRRWSEASVQITDLDL

SVRGARKLAGSLRGSWAMHRYFTGQFAVGGLKGSDTILGRLLAENAAGSL

PDRELFYFAMLLLLAGNETTTNLLGGMFDTLAAHPEQYELLRADPDSVPM

AVEELLRYLSPAQNVYRTALNDYRVGDATIPARSRIMLSIGAANRDPLAF

DEPDAFRVHRNPTQHMTFGFGAHLCIGAQLTRMEAQAVLRELVTRVDRIS

VVGAPRWSTNSLLRGPTRLAVRLTPA

>CYP128B4 (2675134246) Mycobacterium abscessus PAP161

MSRWRSDSAVRQIGAPVVAAVGMNIAAAVRVRRRGYAGWTGAVNTDYDPL

DPATAAQPFDAYRALHAGGRVHYNPKRATFILSRHEDIRAALRDTDAVTS

SQGVTRMKISAPILVLTDGDDHTRLRKQVQPGFTRGAMSDWQGMADQLAK

ELVADVVANPGCDVMERLAVPLPIRMIAHIIGIPPEDVQNFRSWSEDGVG

VINAGVSPAGLRQGLKGVRAIAALRRYFKDQLASGKLKGSDTVLGRLVDN

NEDGKLSDDELFFIAMLLLFAGNETTTNLIGGMFDTLAHAPDQFAMIRDD

PDLIPSAVEEQLRYSAPIQNLYRYTRTDYRVGEVTIPSGSRLLLAFGAAN

RDPEVFEDPDTYRADRNPRNHIAFGYGVHMCIGATLSRMEGQAVLRELTS

QASAIAAAGSATWSTNSSLRGTTYLPIRLTPAR

>CYP128B2 (2508747744) Mycobacterium tusciae JS617

MVVSSKTGSGGTLLKPLASAVQMNLVGALRTRWRGCDGWTGAINTDYDPL

NPVTAAQPHEAYRALHRQGRVHYNPKRATWILNQLNDVRAALRNTDQVTS

ALGVTRVRMAADLVVLTDGDRHAELRKQVQPGFTRGALEGWQEINDNLAH

ELVSDLIANPGCDAVQRLAIPMPMRLIASILGVPESDVPDFRRWSEKSVR

VMDFSPTPRSLVETAASMRAVIALRRYFLRHLAAGELKDSQTVLGQLLRH

NTDGNLDDDQLFYIALLLLIAGNETTTNLLGGMLDTLARNPDQYDMIRAN

PDLIPMAVEEQLRFSSPIQNLYRYTRAAYQVGNVTIPAGARLLLSFGAAN

RDPLIFEDPDTFRADRDPRMHIGFGHGAHMCVGAPLARMEAQAVLRELVT

RVTRITAGAATWSTNSSLRGPTRLPVWLHSG

>CYP128B4 (2774727798) Mycobacterium aurum liquid

VTTPRRAEARGAFAGALSSALRLNTAATVRTWRRGHAGWTGAINTDYDPL

DRAVAAQPFDAFRALHRSGGVHYNPKRATWILCRLADVRAALRDTDAVTS

TEGVTRVKIAAPVLVLTDGAEHARLRKQVQPAFSKGAMADWQEMIDRLAA

ELVADLLDDPGCDVVRRLTIPMPMRVIAHILGVPDADVDQFRAWSEAGMQ

IVDFEPTGRGVVRSAKALASLAALRRYFMKQFSRGGLKDSTTVLGRLLTH

NDEGELTEDQLFLIAVLLLIAGNETTTNLLGGMFDTLAHHPDQFDMIRAD

PSLIPSAVEEQLRFSSPIQNLYRYTRADYRVGEVTIPAGSRLMLNFAAAN

RDPLVFDDPDTYRADRNPRSHIAFGHGAHMCIGAPLARMEAQAVLRELVT

QVSAIAPAAPTVWSTNTSLRGPVRLPIALTGA

>CYP128B1 (2809989877) Mycobacterium sp. E787

VSISQTAHKAAQTLSPVARAVRMNVAAAIRTRRRGYDGWTGAINTDYDPQ

NPATAADPFDAYRALHRSGRVHYNPRRATFILSRLDDVRAALRDTDQVTS

AQGVTRLRISGPLAVVTDGEEHARLRKQVQPGFSKGAMKSWQEMTEKLAT

DLVTDVLNDPGCDVVQRLAIPMPISLIAQILGIPDTDIGDFRRWSERAVG

VMDLTPTLSSLVDAAKTMSAMLALQRYFTGQFATGGLKGSNTVLGRLIEH

NTDGSLTDSQLLLIAIHLLIAGNETTTNLLGGMFDTLARRPDQYDLIRAN

PDLIPLAVEEQLRITTPIQNLYRYTRADYPIGDVTIPTGSRVLLSFGAAN

RDPTAFDDPDEYRADRDPRTHVAFGYGAHMCLGAPLARMEAQAVLRQLVT

RVARIIPEGATQWSTHSSLRGPTHLPIRLTPA

>CYP128A1 (2555520830) Mycobacterium tuberculosis PanR0411

MTATQSPPEPAPDRVRLAGCPLAGTPDVGLTAQDATTALGVPTRRRASSG

GIPVATSMWRDAQTVRTYGPAVAKALALRVAGKARSRLTGRHCRKFMQLT

DFDPFDPAIAADPYPHYRELLAGERVQYNPKRDVYILSRYADVREAARNH

DTLSSARGVTFSRGWLPFLPTSDPPAHTRMRKQLAPGMARGALETWRPMV

DQLARELVGGLLTQTPADVVSTVAAPMPMRAITSVLGVDGPDEAAFCRLS

NQAVRITDVALSASGLISLVQGFAGFRRLRALFTHRRDNGLLRECTVLGK

LATHAEQGRLSDDELFFFAVLLLVAGYESTAHMISTLFLTLADYPDQLTL

LAQQPDLIPSAIEEHLRFISPIQNICRTTRVDYSVGQAVIPAGSLVLLAW

GAANRDPRQYEDPDVFRADRNPVGHLAFGSGIHLCPGTQLARMEGQAILR

EIVANIDRIEVVEPPTWTTNANLRGLTRLRVAVTPRVAP

>CYP128A1 (2575187510) Mycobacterium tuberculosis MD19964

MTATQSPPEPAPDRVRLAGCPLAGTPDVGLTAQDATTALGVPTRRRASSG

GIPVATSMWRDAQTVRTYGPAVAKALALRVAGKARSRLTGRHCRKFMQLT

DFDPFDPAIAADPYPHYRELLAGERVQYNPKRDVYILSRYADVREAARNH

DTLSSARGVTFSRGWLPFLPTSDPPAHTRMRKQLAPGMARGALETWRPMV

DQLARELVGGLLTQTPADVVSTVAAPMPMRAITSVLGVDGPDEAAFCRLS

NQAVRITDVALSASGLISLVQGFAGFRRLRALFTHRRDNGLLRECTVLGK

LATHAEQGRLSDDELFFFAVLLLVAGYESTAHMISTLFLTLADYPDQLTL

LAQQPDLIPSAIEEHLRFISPIQNICRTTRVDYSVGQAVIPAGSLVLLAW

GAANRDPRQYEDPDVFRADRNPVGHLAFGSGIHLCPGTQLARMEGQAILR

EIVANIDRIEVVEPPTWTTNANLRGLTRLRVAVTPRVAP

>CYP128A1 (2576252224) Mycobacterium tuberculosis KT-0110

MTATQSPPEPAPDRVRLAGCPLAGTPDVGLTAQDATTALGVPTRRRASSG

GIPVATSMWRDAQTVRTYGPAVAKALALRVAGKARSRLTGRHCRKFMQLT

DFDPFDPAIAADPYPHYRELLAGERVQYNPKRDVYILSRYADVREAARNH

DTLSSARGVTFSRGWLPFLPTSDPPAHTRMRKQLAPGMARGALETWRPMV

DQLARELVGGLLTQTPADVVSTVAAPMPMRAITSVLGVDGPDEAAFCRLS

NQAVRITDVALSASGLISLVQGFAGFRRLRALFTHRRDNGLLRECTVLGK

LATHAEQGRLSDDELFFFAVLLLVAGYESTAHMISTLFLTLADYPDQLTL

LAQQPDLIPSAIEEHLRFISPIQNICRTTRVDYSVGQAVIPAGSLVLLAW

GAANRDPRQYEDPDVFRADRNPVGHLAFGSGIHLCPGTQLARMEGQAILR

EIVANIDRIEVVEPPTWTTNANLRGLTRLRVAVTPRVAP

>CYP128A1 (2577642534) Mycobacterium tuberculosis TB_RSA70

MTATQSPPEPAPDRVRLAGCPLAGTPDVGLTAQDATTALGVPTRRRASSG

GIPVATSMWRDAQTVRTYGPAVAKALALRVAGKARSRLTGRHCRKFMQLT

DFDPFDPAIAADPYPHYRELLAGERVQYNPKRDVYILSRYADVREAARNH

DTLSSARGVTFSRGWLPFLPTSDPPAHTRMRKQLAPGMARGALETWRPMV

DQLARELVGGLLTQTPADVVSTVAAPMPMRAITSVLGVDGPDEAAFCRLS

NQAVRITDVALSASGLISLVQGFAGFRRLRALFTHRRDNGLLRECTVLGK

LATHAEQGRLSDDELFFFAVLLLVAGYESTAHMISTLFLTLADYPDQLTL

LAQQPDLIPSAIEEHLRFISPIQNICRTTRVDYSVGQAVIPAGSLVLLAW

GAANRDPRQYEDPDVFRADRNPVGHLAFGSGIHLCPGTQLARMEGQAILR

EIVANIDRIEVVEPPTWTTNANLRGLTRLRVAVTPRVAP

>CYP128A1 (2589103511) Mycobacterium tuberculosis TBR56

MTATQSPPEPAPDRVRLAGCPLAGTPDVGLTAQDATTALGVPTRRRASSG

GIPVATSMWRDAQTVRTYGPAVAKALALRVAGKARSRLTGRHCRKFMQLT

DFDPFDPAIAADPYPHYRELLAGERVQYNPKRDVYILSRYADVREAARNH

DTLSSARGVTFSRGWLPFLPTSDPPAHTRMRKQLAPGMARGALETWRPMV

DQLARELVGGLLTQTPADVVSTVAAPMPMRAITSVLGVDGPDEAAFCRLS

NQAVRITDVALSASGLISLVQGFAGFRRLRALFTHRRDNGLLRECTVLGK

LATHAEQGRLSDDELFFFAVLLLVAGYESTAHMISTLFLTLADYPDQLTL

LAQQPDLIPSAIEEHLRFISPIQNICRTTRVDYSVGQAVIPAGSLVLLAW

GAANRDPRQYEDPDVFRADRNPVGHLAFGSGIHLCPGTQLARMEGQAILR

EIVANIDRIEVVEPPTWTTNANLRGLTRLRVAVTPRVAP

>CYP128A1 (2604557417) Mycobacterium tuberculosis BTB07-300

MTATQSPPEPAPDRVRLAGCPLAGTPDVGLTAQDATTALGVPTRRRASSG

GIPVATSMWRDAQTVRTYGPAVAKALALRVAGKARSRLTGRHCRKFMQLT

DFDPFDPAIAADPYPHYRELLAGERVQYNPKRDVYILSRYADVREAARNH

DTLSSARGVTFSRGWLPFLPTSDPPAHTRMRKQLAPGMARGALETWRPMV

DQLARELVGGLLTQTPADVVSTVAAPMPMRAITSVLGVDGPDEAAFCRLS

NQAVRITDVALSASGLISLVQGFAGFRRLRALFTHRRDNGLLRECTVLGK

LATHAEQGRLSDDELFFFAVLLLVAGYESTAHMISTLFLTLADYPDQLTL

LAQQPDLIPSAIEEHLRFISPIQNICRTTRVDYSVGQAVIPAGSLVLLAW

GAANRDPRQYEDPDVFRADRNPVGHLAFGSGIHLCPGTQLARMEGQAILR

EIVANIDRIEVVEPPTWTTNANLRGLTRLRVAVTPRVAP

>CYP128A1 (2622386896) Mycobacterium tuberculosis M1232

MTATQSPPEPAPDRVRLAGCPLAGTPDVGLTAQDATTALGVPTRRRASSG

GIPVATSMWRDAQTVRTYGPAVAKALALRVAGKARSRLTGRHCRKFMQLT

DFDPFDPAIAADPYPHYRELLAGERVQYNPKRDVYILSRYADVREAARNH

DTLSSARGVTFSRGWLPFLPTSDPPAHTRMRKQLAPGMARGALETWRPMV

DQLARELVGGLLTQTPADVVSTVAAPMPMRAITSVLGVDGPDEAAFCRLS

NQAVRITDVALSASGLISLVQGFAGFRRLRALFTHRRDNGLLRECTVLGK

LATHAEQGRLSDDELFFFAVLLLVAGYESTAHMISTLFLTLADYPDQLTL

LAQQPDLIPSAIEEHLRFISPIQNICRTTRVDYSVGQAVIPAGSLVLLAW

GAANRDPRQYEDPDVFRADRNPVGHLAFGSGIHLCPGTQLARMEGQAILR

EIVANIDRIEVVEPPTWTTNANLRGLTRLRVAVTPRVAP

>CYP128A1 (2652672077) Mycobacterium tuberculosis TKK_04_0125

MTATQSPPEPAPDRVRLAGCPLAGTPDVGLTAQDATTALGVPTRRRASSG

GIPVATSMWRDAQTVRTYGPAVAKALALRVAGKARSRLTGRHCRKFMQLT

DFDPFDPAIAADPYPHYRELLAGERVQYNPKRDVYILSRYADVREAARNH

DTLSSARGVTFSRGWLPFLPTSDPPAHTRMRKQLAPGMARGALETWRPMV

DQLARELVGGLLTQTPADVVSTVAAPMPMRAITSVLGVDGPDEAAFCRLS

NQAVRITDVALSASGLISLVQGFAGFRRLRALFTHRRDNGLLRECTVLGK

LATHAEQGRLSDDELFFFAVLLLVAGYESTAHMISTLFLTLADYPDQLTL

LAQQPDLIPSAIEEHLRFISPIQNICRTTRVDYSVGQAVIPAGSLVLLAW

GAANRDPRQYEDPDVFRADRNPVGHLAFGSGIHLCPGTQLARMEGQAILR

EIVANIDRIEVVEPPTWTTNANLRGLTRLRVAVTPRVAP

>CYP128A1 (2701647631) Mycobacterium tuberculosis 302203

MTATQSPPEPAPDRVRLAGCPLAGTPDVGLTAQDATTALGVPTRRRASSG

GIPVATSMWRDAQTVRTYGPAVAKALALRVAGKARSRLTGRHCRKFMQLT

DFDPFDPAIAADPYPHYRELLAGERVQYNPKRDVYILSRYADVREAARNH

DTLSSARGVTFSRGWLPFLPTSDPPAHTRMRKQLAPGMARGALETWRPMV

DQLARELVGGLLTQTPADVVSTVAAPMPMRAITSVLGVDGPDEAAFCRLS

NQAVRITDVALSASGLISLVQGFAGFRRLRALFTHRRDNGLLRECTVLGK

LATHAEQGRLSDDELFFFAVLLLVAGYESTAHMISTLFLTLADYPDQLTL

LAQQPDLIPSAIEEHLRFISPIQNICRTTRVDYSVGQAVIPAGSLVLLAW

GAANRDPRQYEDPDVFRADRNPVGHLAFGSGIHLCPGTQLARMEGQAILR

EIVANIDRIEVVEPPTWTTNANLRGLTRLRVAVTPRVAP

>CYP128A1 (637096678) Mycobacterium tuberculosis CDC1551

MTATQSPPEPAPDRVRLAGCPLAGTPDVGLTAQDATTALGVPTRRRASSG

GIPVATSMWRDAQTVRTYGPAVAKALALRVAGKARSRLTGRHCRKFMQLT

DFDPFDPAIAADPYPHYRELLAGERVQYNPKRDVYILSRYADVREAARNH

DTLSSARGVTFSRGWLPFLPTSDPPAHTRMRKQLAPGMARGALETWRPMV

DQLARELVGGLLTQTPADVVSTVAAPMPMRAITSVLGVDGPDEAAFCRLS

NQAVRITDVALSASGLISLVQGFAGFRRLRALFTHRRDNGLLRECTVLGK

LATHAEQGRLSDDELFFFAVLLLVAGYESTAHMISTLFLTLADYPDQLTL

LAQQPDLIPSAIEEHLRFISPIQNICRTTRVDYSVGQAVIPAGSLVLLAW

GAANRDPRQYEDPDVFRADRNPVGHLAFGSGIHLCPGTQLARMEGQAILR

EIVANIDRIEVVEPPTWTTNANLRGLTRLRVAVTPRVAP

>CYP128A1 (2606338201) Mycobacterium tuberculosis XTB13-179

MTATQSPPEPAPDRVRLAGCPLAGTPDVGLTAQDATTALGVPTRRRAASG

GIPVATSMWRDAQTVRTYGPAVAKALALRVAGKARSRLTGRHCRKFMQLT

DFDPFDPAIAADPYPHYRELLAGERVQYNPKRDVYILSRYADVREAARNH

DTLSSARGVTFSRGWLPFLPTSDPPAHTRMRKQLAPGMARGALETWRPMV

DQLARELVGGLLTQTPADVVSTVAAPMPMRAITSVLGVDGPDEAAFCRLS

NQAVRITDVALSASGLISLVQGFAGFRRLRALFTHRRDNGLLRECTVLGK

LATHAEQGRLSDDELFFFAVLLLVAGYESTAHMISTLFLTLADYPDQLTL

LAQQPDLIPSAIEEHLRFISPIQNICRTTRVDYSVGQAVIPAGSLVLLAW

GAANRDPRQYEDPDVFRADRNPVGHLAFGSGIHLCPGTQLARMEGQAILR

EIVANIDRIEVVEPPTWTTNANLRGLTRLRVAVTPRVAP

>CYP128A1 (2719114279) Mycobacterium tuberculosis 0A117DS

MTATQSPPEPAPDRVRLAGCPLAGTPDVGLTAQDATTALGVPTRRRASSG

GIPVATSMWRDAQTVRTYGPAVAKALALRVAGKARSRLTGRHCRKFMQLT

DFDPFDPAIAADPYPHYRELLAGERVQYNPKRDVYILSRYADVREAARNH

DTLSSARGVTFSRGWLPFLPTSDPPAHTRMRKQLAPGMARGALETWRPMV

DQLARELVGGLLTQTPADVVSTVAAPMPMRAITSVLGVDGPDEAAFCRLS

NQAVRITDVALSASGLISLVQGFAGFRRLRALFTHRRDNGLLRECTVLGK

LATHAEQGRLSDDELFFFAVLLLVAGYESTAHMISTLFLTLADYPDQLTL

LAQQPDLIPSAIEEHLRFISPIQNICRTTRVDYSVGQAVIPAGSLVLLAW

GAANRDPRQYEDPDVFRADRNPVGHLAFGSGIHLCPGTQLARMEGQAILR

EIVANIDRIEVGEPPTWTTNANLRGLTRLRVAVTPRVAP

>CYP128B4 (2798359073) Mycobacteroides abscessus abscessus 680

MSRWRSDSAVRQIGAPVVAAVGMNIAAAVRVRRRGYAGWTGAVNTDYDPL

DPATAAQPFDAYRALHAGGRVHYNPKRATFILSRHEDIRAALRDTDAVTS

SQGVTRMKISAPILVLTDGDDHTRLRKQVQPGFTRGAMSDWQGMADQLAK

ELVADVVANPGCDVMERLAVPLPIRMIAHIIGIPPEDVQNFRSWSEDGVG

VINAGVSPAGLRQGLKGVRAIAALRRYFKDQLASGKLKGSDTVLGRLVDN

NEDGKLSDDELFFIAMLLLFAGNETTTNLIGGMFDTLAHAPDQFAMIRDD

PDLIPSAVEEQLRYSAPIQNLYRYTRTDYRVGEVTIPSGSRLLLAFGAAN

RDPEVFEDPDTYRADRNPRNHIAFGYGVHMCIGATLSRMEGQAVLRELTS

QASAIAAAGSATWSTNSSLRGTTYLPIRLTPA

>CYP128B4 (2804411252) Mycobacteroides abscessus abscessus 431

MSRWRSDSAVRQIGAPVVAAVGMNIAAAVRVRRRGYAGWTGAVNTDYDPL

DPATAAQPFDAYRALHAGGRVHYNPKRATFILSRHEDIRAALRDTDAVTS

SQGVTRMKISAPILVLTDGDDHTRLRKQVQPGFTRGAMSDWQGMADQLAK

ELVADVVANPGCDVMERLAVPLPIRMIAHIIGIPPEDVQNFRSWSEDGVG

VINAGVSPAGLRQGLKGVRAIAALRRYFKDQLASGKLKGSDTVLGRLVDN

NEDGKLSDDELFFIAMLLLFAGNETTTNLIGGMFDTLAHAPDQFAMIRDD

PDLIPSAVEEQLRYSAPIQNLYRYTRTDYRVGEVTIPSGSRLLLAFGAAN

RDPEVFEDPDTYRADRNPRNHIAFGYGVHMCIGATLSRMEGQAVLRELTS

QASAIAAAGSATWSTNSSLRGTTYLPIRLTPAR

>CYP128A1 (2554690947) Mycobacterium tuberculosis EAI/OSDD271

MTATQSPPEPAPDRVRLAGCPLAGTPDVGLTAQDATTALGVPTRRRASSG

GIPVATSMWRDAQTVRTYGPAVAKALALRVAGKARSRLTGRHCRKFMQLT

DFDPFDPAIAADPYPHYRELLAGERVQYNPKRDVYILSRYADVREAARNH

DTLSSARGVTFSRGWLPFLPTSDPPAHTRMRKQLAPGMARGALETWRPMV

DQLARELVGGLLTQTPADVVSTVAAPMPMRAITSVLGVDGPDEAAFCRLS

NQAVRITDVALSASGLISLVQGFAGFRRLRALFTHRRDNGLLRECTVLGK

LATHAEQGRLSDDELFFFAVLLLVAGYESTAHMISTLFLTLADYPDQLTL

LAQQPDLIPSAIEEHLRFISPIQNICRTTRVDYSVGQAVIPAGSLVLLAW

GAANRDPRQYEDPDVFRADRNPVGHLAFGSGIHLCPGTQLARMEGQAILR

EIVANIDRIEVVEPPTWTTNANLRGLTRLRVAVTPRVAP

>CYP128A1 (2577633836) Mycobacterium tuberculosis MD17517

MTATQSPPEPAPDRVRLAGCPLAGTPDVGLTAQDATTALGVPTRRRASSG

GIPVATSMWRDAQTVRTYGPAVAKALALRVAGKARSRLTGRHCRKFMQLT

DFDPFDPAIAADPYPHYRELLAGERVQYNPKRDVYILSRYADVREAARNH

DTLSSARGVTFSRGWLPFLPTSDPPAHTRMRKQLAPGMARGALETWRPMV

DQLARELVGGLLTQTPADVVSTVAAPMPMRAITSVLGVDGPDEAAFCRLS

NQAVRITDVALSASGLISLVQGFAGFRRLRALFTHRRDNGLLRECTVLGK

LATHAEQGRLSDDELFFFAVLLLVAGYESTAHMISTLFLTLADYPDQLTL

LAQQPDLIPSAIEEHLRFISPIQNICRTTRVDYSVGQAVIPAGSLVLLAW

GAANRDPRQYEDPDVFRADRNPVGHLAFGSGIHLCPGTQLARMEGQAILR

EIVANIDRIEVVEPPTWTTNANLRGLTRLRVAVTPRVAP

>CYP128A1 (2584803896) Mycobacterium tuberculosis TB_RSA104

MTATQSPPEPAPDRVRLAGCPLAGTPDVGLTAQDATTALGVPTRRRASSG

GIPVATSMWRDAQTVRTYGPAVAKALALRVAGKARSRLTGRHCRKFMQLT

DFDPFDPAIAADPYPHYRELLAGERVQYNPKRDVYILSRYADVREAARNH

DTLSSARGVTFSRGWLPFLPTSDPPAHTRMRKQLAPGMARGALETWRPMV

DQLARELVGGLLTQTPADVVSTVAAPMPMRAITSVLGVDGPDEAAFCRLS

NQAVRITDVALSASGLISLVQGFAGFRRLRALFTHRRDNGLLRECTVLGK

LATHAEQGRLSDDELFFFAVLLLVAGYESTAHMISTLFLTLADYPDQLTL

LAQQPDLIPSAIEEHLRFISPIQNICRTTRVDYSVGQAVIPAGSLVLLAW

GAANRDPRQYEDPDVFRADRNPVGHLAFGSGIHLCPGTQLARMEGQAILR

EIVANIDRIEVVEPPTWTTNANLRGLTRLRVAVTPRVAP

>CYP128A1 (2584850265) Mycobacterium tuberculosis TRUG0083

MTATQSPPEPAPDRVRLAGCPLAGTPDVGLTAQDATTALGVPTRRRASSG

GIPVATSMWRDAQTVRTYGPAVAKALALRVAGKARSRLTGRHCRKFMQLT

DFDPFDPAIAADPYPHYRELLAGERVQYNPKRDVYILSRYADVREAARNH

DTLSSARGVTFSRGWLPFLPTSDPPAHTRMRKQLAPGMARGALETWRPMV

DQLARELVGGLLTQTPADVVSTVAAPMPMRAITSVLGVDGPDEAAFCRLS

NQAVRITDVALSASGLISLVQGFAGFRRLRALFTHRRDNGLLRECTVLGK

LATHAEQGRLSDDELFFFAVLLLVAGYESTAHMISTLFLTLADYPDQLTL

LAQQPDLIPSAIEEHLRFISPIQNICRTTRVDYSVGQAVIPAGSLVLLAW

GAANRDPRQYEDPDVFRADRNPVGHLAFGSGIHLCPGTQLARMEGQAILR

EIVANIDRIEVVEPPTWTTNANLRGLTRLRVAVTPRVAP

>CYP128A1 (2590118525) Mycobacterium tuberculosis MAL020157

MTATQSPPEPAPDRVRLAGCPLAGTPDVGLTAQDATTALGVPTRRRASSG

GIPVATSMWRDAQTVRTYGPAVAKALALRVAGKARSRLTGRHCRKFMQLT

DFDPFDPAIAADPYPHYRELLAGERVQYNPKRDVYILSRYADVREAARNH

DTLSSARGVTFSRGWLPFLPTSDPPAHTRMRKQLAPGMARGALETWRPMV

DQLARELVGGLLTQTPADVVSTVAAPMPMRAITSVLGVDGPDEAAFCRLS

NQAVRITDVALSASGLISLVQGFAGFRRLRALFTHRRDNGLLRECTVLGK

LATHAEQGRLSDDELFFFAVLLLVAGYESTAHMISTLFLTLADYPDQLTL

LAQQPDLIPSAIEEHLRFISPIQNICRTTRVDYSVGQAVIPAGSLVLLAW

GAANRDPRQYEDPDVFRADRNPVGHLAFGSGIHLCPGTQLARMEGQAILR

EIVANIDRIEVVEPPTWTTNANLRGLTRLRVAVTPRVAP

>CYP128A1 (2603168850) Mycobacterium tuberculosis BTB10-258

MTATQSPPEPAPDRVRLAGCPLAGTPDVGLTAQDATTALGVPTRRRASSG

GIPVATSMWRDAQTVRTYGPAVAKALALRVAGKARSRLTGRHCRKFMQLT

DFDPFDPAIAADPYPHYRELLAGERVQYNPKRDVYILSRYADVREAARNH

DTLSSARGVTFSRGWLPFLPTSDPPAHTRMRKQLAPGMARGALETWRPMV

DQLARELVGGLLTQTPADVVSTVAAPMPMRAITSVLGVDGPDEAAFCRLS

NQAVRITDVALSASGLISLVQGFAGFRRLRALFTHRRDNGLLRECTVLGK

LATHAEQGRLSDDELFFFAVLLLVAGYESTAHMISTLFLTLADYPDQLTL

LAQQPDLIPSAIEEHLRFISPIQNICRTTRVDYSVGQAVIPAGSLVLLAW

GAANRDPRQYEDPDVFRADRNPVGHLAFGSGIHLCPGTQLARMEGQAILR

EIVANIDRIEVVEPPTWTTNANLRGLTRLRVAVTPRVAP

>CYP128A1 (2603469377) Mycobacterium tuberculosis TB_RSA141

MTATQSPPEPAPDRVRLAGCPLAGTPDVGLTAQDATTALGVPTRRRASSG

GIPVATSMWRDAQTVRTYGPAVAKALALRVAGKARSRLTGRHCRKFMQLT

DFDPFDPAIAADPYPHYRELLAGERVQYNPKRDVYILSRYADVREAARNH

DTLSSARGVTFSRGWLPFLPTSDPPAHTRMRKQLAPGMARGALETWRPMV

DQLARELVGGLLTQTPADVVSTVAAPMPMRAITSVLGVDGPDEAAFCRLS

NQAVRITDVALSASGLISLVQGFAGFRRLRALFTHRRDNGLLRECTVLGK

LATHAEQGRLSDDELFFFAVLLLVAGYESTAHMISTLFLTLADYPDQLTL

LAQQPDLIPSAIEEHLRFISPIQNICRTTRVDYSVGQAVIPAGSLVLLAW

GAANRDPRQYEDPDVFRADRNPVGHLAFGSGIHLCPGTQLARMEGQAILR

EIVANIDRIEVVEPPTWTTNANLRGLTRLRVAVTPRVAP

>CYP128A1 (2604001307) Mycobacterium tuberculosis TB_RSA36

MTATQSPPEPAPDRVRLAGCPLAGTPDVGLTAQDATTALGVPTRRRASSG

GIPVATSMWRDAQTVRTYGPAVAKALALRVAGKARSRLTGRHCRKFMQLT

DFDPFDPAIAADPYPHYRELLAGERVQYNPKRDVYILSRYADVREAARNH

DTLSSARGVTFSRGWLPFLPTSDPPAHTRMRKQLAPGMARGALETWRPMV

DQLARELVGGLLTQTPADVVSTVAAPMPMRAITSVLGVDGPDEAAFCRLS

NQAVRITDVALSASGLISLVQGFAGFRRLRALFTHRRDNGLLRECTVLGK

LATHAEQGRLSDDELFFFAVLLLVAGYESTAHMISTLFLTLADYPDQLTL

LAQQPDLIPSAIEEHLRFISPIQNICRTTRVDYSVGQAVIPAGSLVLLAW

GAANRDPRQYEDPDVFRADRNPVGHLAFGSGIHLCPGTQLARMEGQAILR

EIVANIDRIEVVEPPTWTTNANLRGLTRLRVAVTPRVAP

>CYP128A1 (2629196090) Mycobacterium tuberculosis TKK_04_0083

MTATQSPPEPAPDRVRLAGCPLAGTPDVGLTAQDATTALGVPTRRRASSG

GIPVATSMWRDAQTVRTYGPAVAKALALRVAGKARSRLTGRHCRKFMQLT

DFDPFDPAIAADPYPHYRELLAGERVQYNPKRDVYILSRYADVREAARNH

DTLSSARGVTFSRGWLPFLPTSDPPAHTRMRKQLAPGMARGALETWRPMV

DQLARELVGGLLTQTPADVVSTVAAPMPMRAITSVLGVDGPDEAAFCRLS

NQAVRITDVALSASGLISLVQGFAGFRRLRALFTHRRDNGLLRECTVLGK

LATHAEQGRLSDDELFFFAVLLLVAGYESTAHMISTLFLTLADYPDQLTL

LAQQPDLIPSAIEEHLRFISPIQNICRTTRVDYSVGQAVIPAGSLVLLAW

GAANRDPRQYEDPDVFRADRNPVGHLAFGSGIHLCPGTQLARMEGQAILR

EIVANIDRIEVVEPPTWTTNANLRGLTRLRVAVTPRVAP

>CYP128A1 (2639251383) Mycobacterium tuberculosis M2384

MTATQSPPEPAPDRVRLAGCPLAGTPDVGLTAQDATTALGVPTRRRASSG

GIPVATSMWRDAQTVRTYGPAVAKALALRVAGKARSRLTGRHCRKFMQLT

DFDPFDPAIAADPYPHYRELLAGERVQYNPKRDVYILSRYADVREAARNH

DTLSSARGVTFSRGWLPFLPTSDPPAHTRMRKQLAPGMARGALETWRPMV

DQLARELVGGLLTQTPADVVSTVAAPMPMRAITSVLGVDGPDEAAFCRLS

NQAVRITDVALSASGLISLVQGFAGFRRLRALFTHRRDNGLLRECTVLGK

LATHAEQGRLSDDELFFFAVLLLVAGYESTAHMISTLFLTLADYPDQLTL

LAQQPDLIPSAIEEHLRFISPIQNICRTTRVDYSVGQAVIPAGSLVLLAW

GAANRDPRQYEDPDVFRADRNPVGHLAFGSGIHLCPGTQLARMEGQAILR

EIVANIDRIEVVEPPTWTTNANLRGLTRLRVAVTPRVAP

>CYP128A1 (2653109957) Mycobacterium tuberculosis KT-0005

MTATQSPPEPAPDRVRLAGCPLAGTPDVGLTAQDATTALGVPTRRRASSG

GIPVATSMWRDAQTVRTYGPAVAKALALRVAGKARSRLTGRHCRKFMQLT

DFDPFDPAIAADPYPHYRELLAGERVQYNPKRDVYILSRYADVREAARNH

DTLSSARGVTFSRGWLPFLPTSDPPAHTRMRKQLAPGMARGALETWRPMV

DQLARELVGGLLTQTPADVVSTVAAPMPMRAITSVLGVDGPDEAAFCRLS

NQAVRITDVALSASGLISLVQGFAGFRRLRALFTHRRDNGLLRECTVLGK

LATHAEQGRLSDDELFFFAVLLLVAGYESTAHMISTLFLTLADYPDQLTL

LAQQPDLIPSAIEEHLRFISPIQNICRTTRVDYSVGQAVIPAGSLVLLAW

GAANRDPRQYEDPDVFRADRNPVGHLAFGSGIHLCPGTQLARMEGQAILR

EIVANIDRIEVVEPPTWTTNANLRGLTRLRVAVTPRVAP

>CYP128A1 (2719881473) Mycobacterium tuberculosis TB282

MTATQSPPEPAPDRVRLAGCPLAGTPDVGLTAQDATTALGVPTRRRASSG

GIPVATSMWRDAQTVRTYGPAVAKALALRVAGKARSRLTGRHCRKFMQLT

DFDPFDPAIAADPYPHYRELLAGERVQYNPKRDVYILSRYADVREAARNH

DTLSSARGVTFSRGWLPFLPTSDPPAHTRMRKQLAPGMARGALETWRPMV

DQLARELVGGLLTQTPADVVSTVAAPMPMRAITSVLGVDGPDEAAFCRLS

NQAVRITDVALSASGLISLVQGFAGFRRLRALFTHRRDNGLLRECTVLGK

LATHAEQGRLSDDELFFFAVLLLVAGYESTAHMISTLFLTLADYPDQLTL

LAQQPDLIPSAIEEHLRFISPIQNICRTTRVDYSVGQAVIPAGSLVLLAW

GAANRDPRQYEDPDVFRADRNPVGHLAFGSGIHLCPGTQLARMEGQAILR

EIVANIDRIEVVEPPTWTTNANLRGLTRLRVAVTPRVAP

>CYP128A1 (2576315950) Mycobacterium tuberculosis XTB13-143

MTATQSPPEPAPDRVRLAGCPLAGTPDVGLTAQDATTALGVPTRRRAASG

GIPVATSMWRDAQTVRTYGPAVAKALALRVAGKARSRLTGRHCRKFMQLT

DFDPFDPAIAADPYPHYRELLAGERVQYNPKRDVYILSRYADVREAARNH

DTLSSARGVTFSRGWLPFLPTSDPPAHTRMRKQLAPGMARGALETWRPMV

DQLARELVGGLLTQTPADVVSTVAAPMPMRAITSVLGVDGPDEAAFCRLS

NQAVRITDVALSASGLISLVQGFAGFRRLRALFTHRRDNGLLRECTVLGK

LATHAEQGRLSDDELFFFAVLLLVAGYESTAHMISTLFLTLADYPDQLTL

LAQQPDLIPSAIEEHLRFISPIQNICRTTRVDYSVGQAVIPAGSLVLLAW

GAANRDPRQYEDPDVFRADRNPVGHLAFGSGIHLCPGTQLARMEGQAILR

EIVANIDRIEVVEPPTWTTNANLRGLTRLRVAVTPRVAP

>CYP128A1 (2621626759) Mycobacterium tuberculosis XTB13-235

MTATQSPPEPAPDRVRLAGCPLAGTPDVGLTAQDATTALGVPTRRRAASG

GIPVATSMWRDAQTVRTYGPAVAKALALRVAGKARSRLTGRHCRKFMQLT

DFDPFDPAIAADPYPHYRELLAGERVQYNPKRDVYILSRYADVREAARNH

DTLSSARGVTFSRGWLPFLPTSDPPAHTRMRKQLAPGMARGALETWRPMV

DQLARELVGGLLTQTPADVVSTVAAPMPMRAITSVLGVDGPDEAAFCRLS

NQAVRITDVALSASGLISLVQGFAGFRRLRALFTHRRDNGLLRECTVLGK

LATHAEQGRLSDDELFFFAVLLLVAGYESTAHMISTLFLTLADYPDQLTL

LAQQPDLIPSAIEEHLRFISPIQNICRTTRVDYSVGQAVIPAGSLVLLAW

GAANRDPRQYEDPDVFRADRNPVGHLAFGSGIHLCPGTQLARMEGQAILR

EIVANIDRIEVVEPPTWTTNANLRGLTRLRVAVTPRVAP

>CYP128A1 (2549408356) Mycobacterium tuberculosis OSDD504

MTATQSPPEPAPDRVRLAGCPLAGTPDVGLTAQDATTALGVPTRRRASSG

GIPVATSMWRDAQTVRTYGPAVAKALALRVAGKARSRLTGRHCRKFMQLT

DFDPFDPAIAADPYPHYRELLAGERVQYNPKRDVYILSRYADVREAARNH

DTLSSARGVTFSRGWLPFLPTSDPPAHTRMRKQLAPGMARGALETWRPMV

DQLARELVGGLLTQTPADVVSTVAAPMPMRAITSVLGVDGPDEAAFCRLS

NQAVRITDVALSASGLISLVQGFAGFRRLRALFTHRRDNGLLRECTVLGK

LATHAEQGRLSDDELFFFAVLLLVAGYESTAHMISTLFLTLADYPDQLTL

LAQQPDLIPSAIEEHLRFISPIQNICRTTRVDYSVGQAVIPAGSLVLLAW

GAANRDPRQYEDPDVFRADRNPVGHLAFGSGIHLCPGTQLARMEGQAILR

EIVANIDRIEVVEPPTWTTNANLRGLTRLRVAVTPRVAP

>CYP128A1 (2555600470) Mycobacterium tuberculosis PanR0401

MTATQSPPEPAPDRVRLAGCPLAGTPDVGLTAQDATTALGVPTRRRASSG

GIPVATSMWRDAQTVRTYGPAVAKALALRVAGKARSRLTGRHCRKFMQLT

DFDPFDPAIAADPYPHYRELLAGERVQYNPKRDVYILSRYADVREAARNH

DTLSSARGVTFSRGWLPFLPTSDPPAHTRMRKQLAPGMARGALETWRPMV

DQLARELVGGLLTQTPADVVSTVAAPMPMRAITSVLGVDGPDEAAFCRLS

NQAVRITDVALSASGLISLVQGFAGFRRLRALFTHRRDNGLLRECTVLGK

LATHAEQGRLSDDELFFFAVLLLVAGYESTAHMISTLFLTLADYPDQLTL

LAQQPDLIPSAIEEHLRFISPIQNICRTTRVDYSVGQAVIPAGSLVLLAW

GAANRDPRQYEDPDVFRADRNPVGHLAFGSGIHLCPGTQLARMEGQAILR

EIVANIDRIEVVEPPTWTTNANLRGLTRLRVAVTPRVAP

>CYP128A1 (2575871532) Mycobacterium tuberculosis KT-0034

MTATQSPPEPAPDRVRLAGCPLAGTPDVGLTAQDATTALGVPTRRRASSG

GIPVATSMWRDAQTVRTYGPAVAKALALRVAGKARSRLTGRHCRKFMQLT

DFDPFDPAIAADPYPHYRELLAGERVQYNPKRDVYILSRYADVREAARNH

DTLSSARGVTFSRGWLPFLPTSDPPAHTRMRKQLAPGMARGALETWRPMV

DQLARELVGGLLTQTPADVVSTVAAPMPMRAITSVLGVDGPDEAAFCRLS

NQAVRITDVALSASGLISLVQGFAGFRRLRALFTHRRDNGLLRECTVLGK

LATHAEQGRLSDDELFFFAVLLLVAGYESTAHMISTLFLTLADYPDQLTL

LAQQPDLIPSAIEEHLRFISPIQNICRTTRVDYSVGQAVIPAGSLVLLAW

GAANRDPRQYEDPDVFRADRNPVGHLAFGSGIHLCPGTQLARMEGQAILR

EIVANIDRIEVVEPPTWTTNANLRGLTRLRVAVTPRVAP

>CYP128A1 (2576932899) Mycobacterium tuberculosis TKK_03_0158

MTATQSPPEPAPDRVRLAGCPLAGTPDVGLTAQDATTALGVPTRRRASSG

GIPVATSMWRDAQTVRTYGPAVAKALALRVAGKARSRLTGRHCRKFMQLT

DFDPFDPAIAADPYPHYRELLAGERVQYNPKRDVYILSRYADVREAARNH

DTLSSARGVTFSRGWLPFLPTSDPPAHTRMRKQLAPGMARGALETWRPMV

DQLARELVGGLLTQTPADVVSTVAAPMPMRAITSVLGVDGPDEAAFCRLS

NQAVRITDVALSASGLISLVQGFAGFRRLRALFTHRRDNGLLRECTVLGK

LATHAEQGRLSDDELFFFAVLLLVAGYESTAHMISTLFLTLADYPDQLTL

LAQQPDLIPSAIEEHLRFISPIQNICRTTRVDYSVGQAVIPAGSLVLLAW

GAANRDPRQYEDPDVFRADRNPVGHLAFGSGIHLCPGTQLARMEGQAILR

EIVANIDRIEVVEPPTWTTNANLRGLTRLRVAVTPRVAP

>CYP128A1 (2579809103) Mycobacterium tuberculosis M995

MTATQSPPEPAPDRVRLAGCPLAGTPDVGLTAQDATTALGVPTRRRASSG

GIPVATSMWRDAQTVRTYGPAVAKALALRVAGKARSRLTGRHCRKFMQLT

DFDPFDPAIAADPYPHYRELLAGERVQYNPKRDVYILSRYADVREAARNH

DTLSSARGVTFSRGWLPFLPTSDPPAHTRMRKQLAPGMARGALETWRPMV

DQLARELVGGLLTQTPADVVSTVAAPMPMRAITSVLGVDGPDEAAFCRLS

NQAVRITDVALSASGLISLVQGFAGFRRLRALFTHRRDNGLLRECTVLGK

LATHAEQGRLSDDELFFFAVLLLVAGYESTAHMISTLFLTLADYPDQLTL

LAQQPDLIPSAIEEHLRFISPIQNICRTTRVDYSVGQAVIPAGSLVLLAW

GAANRDPRQYEDPDVFRADRNPVGHLAFGSGIHLCPGTQLARMEGQAILR

EIVANIDRIEVVEPPTWTTNANLRGLTRLRVAVTPRVAP

>CYP128A1 (2584609698) Mycobacterium tuberculosis TBR51

MTATQSPPEPAPDRVRLAGCPLAGTPDVGLTAQDATTALGVPTRRRASSG

GIPVATSMWRDAQTVRTYGPAVAKALALRVAGKARSRLTGRHCRKFMQLT

DFDPFDPAIAADPYPHYRELLAGERVQYNPKRDVYILSRYADVREAARNH

DTLSSARGVTFSRGWLPFLPTSDPPAHTRMRKQLAPGMARGALETWRPMV

DQLARELVGGLLTQTPADVVSTVAAPMPMRAITSVLGVDGPDEAAFCRLS

NQAVRITDVALSASGLISLVQGFAGFRRLRALFTHRRDNGLLRECTVLGK

LATHAEQGRLSDDELFFFAVLLLVAGYESTAHMISTLFLTLADYPDQLTL

LAQQPDLIPSAIEEHLRFISPIQNICRTTRVDYSVGQAVIPAGSLVLLAW

GAANRDPRQYEDPDVFRADRNPVGHLAFGSGIHLCPGTQLARMEGQAILR

EIVANIDRIEVVEPPTWTTNANLRGLTRLRVAVTPRVAP

>CYP128A1 (2589704117) Mycobacterium tuberculosis TKK-01-0088

MTATQSPPEPAPDRVRLAGCPLAGTPDVGLTAQDATTALGVPTRRRASSG

GIPVATSMWRDAQTVRTYGPAVAKALALRVAGKARSRLTGRHCRKFMQLT

DFDPFDPAIAADPYPHYRELLAGERVQYNPKRDVYILSRYADVREAARNH

DTLSSARGVTFSRGWLPFLPTSDPPAHTRMRKQLAPGMARGALETWRPMV

DQLARELVGGLLTQTPADVVSTVAAPMPMRAITSVLGVDGPDEAAFCRLS

NQAVRITDVALSASGLISLVQGFAGFRRLRALFTHRRDNGLLRECTVLGK

LATHAEQGRLSDDELFFFAVLLLVAGYESTAHMISTLFLTLADYPDQLTL

LAQQPDLIPSAIEEHLRFISPIQNICRTTRVDYSVGQAVIPAGSLVLLAW

GAANRDPRQYEDPDVFRADRNPVGHLAFGSGIHLCPGTQLARMEGQAILR

EIVANIDRIEVVEPPTWTTNANLRGLTRLRVAVTPRVAP

>CYP128A1 (2590049339) Mycobacterium tuberculosis MAL010124

MTATQSPPEPAPDRVRLAGCPLAGTPDVGLTAQDATTALGVPTRRRASSG

GIPVATSMWRDAQTVRTYGPAVAKALALRVAGKARSRLTGRHCRKFMQLT

DFDPFDPAIAADPYPHYRELLAGERVQYNPKRDVYILSRYADVREAARNH

DTLSSARGVTFSRGWLPFLPTSDPPAHTRMRKQLAPGMARGALETWRPMV

DQLARELVGGLLTQTPADVVSTVAAPMPMRAITSVLGVDGPDEAAFCRLS

NQAVRITDVALSASGLISLVQGFAGFRRLRALFTHRRDNGLLRECTVLGK

LATHAEQGRLSDDELFFFAVLLLVAGYESTAHMISTLFLTLADYPDQLTL

LAQQPDLIPSAIEEHLRFISPIQNICRTTRVDYSVGQAVIPAGSLVLLAW

GAANRDPRQYEDPDVFRADRNPVGHLAFGSGIHLCPGTQLARMEGQAILR

EIVANIDRIEVVEPPTWTTNANLRGLTRLRVAVTPRVAP

>CYP128A1 (2603917308) Mycobacterium tuberculosis TRUG0071

MTATQSPPEPAPDRVRLAGCPLAGTPDVGLTAQDATTALGVPTRRRASSG

GIPVATSMWRDAQTVRTYGPAVAKALALRVAGKARSRLTGRHCRKFMQLT

DFDPFDPAIAADPYPHYRELLAGERVQYNPKRDVYILSRYADVREAARNH

DTLSSARGVTFSRGWLPFLPTSDPPAHTRMRKQLAPGMARGALETWRPMV

DQLARELVGGLLTQTPADVVSTVAAPMPMRAITSVLGVDGPDEAAFCRLS

NQAVRITDVALSASGLISLVQGFAGFRRLRALFTHRRDNGLLRECTVLGK

LATHAEQGRLSDDELFFFAVLLLVAGYESTAHMISTLFLTLADYPDQLTL

LAQQPDLIPSAIEEHLRFISPIQNICRTTRVDYSVGQAVIPAGSLVLLAW

GAANRDPRQYEDPDVFRADRNPVGHLAFGSGIHLCPGTQLARMEGQAILR

EIVANIDRIEVVEPPTWTTNANLRGLTRLRVAVTPRVAP

>CYP128A1 (2622290155) Mycobacterium tuberculosis H2300

MTATQSPPEPAPDRVRLAGCPLAGTPDVGLTAQDATTALGVPTRRRASSG

GIPVATSMWRDAQTVRTYGPAVAKALALRVAGKARSRLTGRHCRKFMQLT

DFDPFDPAIAADPYPHYRELLAGERVQYNPKRDVYILSRYADVREAARNH

DTLSSARGVTFSRGWLPFLPTSDPPAHTRMRKQLAPGMARGALETWRPMV

DQLARELVGGLLTQTPADVVSTVAAPMPMRAITSVLGVDGPDEAAFCRLS

NQAVRITDVALSASGLISLVQGFAGFRRLRALFTHRRDNGLLRECTVLGK

LATHAEQGRLSDDELFFFAVLLLVAGYESTAHMISTLFLTLADYPDQLTL

LAQQPDLIPSAIEEHLRFISPIQNICRTTRVDYSVGQAVIPAGSLVLLAW

GAANRDPRQYEDPDVFRADRNPVGHLAFGSGIHLCPGTQLARMEGQAILR

EIVANIDRIEVVEPPTWTTNANLRGLTRLRVAVTPRVAP

>CYP128A1 (2622689719) Mycobacterium tuberculosis M1955

MTATQSPPEPAPDRVRLAGCPLAGTPDVGLTAQDATTALGVPTRRRASSG

GIPVATSMWRDAQTVRTYGPAVAKALALRVAGKARSRLTGRHCRKFMQLT

DFDPFDPAIAADPYPHYRELLAGERVQYNPKRDVYILSRYADVREAARNH

DTLSSARGVTFSRGWLPFLPTSDPPAHTRMRKQLAPGMARGALETWRPMV

DQLARELVGGLLTQTPADVVSTVAAPMPMRAITSVLGVDGPDEAAFCRLS

NQAVRITDVALSASGLISLVQGFAGFRRLRALFTHRRDNGLLRECTVLGK

LATHAEQGRLSDDELFFFAVLLLVAGYESTAHMISTLFLTLADYPDQLTL

LAQQPDLIPSAIEEHLRFISPIQNICRTTRVDYSVGQAVIPAGSLVLLAW

GAANRDPRQYEDPDVFRADRNPVGHLAFGSGIHLCPGTQLARMEGQAILR

EIVANIDRIEVVEPPTWTTNANLRGLTRLRVAVTPRVAP

>CYP128A1 (2622715264) Mycobacterium tuberculosis M2003

MTATQSPPEPAPDRVRLAGCPLAGTPDVGLTAQDATTALGVPTRRRASSG

GIPVATSMWRDAQTVRTYGPAVAKALALRVAGKARSRLTGRHCRKFMQLT

DFDPFDPAIAADPYPHYRELLAGERVQYNPKRDVYILSRYADVREAARNH

DTLSSARGVTFSRGWLPFLPTSDPPAHTRMRKQLAPGMARGALETWRPMV

DQLARELVGGLLTQTPADVVSTVAAPMPMRAITSVLGVDGPDEAAFCRLS

NQAVRITDVALSASGLISLVQGFAGFRRLRALFTHRRDNGLLRECTVLGK

LATHAEQGRLSDDELFFFAVLLLVAGYESTAHMISTLFLTLADYPDQLTL

LAQQPDLIPSAIEEHLRFISPIQNICRTTRVDYSVGQAVIPAGSLVLLAW

GAANRDPRQYEDPDVFRADRNPVGHLAFGSGIHLCPGTQLARMEGQAILR

EIVANIDRIEVVEPPTWTTNANLRGLTRLRVAVTPRVAP

>CYP128A1 (2622809238) Mycobacterium tuberculosis M2021

MTATQSPPEPAPDRVRLAGCPLAGTPDVGLTAQDATTALGVPTRRRASSG

GIPVATSMWRDAQTVRTYGPAVAKALALRVAGKARSRLTGRHCRKFMQLT

DFDPFDPAIAADPYPHYRELLAGERVQYNPKRDVYILSRYADVREAARNH

DTLSSARGVTFSRGWLPFLPTSDPPAHTRMRKQLAPGMARGALETWRPMV

DQLARELVGGLLTQTPADVVSTVAAPMPMRAITSVLGVDGPDEAAFCRLS

NQAVRITDVALSASGLISLVQGFAGFRRLRALFTHRRDNGLLRECTVLGK

LATHAEQGRLSDDELFFFAVLLLVAGYESTAHMISTLFLTLADYPDQLTL

LAQQPDLIPSAIEEHLRFISPIQNICRTTRVDYSVGQAVIPAGSLVLLAW

GAANRDPRQYEDPDVFRADRNPVGHLAFGSGIHLCPGTQLARMEGQAILR

EIVANIDRIEVVEPPTWTTNANLRGLTRLRVAVTPRVAP

>CYP128A1 (2699415131) Mycobacterium tuberculosis K09600911

MTATQSPPEPAPDRVRLAGCPLAGTPDVGLTAQDATTALGVPTRRRASSG

GIPVATSMWRDAQTVRTYGPAVAKALALRVAGKARSRLTGRHCRKFMQLT

DFDPFDPAIAADPYPHYRELLAGERVQYNPKRDVYILSRYADVREAARNH

DTLSSARGVTFSRGWLPFLPTSDPPAHTRMRKQLAPGMARGALETWRPMV

DQLARELVGGLLTQTPADVVSTVAAPMPMRAITSVLGVDGPDEAAFCRLS

NQAVRITDVALSASGLISLVQGFAGFRRLRALFTHRRDNGLLRECTVLGK

LATHAEQGRLSDDELFFFAVLLLVAGYESTAHMISTLFLTLADYPDQLTL

LAQQPDLIPSAIEEHLRFISPIQNICRTTRVDYSVGQAVIPAGSLVLLAW

GAANRDPRQYEDPDVFRADRNPVGHLAFGSGIHLCPGTQLARMEGQAILR

EIVANIDRIEVVEPPTWTTNANLRGLTRLRVAVTPRVAP

>CYP128B4 (2803364748) Mycobacteroides abscessus abscessus 521

MSRWRSDSAVRQIGAPVVAAVGMNIAAAVRVRRRGYAGWTGAVNTDYDPL

DPATAAQPFDAYRALHAGGRVHYNPKRATFILSRHEDIRAALRDTDAVTS

SQGVTRMKISAPILVLTDGDDHTRLRKQVQPGFTRGAMSDWQGMADQLAK

ELVADVVANPGCDVMERLAVPLPIRMIAHIIGIPPEDVQNFRSWSEDGVG

VINAGVSPAGLRQGLKGVRAIAALRRYFKDQLASGKLKGSDTVLGRLVDN

NEDGKLSDDELFFIAMLLLFAGNETTTNLIGGMFDTLAHAPDQFAMIRDD

PDLIPSAVEEQLRYSAPIQNLYRYTRTDYRVGEVTIPSGSRLLLAFGAAN

RDPEVFEDPDTYRADRNPRNHIAFGYGVHMCIGATLSRMEGQAVLRELTS

QASAIAAAGSATWSTNSSLRGTTYLPIRLTPAR

>CYP128A1 (2540620657) Mycobacterium tuberculosis Erdman, ATCC 35801

MQLTDFDPFDPAIAADPYPHYRELLAGERVQYNPKRDVYILSRYADVREA

ARNHDTLSSARGVTFSRGWLPFLPTSDPPAHTRMRKQLAPGMARGALETW

RPMVDQLARELVGGLLTQTPADVVSTVAAPMPMRAITSVLGVDGPDEAAF

CRLSNQAVRITDVALSASGLISLVQGFAGFRRLRALFTHRRDNGLLRECT

VLGKLATHAEQGRLSDDELFFFAVLLLVAGYESTAHMISTLFLTLADYPD

QLTLLAQQPDLIPSAIEEHLRFISPIQNICRTTRVDYSVGQAVIPAGSLV

LLAWGAANRDPRQYEDPDVFRADRNPVGHLAFGSGIHLCPGPSWRAWRVR

RSCARSSPISTE

>CYP128A1 (2574859569) Mycobacterium tuberculosis TKK_02_0071

MTATQSPPEPAPDRVRLAGCPLAGTPDVGLTAQDATTALGVPTRRRASSG

GIPVATSMWRDAQTVRTYGPAVAKALALRVAGKARSRLTGRHCRKFMQLT

DFDPFDPAIAADPYPHYRELLAGERVQYNPKRDVYILSRYADVREAARNH

DTLSSARGVTFSRGWLPFLPTSDPPAHTRMRKQLAPGMARGALETWRPMV

DQLARELVGGLLTQTPADVVSTVAAPMPMRAITSVLGVDGPDEAAFCRLS

NQAVRITDVALSASGLISLVQGFAGFRRLRALFTHRRDNGLLRECTVLGK

LATHAEQGRLSDDELFFFAVLLLVAGYESTAHMISTLFLTLADYPDQLTL

LAQQPDLIPSAIEEHLRFISPIQNICRTTRVDYSVGQAVIPAGSLVLLAW

GAANRDPRQYEDPDVFRADRNPVGHLAFGSGIHLCPGTQLARMEGQAILR

EIVANIDRIEVVEPPTWTTNANLRGLTRLRVAVTPRVAP

>CYP128A1 (2577656011) Mycobacterium tuberculosis KT-0070

MTATQSPPEPAPDRVRLAGCPLAGTPDVGLTAQDATTALGVPTRRRASSG

GIPVATSMWRDAQTVRTYGPAVAKALALRVAGKARSRLTGRHCRKFMQLT

DFDPFDPAIAADPYPHYRELLAGERVQYNPKRDVYILSRYADVREAARNH

DTLSSARGVTFSRGWLPFLPTSDPPAHTRMRKQLAPGMARGALETWRPMV

DQLARELVGGLLTQTPADVVSTVAAPMPMRAITSVLGVDGPDEAAFCRLS

NQAVRITDVALSASGLISLVQGFAGFRRLRALFTHRRDNGLLRECTVLGK

LATHAEQGRLSDDELFFFAVLLLVAGYESTAHMISTLFLTLADYPDQLTL

LAQQPDLIPSAIEEHLRFISPIQNICRTTRVDYSVGQAVIPAGSLVLLAW

GAANRDPRQYEDPDVFRADRNPVGHLAFGSGIHLCPGTQLARMEGQAILR

EIVANIDRIEVVEPPTWTTNANLRGLTRLRVAVTPRVAP

>CYP128A1 (2584853577) Mycobacterium tuberculosis TRUG0124

MTATQSPPEPAPDRVRLAGCPLAGTPDVGLTAQDATTALGVPTRRRASSG

GIPVATSMWRDAQTVRTYGPAVAKALALRVAGKARSRLTGRHCRKFMQLT

DFDPFDPAIAADPYPHYRELLAGERVQYNPKRDVYILSRYADVREAARNH

DTLSSARGVTFSRGWLPFLPTSDPPAHTRMRKQLAPGMARGALETWRPMV

DQLARELVGGLLTQTPADVVSTVAAPMPMRAITSVLGVDGPDEAAFCRLS

NQAVRITDVALSASGLISLVQGFAGFRRLRALFTHRRDNGLLRECTVLGK

LATHAEQGRLSDDELFFFAVLLLVAGYESTAHMISTLFLTLADYPDQLTL

LAQQPDLIPSAIEEHLRFISPIQNICRTTRVDYSVGQAVIPAGSLVLLAW

GAANRDPRQYEDPDVFRADRNPVGHLAFGSGIHLCPGTQLARMEGQAILR

EIVANIDRIEVVEPPTWTTNANLRGLTRLRVAVTPRVAP

>CYP128A1 (2590167390) Mycobacterium tuberculosis MAL020199

MTATQSPPEPAPDRVRLAGCPLAGTPDVGLTAQDATTALGVPTRRRASSG

GIPVATSMWRDAQTVRTYGPAVAKALALRVAGKARSRLTGRHCRKFMQLT

DFDPFDPAIAADPYPHYRELLAGERVQYNPKRDVYILSRYADVREAARNH

DTLSSARGVTFSRGWLPFLPTSDPPAHTRMRKQLAPGMARGALETWRPMV

DQLARELVGGLLTQTPADVVSTVAAPMPMRAITSVLGVDGPDEAAFCRLS

NQAVRITDVALSASGLISLVQGFAGFRRLRALFTHRRDNGLLRECTVLGK

LATHAEQGRLSDDELFFFAVLLLVAGYESTAHMISTLFLTLADYPDQLTL

LAQQPDLIPSAIEEHLRFISPIQNICRTTRVDYSVGQAVIPAGSLVLLAW

GAANRDPRQYEDPDVFRADRNPVGHLAFGSGIHLCPGTQLARMEGQAILR

EIVANIDRIEVVEPPTWTTNANLRGLTRLRVAVTPRVAP

>CYP128A1 (2590358501) Mycobacterium tuberculosis OFXR-29

MTATQSPPEPAPDRVRLAGCPLAGTPDVGLTAQDATTALGVPTRRRASSG

GIPVATSMWRDAQTVRTYGPAVAKALALRVAGKARSRLTGRHCRKFMQLT

DFDPFDPAIAADPYPHYRELLAGERVQYNPKRDVYILSRYADVREAARNH

DTLSSARGVTFSRGWLPFLPTSDPPAHTRMRKQLAPGMARGALETWRPMV

DQLARELVGGLLTQTPADVVSTVAAPMPMRAITSVLGVDGPDEAAFCRLS

NQAVRITDVALSASGLISLVQGFAGFRRLRALFTHRRDNGLLRECTVLGK

LATHAEQGRLSDDELFFFAVLLLVAGYESTAHMISTLFLTLADYPDQLTL

LAQQPDLIPSAIEEHLRFISPIQNICRTTRVDYSVGQAVIPAGSLVLLAW

GAANRDPRQYEDPDVFRADRNPVGHLAFGSGIHLCPGTQLARMEGQAILR

EIVANIDRIEVVEPPTWTTNANLRGLTRLRVAVTPRVAP

>CYP128A1 (2603412059) Mycobacterium tuberculosis TB_RSA157

MTATQSPPEPAPDRVRLAGCPLAGTPDVGLTAQDATTALGVPTRRRASSG

GIPVATSMWRDAQTVRTYGPAVAKALALRVAGKARSRLTGRHCRKFMQLT

DFDPFDPAIAADPYPHYRELLAGERVQYNPKRDVYILSRYADVREAARNH

DTLSSARGVTFSRGWLPFLPTSDPPAHTRMRKQLAPGMARGALETWRPMV

DQLARELVGGLLTQTPADVVSTVAAPMPMRAITSVLGVDGPDEAAFCRLS

NQAVRITDVALSASGLISLVQGFAGFRRLRALFTHRRDNGLLRECTVLGK

LATHAEQGRLSDDELFFFAVLLLVAGYESTAHMISTLFLTLADYPDQLTL

LAQQPDLIPSAIEEHLRFISPIQNICRTTRVDYSVGQAVIPAGSLVLLAW

GAANRDPRQYEDPDVFRADRNPVGHLAFGSGIHLCPGTQLARMEGQAILR

EIVANIDRIEVVEPPTWTTNANLRGLTRLRVAVTPRVAP

>CYP128A1 (2603890139) Mycobacterium tuberculosis BTB11-160

MTATQSPPEPAPDRVRLAGCPLAGTPDVGLTAQDATTALGVPTRRRASSG

GIPVATSMWRDAQTVRTYGPAVAKALALRVAGKARSRLTGRHCRKFMQLT

DFDPFDPAIAADPYPHYRELLAGERVQYNPKRDVYILSRYADVREAARNH

DTLSSARGVTFSRGWLPFLPTSDPPAHTRMRKQLAPGMARGALETWRPMV

DQLARELVGGLLTQTPADVVSTVAAPMPMRAITSVLGVDGPDEAAFCRLS

NQAVRITDVALSASGLISLVQGFAGFRRLRALFTHRRDNGLLRECTVLGK

LATHAEQGRLSDDELFFFAVLLLVAGYESTAHMISTLFLTLADYPDQLTL

LAQQPDLIPSAIEEHLRFISPIQNICRTTRVDYSVGQAVIPAGSLVLLAW

GAANRDPRQYEDPDVFRADRNPVGHLAFGSGIHLCPGTQLARMEGQAILR

EIVANIDRIEVVEPPTWTTNANLRGLTRLRVAVTPRVAP

>CYP128A1 (2605592908) Mycobacterium tuberculosis BTB05-371

MTATQSPPEPAPDRVRLAGCPLAGTPDVGLTAQDATTALGVPTRRRASSG

GIPVATSMWRDAQTVRTYGPAVAKALALRVAGKARSRLTGRHCRKFMQLT

DFDPFDPAIAADPYPHYRELLAGERVQYNPKRDVYILSRYADVREAARNH

DTLSSARGVTFSRGWLPFLPTSDPPAHTRMRKQLAPGMARGALETWRPMV

DQLARELVGGLLTQTPADVVSTVAAPMPMRAITSVLGVDGPDEAAFCRLS

NQAVRITDVALSASGLISLVQGFAGFRRLRALFTHRRDNGLLRECTVLGK

LATHAEQGRLSDDELFFFAVLLLVAGYESTAHMISTLFLTLADYPDQLTL

LAQQPDLIPSAIEEHLRFISPIQNICRTTRVDYSVGQAVIPAGSLVLLAW

GAANRDPRQYEDPDVFRADRNPVGHLAFGSGIHLCPGPSWRAWRVRRSCA

RSSPISTE

>CYP128A1 (2621723399) Mycobacterium tuberculosis 2074CJ

MTATQSPPEPAPDRVRLAGCPLAGTPDVGLTAQDATTALGVPTRRRASSG

GIPVATSMWRDAQTVRTYGPAVAKALALRVAGKARSRLTGRHCRKFMQLT

DFDPFDPAIAADPYPHYRELLAGERVQYNPKRDVYILSRYADVREAARNH

DTLSSARGVTFSRGWLPFLPTSDPPAHTRMRKQLAPGMARGALETWRPMV

DQLARELVGGLLTQTPADVVSTVAAPMPMRAITSVLGVDGPDEAAFCRLS

NQAVRITDVALSASGLISLVQGFAGFRRLRALFTHRRDNGLLRECTVLGK

LATHAEQGRLSDDELFFFAVLLLVAGYESTAHMISTLFLTLADYPDQLTL

LAQQPDLIPSAIEEHLRFISPIQNICRTTRVDYSVGQAVIPAGSLVLLAW

GAANRDPRQYEDPDVFRADRNPVGHLAFGSGIHLCPGPSWRAWRVRRSCA

RSSPISTE

>CYP128A1 (2621766309) Mycobacterium tuberculosis TKK_04_0139

MTATQSPPEPAPDRVRLAGCPLAGTPDVGLTAQDATTALGVPTRRRASSG

GIPVATSMWRDAQTVRTYGPAVAKALALRVAGKARSRLTGRHCRKFMQLT

DFDPFDPAIAADPYPHYRELLAGERVQYNPKRDVYILSRYADVREAARNH

DTLSSARGVTFSRGWLPFLPTSDPPAHTRMRKQLAPGMARGALETWRPMV

DQLARELVGGLLTQTPADVVSTVAAPMPMRAITSVLGVDGPDEAAFCRLS

NQAVRITDVALSASGLISLVQGFAGFRRLRALFTHRRDNGLLRECTVLGK

LATHAEQGRLSDDELFFFAVLLLVAGYESTAHMISTLFLTLADYPDQLTL

LAQQPDLIPSAIEEHLRFISPIQNICRTTRVDYSVGQAVIPAGSLVLLAW

GAANRDPRQYEDPDVFRADRNPVGHLAFGSGIHLCPGTQLARMEGQAILR

EIVANIDRIEVVEPPTWTTNANLRGLTRLRVAVTPRVAP

>CYP128A1 (2621928209) Mycobacterium tuberculosis TKK_05SA_0043

MTATQSPPEPAPDRVRLAGCPLAGTPDVGLTAQDATTALGVPTRRRASSG

GIPVATSMWRDAQTVRTYGPAVAKALALRVAGKARSRLTGRHCRKFMQLT

DFDPFDPAIAADPYPHYRELLAGERVQYNPKRDVYILSRYADVREAARNH

DTLSSARGVTFSRGWLPFLPTSDPPAHTRMRKQLAPGMARGALETWRPMV

DQLARELVGGLLTQTPADVVSTVAAPMPMRAITSVLGVDGPDEAAFCRLS

NQAVRITDVALSASGLISLVQGFAGFRRLRALFTHRRDNGLLRECTVLGK

LATHAEQGRLSDDELFFFAVLLLVAGYESTAHMISTLFLTLADYPDQLTL

LAQQPDLIPSAIEEHLRFISPIQNICRTTRVDYSVGQAVIPAGSLVLLAW

GAANRDPRQYEDPDVFRADRNPVGHLAFGSGIHLCPGTQLARMEGQAILR

EIVANIDRIEVVEPPTWTTNANLRGLTRLRVAVTPRVAP

>CYP128A1 (2720569018) Mycobacterium tuberculosis 0A036DS

MTATQSPPEPAPDRVRLAGCPLAGTPDVGLTAQDATTALGVPTRRRASSG

GIPVATSMWRDAQTVRTYGPAVAKALALRVAGKARSRLTGRHCRKFMQLT

DFDPFDPAIAADPYPHYRELLAGERVQYNPKRDVYILSRYADVREAARNH

DTLSSARGVTFSRGWLPFLPTSDPPAHTRMRKQLAPGMARGALETWRPMV

DQLARELVGGLLTQTPADVVSTVAAPMPMRAITSVLGVDGPDEAAFCRLS

NQAVRITDVALSASGLISLVQGFAGFRRLRALFTHRRDNGLLRECTVLGK

LATHAEQGRLSDDELFFFAVLLLVAGYESTAHMISTLFLTLADYPDQLTL

LAQQPDLIPSAIEEHLRFISPIQNICRTTRVDYSVGQAVIPAGSLVLLAW

GAANRDPRQYEDPDVFRADRNPVGHLAFGSGIHLCPGTQLARMEGQAILR

EIVANIDRIEVVEPPTWTTNANLRGLTRLRVAVTPRVAP

>CYP128A1 (2621426333) Mycobacterium tuberculosis XTB13-122

MTATQSPPEPAPDRVRLAGCPLAGTPDVGLTAQDATTALGVPTRRRAASG

GIPVATSMWRDAQTVRTYGPAVAKALALRVAGKARSRLTGRHCRKFMQLT

DFDPFDPAIAADPYPHYRELLAGERVQYNPKRDVYILSRYADVREAARNH

DTLSSARGVTFSRGWLPFLPTSDPPAHTRMRKQLAPGMARGALETWRPMV

DQLARELVGGLLTQTPADVVSTVAAPMPMRAITSVLGVDGPDEAAFCRLS

NQAVRITDVALSASGLISLVQGFAGFRRLRALFTHRRDNGLLRECTVLGK

LATHAEQGRLSDDELFFFAVLLLVAGYESTAHMISTLFLTLADYPDQLTL

LAQQPDLIPSAIEEHLRFISPIQNICRTTRVDYSVGQAVIPAGSLVLLAW

GAANRDPRQYEDPDVFRADRNPVGHLAFGSGIHLCPGTQLARMEGQAILR

EIVANIDRIEVVEPPTWTTNANLRGLTRLRVAVTPRVAP

>CYP128B4 (2803390603) Mycobacteroides abscessus abscessus 279

MSRWRSDSAVRQIGAPVVAAVGMNIAAAVRVRRRGYAGWTGAVNTDYDPL

DPATAAQPFDAYRALHAGGRVHYNPKRATFILSRHEDIRAALRDTDAVTS

SQGVTRMKISAPILVLTDGDDHTRLRKQVQPGFTRGAMSDWQGMADQLAK

ELVADVVANPGCDVMERLAVPLPIRMIAHIIGIPPEDVQNFRSWSEDGVG

VINAGVSPAGLRQGLKGVRAIAALRRYFKDQLASGKLKGSDTVLGRLVDN

NEDGKLSDDELFFIAMLLLFAGNETTTNLIGGMFDTLAHAPDQFAMIRDD

PDLIPSAVEEQLRYSAPIQNLYRYTRTDYRVGEVTIPSGSRLLLAFGAAN

RDPEVFEDPDTYRADRNPRNHIAFGYGVHMCIGATLSRMEGQAVLRELTS

QASAIAAAGSATWSTNSSLRGTTYLPIRLTPAR

>CYP128B2 (2810080781) Mycobacterium peregrinum 852002-51209_SCH5440388

MLKPLTSAIQMNLSAAVRTRRLGYRGWAGAVNTDYDPLDPATAAQPFEAY

AQLHSSDRVHYSPRRATWILHRLEDVRAALRDTEQVTSSHGVTRIRMAAD

LVVVTDGEDHNRLRKQVQPAFTKRALDSWQTTINDLAAELVDTLIAEPGS

DVVGRLAIPMPLRLIAAILGVPERDIDDFRRWSEDSVKLIDFTPTPAGVL

NTAKSLRAAMALRRYFLDHLATGELKGSDTVLGRLLEHNTDGTLTDDQLF

YIAILLLIAGNETTTNLLGGLFDTFARYPDQYDMIRAEPDLIPMAVEEQL

RYGSPVQNLYRYTRAPYRVGEVTIPSGARLLLSFGAANRDPSAFDDPDTY

RADRNPRAHVAFGYGAHMCLGAPLARMEAVAVLRELVNRVSRISATAPAV

WSTNSSLRGPVHLPVRLHC

>CYP128A1 (2555160922) Mycobacterium tuberculosis PanR0207

MTATQSPPEPAPDRVRLAGCPLAGTPDVGLTAQDATTALGVPTRRRASSG

GIPVATSMWRDAQTVRTYGPAVAKALALRVAGKARSRLTGRHCRKFMQLT

DFDPFDPAIAADPYPHYRELLAGERVQYNPKRDVYILSRYADVREAARNH

DTLSSARGVTFSRGWLPFLPTSDPPAHTRMRKQLAPGMARGALETWRPMV

DQLARELVGGLLTQTPADVVSTVAAPMPMRAITSVLGVDGPDEAAFCRLS

NQAVRITDVALSASGLISLVQGFAGFRRLRALFTHRRDNGLLRECTVLGK

LATHAEQGRLSDDELFFFAVLLLVAGYESTAHMISTLFLTLADYPDQLTL

LAQQPDLIPSAIEEHLRFISPIQNICRTTRVDYSVGQAVIPAGSLVLLAW

GAANRDPRQYEDPDVFRADRNPVGHLAFGSGIHLCPGTQLARMEGQAILR

EIVANIDRIEVVEPPTWTTNANLRGLTRLRVAVTPRVAP

>CYP128A1 (2575477975) Mycobacterium tuberculosis TB_RSA90

MTATQSPPEPAPDRVRLAGCPLAGTPDVGLTAQDATTALGVPTRRRASSG

GIPVATSMWRDAQTVRTYGPAVAKALALRVAGKARSRLTGRHCRKFMQLT

DFDPFDPAIAADPYPHYRELLAGERVQYNPKRDVYILSRYADVREAARNH

DTLSSARGVTFSRGWLPFLPTSDPPAHTRMRKQLAPGMARGALETWRPMV

DQLARELVGGLLTQTPADVVSTVAAPMPMRAITSVLGVDGPDEAAFCRLS

NQAVRITDVALSASGLISLVQGFAGFRRLRALFTHRRDNGLLRECTVLGK

LATHAEQGRLSDDELFFFAVLLLVAGYESTAHMISTLFLTLADYPDQLTL

LAQQPDLIPSAIEEHLRFISPIQNICRTTRVDYSVGQAVIPAGSLVLLAW

GAANRDPRQYEDPDVFRADRNPVGHLAFGSGIHLCPGTQLARMEGQAILR

EIVANIDRIEVVEPPTWTTNANLRGLTRLRVAVTPRVAP

>CYP128A1 (2577519954) Mycobacterium tuberculosis MD15212

MTATQSPPEPAPDRVRLAGCPLAGTPDVGLTAQDATTALGVPTRRRASSG

GIPVATSMWRDAQTVRTYGPAVAKALALRVAGKARSRLTGRHCRKFMQLT

DFDPFDPAIAADPYPHYRELLAGERVQYNPKRDVYILSRYADVREAARNH

DTLSSARGVTFSRGWLPFLPTSDPPAHTRMRKQLAPGMARGALETWRPMV

DQLARELVGGLLTQTPADVVSTVAAPMPMRAITSVLGVDGPDEAAFCRLS

NQAVRITDVALSASGLISLVQGFAGFRRLRALFTHRRDNGLLRECTVLGK

LATHAEQGRLSDDELFFFAVLLLVAGYESTAHMISTLFLTLADYPDQLTL

LAQQPDLIPSAIEEHLRFISPIQNICRTTRVDYSVGQAVIPAGSLVLLAW

GAANRDPRQYEDPDVFRADRNPVGHLAFGSGIHLCPGTQLARMEGQAILR

EIVANIDRIEVVEPPTWTTNANLRGLTRLRVAVTPRVAP

>CYP128A1 (2578171622) Mycobacterium tuberculosis BTB11-236

MTATQSPPEPAPDRVRLAGCPLAGTPDVGLTAQDATTALGVPTRRRASSG

GIPVATSMWRDAQTVRTYGPAVAKALALRVAGKARSRLTGRHCRKFMQLT

DFDPFDPAIAADPYPHYRELLAGERVQYNPKRDVYILSRYADVREAARNH

DTLSSARGVTFSRGWLPFLPTSDPPAHTRMRKQLAPGMARGALETWRPMV

DQLARELVGGLLTQTPADVVSTVAAPMPMRAITSVLGVDGPDEAAFCRLS

NQAVRITDVALSASGLISLVQGFAGFRRLRALFTHRRDNGLLRECTVLGK

LATHAEQGRLSDDELFFFAVLLLVAGYESTAHMISTLFLTLADYPDQLTL

LAQQPDLIPSAIEEHLRFISPIQNICRTTRVDYSVGQAVIPAGSLVLLAW

GAANRDPRQYEDPDVFRADRNPVGHLAFGSGIHLCPGTQLARMEGQAILR

EIVANIDRIEVVEPPTWTTNANLRGLTRLRVAVTPRVAP

>CYP128A1 (2584841660) Mycobacterium tuberculosis TKK_04_0060

MTATQSPPEPAPDRVRLAGCPLAGTPDVGLTAQDATTALGVPTRRRASSG

GIPVATSMWRDAQTVRTYGPAVAKALALRVAGKARSRLTGRHCRKFMQLT

DFDPFDPAIAADPYPHYRELLAGERVQYNPKRDVYILSRYADVREAARNH

DTLSSARGVTFSRGWLPFLPTSDPPAHTRMRKQLAPGMARGALETWRPMV

DQLARELVGGLLTQTPADVVSTVAAPMPMRAITSVLGVDGPDEAAFCRLS

NQAVRITDVALSASGLISLVQGFAGFRRLRALFTHRRDNGLLRECTVLGK

LATHAEQGRLSDDELFFFAVLLLVAGYESTAHMISTLFLTLADYPDQLTL

LAQQPDLIPSAIEEHLRFISPIQNICRTTRVDYSVGQAVIPAGSLVLLAW

GAANRDPRQYEDPDVFRADRNPVGHLAFGSGIHLCPGTQLARMEGQAILR

EIVANIDRIEVVEPPTWTTNANLRGLTRLRVAVTPRVAP

>CYP128A1 (2621878489) Mycobacterium tuberculosis TKK_05MA_2005

MTATQSPPEPAPDRVRLAGCPLAGTPDVGLTAQDATTALGVPTRRRASSG

GIPVATSMWRDAQTVRTYGPAVAKALALRVAGKARSRLTGRHCRKFMQLT

DFDPFDPAIAADPYPHYRELLAGERVQYNPKRDVYILSRYADVREAARNH

DTLSSARGVTFSRGWLPFLPTSDPPAHTRMRKQLAPGMARGALETWRPMV

DQLARELVGGLLTQTPADVVSTVAAPMPMRAITSVLGVDGPDEAAFCRLS

NQAVRITDVALSASGLISLVQGFAGFRRLRALFTHRRDNGLLRECTVLGK

LATHAEQGRLSDDELFFFAVLLLVAGYESTAHMISTLFLTLADYPDQLTL

LAQQPDLIPSAIEEHLRFISPIQNICRTTRVDYSVGQAVIPAGSLVLLAW

GAANRDPRQYEDPDVFRADRNPVGHLAFGSGIHLCPGTQLARMEGQAILR

EIVANIDRIEVVEPPTWTTNANLRGLTRLRVAVTPRVAP

>CYP128A1 (2622201273) Mycobacterium tuberculosis MD14832

MTATQSPPEPAPDRVRLAGCPLAGTPDVGLTAQDATTALGVPTRRRASSG

GIPVATSMWRDAQTVRTYGPAVAKALALRVAGKARSRLTGRHCRKFMQLT

DFDPFDPAIAADPYPHYRELLAGERVQYNPKRDVYILSRYADVREAARNH

DTLSSARGVTFSRGWLPFLPTSDPPAHTRMRKQLAPGMARGALETWRPMV

DQLARELVGGLLTQTPADVVSTVAAPMPMRAITSVLGVDGPDEAAFCRLS

NQAVRITDVALSASGLISLVQGFAGFRRLRALFTHRRDNGLLRECTVLGK

LATHAEQGRLSDDELFFFAVLLLVAGYESTAHMISTLFLTLADYPDQLTL

LAQQPDLIPSAIEEHLRFISPIQNICRTTRVDYSVGQAVIPAGSLVLLAW

GAANRDPRQYEDPDVFRADRNPVGHLAFGSGIHLCPGTQLARMEGQAILR

EIVANIDRIEVVEPPTWTTNANLRGLTRLRVAVTPRVAP

>CYP128A1 (2630814333) Mycobacterium tuberculosis MD15774

MTATQSPPEPAPDRVRLAGCPLAGTPDVGLTAQDATTALGVPTRRRASSG

GIPVATSMWRDAQTVRTYGPAVAKALALRVAGKARSRLTGRHCRKFMQLT

DFDPFDPAIAADPYPHYRELLAGERVQYNPKRDVYILSRYADVREAARNH

DTLSSARGVTFSRGWLPFLPTSDPPAHTRMRKQLAPGMARGALETWRPMV

DQLARELVGGLLTQTPADVVSTVAAPMPMRAITSVLGVDGPDEAAFCRLS

NQAVRITDVALSASGLISLVQGFAGFRRLRALFTHRRDNGLLRECTVLGK

LATHAEQGRLSDDELFFFAVLLLVAGYESTAHMISTLFLTLADYPDQLTL

LAQQPDLIPSAIEEHLRFISPIQNICRTTRVDYSVGQAVIPAGSLVLLAW

GAANRDPRQYEDPDVFRADRNPVGHLAFGSGIHLCPGTQLARMEGQAILR

EIVANIDRIEVVEPPTWTTNANLRGLTRLRVAVTPRVAP

>CYP128A1 (2636778551) Mycobacterium tuberculosis TKK_04_0104

MTATQSPPEPAPDRVRLAGCPLAGTPDVGLTAQDATTALGVPTRRRASSG

GIPVATSMWRDAQTVRTYGPAVAKALALRVAGKARSRLTGRHCRKFMQLT

DFDPFDPAIAADPYPHYRELLAGERVQYNPKRDVYILSRYADVREAARNH

DTLSSARGVTFSRGWLPFLPTSDPPAHTRMRKQLAPGMARGALETWRPMV

DQLARELVGGLLTQTPADVVSTVAAPMPMRAITSVLGVDGPDEAAFCRLS

NQAVRITDVALSASGLISLVQGFAGFRRLRALFTHRRDNGLLRECTVLGK

LATHAEQGRLSDDELFFFAVLLLVAGYESTAHMISTLFLTLADYPDQLTL

LAQQPDLIPSAIEEHLRFISPIQNICRTTRVDYSVGQAVIPAGSLVLLAW

GAANRDPRQYEDPDVFRADRNPVGHLAFGSGIHLCPGTQLARMEGQAILR

EIVANIDRIEVVEPPTWTTNANLRGLTRLRVAVTPRVAP

>CYP128A1 (2649200941) Mycobacterium tuberculosis TKK_04_0068

MTATQSPPEPAPDRVRLAGCPLAGTPDVGLTAQDATTALGVPTRRRASSG

GIPVATSMWRDAQTVRTYGPAVAKALALRVAGKARSRLTGRHCRKFMQLT

DFDPFDPAIAADPYPHYRELLAGERVQYNPKRDVYILSRYADVREAARNH

DTLSSARGVTFSRGWLPFLPTSDPPAHTRMRKQLAPGMARGALETWRPMV

DQLARELVGGLLTQTPADVVSTVAAPMPMRAITSVLGVDGPDEAAFCRLS

NQAVRITDVALSASGLISLVQGFAGFRRLRALFTHRRDNGLLRECTVLGK

LATHAEQGRLSDDELFFFAVLLLVAGYESTAHMISTLFLTLADYPDQLTL

LAQQPDLIPSAIEEHLRFISPIQNICRTTRVDYSVGQAVIPAGSLVLLAW

GAANRDPRQYEDPDVFRADRNPVGHLAFGSGIHLCPGTQLARMEGQAILR

EIVANIDRIEVVEPPTWTTNANLRGLTRLRVAVTPRVAP

>CYP128A1 (2649310289) Mycobacterium tuberculosis Mtb562

MTATQSPPEPAPDRVRLAGCPLAGTPDVGLTAQDATTALGVPTRRRASSG

GIPVATSMWRDAQTVRTYGPAVAKALALRVAGKARSRLTGRHCRKFMQLT

DFDPFDPAIAADPYPHYRELLAGERVQYNPKRDVYILSRYADVREAARNH

DTLSSARGVTFSRGWLPFLPTSDPPAHTRMRKQLAPGMARGALETWRPMV

DQLARELVGGLLTQTPADVVSTVAAPMPMRAITSVLGVDGPDEAAFCRLS

NQAVRITDVALSASGLISLVQGFAGFRRLRALFTHRRDNGLLRECTVLGK

LATHAEQGRLSDDELFFFAVLLLVAGYESTAHMISTLFLTLADYPDQLTL

LAQQPDLIPSAIEEHLRFISPIQNICRTTRVDYSVGQAVIPAGSLVLLAW

GAANRDPRQYEDPDVFRADRNPVGHLAFGSGIHLCPGTQLARMEGQAILR

EIVANIDRIEVVEPPTWTTNANLRGLTRLRVAVTPRVAP

>CYP128A1 (2678682379) Mycobacterium tuberculosis W09900339

MTATQSPPEPAPDRVRLAGCPLAGTPDVGLTAQDATTALGVPTRRRASSG

GIPVATSMWRDAQTVRTYGPAVAKALALRVAGKARSRLTGRHCRKFMQLT

DFDPFDPAIAADPYPHYRELLAGERVQYNPKRDVYILSRYADVREAARNH

DTLSSARGVTFSRGWLPFLPTSDPPAHTRMRKQLAPGMARGALETWRPMV

DQLARELVGGLLTQTPADVVSTVAAPMPMRAITSVLGVDGPDEAAFCRLS

NQAVRITDVALSASGLISLVQGFAGFRRLRALFTHRRDNGLLRECTVLGK

LATHAEQGRLSDDELFFFAVLLLVAGYESTAHMISTLFLTLADYPDQLTL

LAQQPDLIPSAIEEHLRFISPIQNICRTTRVDYSVGQAVIPAGSLVLLAW

GAANRDPRQYEDPDVFRADRNPVGHLAFGSGIHLCPGPSWRAWRVRRSCA

RSSPISTE

>CYP128A1 (2696136240) Mycobacterium tuberculosis A70451

MTATQSPPEPAPDRVRLAGCPLAGTPDVGLTAQDATTALGVPTRRRASSG

GIPVATSMWRDAQTVRTYGPAVAKALALRVAGKARSRLTGRHCRKFMQLT

DFDPFDPAIAADPYPHYRELLAGERVQYNPKRDVYILSRYADVREAARNH

DTLSSARGVTFSRGWLPFLPTSDPPAHTRMRKQLAPGMARGALETWRPMV

DQLARELVGGLLTQTPADVVSTVAAPMPMRAITSVLGVDGPDEAAFCRLS

NQAVRITDVALSASGLISLVQGFAGFRRLRALFTHRRDNGLLRECTVLGK

LATHAEQGRLSDDELFFFAVLLLVAGYESTAHMISTLFLTLADYPDQLTL

LAQQPDLIPSAIEEHLRFISPIQNICRTTRVDYSVGQAVIPAGSLVLLAW

GAANRDPRQYEDPDVFRADRNPVGHLAFGSGIHLCPGTQLARMEGQAILR

EIVANIDRIEVVEPPTWTTNANLRGLTRLRVAVTPRVAP

>CYP128A1 (2589587598) Mycobacterium tuberculosis TKK-01-0043

MTATQSPPEPAPDRVRLAGCPLAGTPDVGLTAQDATTALGVPTRRRASSG

GIPVATSMWRDAQTVRTYGPAVAKALALRVAGKARSRLTGRHCRKFMQLT

DFDPFDPAIAADPYPHYRELLAGERVQYNPKRDVYILSRYADVREAARNH

DTLSSARGVTFSRGWLPFLPTSDPPAHTRMRKQLAPGMARGALETWRPMV

DQLARELVGGLLTQTPADVVSTVAAPMPMRAITSVLGVDGPDEAAFCRLS

NQAVRITDVALSASGLISLVQGFAGFRRLRALFTHRRDNGLLRECTVLGK

LATHAEQGRLSDDELFFFAVLLLVAGYESPAHMISTLFLTLADYPDQLTL

LAQQPDLIPSAIEEHLRFISPIQNICRTTRVDYSVGQAVIPAGSLVLLAW

GAANRDPRQYEDPDVFRADRNPVGHLAFGSGIHLCPGTQLARMEGQAILR

EIVANIDRIEVVEPPTWTTNANLRGLTRLRVAVTPRVAP

>CYP128A1 (2635674489) Mycobacterium tuberculosis NRITLD16

MTATQSPPEPAPDRVRLAGCPLAGTPDVGLTAQDATTALGVPTRRRAASG

GIPVATSMWRDAQTVRTYGPAVAKALALRVAGKARSRLTGRHCRKFMQLT

DFDPFDPAIAADPYPHYRELLAGERVQYNPKRDVYILSRYADVREAARNH

DTLSSARGVTFSRGWLPFLPTSDPPAHTRMRKQLAPGMARGALETWRPMV

DQLARELVGGLLTQTPADVVSTVAAPMPMRAITSVLGVDGPDEAAFCRLS

NQAVRITDVALSASGLISLVQGFAGFRRLRALFTHRRDNGLLRECTVLGK

LATHAEQGRLSDDELFFFAVLLLVAGYESTAHMISTLFLTLADYPDQLTL

LAQQPDLIPSAIEEHLRFISPIQNICRTTRVDYSVGQAVIPAGSLVLLAW

GAANRDPRQYEDPDVFRADRNPVGHLAFGSGIHLCPGTQLARMEGQAILR

EIVANIDRIEVVEPPTWTTNANLRGLTRLRVAVTPRVAP

>CYP128A1 (2587481339) Mycobacterium kansasii SMC1

MRLALRAAGKVRSRLDVKRSQKQVQFTDFDPFDPVTAADPYPHYRELLAG

ERVQYNPKRDVYIVSRYTDVREAARNHHALSSAGGVTFSRGCLPFLPTSD

PPTHTRLRKQLAPSMARGAVESWRPMIDRLAHELVAELLTRTPADVVATV

AAPLPMRTITAVLGVPGPDEGMFCRLSNQAARITDVNLSASGLVSLAQGF

TGFRRLRALFTQRRATGLLGEHTVFGRLAAHAEHGRLSDDELFFFAVLLL

VAGYESTAHMISTLFLTLTEFPDQLSLLARRPDLIPSAIEEQLRFVSPIQ

NICRTTRVDYPVGGAVIPAGSLVLLAWGAANRDPRQYDDPDVFRADRNPT

GHLAFGSGIHLCPGTQLARMEGHAVLREIVTNIDRIDVVEPPAWTTNANL

RGPTRLRVAVTPRASR

>CYP128B1 (2810637513) Mycobacterium asiaticum 1137369.8

MAAKQAFQGTAEGARLLGHAARMNLAAAVRTRRRGCAGWTGAINTDYDPQ

DPLTAAQPFDAYRALHRGGRVHYNPRRATFIISRLDDVRAALRDTDQVTS

SQGVTRLRMSAPLAVLTDGEEHARLRRQVQPGFSKGAMKAWQGMIEELAE

ELVGDVLANPGCDVVRQLAIPMPIRLIAKILGIPNDDVGDFRRWSERGVG

VMDVTPTLPGLIGAARSVTAMAALQRYFVKQFTAGGLKGSDTVLGRLLAH

NTDGSLTDRQLLLIAIHLLIAGNETTTNLLGGMFDTLAHHPDQYEMIRAQ

PDLIPLAVEEQLRITTPIQNLYRYTRADYQVGDVTIPNGSRVLLSFGAAN

RDPTAFEEPDRYRADRNPRTHVAFGYGAHMCLGAPLARMEAQAVLRQLIT

RVSRITPAGPTTWSTHSSLRGPTRLPIRLTAA

>CYP128A1 (2555149124) Mycobacterium tuberculosis PanR0203

MTATQSPPEPAPDRVRLAGCPLAGTPDVGLTAQDATTALGVPTRRRASSG

GIPVATSMWRDAQTVRTYGPAVAKALALRVAGKARSRLTGRHCRKFMQLT

DFDPFDPAIAADPYPHYRELLAGERVQYNPKRDVYILSRYADVREAARNH

DTLSSARGVTFSRGWLPFLPTSDPPAHTRMRKQLAPGMARGALETWRPMV

DQLARELVGGLLTQTPADVVSTVAAPMPMRAITSVLGVDGPDEAAFCRLS

NQAVRITDVALSASGLISLVQGFAGFRRLRALFTHRRDNGLLRECTVLGK

LATHAEQGRLSDDELFFFAVLLLVAGYESTAHMISTLFLTLADYPDQLTL

LAQQPDLIPSAIEEHLRFISPIQNICRTTRVDYSVGQAVIPAGSLVLLAW

GAANRDPRQYEDPDVFRADRNPVGHLAFGSGIHLCPGTQLARMEGQAILR

EIVANIDRIEVVEPPTWTTNANLRGLTRLRVAVTPRVAP

>CYP128A1 (2575305152) Mycobacterium tuberculosis UG-C

MTATQSPPEPAPDRVRLAGCPLAGTPDVGLTAQDATTALGVPTRRRASSG

GIPVATSMWRDAQTVRTYGPAVAKALALRVAGKARSRLTGRHCRKFMQLT

DFDPFDPAIAADPYPHYRELLAGERVQYNPKRDVYILSRYADVREAARNH

DTLSSARGVTFSRGWLPFLPTSDPPAHTRMRKQLAPGMARGALETWRPMV

DQLARELVGGLLTQTPADVVSTVAAPMPMRAITSVLGVDGPDEAAFCRLS

NQAVRITDVALSASGLISLVQGFAGFRRLRALFTHRRDNGLLRECTVLGK

LATHAEQGRLSDDELFFFAVLLLVAGYESTAHMISTLFLTLADYPDQLTL

LAQQPDLIPSAIEEHLRFISPIQNICRTTRVDYSVGQAVIPAGSLVLLAW

GAANRDPRQYEDPDVFRADRNPVGHLAFGSGIHLCPGTQLARMEGQAILR

EIVANIDRIEVVEPPTWTTNANLRGLTRLRVAVTPRVAP

>CYP128A1 (2576164460) Mycobacterium tuberculosis TB_RSA140

MTATQSPPEPAPDRVRLAGCPLAGTPDVGLTAQDATTALGVPTRRRASSG

GIPVATSMWRDAQTVRTYGPAVAKALALRVAGKARSRLTGRHCRKFMQLT

DFDPFDPAIAADPYPHYRELLAGERVQYNPKRDVYILSRYADVREAARNH

DTLSSARGVTFSRGWLPFLPTSDPPAHTRMRKQLAPGMARGALETWRPMV

DQLARELVGGLLTQTPADVVSTVAAPMPMRAITSVLGVDGPDEAAFCRLS

NQAVRITDVALSASGLISLVQGFAGFRRLRALFTHRRDNGLLRECTVLGK

LATHAEQGRLSDDELFFFAVLLLVAGYESTAHMISTLFLTLADYPDQLTL

LAQQPDLIPSAIEEHLRFISPIQNICRTTRVDYSVGQAVIPAGSLVLLAW

GAANRDPRQYEDPDVFRADRNPVGHLAFGSGIHLCPGTQLARMEGQAILR

EIVANIDRIEVVEPPTWTTNANLRGLTRLRVAVTPRVAP

>CYP128A1 (2577312756) Mycobacterium tuberculosis TRUG0085

MTATQSPPEPAPDRVRLAGCPLAGTPDVGLTAQDATTALGVPTRRRASSG

GIPVATSMWRDAQTVRTYGPAVAKALALRVAGKARSRLTGRHCRKFMQLT

DFDPFDPAIAADPYPHYRELLAGERVQYNPKRDVYILSRYADVREAARNH

DTLSSARGVTFSRGWLPFLPTSDPPAHTRMRKQLAPGMARGALETWRPMV

DQLARELVGGLLTQTPADVVSTVAAPMPMRAITSVLGVDGPDEAAFCRLS

NQAVRITDVALSASGLISLVQGFAGFRRLRALFTHRRDNGLLRECTVLGK

LATHAEQGRLSDDELFFFAVLLLVAGYESTAHMISTLFLTLADYPDQLTL

LAQQPDLIPSAIEEHLRFISPIQNICRTTRVDYSVGQAVIPAGSLVLLAW

GAANRDPRQYEDPDVFRADRNPVGHLAFGSGIHLCPGTQLARMEGQAILR

EIVANIDRIEVVEPPTWTTNANLRGLTRLRVAVTPRVAP

>CYP128A1 (2584866541) Mycobacterium tuberculosis TB_RSA46

MTATQSPPEPAPDRVRLAGCPLAGTPDVGLTAQDATTALGVPTRRRASSG

GIPVATSMWRDAQTVRTYGPAVAKALALRVAGKARSRLTGRHCRKFMQLT

DFDPFDPAIAADPYPHYRELLAGERVQYNPKRDVYILSRYADVREAARNH

DTLSSARGVTFSRGWLPFLPTSDPPAHTRMRKQLAPGMARGALETWRPMV

DQLARELVGGLLTQTPADVVSTVAAPMPMRAITSVLGVDGPDEAAFCRLS

NQAVRITDVALSASGLISLVQGFAGFRRLRALFTHRRDNGLLRECTVLGK

LATHAEQGRLSDDELFFFAVLLLVAGYESTAHMISTLFLTLADYPDQLTL

LAQQPDLIPSAIEEHLRFISPIQNICRTTRVDYSVGQAVIPAGSLVLLAW

GAANRDPRQYEDPDVFRADRNPVGHLAFGSGIHLCPGTQLARMEGQAILR

EIVANIDRIEVVEPPTWTTNANLRGLTRLRVAVTPRVAP

>CYP128A1 (2604302343) Mycobacterium tuberculosis TB_RSA172

MTATQSPPEPAPDRVRLAGCPLAGTPDVGLTAQDATTALGVPTRRRASSG

GIPVATSMWRDAQTVRTYGPAVAKALALRVAGKARSRLTGRHCRKFMQLT

DFDPFDPAIAADPYPHYRELLAGERVQYNPKRDVYILSRYADVREAARNH

DTLSSARGVTFSRGWLPFLPTSDPPAHTRMRKQLAPGMARGALETWRPMV

DQLARELVGGLLTQTPADVVSTVAAPMPMRAITSVLGVDGPDEAAFCRLS

NQAVRITDVALSASGLISLVQGFAGFRRLRALFTHRRDNGLLRECTVLGK

LATHAEQGRLSDDELFFFAVLLLVAGYESTAHMISTLFLTLADYPDQLTL

LAQQPDLIPSAIEEHLRFISPIQNICRTTRVDYSVGQAVIPAGSLVLLAW

GAANRDPRQYEDPDVFRADRNPVGHLAFGSGIHLCPGTQLARMEGQAILR

EIVANIDRIEVVEPPTWTTNANLRGLTRLRVAVTPRVAP

>CYP128A1 (2605800579) Mycobacterium tuberculosis BTB07-234

MTATQSPPEPAPDRVRLAGCPLAGTPDVGLTAQDATTALGVPTRRRASSG

GIPVATSMWRDAQTVRTYGPAVAKALALRVAGKARSRLTGRHCRKFMQLT

DFDPFDPAIAADPYPHYRELLAGERVQYNPKRDVYILSRYADVREAARNH

DTLSSARGVTFSRGWLPFLPTSDPPAHTRMRKQLAPGMARGALETWRPMV

DQLARELVGGLLTQTPADVVSTVAAPMPMRAITSVLGVDGPDEAAFCRLS

NQAVRITDVALSASGLISLVQGFAGFRRLRALFTHRRDNGLLRECTVLGK

LATHAEQGRLSDDELFFFAVLLLVAGYESTAHMISTLFLTLADYPDQLTL

LAQQPDLIPSAIEEHLRFISPIQNICRTTRVDYSVGQAVIPAGSLVLLAW

GAANRDPRQYEDPDVFRADRNPVGHLAFGSGIHLCPGTQLARMEGQAILR

EIVANIDRIEVVEPPTWTTNANLRGLTRLRVAVTPRVAP

>CYP128A1 (2622610230) Mycobacterium tuberculosis M1570

MTATQSPPEPAPDRVRLAGCPLAGTPDVGLTAQDATTALGVPTRRRASSG

GIPVATSMWRDAQTVRTYGPAVAKALALRVAGKARSRLTGRHCRKFMQLT

DFDPFDPAIAADPYPHYRELLAGERVQYNPKRDVYILSRYADVREAARNH

DTLSSARGVTFSRGWLPFLPTSDPPAHTRMRKQLAPGMARGALETWRPMV

DQLARELVGGLLTQTPADVVSTVAAPMPMRAITSVLGVDGPDEAAFCRLS

NQAVRITDVALSASGLISLVQGFAGFRRLRALFTHRRDNGLLRECTVLGK

LATHAEQGRLSDDELFFFAVLLLVAGYESTAHMISTLFLTLADYPDQLTL

LAQQPDLIPSAIEEHLRFISPIQNICRTTRVDYSVGQAVIPAGSLVLLAW

GAANRDPRQYEDPDVFRADRNPVGHLAFGSGIHLCPGTQLARMEGQAILR

EIVANIDRIEVVEPPTWTTNANLRGLTRLRVAVTPRVAP

>CYP128A1 (2659397888) Mycobacterium tuberculosis A70011_4

MTATQSPPEPAPDRVRLAGCPLAGTPDVGLTAQDATTALGVPTRRRASSG

GIPVATSMWRDAQTVRTYGPAVAKALALRVAGKARSRLTGRHCRKFMQLT

DFDPFDPAIAADPYPHYRELLAGERVQYNPKRDVYILSRYADVREAARNH

DTLSSARGVTFSRGWLPFLPTSDPPAHTRMRKQLAPGMARGALETWRPMV

DQLARELVGGLLTQTPADVVSTVAAPMPMRAITSVLGVDGPDEAAFCRLS

NQAVRITDVALSASGLISLVQGFAGFRRLRALFTHRRDNGLLRECTVLGK

LATHAEQGRLSDDELFFFAVLLLVAGYESTAHMISTLFLTLADYPDQLTL

LAQQPDLIPSAIEEHLRFISPIQNICRTTRVDYSVGQAVIPAGSLVLLAW

GAANRDPRQYEDPDVFRADRNPVGHLAFGSGIHLCPGTQLARMEGQAILR

EIVANIDRIEVVEPPTWTTNANLRGLTRLRVAVTPRVAP

>CYP128A1 (2706609331) Mycobacterium tuberculosis J09700837

MTATQSPPEPAPDRVRLAGCPLAGTPDVGLTAQDATTALGVPTRRRASSG

GIPVATSMWRDAQTVRTYGPAVAKALALRVAGKARSRLTGRHCRKFMQLT

DFDPFDPAIAADPYPHYRELLAGERVQYNPKRDVYILSRYADVREAARNH

DTLSSARGVTFSRGWLPFLPTSDPPAHTRMRKQLAPGMARGALETWRPMV

DQLARELVGGLLTQTPADVVSTVAAPMPMRAITSVLGVDGPDEAAFCRLS

NQAVRITDVALSASGLISLVQGFAGFRRLRALFTHRRDNGLLRECTVLGK

LATHAEQGRLSDDELFFFAVLLLVAGYESTAHMISTLFLTLADYPDQLTL

LAQQPDLIPSAIEEHLRFISPIQNICRTTRVDYSVGQAVIPAGSLVLLAW

GAANRDPRQYEDPDVFRADRNPVGHLAFGSGIHLCPGTQLARMEGQAILR

EIVANIDRIEVVEPPTWTTNANLRGLTRLRVAVTPRVAP

>CYP128A1 (2583732568) Mycobacterium africanum MAL020135

MTATQSPPEPAPDRVRLAGCPLAGTPDVGLTAQDATTALGVPTRRRASSG

GIPVATSMWRDAQTVRTYGPAVAKALALRVAGKARSRLTGRHCRKFMLLT

DFDPFDPAIAADPYPHYRELLAGERVQYNPKRDVYILSRYADVREAARNH

DTLSSARGVTFSRGWLPFLPTSDPPAHTRMRKQLAPGMARGALETWRPMV

DQLARELVGGLLTQTPADVVSTVAAPMPMRAITSVLGVDGPDEAAFCRLS

NQAVRITDVALSASGLISLVQGFAGFRRLRALFTHRRDNGLLRECTVLGK

LATHAEQGRLSDDELFFFAVLLLVAGYESTAHMISTLFLTLADYPDQLTL

LAQQPDLIPSAIEEHLRFISPIQNICRTTRVDYSVGQAVIPAGSLVLLAW

GAANRDPRQYEDPDVFRADRNPVGHLAFGSGIHLCPGTQLARMEGQAILR

EIVANIDRIEVVEPPTWTTNANLRGLTRLRVAVTPRVAP

>CYP128A1 (2590524167) Mycobacterium tuberculosis KT-0089

MTATQSPPEPAPDRVRLAGCPLAGTPDVGLTAQDATTALGVPTRRRASSG

GIPVATSMWRDAQTVRTYGPAVAKALALRVAGKARSRLTGRHCRKFMQLT

DFDPFDPAIAADPYPHYRELLAGERVQYNPKRDVYILSRYADVREAARNH

DTLSSARGVTFSRGWLPFLPTSDPPAHTRMRKQLAPGMARGALETWRPMV

DQLARELVGGLLTQTPADVVSTVAAPMPMRAITSVLGVDGPDEAAFCRLS

NQAVRITDVALSASGLISLVQGFAGFRRLRALFTHRRDNGLLRECTVLGK

LAAHAEQGRLSDDELFFFAVLLLVAGYESTAHMISTLFLTLADYPDQLTL

LAQQPDLIPSAIEEHLRFISPIQNICRTTRVDYSVGQAVIPAGSLVLLAW

GAANRDPRQYEDPDVFRADRNPVGHLAFGSGIHLCPGTQLARMEGQAILR

EIVANIDRIEVVEPPTWTTNANLRGLTRLRVAVTPRVAP

>CYP128A1 (2603196603) Mycobacterium tuberculosis TRUG0036

MTATQSPPEPAPDRVRLAGCPLAGTPDVGLTAQDATTALGVPTRRRASSG

GIPVATSMWRDAQTVRTYGPAVAKALALRVAGKARSRLTGRHCRKFMQLT

DFDPFDPAIAADPYPHYRELLAGERVQYNPKRDVYILSRYADVREAARNH

DTLSSARGVTFSRGWLPFLPTSDPPAHTRMRKQLAPGMARGALETWRPMV

DQLARELVGGLLTQTPADVVSTVVAPMPMRAITSVLGVDGPDEAAFCRLS

NQAVRITDVALSASGLISLVQGFAGFRRLRALFTHRRDNGLLRECTVLGK

LATHAEQGRLSDDELFFFAVLLLVAGYESTAHMISTLFLTLADYPDQLTL

LAQQPDLIPSAIEEHLRFISPIQNICRTTRVDYSVGQAVIPAGSLVLLAW

GAANRDPRQYEDPDVFRADRNPVGHLAFGSGIHLCPGTQLARMEGQAILR

EIVANIDRIEVVEPPTWTTNANLRGLTRLRVAVTPRVAP

>CYP128A1 (2622801911) Mycobacterium tuberculosis M2010

MTATQSPPEPAPDRVRLAGCPLAGTPDVGLTAQDATTALGVPTRRRASSG

GIPVATSMWRDAQTVRTYGPAVAKALALRVAGKARSRLTGRHCRKFMQLT

DFDPFDPAIAADPYPHYRELLAGERVQYNPKRDVYILSRYADVREAARNH

DTLSSSRGVTFSRGWLPFLPTSDPPAHTRMRKQLAPGMARGALETWRPMV

DQLARELVGGLLTQTPADVVSTVAAPMPMRAITSVLGVDGPDEAAFCRLS

NQAVRITDVALSASGLISLVQGFAGFRRLRALFTHRRDNGLLRECTVLGK

LATHAEQGRLSDDELFFFAVLLLVAGYESTAHMISTLFLTLADYPDQLTL

LAQQPDLIPSAIEEHLRFISPIQNICRTTRVDYSVGQAVIPAGSLVLLAW

GAANRDPRQYEDPDVFRADRNPVGHLAFGSGIHLCPGTQLARMEGQAILR

EIVANIDRIEVVEPPTWTTNANLRGLTRLRVAVTPRVAP

>CYP128A1 (2694127190) Mycobacterium tuberculosis Q09600228

MTATQSPPEPAPDRVRLAGCPLAGTPDVGLTAQDATTALGVPTRRRASSG

GIPVATSMWRDAQTVRTYGPAVAKALALRVAGKARSRLTGRHCRKFMQLT

DFDPFDPAIAADPYPHYRELLAGERVQYNPKRDVYILSRYADVREAARNH

DTLSSARGVTFSRGWLPFLPTSDPPAHTRMRKQLAPGMARGALETWRPMV

DQLARELVGGLLTQTPADVVSTVAAPMPMRAITSVLGVDGPDEAAFCRLS

NQAVRITDVALSASGLISLVQGFAGFRRLRALFTHRRDNGLLRECTVLGK

LATHAEQGRLSDDELFFFAVLLLVAGYESTAHMISTLFLTLADYPDQLTL

LAQQPDLIPSAIEEHLRFISPIQNICRTTRVDYSVGQAVIPAGSLVLMAW

GAANRDPRQYEDPDVFRADRNPVGHLAFGSGIHLCPGPSWRAWRVRRSCA

RSSPISTE

>CYP128A1 (2560454601) Mycobacterium tuberculosis XDR1221

MTATQSPPEPAPDRVRLAGCPLAGTPDVGLTAQDATTALGVPTRRRASSG

GIPVATSMWRDAQTVRTYGPAVAKALALRVAGKARSRLTGRHCRKFMQLT

DFDPFDPAIAADPYPHYRELLAGERVQYNPKRDVYILSRYADVREAARNH

DTLSSARGVTFSRGWLPFLPTSDPPAHTRMRKQLAPGMARGALETWRPMV

DQLARELVGGLLTQTPADVVSTVAAPMPMRAITSVLGVDGPDEAAFCRLS

NQAVRITDVALSASGLISLVQGFAGFRRLRALFTHRRDNGLLRECTVLGK

LATHAEQGRLSDDELFFFAVLLLVAGYESTAHMISTLFLTLADYPDQLTL

LAQQPDLIPSAIEEHLRFISPIQNICRTTRVDYSVGQAVIPAGSLVLLAW

GAANRDPRQYEDPDVFRADRNPVGHLAFGSGIHLCPGTQLARMEGQAILR

EIVANIDRIEVVEPPTWTTNANLRGLTRLRVAVTPASHHEGSPFVVSTFP

VARVGRTAVGRLQLLGLDAAFDP

>CYP128A1 (2575654440) Mycobacterium tuberculosis 3280CJ

MTATQSPPEPAPDRVRLAGCPLAGTPDVGLTAQDATTALGVPTRRRASSG

GIPVATSMWRDAQTVRTYGPAVAKALALRVAGKARSRLTGRHCRKFMQLT

DFDPFDPAIAADPYPHYRELLAGERVQYNPKRDVYILSRYADVREAARNH

DTLSSARGVTFSRGWLPFLPTSDPPAHTRMRKQLAPGMARGALETWRPMV

DQLARELVGGLLTQTPADVVSTVAAPMPMRAITSVLGVDGPDEAAFCRLS

NQAVRITDVALSASGLISLVQGFAGFRRLRALFTHRRDNGLLRECTVLGK

LATHAEQGRLSDDELFFFAVLLLVAGYESTAHMISTLFLTLADYPDQLTL

LAQQPDLIPSAIEEHLRFISPIQNICRTTRVDYSVGQAVIPAGSLVLLAW

GAANRDPRQYEDPDVFRADRNPVGHLAFGSGIHLCPGTQLARMEGQAILR

EIVANIDRIEVVEPPTWTTNANLRGLTRLRVAVTPRVAP

>CYP128A1 (2575791731) Mycobacterium tuberculosis TKK_05SA_0048

MTATQSPPEPAPDRVRLAGCPLAGTPDVGLTAQDATTALGVPTRRRASSG

GIPVATSMWRDAQTVRTYGPAVAKALALRVAGKARSRLTGRHCRKFMQLT

DFDPFDPAIAADPYPHYRELLAGERVQYNPKRDVYILSRYADVREAARNH

DTLSSARGVTFSRGWLPFLPTSDPPAHTRMRKQLAPGMARGALETWRPMV

DQLARELVGGLLTQTPADVVSTVAAPMPMRAITSVLGVDGPDEAAFCRLS

NQAVRITDVALSASGLISLVQGFAGFRRLRALFTHRRDNGLLRECTVLGK

LATHAEQGRLSDDELFFFAVLLLVAGYESTAHMISTLFLTLADYPDQLTL

LAQQPDLIPSAIEEHLRFISPIQNICRTTRVDYSVGQAVIPAGSLVLLAW

GAANRDPRQYEDPDVFRADRNPVGHLAFGSGIHLCPGTQLARMEGQAILR

EIVANIDRIEVVEPPTWTTNANLRGLTRLRVAVTPRVAP

>CYP128A1 (2589625331) Mycobacterium tuberculosis TKK-01-0057

MTATQSPPEPAPDRVRLAGCPLAGTPDVGLTAQDATTALGVPTRRRASSG

GIPVATSMWRDAQTVRTYGPAVAKALALRVAGKARSRLTGRHCRKFMQLT

DFDPFDPAIAADPYPHYRELLAGERVQYNPKRDVYILSRYADVREAARNH

DTLSSARGVTFSRGWLPFLPTSDPPAHTRMRKQLAPGMARGALETWRPMV

DQLARELVGGLLTQTPADVVSTVAAPMPMRAITSVLGVDGPDEAAFCRLS

NQAVRITDVALSASGLISLVQGFAGFRRLRALFTHRRDNGLLRECTVLGK

LATHAEQGRLSDDELFFFAVLLLVAGYESTAHMISTLFLTLADYPDQLTL

LAQQPDLIPSAIEEHLRFISPIQNICRTTRVDYSVGQAVIPAGSLVLLAW

GAANRDPRQYEDPDVFRADRNPVGHLAFGSGIHLCPGTQLARMEGQAILR

EIVANIDRIEVVEPPTWTTNANLRGLTRLRVAVTPRVAP

>CYP128A1 (2592434654) Mycobacterium tuberculosis TKK_02_0006

MTATQSPPEPAPDRVRLAGCPLAGTPDVGLTAQDATTALGVPTRRRASSG

GIPVATSMWRDAQTVRTYGPAVAKALALRVAGKARSRLTGRHCRKFMQLT

DFDPFDPAIAADPYPHYRELLAGERVQYNPKRDVYILSRYADVREAARNH

DTLSSARGVTFSRGWLPFLPTSDPPAHTRMRKQLAPGMARGALETWRPMV

DQLARELVGGLLTQTPADVVSTVAAPMPMRAITSVLGVDGPDEAAFCRLS

NQAVRITDVALSASGLISLVQGFAGFRRLRALFTHRRDNGLLRECTVLGK

LATHAEQGRLSDDELFFFAVLLLVAGYESTAHMISTLFLTLADYPDQLTL

LAQQPDLIPSAIEEHLRFISPIQNICRTTRVDYSVGQAVIPAGSLVLLAW

GAANRDPRQYEDPDVFRADRNPVGHLAFGSGIHLCPGTQLARMEGQAILR

EIVANIDRIEVVEPPTWTTNANLRGLTRLRVAVTPRVAP

>CYP128A1 (2603366816) Mycobacterium tuberculosis BTB13-089

MTATQSPPEPAPDRVRLAGCPLAGTPDVGLTAQDATTALGVPTRRRASSG

GIPVATSMWRDAQTVRTYGPAVAKALALRVAGKARSRLTGRHCRKFMQLT

DFDPFDPAIAADPYPHYRELLAGERVQYNPKRDVYILSRYADVREAARNH

DTLSSARGVTFSRGWLPFLPTSDPPAHTRMRKQLAPGMARGALETWRPMV

DQLARELVGGLLTQTPADVVSTVAAPMPMRAITSVLGVDGPDEAAFCRLS

NQAVRITDVALSASGLISLVQGFAGFRRLRALFTHRRDNGLLRECTVLGK

LATHAEQGRLSDDELFFFAVLLLVAGYESTAHMISTLFLTLADYPDQLTL

LAQQPDLIPSAIEEHLRFISPIQNICRTTRVDYSVGQAVIPAGSLVLLAW

GAANRDPRQYEDPDVFRADRNPVGHLAFGSGIHLCPGTQLARMEGQAILR

EIVANIDRIEVVEPPTWTTNANLRGLTRLRVAVTPRVAP

>CYP128A1 (2605361149) Mycobacterium tuberculosis TB_RSA139

MTATQSPPEPAPDRVRLAGCPLAGTPDVGLTAQDATTALGVPTRRRASSG

GIPVATSMWRDAQTVRTYGPAVAKALALRVAGKARSRLTGRHCRKFMQLT

DFDPFDPAIAADPYPHYRELLAGERVQYNPKRDVYILSRYADVREAARNH

DTLSSARGVTFSRGWLPFLPTSDPPAHTRMRKQLAPGMARGALETWRPMV

DQLARELVGGLLTQTPADVVSTVAAPMPMRAITSVLGVDGPDEAAFCRLS

NQAVRITDVALSASGLISLVQGFAGFRRLRALFTHRRDNGLLRECTVLGK

LATHAEQGRLSDDELFFFAVLLLVAGYESTAHMISTLFLTLADYPDQLTL

LAQQPDLIPSAIEEHLRFISPIQNICRTTRVDYSVGQAVIPAGSLVLLAW

GAANRDPRQYEDPDVFRADRNPVGHLAFGSGIHLCPGTQLARMEGQAILR

EIVANIDRIEVVEPPTWTTNANLRGLTRLRVAVTPRVAP

>CYP128A1 (2605679983) Mycobacterium tuberculosis TRUG0024

MTATQSPPEPAPDRVRLAGCPLAGTPDVGLTAQDATTALGVPTRRRASSG

GIPVATSMWRDAQTVRTYGPAVAKALALRVAGKARSRLTGRHCRKFMQLT

DFDPFDPAIAADPYPHYRELLAGERVQYNPKRDVYILSRYADVREAARNH

DTLSSARGVTFSRGWLPFLPTSDPPAHTRMRKQLAPGMARGALETWRPMV

DQLARELVGGLLTQTPADVVSTVAAPMPMRAITSVLGVDGPDEAAFCRLS

NQAVRITDVALSASGLISLVQGFAGFRRLRALFTHRRDNGLLRECTVLGK

LATHAEQGRLSDDELFFFAVLLLVAGYESTAHMISTLFLTLADYPDQLTL

LAQQPDLIPSAIEEHLRFISPIQNICRTTRVDYSVGQAVIPAGSLVLLAW

GAANRDPRQYEDPDVFRADRNPVGHLAFGSGIHLCPGTQLARMEGQAILR

EIVANIDRIEVVEPPTWTTNANLRGLTRLRVAVTPRVAP

>CYP128A1 (2622553153) Mycobacterium tuberculosis M1431

MTATQSPPEPAPDRVRLAGCPLAGTPDVGLTAQDATTALGVPTRRRASSG

GIPVATSMWRDAQTVRTYGPAVAKALALRVAGKARSRLTGRHCRKFMQLT

DFDPFDPAIAADPYPHYRELLAGERVQYNPKRDVYILSRYADVREAARNH

DTLSSARGVTFSRGWLPFLPTSDPPAHTRMRKQLAPGMARGALETWRPMV

DQLARELVGGLLTQTPADVVSTVAAPMPMRAITSVLGVDGPDEAAFCRLS

NQAVRITDVALSASGLISLVQGFAGFRRLRALFTHRRDNGLLRECTVLGK

LATHAEQGRLSDDELFFFAVLLLVAGYESTAHMISTLFLTLADYPDQLTL

LAQQPDLIPSAIEEHLRFISPIQNICRTTRVDYSVGQAVIPAGSLVLLAW

GAANRDPRQYEDPDVFRADRNPVGHLAFGSGIHLCPGTQLARMEGQAILR

EIVANIDRIEVVEPPTWTTNANLRGLTRLRVAVTPRVAP

>CYP128A1 (2630865639) Mycobacterium tuberculosis TKK_04_0113

MTATQSPPEPAPDRVRLAGCPLAGTPDVGLTAQDATTALGVPTRRRASSG

GIPVATSMWRDAQTVRTYGPAVAKALALRVAGKARSRLTGRHCRKFMQLT

DFDPFDPAIAADPYPHYRELLAGERVQYNPKRDVYILSRYADVREAARNH

DTLSSARGVTFSRGWLPFLPTSDPPAHTRMRKQLAPGMARGALETWRPMV

DQLARELVGGLLTQTPADVVSTVAAPMPMRAITSVLGVDGPDEAAFCRLS

NQAVRITDVALSASGLISLVQGFAGFRRLRALFTHRRDNGLLRECTVLGK

LATHAEQGRLSDDELFFFAVLLLVAGYESTAHMISTLFLTLADYPDQLTL

LAQQPDLIPSAIEEHLRFISPIQNICRTTRVDYSVGQAVIPAGSLVLLAW

GAANRDPRQYEDPDVFRADRNPVGHLAFGSGIHLCPGTQLARMEGQAILR

EIVANIDRIEVVEPPTWTTNANLRGLTRLRVAVTPRVAP

>CYP128A1 (2648997100) Mycobacterium tuberculosis Manu ancestor SIT523

MTATQSPPEPAPDRVRLAGCPLAGTPDVGLTAQDATTALGVPTRRRASSG

GIPVATSMWRDAQTVRTYGPAVAKALALRVAGKARSRLTGRHCRKFMQLT

DFDPFDPAIAADPYPHYRELLAGERVQYNPKRDVYILSRYADVREAARNH

DTLSSARGVTFSRGWLPFLPTSDPPAHTRMRKQLAPGMARGALETWRPMV

DQLARELVGGLLTQTPADVVSTVAAPMPMRAITSVLGVDGPDEAAFCRLS

NQAVRITDVALSASGLISLVQGFAGFRRLRALFTHRRDNGLLRECTVLGK

LATHAEQGRLSDDELFFFAVLLLVAGYESTAHMISTLFLTLADYPDQLTL

LAQQPDLIPSAIEEHLRFISPIQNICRTTRVDYSVGQAVIPAGSLVLLAW

GAANRDPRQYEDPDVFRADRNPVGHLAFGSGIHLCPGTQLARMEGQAILR

EIVANIDRIEVVEPPTWTTNANLRGLTRLRVAVTPRVAP

>CYP128A1 (2667082838) Mycobacterium tuberculosis 1700089

MTATQSPPEPAPDRVRLAGCPLAGTPDVGLTAQDATTALGVPTRRRASSG

GIPVATSMWRDAQTVRTYGPAVAKALALRVAGKARSRLTGRHCRKFMQLT

DFDPFDPAIAADPYPHYRELLAGERVQYNPKRDVYILSRYADVREAARNH

DTLSSARGVTFSRGWLPFLPTSDPPAHTRMRKQLAPGMARGALETWRPMV

DQLARELVGGLLTQTPADVVSTVAAPMPMRAITSVLGVDGPDEAAFCRLS

NQAVRITDVALSASGLISLVQGFAGFRRLRALFTHRRDNGLLRECTVLGK

LATHAEQGRLSDDELFFFAVLLLVAGYESTAHMISTLFLTLADYPDQLTL

LAQQPDLIPSAIEEHLRFISPIQNICRTTRVDYSVGQAVIPAGSLVLLAW

GAANRDPRQYEDPDVFRADRNPVGHLAFGSGIHLCPGTQLARMEGQAILR

EIVANIDRIEVVEPPTWTTNANLRGLTRLRVAVTPRVAP

>CYP128A1 (2720702678) Mycobacterium tuberculosis I0004000-1

MTATQSPPEPAPDRVRLAGCPLAGTPDVGLTAQDATTALGVPTRRRASSG

GIPVATSMWRDAQTVRTYGPAVAKALALRVAGKARSRLTGRHCRKFMQLT

DFDPFDPAIAADPYPHYRELLAGERVQYNPKRDVYILSRYADVREAARNH

DTLSSARGVTFSRGWLPFLPTSDPPAHTRMRKQLAPGMARGALETWRPMV

DQLARELVGGLLTQTPADVVSTVAAPMPMRAITSVLGVDGPDEAAFCRLS

NQAVRITDVALSASGLISLVQGFAGFRRLRALFTHRRDNGLLRECTVLGK

LATHAEQGRLSDDELFFFAVLLLVAGYESTAHMISTLFLTLADYPDQLTL

LAQQPDLIPSAIEEHLRFISPIQNICRTTRVDYSVGQAVIPAGSLVLLAW

GAANRDPRQYEDPDVFRADRNPVGHLAFGSGIHLCPGTQLARMEGQAILR

EIVANIDRIEVVEPPTWTTNANLRGLTRLRVAVTPRVAP

>CYP128B4 (2809727232) Mycobacterium sp. 1081908.1

MTLTDGVRFTGSAARTLAGAVLANAAGARRRSPVDADITAYDPLDAATAA

QPHEAYRRLHAGARVQYSPKRRVFILSRLDDVRAAARDDANLSSADGPML

TRIRTPILVSLDGEEHARQRRQVLPAFTRAALDSWRPIIDRLAAETVHDV

LANPGCDVMQRLAIPMPVRLIAQLLGVPDTDVDDFRRWSEASVQITDVDL

SARGVRKLAGSMFGSVAMHRYFTRQFAAGGLKGSDTILGRLLAENEAGSL

PDRELFYFAMLLLLAGNETTTNLLGGMFATLADHPEQYGLLRAGPDLVPM

AVEELLRYLSPAQNVYRTALREYPVGDTTIPAGARIMLSIGAANRDPRAF

DQPDAFRVDRNPTQHMTFGFGAHLCIGAQLTRMEAQAVLRELVTRVDRIS

VVGAPQWSTNSLLRGPTRLAVRLTPA

>CYP128B4 (2796322064) Mycobacteroides abscessus abscessus 581

MSRWRSDSAVRQIGAPVVAAVGMNIAAAVRVRRRGYAGWTGAVNTDYDPL

DPATAAQPFDAYRALHAGGRVHYNPKRATFILSRHEDIRAALRDTDAVTS

SQGVTRMKISAPILVLTDGDDHTRLRKQVQPGFTRGAMSDWQGMADQLAK

ELVADVVANPGCDVMERLAVPLPIRMIAHIIGIPPEDVQNFRSWSEDGVG

VINAGVSPAGLRQGLKGVRAIAALRRYFKDQLASGKLKGSDTVLGRLVDN

NEDGKLSDDELFFIAMLLLFAGNETTTNLIGGMFDTLAHAPDQFAMIRDD

PDLIPSAVEEQLRYSAPIQNLYRYTRTDYRVGEVTIPSGSRLLLAFGAAN

RDPEVFEDPDTYRADRNPRNHIAFGYGVHMCIGATLSRMEGQAVLRELTS

QASAIAAAGSATWSTNSSLRGTTYLPIRLTPAR

>CYP128B4 (2797111184) Mycobacteroides abscessus abscessus 646

MSRWRSDSAVRQIGAPVVAAVGMNIAAAVRVRRRGYAGWTGAVNTDYDPL

DPATAAQPFDAYRALHAGGRVHYNPKRATFILSRHEDIRAALRDTDAVTS

SQGVTRMKISAPILVLTDGDDHTRLRKQVQPGFTRGAMSDWQGMADQLAK

ELVADVVANPGCDVMERLAVPLPIRMIAHIIGIPPEDVQNFRSWSEDGVG

VINAGVSPAGLRQGLKGVRAIAALRRYFKDQLASGKLKGSDTVLGRLVDN

NEDGKLSDDELFFIAMLLLFAGNETTTNLIGGMFDTLAHAPDQFAMIRDD

PDLIPSAVEEQLRYSAPIQNLYRYTRTDYRVGEVTIPSGSRLLLAFGAAN

RDPEVFEDPDTYRADRNPRNHIAFGYGVHMCIGATLSRMEGQAVLRELTS

QASAIAAAGSATWSTNSSLRGTTYLPIRLTPAR

>CYP128B1 (2809758932) Mycobacterium scrofulaceum E2838

VSIAETAQKATESLRPVAGLVRMNVAGAVRTRRRGYNGWIGAINTDYDPQ

DPATAADPFDAYRALHRSGRVHYNPRRATFILSRLDDVRAALRDTDQVTS

TQGVTRLRMSGSLAVLTDGEEHTRLRKQVQPGFSTGAMKSWQEMTEKLAT

ELVTDVLNDPGCDVVQRLAIPMPIRLIAQILGVPDTDVGDFRRWSERAVG

VMELKPTLSGVVDAAKSMSAMVALQRYFTSQFATGGLKGSSTVLGRLVEH

NTDGSLTDSQLLLIAIHLLIAGNETTTNLLGGMFDTLARRPEQYDLIRAN

PDLIPLAVEEQLRFTTPIQNLYRYTRADYRIGDVTIPTGSRVLLSFGAAN

RDPAAFDDPDEYRADRNPRMHVAFGYGPHMCLGAPLARMEAQAILRQLVT

RVARITPAGPTRWSGHSSLRGPTRLPIRLSPA

>CYP128B1 (2810025222) Mycobacterium sp. 852014-52450_SCH5900713

VSITETAQKATKTLRPVAGLVRMNVAAAVRTRRRGYNGWTGAINTDYDPQ

DPATAADPFDAYRTLHRSGRVHYNPRRAAFIISRLDDVRAALRDTEQVTS

TQGVTRLRMSGSLAVLTDGEEHTRLRKQVQPGFSKGAMKSWQEMTEKLAI

ELVTDVLNDQGCDVVQRLAIPMPIRLIAQILGVPDTDVGDFRRWSERAVG

VMELKPTLSGVVDAAKSILAMVALQRYFTSQFATGGLKGSNTVLGRLIEH

NNDGSLTDTQLLLIAIHLLIAGNETTTNLLGGMFDTLARHPDQYDLIRNN

PDLIPLAVEEQLRVTTPIQNLYRYTRADYRIGDVTIPTGSRVLLSFGAAN

RDPTAFDDPDEYRADRDPRAHVAFGYGAHMCLGAPLARMEAQAVLRQLVT

RVARITPAGPTQWSSHSSLRGPTRLPIRLTPA

>CYP128A1 (2555422519) Mycobacterium tuberculosis PanR0908

MTATQSPPEPAPDRVRLAGCPLAGTPDVGLTAQDATTALGVPTRRRASSG

GIPVATSMWRDAQTVRTYGPAVAKALALRVAGKARSRLTGRHCRKFMQLT

DFDPFDPAIAADPYPHYRELLAGERVQYNPKRDVYILSRYADVREAARNH

DTLSSARGVTFSRGWLPFLPTSDPPAHTRMRKQLAPGMARGALETWRPMV

DQLARELVGGLLTQTPADVVSTVAAPMPMRAITSVLGVDGPDEAAFCRLS

NQAVRITDVALSASGLISLVQGFAGFRRLRALFTHRRDNGLLRECTVLGK

LATHAEQGRLSDDELFFFAVLLLVAGYESTAHMISTLFLTLADYPDQLTL

LAQQPDLIPSAIEEHLRFISPIQNICRTTRVDYSVGQAVIPAGSLVLLAW

GAANRDPRQYEDPDVFRADRNPVGHLAFGSGIHLCPGTQLARMEGQAILR

EIVANIDRIEVVEPPTWTTNANLRGLTRLRVAVTPRVAP

>CYP128A1 (2575385682) Mycobacterium tuberculosis NRITLD57

MTATQSPPEPAPDRVRLAGCPLAGTPDVGLTAQDATTALGVPTRRRASSG

GIPVATSMWRDAQTVRTYGPAVAKALALRVAGKARSRLTGRHCRKFMQLT

DFDPFDPAIAADPYPHYRELLAGERVQYNPKRDVYILSRYADVREAARNH

DTLSSARGVTFSRGWLPFLPTSDPPAHTRMRKQLAPGMARGALETWRPMV

DQLARELVGGLLTQTPADVVSTVAAPMPMRAITSVLGVDGPDEAAFCRLS

NQAVRITDVALSASGLISLVQGFAGFRRLRALFTHRRDNGLLRECTVLGK

LATHAEQGRLSDDELFFFAVLLLVAGYESTAHMISTLFLTLADYPDQLTL

LAQQPDLIPSAIEEHLRFISPIQNICRTTRVDYSVGQAVIPAGSLVLLAW

GAANRDPRQYEDPDVFRADRNPVGHLAFGSGIHLCPGTQLARMEGQAILR

EIVANIDRIEVVEPPTWTTNANLRGLTRLRVAVTPRVAP

>CYP128A1 (2590527040) Mycobacterium tuberculosis KT-0085

MTATQSPPEPAPDRVRLAGCPLAGTPDVGLTAQDATTALGVPTRRRASSG

GIPVATSMWRDAQTVRTYGPAVAKALALRVAGKARSRLTGRHCRKFMQLT

DFDPFDPAIAADPYPHYRELLAGERVQYNPKRDVYILSRYADVREAARNH

DTLSSARGVTFSRGWLPFLPTSDPPAHTRMRKQLAPGMARGALETWRPMV

DQLARELVGGLLTQTPADVVSTVAAPMPMRAITSVLGVDGPDEAAFCRLS

NQAVRITDVALSASGLISLVQGFAGFRRLRALFTHRRDNGLLRECTVLGK

LATHAEQGRLSDDELFFFAVLLLVAGYESTAHMISTLFLTLADYPDQLTL

LAQQPDLIPSAIEEHLRFISPIQNICRTTRVDYSVGQAVIPAGSLVLLAW

GAANRDPRQYEDPDVFRADRNPVGHLAFGSGIHLCPGTQLARMEGQAILR

EIVANIDRIEVVEPPTWTTNANLRGLTRLRVAVTPRVAP

>CYP128A1 (2590551662) Mycobacterium tuberculosis KT-0077

MTATQSPPEPAPDRVRLAGCPLAGTPDVGLTAQDATTALGVPTRRRASSG

GIPVATSMWRDAQTVRTYGPAVAKALALRVAGKARSRLTGRHCRKFMQLT

DFDPFDPAIAADPYPHYRELLAGERVQYNPKRDVYILSRYADVREAARNH

DTLSSARGVTFSRGWLPFLPTSDPPAHTRMRKQLAPGMARGALETWRPMV

DQLARELVGGLLTQTPADVVSTVAAPMPMRAITSVLGVDGPDEAAFCRLS

NQAVRITDVALSASGLISLVQGFAGFRRLRALFTHRRDNGLLRECTVLGK

LATHAEQGRLSDDELFFFAVLLLVAGYESTAHMISTLFLTLADYPDQLTL

LAQQPDLIPSAIEEHLRFISPIQNICRTTRVDYSVGQAVIPAGSLVLLAW

GAANRDPRQYEDPDVFRADRNPVGHLAFGSGIHLCPGTQLARMEGQAILR

EIVANIDRIEVVEPPTWTTNANLRGLTRLRVAVTPRVAP

>CYP128A1 (2604953087) Mycobacterium tuberculosis BTB09-184

MTATQSPPEPAPDRVRLAGCPLAGTPDVGLTAQDATTALGVPTRRRASSG

GIPVATSMWRDAQTVRTYGPAVAKALALRVAGKARSRLTGRHCRKFMQLT

DFDPFDPAIAADPYPHYRELLAGERVQYNPKRDVYILSRYADVREAARNH

DTLSSARGVTFSRGWLPFLPTSDPPAHTRMRKQLAPGMARGALETWRPMV

DQLARELVGGLLTQTPADVVSTVAAPMPMRAITSVLGVDGPDEAAFCRLS

NQAVRITDVALSASGLISLVQGFAGFRRLRALFTHRRDNGLLRECTVLGK

LATHAEQGRLSDDELFFFAVLLLVAGYESTAHMISTLFLTLADYPDQLTL

LAQQPDLIPSAIEEHLRFISPIQNICRTTRVDYSVGQAVIPAGSLVLLAW

GAANRDPRQYEDPDVFRADRNPVGHLAFGSGIHLCPGTQLARMEGQAILR

EIVANIDRIEVVEPPTWTTNANLRGLTRLRVAVTPRVAP

>CYP128A1 (2621350164) Mycobacterium tuberculosis INS_MDR

MTATQSPPEPAPDRVRLAGCPLAGTPDVGLTAQDATTALGVPTRRRASSG

GIPVATSMWRDAQTVRTYGPAVAKALALRVAGKARSRLTGRHCRKFMQLT

DFDPFDPAIAADPYPHYRELLAGERVQYNPKRDVYILSRYADVREAARNH

DTLSSARGVTFSRGWLPFLPTSDPPAHTRMRKQLAPGMARGALETWRPMV

DQLARELVGGLLTQTPADVVSTVAAPMPMRAITSVLGVDGPDEAAFCRLS

NQAVRITDVALSASGLISLVQGFAGFRRLRALFTHRRDNGLLRECTVLGK

LATHAEQGRLSDDELFFFAVLLLVAGYESTAHMISTLFLTLADYPDQLTL

LAQQPDLIPSAIEEHLRFISPIQNICRTTRVDYSVGQAVIPAGSLVLLAW

GAANRDPRQYEDPDVFRADRNPVGHLAFGSGIHLCPGTQLARMEGQAILR

EIVANIDRIEVVEPPTWTTNANLRGLTRLRVAVTPRVAP

>CYP128A1 (2622006810) Mycobacterium tuberculosis MD17904

MTATQSPPEPAPDRVRLAGCPLAGTPDVGLTAQDATTALGVPTRRRASSG

GIPVATSMWRDAQTVRTYGPAVAKALALRVAGKARSRLTGRHCRKFMQLT

DFDPFDPAIAADPYPHYRELLAGERVQYNPKRDVYILSRYADVREAARNH

DTLSSARGVTFSRGWLPFLPTSDPPAHTRMRKQLAPGMARGALETWRPMV

DQLARELVGGLLTQTPADVVSTVAAPMPMRAITSVLGVDGPDEAAFCRLS

NQAVRITDVALSASGLISLVQGFAGFRRLRALFTHRRDNGLLRECTVLGK

LATHAEQGRLSDDELFFFAVLLLVAGYESTAHMISTLFLTLADYPDQLTL

LAQQPDLIPSAIEEHLRFISPIQNICRTTRVDYSVGQAVIPAGSLVLLAW

GAANRDPRQYEDPDVFRADRNPVGHLAFGSGIHLCPGTQLARMEGQAILR

EIVANIDRIEVVEPPTWTTNANLRGLTRLRVAVTPRVAP

>CYP128A1 (2622115749) Mycobacterium tuberculosis MD17647

MTATQSPPEPAPDRVRLAGCPLAGTPDVGLTAQDATTALGVPTRRRASSG

GIPVATSMWRDAQTVRTYGPAVAKALALRVAGKARSRLTGRHCRKFMQLT

DFDPFDPAIAADPYPHYRELLAGERVQYNPKRDVYILSRYADVREAARNH

DTLSSARGVTFSRGWLPFLPTSDPPAHTRMRKQLAPGMARGALETWRPMV

DQLARELVGGLLTQTPADVVSTVAAPMPMRAITSVLGVDGPDEAAFCRLS

NQAVRITDVALSASGLISLVQGFAGFRRLRALFTHRRDNGLLRECTVLGK

LATHAEQGRLSDDELFFFAVLLLVAGYESTAHMISTLFLTLADYPDQLTL

LAQQPDLIPSAIEEHLRFISPIQNICRTTRVDYSVGQAVIPAGSLVLLAW

GAANRDPRQYEDPDVFRADRNPVGHLAFGSGIHLCPGTQLARMEGQAILR

EIVANIDRIEVVEPPTWTTNANLRGLTRLRVAVTPRVAP

>CYP128A1 (2622437918) Mycobacterium tuberculosis M1295

MTATQSPPEPAPDRVRLAGCPLAGTPDVGLTAQDATTALGVPTRRRASSG

GIPVATSMWRDAQTVRTYGPAVAKALALRVAGKARSRLTGRHCRKFMQLT

DFDPFDPAIAADPYPHYRELLAGERVQYNPKRDVYILSRYADVREAARNH

DTLSSARGVTFSRGWLPFLPTSDPPAHTRMRKQLAPGMARGALETWRPMV

DQLARELVGGLLTQTPADVVSTVAAPMPMRAITSVLGVDGPDEAAFCRLS

NQAVRITDVALSASGLISLVQGFAGFRRLRALFTHRRDNGLLRECTVLGK

LATHAEQGRLSDDELFFFAVLLLVAGYESTAHMISTLFLTLADYPDQLTL

LAQQPDLIPSAIEEHLRFISPIQNICRTTRVDYSVGQAVIPAGSLVLLAW

GAANRDPRQYEDPDVFRADRNPVGHLAFGSGIHLCPGTQLARMEGQAILR

EIVANIDRIEVVEPPTWTTNANLRGLTRLRVAVTPRVAP

>CYP128A1 (2632132946) Mycobacterium tuberculosis TKK_05MA_0037

MTATQSPPEPAPDRVRLAGCPLAGTPDVGLTAQDATTALGVPTRRRASSG

GIPVATSMWRDAQTVRTYGPAVAKALALRVAGKARSRLTGRHCRKFMQLT

DFDPFDPAIAADPYPHYRELLAGERVQYNPKRDVYILSRYADVREAARNH

DTLSSARGVTFSRGWLPFLPTSDPPAHTRMRKQLAPGMARGALETWRPMV

DQLARELVGGLLTQTPADVVSTVAAPMPMRAITSVLGVDGPDEAAFCRLS

NQAVRITDVALSASGLISLVQGFAGFRRLRALFTHRRDNGLLRECTVLGK

LATHAEQGRLSDDELFFFAVLLLVAGYESTAHMISTLFLTLADYPDQLTL

LAQQPDLIPSAIEEHLRFISPIQNICRTTRVDYSVGQAVIPAGSLVLLAW

GAANRDPRQYEDPDVFRADRNPVGHLAFGSGIHLCPGTQLARMEGQAILR

EIVANIDRIEVVEPPTWTTNANLRGLTRLRVAVTPRVAP

>CYP128A1 (2638161499) Mycobacterium tuberculosis KT-0188

MTATQSPPEPAPDRVRLAGCPLAGTPDVGLTAQDATTALGVPTRRRASSG

GIPVATSMWRDAQTVRTYGPAVAKALALRVAGKARSRLTGRHCRKFMQLT

DFDPFDPAIAADPYPHYRELLAGERVQYNPKRDVYILSRYADVREAARNH

DTLSSARGVTFSRGWLPFLPTSDPPAHTRMRKQLAPGMARGALETWRPMV

DQLARELVGGLLTQTPADVVSTVAAPMPMRAITSVLGVDGPDEAAFCRLS

NQAVRITDVALSASGLISLVQGFAGFRRLRALFTHRRDNGLLRECTVLGK

LATHAEQGRLSDDELFFFAVLLLVAGYESTAHMISTLFLTLADYPDQLTL

LAQQPDLIPSAIEEHLRFISPIQNICRTTRVDYSVGQAVIPAGSLVLLAW

GAANRDPRQYEDPDVFRADRNPVGHLAFGSGIHLCPGTQLARMEGQAILR

EIVANIDRIEVVEPPTWTTNANLRGLTRLRVAVTPRVAP

>CYP128A1 (2686590682) Mycobacterium tuberculosis 2279

MTATQSPPEPAPDRVRLAGCPLAGTPDVGLTAQDATTALGVPTRRRASSG

GIPVATSMWRDAQTVRTYGPAVAKALALRVAGKARSRLTGRHCRKFMQLT

DFDPFDPAIAADPYPHYRELLAGERVQYNPKRDVYILSRYADVREAARNH

DTLSSARGVTFSRGWLPFLPTSDPPAHTRMRKQLAPGMARGALETWRPMV

DQLARELVGGLLTQTPADVVSTVAAPMPMRAITSVLGVDGPDEAAFCRLS

NQAVRITDVALSASGLISLVQGFAGFRRLRALFTHRRDNGLLRECTVLGK

LATHAEQGRLSDDELFFFAVLLLVAGYESTAHMISTLFLTLADYPDQLTL

LAQQPDLIPSAIEEHLRFISPIQNICRTTRVDYSVGQAVIPAGSLVLLAW

GAANRDPRQYEDPDVFRADRNPVGHLAFGSGIHLCPGTQLARMEGQAILR

EIVANIDRIEVVEPPTWTTNANLRGLTRLRVAVTPRVAP

>CYP128A1 (2703502356) Mycobacterium tuberculosis J09800745

MTATQSPPEPAPDRVRLAGCPLAGTPDVGLTAQDATTALGVPTRRRASSG

GIPVATSMWRDAQTVRTYGPAVAKALALRVAGKARSRLTGRHCRKFMQLT

DFDPFDPAIAADPYPHYRELLAGERVQYNPKRDVYILSRYADVREAARNH

DTLSSARGVTFSRGWLPFLPTSDPPAHTRMRKQLAPGMARGALETWRPMV

DQLARELVGGLLTQTPADVVSTVAAPMPMRAITSVLGVDGPDEAAFCRLS

NQAVRITDVALSASGLISLVQGFAGFRRLRALFTHRRDNGLLRECTVLGK

LATHAEQGRLSDDELFFFAVLLLVAGYESTAHMISTLFLTLADYPDQLTL

LAQQPDLIPSAIEEHLRFISPIQNICRTTRVDYSVGQAVIPAGSLVLLAW

GAANRDPRQYEDPDVFRADRNPVGHLAFGSGIHLCPGPSWRAWRVRRSCA

RSSPISTE

>CYP128A1 (2706477835) Mycobacterium tuberculosis I00601066

MTATQSPPEPAPDRVRLAGCPLAGTPDVGLTAQDATTALGVPTRRRASSG

GIPVATSMWRDAQTVRTYGPAVAKALALRVAGKARSRLTGRHCRKFMQLT

DFDPFDPAIAADPYPHYRELLAGERVQYNPKRDVYILSRYADVREAARNH

DTLSSARGVTFSRGWLPFLPTSDPPAHTRMRKQLAPGMARGALETWRPMV

DQLARELVGGLLTQTPADVVSTVAAPMPMRAITSVLGVDGPDEAAFCRLS

NQAVRITDVALSASGLISLVQGFAGFRRLRALFTHRRDNGLLRECTVLGK

LATHAEQGRLSDDELFFFAVLLLVAGYESTAHMISTLFLTLADYPDQLTL

LAQQPDLIPSAIEEHLRFISPIQNICRTTRVDYSVGQAVIPAGSLVLLAW

GAANRDPRQYEDPDVFRADRNPVGHLAFGSGIHLCPGPSWRAWRVRRSCA

RSSPISTE

>CYP128A1 (2716299353) Mycobacterium tuberculosis H6_7420

MTATQSPPEPAPDRVRLAGCPLAGTPDVGLTAQDATTALGVPTRRRASSG

GIPVATSMWRDAQTVRTYGPAVAKALALRVAGKARSRLTGRHCRKFMQLT

DFDPFDPAIAADPYPHYRELLAGERVQYNPKRDVYILSRYADVREAARNH

DTLSSARGVTFSRGWLPFLPTSDPPAHTRMRKQLAPGMARGALETWRPMV

DQLARELVGGLLTQTPADVVSTVAAPMPMRAITSVLGVDGPDEAAFCRLS

NQAVRITDVALSASGLISLVQGFAGFRRLRALFTHRRDNGLLRECTVLGK

LATHAEQGRLSDDELFFFAVLLLVAGYESTAHMISTLFLTLADYPDQLTL

LAQQPDLIPSAIEEHLRFISPIQNICRTTRVDYSVGQAVIPAGSLVLLAW

GAANRDPRQYEDPDVFRADRNPVGHLAFGSGIHLCPGTQLARMEGQAILR

EIVANIDRIEVVEPPTWTTNANLRGLTRLRVAVTPRVAP

>CYP128A1 (2730275417) Mycobacterium tuberculosis 0A033DS

MTATQSPPEPAPDRVRLAGCPLAGTPDVGLTAQDATTALGVPTRRRASSG

GIPVATSMWRDAQTVRTYGPAVAKALALRVAGKARSRLTGRHCRKFMQLT

DFDPFDPAIAADPYPHYRELLAGERVQYNPKRDVYILSRYADVREAARNH

DTLSSARGVTFSRGWLPFLPTSDPPAHTRMRKQLAPGMARGALETWRPMV

DQLARELVGGLLTQTPADVVSTVAAPMPMRAITSVLGVDGPDEAAFCRLS

NQAVRITDVALSASGLISLVQGFAGFRRLRALFTHRRDNGLLRECTVLGK

LATHAEQGRLSDDELFFFAVLLLVAGYESTAHMISTLFLTLADYPDQLTL

LAQQPDLIPSAIEEHLRFISPIQNICRTTRVDYSVGQAVIPAGSLVLLAW

GAANRDPRQYEDPDVFRADRNPVGHLAFGSGIHLCPGTQLARMEGQAILR

EIVANIDRIEVVEPPTWTTNANLRGLTRLRVAVTPRVAP

>CYP128A1 (2511811874) Mycobacterium bovis BCG Mexico

MTATQSPPEPAPDRVRLAGCPLAGTPDVGLTAQDATTALGVPTRRRASSG

GIPVATSMWRDAQTVRTYGPAVAKALALRVAGKARSRLTGRHCRKFMQLT

DFDPFDPAIAADPYPHYRELLAGERVQYNPKRDVYILSRYADVREAARNH

DTLSSARGVTFSRGWLPFLPTSDPPAHTRMRKQLAPGMARGALETWRPMV

DQFARELVGGLLTQTPADVVSTVAAPMPMRAITSVLGVDGPDEAAFCRLS

NQAVRITDVALSASGLISLVQGFAGFRRLRALFTHRRDNGLLRECTVLGK

LATHAEQGRLSDDELFFFAVLLLVAGYESTAHMISTLFLTLADYPDQLTL

LAQQPDLIPSAIEEHLRFISPIQNICRTTRVDYSVGQAVIPAGSLVLLAW

GAANRDPRQYEDPDVFRADRNPVGHLAFGSGIHLCPGTQLARMEGQAILR

EIVANIDRIEVVEPPTWTTNANLRGLTRLRVAVTPRVAP

>CYP128A1 (2600675250) Mycobacterium bovis BCG Prague

MTATQSPPEPAPDRVRLAGCPLAGTPDVGLTAQDATTALGVPTRRRASSG

GIPVATSMWRDAQTVRTYGPAVAKALALRVAGKARSRLTGRHCRKFMQLT

DFDPFDPAIAADPYPHYRELLAGERVQYNPKRDVYILSRYADVREAARNH

DTLSSARGVTFSRGWLPFLPTSDPPAHTRMRKQLAPGMARGALETWRPMV

DQFARELVGGLLTQTPADVVSTVAAPMPMRAITSVLGVDGPDEAAFCRLS

NQAVRITDVALSASGLISLVQGFAGFRRLRALFTHRRDNGLLRECTVLGK

LATHAEQGRLSDDELFFFAVLLLVAGYESTAHMISTLFLTLADYPDQLTL

LAQQPDLIPSAIEEHLRFISPIQNICRTTRVDYSVGQAVIPAGSLVLLAW

GAANRDPRQYEDPDVFRADRNPVGHLAFGSGIHLCPGTQLARMEGQAILR

EIVANIDRIEVVEPPTWTTNANLRGLTRLRVAVTPRVAP

>CYP128B4 (2802021932) Mycobacteroides abscessus abscessus 984

MSRWRSDSAVRQIGAPVVAAVGMNIAAAVRVRRRGYAGWTGAVNTDYDPL

DPATAAQPFDAYRALHAGGRVHYNPKRATFILSRHEDIRAALRDTDAVTS

SQGVTRMKISAPILVLTDGDDHTRLRKQVQPGFTRGAMSDWQGMADQLAK

ELVADVVANPGCDVMERLAVPLPIRMIAHIIGIPPEDVQNFRSWSEDGVG

VINAGVSPAGLRQGLKGVRAIAALRRYFKDQLASGKLKGSDTVLGRLVDN

NEDGKLSDDELFFIAMLLLFAGNETTTNLIGGMFDTLAHAPDQFAMIRDD

PDLIPSAVEEQLRYSAPIQNLYRYTRTDYRVGEVTIPSGSRLLLAFGAAN

RDPEVFEDPDTYRADRNPRNHIAFGYGVHMCIGATLSRMEGQAVLRELTS

QASAIAAAGSATWSTNSSLRGTTYLPIRLTPAR

>CYP128A1 (2574605708) Mycobacterium tuberculosis TKK-01-0085

MTATQSPPEPAPDRVRLAGCPLAGTPDVGLTAQDATTALGVPTRRRASSG

GIPVATSMWRDAQTVRTYGPAVAKALALRVAGKARSRLTGRHCRKFMQLT

DFDPFDPAIAADPYPHYRELLAGERVQYNPKRDVYILSRYADVREAARNH

DTLSSARGVTFSRGWLPFLPTSDPPAHTRMRKQLAPGMARGALETWRPMV

DQLARELVGGLLTQTPADVVSTVAAPMPMRAITSVLGVDGPDEAAFCRLS

NQAVRITDVALSASGLISLVQGFAGFRRLRALFTHRRDNGLLRECTVLGK

LATHAEQGRLSDDELFFFAVLLLVAGYESTAHMISTLFLTLADYPDQLTL

LAQQPDLIPSAIEEHLRFISPIQNICRTTRVDYSVGQAVIPAGSLVLLAW

GAANRDPRQYEDPDVFRADRNPVGHLAFGSGIHLCPGTQLARMEGQAILR

EIVANIDRIEVVEPPTWTTNANLRGLTRLRVAVTPRVAP

>CYP128A1 (2580124457) Mycobacterium bovis Kc 9614

MTATQSPPEPAPDRVRLAGCPLAGTPDVGLTAQDATTALGVPTRRRASSG

GIPVATSMWRDAQTVRTYGPAVAKALALRVAGKARSRLTGRHCRKFMQLT

DFDPFDPAIAADPYPHYRELLAGERVQYNPKRDVYILSRYADVREAARNH

DTLSSARGVTFSRGWLPFLPTSDPPAHTRMRKQLAPGMARGALETWRPMV

DQLARELVGGLLTQTPADVVSTVAAPMPMRAITSVLGVDGPDEAAFCRLS

NQAVRITDVALSASGLISLVQGFAGFRRLRALFTHRRDNGLLRECTVLGK

LATHAEQGRLSDDELFFFAVLLLVAGYESTAHMISTLFLTLADYPDQLTL

LAQQPDLIPSAIEEHLRFISPIQNICRTTRVDYSVGQAVIPAGSLVLLAW

GAANRDPRQYEDPDVFRADRNPVGHLAFGSGIHLCPGTQLARMEGQAILR

EIVANIDRIEVVEPPTWTTNANLRGLTRLRVAVTPRVAP

>CYP128A1 (2588979017) Mycobacterium tuberculosis TKK-01-0017

MTATQSPPEPAPDRVRLAGCPLAGTPDVGLTAQDATTALGVPTRRRASSG

GIPVATSMWRDAQTVRTYGPAVAKALALRVAGKARSRLTGRHCRKFMQLT

DFDPFDPAIAADPYPHYRELLAGERVQYNPKRDVYILSRYADVREAARNH

DTLSSARGVTFSRGWLPFLPTSDPPAHTRMRKQLAPGMARGALETWRPMV

DQLARELVGGLLTQTPADVVSTVAAPMPMRAITSVLGVDGPDEAAFCRLS

NQAVRITDVALSASGLISLVQGFAGFRRLRALFTHRRDNGLLRECTVLGK

LATHAEQGRLSDDELFFFAVLLLVAGYESTAHMISTLFLTLADYPDQLTL

LAQQPDLIPSAIEEHLRFISPIQNICRTTRVDYSVGQAVIPAGSLVLLAW

GAANRDPRQYEDPDVFRADRNPVGHLAFGSGIHLCPGTQLARMEGQAILR

EIVANIDRIEVVEPPTWTTNANLRGLTRLRVAVTPRVAP

>CYP128A1 (2589531613) Mycobacterium tuberculosis TKK-01-0018

MTATQSPPEPAPDRVRLAGCPLAGTPDVGLTAQDATTALGVPTRRRASSG

GIPVATSMWRDAQTVRTYGPAVAKALALRVAGKARSRLTGRHCRKFMQLT

DFDPFDPAIAADPYPHYRELLAGERVQYNPKRDVYILSRYADVREAARNH

DTLSSARGVTFSRGWLPFLPTSDPPAHTRMRKQLAPGMARGALETWRPMV

DQLARELVGGLLTQTPADVVSTVAAPMPMRAITSVLGVDGPDEAAFCRLS

NQAVRITDVALSASGLISLVQGFAGFRRLRALFTHRRDNGLLRECTVLGK

LATHAEQGRLSDDELFFFAVLLLVAGYESTAHMISTLFLTLADYPDQLTL

LAQQPDLIPSAIEEHLRFISPIQNICRTTRVDYSVGQAVIPAGSLVLLAW

GAANRDPRQYEDPDVFRADRNPVGHLAFGSGIHLCPGTQLARMEGQAILR

EIVANIDRIEVVEPPTWTTNANLRGLTRLRVAVTPRVAP

>CYP128A1 (2590097102) Mycobacterium tuberculosis MAL020146

MTATQSPPEPAPDRVRLAGCPLAGTPDVGLTAQDATTALGVPTRRRASSG

GIPVATSMWRDAQTVRTYGPAVAKALALRVAGKARSRLTGRHCRKFMQLT

DFDPFDPAIAADPYPHYRELLAGERVQYNPKRDVYILSRYADVREAARNH

DTLSSARGVTFSRGWLPFLPTSDPPAHTRMRKQLAPGMARGALETWRPMV

DQLARELVGGLLTQTPADVVSTVAAPMPMRAITSVLGVDGPDEAAFCRLS

NQAVRITDVALSASGLISLVQGFAGFRRLRALFTHRRDNGLLRECTVLGK

LATHAEQGRLSDDELFFFAVLLLVAGYESTAHMISTLFLTLADYPDQLTL

LAQQPDLIPSAIEEHLRFISPIQNICRTTRVDYSVGQAVIPAGSLVLLAW

GAANRDPRQYEDPDVFRADRNPVGHLAFGSGIHLCPGPSWRAWRVRRSCA

RSSPISTE

>CYP128A1 (2621675461) Mycobacterium tuberculosis XTB13-299

MTATQSPPEPAPDRVRLAGCPLAGTPDVGLTAQDATTALGVPTRRRASSG

GIPVATSMWRDAQTVRTYGPAVAKALALRVAGKARSRLTGRHCRKFMQLT

DFDPFDPAIAADPYPHYRELLAGERVQYNPKRDVYILSRYADVREAARNH

DTLSSARGVTFSRGWLPFLPTSDPPAHTRMRKQLAPGMARGALETWRPMV

DQLARELVGGLLTQTPADVVSTVAAPMPMRAITSVLGVDGPDEAAFCRLS

NQAVRITDVALSASGLISLVQGFAGFRRLRALFTHRRDNGLLRECTVLGK

LATHAEQGRLSDDELFFFAVLLLVAGYESTAHMISTLFLTLADYPDQLTL

LAQQPDLIPSAIEEHLRFISPIQNICRTTRVDYSVGQAVIPAGSLVLLAW

GAANRDPRQYEDPDVFRADRNPVGHLAFGSGIHLCPGTQLARMEGQAILR

EIVANIDRIEVVEPPTWTTNANLRGLTRLRVAVTPRVAP

>CYP128A1 (2621911925) Mycobacterium tuberculosis TKK_05SA_0026

MTATQSPPEPAPDRVRLAGCPLAGTPDVGLTAQDATTALGVPTRRRASSG

GIPVATSMWRDAQTVRTYGPAVAKALALRVAGKARSRLTGRHCRKFMQLT

DFDPFDPAIAADPYPHYRELLAGERVQYNPKRDVYILSRYADVREAARNH

DTLSSARGVTFSRGWLPFLPTSDPPAHTRMRKQLAPGMARGALETWRPMV

DQLARELVGGLLTQTPADVVSTVAAPMPMRAITSVLGVDGPDEAAFCRLS

NQAVRITDVALSASGLISLVQGFAGFRRLRALFTHRRDNGLLRECTVLGK

LATHAEQGRLSDDELFFFAVLLLVAGYESTAHMISTLFLTLADYPDQLTL

LAQQPDLIPSAIEEHLRFISPIQNICRTTRVDYSVGQAVIPAGSLVLLAW

GAANRDPRQYEDPDVFRADRNPVGHLAFGSGIHLCPGTQLARMEGQAILR

EIVANIDRIEVVEPPTWTTNANLRGLTRLRVAVTPRVAP

>CYP128A1 (2633916029) Mycobacterium tuberculosis M2130

MTATQSPPEPAPDRVRLAGCPLAGTPDVGLTAQDATTALGVPTRRRASSG

GIPVATSMWRDAQTVRTYGPAVAKALALRVAGKARSRLTGRHCRKFMQLT

DFDPFDPAIAADPYPHYRELLAGERVQYNPKRDVYILSRYADVREAARNH

DTLSSARGVTFSRGWLPFLPTSDPPAHTRMRKQLAPGMARGALETWRPMV

DQLARELVGGLLTQTPADVVSTVAAPMPMRAITSVLGVDGPDEAAFCRLS

NQAVRITDVALSASGLISLVQGFAGFRRLRALFTHRRDNGLLRECTVLGK

LATHAEQGRLSDDELFFFAVLLLVAGYESTAHMISTLFLTLADYPDQLTL

LAQQPDLIPSAIEEHLRFISPIQNICRTTRVDYSVGQAVIPAGSLVLLAW

GAANRDPRQYEDPDVFRADRNPVGHLAFGSGIHLCPGTQLARMEGQAILR

EIVANIDRIEVVEPPTWTTNANLRGLTRLRVAVTPRVAP

>CYP128A1 (2659552631) Mycobacterium tuberculosis 200470

MTATQSPPEPAPDRVRLAGCPLAGTPDVGLTAQDATTALGVPTRRRASSG

GIPVATSMWRDAQTVRTYGPAVAKALALRVAGKARSRLTGRHCRKFMQLT

DFDPFDPAIAADPYPHYRELLAGERVQYNPKRDVYILSRYADVREAARNH

DTLSSARGVTFSRGWLPFLPTSDPPAHTRMRKQLAPGMARGALETWRPMV

DQLARELVGGLLTQTPADVVSTVAAPMPMRAITSVLGVDGPDEAAFCRLS

NQAVRITDVALSASGLISLVQGFAGFRRLRALFTHRRDNGLLRECTVLGK

LATHAEQGRLSDDELFFFAVLLLVAGYESTAHMISTLFLTLADYPDQLTL

LAQQPDLIPSAIEEHLRFISPIQNICRTTRVDYSVGQAVIPAGSLVLLAW

GAANRDPRQYEDPDVFRADRNPVGHLAFGSGIHLCPGTQLARMEGQAILR

EIVANIDRIEVVEPPTWTTNANLRGLTRLRVAVTPRVAP

>CYP128A1 (2691916086) Mycobacterium tuberculosis A70086

MTATQSPPEPAPDRVRLAGCPLAGTPDVGLTAQDATTALGVPTRRRASSG

GIPVATSMWRDAQTVRTYGPAVAKALALRVAGKARSRLTGRHCRKFMQLT

DFDPFDPAIAADPYPHYRELLAGERVQYNPKRDVYILSRYADVREAARNH

DTLSSARGVTFSRGWLPFLPTSDPPAHTRMRKQLAPGMARGALETWRPMV

DQLARELVGGLLTQTPADVVSTVAAPMPMRAITSVLGVDGPDEAAFCRLS

NQAVRITDVALSASGLISLVQGFAGFRRLRALFTHRRDNGLLRECTVLGK

LATHAEQGRLSDDELFFFAVLLLVAGYESTAHMISTLFLTLADYPDQLTL

LAQQPDLIPSAIEEHLRFISPIQNICRTTRVDYSVGQAVIPAGSLVLLAW

GAANRDPRQYEDPDVFRADRNPVGHLAFGSGIHLCPGTQLARMEGQAILR

EIVANIDRIEVVEPPTWTTNANLRGLTRLRVAVTPRVAP

>CYP128A1 (2693220738) Mycobacterium tuberculosis K00700186

MTATQSPPEPAPDRVRLAGCPLAGTPDVGLTAQDATTALGVPTRRRASSG

GIPVATSMWRDAQTVRTYGPAVAKALALRVAGKARSRLTGRHCRKFMQLT

DFDPFDPAIAADPYPHYRELLAGERVQYNPKRDVYILSRYADVREAARNH

DTLSSARGVTFSRGWLPFLPTSDPPAHTRMRKQLAPGMARGALETWRPMV

DQLARELVGGLLTQTPADVVSTVAAPMPMRAITSVLGVDGPDEAAFCRLS

NQAVRITDVALSASGLISLVQGFAGFRRLRALFTHRRDNGLLRECTVLGK

LATHAEQGRLSDDELFFFAVLLLVAGYESTAHMISTLFLTLADYPDQLTL

LAQQPDLIPSAIEEHLRFISPIQNICRTTRVDYSVGQAVIPAGSLVLLAW

GAANRDPRQYEDPDVFRADRNPVGHLAFGSGIHLCPGTQLARMEGQAILR

EIVANIDRIEVVEPPTWTTNANLRGLTRLRVAVTPRVAP

>CYP128A1 (2697407144) Mycobacterium tuberculosis M09400397

MTATQSPPEPAPDRVRLAGCPLAGTPDVGLTAQDATTALGVPTRRRASSG

GIPVATSMWRDAQTVRTYGPAVAKALALRVAGKARSRLTGRHCRKFMQLT

DFDPFDPAIAADPYPHYRELLAGERVQYNPKRDVYILSRYADVREAARNH

DTLSSARGVTFSRGWLPFLPTSDPPAHTRMRKQLAPGMARGALETWRPMV

DQLARELVGGLLTQTPADVVSTVAAPMPMRAITSVLGVDGPDEAAFCRLS

NQAVRITDVALSASGLISLVQGFAGFRRLRALFTHRRDNGLLRECTVLGK

LATHAEQGRLSDDELFFFAVLLLVAGYESTAHMISTLFLTLADYPDQLTL

LAQQPDLIPSAIEEHLRFISPIQNICRTTRVDYSVGQAVIPAGSLVLLAW

GAANRDPRQYEDPDVFRADRNPVGHLAFGSGIHLCPGTQLARMEGQAILR

EIVANIDRIEVVEPPTWTTNANLRGLTRLRVAVTPRVAP

>CYP128A1 (2699010856) Mycobacterium tuberculosis N00000064

MTATQSPPEPAPDRVRLAGCPLAGTPDVGLTAQDATTALGVPTRRRASSG

GIPVATSMWRDAQTVRTYGPAVAKALALRVAGKARSRLTGRHCRKFMQLT

DFDPFDPAIAADPYPHYRELLAGERVQYNPKRDVYILSRYADVREAARNH

DTLSSARGVTFSRGWLPFLPTSDPPAHTRMRKQLAPGMARGALETWRPMV

DQLARELVGGLLTQTPADVVSTVAAPMPMRAITSVLGVDGPDEAAFCRLS

NQAVRITDVALSASGLISLVQGFAGFRRLRALFTHRRDNGLLRECTVLGK

LATHAEQGRLSDDELFFFAVLLLVAGYESTAHMISTLFLTLADYPDQLTL

LAQQPDLIPSAIEEHLRFISPIQNICRTTRVDYSVGQAVIPAGSLVLLAW

GAANRDPRQYEDPDVFRADRNPVGHLAFGSGIHLCPGTQLARMEGQAILR

EIVANIDRIEVVEPPTWTTNANLRGLTRLRVAVTPRVAP

>CYP128A1 (2716358548) Mycobacterium tuberculosis H_13571

MTATQSPPEPAPDRVRLAGCPLAGTPDVGLTAQDATTALGVPTRRRASSG

GIPVATSMWRDAQTVRTYGPAVAKALALRVAGKARSRLTGRHCRKFMQLT

DFDPFDPAIAADPYPHYRELLAGERVQYNPKRDVYILSRYADVREAARNH

DTLSSARGVTFSRGWLPFLPTSDPPAHTRMRKQLAPGMARGALETWRPMV

DQLARELVGGLLTQTPADVVSTVAAPMPMRAITSVLGVDGPDEAAFCRLS

NQAVRITDVALSASGLISLVQGFAGFRRLRALFTHRRDNGLLRECTVLGK

LATHAEQGRLSDDELFFFAVLLLVAGYESTAHMISTLFLTLADYPDQLTL

LAQQPDLIPSAIEEHLRFISPIQNICRTTRVDYSVGQAVIPAGSLVLLAW

GAANRDPRQYEDPDVFRADRNPVGHLAFGSGIHLCPGTQLARMEGQAILR

EIVANIDRIEVVEPPTWTTNANLRGLTRLRVAVTPRVAP

>CYP128A1 (2574656816) Mycobacterium tuberculosis NRITLD12

MTATQSPPEPAPDRVRLAGCPLAGTPDVGLTAQDATTALGVPTRRRAASG

GIPVATSMWRDAQTVRTYGPAVAKALALRVAGKARSRLTGRHCRKFMQLT

DFDPFDPAIAADPYPHYRELLAGERVQYNPKRDVYILSRYADVREAARNH

DTLSSARGVTFSRGWLPFLPTSDPPAHTRMRKQLAPGMARGALETWRPMV

DQLARELVGGLLTQTPADVVSTVAAPMPMRAITSVLGVDGPDEAAFCRLS

NQAVRITDVALSASGLISLVQGFAGFRRLRALFTHRRDNGLLRECTVLGK

LATHAEQGRLSDDELFFFAVLLLVAGYESTAHMISTLFLTLADYPDQLTL

LAQQPDLIPSAIEEHLRFISPIQNICRTTRVDYSVGQAVIPAGSLVLLAW

GAANRDPRQYEDPDVFRADRNPVGHLAFGSGIHLCPGTQLARMEGQAILR

EIVANIDRIEVVEPPTWTTNANLRGLTRLRVAVTPRVAP

>CYP128B4 (2798610152) Mycobacteroides abscessus abscessus 980

MSRWRSDSAVRQIGAPVVAAVGMNIAAAVRVRRRGYAGWTGAVNTDYDPL

DPATAAQPFDAYRALHAGGRVHYNPKRATFILSRHEDIRAALRDTDAVTS

SQGVTRMKISAPILVLTDGDDHTRLRKQVQPGFTRGAMSDWQGMADQLAK

ELVADVVANPGCDVMERLAVPLPIRMIAHIIGIPPEDVQNFRSWSEDGVG

VINAGVSPAGLRQGLKGVRAIAALRRYFKDQLASGKLKGSDTVLGRLVDN

NEDGKLSDDELFFIAMLLLFAGNETTTNLIGGMFDTLAHAPDQFAMIRDD

PDLIPSAVEEQLRYSAPIQNLYRYTRTDYRVGEVTIPSGSRLLLAFGAAN

RDPEVFEDPDTYRADRNPRNHIAFGYGVHMCIGATLSRMEGQAVLRELTS

QASAIAAAGSATWSTNSSLRGTTYLPIRLTPAR

>CYP128B4 (2803008563) Mycobacteroides abscessus abscessus 100

MSRWRSDSAVRQIGAPVVAAVGMNIAAAVRVRRRGYAGWTGAVNTDYDPL

DPATAAQPFDAYRALHAGGRVHYNPKRATFILSRHEDIRAALRDTDAVTS

SQGVTRMKISAPILVLTDGDDHTRLRKQVQPGFTRGAMSDWQGMADQLAK

ELVADVVANPGCDVMERLAVPLPIRMIAHIIGIPPEDVQNFRSWSEDGVG

VINAGVSPAGLRQGLKGVRAIAALRRYFKDQLASGKLKGSDTVLGRLVDN

NEDGKLSDDELFFIAMLLLFAGNETTTNLIGGMFDTLAHAPDQFAMIRDD

PDLIPSAVEEQLRYSAPIQNLYRYTRTDYRVGEVTIPSGSRLLLAFGAAN

RDPEVFEDPDTYRADRNPRNHIAFGYGVHMCIGATLSRMEGQAVLRELTS

QASAIAAAGSATWSTNSSLRGTTYLPIRLTPAR

>CYP128B1 (2809907579) Mycobacterium sp. E2238

VSLSQTAQKATETLRPVADVVRMNVAAAVRTRRRGYNGWTGAINTDYDPQ

DPATAADPFEAYRALHRSGRVHYNPRRATFVLNRLDDVRAALRDTDKVTS

TQGVTRLRMSGSLAVLTDGEEHTRLRKQVQPGFSTGAMKSWQEMTEKLAI

DLVTDVLKDPGCDVVQRLAIPMPIRLIAQILGVPETDVGDFRRWSERAVG

VMELKPTLSGVVGAAKSMSAMVALQRYFTSQFAAGGLKGSSTVLGRLIEH

NTDGSLTDNQLLLIAIHLLIAGNETTTNLLGGMFDTLARRPEQYDLIRAN

PDLIPLAVEEQLRFTTPIQNLYRYTRADYRIGDVTIPTGSRVLLSFGAAN

RDPAAFDDPDEYRADRNPRMHVAFGYGPHMCLGAPLARMEAQAILRQLVT

RVARITPAGPTRWSSHSSLRGPTRLPIRLTPA

>CYP128A1 (2577509335) Mycobacterium tuberculosis MD20344

MTATQSPPEPAPDRVRLAGCPLAGTPDVGLTAQDATTALGVPTRRRASSG

GIPVATSMWRDAQTVRTYGPAVAKALALRVAGKARSRLTGRHCRKFMQLT

DFDPFDPAIAADPYPHYRELLAGERVQYNPKRDVYILSRYADVREAARNH

DTLSSARGVTFSRGWLPFLPTSDPPAHTRMRKQLAPGMARGALETWRPMV

DQLARELVGGLLTQTPADVVSTVAAPMPMRAITSVLGVDGPDEAAFCRLS

NQAVRITDVALSASGLISLVQGFAGFRRLRALFTHRRDNGLLRECTVLGK

LATHAEQGRLSDDELFFFAVLLLVAGYESTAHMISTLFLTLADYPDQLTL

LAQQPDLIPSAIEEHLRFISPIQNICRTTRVDYSVGQAVIPAGSLVLLAW

GAANRDPRQYEDPDVFRADRNPVGHLAFGSGIHLCPGTQLARMEGQAILR

EIVANIDRIEVVEPPTWTTNANLRGLTRLRVAVTPRVAP

>CYP128A1 (2604960481) Mycobacterium tuberculosis BTB09-377

MTATQSPPEPAPDRVRLAGCPLAGTPDVGLTAQDATTALGVPTRRRASSG

GIPVATSMWRDAQTVRTYGPAVAKALALRVAGKARSRLTGRHCRKFMQLT

DFDPFDPAIAADPYPHYRELLAGERVQYNPKRDVYILSRYADVREAARNH

DTLSSARGVTFSRGWLPFLPTSDPPAHTRMRKQLAPGMARGALETWRPMV

DQLARELVGGLLTQTPADVVSTVAAPMPMRAITSVLGVDGPDEAAFCRLS

NQAVRITDVALSASGLISLVQGFAGFRRLRALFTHRRDNGLLRECTVLGK

LATHAEQGRLSDDELFFFAVLLLVAGYESTAHMISTLFLTLADYPDQLTL

LAQQPDLIPSAIEEHLRFISPIQNICRTTRVDYSVGQAVIPAGSLVLLAW

GAANRDPRQYEDPDVFRADRNPVGHLAFGSGIHLCPGTQLARMEGQAILR

EIVANIDRIEVVEPPTWTTNANLRGLTRLRVAVTPRVAP

>CYP128A1 (2605034972) Mycobacterium tuberculosis TB_RSA42

MTATQSPPEPAPDRVRLAGCPLAGTPDVGLTAQDATTALGVPTRRRASSG

GIPVATSMWRDAQTVRTYGPAVAKALALRVAGKARSRLTGRHCRKFMQLT

DFDPFDPAIAADPYPHYRELLAGERVQYNPKRDVYILSRYADVREAARNH

DTLSSARGVTFSRGWLPFLPTSDPPAHTRMRKQLAPGMARGALETWRPMV

DQLARELVGGLLTQTPADVVSTVAAPMPMRAITSVLGVDGPDEAAFCRLS

NQAVRITDVALSASGLISLVQGFAGFRRLRALFTHRRDNGLLRECTVLGK

LATHAEQGRLSDDELFFFAVLLLVAGYESTAHMISTLFLTLADYPDQLTL

LAQQPDLIPSAIEEHLRFISPIQNICRTTRVDYSVGQAVIPAGSLVLLAW

GAANRDPRQYEDPDVFRADRNPVGHLAFGSGIHLCPGTQLARMEGQAILR

EIVANIDRIEVVEPPTWTTNANLRGLTRLRVAVTPRVAP

>CYP128A1 (2621687005) Mycobacterium tuberculosis 7057

MTATQSPPEPAPDRVRLAGCPLAGTPDVGLTAQDATTALGVPTRRRASSG

GIPVATSMWRDAQTVRTYGPAVAKALALRVAGKARSRLTGRHCRKFMQLT

DFDPFDPAIAADPYPHYRELLAGERVQYNPKRDVYILSRYADVREAARNH

DTLSSARGVTFSRGWLPFLPTSDPPAHTRMRKQLAPGMARGALETWRPMV

DQLARELVGGLLTQTPADVVSTVAAPMPMRAITSVLGVDGPDEAAFCRLS

NQAVRITDVALSASGLISLVQGFAGFRRLRALFTHRRDNGLLRECTVLGK

LATHAEQGRLSDDELFFFAVLLLVAGYESTAHMISTLFLTLADYPDQLTL

LAQQPDLIPSAIEEHLRFISPIQNICRTTRVDYSVGQAVIPAGSLVLLAW

GAANRDPRQYEDPDVFRADRNPVGHLAFGSGIHLCPGTQLARMEGQAILR

EIVANIDRIEVVEPPTWTTNANLRGLTRLRVAVTPRVAP

>CYP128A1 (2621711334) Mycobacterium tuberculosis 6273

MTATQSPPEPAPDRVRLAGCPLAGTPDVGLTAQDATTALGVPTRRRASSG

GIPVATSMWRDAQTVRTYGPAVAKALALRVAGKARSRLTGRHCRKFMQLT

DFDPFDPAIAADPYPHYRELLAGERVQYNPKRDVYILSRYADVREAARNH

DTLSSARGVTFSRGWLPFLPTSDPPAHTRMRKQLAPGMARGALETWRPMV

DQLARELVGGLLTQTPADVVSTVAAPMPMRAITSVLGVDGPDEAAFCRLS

NQAVRITDVALSASGLISLVQGFAGFRRLRALFTHRRDNGLLRECTVLGK

LATHAEQGRLSDDELFFFAVLLLVAGYESTAHMISTLFLTLADYPDQLTL

LAQQPDLIPSAIEEHLRFISPIQNICRTTRVDYSVGQAVIPAGSLVLLAW

GAANRDPRQYEDPDVFRADRNPVGHLAFGSGIHLCPGPSWRAWRVRRSCA

RSSPISTE

>CYP128A1 (2638527330) Mycobacterium tuberculosis M2145

MTATQSPPEPAPDRVRLAGCPLAGTPDVGLTAQDATTALGVPTRRRASSG

GIPVATSMWRDAQTVRTYGPAVAKALALRVAGKARSRLTGRHCRKFMQLT

DFDPFDPAIAADPYPHYRELLAGERVQYNPKRDVYILSRYADVREAARNH

DTLSSARGVTFSRGWLPFLPTSDPPAHTRMRKQLAPGMARGALETWRPMV

DQLARELVGGLLTQTPADVVSTVAAPMPMRAITSVLGVDGPDEAAFCRLS

NQAVRITDVALSASGLISLVQGFAGFRRLRALFTHRRDNGLLRECTVLGK

LATHAEQGRLSDDELFFFAVLLLVAGYESTAHMISTLFLTLADYPDQLTL

LAQQPDLIPSAIEEHLRFISPIQNICRTTRVDYSVGQAVIPAGSLVLLAW

GAANRDPRQYEDPDVFRADRNPVGHLAFGSGIHLCPGTQLARMEGQAILR

EIVANIDRIEVVEPPTWTTNANLRGLTRLRVAVTPRVAP

>CYP128A1 (2688178339) Mycobacterium tuberculosis 26105

MTATQSPPEPAPDRVRLAGCPLAGTPDVGLTAQDATTALGVPTRRRASSG

GIPVATSMWRDAQTVRTYGPAVAKALALRVAGKARSRLTGRHCRKFMQLT

DFDPFDPAIAADPYPHYRELLAGERVQYNPKRDVYILSRYADVREAARNH

DTLSSARGVTFSRGWLPFLPTSDPPAHTRMRKQLAPGMARGALETWRPMV

DQLARELVGGLLTQTPADVVSTVAAPMPMRAITSVLGVDGPDEAAFCRLS

NQAVRITDVALSASGLISLVQGFAGFRRLRALFTHRRDNGLLRECTVLGK

LATHAEQGRLSDDELFFFAVLLLVAGYESTAHMISTLFLTLADYPDQLTL

LAQQPDLIPSAIEEHLRFISPIQNICRTTRVDYSVGQAVIPAGSLVLLAW

GAANRDPRQYEDPDVFRADRNPVGHLAFGSGIHLCPGTQLARMEGQAILR

EIVANIDRIEVVEPPTWTTNANLRGLTRLRVAVTPRVAP

>CYP128A1 (2703582642) Mycobacterium tuberculosis T09800928

MTATQSPPEPAPDRVRLAGCPLAGTPDVGLTAQDATTALGVPTRRRASSG

GIPVATSMWRDAQTVRTYGPAVAKALALRVAGKARSRLTGRHCRKFMQLT

DFDPFDPAIAADPYPHYRELLAGERVQYNPKRDVYILSRYADVREAARNH

DTLSSARGVTFSRGWLPFLPTSDPPAHTRMRKQLAPGMARGALETWRPMV

DQLARELVGGLLTQTPADVVSTVAAPMPMRAITSVLGVDGPDEAAFCRLS

NQAVRITDVALSASGLISLVQGFAGFRRLRALFTHRRDNGLLRECTVLGK

LATHAEQGRLSDDELFFFAVLLLVAGYESTAHMISTLFLTLADYPDQLTL

LAQQPDLIPSAIEEHLRFISPIQNICRTTRVDYSVGQAVIPAGSLVLLAW

GAANRDPRQYEDPDVFRADRNPVGHLAFGSGIHLCPGTQLARMEGQAILR

EIVANIDRIEVVEPPTWTTNANLRGLTRLRVAVTPRVAP

>CYP128A1 (2706580312) Mycobacterium tuberculosis E09701891

MTATQSPPEPAPDRVRLAGCPLAGTPDVGLTAQDATTALGVPTRRRASSG

GIPVATSMWRDAQTVRTYGPAVAKALALRVAGKARSRLTGRHCRKFMQLT

DFDPFDPAIAADPYPHYRELLAGERVQYNPKRDVYILSRYADVREAARNH

DTLSSARGVTFSRGWLPFLPTSDPPAHTRMRKQLAPGMARGALETWRPMV

DQLARELVGGLLTQTPADVVSTVAAPMPMRAITSVLGVDGPDEAAFCRLS

NQAVRITDVALSASGLISLVQGFAGFRRLRALFTHRRDNGLLRECTVLGK

LATHAEQGRLSDDELFFFAVLLLVAGYESTAHMISTLFLTLADYPDQLTL

LAQQPDLIPSAIEEHLRFISPIQNICRTTRVDYSVGQAVIPAGSLVLLAW

GAANRDPRQYEDPDVFRADRNPVGHLAFGSGIHLCPGTQLARMEGQAILR

EIVANIDRIEVVEPPTWTTNANLRGLTRLRVAVTPRVAP

>CYP128A1 (643019675) Mycobacterium tuberculosis 94_M4241A

MRLAGCPLAGTPDVGLTAQDATTALGVPTRRRASSGGIPVATSMWRDAQT

VRTYGPAVAKALALRVAGKARSRLTGRHCRKFMQLTDFDPFDPAIAADPY

PHYRELLAGERVQYNPKRDVYILSRYADVREAARNHDTLSSARGVTFSRG

WLPFLPTSDPPAHTRMRKQLAPGMARGALETWRPMVDQLARELVGGLLTQ

TPADVVSTVAAPMPMRAITSVLGVDGPDEAAFCRLSNQAVRITDVALSAS

GLISLVQGFAGFRRLRALFTHRRDNGLLRECTVLGKLATHAEQGRLSDDE

LFFFAVLLLVAGYESTAHMISTLFLTLADYPDQLTLLAQQPDLIPSAIEE

HLRFISPIQNICRTTRVDYSVGQAVIPAGSLVLLAWGAANRDPRQYEDPD

VFRADRNPVGHLAFGSGIHLCPGTQLARMEGQAILREIVANIDRIEVVEP

PTWTTNANLRGLTRLRVAVTPRVAP

>CYP128B4 (2659570677) Mycobacterium abscessus PAP060

MSRWRSDSAVRQIGAPVVAAVGMNIAAAVRVRRRGYAGWTGAVNTDYDPL

DPATAAQPFDAYRALHAGGRVHYNPKRATFILSRHEDIRAALRDTDAVTS

SQGVTRMKISAPILVLTDGDDHTRLRKQVQPGFTRGAMSDWQGMADQLAK

ELVADVVANPGCDVMERLAVPLPIRMIAHIIGIPPEDVQNFRSWSEDGVG

VINAGVSPAGLRQGLKGVRAIAALRRYFKDQLASGKLKGSDTVLGRLVDN

NEDGKLSDDELFFIAMLLLFAGNETTTNLIGGMFDTLAHAPDQFAMIRDD

PDLIPSAVEEQLRYSAPIQNLYRYTRTDYRVGEVTIPSGSRLLLAFGAAN

RDPEVFEDPDTYRADRNPRNHIAFGYGVHMCIGATLSRMEGQAVLRELTS

QASAIAAAGSATWSTNSSLRGTTYLPIRLTPAR

>CYP128B4 (2691741634) Mycobacterium abscessus PAP012

MSRWRSDSAVRQIGAPVVAAVGMNIAAAVRVRRRGYAGWTGAVNTDYDPL

DPATAAQPFDAYRALHAGGRVHYNPKRATFILSRHEDIRAALRDTDAVTS

SQGVTRMKISAPILVLTDGDDHTRLRKQVQPGFTRGAMSDWQGMADQLAK

ELVADVVANPGCDVMERLAVPLPIRMIAHIIGIPPEDVQNFRSWSEDGVG

VINAGVSPAGLRQGLKGVRAIAALRRYFKDQLASGKLKGSDTVLGRLVDN

NEDGKLSDDELFFIAMLLLFAGNETTTNLIGGMFDTLAHAPDQFAMIRDD

PDLIPSAVEEQLRYSAPIQNLYRYTRTDYRVGEVTIPSGSRLLLAFGAAN

RDPEVFEDPDTYRADRNPRNHIAFGYGVHMCIGATLSRMEGQAVLRELTS

QASAIAAAGSATWSTNSSLRGTTYLPIRLTPAR

>CYP128B1 (2809950795) Mycobacterium alsense 1137317.9

MSISQTAQGAAEAIRPFSNAVRMNLTAAVRTRRRGYGAWTGAVNTDYDPQ

NPTTAAQPFDAYRALHRTGRVHYNPRRATWIISRLDDVRAALRDTDQVTS

TQGVTRLRMSAPLAVLTDGEEHTRLRKQVQPGFSKGAMSAWQEIVEKLAV

ELVADLLNNPGCDVVQHLAIPMPIRLIAQILGVPEGDVNDFRRWSENAVK

VMELTPTRAGFVGAVRSISAMVALQRYFVKQFAVGGLKGSGTVLGRLLDQ

NTDGSLTDRQLWLIAIHLLIAGNETTTNLLGGMFDTLANHPDQYDLICAN

PDIIPMAVEEQLRITTPIQNLYRYTRADYQIGHVTIPAGSRVLLSFGAAN

RDPAVFDQPDEYRADRNPRTHVAFGYGAHMCLGAPLARMEAQAVLRELVT

RVSRLTPKGSTTWSTHSSLRGPTHLPIHLTSA

>CYP128A1 (2546202677) Mycobacterium tuberculosis CAS/NITR204

MQLTDFDPFDPAIAADPYPHYRELLAGERVQYNPKRDVYILSRYADVREA

ARNHDTLSSARGVTFSRGWLPFLPTSDPPAHTRMRKQLAPGMARGALETW

RPMVDQLARELVGGLLTQTPADVVSTVAAPMPMRAITSVLGVDGPDEAAF

CRLSNQAVRITDVALSASGLISLVQGFAGFRRLRALFTHRRDNGLLRECT

VLGKLATHAEQGRLSDDELFFFAVLLLVAGYESTAHMISTLFLTLADYPD

QLTLLAQQPDLIPSAIEEHLRFISPIQNICRTTRVDYSVGQAVIPAGSLV

LLAWGAANRDPRQYEDPDVFRADRNPVGHLAFGSGIHLCPGTQLARMEGQ

AILREIVANIDRIEVVEPPTWTTNANLRGLTRLRVAVTPRVAP

>CYP128A1 (2577060968) Mycobacterium tuberculosis BTB12-314

MTATQSPPEPAPDRVRLAGCPLAGTPDVGLTAQDATTALGVPTRRRASSG

GIPVATSMWRDAQTVRTYGPAVAKALALRVAGKARSRLTGRHCRKFMQLT

DFDPFDPAIAADPYPHYRELLAGERVQYNPKRDVYILSRYADVREAARNH

DTLSSARGVTFSRGWLPFLPTSDPPAHTRMRKQLAPGMARGALETWRPMV

DQLARELVGGLLTQTPADVVSTVAAPMPMRAITSVLGVDGPDEAAFCRLS

NQAVRITDVALSASGLISLVQGFAGFRRLRALFTHRRDNGLLRECTVLGK

LATHAEQGRLSDDELFFFAVLLLVAGYESTAHMISTLFLTLADYPDQLTL

LAQQPDLIPSAIEEHLRFISPIQNICRTTRVDYSVGQAVIPAGSLVLLAW

GAANRDPRQYEDPDVFRADRNPVGHLAFGSGIHLCPGTQLARMEGQAILR

EIVANIDRIEVVEPPTWTTNANLRGLTRLRVAVTPRVAP

>CYP128A1 (2584964024) Mycobacterium tuberculosis H2438

MTATQSPPEPAPDRVRLAGCPLAGTPDVGLTAQDATTALGVPTRRRASSG

GIPVATSMWRDAQTVRTYGPAVAKALALRVAGKARSRLTGRHCRKFMQLT

DFDPFDPAIAADPYPHYRELLAGERVQYNPKRDVYILSRYADVREAARNH

DTLSSARGVTFSRGWLPFLPTSDPPAHTRMRKQLAPGMARGALETWRPMV

DQLARELVGGLLTQTPADVVSTVAAPMPMRAITSVLGVDGPDEAAFCRLS

NQAVRITDVALSASGLISLVQGFAGFRRLRALFTHRRDNGLLRECTVLGK

LATHAEQGRLSDDELFFFAVLLLVAGYESTAHMISTLFLTLADYPDQLTL

LAQQPDLIPSAIEEHLRFISPIQNICRTTRVDYSVGQAVIPAGSLVLLAW

GAANRDPRQYEDPDVFRADRNPVGHLAFGSGIHLCPGTQLARMEGQAILR

EIVANIDRIEVVEPPTWTTNANLRGLTRLRVAVTPRVAP

>CYP128A1 (2590036050) Mycobacterium tuberculosis MAL010106

MTATQSPPEPAPDRVRLAGCPLAGTPDVGLTAQDATTALGVPTRRRASSG

GIPVATSMWRDAQTVRTYGPAVAKALALRVAGKARSRLTGRHCRKFMQLT

DFDPFDPAIAADPYPHYRELLAGERVQYNPKRDVYILSRYADVREAARNH

DTLSSARGVTFSRGWLPFLPTSDPPAHTRMRKQLAPGMARGALETWRPMV

DQLARELVGGLLTQTPADVVSTVAAPMPMRAITSVLGVDGPDEAAFCRLS

NQAVRITDVALSASGLISLVQGFAGFRRLRALFTHRRDNGLLRECTVLGK

LATHAEQGRLSDDELFFFAVLLLVAGYESTAHMISTLFLTLADYPDQLTL

LAQQPDLIPSAIEEHLRFISPIQNICRTTRVDYSVGQAVIPAGSLVLLAW

GAANRDPRQYEDPDVFRADRNPVGHLAFGSGIHLCPGTQLARMEGQAILR

EIVANIDRIEVVEPPTWTTNANLRGLTRLRVAVTPRVAP

>CYP128A1 (2603258355) Mycobacterium tuberculosis TB_RSA169

MTATQSPPEPAPDRVRLAGCPLAGTPDVGLTAQDATTALGVPTRRRASSG

GIPVATSMWRDAQTVRTYGPAVAKALALRVAGKARSRLTGRHCRKFMQLT

DFDPFDPAIAADPYPHYRELLAGERVQYNPKRDVYILSRYADVREAARNH

DTLSSARGVTFSRGWLPFLPTSDPPAHTRMRKQLAPGMARGALETWRPMV

DQLARELVGGLLTQTPADVVSTVAAPMPMRAITSVLGVDGPDEAAFCRLS

NQAVRITDVALSASGLISLVQGFAGFRRLRALFTHRRDNGLLRECTVLGK

LATHAEQGRLSDDELFFFAVLLLVAGYESTAHMISTLFLTLADYPDQLTL

LAQQPDLIPSAIEEHLRFISPIQNICRTTRVDYSVGQAVIPAGSLVLLAW

GAANRDPRQYEDPDVFRADRNPVGHLAFGSGIHLCPGTQLARMEGQAILR

EIVANIDRIEVVEPPTWTTNANLRGLTRLRVAVTPRVAP

>CYP128A1 (2622142178) Mycobacterium tuberculosis MD15226

MTATQSPPEPAPDRVRLAGCPLAGTPDVGLTAQDATTALGVPTRRRASSG

GIPVATSMWRDAQTVRTYGPAVAKALALRVAGKARSRLTGRHCRKFMQLT

DFDPFDPAIAADPYPHYRELLAGERVQYNPKRDVYILSRYADVREAARNH

DTLSSARGVTFSRGWLPFLPTSDPPAHTRMRKQLAPGMARGALETWRPMV

DQLARELVGGLLTQTPADVVSTVAAPMPMRAITSVLGVDGPDEAAFCRLS

NQAVRITDVALSASGLISLVQGFAGFRRLRALFTHRRDNGLLRECTVLGK

LATHAEQGRLSDDELFFFAVLLLVAGYESTAHMISTLFLTLADYPDQLTL

LAQQPDLIPSAIEEHLRFISPIQNICRTTRVDYSVGQAVIPAGSLVLLAW

GAANRDPRQYEDPDVFRADRNPVGHLAFGSGIHLCPGTQLARMEGQAILR

EIVANIDRIEVVEPPTWTTNANLRGLTRLRVAVTPRVAP

>CYP128A1 (2645903075) Mycobacterium tuberculosis Mtb293

MTATQSPPEPAPDRVRLAGCPLAGTPDVGLTAQDATTALGVPTRRRASSG

GIPVATSMWRDAQTVRTYGPAVAKALALRVAGKARSRLTGRHCRKFMQLT

DFDPFDPAIAADPYPHYRELLAGERVQYNPKRDVYILSRYADVREAARNH

DTLSSARGVTFSRGWLPFLPTSDPPAHTRMRKQLAPGMARGALETWRPMV

DQLARELVGGLLTQTPADVVSTVAAPMPMRAITSVLGVDGPDEAAFCRLS

NQAVRITDVALSASGLISLVQGFAGFRRLRALFTHRRDNGLLRECTVLGK

LATHAEQGRLSDDELFFFAVLLLVAGYESTAHMISTLFLTLADYPDQLTL

LAQQPDLIPSAIEEHLRFISPIQNICRTTRVDYSVGQAVIPAGSLVLLAW

GAANRDPRQYEDPDVFRADRNPVGHLAFGSGIHLCPGTQLARMEGQAILR

EIVANIDRIEVVEPPTWTTNANLRGLTRLRVAVTPRVAP

>CYP128A1 (2677315155) Mycobacterium tuberculosis A00400237

MTATQSPPEPAPDRVRLAGCPLAGTPDVGLTAQDATTALGVPTRRRASSG

GIPVATSMWRDAQTVRTYGPAVAKALALRVAGKARSRLTGRHCRKFMQLT

DFDPFDPAIAADPYPHYRELLAGERVQYNPKRDVYILSRYADVREAARNH

DTLSSARGVTFSRGWLPFLPTSDPPAHTRMRKQLAPGMARGALETWRPMV

DQLARELVGGLLTQTPADVVSTVAAPMPMRAITSVLGVDGPDEAAFCRLS

NQAVRITDVALSASGLISLVQGFAGFRRLRALFTHRRDNGLLRECTVLGK

LATHAEQGRLSDDELFFFAVLLLVAGYESTAHMISTLFLTLADYPDQLTL

LAQQPDLIPSAIEEHLRFISPIQNICRTTRVDYSVGQAVIPAGSLVLLAW

GAANRDPRQYEDPDVFRADRNPVGHLAFGSGIHLCPGTQLARMEGQAILR

EIVANIDRIEVVEPPTWTTNANLRGLTRLRVAVTPRVAP

>CYP128A1 (2694243526) Mycobacterium tuberculosis A70196

MTATQSPPEPAPDRVRLAGCPLAGTPDVGLTAQDATTALGVPTRRRASSG

GIPVATSMWRDAQTVRTYGPAVAKALALRVAGKARSRLTGRHCRKFMQLT

DFDPFDPAIAADPYPHYRELLAGERVQYNPKRDVYILSRYADVREAARNH

DTLSSARGVTFSRGWLPFLPTSDPPAHTRMRKQLAPGMARGALETWRPMV

DQLARELVGGLLTQTPADVVSTVAAPMPMRAITSVLGVDGPDEAAFCRLS

NQAVRITDVALSASGLISLVQGFAGFRRLRALFTHRRDNGLLRECTVLGK

LATHAEQGRLSDDELFFFAVLLLVAGYESTAHMISTLFLTLADYPDQLTL

LAQQPDLIPSAIEEHLRFISPIQNICRTTRVDYSVGQAVIPAGSLVLLAW

GAANRDPRQYEDPDVFRADRNPVGHLAFGSGIHLCPGTQLARMEGQAILR

EIVANIDRIEVVEPPTWTTNANLRGLTRLRVAVTPRVAP

>CYP128A1 (2548029549) Mycobacterium tuberculosis S96-129

MTATQSPPEPAPDRVRLAGCPLAGTPDVGLTAQDATTALGVPTRRRASSG

GIPVATSMWRDAQTVRTYGPAVAKALALRVAGKARSRLTGRHCRKFMQLT

DFDPFDPAIAADPYPHYRELLAGERVQDNPKRDVYILSRYADVREAARNH

DTLSSARGVTFSRGWLPFLPTSDPPAHTRMRKQLAPGMARGALETWRPMV

DQLARELVGGLLTQTPADVVSTVAAPMPMRAITSVLGVDGPDEAAFCRLS

NQAVRITDVALSASGLISLVQGFAGFRRLRALFTHRRDNGLLRECTVLGK

LATHAEQGRLSDDELFFFAVLLLVAGYESTAHMISTLFLTLADYPDQLTL

LAQQPDLIPSAIEEHLRFISPIQNICRTTRVDYSVGQAVIPAGSLVLLAW

GAANRDPRQYEDPDVFRADRNPVGHLAFGSGIHLCPGTQLARMEGQAILR

EIVANIDRIEVVEPPTWTTNANLRGLTRLRVAVTPRVAP

>CYP128A1 (2605386092) Mycobacterium tuberculosis TB_RSA175

MTATQSPPEPAPDRVRLAGCPLAGTPDVGLTAQDATTALGVPTRRRASSG

GIPVATSMWRDAQTVRTYGPAVAKALALRVAGKARSRLTGRHCRKFMQLT

DFDPFDPAIAADPYPHYRELLAGERVQYNPKRDVYILSRYADVREAARNH

DTLSSARGVTFSRGWLPFLPTSDPPAHTRMRKQLAPGMARGALETWRPMV

DQLARELVGGLLTQTPADVVSTVAAPMPMRAITSVLGVDGPDEAAFCRLS

NQAVRITDVALSASGLISLVQGFAGFRRLRALFTHRRDNGLLRECTVLGK

LATHAEQGRLSDDELFFFAVLLLVAGYESPAHMISTLFLTLADYPDQLTL

LAQQPDLIPSAIEEHLRFISPIQNICRTTRVDYSVGQAVIPAGSLVLLAW

GAANRDPRQYEDPDVFRADRNPVGHLAFGSGIHLCPGTQLARMEGQAILR

EIVANIDRIEVVEPPTWTTNANLRGLTRLRVAVTPRVAP

>CYP128A1 (2629872102) Mycobacterium tuberculosis TKK_03_0065

MTATQSPPEPAPDRVRLAGCPLAGTPDVGLTAQDATTALGVPTRRRASSG

GIPVATSMWRDAQTVRTYGPAVAKALALRVAGKARSRLTGRHCRKFMQLT

DFDPFDPAIAADPYPHYRELLAGERVQYNPKRDVYILSRYADVREAARNH

DTLSSARGVTFSRGWLPFLPTSDPPAHTRMRKQLAPGMARGALETWRPMV

DQLARELVGGLLTQTPADVVSTVAAPMPMRAITSVLGVDGPDEAAFCRLS

NQAVRITDVALSASGLISLVQGFAGFRRLRALFTHRRDNGLLRECTVLGK

LATHAEQGRLSDDELFFFAVLLLVAGYESPAHMISTLFLTLADYPDQLTL

LAQQPDLIPSAIEEHLRFISPIQNICRTTRVDYSVGQAVIPAGSLVLLAW

GAANRDPRQYEDPDVFRADRNPVGHLAFGSGIHLCPGTQLARMEGQAILR

EIVANIDRIEVVEPPTWTTNANLRGLTRLRVAVTPRVAP

>CYP128A1 (2772258230) Mycobacterium sp. EPM10906

MTLKHDMRLVRTTTRPVVRSIVTNASNELRAKVRRRPFPGVQDTAFDPMD

PQTAANPHAGYRELLAGGRVHYNRKRNIFILCRYEDVRAAARNDALLSNR

DGVVRARFEVPVLLNMDRPRHTELRRKALPGFTRGALEGWAPTVDRLAHE

LMTDLLDNPGVDVVEHLAVPLPMRMIAHVLGIPPEDEAFFRHWSNESVRV

ADVQFSLKGVGQVPATLNGVRHLHDYFLTQLDKGNLLGPDTLLGKLVGEA

GENEISHDELFFLALLLLLAGNETTTNLLSTMFLTLSENPDQFELIRSDP

GLVAGAVEEQLRFSSPIQNFYRTAAQDYCVGDAIIPAGARVALLWGAANR

DPREFDEPDRFLAARPVGQHVAFGSGVHLCLGAGLARMEGQAVLRELVRR

VDRIDITGTPRWTTNSSLRGLEELRVRLVSL

>CYP128B4 (2797137862) Mycobacteroides abscessus abscessus 503

MSRWRSDSAVRQIGAPVVAAVGMNIAAAVRVRRRGYAGWTGAVNTDYDPL

DPATAAQPFDAYRALHAGGRVHYNPKRATFILSRHEDIRAALRDTDAVTS

SQGVTRMKISAPILVLTDGDDHTRLRKQVQPGFTRGAMSDWQGMADQLAK

ELVADVVANPGCDVMERLAVPLPIRMIAHIIGIPPEDVQNFRSWSEDGVG

VINAGVSPAGLRQGLKGVRAIAALRRYFKDQLASGKLKGSDTVLGRLVDN

NEDGKLSDDELFFIAMLLLFAGNETTTNLIGGMFDTLAHAPDQFAMIRDD

PDLIPSAVEEQLRYSAPIQNLYRYTRTDYRVGEVTIPSGSRLLLAFGAAN

RDPEVFEDPDTYRADRNPRNHIAFGYGVHMCIGATLSRMEGQAVLRELTS

QASAIAAAGSATWSTNSSLRGTTYLPIRLTPAR

>CYP128B4 (2803064059) Mycobacteroides abscessus abscessus 382

MSRWRSDSAVRQIGAPVVAAVGMNIAAAVRVRRRGYAGWTGAVNTDYDPL

DPATAAQPFDAYRALHAGGRVHYNPKRATFILSRHEDIRAALRDTDAVTS

SQGVTRMKISAPILVLTDGDDHTRLRKQVQPGFTRGAMSDWQGMADQLAK

ELVADVVANPGCDVMERLAVPLPIRMIAHIIGIPPEDVQNFRSWSEDGVG

VINAGVSPAGLRQGLKGVRAIAALRRYFKDQLASGKLKGSDTVLGRLVDN

NEDGKLSDDELFFIAMLLLFAGNETTTNLIGGMFDTLAHAPDQFAMIRDD

PDLIPSAVEEQLRYSAPIQNLYRYTRTDYRVGEVTIPSGSRLLLAFGAAN

RDPEVFEDPDTYRADRNPRNHIAFGYGVHMCIGATLSRMEGQAVLRELTS

QASAIAAAGSATWSTNSSLRGTTYLPIRLTPAR

>CYP128B4 (2803857202) Mycobacteroides abscessus abscessus 1016

MSRWRSDSAVRQIGAPVVAAVGMNIAAAVRVRRRGYAGWTGAVNTDYDPL

DPATAAQPFDAYRALHAGGRVHYNPKRATFILSRHEDIRAALRDTDAVTS

SQGVTRMKISAPILVLTDGDDHTRLRKQVQPGFTRGAMSDWQGMADQLAK

ELVADVVANPGCDVMERLAVPLPIRMIAHIIGIPPEDVQNFRSWSEDGVG

VINAGVSPAGLRQGLKGVRAIAALRRYFKDQLASGKLKGSDTVLGRLVDN

NEDGKLSDDELFFIAMLLLFAGNETTTNLIGGMFDTLAHAPDQFAMIRDD

PDLIPSAVEEQLRYSAPIQNLYRYTRTDYRVGEVTIPSGSRLLLAFGAAN

RDPEVFEDPDTYRADRNPRNHIAFGYGVHMCIGATLSRMEGQAVLRELTS

QASAIAAAGSATWSTNSSLRGTTYLPIRLTPAR

>CYP128A1 (2573561338) Mycobacterium tuberculosis INS_XDR

MTATQSPPEPAPDRVRLAGCPLAGTPDVGLTAQDATTALGVPTRRRASSG

GIPVATSMWRDAQTVRTYGPAVAKALALRVAGKARSRLTGRHCRKFMQLT

DFDPFDPAIAADPYPHYRELLAGERVQYNPKRDVYILSRYADVREAARNH

DTLSSARGVTFSRGWLPFLPTSDPPAHTRMRKQLAPGMARGALETWRPMV

DQLARELVGGLLTQTPADVVSTVAAPMPMRAITSVLGVDGPDEAAFCRLS

NQAVRITDVALSASGLISLVQGFAGFRRLRALFTHRRDNGLLRECTVLGK

LATHAEQGRLSDDELFFFAVLLLVAGYESTAHMISTLFLTLADYPDQLTL

LAQQPDLIPSAIEEHLRFISPIQNICRTTRVDYSVGQAVIPAGSLVLLAW

GAANRDPRQYEDPDVFRADRNPVGHLAFGSGIHLCPGTQLARMEGQAILR

EIVANIDRIEVVEPPTWTTNANLRGLTRLRVAVTPRVAP

>CYP128A1 (2582002507) Mycobacterium africanum MAL010128

MTATQSPPEPAPDRVRLAGCPLAGTPDVGLTAQDATTALGVPTRRRASSG

GIPVATSMWRDAQTVRTYGPAVAKALALRVAGKARSRLTGRHCRKFMQLT

DFDPFDPAIAADPYPHYRELLAGERVQYNPKRDVYILSRYADVREAARNH

DTLSSARGVTFSRGWLPFLPTSDPPAHTRMRKQLAPGMARGALETWRPMV

DQLARELVGGLLTQTPADVVSTVAAPMPMRAITSVLGVDGPDEAAFCRLS

NQAVRITDVALSASGLISLVQGFAGFRRLRALFTHRRDNGLLRECTVLGK

LATHAEQGRLSDDELFFFAVLLLVAGYESTAHMISTLFLTLADYPDQLTL

LAQQPDLIPSAIEEHLRFISPIQNICRTTRVDYSVGQAVIPAGSLVLLAW

GAANRDPRQYEDPDVFRADRNPVGHLAFGSGIHLCPGTQLARMEGQAILR

EIVANIDRIEVVEPPTWTTNANLRGLTRLRVAVTPRVAP

>CYP128A1 (2584197042) Mycobacterium africanum K85

MTATQSPPEPAPDRVRLAGCPLAGTPDVGLTAQDATTALGVPTRRRASSG

GIPVATSMWRDAQTVRTYGPAVAKALALRVAGKARSRLTGRHCRKFMQLT

DFDPFDPAIAADPYPHYRELLAGERVQYNPKRDVYILSRYADVREAARNH

DTLSSARGVTFSRGWLPFLPTSDPPAHTRMRKQLAPGMARGALETWRPMV

DQLARELVGGLLTQTPADVVSTVAAPMPMRAITSVLGVDGPDEAAFCRLS

NQAVRITDVALSASGLISLVQGFAGFRRLRALFTHRRDNGLLRECTVLGK

LATHAEQGRLSDDELFFFAVLLLVAGYESTAHMISTLFLTLADYPDQLTL

LAQQPDLIPSAIEEHLRFISPIQNICRTTRVDYSVGQAVIPAGSLVLLAW

GAANRDPRQYEDPDVFRADRNPVGHLAFGSGIHLCPGTQLARMEGQAILR

EIVANIDRIEVVEPPTWTTNANLRGLTRLRVAVTPRVAP

>CYP128A1 (2588539573) Mycobacterium tuberculosis K

MTATQSPPEPAPDRVRLAGCPLAGTPDVGLTAQDATTALGVPTRRRASSG

GIPVATSMWRDAQTVRTYGPAVAKALALRVAGKARSRLTGRHCRKFMQLT

DFDPFDPAIAADPYPHYRELLAGERVQYNPKRDVYILSRYADVREAARNH

DTLSSARGVTFSRGWLPFLPTSDPPAHTRMRKQLAPGMARGALETWRPMV

DQLARELVGGLLTQTPADVVSTVAAPMPMRAITSVLGVDGPDEAAFCRLS

NQAVRITDVALSASGLISLVQGFAGFRRLRALFTHRRDNGLLRECTVLGK

LATHAEQGRLSDDELFFFAVLLLVAGYESTAHMISTLFLTLADYPDQLTL

LAQQPDLIPSAIEEHLRFISPIQNICRTTRVDYSVGQAVIPAGSLVLLAW

GAANRDPRQYEDPDVFRADRNPVGHLAFGSGIHLCPGTQLARMEGQAILR

EIVANIDRIEVVEPPTWTTNANLRGLTRLRVAVTPRVAP

>CYP128A1 (2590076507) Mycobacterium tuberculosis MAL010134

MTATQSPPEPAPDRVRLAGCPLAGTPDVGLTAQDATTALGVPTRRRASSG

GIPVATSMWRDAQTVRTYGPAVAKALALRVAGKARSRLTGRHCRKFMQLT

DFDPFDPAIAADPYPHYRELLAGERVQYNPKRDVYILSRYADVREAARNH

DTLSSARGVTFSRGWLPFLPTSDPPAHTRMRKQLAPGMARGALETWRPMV

DQLARELVGGLLTQTPADVVSTVAAPMPMRAITSVLGVDGPDEAAFCRLS

NQAVRITDVALSASGLISLVQGFAGFRRLRALFTHRRDNGLLRECTVLGK

LATHAEQGRLSDDELFFFAVLLLVAGYESTAHMISTLFLTLADYPDQLTL

LAQQPDLIPSAIEEHLRFISPIQNICRTTRVDYSVGQAVIPAGSLVLLAW

GAANRDPRQYEDPDVFRADRNPVGHLAFGSGIHLCPGTQLARMEGQAILR

EIVANIDRIEVVEPPTWTTNANLRGLTRLRVAVTPRVAP

>CYP128A1 (2621490091) Mycobacterium tuberculosis XTB13-158

MTATQSPPEPAPDRVRLAGCPLAGTPDVGLTAQDATTALGVPTRRRASSG

GIPVATSMWRDAQTVRTYGPAVAKALALRVAGKARSRLTGRHCRKFMQLT

DFDPFDPAIAADPYPHYRELLAGERVQYNPKRDVYILSRYADVREAARNH

DTLSSARGVTFSRGWLPFLPTSDPPAHTRMRKQLAPGMARGALETWRPMV

DQLARELVGGLLTQTPADVVSTVAAPMPMRAITSVLGVDGPDEAAFCRLS

NQAVRITDVALSASGLISLVQGFAGFRRLRALFTHRRDNGLLRECTVLGK

LATHAEQGRLSDDELFFFAVLLLVAGYESTAHMISTLFLTLADYPDQLTL

LAQQPDLIPSAIEEHLRFISPIQNICRTTRVDYSVGQAVIPAGSLVLLAW

GAANRDPRQYEDPDVFRADRNPVGHLAFGSGIHLCPGTQLARMEGQAILR

EIVANIDRIEVVEPPTWTTNANLRGLTRLRVAVTPRVAP

>CYP128A1 (2622147172) Mycobacterium tuberculosis MD16568

MTATQSPPEPAPDRVRLAGCPLAGTPDVGLTAQDATTALGVPTRRRASSG

GIPVATSMWRDAQTVRTYGPAVAKALALRVAGKARSRLTGRHCRKFMQLT

DFDPFDPAIAADPYPHYRELLAGERVQYNPKRDVYILSRYADVREAARNH

DTLSSARGVTFSRGWLPFLPTSDPPAHTRMRKQLAPGMARGALETWRPMV

DQLARELVGGLLTQTPADVVSTVAAPMPMRAITSVLGVDGPDEAAFCRLS

NQAVRITDVALSASGLISLVQGFAGFRRLRALFTHRRDNGLLRECTVLGK

LATHAEQGRLSDDELFFFAVLLLVAGYESTAHMISTLFLTLADYPDQLTL

LAQQPDLIPSAIEEHLRFISPIQNICRTTRVDYSVGQAVIPAGSLVLLAW

GAANRDPRQYEDPDVFRADRNPVGHLAFGSGIHLCPGTQLARMEGQAILR

EIVANIDRIEVVEPPTWTTNANLRGLTRLRVAVTPRVAP

>CYP128A1 (2630849232) Mycobacterium tuberculosis TKK_04_0081

MTATQSPPEPAPDRVRLAGCPLAGTPDVGLTAQDATTALGVPTRRRASSG

GIPVATSMWRDAQTVRTYGPAVAKALALRVAGKARSRLTGRHCRKFMQLT

DFDPFDPAIAADPYPHYRELLAGERVQYNPKRDVYILSRYADVREAARNH

DTLSSARGVTFSRGWLPFLPTSDPPAHTRMRKQLAPGMARGALETWRPMV

DQLARELVGGLLTQTPADVVSTVAAPMPMRAITSVLGVDGPDEAAFCRLS

NQAVRITDVALSASGLISLVQGFAGFRRLRALFTHRRDNGLLRECTVLGK

LATHAEQGRLSDDELFFFAVLLLVAGYESTAHMISTLFLTLADYPDQLTL

LAQQPDLIPSAIEEHLRFISPIQNICRTTRVDYSVGQAVIPAGSLVLLAW

GAANRDPRQYEDPDVFRADRNPVGHLAFGSGIHLCPGTQLARMEGQAILR

EIVANIDRIEVVEPPTWTTNANLRGLTRLRVAVTPRVAP

>CYP128A1 (2639514609) Mycobacterium tuberculosis M2032

MTATQSPPEPAPDRVRLAGCPLAGTPDVGLTAQDATTALGVPTRRRASSG

GIPVATSMWRDAQTVRTYGPAVAKALALRVAGKARSRLTGRHCRKFMQLT

DFDPFDPAIAADPYPHYRELLAGERVQYNPKRDVYILSRYADVREAARNH

DTLSSARGVTFSRGWLPFLPTSDPPAHTRMRKQLAPGMARGALETWRPMV

DQLARELVGGLLTQTPADVVSTVAAPMPMRAITSVLGVDGPDEAAFCRLS

NQAVRITDVALSASGLISLVQGFAGFRRLRALFTHRRDNGLLRECTVLGK

LATHAEQGRLSDDELFFFAVLLLVAGYESTAHMISTLFLTLADYPDQLTL

LAQQPDLIPSAIEEHLRFISPIQNICRTTRVDYSVGQAVIPAGSLVLLAW

GAANRDPRQYEDPDVFRADRNPVGHLAFGSGIHLCPGTQLARMEGQAILR

EIVANIDRIEVVEPPTWTTNANLRGLTRLRVAVTPRVAP

>CYP128A1 (2674052595) Mycobacterium tuberculosis H09601792

MTATQSPPEPAPDRVRLAGCPLAGTPDVGLTAQDATTALGVPTRRRASSG

GIPVATSMWRDAQTVRTYGPAVAKALALRVAGKARSRLTGRHCRKFMQLT

DFDPFDPAIAADPYPHYRELLAGERVQYNPKRDVYILSRYADVREAARNH

DTLSSARGVTFSRGWLPFLPTSDPPAHTRMRKQLAPGMARGALETWRPMV

DQLARELVGGLLTQTPADVVSTVAAPMPMRAITSVLGVDGPDEAAFCRLS

NQAVRITDVALSASGLISLVQGFAGFRRLRALFTHRRDNGLLRECTVLGK

LATHAEQGRLSDDELFFFAVLLLVAGYESTAHMISTLFLTLADYPDQLTL

LAQQPDLIPSAIEEHLRFISPIQNICRTTRVDYSVGQAVIPAGSLVLLAW

GAANRDPRQYEDPDVFRADRNPVGHLAFGSGIHLCPGPSWRAWRVRRSCA

RSSPISTE

>CYP128A1 (2703549676) Mycobacterium tuberculosis R00301759

MTATQSPPEPAPDRVRLAGCPLAGTPDVGLTAQDATTALGVPTRRRASSG

GIPVATSMWRDAQTVRTYGPAVAKALALRVAGKARSRLTGRHCRKFMQLT

DFDPFDPAIAADPYPHYRELLAGERVQYNPKRDVYILSRYADVREAARNH

DTLSSARGVTFSRGWLPFLPTSDPPAHTRMRKQLAPGMARGALETWRPMV

DQLARELVGGLLTQTPADVVSTVAAPMPMRAITSVLGVDGPDEAAFCRLS

NQAVRITDVALSASGLISLVQGFAGFRRLRALFTHRRDNGLLRECTVLGK

LATHAEQGRLSDDELFFFAVLLLVAGYESTAHMISTLFLTLADYPDQLTL

LAQQPDLIPSAIEEHLRFISPIQNICRTTRVDYSVGQAVIPAGSLVLLAW

GAANRDPRQYEDPDVFRADRNPVGHLAFGSGIHLCPGTQLARMEGQAILR

EIVANIDRIEVVEPPTWTTNANLRGLTRLRVAVTPRVAP

>CYP128A1 (2721365624) Mycobacterium tuberculosis DK9897

MTATQSPPEPAPDRVRLAGCPLAGTPDVGLTAQDATTALGVPTRRRASSG

GIPVATSMWRDAQTVRTYGPAVAKALALRVAGKARSRLTGRHCRKFMQLT

DFDPFDPAIAADPYPHYRELLAGERVQYNPKRDVYILSRYADVREAARNH

DTLSSARGVTFSRGWLPFLPTSDPPAHTRMRKQLAPGMARGALETWRPMV

DQLARELVGGLLTQTPADVVSTVAAPMPMRAITSVLGVDGPDEAAFCRLS

NQAVRITDVALSASGLISLVQGFAGFRRLRALFTHRRDNGLLRECTVLGK

LATHAEQGRLSDDELFFFAVLLLVAGYESTAHMISTLFLTLADYPDQLTL

LAQQPDLIPSAIEEHLRFISPIQNICRTTRVDYSVGQAVIPAGSLVLLAW

GAANRDPRQYEDPDVFRADRNPVGHLAFGSGIHLCPGTQLARMEGQAILR

EIVANIDRIEVVEPPTWTTNANLRGLTRLRVAVTPRVAP

>CYP128A1 (640603013) Mycobacterium tuberculosis H37Ra

MTATQSPPEPAPDRVRLAGCPLAGTPDVGLTAQDATTALGVPTRRRASSG

GIPVATSMWRDAQTVRTYGPAVAKALALRVAGKARSRLTGRHCRKFMQLT

DFDPFDPAIAADPYPHYRELLAGERVQYNPKRDVYILSRYADVREAARNH

DTLSSARGVTFSRGWLPFLPTSDPPAHTRMRKQLAPGMARGALETWRPMV

DQLARELVGGLLTQTPADVVSTVAAPMPMRAITSVLGVDGPDEAAFCRLS

NQAVRITDVALSASGLISLVQGFAGFRRLRALFTHRRDNGLLRECTVLGK

LATHAEQGRLSDDELFFFAVLLLVAGYESTAHMISTLFLTLADYPDQLTL

LAQQPDLIPSAIEEHLRFISPIQNICRTTRVDYSVGQAVIPAGSLVLLAW

GAANRDPRQYEDPDVFRADRNPVGHLAFGSGIHLCPGTQLARMEGQAILR

EIVANIDRIEVVEPPTWTTNANLRGLTRLRVAVTPRVAP

>CYP128A1 (2622362621) Mycobacterium tuberculosis M1029

MTATQSPPEPAPDRVRLAGCPLAGTPDVGLTAQDATTALGVPTRRRASSG

GIPVATSMWRDAQTVRTYGPAVAKALALRVAGKARSRLTGRHCRKFMQLT

DFDPFDPAIAADPYPHYRELLAGERVQYNPKRDVYILSRYADVREAARNH

DTLSSSRGVTFSRGWLPFLPTSDPPAHTRMRKQLAPGMARGALETWRPMV

DQLARELVGGLLTQTPADVVSTVAAPMPMRAITSVLGVDGPDEAAFCRLS

NQAVRITDVALSASGLISLVQGFAGFRRLRALFTHRRDNGLLRECTVLGK

LATHAEQGRLSDDELFFFAVLLLVAGYESTAHMISTLFLTLADYPDQLTL

LAQQPDLIPSAIEEHLRFISPIQNICRTTRVDYSVGQAVIPAGSLVLLAW

GAANRDPRQYEDPDVFRADRNPVGHLAFGSGIHLCPGTQLARMEGQAILR

EIVANIDRIEVVEPPTWTTNANLRGLTRLRVAVTPRVAP

>CYP128B4 (2802951330) Mycobacteroides abscessus abscessus 1054

MSRWRSDSAVRQIGAPVVAAVGMNIAAAVRVRRRGYAGWTGAVNTDYDPL

DPATAAQPFDAYRALHAGGRVHYNPKRATFILSRHEDIRAALRDTDAVTS

SQGVTRMKISAPILVLTDGDDHTRLRKQVQPGFTRGAMSDWQGMADQLAK

ELVADVVANPGCDVMERLAVPLPIRMIAHIIGIPPEDVQNFRSWSEDGVG

VINAGVSPAGLRQGLKGVRAIAALRRYFKDQLASGKLKGSDTVLGRLVDN

NEDGKLSDDELFFIAMLLLFAGNETTTNLIGGMFDTLAHAPDQFAMIRDD

PDLIPSAVEEQLRYSAPIQNLYRYTRTDYRVGEVTIPSGSRLLLAFGAAN

RDPEVFEDPDTYRADRNPRNHIAFGYGVHMCIGATLSRMEGQAVLRELTS

QASAIAAAGSATWSTNSSLRGTTYLPIRLTPAR

>CYP128B2 (2725155723) Mycobacterium avium hominissuis MAH-P-7673-04

LTLSQDVRFLGTMAQPLSHAARVNTARAFRSRWRQYRFWTGAQITDYDPI

DPANIAQPDAAYRALHAGGRVHYNPKLGLWILTRLPDVRAGARAAETLSS

ADGVTRLRMAGPLLVTMDGKPHNEMRRHVLPAFTKAALESWQAMIDELAA

KLVGEVLDNPGCDVVQRLAIPMPMLLIAHMLGIPNGDIDDFRRWSSDAVK

MADVDISRRGLTRLKSSIGGIRDIYRYFRQQFAVGGLKGSDTLLGKLLSV

NASGSIDDNELFFFAMLLLIAGNETTTNLLGGMFDIFARHPEKFDMVRAD

HSLIPKVVEEQLRFSSPVQMLYRTARTNYEVGPVTIPAGARVLLSLGAAN

RDPQVFDEPDEFRVDRNPTEHLAFGFGAHLCLGAQLTRMEAQAVLRELVT

RARRIEAIGETQWSTGYLLRGPERMNVRLTPSVVAG

>CYP128A1 (2584646498) Mycobacterium tuberculosis TKK-01-0038

MTATQSPPEPAPDRVRLAGCPLAGTPDVGLTAQDATTALGVPTRRRASSG

GIPVATSMWRDAQTVRTYGPAVAKALALRVAGKARSRLTGRHCRKFMQLT

DFDPFDPAIAADPYPHYRELLAGERVQYNPKRDVYILSRYADVREAARNH

DTLSSARGVTFSRGWLPFLPTSDPPAHTRMRKQLAPGMARGALETWRPMV

DQLARELVGGLLTQTPADVVSTVAAPMPMRAITSVLGVDGPDEAAFCRLS

NQAVRITDVALSASGLISLVQGFAGFRRLRALFTHRRDNGLLRECTVLGK

LATHAEQGRLSDDELFFFAVLLLVAGYESTAHMISTLFLTLADYPDQLTL

LAQQPDLIPSAIEEHLRFISPIQNICRTTRVDYSVGQAVIPAGSLVLLAW

GAANRDPRQYEDPDVFRADRNPVGHLAFGSGIHLCPGTQLARMEGQAILR

EIVANIDRIEVVEPPTWTTNANLRGLTRLRVAVTPRVAP

>CYP128A1 (2589065223) Mycobacterium tuberculosis TBR40

MTATQSPPEPAPDRVRLAGCPLAGTPDVGLTAQDATTALGVPTRRRASSG

GIPVATSMWRDAQTVRTYGPAVAKALALRVAGKARSRLTGRHCRKFMQLT

DFDPFDPAIAADPYPHYRELLAGERVQYNPKRDVYILSRYADVREAARNH

DTLSSARGVTFSRGWLPFLPTSDPPAHTRMRKQLAPGMARGALETWRPMV

DQLARELVGGLLTQTPADVVSTVAAPMPMRAITSVLGVDGPDEAAFCRLS

NQAVRITDVALSASGLISLVQGFAGFRRLRALFTHRRDNGLLRECTVLGK

LATHAEQGRLSDDELFFFAVLLLVAGYESTAHMISTLFLTLADYPDQLTL

LAQQPDLIPSAIEEHLRFISPIQNICRTTRVDYSVGQAVIPAGSLVLLAW

GAANRDPRQYEDPDVFRADRNPVGHLAFGSGIHLCPGTQLARMEGQAILR

EIVANIDRIEVVEPPTWTTNANLRGLTRLRVAVTPRVAP

>CYP128A1 (2589114193) Mycobacterium tuberculosis TBR66

MTATQSPPEPAPDRVRLAGCPLAGTPDVGLTAQDATTALGVPTRRRASSG

GIPVATSMWRDAQTVRTYGPAVAKALALRVAGKARSRLTGRHCRKFMQLT

DFDPFDPAIAADPYPHYRELLAGERVQYNPKRDVYILSRYADVREAARNH

DTLSSARGVTFSRGWLPFLPTSDPPAHTRMRKQLAPGMARGALETWRPMV

DQLARELVGGLLTQTPADVVSTVAAPMPMRAITSVLGVDGPDEAAFCRLS

NQAVRITDVALSASGLISLVQGFAGFRRLRALFTHRRDNGLLRECTVLGK

LATHAEQGRLSDDELFFFAVLLLVAGYESTAHMISTLFLTLADYPDQLTL

LAQQPDLIPSAIEEHLRFISPIQNICRTTRVDYSVGQAVIPAGSLVLLAW

GAANRDPRQYEDPDVFRADRNPVGHLAFGSGIHLCPGTQLARMEGQAILR

EIVANIDRIEVVEPPTWTTNANLRGLTRLRVAVTPRVAP

>CYP128A1 (2590072686) Mycobacterium tuberculosis MAL020102

MTATQSPPEPAPDRVRLAGCPLAGTPDVGLTAQDATTALGVPTRRRASSG

GIPVATSMWRDAQTVRTYGPAVAKALALRVAGKARSRLTGRHCRKFMQLT

DFDPFDPAIAADPYPHYRELLAGERVQYNPKRDVYILSRYADVREAARNH

DTLSSARGVTFSRGWLPFLPTSDPPAHTRMRKQLAPGMARGALETWRPMV

DQLARELVGGLLTQTPADVVSTVAAPMPMRAITSVLGVDGPDEAAFCRLS

NQAVRITDVALSASGLISLVQGFAGFRRLRALFTHRRDNGLLRECTVLGK

LATHAEQGRLSDDELFFFAVLLLVAGYESTAHMISTLFLTLADYPDQLTL

LAQQPDLIPSAIEEHLRFISPIQNICRTTRVDYSVGQAVIPAGSLVLLAW

GAANRDPRQYEDPDVFRADRNPVGHLAFGSGIHLCPGTQLARMEGQAILR

EIVANIDRIEVVEPPTWTTNANLRGLTRLRVAVTPRVAP

>CYP128A1 (2590248851) Mycobacterium tuberculosis KT-0028

MTATQSPPEPAPDRVRLAGCPLAGTPDVGLTAQDATTALGVPTRRRASSG

GIPVATSMWRDAQTVRTYGPAVAKALALRVAGKARSRLTGRHCRKFMQLT

DFDPFDPAIAADPYPHYRELLAGERVQYNPKRDVYILSRYADVREAARNH

DTLSSARGVTFSRGWLPFLPTSDPPAHTRMRKQLAPGMARGALETWRPMV

DQLARELVGGLLTQTPADVVSTVAAPMPMRAITSVLGVDGPDEAAFCRLS

NQAVRITDVALSASGLISLVQGFAGFRRLRALFTHRRDNGLLRECTVLGK

LATHAEQGRLSDDELFFFAVLLLVAGYESTAHMISTLFLTLADYPDQLTL

LAQQPDLIPSAIEEHLRFISPIQNICRTTRVDYSVGQAVIPAGSLVLLAW

GAANRDPRQYEDPDVFRADRNPVGHLAFGSGIHLCPGTQLARMEGQAILR

EIVANIDRIEVVEPPTWTTNANLRGLTRLRVAVTPRVAP

>CYP128A1 (2604014426) Mycobacterium tuberculosis TB_RSA60

MTATQSPPEPAPDRVRLAGCPLAGTPDVGLTAQDATTALGVPTRRRASSG

GIPVATSMWRDAQTVRTYGPAVAKALALRVAGKARSRLTGRHCRKFMQLT

DFDPFDPAIAADPYPHYRELLAGERVQYNPKRDVYILSRYADVREAARNH

DTLSSARGVTFSRGWLPFLPTSDPPAHTRMRKQLAPGMARGALETWRPMV

DQLARELVGGLLTQTPADVVSTVAAPMPMRAITSVLGVDGPDEAAFCRLS

NQAVRITDVALSASGLISLVQGFAGFRRLRALFTHRRDNGLLRECTVLGK

LATHAEQGRLSDDELFFFAVLLLVAGYESTAHMISTLFLTLADYPDQLTL

LAQQPDLIPSAIEEHLRFISPIQNICRTTRVDYSVGQAVIPAGSLVLLAW

GAANRDPRQYEDPDVFRADRNPVGHLAFGSGIHLCPGTQLARMEGQAILR

EIVANIDRIEVVEPPTWTTNANLRGLTRLRVAVTPRVAP

>CYP128A1 (2606463806) Mycobacterium tuberculosis XTB13-253

MTATQSPPEPAPDRVRLAGCPLAGTPDVGLTAQDATTALGVPTRRRASSG

GIPVATSMWRDAQTVRTYGPAVAKALALRVAGKARSRLTGRHCRKFMQLT

DFDPFDPAIAADPYPHYRELLAGERVQYNPKRDVYILSRYADVREAARNH

DTLSSARGVTFSRGWLPFLPTSDPPAHTRMRKQLAPGMARGALETWRPMV

DQLARELVGGLLTQTPADVVSTVAAPMPMRAITSVLGVDGPDEAAFCRLS

NQAVRITDVALSASGLISLVQGFAGFRRLRALFTHRRDNGLLRECTVLGK

LATHAEQGRLSDDELFFFAVLLLVAGYESTAHMISTLFLTLADYPDQLTL

LAQQPDLIPSAIEEHLRFISPIQNICRTTRVDYSVGQAVIPAGSLVLLAW

GAANRDPRQYEDPDVFRADRNPVGHLAFGSGIHLCPGPSWRAWRVRRSCA

RSSPISTE

>CYP128A1 (2622396675) Mycobacterium tuberculosis M1253

MTATQSPPEPAPDRVRLAGCPLAGTPDVGLTAQDATTALGVPTRRRASSG

GIPVATSMWRDAQTVRTYGPAVAKALALRVAGKARSRLTGRHCRKFMQLT

DFDPFDPAIAADPYPHYRELLAGERVQYNPKRDVYILSRYADVREAARNH

DTLSSARGVTFSRGWLPFLPTSDPPAHTRMRKQLAPGMARGALETWRPMV

DQLARELVGGLLTQTPADVVSTVAAPMPMRAITSVLGVDGPDEAAFCRLS

NQAVRITDVALSASGLISLVQGFAGFRRLRALFTHRRDNGLLRECTVLGK

LATHAEQGRLSDDELFFFAVLLLVAGYESTAHMISTLFLTLADYPDQLTL

LAQQPDLIPSAIEEHLRFISPIQNICRTTRVDYSVGQAVIPAGSLVLLAW

GAANRDPRQYEDPDVFRADRNPVGHLAFGSGIHLCPGTQLARMEGQAILR

EIVANIDRIEVVEPPTWTTNANLRGLTRLRVAVTPRVAP

>CYP128A1 (2632105812) Mycobacterium tuberculosis NRITLD53

MTATQSPPEPAPDRVRLAGCPLAGTPDVGLTAQDATTALGVPTRRRASSG

GIPVATSMWRDAQTVRTYGPAVAKALALRVAGKARSRLTGRHCRKFMQLT

DFDPFDPAIAADPYPHYRELLAGERVQYNPKRDVYILSRYADVREAARNH

DTLSSARGVTFSRGWLPFLPTSDPPAHTRMRKQLAPGMARGALETWRPMV

DQLARELVGGLLTQTPADVVSTVAAPMPMRAITSVLGVDGPDEAAFCRLS

NQAVRITDVALSASGLISLVQGFAGFRRLRALFTHRRDNGLLRECTVLGK

LATHAEQGRLSDDELFFFAVLLLVAGYESTAHMISTLFLTLADYPDQLTL

LAQQPDLIPSAIEEHLRFISPIQNICRTTRVDYSVGQAVIPAGSLVLLAW

GAANRDPRQYEDPDVFRADRNPVGHLAFGSGIHLCPGTQLARMEGQAILR

EIVANIDRIEVVEPPTWTTNANLRGLTRLRVAVTPRVAP

>CYP128A1 (2636427467) Mycobacterium tuberculosis KT-0192

MTATQSPPEPAPDRVRLAGCPLAGTPDVGLTAQDATTALGVPTRRRASSG

GIPVATSMWRDAQTVRTYGPAVAKALALRVAGKARSRLTGRHCRKFMQLT

DFDPFDPAIAADPYPHYRELLAGERVQYNPKRDVYILSRYADVREAARNH

DTLSSARGVTFSRGWLPFLPTSDPPAHTRMRKQLAPGMARGALETWRPMV

DQLARELVGGLLTQTPADVVSTVAAPMPMRAITSVLGVDGPDEAAFCRLS

NQAVRITDVALSASGLISLVQGFAGFRRLRALFTHRRDNGLLRECTVLGK

LATHAEQGRLSDDELFFFAVLLLVAGYESTAHMISTLFLTLADYPDQLTL

LAQQPDLIPSAIEEHLRFISPIQNICRTTRVDYSVGQAVIPAGSLVLLAW

GAANRDPRQYEDPDVFRADRNPVGHLAFGSGIHLCPGTQLARMEGQAILR

EIVANIDRIEVVEPPTWTTNANLRGLTRLRVAVTPRVAP

>CYP128A1 (2651420867) Mycobacterium tuberculosis VRFCWCF PRTB19

MTATQSPPEPAPDRVRLAGCPLAGTPDVGLTAQDATTALGVPTRRRASSG

GIPVATSMWRDAQTVRTYGPAVAKALALRVAGKARSRLTGRHCRKFMQLT

DFDPFDPAIAADPYPHYRELLAGERVQYNPKRDVYILSRYADVREAARNH

DTLSSARGVTFSRGWLPFLPTSDPPAHTRMRKQLAPGMARGALETWRPMV

DQLARELVGGLLTQTPADVVSTVAAPMPMRAITSVLGVDGPDEAAFCRLS

NQAVRITDVALSASGLISLVQGFAGFRRLRALFTHRRDNGLLRECTVLGK

LATHAEQGRLSDDELFFFAVLLLVAGYESTAHMISTLFLTLADYPDQLTL

LAQQPDLIPSAIEEHLRFISPIQNICRTTRVDYSVGQAVIPAGSLVLLAW

GAANRDPRQYEDPDVFRADRNPVGHLAFGSGIHLCPGTQLARMEGQAILR

EIVANIDRIEVVEPPTWTTNANLRGLTRLRVAVTPRVAP

>CYP128A1 (651025744) Mycobacterium africanum GM041182

MTATQSPPEPAPDRVRLAGCPLAGTPDVGLTAQDATTALGVPTRRRASSG

GIPVATSMWRDAQTVRTYGPAVAKALALRVAGKARSRLTGRHCRKFMQLT

DFDPFDPAIAADPYPHYRELLAGERVQYNPKRDVYILSRYADVREAARNH

DTLSSARGVTFSRGWLPFLPTSDPPAHTRMRKQLAPGMARGALETWRPMV

DQLARELVGGLLTQTPADVVSTVAAPMPMRAITSVLGVDGPDEAAFCRLS

NQAVRITDVALSASGLISLVQGFAGFRRLRALFTHRRDNGLLRECTVLGK

LATHAEQGRLSDDELFFFAVLLLVAGYESTAHMISTLFLTLADYPDQLTL

LAQQPDLIPSAIEEHLRFISPIQNICRTTRVDYSVGQAVIPAGSLVLLAW

GAANRDPRQYEDPDVFRADRNPVGHLAFGSGIHLCPGTQLARMEGQAILR

EIVANIDRIEVVEPPTWTTNANLRGLTRLRVAVTPRVAP

>CYP128A1 (2576709444) Mycobacterium tuberculosis TKK_03_0090

MTATQSPPEPAPDRVRLAGCPLAGTPDVGLTAQDATTALGVPTRRRASSG

GIPVATSMWRDAQTVRTYGPAVAKALALRVAGKARSRLTGRHCRKFMQLT

DFDPFDPAIAADPYPHYRELLAGERVQYNPKRDVYILSRYADVREAARNH

DTLSSARGVTFSRGWLPFLPTSDPPAHTRMRKQLAPGMARGALETWRPMV

DQLARELVGGLLTQTPADVVSTVAAPMPMRAITSVLGVDGPDEAAFCRLS

NQAVRITDVALSASGLISLVQGFAGFRRLRALFTHRRDNGLLRECTVLGK

LATHAEQGRLSDDELFFFAVLLLVAGYESPAHMISTLFLTLADYPDQLTL

LAQQPDLIPSAIEEHLRFISPIQNICRTTRVDYSVGQAVIPAGSLVLLAW

GAANRDPRQYEDPDVFRADRNPVGHLAFGSGIHLCPGTQLARMEGQAILR

EIVANIDRIEVVEPPTWTTNANLRGLTRLRVAVTPRVAP

>CYP128A1 (2578040602) Mycobacterium tuberculosis TKK_05SA_0017

MTATQSPPEPAPDRVRLAGCPLAGTPDVGLTAQDATTALGVPTRRRASSG

GIPVATSMWRDAQTVRTYGPAVAKALALRVAGKARSRLTGRHCRKFMQLT

DFDPFDPAIAADPYPHYRELLAGERVQYNPKRDVYILSRYADVREAARNH

DTLSSARGVTFSRGWLPFLPTSDPPAHTRMRKQLAPGMARGALETWRPMV

DQLARELVGGLLTQTPADVVSTVAAPMPMRAITSVLGVDGPDEAAFCRLS

NQAVRITDVALSASGLISLVQGFAGFRRLRALFTHRRDNGLLRECTVLGK

LATHAEQGRLSDDELFFFAVLLLVAGYESPAHMISTLFLTLADYPDQLTL

LAQQPDLIPSAIEEHLRFISPIQNICRTTRVDYSVGQAVIPAGSLVLLAW

GAANRDPRQYEDPDVFRADRNPVGHLAFGSGIHLCPGTQLARMEGQAILR

EIVANIDRIEVVEPPTWTTNANLRGLTRLRVAVTPRVAP

>CYP128A1 (2584659819) Mycobacterium tuberculosis XTB13-194

MTATQSPPEPAPDRVRLAGCPLAGTPDVGLTAQDATTALGVPTRRRAASG

GIPVATSMWRDAQTVRTYGPAVAKALALRVAGKARSRLTGRHCRKFMQLT

DFDPFDPAIAADPYPHYRELLAGERVQYNPKRDVYILSRYADVREAARNH

DTLSSARGVTFSRGWLPFLPTSDPPAHTRMRKQLAPGMARGALETWRPMV

DQLARELVGGLLTQTPADVVSTVAAPMPMRAITSVLGVDGPDEAAFCRLS

NQAVRITDVALSASGLISLVQGFAGFRRLRALFTHRRDNGLLRECTVLGK

LATHAEQGRLSDDELFFFAVLLLVAGYESTAHMISTLFLTLADYPDQLTL

LAQQPDLIPSAIEEHLRFISPIQNICRTTRVDYSVGQAVIPAGSLVLLAW

GAANRDPRQYEDPDVFRADRNPVGHLAFGSGIHLCPGTQLARMEGQAILR

EIVANIDRIEVVEPPTWTTNANLRGLTRLRVAVTPRVAP

>CYP128A1 (2622639234) Mycobacterium tuberculosis M1734

MTATQSPPEPAPDRVRLAGCPLAGTPDVGLTAQDATTALGVPTRRRASSG

GIPVATSMWRDAQTVRTYGPAVAKALALRVAGKARSRLTGRHCRKFMQLT

DFDPFDPAIAADPYPHYRELLAGERVQYNPKRDVYILSRYADVREAARNH

DTLSSSRGVTFSRGWLPFLPTSDPPAHTRMRKQLAPGMARGALETWRPMV

DQLARELVGGLLTQTPADVVSTVAAPMPMRAITSVLGVDGPDEAAFCRLS

NQAVRITDVALSASGLISLVQGFAGFRRLRALFTHRRDNGLLRECTVLGK

LATHAEQGRLSDDELFFFAVLLLVAGYESTAHMISTLFLTLADYPDQLTL

LAQQPDLIPSAIEEHLRFISPIQNICRTTRVDYSVGQAVIPAGSLVLLAW

GAANRDPRQYEDPDVFRADRNPVGHLAFGSGIHLCPGTQLARMEGQAILR

EIVANIDRIEVVEPPTWTTNANLRGLTRLRVAVTPRVAP

>CYP128A1 (2630499619) Mycobacterium tuberculosis TKK_03_0111

MTATQSPPEPAPDRVRLAGCPLAGTPDVGLTAQDATTALGVPTRRRASSG

GIPVATSMWRDAQTVRTYGPAVAKALALRVAGKARSRLTGRHCRKFMQLT

DFDPFDPAIAADPYPHYRELLAGERVQYNPKRDVYILSRYADVREAARNH

DTLSSARGVTFSRGWLPFLPTSDPPAHTRMRKQLAPGMARGALETWRPMV

DQLARELVGGLLTQTPADVVSTVAAPMPMRAITSVLGVDGPDEAAFCRLS

NQAVRITDVALSASGLISLVQGFAGFRRLRALFTHRRDNGLLRECTVLGK

LATHAEQGRLSDDELFFFAVLLLVAGYESPAHMISTLFLTLADYPDQLTL

LAQQPDLIPSAIEEHLRFISPIQNICRTTRVDYSVGQAVIPAGSLVLLAW

GAANRDPRQYEDPDVFRADRNPVGHLAFGSGIHLCPGTQLARMEGQAILR

EIVANIDRIEVVEPPTWTTNANLRGLTRLRVAVTPRVAP

>CYP128A1 (2555525016) Mycobacterium tuberculosis PanR0802

MTATQSPPEPAPDRVRLAGCPLAGTPDVGLTAQDATTALGVPTRRRASSG

GIPVATSMWRDAQTVRTYGPAVAKALALRVAGKARSRLTGRHCRKFMQLT

DFDPFDPAIAADPYPHYRELLAGERVQYNPKRDVYILSRYADVREAARNH

DTLSSARGVTFSRGWLPFLPTSDPPAHTRMRKQLAPGMARGALETWRPMV

DQLARELVGGLLTQTPADVVSTVAAPMPMRAITSVLGVDGPDEAAFCRLS

NQAVRITDVALSASGLISLVQGFAGFRRLRALFTHRRDNGLLRECTVLGK

LATHAEQGRLSDDELFFFAVLLLVAGYESTAHMISTLFLTLADYPDQLTL

LAQQPDLIPSAIEEHLRFISPIQNICRTTRVDYSVGQAVIPAGSLVLLAW

GAANRDPRQYEDPDVFRADRNPVGHLAFGSGIHLCPGTQLARMEGQAILR

EIVANIDRIEVVEPPTWTTNANLRGLTRLRVAVTPRVAP

>CYP128A1 (2575256114) Mycobacterium tuberculosis KT-0004

MTATQSPPEPAPDRVRLAGCPLAGTPDVGLTAQDATTALGVPTRRRASSG

GIPVATSMWRDAQTVRTYGPAVAKALALRVAGKARSRLTGRHCRKFMQLT

DFDPFDPAIAADPYPHYRELLAGERVQYNPKRDVYILSRYADVREAARNH

DTLSSARGVTFSRGWLPFLPTSDPPAHTRMRKQLAPGMARGALETWRPMV

DQLARELVGGLLTQTPADVVSTVAAPMPMRAITSVLGVDGPDEAAFCRLS

NQAVRITDVALSASGLISLVQGFAGFRRLRALFTHRRDNGLLRECTVLGK

LATHAEQGRLSDDELFFFAVLLLVAGYESTAHMISTLFLTLADYPDQLTL

LAQQPDLIPSAIEEHLRFISPIQNICRTTRVDYSVGQAVIPAGSLVLLAW

GAANRDPRQYEDPDVFRADRNPVGHLAFGSGIHLCPGTQLARMEGQAILR

EIVANIDRIEVVEPPTWTTNANLRGLTRLRVAVTPRVAP

>CYP128A1 (2579817329) Mycobacterium tuberculosis M1787

MTATQSPPEPAPDRVRLAGCPLAGTPDVGLTAQDATTALGVPTRRRASSG

GIPVATSMWRDAQTVRTYGPAVAKALALRVAGKARSRLTGRHCRKFMQLT

DFDPFDPAIAADPYPHYRELLAGERVQYNPKRDVYILSRYADVREAARNH

DTLSSARGVTFSRGWLPFLPTSDPPAHTRMRKQLAPGMARGALETWRPMV

DQLARELVGGLLTQTPADVVSTVAAPMPMRAITSVLGVDGPDEAAFCRLS

NQAVRITDVALSASGLISLVQGFAGFRRLRALFTHRRDNGLLRECTVLGK

LATHAEQGRLSDDELFFFAVLLLVAGYESTAHMISTLFLTLADYPDQLTL

LAQQPDLIPSAIEEHLRFISPIQNICRTTRVDYSVGQAVIPAGSLVLLAW

GAANRDPRQYEDPDVFRADRNPVGHLAFGSGIHLCPGTQLARMEGQAILR

EIVANIDRIEVVEPPTWTTNANLRGLTRLRVAVTPRVAP

>CYP128A1 (2590560560) Mycobacterium tuberculosis KT-0071

MTATQSPPEPAPDRVRLAGCPLAGTPDVGLTAQDATTALGVPTRRRASSG

GIPVATSMWRDAQTVRTYGPAVAKALALRVAGKARSRLTGRHCRKFMQLT

DFDPFDPAIAADPYPHYRELLAGERVQYNPKRDVYILSRYADVREAARNH

DTLSSARGVTFSRGWLPFLPTSDPPAHTRMRKQLAPGMARGALETWRPMV

DQLARELVGGLLTQTPADVVSTVAAPMPMRAITSVLGVDGPDEAAFCRLS

NQAVRITDVALSASGLISLVQGFAGFRRLRALFTHRRDNGLLRECTVLGK

LATHAEQGRLSDDELFFFAVLLLVAGYESTAHMISTLFLTLADYPDQLTL

LAQQPDLIPSAIEEHLRFISPIQNICRTTRVDYSVGQAVIPAGSLVLLAW

GAANRDPRQYEDPDVFRADRNPVGHLAFGSGIHLCPGTQLARMEGQAILR

EIVANIDRIEVVEPPTWTTNANLRGLTRLRVAVTPRVAP

>CYP128A1 (2603482056) Mycobacterium tuberculosis BTB06-340

MTATQSPPEPAPDRVRLAGCPLAGTPDVGLTAQDATTALGVPTRRRASSG

GIPVATSMWRDAQTVRTYGPAVAKALALRVAGKARSRLTGRHCRKFMQLT

DFDPFDPAIAADPYPHYRELLAGERVQYNPKRDVYILSRYADVREAARNH

DTLSSARGVTFSRGWLPFLPTSDPPAHTRMRKQLAPGMARGALETWRPMV

DQLARELVGGLLTQTPADVVSTVAAPMPMRAITSVLGVDGPDEAAFCRLS

NQAVRITDVALSASGLISLVQGFAGFRRLRALFTHRRDNGLLRECTVLGK

LATHAEQGRLSDDELFFFAVLLLVAGYESTAHMISTLFLTLADYPDQLTL

LAQQPDLIPSAIEEHLRFISPIQNICRTTRVDYSVGQAVIPAGSLVLLAW

GAANRDPRQYEDPDVFRADRNPVGHLAFGSGIHLCPGTQLARMEGQAILR

EIVANIDRIEVVEPPTWTTNANLRGLTRLRVAVTPRVAP

>CYP128A1 (2605745581) Mycobacterium tuberculosis TB_RSA41

MTATQSPPEPAPDRVRLAGCPLAGTPDVGLTAQDATTALGVPTRRRASSG

GIPVATSMWRDAQTVRTYGPAVAKALALRVAGKARSRLTGRHCRKFMQLT

DFDPFDPAIAADPYPHYRELLAGERVQYNPKRDVYILSRYADVREAARNH

DTLSSARGVTFSRGWLPFLPTSDPPAHTRMRKQLAPGMARGALETWRPMV

DQLARELVGGLLTQTPADVVSTVAAPMPMRAITSVLGVDGPDEAAFCRLS

NQAVRITDVALSASGLISLVQGFAGFRRLRALFTHRRDNGLLRECTVLGK

LATHAEQGRLSDDELFFFAVLLLVAGYESTAHMISTLFLTLADYPDQLTL

LAQQPDLIPSAIEEHLRFISPIQNICRTTRVDYSVGQAVIPAGSLVLLAW

GAANRDPRQYEDPDVFRADRNPVGHLAFGSGIHLCPGTQLARMEGQAILR

EIVANIDRIEVVEPPTWTTNANLRGLTRLRVAVTPRVAP

>CYP128A1 (2606487827) Mycobacterium tuberculosis 1429BH

MTATQSPPEPAPDRVRLAGCPLAGTPDVGLTAQDATTALGVPTRRRASSG

GIPVATSMWRDAQTVRTYGPAVAKALALRVAGKARSRLTGRHCRKFMQLT

DFDPFDPAIAADPYPHYRELLAGERVQYNPKRDVYILSRYADVREAARNH

DTLSSARGVTFSRGWLPFLPTSDPPAHTRMRKQLAPGMARGALETWRPMV

DQLARELVGGLLTQTPADVVSTVAAPMPMRAITSVLGVDGPDEAAFCRLS

NQAVRITDVALSASGLISLVQGFAGFRRLRALFTHRRDNGLLRECTVLGK

LATHAEQGRLSDDELFFFAVLLLVAGYESTAHMISTLFLTLADYPDQLTL

LAQQPDLIPSAIEEHLRFISPIQNICRTTRVDYSVGQAVIPAGSLVLLAW

GAANRDPRQYEDPDVFRADRNPVGHLAFGSGIHLCPGTQLARMEGQAILR

EIVANIDRIEVVEPPTWTTNANLRGLTRLRVAVTPRVAP

>CYP128A1 (2622018089) Mycobacterium tuberculosis MD18489

MTATQSPPEPAPDRVRLAGCPLAGTPDVGLTAQDATTALGVPTRRRASSG

GIPVATSMWRDAQTVRTYGPAVAKALALRVAGKARSRLTGRHCRKFMQLT

DFDPFDPAIAADPYPHYRELLAGERVQYNPKRDVYILSRYADVREAARNH

DTLSSARGVTFSRGWLPFLPTSDPPAHTRMRKQLAPGMARGALETWRPMV

DQLARELVGGLLTQTPADVVSTVAAPMPMRAITSVLGVDGPDEAAFCRLS

NQAVRITDVALSASGLISLVQGFAGFRRLRALFTHRRDNGLLRECTVLGK

LATHAEQGRLSDDELFFFAVLLLVAGYESTAHMISTLFLTLADYPDQLTL

LAQQPDLIPSAIEEHLRFISPIQNICRTTRVDYSVGQAVIPAGSLVLLAW

GAANRDPRQYEDPDVFRADRNPVGHLAFGSGIHLCPGTQLARMEGQAILR

EIVANIDRIEVVEPPTWTTNANLRGLTRLRVAVTPRVAP

>CYP128A1 (2661455782) Mycobacterium tuberculosis 302174

MTATQSPPEPAPDRVRLAGCPLAGTPDVGLTAQDATTALGVPTRRRASSG

GIPVATSMWRDAQTVRTYGPAVAKALALRVAGKARSRLTGRHCRKFMQLT

DFDPFDPAIAADPYPHYRELLAGERVQYNPKRDVYILSRYADVREAARNH

DTLSSARGVTFSRGWLPFLPTSDPPAHTRMRKQLAPGMARGALETWRPMV

DQLARELVGGLLTQTPADVVSTVAAPMPMRAITSVLGVDGPDEAAFCRLS

NQAVRITDVALSASGLISLVQGFAGFRRLRALFTHRRDNGLLRECTVLGK

LATHAEQGRLSDDELFFFAVLLLVAGYESTAHMISTLFLTLADYPDQLTL

LAQQPDLIPSAIEEHLRFISPIQNICRTTRVDYSVGQAVIPAGSLVLLAW

GAANRDPRQYEDPDVFRADRNPVGHLAFGSGIHLCPGTQLARMEGQAILR

EIVANIDRIEVVEPPTWTTNANLRGLTRLRVAVTPRVAP

>CYP128A1 (2663319428) Mycobacterium tuberculosis TBR-175

MTATQSPPEPAPDRVRLAGCPLAGTPDVGLTAQDATTALGVPTRRRASSG

GIPVATSMWRDAQTVRTYGPAVAKALALRVAGKARSRLTGRHCRKFMQLT

DFDPFDPAIAADPYPHYRELLAGERVQYNPKRDVYILSRYADVREAARNH

DTLSSARGVTFSRGWLPFLPTSDPPAHTRMRKQLAPGMARGALETWRPMV

DQLARELVGGLLTQTPADVVSTVAAPMPMRAITSVLGVDGPDEAAFCRLS

NQAVRITDVALSASGLISLVQGFAGFRRLRALFTHRRDNGLLRECTVLGK

LATHAEQGRLSDDELFFFAVLLLVAGYESTAHMISTLFLTLADYPDQLTL

LAQQPDLIPSAIEEHLRFISPIQNICRTTRVDYSVGQAVIPAGSLVLLAW

GAANRDPRQYEDPDVFRADRNPVGHLAFGSGIHLCPGTQLARMEGQAILR

EIVANIDRIEVVEPPTWTTNANLRGLTRLRVAVTPRVAP

>CYP128A1 (2670570216) Mycobacterium tuberculosis A70655

MTATQSPPEPAPDRVRLAGCPLAGTPDVGLTAQDATTALGVPTRRRASSG

GIPVATSMWRDAQTVRTYGPAVAKALALRVAGKARSRLTGRHCRKFMQLT

DFDPFDPAIAADPYPHYRELLAGERVQYNPKRDVYILSRYADVREAARNH

DTLSSARGVTFSRGWLPFLPTSDPPAHTRMRKQLAPGMARGALETWRPMV

DQLARELVGGLLTQTPADVVSTVAAPMPMRAITSVLGVDGPDEAAFCRLS

NQAVRITDVALSASGLISLVQGFAGFRRLRALFTHRRDNGLLRECTVLGK

LATHAEQGRLSDDELFFFAVLLLVAGYESTAHMISTLFLTLADYPDQLTL

LAQQPDLIPSAIEEHLRFISPIQNICRTTRVDYSVGQAVIPAGSLVLLAW

GAANRDPRQYEDPDVFRADRNPVGHLAFGSGIHLCPGTQLARMEGQAILR

EIVANIDRIEVVEPPTWTTNANLRGLTRLRVAVTPRVAP

>CYP128A1 (2675698416) Mycobacterium tuberculosis O09700920

MTATQSPPEPAPDRVRLAGCPLAGTPDVGLTAQDATTALGVPTRRRASSG

GIPVATSMWRDAQTVRTYGPAVAKALALRVAGKARSRLTGRHCRKFMQLT

DFDPFDPAIAADPYPHYRELLAGERVQYNPKRDVYILSRYADVREAARNH

DTLSSARGVTFSRGWLPFLPTSDPPAHTRMRKQLAPGMARGALETWRPMV

DQLARELVGGLLTQTPADVVSTVAAPMPMRAITSVLGVDGPDEAAFCRLS

NQAVRITDVALSASGLISLVQGFAGFRRLRALFTHRRDNGLLRECTVLGK

LATHAEQGRLSDDELFFFAVLLLVAGYESTAHMISTLFLTLADYPDQLTL

LAQQPDLIPSAIEEHLRFISPIQNICRTTRVDYSVGQAVIPAGSLVLLAW

GAANRDPRQYEDPDVFRADRNPVGHLAFGSGIHLCPGTQLARMEGQAILR

EIVANIDRIEVVEPPTWTTNANLRGLTRLRVAVTPRVAP

>CYP128A1 (640607052) Mycobacterium tuberculosis F11

MTATQSPPEPAPDRVRLAGCPLAGTPDVGLTAQDATTALGVPTRRRASSG

GIPVATSMWRDAQTVRTYGPAVAKALALRVAGKARSRLTGRHCRKFMQLT

DFDPFDPAIAADPYPHYRELLAGERVQYNPKRDVYILSRYADVREAARNH

DTLSSARGVTFSRGWLPFLPTSDPPAHTRMRKQLAPGMARGALETWRPMV

DQLARELVGGLLTQTPADVVSTVAAPMPMRAITSVLGVDGPDEAAFCRLS

NQAVRITDVALSASGLISLVQGFAGFRRLRALFTHRRDNGLLRECTVLGK

LATHAEQGRLSDDELFFFAVLLLVAGYESTAHMISTLFLTLADYPDQLTL

LAQQPDLIPSAIEEHLRFISPIQNICRTTRVDYSVGQAVIPAGSLVLLAW

GAANRDPRQYEDPDVFRADRNPVGHLAFGSGIHLCPGTQLARMEGQAILR

EIVANIDRIEVVEPPTWTTNANLRGLTRLRVAVTPRVAP

>CYP128A1 (2584718126) Mycobacterium tuberculosis XTB13-082

MTATQSPPEPAPDRVRLAGCPLAGTPDVGLTAQDATTALGVPTRRRAASG

GIPVATSMWRDAQTVRTYGPAVAKALALRVAGKARSRLTGRHCRKFMQLT

DFDPFDPAIAADPYPHYRELLAGERVQYNPKRDVYILSRYADVREAARNH

DTLSSARGVTFSRGWLPFLPTSDPPAHTRMRKQLAPGMARGALETWRPMV

DQLARELVGGLLTQTPADVVSTVAAPMPMRAITSVLGVDGPDEAAFCRLS

NQAVRITDVALSASGLISLVQGFAGFRRLRALFTHRRDNGLLRECTVLGK

LATHAEQGRLSDDELFFFAVLLLVAGYESTAHMISTLFLTLADYPDQLTL

LAQQPDLIPSAIEEHLRFISPIQNICRTTRVDYSVGQAVIPAGSLVLLAW

GAANRDPRQYEDPDVFRADRNPVGHLAFGSGIHLCPGTQLARMEGQAILR

EIVANIDRIEVVEPPTWTTNANLRGLTRLRVAVTPRVAP

>CYP128A1 (2600670632) Mycobacterium bovis BCG Sweden

MTATQSPPEPAPDRVRLAGCPLAGTPDVGLTAQDATTALGVPTRRRASSG

GIPVATSMWRDAQTVRTYGPAVAKALALRVAGKARSRLTGRHCRKFMQLT

DFDPFDPAIAADPYPHYRELLAGERVQYNPKRDVYILSRYADVREAARNH

DTLSSARGVTFSRGWLPFLPTSDPPAHTRMRKQLAPGMARGALETWRPMV

DQFARELVGGLLTQTPADVVSTVAAPMPMRAITSVLGVDGPDEAAFCRLS

NQAVRITDVALSASGLISLVQGFAGFRRLRALFTHRRDNGLLRECTVLGK

LATHAEQGRLSDDELFFFAVLLLVAGYESTAHMISTLFLTLADYPDQLTL

LAQQPDLIPSAIEEHLRFISPIQNICRTTRVDYSVGQAVIPAGSLVLLAW

GAANRDPRQYEDPDVFRADRNPVGHLAFGSGIHLCPGTQLARMEGQAILR

EIVANIDRIEVVEPPTWTTNANLRGLTRLRVAVTPRVAP

>CYP128A1 (2605077792) Mycobacterium tuberculosis BTB07-170

MTATQSPPEPAPDRVRLAGCPLAGTPDVGLTAQDATTALGVPTRRRAASG

GIPVATSMWRDAQTVRTYGPAVAKALALRVAGKARSRLTGRHCRKFMQLT

DFDPFDPAIAADPYPHYRELLAGERVQYNPKRDVYILSRYADVREAARNH

DTLSSARGVTFSRGWLPFLPTSDPPAHTRMRKQLAPGMARGALETWRPMV

DQLARELVGGLLTQTPADVVSTVAAPMPMRAITSVLGVDGPDEAAFCRLS

NQAVRITDVALSASGLISLVQGFAGFRRLRALFTHRRDNGLLRECTVLGK

LATHAEQGRLSDDELFFFAVLLLVAGYESTAHMISTLFLTLADYPDQLTL

LAQQPDLIPSAIEEHLRFISPIQNICRTTRVDYSVGQAVIPAGSLVLLAW

GAANRDPRQYEDPDVFRADRNPVGHLAFGSGIHLCPGTQLARMEGQAILR

EIVANIDRIEVVEPPTWTTNANLRGLTRLRVAVTPRVAP

>CYP128A1 (2605296398) Mycobacterium tuberculosis BTB10-092

MTATQSPPEPAPDRVRLAGCPLAGTPDVGLTAQDATTALGVPTRRRASSG

GIPVATSMWRDAQTVRTYGPAVAKALALRVAGKARSRLTGRHCRKFMQLT

DFDPFDPAIAADPYPHYRELLAGERVQYNPKRDVYILSRYADVREAARNH

DTLSSSRGVTFSRGWLPFLPTSDPPAHTRMRKQLAPGMARGALETWRPMV

DQLARELVGGLLTQTPADVVSTVAAPMPMRAITSVLGVDGPDEAAFCRLS

NQAVRITDVALSASGLISLVQGFAGFRRLRALFTHRRDNGLLRECTVLGK

LATHAEQGRLSDDELFFFAVLLLVAGYESTAHMISTLFLTLADYPDQLTL

LAQQPDLIPSAIEEHLRFISPIQNICRTTRVDYSVGQAVIPAGSLVLLAW

GAANRDPRQYEDPDVFRADRNPVGHLAFGSGIHLCPGTQLARMEGQAILR

EIVANIDRIEVVEPPTWTTNANLRGLTRLRVAVTPRVAP

>CYP128B4 (2661641867) Mycobacterium abscessus PAP054

MSRWRSDSAVRQIGAPVVAAVGMNIAAAVRVRRRGYAGWTGAVNTDYDPL

DPATAAQPFDAYRALHAGGRVHYNPKRATFILSRHEDIRAALRDTDAVTS

SQGVTRMKISAPILVLTDGDDHTRLRKQVQPGFTRGAMSDWQGMADQLAK

ELVADVVANPGCDVMERLAVPLPIRMIAHIIGIPPEDVQNFRSWSEDGVG

VINAGVSPAGLRQGLKGVRAIAALRRYFKDQLASGKLKGSDTVLGRLVDN

NEDGKLSDDELFFIAMLLLFAGNETTTNLIGGMFDTLAHAPDQFAMIRDD

PDLIPSAVEEQLRYSAPIQNLYRYTRTDYRVGEVTIPSGSRLLLAFGAAN

RDPEVFEDPDTYRADRNPRNHIAFGYGVHMCIGATLSRMEGQAVLRELTS

QASAIAAAGSATWSTNSSLRGTTYLPIRLTPAR

>CYP128A1 (2589560079) Mycobacterium tuberculosis TKK-01-0029

MTATQSPPEPAPDRVRLAGCPLAGTPDVGLTAQDATTALGVPTRRRASSG

GIPVATSMWRDAQTVRTYGPAVAKALALRVAGKARSRLTGRHCRKFMQLT

DFDPFDPAIAADPYPHYRELLAGERVQYNPKRDVYILSRYADVREAARNH

DTLSSARGVTFSRGWLPFLPTSDPPAHTRMRKQLAPGMARGALETWRPMV

DQLARELVGGLLTQTPADVVSTVAAPMPMRAITSVLGVDGPDEAAFCRLS

NQAVRITDVALSASGLISLVQGFAGFRRLRALFTHRRDNGLLRECTVLGK

LATHAEQGRLSDDELFFFAVLLLVAGYESTAHMISTLFLTLADYPDQLTL

LAQQPDLIPSAIEEHLRFISPIQNICRTTRVDYSVGQAVIPAGSLVLLAW

GAANRDPRQYEDPDVFRADRNPVGHLAFGSGIHLCPGTQLARMEGQAILR

EIVANIDRIEVVEPPTWTTNANLRGLTRLRVAVTPRVAP

>CYP128A1 (2603173261) Mycobacterium tuberculosis BTB12-211

MTATQSPPEPAPDRVRLAGCPLAGTPDVGLTAQDATTALGVPTRRRASSG

GIPVATSMWRDAQTVRTYGPAVAKALALRVAGKARSRLTGRHCRKFMQLT

DFDPFDPAIAADPYPHYRELLAGERVQYNPKRDVYILSRYADVREAARNH

DTLSSARGVTFSRGWLPFLPTSDPPAHTRMRKQLAPGMARGALETWRPMV

DQLARELVGGLLTQTPADVVSTVAAPMPMRAITSVLGVDGPDEAAFCRLS

NQAVRITDVALSASGLISLVQGFAGFRRLRALFTHRRDNGLLRECTVLGK

LATHAEQGRLSDDELFFFAVLLLVAGYESTAHMISTLFLTLADYPDQLTL

LAQQPDLIPSAIEEHLRFISPIQNICRTTRVDYSVGQAVIPAGSLVLLAW

GAANRDPRQYEDPDVFRADRNPVGHLAFGSGIHLCPGTQLARMEGQAILR

EIVANIDRIEVVEPPTWTTNANLRGLTRLRVAVTPRVAP

>CYP128A1 (2604283607) Mycobacterium tuberculosis TRUG0113

MTATQSPPEPAPDRVRLAGCPLAGTPDVGLTAQDATTALGVPTRRRASSG

GIPVATSMWRDAQTVRTYGPAVAKALALRVAGKARSRLTGRHCRKFMQLT

DFDPFDPAIAADPYPHYRELLAGERVQYNPKRDVYILSRYADVREAARNH

DTLSSARGVTFSRGWLPFLPTSDPPAHTRMRKQLAPGMARGALETWRPMV

DQLARELVGGLLTQTPADVVSTVAAPMPMRAITSVLGVDGPDEAAFCRLS

NQAVRITDVALSASGLISLVQGFAGFRRLRALFTHRRDNGLLRECTVLGK

LATHAEQGRLSDDELFFFAVLLLVAGYESTAHMISTLFLTLADYPDQLTL

LAQQPDLIPSAIEEHLRFISPIQNICRTTRVDYSVGQAVIPAGSLVLLAW

GAANRDPRQYEDPDVFRADRNPVGHLAFGSGIHLCPGTQLARMEGQAILR

EIVANIDRIEVVEPPTWTTNANLRGLTRLRVAVTPRVAP

>CYP128A1 (2606423795) Mycobacterium tuberculosis Mtb194

MTATQSPPEPAPDRVRLAGCPLAGTPDVGLTAQDATTALGVPTRRRASSG

GIPVATSMWRDAQTVRTYGPAVAKALALRVAGKARSRLTGRHCRKFMQLT

DFDPFDPAIAADPYPHYRELLAGERVQYNPKRDVYILSRYADVREAARNH

DTLSSARGVTFSRGWLPFLPTSDPPAHTRMRKQLAPGMARGALETWRPMV

DQLARELVGGLLTQTPADVVSTVAAPMPMRAITSVLGVDGPDEAAFCRLS

NQAVRITDVALSASGLISLVQGFAGFRRLRALFTHRRDNGLLRECTVLGK

LATHAEQGRLSDDELFFFAVLLLVAGYESTAHMISTLFLTLADYPDQLTL

LAQQPDLIPSAIEEHLRFISPIQNICRTTRVDYSVGQAVIPAGSLVLLAW

GAANRDPRQYEDPDVFRADRNPVGHLAFGSGIHLCPGTQLARMEGQAILR

EIVANIDRIEVVEPPTWTTNANLRGLTRLRVAVTPRVAP

>CYP128A1 (2662949377) Mycobacterium tuberculosis 400285

MTATQSPPEPAPDRVRLAGCPLAGTPDVGLTAQDATTALGVPTRRRASSG

GIPVATSMWRDAQTVRTYGPAVAKALALRVAGKARSRLTGRHCRKFMQLT

DFDPFDPAIAADPYPHYRELLAGERVQYNPKRDVYILSRYADVREAARNH

DTLSSARGVTFSRGWLPFLPTSDPPAHTRMRKQLAPGMARGALETWRPMV

DQLARELVGGLLTQTPADVVSTVAAPMPMRAITSVLGVDGPDEAAFCRLS

NQAVRITDVALSASGLISLVQGFAGFRRLRALFTHRRDNGLLRECTVLGK

LATHAEQGRLSDDELFFFAVLLLVAGYESTAHMISTLFLTLADYPDQLTL

LAQQPDLIPSAIEEHLRFISPIQNICRTTRVDYSVGQAVIPAGSLVLLAW

GAANRDPRQYEDPDVFRADRNPVGHLAFGSGIHLCPGTQLARMEGQAILR

EIVANIDRIEVVEPPTWTTNANLRGLTRLRVAVTPRVAP

>CYP128A1 (2697962192) Mycobacterium tuberculosis P00301553

MTATQSPPEPAPDRVRLAGCPLAGTPDVGLTAQDATTALGVPTRRRASSG

GIPVATSMWRDAQTVRTYGPAVAKALALRVAGKARSRLTGRHCRKFMQLT

DFDPFDPAIAADPYPHYRELLAGERVQYNPKRDVYILSRYADVREAARNH

DTLSSARGVTFSRGWLPFLPTSDPPAHTRMRKQLAPGMARGALETWRPMV

DQLARELVGGLLTQTPADVVSTVAAPMPMRAITSVLGVDGPDEAAFCRLS

NQAVRITDVALSASGLISLVQGFAGFRRLRALFTHRRDNGLLRECTVLGK

LATHAEQGRLSDDELFFFAVLLLVAGYESTAHMISTLFLTLADYPDQLTL

LAQQPDLIPSAIEEHLRFISPIQNICRTTRVDYSVGQAVIPAGSLVLLAW

GAANRDPRQYEDPDVFRADRNPVGHLAFGSGIHLCPGTQLARMEGQAILR

EIVANIDRIEVVEPPTWTTNANLRGLTRLRVAVTPRVAP

>CYP128A1 (2701676286) Mycobacterium tuberculosis 1200172

MTATQSPPEPAPDRVRLAGCPLAGTPDVGLTAQDATTALGVPTRRRASSG

GIPVATSMWRDAQTVRTYGPAVAKALALRVAGKARSRLTGRHCRKFMQLT

DFDPFDPAIAADPYPHYRELLAGERVQYNPKRDVYILSRYADVREAARNH

DTLSSARGVTFSRGWLPFLPTSDPPAHTRMRKQLAPGMARGALETWRPMV

DQLARELVGGLLTQTPADVVSTVAAPMPMRAITSVLGVDGPDEAAFCRLS

NQAVRITDVALSASGLISLVQGFAGFRRLRALFTHRRDNGLLRECTVLGK

LATHAEQGRLSDDELFFFAVLLLVAGYESTAHMISTLFLTLADYPDQLTL

LAQQPDLIPSAIEEHLRFISPIQNICRTTRVDYSVGQAVIPAGSLVLLAW

GAANRDPRQYEDPDVFRADRNPVGHLAFGSGIHLCPGTQLARMEGQAILR

EIVANIDRIEVVEPPTWTTNANLRGLTRLRVAVTPRVAP

>CYP128A1 (2683834420) Mycobacterium tuberculosis EE0506784

MTATQSPPEPAPDRVRLAGCPLAGTPDVGLTAQDATTALGVPTRRRAASG

GIPVATSMWRDAQTVRTYGPAVAKALALRVAGKARSRLTGRHCRKFMQLT

DFDPFDPAIAADPYPHYRELLAGERVQYNPKRDVYILSRYADVREAARNH

DTLSSARGVTFSRGWLPFLPTSDPPAHTRMRKQLAPGMARGALETWRPMV

DQLARELVGGLLTQTPADVVSTVAAPMPMRAITSVLGVDGPDEAAFCRLS

NQAVRITDVALSASGLISLVQGFAGFRRLRALFTHRRDNGLLRECTVLGK

LATHAEQGRLSDDELFFFAVLLLVAGYESTAHMISTLFLTLADYPDQLTL

LAQQPDLIPSAIEEHLRFISPIQNICRTTRVDYSVGQAVIPAGSLVLLAW

GAANRDPRQYEDPDVFRADRNPVGHLAFGSGIHLCPGTQLARMEGQAILR

EIVANIDRIEVVEPPTWTTNANLRGLTRLRVAVTPRVAP

>CYP128B4 (2798221432) Mycobacteroides abscessus abscessus 956

MSRWRSDSAVRQIGAPVVAAVGMNIAAAVRVRRRGYAGWTGAVNTDYDPL

DPATAAQPFDAYRALHAGGRVHYNPKRATFILSRHEDIRAALRDTDAVTS

SQGVTRMKISAPILVLTDGDDHTRLRKQVQPGFTRGAMSDWQGMADQLAK

ELVADVVANPGCDVMERLAVPLPIRMIAHIIGIPPEDVQNFRSWSEDGVG

VINAGVSPAGLRQGLKGVRAIAALRRYFKDQLASGKLKGSDTVLGRLVDN

NEDGKLSDDELFFIAMLLLFAGNETTTNLIGGMFDTLAHAPDQFAMIRDD

PDLIPSAVEEQLRYSAPIQNLYRYTRTDYRVGEVTIPSGSRLLLAFGAAN

RDPEVFEDPDTYRADRNPRNHIAFGYGVHMCIGATLSRMEGQAVLRELTS

QASAIAAAGSATWSTNSSLRGTTYLPIRLTPAR

>CYP128B1 (2810781720) Mycobacterium asiaticum 1276495.2

MAAKQAFQGTAEGARLLGHAARMNLAAAVRTRRRGCAGWTGAINTDYDPQ

DPLTAAQPFDAYRALHRGGRVHYNPRRATFIISRLDDVRAALRDTDQVTS

SQGVTRLRMSAPLAVLTDGEEHARLRRQVQPGFSKGAMKAWQGMIEELAE

ELVGDVLANPGCDVVRQLAIPMPIRLIAKILGIPNDDVGDFRRWSERGVG

VMDVTPTLPGLVDAARSVTAMAALQRYFVKQFTAGGLKGSDTVLGRLLAH

NTDGSLTDRQLLLIAIHLLIAGNETTTNLLGGMFDTLANIHDQYEMIRTQ

PDLIPLAVEEQLRITTPIQNLYRYTRADYQVGDVTIPNGSRVLLSFGAAN

RDPTAFEEPDQYRADRNPRTHVAFGYGAHMCLGAPLARMEAQAVLRQLIT

RVSRITPAGPTTWSTHSSLRGPTRLPIRLTAA

>CYP128A1 (2575253008) Mycobacterium tuberculosis M1481

MTATQSPPEPAPDRVRLAGCPLAGTPDVGLTAQDATTALGVPTRRRASSG

GIPVATSMWRDAQTVRTYGPAVAKALALRVAGKARSRLTGRHCRKFMQLT

DFDPFDPAIAADPYPHYRELLAGERVQYNPKRDVYILSRYADVREAARNH

DTLSSARGVTFSRGWLPFLPTSDPPAHTRMRKQLAPGMARGALETWRPMV

DQLARELVGGLLTQTPADVVSTVAAPMPMRAITSVLGVDGPDEAAFCRLS

NQAVRITDVALSASGLISLVQGFAGFRRLRALFTHRRDNGLLRECTVLGK

LATHAEQGRLSDDELFFFAVLLLVAGYESTAHMISTLFLTLADYPDQLTL

LAQQPDLIPSAIEEHLRFISPIQNICRTTRVDYSVGQAVIPAGSLVLLAW

GAANRDPRQYEDPDVFRADRNPVGHLAFGSGIHLCPGTQLARMEGQAILR

EIVANIDRIEVVEPPTWTTNANLRGLTRLRVAVTPRVAP

>CYP128A1 (2578074393) Mycobacterium tuberculosis TB_RSA74

MTATQSPPEPAPDRVRLAGCPLAGTPDVGLTAQDATTALGVPTRRRASSG

GIPVATSMWRDAQTVRTYGPAVAKALALRVAGKARSRLTGRHCRKFMQLT

DFDPFDPAIAADPYPHYRELLAGERVQYNPKRDVYILSRYADVREAARNH

DTLSSARGVTFSRGWLPFLPTSDPPAHTRMRKQLAPGMARGALETWRPMV

DQLARELVGGLLTQTPADVVSTVAAPMPMRAITSVLGVDGPDEAAFCRLS

NQAVRITDVALSASGLISLVQGFAGFRRLRALFTHRRDNGLLRECTVLGK

LATHAEQGRLSDDELFFFAVLLLVAGYESTAHMISTLFLTLADYPDQLTL

LAQQPDLIPSAIEEHLRFISPIQNICRTTRVDYSVGQAVIPAGSLVLLAW

GAANRDPRQYEDPDVFRADRNPVGHLAFGSGIHLCPGTQLARMEGQAILR

EIVANIDRIEVVEPPTWTTNANLRGLTRLRVAVTPRVAP

>CYP128A1 (2584730923) Mycobacterium tuberculosis M1703

MTATQSPPEPAPDRVRLAGCPLAGTPDVGLTAQDATTALGVPTRRRASSG

GIPVATSMWRDAQTVRTYGPAVAKALALRVAGKARSRLTGRHCRKFMQLT

DFDPFDPAIAADPYPHYRELLAGERVQYNPKRDVYILSRYADVREAARNH

DTLSSARGVTFSRGWLPFLPTSDPPAHTRMRKQLAPGMARGALETWRPMV

DQLARELVGGLLTQTPADVVSTVAAPMPMRAITSVLGVDGPDEAAFCRLS

NQAVRITDVALSASGLISLVQGFAGFRRLRALFTHRRDNGLLRECTVLGK

LATHAEQGRLSDDELFFFAVLLLVAGYESTAHMISTLFLTLADYPDQLTL

LAQQPDLIPSAIEEHLRFISPIQNICRTTRVDYSVGQAVIPAGSLVLLAW

GAANRDPRQYEDPDVFRADRNPVGHLAFGSGIHLCPGTQLARMEGQAILR

EIVANIDRIEVVEPPTWTTNANLRGLTRLRVAVTPRVAP

>CYP128A1 (2584792119) Mycobacterium tuberculosis 1010SM

MTATQSPPEPAPDRVRLAGCPLAGTPDVGLTAQDATTALGVPTRRRASSG

GIPVATSMWRDAQTVRTYGPAVAKALALRVAGKARSRLTGRHCRKFMQLT

DFDPFDPAIAADPYPHYRELLAGERVQYNPKRDVYILSRYADVREAARNH

DTLSSARGVTFSRGWLPFLPTSDPPAHTRMRKQLAPGMARGALETWRPMV

DQLARELVGGLLTQTPADVVSTVAAPMPMRAITSVLGVDGPDEAAFCRLS

NQAVRITDVALSASGLISLVQGFAGFRRLRALFTHRRDNGLLRECTVLGK

LATHAEQGRLSDDELFFFAVLLLVAGYESTAHMISTLFLTLADYPDQLTL

LAQQPDLIPSAIEEHLRFISPIQNICRTTRVDYSVGQAVIPAGSLVLLAW

GAANRDPRQYEDPDVFRADRNPVGHLAFGSGIHLCPGPSWRAWRVRRSCA

RSSPISTE

>CYP128A1 (2589077242) Mycobacterium tuberculosis TBR43

MTATQSPPEPAPDRVRLAGCPLAGTPDVGLTAQDATTALGVPTRRRASSG

GIPVATSMWRDAQTVRTYGPAVAKALALRVAGKARSRLTGRHCRKFMQLT

DFDPFDPAIAADPYPHYRELLAGERVQYNPKRDVYILSRYADVREAARNH

DTLSSARGVTFSRGWLPFLPTSDPPAHTRMRKQLAPGMARGALETWRPMV

DQLARELVGGLLTQTPADVVSTVAAPMPMRAITSVLGVDGPDEAAFCRLS

NQAVRITDVALSASGLISLVQGFAGFRRLRALFTHRRDNGLLRECTVLGK

LATHAEQGRLSDDELFFFAVLLLVAGYESTAHMISTLFLTLADYPDQLTL

LAQQPDLIPSAIEEHLRFISPIQNICRTTRVDYSVGQAVIPAGSLVLLAW

GAANRDPRQYEDPDVFRADRNPVGHLAFGSGIHLCPGTQLARMEGQAILR

EIVANIDRIEVVEPPTWTTNANLRGLTRLRVAVTPRVAP

>CYP128A1 (2592546730) Mycobacterium tuberculosis TKK_04_0046

MTATQSPPEPAPDRVRLAGCPLAGTPDVGLTAQDATTALGVPTRRRASSG

GIPVATSMWRDAQTVRTYGPAVAKALALRVAGKARSRLTGRHCRKFMQLT

DFDPFDPAIAADPYPHYRELLAGERVQYNPKRDVYILSRYADVREAARNH

DTLSSARGVTFSRGWLPFLPTSDPPAHTRMRKQLAPGMARGALETWRPMV

DQLARELVGGLLTQTPADVVSTVAAPMPMRAITSVLGVDGPDEAAFCRLS

NQAVRITDVALSASGLISLVQGFAGFRRLRALFTHRRDNGLLRECTVLGK

LATHAEQGRLSDDELFFFAVLLLVAGYESTAHMISTLFLTLADYPDQLTL

LAQQPDLIPSAIEEHLRFISPIQNICRTTRVDYSVGQAVIPAGSLVLLAW

GAANRDPRQYEDPDVFRADRNPVGHLAFGSGIHLCPGTQLARMEGQAILR

EIVANIDRIEVVEPPTWTTNANLRGLTRLRVAVTPRVAP

>CYP128A1 (2603129319) Mycobacterium tuberculosis 43-16836

MTATQSPPEPAPDRVRLAGCPLAGTPDVGLTAQDATTALGVPTRRRASSG

GIPVATSMWRDAQTVRTYGPAVAKALALRVAGKARSRLTGRHCRKFMQLT

DFDPFDPAIAADPYPHYRELLAGERVQYNPKRDVYILSRYADVREAARNH

DTLSSARGVTFSRGWLPFLPTSDPPAHTRMRKQLAPGMARGALETWRPMV

DQLARELVGGLLTQTPADVVSTVAAPMPMRAITSVLGVDGPDEAAFCRLS

NQAVRITDVALSASGLISLVQGFAGFRRLRALFTHRRDNGLLRECTVLGK

LATHAEQGRLSDDELFFFAVLLLVAGYESTAHMISTLFLTLADYPDQLTL

LAQQPDLIPSAIEEHLRFISPIQNICRTTRVDYSVGQAVIPAGSLVLLAW

GAANRDPRQYEDPDVFRADRNPVGHLAFGSGIHLCPGTQLARMEGQAILR

EIVANIDRIEVVEPPTWTTNANLRGLTRLRVAVTPRVAP

>CYP128A1 (2622382735) Mycobacterium tuberculosis M1215

MTATQSPPEPAPDRVRLAGCPLAGTPDVGLTAQDATTALGVPTRRRASSG

GIPVATSMWRDAQTVRTYGPAVAKALALRVAGKARSRLTGRHCRKFMQLT

DFDPFDPAIAADPYPHYRELLAGERVQYNPKRDVYILSRYADVREAARNH

DTLSSARGVTFSRGWLPFLPTSDPPAHTRMRKQLAPGMARGALETWRPMV

DQLARELVGGLLTQTPADVVSTVAAPMPMRAITSVLGVDGPDEAAFCRLS

NQAVRITDVALSASGLISLVQGFAGFRRLRALFTHRRDNGLLRECTVLGK

LATHAEQGRLSDDELFFFAVLLLVAGYESTAHMISTLFLTLADYPDQLTL

LAQQPDLIPSAIEEHLRFISPIQNICRTTRVDYSVGQAVIPAGSLVLLAW

GAANRDPRQYEDPDVFRADRNPVGHLAFGSGIHLCPGTQLARMEGQAILR

EIVANIDRIEVVEPPTWTTNANLRGLTRLRVAVTPRVAP

>CYP128A1 (2622450282) Mycobacterium tuberculosis M1304

MTATQSPPEPAPDRVRLAGCPLAGTPDVGLTAQDATTALGVPTRRRASSG

GIPVATSMWRDAQTVRTYGPAVAKALALRVAGKARSRLTGRHCRKFMQLT

DFDPFDPAIAADPYPHYRELLAGERVQYNPKRDVYILSRYADVREAARNH

DTLSSARGVTFSRGWLPFLPTSDPPAHTRMRKQLAPGMARGALETWRPMV

DQLARELVGGLLTQTPADVVSTVAAPMPMRAITSVLGVDGPDEAAFCRLS

NQAVRITDVALSASGLISLVQGFAGFRRLRALFTHRRDNGLLRECTVLGK
[truncated: 1,092,462 more chars]
