# Supplementary material for: Comparative Analysis, Structural Insights, and Substrate/Drug Interaction of CYP128A1 in Mycobacterium tuberculosis
Source: Int J Mol Sci. 2020 Jul 8;21(14):4816. doi: 10.3390/ijms21144816 (PMC7404182; doi:10.3390/ijms21144816)

Article

Comparative analysis, structural insights, and substrate/drug interaction of CYP128A1 in *Mycobacterium tuberculosis*

Nokwanda Samantha Ngcobo ^1^, Zinhle Edith Chiliza ^1^, Wanping Chen ^2^, Jae-Hyuk Yu ^3,4^, David R Nelson ^5^, Jack A Tuszynski ^6,7^, Jordane Preto ^8^*, Khajamohiddin Syed ^1,^*

^1^ Department of Biochemistry and Microbiology, Faculty of Science and Agriculture, University of Zululand, KwaDlangezwa 3886, South Africa; [mskwandosamn@gmail.com](mailto:mskwandosamn@gmail.com) (N.S.N.); zinhlechiliza01@gmail.com (Z.C.E); khajamohiddinsyed@gmail.com (K.S.)

^2^ Department of Molecular Microbiology and Genetics, University of Göttingen, Göttingen 37077, Germany; chenwanping1@foxmail.com (W.C.)

^3^ Department of Bacteriology, University of Wisconsin-Madison, 3155 MSB, 1550 Linden Drive, Madison, WI 53706, USA; [jyu1@wisc.edu](mailto:jyu1@wisc.edu) (J-H.Y.)

^4^ Department of Systems Biotechnology, Konkuk University, Seoul 05029, Korea (J-H. Y.)

^5^ Department of Microbiology, Immunology and Biochemistry, University of Tennessee Health Science Center, Memphis, TN, 38163; [drnelson1@gmail.com](mailto:drnelson1@gmail.com) (D.R.N.)

^6^ Department of Physics and Department of Oncology, University of Alberta, Edmonton, AB T6G 2E1, Canada; [jack.tuszynski@gmail.com](mailto:jack.tuszynski@gmail.com) (J.A.T.)

^7^ Department of Mechanical and Aerospace Engineering, Politecnico di Torino, Corso Duca degli Abruzzi, 24, 10129 Torino TO, Italy

^8^ Université Claude Bernard Lyon 1, INSERM 1052, CNRS 5286, Centre Léon Bérard, Centre de Recherche en Cancérologie de Lyon, France; [jordane.preto@gmail.com](mailto:jordane.preto@gmail.com) (J.P.)

***** Correspondence: jordane.preto@gmail.com (J.P.) and khajamohiddinsyed@gmail.com (K.S.)

Received: date; Accepted: date; Published: date

Table S1. Comparative amino acid conservation acid analysis of CYP128 P450 family with top 10 ranked families. The conservation index score (5-9) is obtained as described elsewhere [32] using PROMALS3D where the number 9 indicates invariantly conserved amino acids in P450 members. CYP128 family is indicated in bold.

| P450 family | Number of member P450s | Kingdom | PROMALS3D conservation Index | | | | | Rank (highest to lowest conservation) |
| --- | --- | --- | --- | --- | --- | --- | --- | --- |
|  |  |  | 5 | 6 | 7 | 8 | 9 |  |
| CYP141 | 29 | Bacteria | 0 | 0 | 0 | 0 | 389 | 1 |
| CYP51 | 50 | Bacteria | 11 | 102 | 0 | 0 | 264 | 2 |
| CYP137 | 38 | Bacteria | 145 | 0 | 0 | 0 | 251 | 3 |
| CYP121 | 34 | Bacteria | 0 | 0 | 0 | 0 | 233 | 4 |
| **CYP128** | **2191** | **Bacteria** | **118** | **25** | **0** | **0** | **217** | **5** |
| CYP132 | 39 | Bacteria | 175 | 0 | 0 | 0 | 217 | 5 |
| CYP5619 | 23 | Stramenopila | 118 | 38 | 170 | 0 | 199 | 6 |
| CYP124 | 71 | Bacteria | 52 | 35 | 59 | 0 | 170 | 7 |
| CYP139 | 894 | Bacteria | 0 | 127 | 0 | 0 | 165 | 8 |
| CYP188 | 67 | Bacteria | 62 | 0 | 100 | 0 | 141 | 9 |
| CYP123 | 74 | Bacteria | 62 | 0 | 82 | 0 | 137 | 10 |
| CYP108 | 67 | Bacteria | 52 | 12 | 92 | 0 | 134 | 11 |
| CYP126 | 78 | Bacteria | 65 | 16 | 98 | 0 | 132 | 12 |
| CYP21 | 84 | Animal | 84 | 30 | 104 | 0 | 126 | 13 |
| CYP139 | 54 | Bacteria | 126 | 0 | 0 | 0 | 116 | 14 |
| CYP291 | 23 | Bacteria | 0 | 111 | 0 | 0 | 115 | 15 |
| CYP142 | 90 | Bacteria | 60 | 6 | 83 | 0 | 112 | 16 |
| CYP501 | 106 | Fungi | 44 | 60 | 113 | 0 | 106 | 17 |
| CYP164 | 50 | Bacteria | 49 | 9 | 94 | 0 | 99 | 19 |
| CYP130 | 98 | Bacteria | 31 | 31 | 91 | 0 | 93 | 20 |
| CYP73 | 155 | Plant | 52 | 69 | 57 | 71 | 91 | 21 |
| CYP5 | 71 | Animal | 37 | 48 | 117 | 0 | 89 | 22 |
| CYP116 | 93 | Bacteria | 98 | 92 | 39 | 55 | 89 | 22 |
| CYP195 | 52 | Bacteria | 35 | 38 | 54 | 21 | 89 | 22 |
| CYP24 | 65 | Animal | 87 | 11 | 96 | 0 | 84 | 23 |
| CYP84 | 62 | Plant | 68 | 64 | 48 | 44 | 80 | 24 |
| CYP98 | 77 | Plant | 68 | 83 | 44 | 48 | 76 | 25 |
| CYP190 | 76 | Bacteria | 34 | 19 | 87 | 0 | 75 | 26 |
| CYP185 | 42 | Bacteria | 65 | 20 | 72 | 0 | 68 | 27 |
| CYP61 | 70 | Fungi | 54 | 63 | 38 | 25 | 68 | 27 |
| CYP191 | 36 | Bacteria | 92 | 29 | 92 | 0 | 65 | 28 |
| CYP202 | 84 | Bacteria | 34 | 34 | 27 | 23 | 65 | 28 |
| CYP39 | 51 | Animal | 51 | 47 | 79 | 0 | 64 | 29 |
| CYP78 | 112 | Plant | 40 | 51 | 36 | 40 | 61 | 30 |
| CYP106 | 93 | Bacteria | 38 | 27 | 64 | 9 | 60 | 31 |
| CYP28 | 51 | Animal | 84 | 59 | 44 | 28 | 55 | 32 |
| CYP97 | 100 | Plant | 37 | 49 | 29 | 25 | 55 | 32 |
| CYP17 | 99 | Animal | 56 | 68 | 44 | 29 | 54 | 33 |
| CYP46 | 53 | Animal | 53 | 42 | 99 | 0 | 54 | 33 |
| CYP279 | 71 | Bacteria | 38 | 49 | 26 | 37 | 54 | 33 |
| CYP19 | 176 | Animal | 53 | 57 | 44 | 60 | 53 | 34 |
| CYP187 | 103 | Bacteria | 35 | 33 | 42 | 13 | 52 | 35 |
| CYP135 | 124 | Bacteria | 48 | 46 | 22 | 23 | 51 | 36 |
| CYP140 | 113 | Bacteria | 38 | 32 | 21 | 26 | 50 | 37 |
| CYP7 | 89 | Animal | 69 | 43 | 34 | 26 | 50 | 37 |
| CYP144 | 107 | Bacteria | 45 | 33 | 34 | 35 | 47 | 38 |
| CYP26 | 131 | Animal | 69 | 63 | 30 | 46 | 47 | 38 |
| CYP143 | 103 | Bacteria | 43 | 43 | 28 | 30 | 45 | 39 |
| CYP147 | 52 | Bacteria | 45 | 29 | 35 | 32 | 45 | 39 |
| CYP138 | 114 | Bacteria | 42 | 44 | 24 | 32 | 44 | 40 |
| CYP51 | 82 | Fungi | 64 | 42 | 39 | 33 | 43 | 41 |
| CYP705 | 50 | Plant | 66 | 49 | 41 | 28 | 43 | 41 |
| CYP33 | 67 | Animal | 61 | 49 | 34 | 40 | 42 | 42 |
| CYP706 | 51 | Plant | 53 | 49 | 34 | 32 | 42 | 42 |
| CYP86 | 139 | Plant | 64 | 39 | 34 | 29 | 40 | 43 |
| CYP707 | 100 | Plant | 59 | 54 | 47 | 57 | 40 | 43 |
| CYP714 | 58 | Plant | 58 | 43 | 25 | 26 | 40 | 43 |
| CYP136 | 171 | Bacteria | 48 | 34 | 25 | 28 | 39 | 44 |
| CYP268 | 73 | Bacteria | 43 | 43 | 27 | 33 | 37 | 45 |
| CYP125 | 103 | Bacteria | 42 | 39 | 29 | 17 | 37 | 45 |
| CYP27 | 116 | Animal | 66 | 46 | 25 | 25 | 37 | 45 |
| CYP150 | 164 | Bacteria | 59 | 45 | 20 | 35 | 36 | 46 |
| CYP8 | 91 | Animal | 65 | 34 | 36 | 37 | 36 | 46 |
| CYP96 | 61 | Plant | 57 | 40 | 29 | 30 | 35 | 47 |
| CYP11 | 171 | Animal | 46 | 47 | 27 | 29 | 33 | 48 |
| CYP153 | 164 | Bacteria | 41 | 34 | 37 | 28 | 33 | 48 |
| CYP53 | 102 | Fungi | 64 | 45 | 48 | 33 | 31 | 49 |
| CYP79 | 102 | Plant | 67 | 55 | 30 | 31 | 31 | 49 |
| CYP90 | 117 | Plant | 63 | 44 | 44 | 30 | 30 | 50 |
| CYP152 | 90 | Bacteria | 46 | 38 | 19 | 25 | 30 | 50 |
| CYP55 | 60 | Fungi | 34 | 56 | 51 | 21 | 29 | 51 |
| CYP709 | 113 | Plant | 50 | 42 | 18 | 26 | 29 | 51 |
| CYP189 | 188 | Bacteria | 54 | 37 | 17 | 27 | 28 | 52 |
| CYP704 | 109 | Plant | 45 | 43 | 33 | 43 | 27 | 53 |
| CYP5152 | 66 | Fungi | 46 | 28 | 25 | 15 | 26 | 54 |
| CYP12 | 119 | Animal | 59 | 37 | 23 | 20 | 26 | 54 |
| CYP505 | 165 | Fungi | 95 | 76 | 48 | 31 | 25 | 55 |
| CYP93 | 151 | Plant | 68 | 42 | 26 | 20 | 24 | 56 |
| CYP58 | 106 | Fungi | 52 | 43 | 29 | 21 | 23 | 57 |
| CYP75 | 251 | Plant | 74 | 48 | 31 | 29 | 23 | 57 |
| CYP584 | 96 | Fungi | 56 | 35 | 30 | 21 | 23 | 57 |
| CYP65 | 203 | Fungi | 38 | 25 | 13 | 11 | 22 | 58 |
| CYP72 | 208 | Plant | 57 | 27 | 28 | 27 | 20 | 59 |
| CYP89 | 134 | Plant | 48 | 38 | 28 | 25 | 20 | 59 |
| CYP52 | 161 | Fungi | 51 | 26 | 24 | 19 | 17 | 60 |
| CYP110 | 113 | Bacteria | 50 | 27 | 17 | 18 | 17 | 60 |
| CYP325 | 53 | Animal | 50 | 31 | 19 | 10 | 17 | 60 |
| CYP5035 | 129 | Fungi | 37 | 30 | 17 | 9 | 15 | 61 |
| CYP620 | 178 | Fungi | 33 | 21 | 12 | 17 | 15 | 61 |
| CYP102 | 333 | Bacteria | 45 | 43 | 16 | 19 | 14 | 62 |
| CYP63 | 133 | Fungi | 51 | 39 | 23 | 13 | 12 | 63 |
| CYP5139 | 181 | Fungi | 35 | 12 | 3 | 9 | 12 | 63 |
| CYP9 | 312 | Animal | 45 | 24 | 9 | 10 | 12 | 63 |
| CYP92 | 167 | Plant | 51 | 32 | 33 | 17 | 12 | 63 |
| CYP716 | 103 | Plant | 53 | 30 | 17 | 23 | 12 | 63 |
| CYP5136 | 68 | Fungi | 44 | 42 | 20 | 15 | 11 | 64 |
| CYP74 | 159 | Plant/Animal | 40 | 29 | 23 | 19 | 11 | 64 |
| CYP105 | 329 | Bacteria | 28 | 14 | 19 | 6 | 11 | 64 |
| CYP157 | 115 | Bacteria | 44 | 26 | 26 | 18 | 11 | 64 |
| CYP5150 | 336 | Fungi | 48 | 26 | 8 | 7 | 10 | 65 |
| CYP51 | 409 | Bacteria/fungi /animal/plant | 36 | 19 | 11 | 13 | 10 | 65 |
| CYP94 | 170 | Plant | 39 | 44 | 34 | 26 | 10 | 65 |
| CYP1 | 289 | Animal | 67 | 28 | 26 | 24 | 9 | 66 |
| CYP3 | 248 | Animal | 59 | 44 | 27 | 20 | 9 | 66 |
| CYP5141 | 86 | Fungi | 40 | 21 | 13 | 11 | 8 | 67 |
| CYP512 | 247 | Fungi | 25 | 12 | 8 | 5 | 8 | 67 |
| CYP82 | 174 | Plant | 68 | 31 | 20 | 21 | 8 | 67 |
| CYP87 | 78 | Plant | 58 | 29 | 23 | 20 | 8 | 67 |
| CYP107 | 217 | Bacteria | 40 | 25 | 8 | 12 | 8 | 67 |
| CYP6 | 921 | Animal | 21 | 9 | 7 | 8 | 6 | 68 |
| CYP5037 | 261 | Fungi | 19 | 6 | 7 | 5 | 5 | 69 |
| CYP5144 | 514 | Fungi | 10 | 4 | 5 | 3 | 5 | 69 |
| CYP76 | 206 | Plant | 56 | 31 | 21 | 10 | 5 | 69 |
| CYP81 | 235 | Plant | 40 | 30 | 29 | 14 | 5 | 69 |
| CYP4 | 1076 | Animal | 27 | 11 | 5 | 6 | 4 | 70 |
| CYP2 | 857 | Animal | 48 | 18 | 10 | 9 | 3 | 71 |
| CYP71 | 780 | Plant | 22 | 7 | 6 | 5 | 3 | 71 |

Figure S1. 3VRM/CYP128A1 alignement.

3VRM.A ALTTTGTEQHDLFSGTFWQNPHPAYAALRAEDPVRKLALPDGPVWLLTRYADVREAFVDP

CYP128A1 CRKFMQLTDFDPFDPAIAADPYPHYRELLAGERVQ..YNPKRDVYILSRYADVREAARN.

3VRM.A RLSKDWRHTLPEDQRADMPATPTPMMILMDPPDHTRLRKLVGRSFAVRRMNELEPRITEI

CYP128A1 ......HDTLSSARGVTFSRGWLPFLPTSDPPAHTRMRKQLAPGMARGALETWRPMVDQL

3VRM.A ADGLLAGLPTDGPVDLMREYAFQIPVQVICELLGVPAEDRDDFSAWSSVLVDDSP.ADDK

CYP128A1 ARELVGGLLTQTPADVVSTVAAPMPMRAITSVLGVDGPDEAAFCRLSNQAVRITDVALSA

3VRM.A NAAMGKLHGY.....LSDLLERKRTEP..DDALLSSLLAVSDEDGDRLSQEELVAMAMLL

CYP128A1 SGLISLVQGFAGFRRLRALFTHRRDNGLLRECTVLGKLATHAEQG.RLSDDELFFFAVLL

3VRM.A LIAGHETTVNLIGNGVLALLTHPDQRKLLAEDPSLISSAVEEFLRFDSPVSQAPIRFTAE

CYP128A1 LVAGYESTAHMISTLFLTLADYPDQLTLLAQQPDLIPSAIEEHLRFISPI.QNICRTTRV

3VRM.A DVTYSGVTIPAGEMVMLGLAAANRDADWMPEPDRLDITRDASGGVFFGHGIHFCLGAQLA

CYP128A1 DYSVGQAVIPAGSLVLLAWGAANRDPRQYEDPDVFRADRNPVGHLAFGSGIHLCPGTQLA

3VRM.A RLEGRVAIGRLFADRPELALAVGLDELVYRESTLVRGLSRMPVTMGPRSAX

CYP128A1 RMEGQAILREIVANIDRIEV...VEPPTWTTNANLRGLTRLRVAVTPRVAP

Figure S2. 2D structures of substrate (menaquinone 9) and azole compounds used in the study.

| 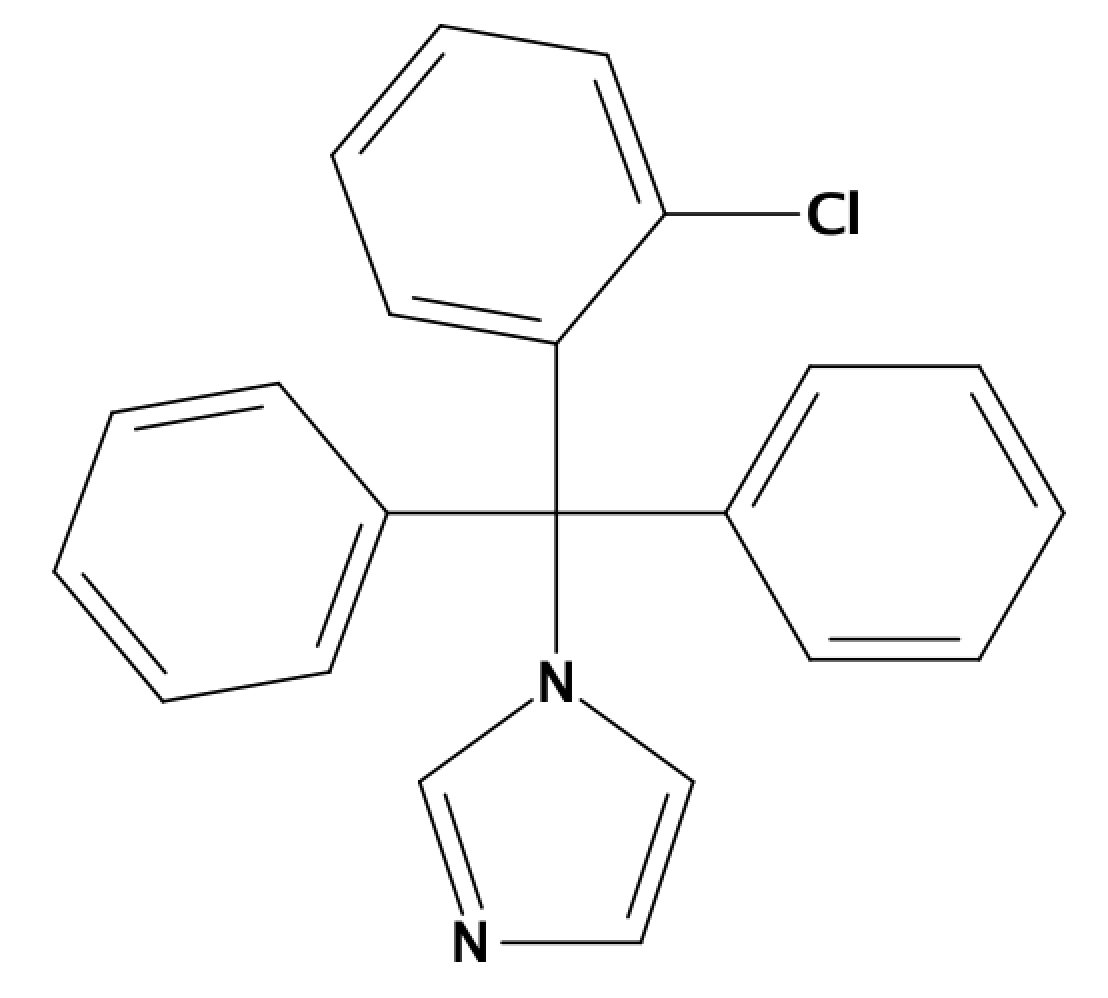  Clotrimazole | 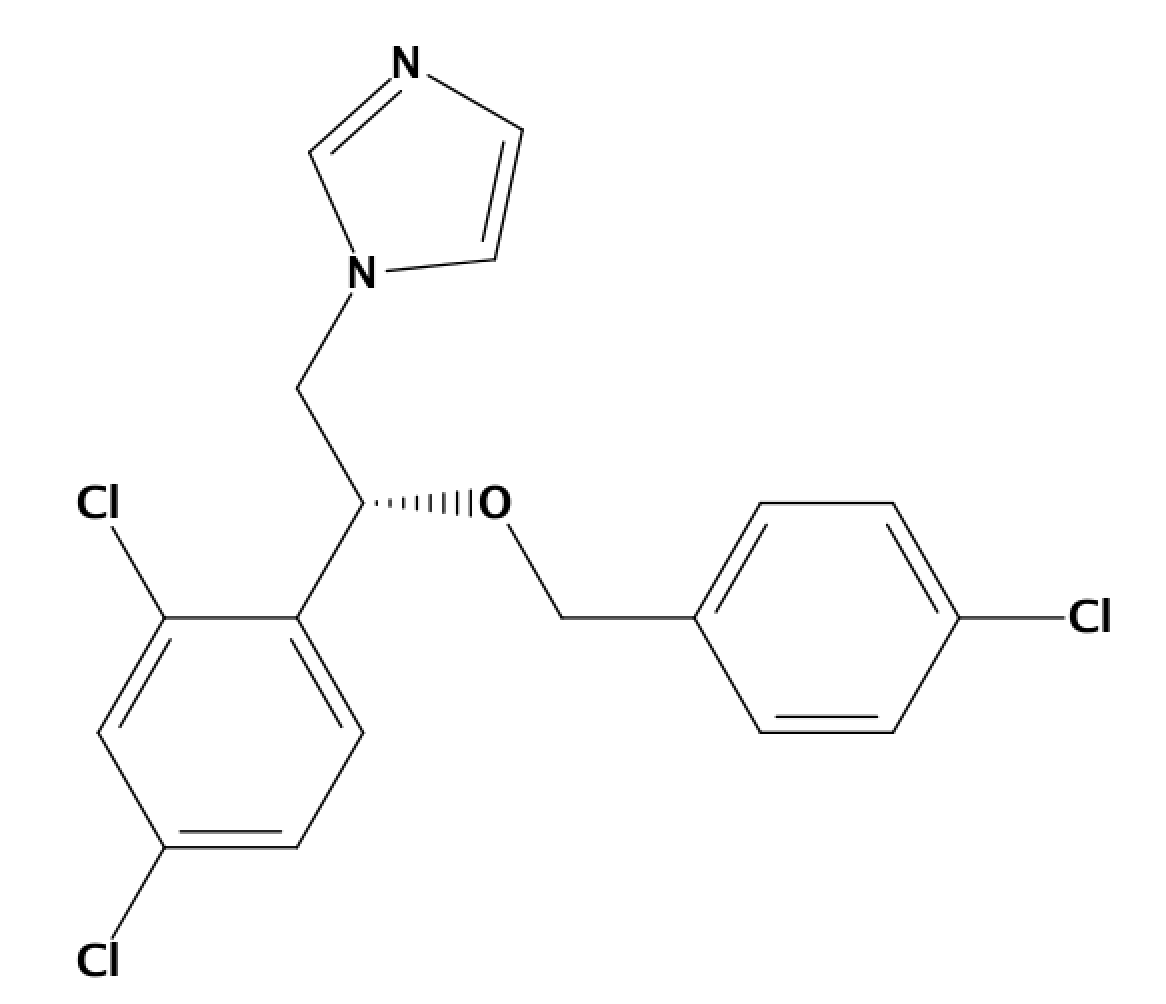  Econazole | | 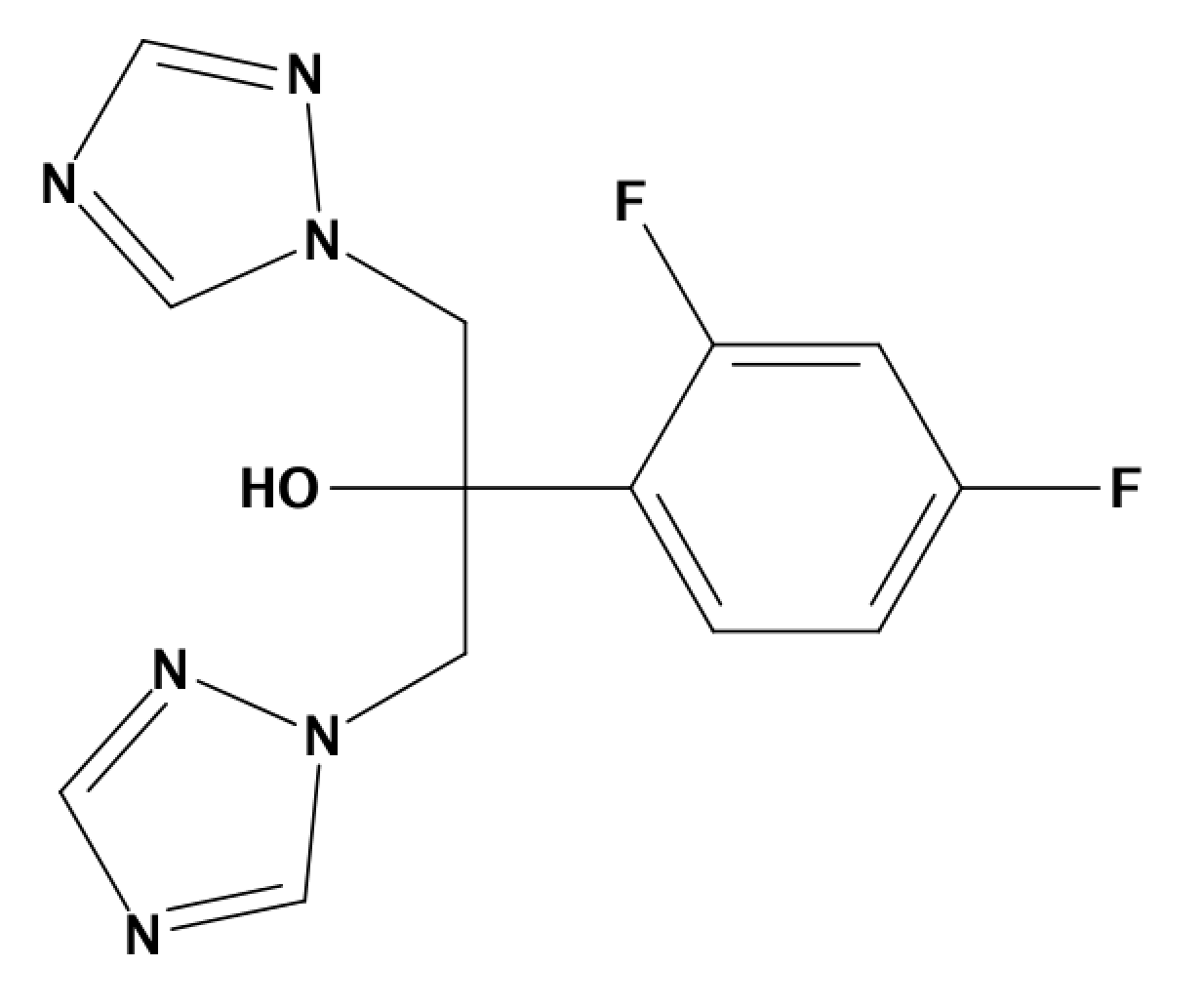  Fluconazole |
| --- | --- | --- | --- |
| 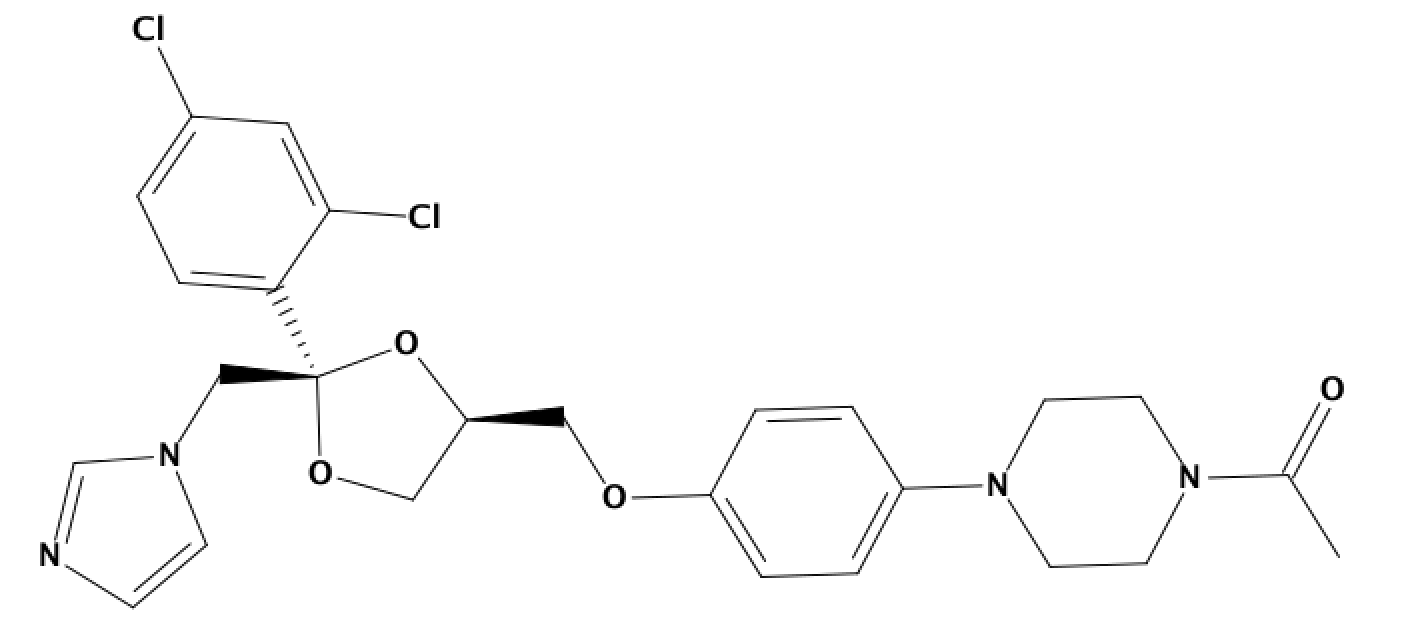  Ketoconazole | | 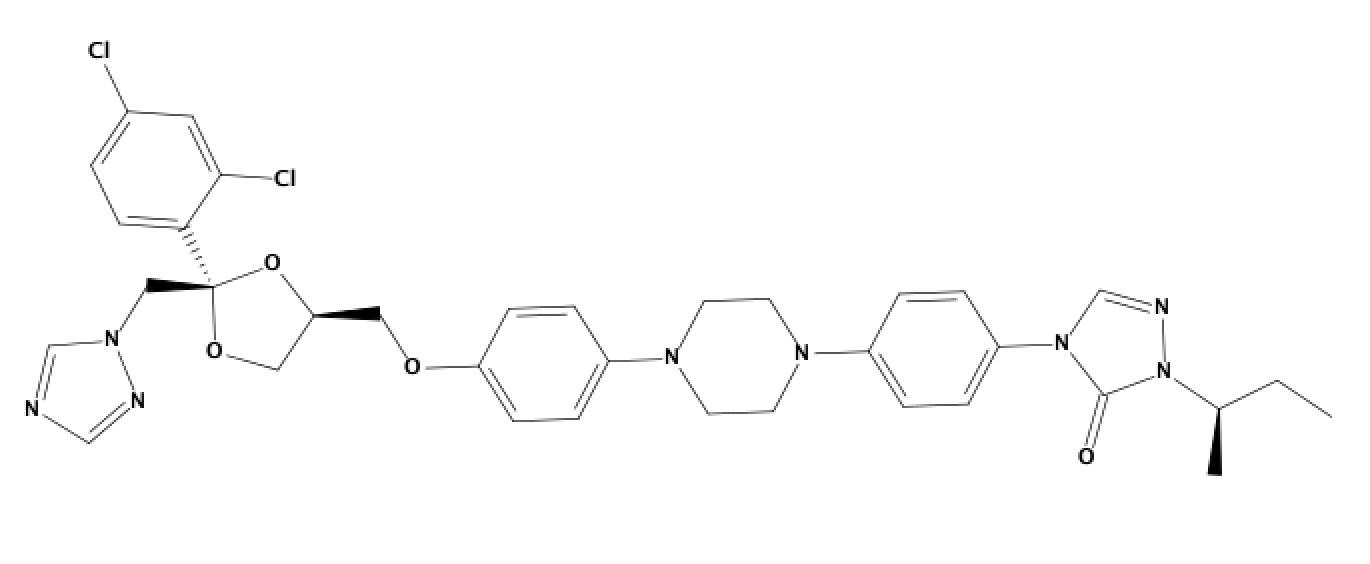  Itraconazole | |
| 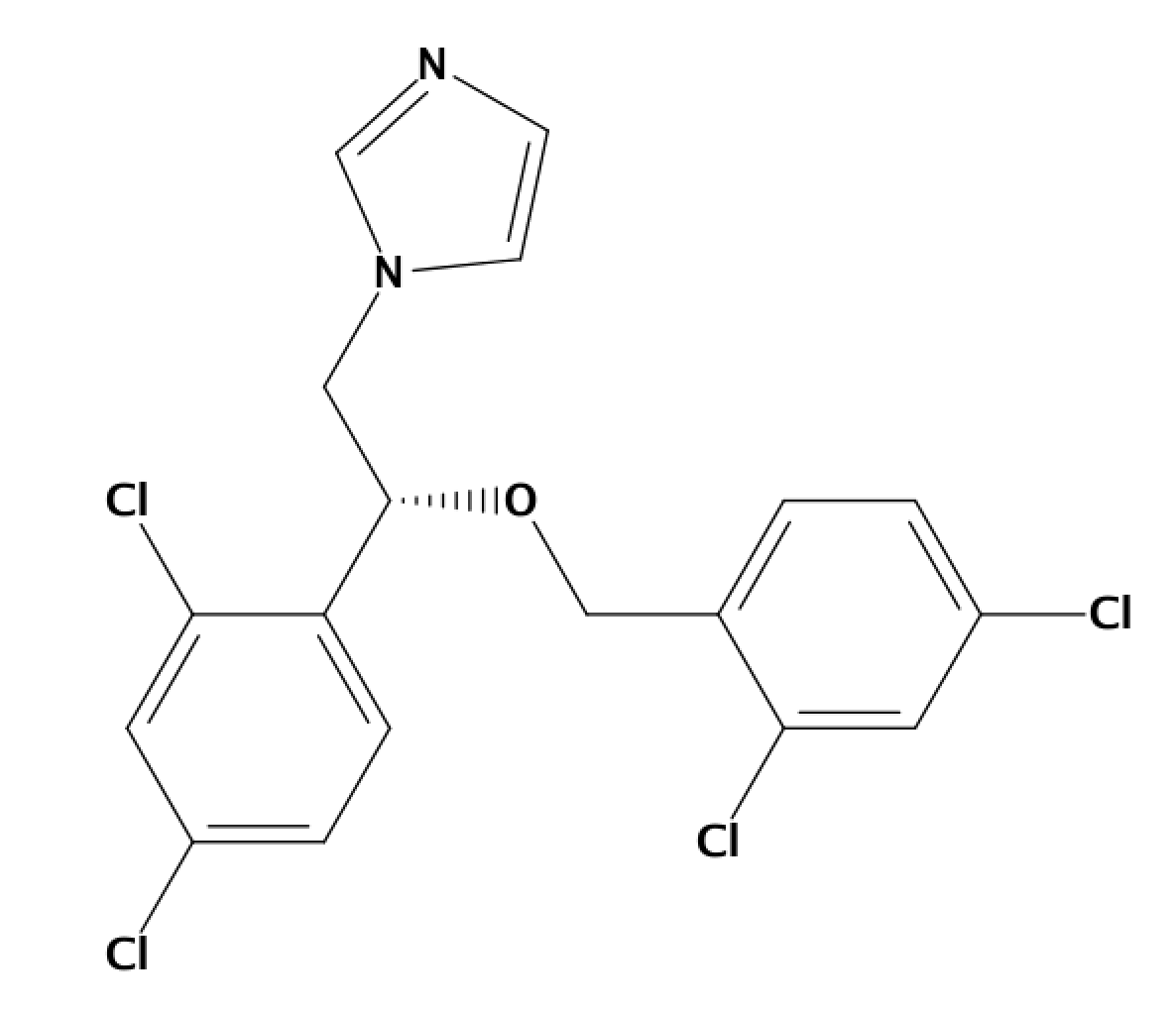  Miconazole | 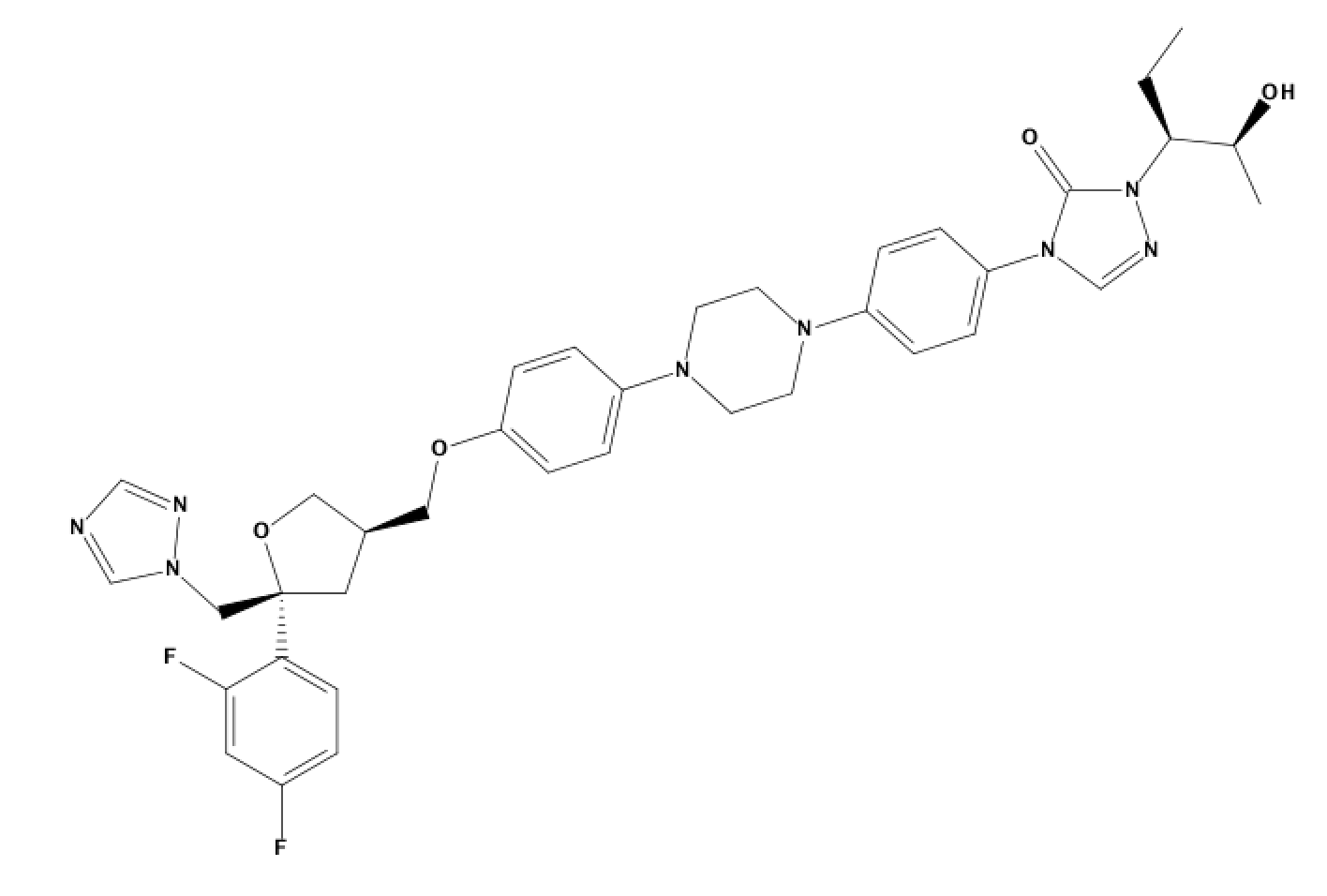  Posaconazole | | |
| 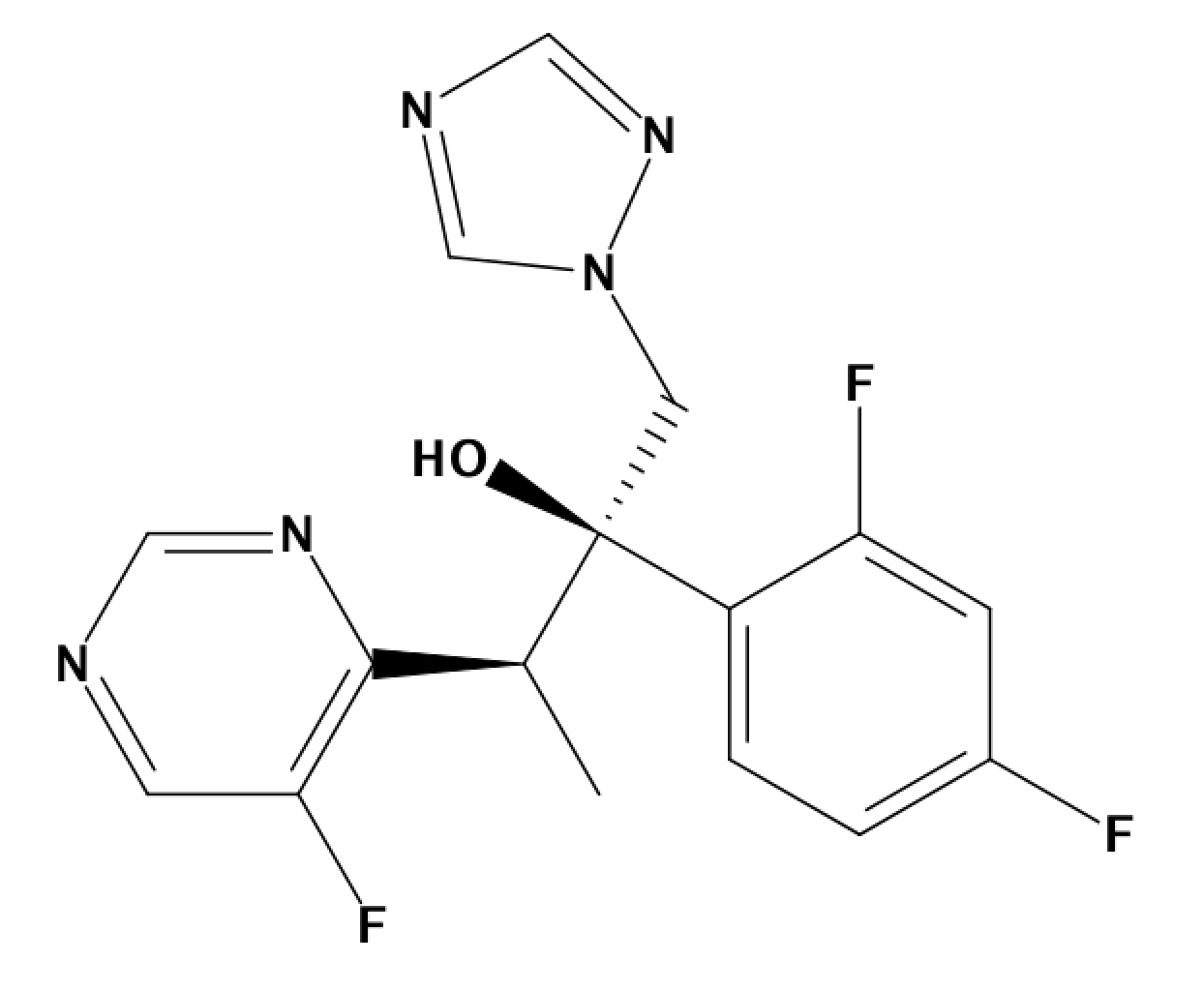  Voriconazole |  |  |  |
| 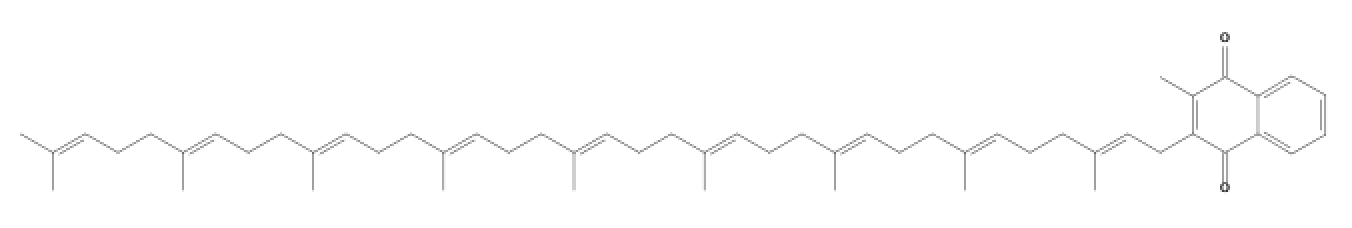  Menaquinone 9 | | | |

Figure S3. RMSD *vs* time during MD simulations of MK9-CYP128A1 complexes (colors are explained in the legend of each figure)


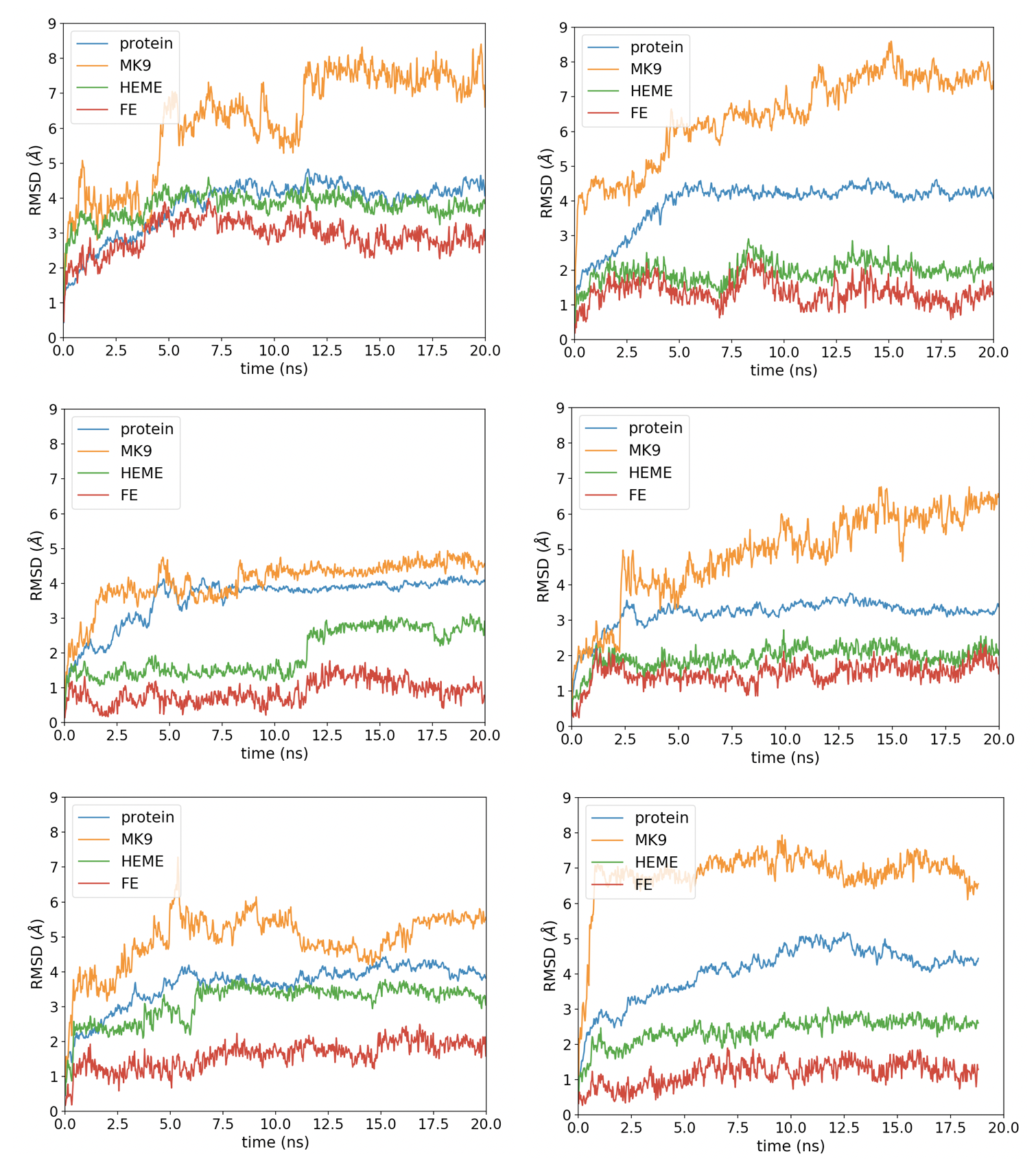

Supplement: Supplementary file 1 [file ijms-21-04816-s001.zip › Supplementary Information/Supplementary Information.docx]
