# Supplementary figures and images for: Comparative Analysis, Structural Insights, and Substrate/Drug Interaction of CYP128A1 in Mycobacterium tuberculosis
Source: Int J Mol Sci. 2020 Jul 8;21(14):4816. doi: 10.3390/ijms21144816 (PMC7404182; doi:10.3390/ijms21144816)

0.1

# Taxonmy

- MTBC
- NTM
- MCAC
- MAC
- SAP

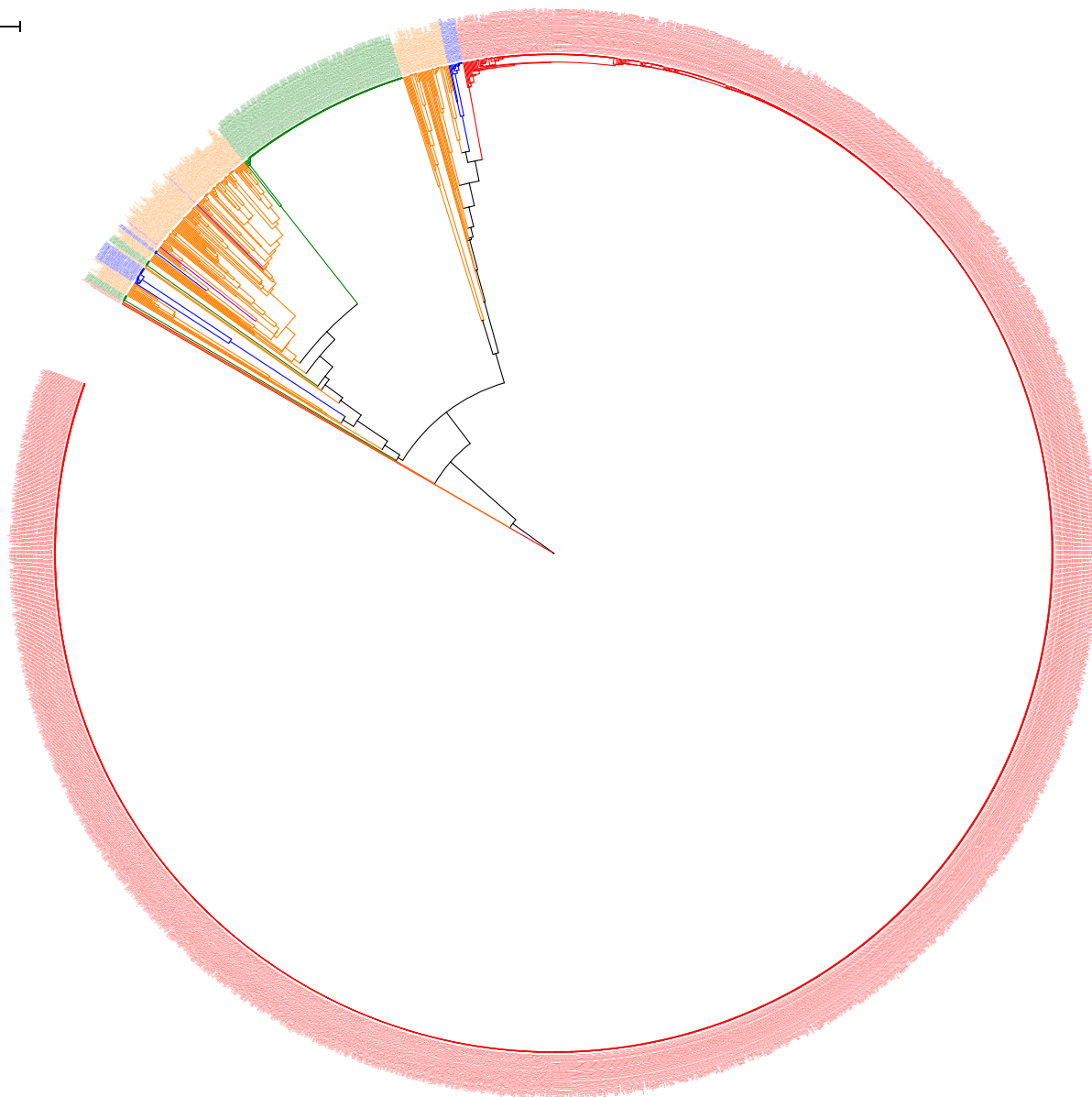

Supplement: Supplementary file 1 [file ijms-21-04816-s001.zip › Supplementary Information/Supplementary Dataset 3.pdf]
